# Supplementary figures and images for: Detection of gene cis-regulatory element perturbations in single-cell transcriptomes
Source: PLoS Comput Biol. 2021 Mar 12;17(3):e1008789. doi: 10.1371/journal.pcbi.1008789 (PMC8011753; doi:10.1371/journal.pcbi.1008789)

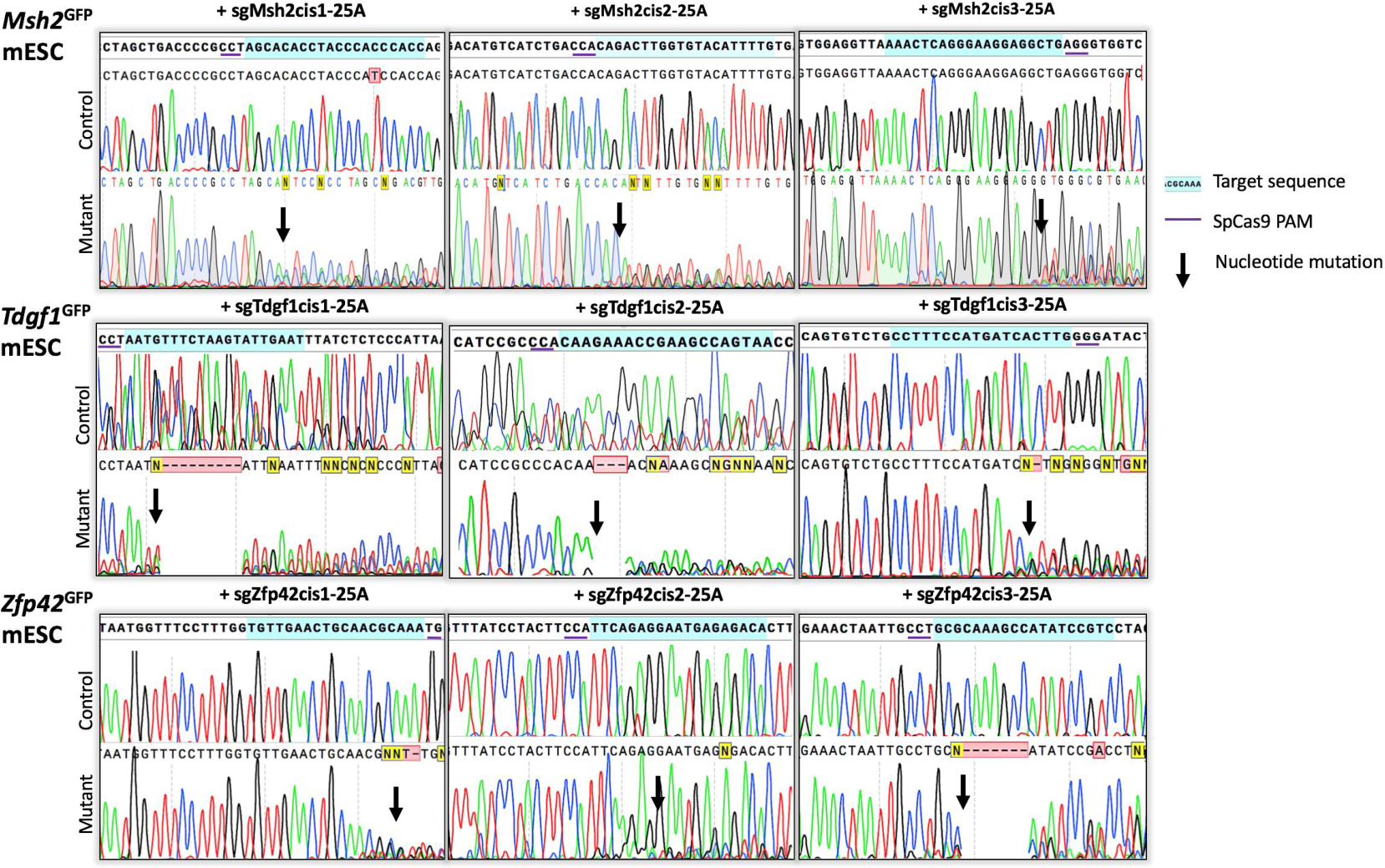

Supplement: S1 Fig — Sanger sequencing analysis of nine 25A-gRNAs targeting cis-regulatory regions of Msh2, Tdgf1 and Zfp42 exhibits consistently robust mutagenic activity. (TIF) [file pcbi.1008789.s001.tif]

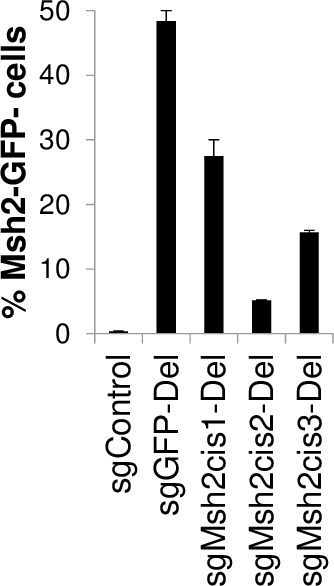

Supplement: S2 Fig — Flow cytometric percentage of Msh2-GFP- cells after targeting with a pair of gRNAs in the GFP ORF (sgGFP- Del) or flanking each of three Msh2 regions detected as hits in the MERA screen. Enrichment of %Msh2-GFP- cells is significant for all non-control gRNAs with respect to sgControl by one-sided t-test (p-value < 0.05). (TIF) [file pcbi.1008789.s002.tif]

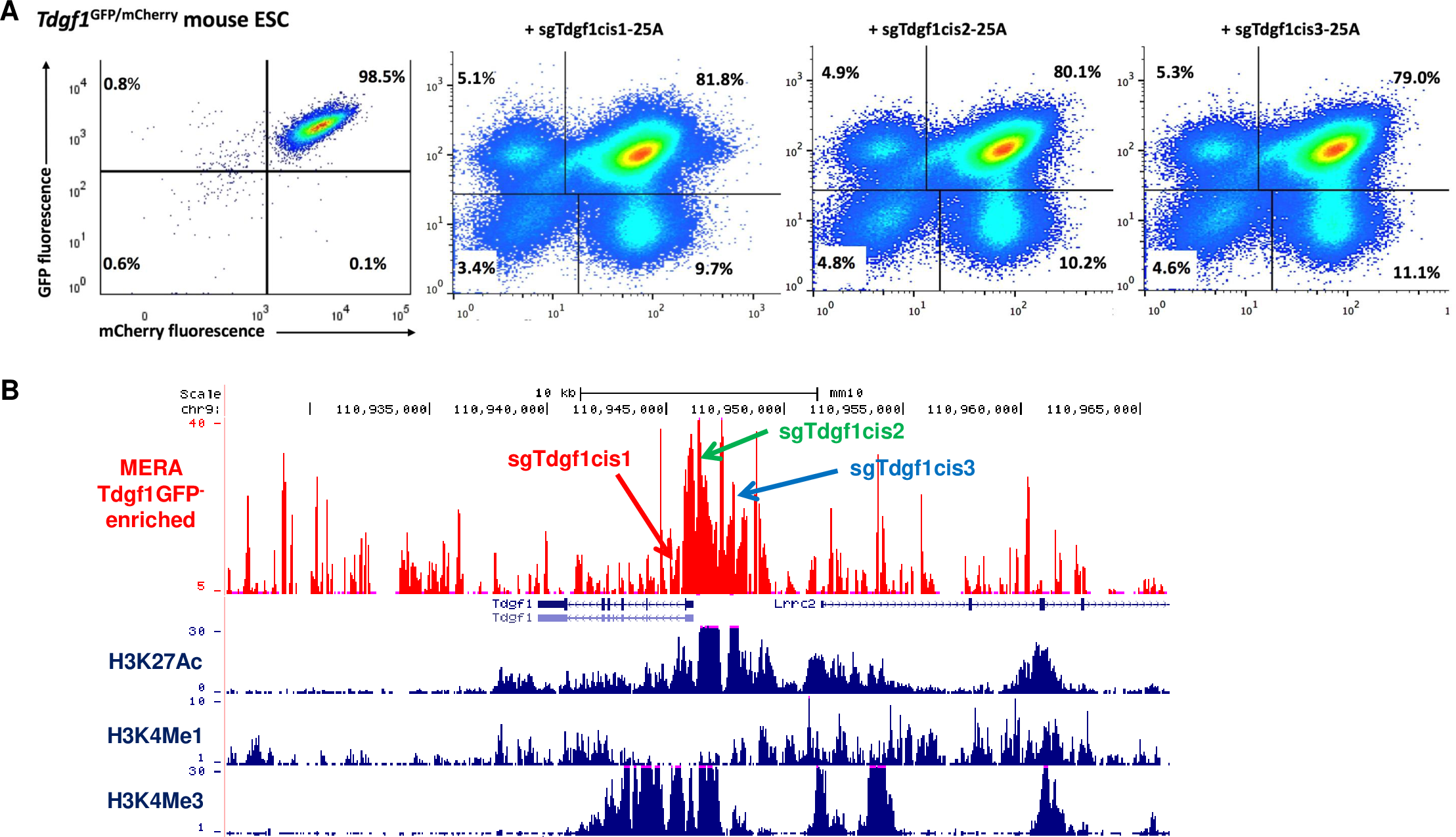

Supplement: S3 Fig — (A) Flow cytometry of Tdgf1GFP/mCherry cells shows uniformly strong bi-allelic fluorescence. (B) Tdgf1 MERA GFP- enrichment of ~4,000 cis-gRNAs from Rajagopal et al study, highlighting locations of cis-gRNAs used in this work. (C) Flow cytometry of Tdgf1GFP/mCherry cells after sgTdgfcis1-3 targeting, showing robust fluorescence loss but rare bi-allelic expression loss. (TIF) [file pcbi.1008789.s003.tif]

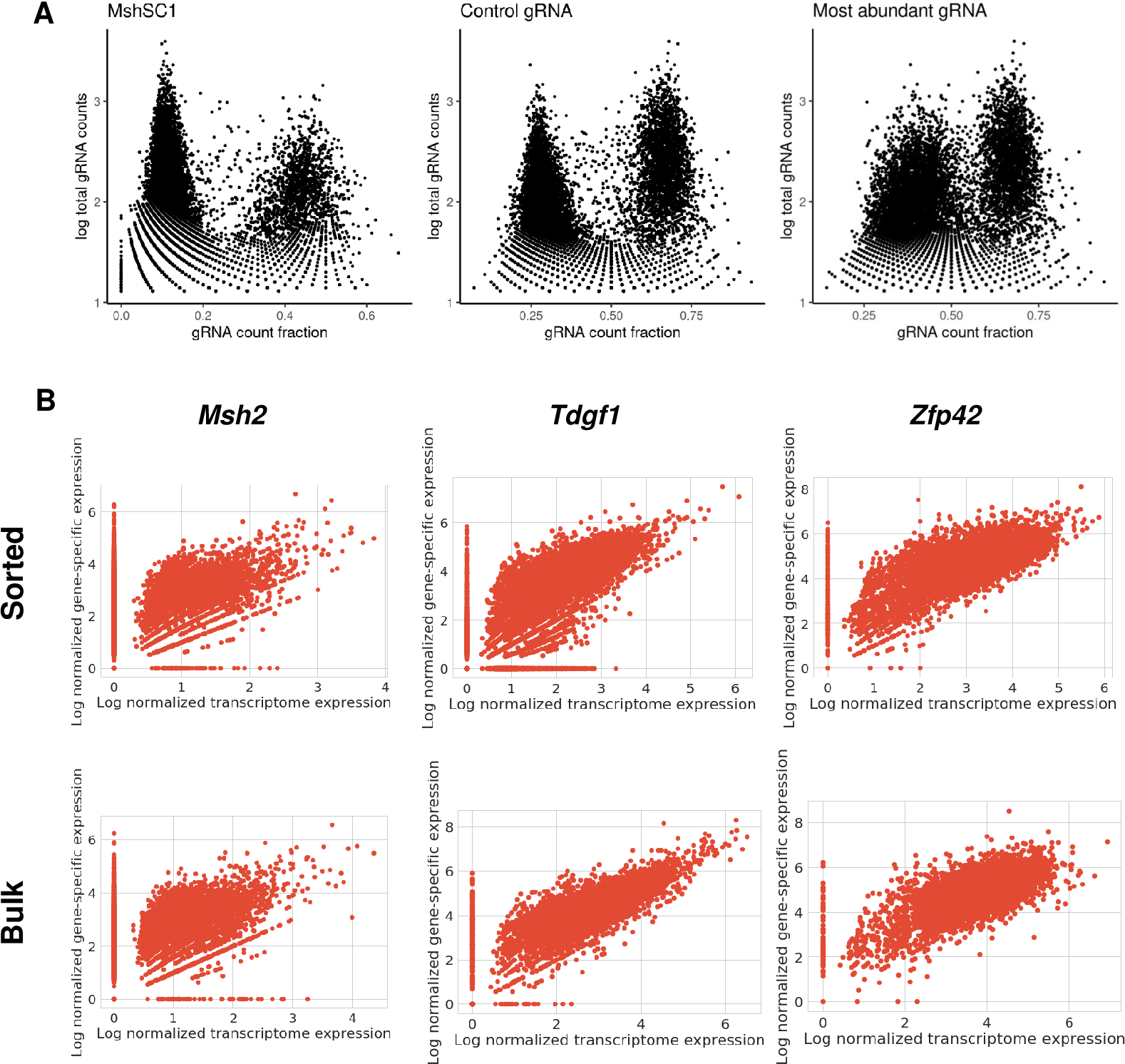

Supplement: S4 Fig — (A) gRNA count fraction vs. log total gRNA counts in each cell in the sorted population for MshSC1, the control gRNA, as well as the most abundant gRNA in each cell (B) PCR-based enrichment of specific transcripts increases UMI-unique reads without skewing relative abundance. Normalized UMI-unique transcriptome expression (X-axis) and gene-specific expression (Y-axis) of Msh2 (left), Tdgf1 (center), and Zfp42 (right) in the sorted (top) and unsorted (bottom) experiments. (TIF) [file pcbi.1008789.s004.tif]

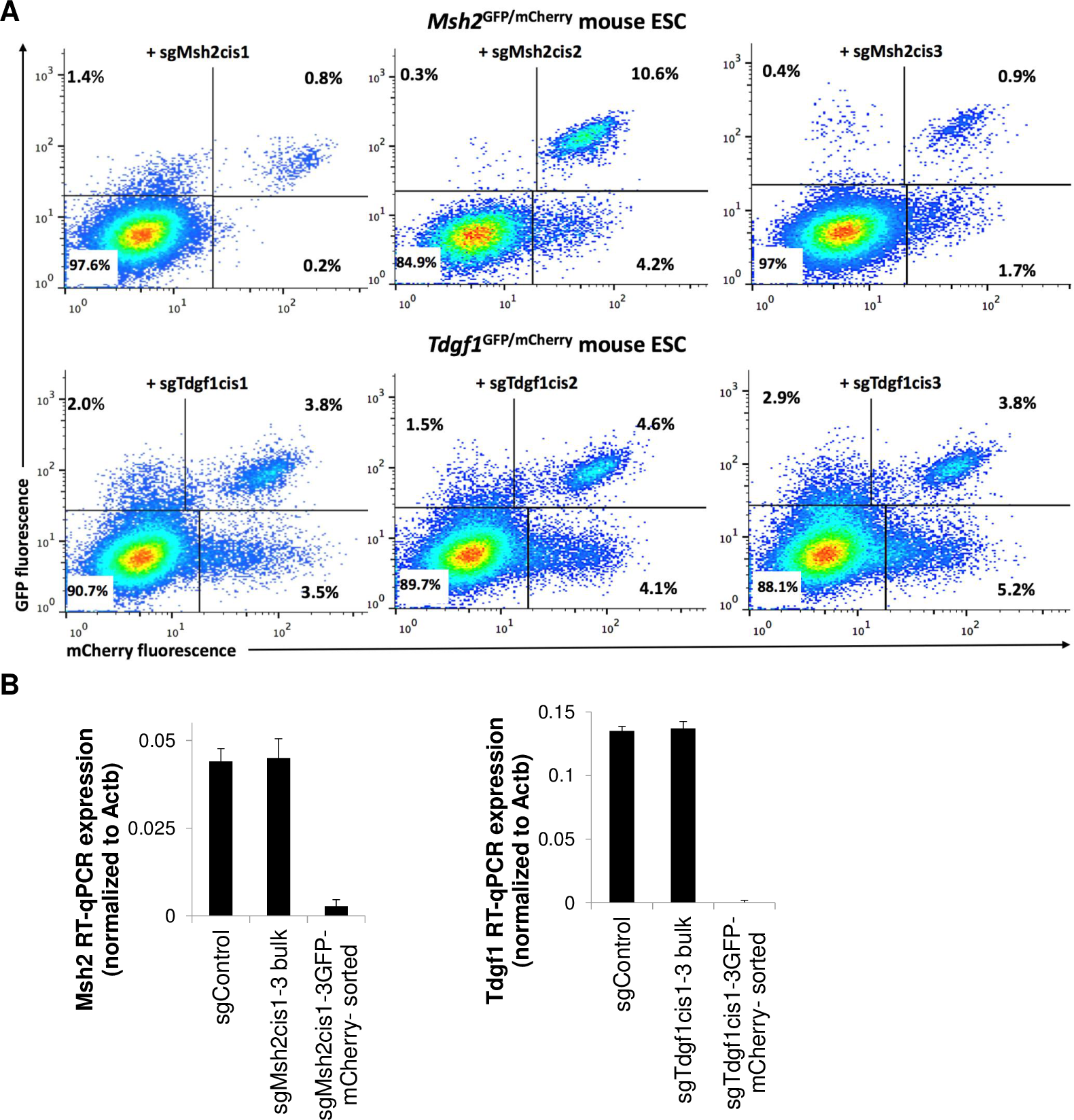

Supplement: S5 Fig — (A) Flow cytometry plots showing purity of 25A-gRNA-targeted and double GFP-mCherry- flow cytometrically purified populations. All populations are >85% pure, although single and double positive subpopulations due to imperfect sorting and/or re-expression of transgenes after sorting. (B) RT-qPCR expression of Msh2 (left plot) and Tdgf1 (right plot) in control gRNA-targeted (left), bulk cis-gRNA targeted (middle), and GFP-mCherry- double-sorted (right) populations, showing strong flow cytometric enrichment of cells lacking target gene expression. (TIF) [file pcbi.1008789.s005.tif]

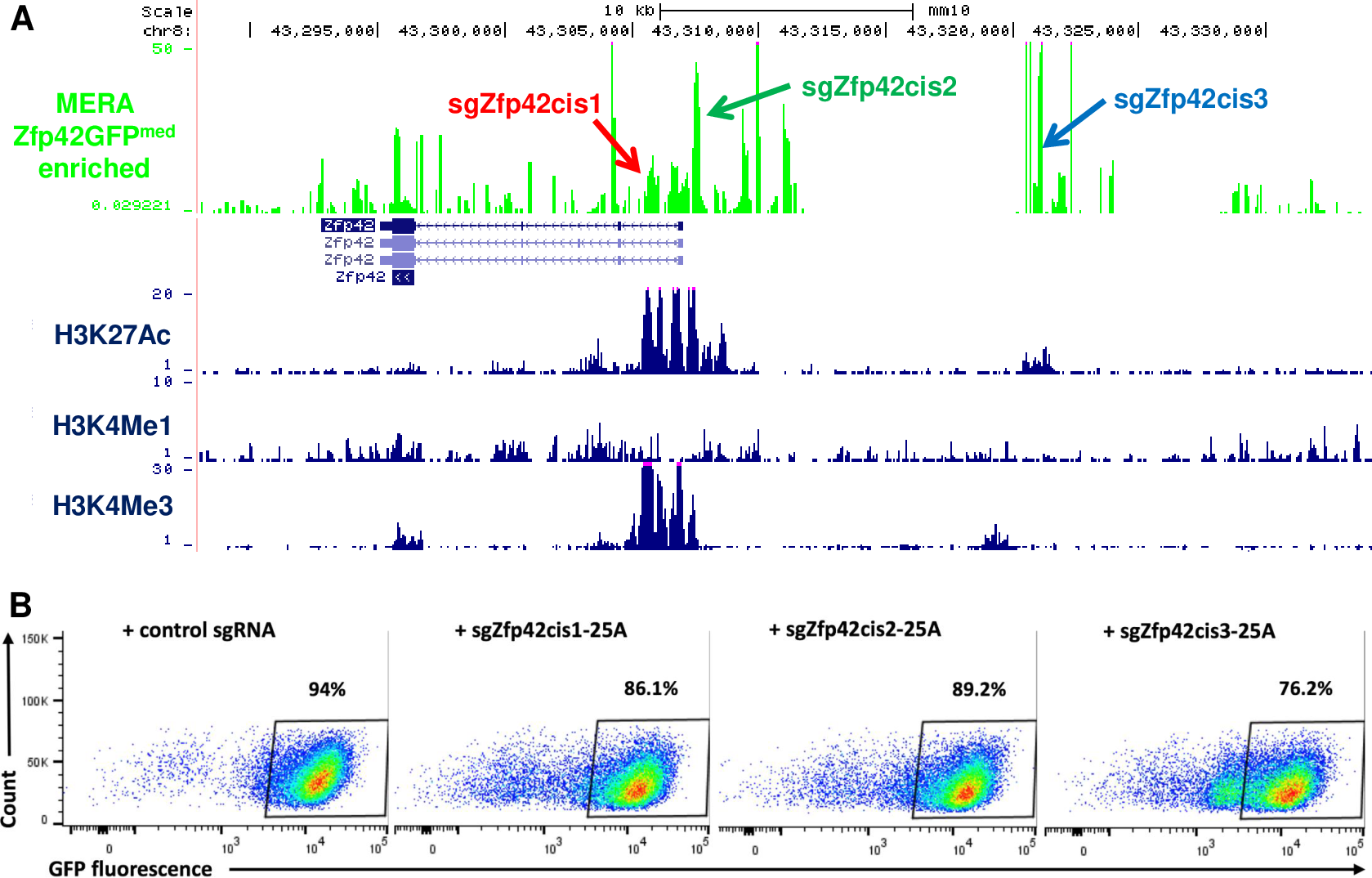

Supplement: S6 Fig — (A) Zfp42 MERA GFP- enrichment of ~4,000 cis-gRNAs from Rajagopal et al study, highlighting locations of cis-gRNAs used in this work. (B) Flow cytometry of Zfp42GFP cells after sgZfp42cis1-3 targeting, showing robust fluorescence loss. (TIF) [file pcbi.1008789.s006.tif]

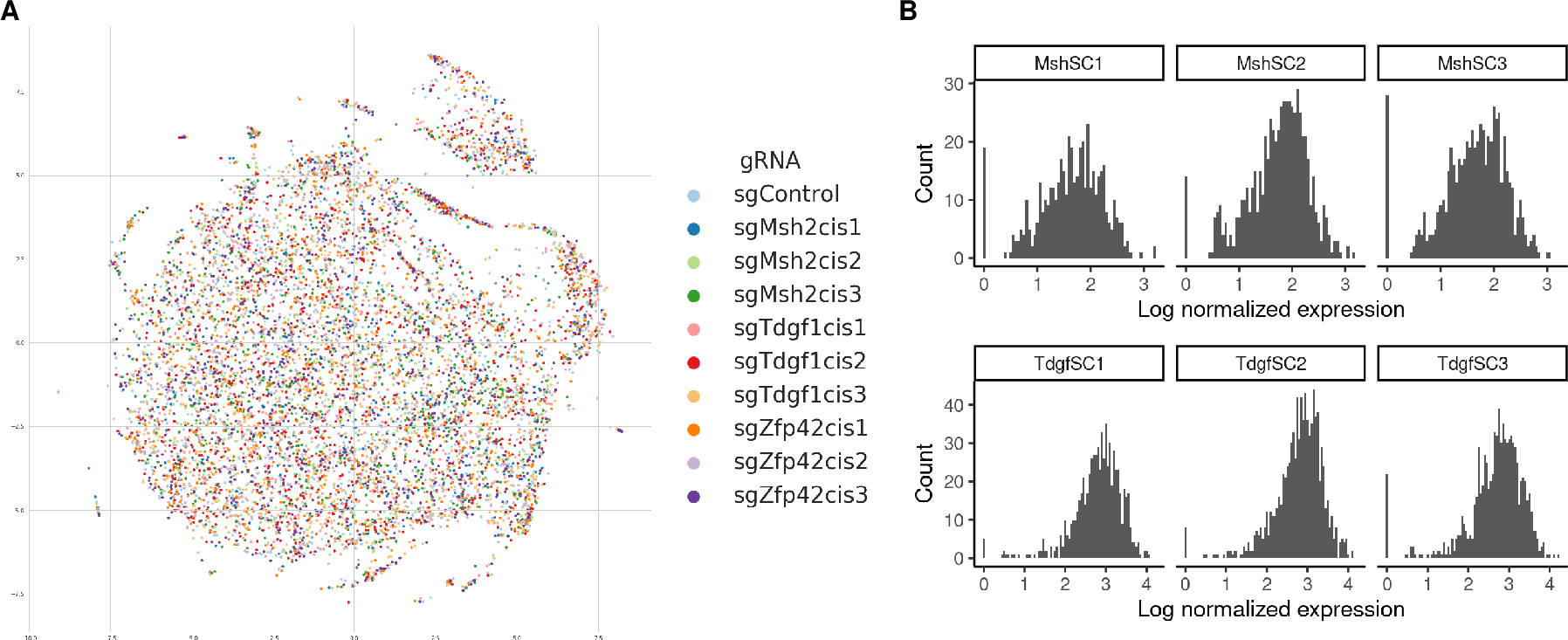

Supplement: S7 Fig — (A) t-SNE plot of wildtype pAC-Seq experiment labeled by gRNA species. t-SNE plot of the wildtype experiment showing cells colored by the gRNA they received. There is no clear separation of cells based on gRNA expression, as would be expected given that cis-gRNAs have subtle effects on the transcriptome. (B) Distribution of log-normalized expression of Msh2 and Tdgf1 in cells receiving Msh2-targeting gRNAs (above) and Tdgf1-targeting gRNAs (below) with transcript-targeted sequencing in wildtype experiment. (TIF) [file pcbi.1008789.s007.tif]

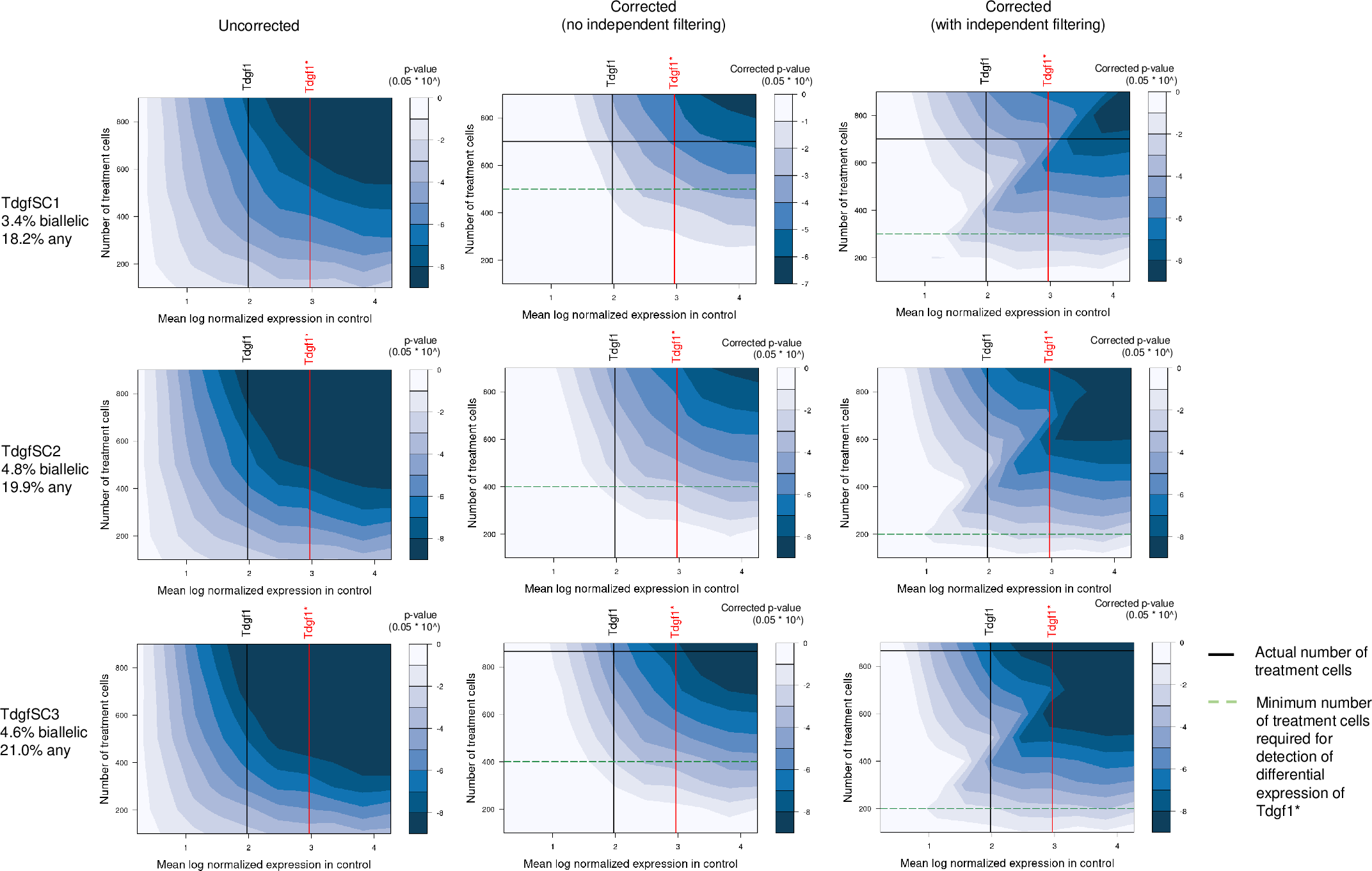

Supplement: S8 Fig — Contour maps depicting raw (left) and adjusted (middle, right) p-values for detecting down-regulation of target gene given fraction of monoallelic and biallelic loss for Tdgf1. p-values are calculated by simulating partial and full loss of genes within each gene bucket corresponding to the observed monoallelic and biallelic loss for the given number of treatment cells, and then performing differential expression via Wilcoxon rank sum. p-values are adjusted either for all genes tested (middle), or for the set of genes with baseline mean expression above the gene with the lowest baseline mean expression in that bucket, i.e. after independent filtering (right). Vertical lines indicate base expression of genes in control population with (red) and without (black) targeted sequencing. Black horizontal lines indicate the actual number of treatment cells observed, while horizontal green dashed lines indicate the minimum number of cells required to achieve significance at corrected p-value < 0.05 to detect differential expression of Tdgf1 with transcript-targeted sequencing. (TIF) [file pcbi.1008789.s008.tif]

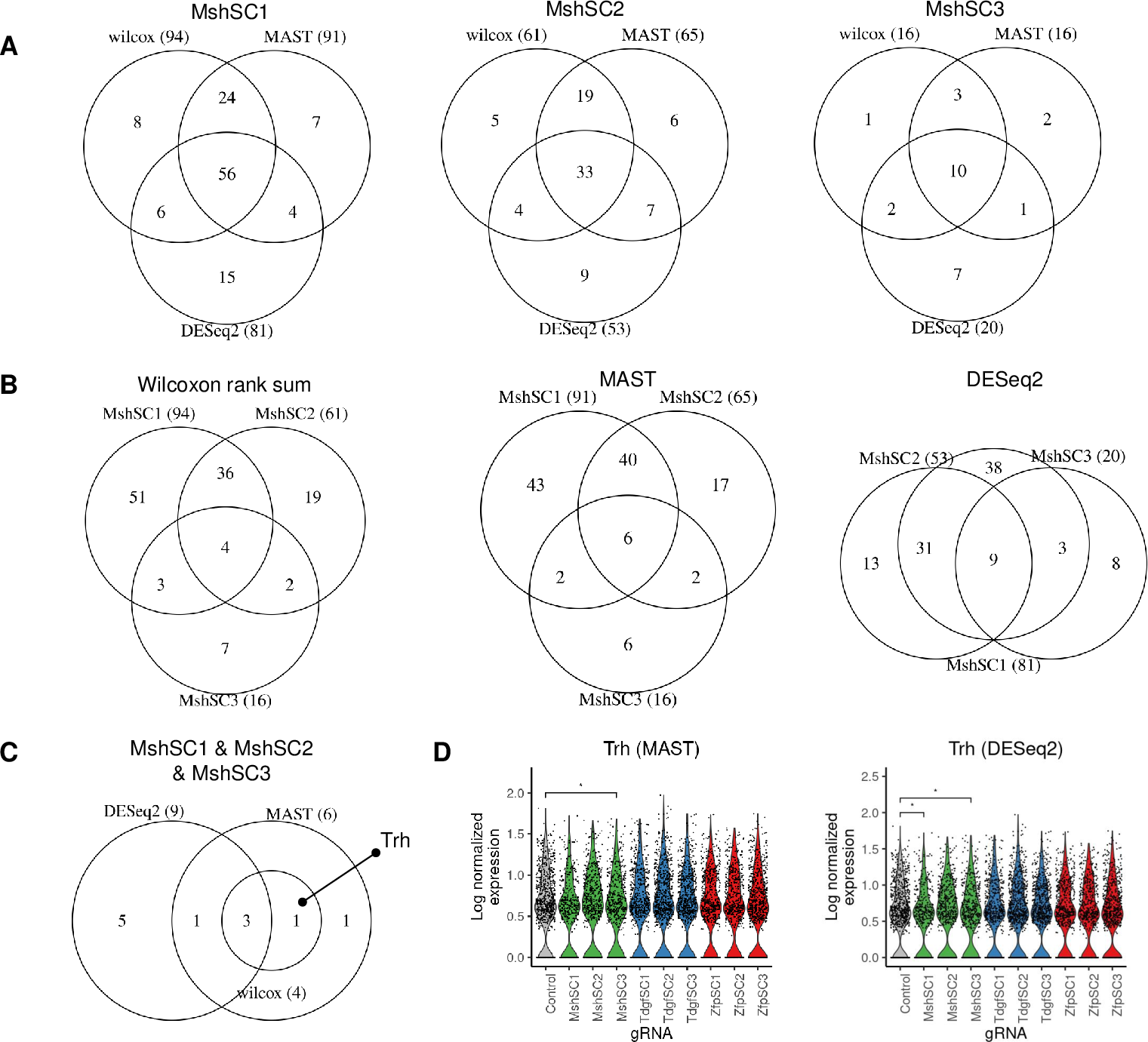

Supplement: S9 Fig — (A) Overlap across different differential expression methods for each Msh2-targeting gRNA in the sorted population. (B) Overlap across Msh-targeting gRNAs for each differential expression method in the sorted population. (C) Overlap across different differential expression methods for consistently differentially expressed genes identified across Msh2-targeting gRNAs in the sorted population. (A-C) Numbers in parenthesis indicate the number of differentially expressed genes identified at adjusted p-value < 0.05 (D) Trh was found to be differentially expressed for MshSC3 across all methods, and for MshSC1 using DESeq2 (indicated by *, adjusted p-value < 0.05) in the unsorted population. (TIF) [file pcbi.1008789.s009.tif]

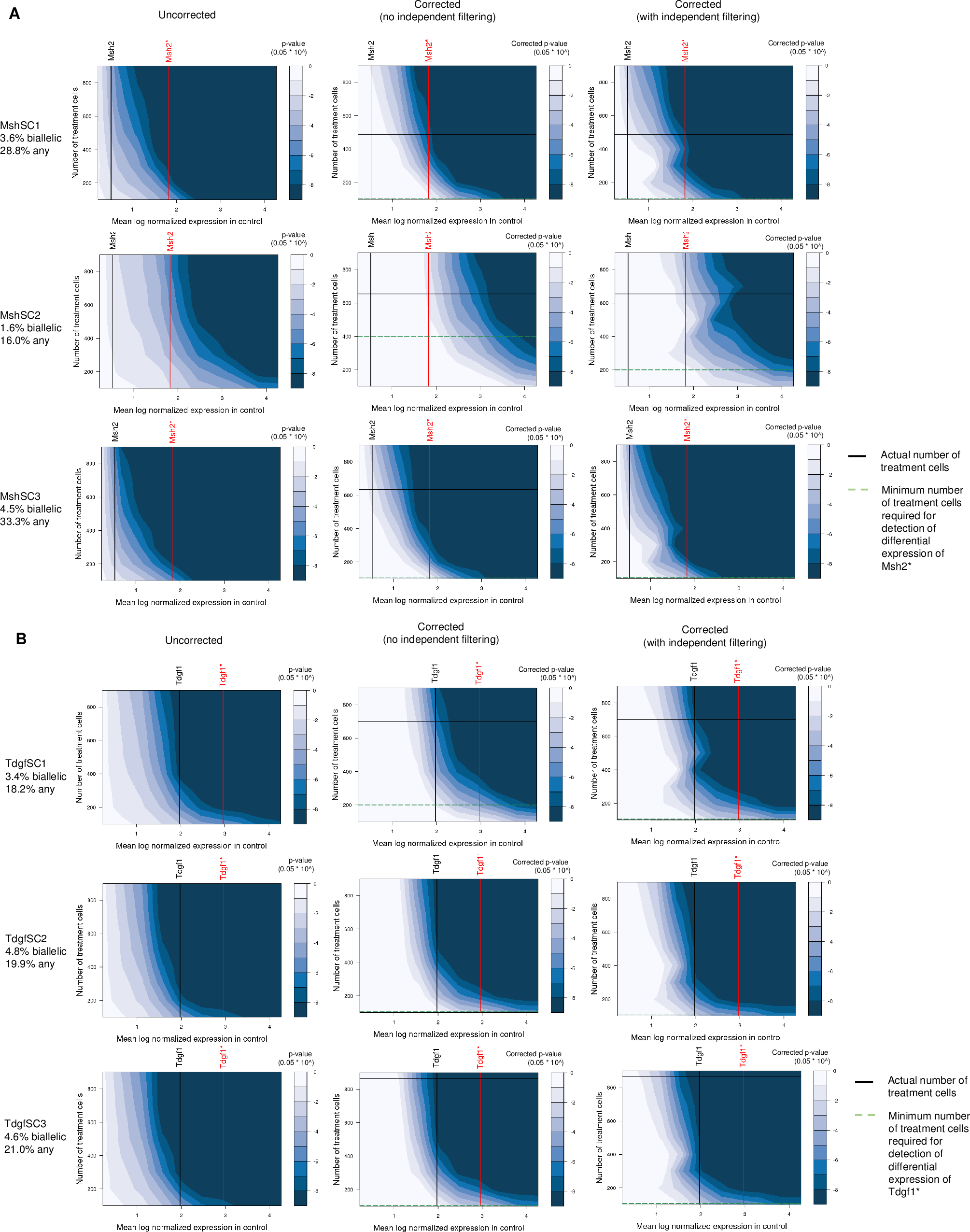

Supplement: S10 Fig — Contour maps depicting raw (left) and adjusted (middle, right) p-values for detecting down-regulation of target gene given fraction of monoallelic and biallelic loss for (A) Msh2 and (B) Tdgf1. p-values are calculated by simulating partial and full loss of genes within each gene bucket corresponding to the observed monoallelic and biallelic loss for the given number of treatment cells, and then performing differential expression via MAST. p-values are adjusted either for all genes tested (middle), or for the set of genes with baseline mean expression above the gene with the lowest baseline mean expression in that bucket, i.e. after independent filtering (right). Vertical lines indicate base expression of genes in control population with (red) and without (black) targeted sequencing. Black horizontal lines indicate the actual number of treatment cells observed, while horizontal green dashed lines indicate the minimum number of cells required to achieve significance at corrected p-value < 0.05 to detect differential expression with transcript-targeted sequencing. (TIF) [file pcbi.1008789.s010.tif]
