# Supplementary material for: Detection of gene cis-regulatory element perturbations in single-cell transcriptomes
Source: PLoS Comput Biol. 2021 Mar 12;17(3):e1008789. doi: 10.1371/journal.pcbi.1008789 (PMC8011753; doi:10.1371/journal.pcbi.1008789)
Supplement: S1 Table — (PDF) [file pcbi.1008789.s011.pdf]

| gRNA sequence | gRNA start (chr17) | gRNA end (chr17) | GFP- enrichment normalized to bulk counts |
|---|---|---|---|
| GTCTAGCCTTTCAGCTTGCTC | 87525307 | 87525329 | 0.02 |
| GCAGTCCCAGAGCAAGCTGAA | 87525312 | 87525334 | 0.40 |
| GCTCTGGGACTGAGGTGTGCC | 87525324 | 87525346 | 0.01 |
| GGCTTTGAACAAAGTTGTCC | 87525342 | 87525364 | 0.01 |
| GCCTTACTGAAGTCTGAAGTC | 87525363 | 87525385 | 0.01 |
| GAAAACTCCCTGCCTACCCTG | 87525386 | 87525408 | 0.06 |
| GAGGCACCTCAGGGTAGGCA | 87525392 | 87525414 | 0.69 |
| GTCACAGAGGCACCTCAGGGT | 87525397 | 87525419 | 0.08 |
| GCCTCTCACAGAGGCACCTC | 87525402 | 87525424 | 0.57 |
| GCCTCTGTGAGAGGCAGTGC | 87525410 | 87525432 | 3.03 |
| GTGAGAGGCAGTGCTGGCAG | 87525416 | 87525438 | 0.01 |
| GCAGTGCTGGCAGAGGAGCT | 87525423 | 87525445 | 1.26 |
| GTATCTTAAGTTCTGAGATC | 87525472 | 87525494 | 0.01 |
| GAAGATACTTCATTTACAAAA | 87525488 | 87525510 | 0.01 |
| GCTCTAGAGAGAAGAAGAAC | 87525512 | 87525534 | 0.14 |
| GTCTTCTTCTCTCTAGAGCAG | 87525517 | 87525539 | 0.02 |
| GCAGAGGTTTGCAACCGTG | 87525533 | 87525554 | 0.58 |
| GCTTGGGGGTCACCACCGCA | 87525547 | 87525569 | 0.00 |
| GCAACAAAAATAACTGTGCT | 87525564 | 87525586 | 0.02 |
| GTTTTTGTTGCTACTTAACAA | 87525577 | 87525599 | 0.01 |
| GATGGTCGTTTTGCTACTGTT | 87525596 | 87525618 | 0.05 |
| GCCAAGTCCGTTGGAAAACAC | 87525635 | 87525657 | 0.01 |
| GCAGGGGTCACCCAAGTCCGT | 87525645 | 87525667 | 0.01 |
| GACTTGGGTGACCCCTGTGAG | 87525652 | 87525674 | 0.01 |
| GTTGAAGGACCCTCTCACAG | 87525662 | 87525684 | 0.03 |
| GACTTCTTTGGGGGGTTGA | 87525677 | 87525698 | 0.03 |
| GTCATTGTGACTTCTTTGGG | 87525684 | 87525706 | 0.11 |
| GAAGTCACAATGACAGGTAGT | 87525694 | 87525716 | 0.24 |
| GTTGCTCTTACTCTAGGGTAG | 87525720 | 87525742 | 0.02 |
| GTTTTATTGCTCTTACTCTA | 87525726 | 87525748 | 0.02 |
| GAAGAGCAATAAAACATAATA | 87525735 | 87525757 | 0.04 |
| GTTTGTTGAGTGTCTTCAGT | 87525783 | 87525805 | 0.01 |
| GCAACAAACAAAACAAACCAG | 87525798 | 87525820 | 0.60 |
| GATTGTCAGGGAGGGACCGC | 87525814 | 87525836 | 0.09 |
| GCTTGAGAAGATTGTCAGGGA | 87525822 | 87525844 | 0.01 |
| GTGCCCTTGAGAAGATTGTC | 87525827 | 87525849 | 0.35 |
| GCTGATTCACCCAGCTTGCCT | 87525861 | 87525883 | 0.01 |
| GCTGATATCCCAAGGCAAGCT | 87525869 | 87525891 | 0.01 |
| GTGGACTATCTGATATCCCA | 87525878 | 87525900 | 0.01 |
| GTTTACACAAGGAATACTATG | 87525897 | 87525919 | 0.01 |
| GTCCTTGTGTAAATGGATGCT | 87525908 | 87525930 | 0.02 |
| GTTCCTAACAAGCCTTCTTT | 87525931 | 87525953 | 0.01 |
| GACCGAGGGAGTCCTAAAGA | 87525943 | 87525965 | 0.02 |
| GGCAAATCTTTCCAGACCGA | 87525957 | 87525979 | 0.56 |
| GTCTGGAAAGATTTGCCTGT | 87525963 | 87525985 | 0.01 |
| GTTGCCTGTTGGGGCCTCTCA | 87525975 | 87525997 | 0.49 |
| GCTTCTGGGTGTTTCCTTGAG | 87525988 | 87526010 | 0.01 |
| GAGACACTATGGGACTCTTC | 87526004 | 87526026 | 0.01 |
| GAAGGAAAGGGGAGACACTA | 87526015 | 87526037 | 0.01 |
| GCCCTTCTAGAAGGAAGGAA | 87526028 | 87526050 | 0.03 |
| GGTCTTCCGGCCCTTCTAGA | 87526037 | 87526059 | 0.07 |

| | | | |
|---|---|---|---|
| GGCCGGAAGACCTGGATACC | 87526048 | 87526070 | 0.62 |
| GAAGACCTGGATACCTGGAG | 87526053 | 87526075 | 0.31 |
| GTTTCCCCTCTCCAGGTATCC | 87526058 | 87526080 | 0.62 |
| GAAAATCCTTTCCCCTCTCC | 87526066 | 87526088 | 0.01 |
| GCGCCACCACACCAGGCCTA | 87526346 | 87526368 | 0.05 |
| GAACAATCCTTAGGCCTGGTG | 87526352 | 87526374 | 0.03 |
| GTAGGAAACAATCCTTAGGCC | 87526357 | 87526379 | 0.06 |
| GTTATTTAGGAAACAATCCTT | 87526362 | 87526384 | 0.02 |
| GAGGGACATTGTCTTTATTT | 87526376 | 87526398 | 0.28 |
| GAAATGGGGGACAGACGCTG | 87526395 | 87526417 | 0.11 |
| GCAAGGGGAGGGACTGAAAT | 87526410 | 87526432 | 0.01 |
| GAAGCATTGCTAAGCAAGGGG | 87526422 | 87526444 | 0.02 |
| GTTTCAAGCATTGCTAAGCA | 87526427 | 87526449 | 0.01 |
| GCAATGCTTGAAACTGCATT | 87526436 | 87526458 | 0.01 |
| GTTGTACAAAGCATGAGAAAG | 87526459 | 87526481 | 0.02 |
| GAAAGCATGAGAAAGAGGTCT | 87526465 | 87526487 | 0.01 |
| GCACCTCCCCCCGTAAGAATT | 87526488 | 87526510 | 0.01 |
| GCCCCTCCCAAATTCTTACG | 87526495 | 87526517 | 0.60 |
| GGGAGGGGCAATTTGTCCTT | 87526509 | 87526531 | 0.00 |
| GACTGGAACATATCACTCCTA | 87526525 | 87526547 | 0.01 |
| GATATGTTCCAGTCAGACCT | 87526535 | 87526557 | 0.01 |
| GTTCCAGTCAGACCTAGGGAG | 87526541 | 87526563 | 0.01 |
| GCTTTAAAGAATCCACTCCCT | 87526552 | 87526574 | 0.15 |
| GGATTCTTTAAAGTTAAAAT | 87526562 | 87526584 | 0.02 |
| GAATATTAGAGCTTTAGAGCC | 87526591 | 87526613 | 0.02 |
| GAAAGGAAGCATCATCATGCC | 87526609 | 87526631 | 0.01 |
| GTCCTAGGTGTTGGTATTAA | 87526627 | 87526649 | 0.03 |
| GACTCAGTGTCCTAGGTGT | 87526636 | 87526657 | 0.56 |
| GAAATTCTGACTCAGTGTCCT | 87526642 | 87526664 | 0.01 |
| GTCAAGACCTGCCTGGGCTGC | 87526670 | 87526692 | 0.01 |
| GGCTATCCTGCAGCCCAGGC | 87526676 | 87526698 | 0.02 |
| GAATATTTTTATTTTGGAAAC | 87526697 | 87526719 | 0.02 |
| GTTTAATAAATATTTTTATTT | 87526704 | 87526726 | 0.10 |
| GAATATTTATTAAATAGCACA | 87526714 | 87526736 | 0.06 |
| GCACATGGAAGTTAAGACT | 87526729 | 87526750 | 0.00 |
| GTAGTTAGCTAAACTTCTGAA | 87526755 | 87526777 | 0.02 |
| GTTCTGAATGGGACCTTCTAC | 87526768 | 87526790 | 0.01 |
| GCCACTCCTCTGGCCTGTAGA | 87526780 | 87526802 | 1.58 |
| GACTGTTGGCTTCCACTCCTC | 87526791 | 87526813 | 0.10 |
| GTGTTTCAGATCTAACTGT | 87526806 | 87526827 | 0.01 |
| GACAAAACACACCATTATTCC | 87526826 | 87526848 | 0.03 |
| GAGTGGGGTCCCTGGAATAA | 87526836 | 87526858 | 0.01 |
| GGGACCCCACTCTCATCACG | 87526847 | 87526869 | 0.14 |
| GCAGCTCCCCGTGATGAGAGT | 87526852 | 87526874 | 0.02 |
| GATCACTTTTGTCTGTGTC | 87526877 | 87526898 | 0.01 |
| GTCTGTGTCAGGTCATTCAGC | 87526888 | 87526910 | 0.02 |
| GTCAGGTCATTCAGCAGGCC | 87526893 | 87526915 | 0.00 |
| GATTCAGCAGGCCTGGTTGTC | 87526901 | 87526923 | 0.50 |
| GCTCAGCTATCCAGACAACC | 87526911 | 87526933 | 0.06 |
| GATGTGCCAATGATAGCATTC | 87526944 | 87526966 | 0.00 |
| GATAACTTTTGAGTGTCTTT | 87526968 | 87526990 | 0.02 |
| GCAAAAGTTATCTGTTAGATC | 87526980 | 87527002 | 0.01 |

| | | | |
|---|---|---|---|
| GTATCTGTTAGATCAGGCTGT | 87526987 | 87527009 | 0.02 |
| GTAGATCAGGCTGTCGGTGCC | 87526994 | 87527016 | 0.01 |
| GGCTGTCGGTGCCAGGTCT | 87527001 | 87527022 | 0.01 |
| GAAGGGCTGGGCCTAGACC | 87527012 | 87527033 | 0.10 |
| GTATCATTATGAGAAGGGCT | 87527023 | 87527045 | 9.66 |
| GCCTGGGTATCATTATGAGAA | 87527028 | 87527050 | 0.01 |
| GATGGAGATTTGTGATTTCC | 87527046 | 87527068 | 0.01 |
| GTATTTGGTATATATTTTAGA | 87527064 | 87527086 | 0.03 |
| GGGATATATTTTATATATT | 87527080 | 87527101 | 0.10 |
| GAAATATATCCCAAGACTGTA | 87527091 | 87527113 | 0.02 |
| GGGCTTTTCCATACAGTCTT | 87527099 | 87527121 | 0.02 |
| GCATTCTTTCTATGATCATTT | 87527119 | 87527141 | 0.00 |
| GTGAATACGATATTTACCCA | 87527151 | 87527173 | 0.00 |
| GCACTTTGGAGTGTCTTCCTT | 87527167 | 87527189 | 0.01 |
| GCACTCCAAAGTGACCCACAG | 87527178 | 87527200 | 0.53 |
| GACCCACAGAGGTCCTCCTC | 87527189 | 87527211 | 0.99 |
| GCAGAGGTCCTCCTCTGGTCT | 87527195 | 87527217 | 0.01 |
| GGAAGACCCAAGACCAGAGG | 87527202 | 87527224 | 0.01 |
| GTCCTGCCCTCCAGCAATACC | 87527222 | 87527244 | 0.02 |
| GAAAACCTGGTATTGCTGGA | 87527227 | 87527249 | 0.14 |
| GCCAGCAAAGGCAGGAAAACC | 87527240 | 87527262 | 1.00 |
| GGTGTGTTCCCAGCAAAGGC | 87527249 | 87527271 | 0.02 |
| GGAAATGGTCTAGAGGGAAA | 87527270 | 87527292 | 0.20 |
| GTCTTCTGGAAATGGTCTAGA | 87527276 | 87527298 | 0.48 |
| GCCAGTAGCTTCTTCTGGAAA | 87527285 | 87527307 | 0.01 |
| GAAGAAGCTACTGGTCTGCAT | 87527295 | 87527317 | 0.01 |
| GCAACTCAGCACCATCTTTC | 87527331 | 87527353 | 0.01 |
| GCACCATCTTTCCGGGACCC | 87527339 | 87527361 | 0.02 |
| GTGTTGGAGAACCAGGGTCC | 87527350 | 87527372 | 0.00 |
| GCTCTGTGTGTTGGAGAACCA | 87527356 | 87527378 | 0.56 |
| GTGTGTCTGTCTCTGTGTGT | 87527366 | 87527388 | 0.02 |
| GAGACAGACACACTCATGA | 87527376 | 87527397 | 0.36 |
| GACATGCACACCAATCAGCCC | 87527399 | 87527421 | 0.51 |
| GTACTGTACCCCAGGGCTGAT | 87527408 | 87527430 | 0.02 |
| GATGGGCCATACTGTACCCC | 87527417 | 87527439 | 0.03 |
| GTTTAGGTGGCTTATGGGAT | 87527434 | 87527456 | 0.03 |
| GCTTTGTTTAGGTGGCTTAT | 87527439 | 87527461 | 0.53 |
| GTCATGATTCCTGCTTTGTTT | 87527450 | 87527472 | 0.03 |
| GATCATGAGATATTTATCAAA | 87527466 | 87527488 | 0.82 |
| GATATTTATCAAACGGGGC | 87527473 | 87527494 | 0.04 |
| GATCAAACGGGGCTGGAGAAT | 87527480 | 87527502 | 0.04 |
| GCACTGGCTGAGCCGGGCGG | 87527517 | 87527539 | 0.00 |
| GCTATATGTGTGTCTGTGTGA | 87527799 | 87527821 | 0.02 |
| GATATCAGATGAATGAGTGAG | 87527866 | 87527888 | 0.01 |
| GATGAATGAGTGAGTGGAG | 87527872 | 87527893 | 0.76 |
| GTGAGTGGAGTGGTGGTGAA | 87527881 | 87527903 | 0.23 |
| GTAGTGTCATACTCTAAGGT | 87527912 | 87527934 | 0.30 |
| GTATGACACTACTTCTAGTCC | 87527924 | 87527946 | 0.03 |
| GCCTAGAAGCACTGGGTTTCC | 87527942 | 87527964 | 0.01 |
| GGTGACGTCCTAGAAGCACT | 87527950 | 87527972 | 0.02 |
| GCACCTGAGACTCTGAGTACC | 87527969 | 87527991 | 0.07 |
| GTCTCCTCATGAACTCCTCC | 87527987 | 87528009 | 0.05 |

| | | | |
|---|---|---|---|
| GACTTGAGGAAAGGTGCATA | 87528009 | 87528031 | 0.01 |
| GGTGAGGGGACTTGAGGAA | 87528018 | 87528039 | 0.08 |
| GAGGGAGGTGAGGGGACTTG | 87528023 | 87528045 | 0.04 |
| GAGGAAAGGAGGGAGGTGAG | 87528031 | 87528053 | 0.02 |
| GTAAGGGAAGGGAGGAAAGGA | 87528041 | 87528063 | 0.07 |
| GCCCTCGATTAAGGGAAGGG | 87528050 | 87528072 | 0.01 |
| GTTGCAAAGTGCCCTCGATTA | 87528059 | 87528081 | 0.00 |
| GCAGTAGAAAGTTTAGCATGT | 87528084 | 87528106 | 0.08 |
| GATGTAGCCGATCCTCCGCAA | 87528110 | 87528132 | 0.01 |
| GCTCACCATTGCGGAGGAT | 87528116 | 87528137 | 0.02 |
| GCACACAGCTCACCATTGCGG | 87528121 | 87528143 | 0.01 |
| GTGAGCTGTGTGGTGAACAG | 87528132 | 87528154 | 0.00 |
| GAAACTGTAAGCATTCGGAAG | 87528183 | 87528205 | 0.01 |
| GAAAATTAAACTGTAAGCATT | 87528189 | 87528211 | 0.01 |
| GATTCCATTTATTTATTTACA | 87528213 | 87528235 | 0.01 |
| GTTATTTATTTACAAGGGTAG | 87528220 | 87528242 | 0.12 |
| GGATGCGTGCATGTAGATA | 87528244 | 87528265 | 0.02 |
| GTATGGAGCCTGTGTGTGCCA | 87528261 | 87528283 | 0.19 |
| GCCTGTGTGTGCCACGGCAC | 87528267 | 87528289 | 0.01 |
| GTGTGCCACGGCACAGGTGC | 87528273 | 87528295 | 0.01 |
| GGCACAGGTGCAGGTGTTAG | 87528282 | 87528304 | 0.01 |
| GTTCTTTCCTTCTAGCACGT | 87528311 | 87528333 | 0.01 |
| GGGCCTCCCCACGTGCTAGA | 87528318 | 87528340 | 0.03 |
| GGAGGCCCGGGGTCAAACTC | 87528333 | 87528355 | 0.03 |
| GGGGTCAAACTCAGGCGGTC | 87528341 | 87528363 | 0.05 |
| GACTCAGGCGGTCAGGCTCAG | 87528349 | 87528371 | 0.00 |
| GCAGTGGTAAGTGCTTTTCCT | 87528366 | 87528388 | 0.00 |
| GCCAGTAGGGTGGCTCACCT | 87528383 | 87528405 | 0.01 |
| GTGAACATGGGCCAGTAGGG | 87528393 | 87528415 | 0.01 |
| GGCCCATGTTCACAATTAGT | 87528403 | 87528425 | 0.13 |
| GTAGGTGAGAACAGCCAGGC | 87528421 | 87528443 | 2.00 |
| GAACAGCCAGGCAGGTACATA | 87528430 | 87528452 | 0.01 |
| GTTGACCCTTATGTACCTGCC | 87528435 | 87528457 | 0.07 |
| GCACAACAGTAAAGCCCACTC | 87528462 | 87528484 | 0.01 |
| GCCCACTCAGGAACTCTTTG | 87528474 | 87528496 | 0.01 |
| GTGGTCAAGAGAATAGAAT | 87528493 | 87528514 | 0.03 |
| GATAGAATAGGTAGCCAGGTG | 87528505 | 87528527 | 0.01 |
| GAATAGGTAGCCAGGTGTGG | 87528508 | 87528530 | 1.06 |
| GTGCAGATCAGGCAGGCCT | 87528584 | 87528605 | 0.01 |
| GCTGGGTGTCCCTGGCTGTCC | 87528614 | 87528636 | 0.01 |
| GAAACCCTGTCTCAGAAAGAC | 87528639 | 87528661 | 0.01 |
| GTCAGAAAGACAGGATGAGGA | 87528649 | 87528671 | 0.01 |
| GAAAGACAGGATGAGGATGG | 87528652 | 87528674 | 0.38 |
| GATGAGGATGGGGGTGGGAGG | 87528662 | 87528684 | 0.01 |
| GGGGGTGGGAGGAGGAGGAT | 87528670 | 87528692 | 0.04 |
| GAGGAGGATAGGATGGTGAC | 87528681 | 87528703 | 0.01 |
| GCTGGAATGTTAGGTCTTCAC | 87528700 | 87528722 | 0.01 |
| GACTGGAACATCTGGAATGTT | 87528710 | 87528732 | 0.01 |
| GACAGAGCAACTGGAACATC | 87528719 | 87528741 | 0.02 |
| GCAGGGGACTGACAGAGCAAC | 87528728 | 87528750 | 0.01 |
| GGGCGTGGTCTTTTGGCCAG | 87528745 | 87528767 | 0.01 |
| GTCTTCCTGGGCGTGGTCTTT | 87528752 | 87528774 | 0.01 |

| | | | |
|---|---|---|---|
| GTAAAACTGTCTTCCTGGGCG | 87528760 | 87528782 | 0.01 |
| GTAAGGTAAAACTGTCTTCCT | 87528765 | 87528787 | 0.36 |
| GTTAAACCATGCTAAAAACAC | 87528785 | 87528807 | 0.02 |
| GTTTTGCCTGTGTTTTTAGCA | 87528790 | 87528812 | 0.00 |
| GAAGTAACGGTGTCTCATTGA | 87528822 | 87528844 | 0.29 |
| GATTGTGGGAAGGAAAAGTAA | 87528836 | 87528858 | 0.04 |
| GCACTAGGAGAGGGAATTGT | 87528851 | 87528873 | 0.01 |
| GTATGGTTCTGCACTAGGAGA | 87528860 | 87528882 | 0.02 |
| GAGCTTTATGGTTCTGCACT | 87528866 | 87528888 | 0.56 |
| GCAGAACCATAAAGCTCCGC | 87528872 | 87528894 | 0.39 |
| GCGGACCCAGCGGAGCTTTA | 87528878 | 87528900 | 0.00 |
| GAAGGGTAGGCGGACCCAG | 87528888 | 87528909 | 0.02 |
| GTTAGGCCTCAGAAGGGTAGG | 87528897 | 87528919 | 0.01 |
| GGCTTAGGCCTCAGAAGGGT | 87528900 | 87528922 | 0.02 |
| GAAAGTTGCACTTTCTGGCTT | 87528915 | 87528937 | 0.01 |
| GAAAGGAAAAGTTGCACTTTC | 87528921 | 87528943 | 0.01 |
| GGTTTGTGGGGAGGAAAAAA | 87528939 | 87528961 | 0.63 |
| GCATCAAAAGAGGGGTTTGT | 87528952 | 87528974 | 0.01 |
| GTTCTGGCTAGCATCAAAAG | 87528962 | 87528984 | 1.33 |
| GTTTGATGCTAGCCAGAACCT | 87528967 | 87528989 | 0.01 |
| GCAAAAAGCTCTCCTAGGTTC | 87528978 | 87529000 | 0.03 |
| GTTGTCACAAAAAGCTCTCCT | 87528984 | 87529006 | 0.36 |
| GAGCTTTTTGTGACAACTGT | 87528991 | 87529013 | 0.01 |
| GCAGGGCATTACAAAAAGAGC | 87529022 | 87529044 | 0.01 |
| GTAGGTAAGGGGGGGGAGAC | 87529041 | 87529063 | 0.05 |
| GTGGGGGGGTAGGTAAGGGG | 87529049 | 87529071 | 0.05 |
| GGACGGGGGCGGGGGTGGGG | 87529063 | 87529085 | 0.01 |
| GTCGGGGACGGGGGCGGGGG | 87529068 | 87529090 | 0.01 |
| GGAGGGGTCGGGGACGGGGG | 87529074 | 87529096 | 0.03 |
| GAGCTGGTGTGGAGGGGTCG | 87529084 | 87529106 | 0.57 |
| GCCACCGGAGCTGGTGTGGAG | 87529090 | 87529112 | 0.23 |
| GAGTTCCACCGGAGCTGGTG | 87529095 | 87529117 | 0.00 |
| GGAGAGAGTTCCACCGGAGC | 87529100 | 87529122 | 1.41 |
| GAGGTGGAGAGAGTTCCAC | 87529106 | 87529127 | 0.01 |
| GCATAGAGGAAGCGGTGAGG | 87529121 | 87529143 | 0.53 |
| GCAGCAGCTGCATAGAGGAAG | 87529129 | 87529151 | 1.68 |
| GAAACGCCAGCAGCTGCATAG | 87529135 | 87529157 | 0.06 |
| GTTTACAGCTTGCCTGCCTT | 87529154 | 87529176 | 0.02 |
| GAAAGAAAAACATCCCAAGGC | 87529166 | 87529188 | 0.03 |
| GGATTCAAGTAGAAGGTTG | 87529209 | 87529230 | 0.01 |
| GAAAGAATGGATTCAAGTAGA | 87529215 | 87529237 | 0.03 |
| GTAACAAAAGGATTAAAGAA | 87529229 | 87529251 | 0.02 |
| GTTCTTGGTCTCAGTAACAAA | 87529241 | 87529263 | 1.61 |
| GAGACCAAGAACCCTAAGAG | 87529253 | 87529275 | 0.02 |
| GAAATCAGTGTCCCCTCTTA | 87529264 | 87529286 | 0.01 |
| GCTGATTTCCTCAGAGTCGTG | 87529279 | 87529301 | 0.07 |
| GTTTGTATCCTCACGACTCTG | 87529286 | 87529308 | 0.11 |
| GTCGTGAGGATACAAATGCA | 87529293 | 87529315 | 0.92 |
| GAAGGACACCTGTACAAGAAC | 87529312 | 87529334 | 0.01 |
| GCCTCGGACCTGTTCTTGTAC | 87529319 | 87529341 | 0.01 |
| GTCCGAGGGAGCGTGCGCTTC | 87529335 | 87529357 | 0.00 |
| GTGCGCTTCCGGTCAGAGCT | 87529346 | 87529368 | 0.02 |

| | | | |
|---|---|---|---|
| GAGTCTTCCCAGCTCTGAC | 87529354 | 87529375 | 0.53 |
| GGGAAGACTCAAAGTCAAAG | 87529366 | 87529388 | 0.47 |
| GTCAAAGTCAAAGAGGCTTCC | 87529374 | 87529396 | 0.57 |
| GGCTTCCTGGAGGAGCCCAA | 87529387 | 87529409 | 1.92 |
| GAGTCCCATTGGGCTCCTCC | 87529392 | 87529414 | 1.93 |
| GCAGCAGCTTCGAGTCCCATT | 87529402 | 87529424 | 0.01 |
| GCTCGAAGCTGCTGTCTGGCC | 87529412 | 87529434 | 0.01 |
| GCTGCTGTCTGGCCTGGCCGT | 87529419 | 87529441 | 0.07 |
| GCTATCAGGCTTCCGACGGCC | 87529430 | 87529452 | 0.01 |
| GCTTCCCTATCAGGCTTCCGA | 87529435 | 87529457 | 0.01 |
| GGAAGCCTGATAGGGAAGAG | 87529440 | 87529462 | 0.04 |
| GCCTGTCCCCTCTTCCCTATC | 87529445 | 87529467 | 0.01 |
| GAAGAGGGGACAGGAAGCTC | 87529454 | 87529476 | 0.07 |
| GTTGTTTGATCTGTTCTTCCC | 87529473 | 87529495 | 0.79 |
| GTAGATTTAATGTACACCAC | 87529507 | 87529529 | 0.01 |
| GATACTCGGCACATTGCCTG | 87529523 | 87529545 | 0.01 |
| GTGCCGAGTATCTTAAGATA | 87529534 | 87529556 | 0.02 |
| GAGTATCTTAAGATAGGGTG | 87529539 | 87529561 | 0.00 |
| GTCTTAAGATAGGGTGAGGGA | 87529544 | 87529566 | 0.02 |
| GGTGAGGGAGGGACAGTTCT | 87529555 | 87529577 | 0.01 |
| GTAATCTTTGTTTAGAGATTC | 87529578 | 87529600 | 0.01 |
| GATCTCTAAACAAAGATTAAG | 87529583 | 87529605 | 0.04 |
| GAAAATAGTTTATTAAAAGTG | 87529609 | 87529631 | 0.04 |
| GTTTATTAAAAGTGTGGCACA | 87529616 | 87529638 | 0.02 |
| GTGTGGCACATGGGAGGTAG | 87529626 | 87529648 | 0.02 |
| GACAAAGAGCAGTGCAATTTT | 87529663 | 87529685 | 0.04 |
| GAGCAGTGCAATTTTAGGAC | 87529668 | 87529690 | 0.01 |
| GCAATTTTAGGACAGGGGAA | 87529675 | 87529697 | 0.10 |
| GAGTCTAGTTCTTGTGTCTT | 87529698 | 87529720 | 0.30 |
| GACAAGAACTAGACTCTTTAG | 87529706 | 87529728 | 0.01 |
| GTTAGAGGCAGAGACTGTGAA | 87529722 | 87529744 | 0.01 |
| GGAAAATCAGATTAAAAAC | 87529743 | 87529764 | 0.03 |
| GCAGATTAAAAACAGGAGACA | 87529750 | 87529772 | 0.01 |
| GTACGCAGCAAGCGTAGATTG | 87529776 | 87529798 | 0.01 |
| GCTTGCTGCGTAGATTCCTC | 87529787 | 87529809 | 0.87 |
| GATTCCTCTGGTGCCACCTC | 87529799 | 87529821 | 0.00 |
| GTGCCACCTCCGGGTGGACG | 87529809 | 87529831 | 0.01 |
| GACTTGAGACCCTCGTCCACC | 87529818 | 87529840 | 0.01 |
| GCTTCCATGATACCACTTTCC | 87529843 | 87529865 | 0.01 |
| GTCTCTGCTTTACCAGGAAAG | 87529854 | 87529876 | 1.80 |
| GTCTCTGTTCTCTGCTTTACC | 87529861 | 87529883 | 0.14 |
| GAAGCTGGATGGAAATGTACA | 87529887 | 87529909 | 0.01 |
| GTACATTTCCATCCAGCTTTT | 87529892 | 87529914 | 0.01 |
| GCTATTTGCCTAAAAGCTGGA | 87529899 | 87529921 | 0.01 |
| GCTTTTAGGCAAATAGGGGA | 87529906 | 87529928 | 0.01 |
| GTAAACGATCAGATGCAAGG | 87529934 | 87529956 | 0.02 |
| GAAATAATCCTTACGCTTAAA | 87529960 | 87529982 | 0.05 |
| GCACACGCCATTTAAGCGTA | 87529967 | 87529989 | 0.01 |
| GTTAAATGGCGTGTGCCCCGG | 87529975 | 87529997 | 1.47 |
| GGCGTGTGCCCCGGTGGTGG | 87529981 | 87530003 | 0.04 |
| GTGCTCTGCCACCCTTCAAT | 87530014 | 87530036 | 0.01 |
| GTTCCCATCCTATTGAAGGG | 87530022 | 87530044 | 0.01 |

| | | | |
|---|---|---|---|
| GATAGGATGGGAACAGCCTCA | 87530032 | 87530054 | 0.01 |
| GAACAGCCTCAAGGCTATGTA | 87530042 | 87530064 | 0.01 |
| GAAGCCCCTTACATAGCCTTG | 87530047 | 87530069 | 0.00 |
| GCTTAGGCCAAGCACCTAGTG | 87530067 | 87530089 | 0.01 |
| GAAAGGGCCTCACTAGGTGCT | 87530073 | 87530095 | 0.01 |
| GACTGCTCAAAGGGCCTCACT | 87530080 | 87530102 | 0.01 |
| GCAGGCTTGTTCACTGCTCAA | 87530091 | 87530113 | 0.02 |
| GCAGTGAACAAGCCTGTGGTA | 87530099 | 87530121 | 0.01 |
| GAACAAGCCTGTGGTAAGGCC | 87530104 | 87530126 | 0.01 |
| GCCAAGGCCAGGCCTTACCAC | 87530110 | 87530132 | 0.01 |
| GTAAGGCCTGGCCTTGGGGC | 87530116 | 87530138 | 0.01 |
| GGCCTTGGGGCAGGGGTGAG | 87530125 | 87530147 | 0.02 |
| GGGCAGGGGTGAGGGGGATG | 87530132 | 87530154 | 0.02 |
| GTGAGGGGGATGTGGGGGAG | 87530140 | 87530162 | 0.01 |
| GGGATGTGGGGGAGTGGAGA | 87530146 | 87530168 | 0.01 |
| GAGTGGAGATGGAGGGAGAC | 87530157 | 87530179 | 0.01 |
| GAGGGAGACAGGAGGATGAG | 87530168 | 87530190 | 0.07 |
| GAATGAAAGGACTTTTTGCTT | 87530198 | 87530220 | 0.01 |
| GTCCTTTCATTTAACTCAAAT | 87530211 | 87530233 | 0.07 |
| GTTAACTCAAATAGGACTGAC | 87530220 | 87530242 | 0.02 |
| GACTGGTTCCTATCGCTCGCC | 87530238 | 87530260 | 0.00 |
| GTTCAGACCCTGGCGAGCGAT | 87530245 | 87530267 | 0.26 |
| GCTATTTACTTTTTCAGACCC | 87530256 | 87530278 | 0.01 |
| GGGCCCGAGTCCTACTCCAG | 87530278 | 87530300 | 0.01 |
| GGCTAGGGACCCCTGGAGT | 87530288 | 87530309 | 0.01 |
| GCACTAGGCTAGGGACCCC | 87530294 | 87530315 | 0.00 |
| GCTGGGACTAAGCACTAGGCT | 87530303 | 87530325 | 0.01 |
| GAAGTCCTGGGACTAAGCACT | 87530308 | 87530330 | 0.28 |
| GCTTAGTCCCAGGACTTTG | 87530314 | 87530335 | 0.01 |
| GACTAATGGCCACAAAGTCCT | 87530321 | 87530343 | 0.06 |
| GACTTTGTGGCCATTAGTGGT | 87530327 | 87530349 | 0.01 |
| GTGGCCATTAGTGGTAGGGC | 87530332 | 87530354 | 0.00 |
| GGTAGGGCAGGGGGCGAAGG | 87530344 | 87530366 | 0.01 |
| GGGGCGAAGGAGGCTTGCTC | 87530354 | 87530376 | 9.25 |
| GTCTCTCCTGCCACAAACTAG | 87530379 | 87530401 | 0.16 |
| GCACAAACTAGCGGATCCTCA | 87530389 | 87530411 | 0.24 |
| GGACGATTGTCAGGACCGTG | 87530404 | 87530426 | 0.81 |
| GGTCCTGACAATCGTCCCC | 87530410 | 87530431 | 0.01 |
| GTAGGTCAGGGTGAGAGCCA | 87530426 | 87530448 | 0.04 |
| GCTGAGAGAGAGGTAGGTCA | 87530438 | 87530460 | 0.01 |
| GGTGTGGCTGAGAGAGAGGT | 87530444 | 87530466 | 0.03 |
| GCTCTCTCAGCCACACCTAGA | 87530451 | 87530473 | 0.01 |
| GTCAGCCACACCTAGAAGGAA | 87530456 | 87530478 | 0.88 |
| GCTGGCCAACCCTTTCCTTCT | 87530465 | 87530487 | 0.01 |
| GAAAGGGTTGGCCAGAGAGA | 87530473 | 87530495 | 0.09 |
| GTTGGCCAGAGAGAAGGGACT | 87530480 | 87530502 | 0.03 |
| GAAGGGACTAGGGTGACCTTC | 87530492 | 87530514 | 0.01 |
| GACCTTCCGGCAAATACACCT | 87530506 | 87530528 | 0.01 |
| GCAGCCCAAGGTGTATTTGC | 87530511 | 87530533 | 0.36 |
| GAAATACACCTTGGGCTGCAG | 87530516 | 87530538 | 0.69 |
| GCAGCTCCCCACTGCAGCCCA | 87530523 | 87530545 | 0.04 |
| GCTGCAGTGGGGAGCTGGGC | 87530529 | 87530551 | 0.01 |

| | | | |
|---|---|---|---|
| GTGGGGAGCTGGGCAGGCGA | 87530535 | 87530557 | 0.01 |
| GCTGGGCAGGCGACGGCCGA | 87530542 | 87530564 | 0.62 |
| GCAGGCGACGGCCGAGGGCC | 87530547 | 87530569 | 0.00 |
| GGGGAGGGCTCCAGGCCCT | 87530558 | 87530579 | 0.01 |
| GCTGAATCCGGGGAGGGCTCC | 87530565 | 87530587 | 0.03 |
| GGAGCTGCTGAATCCGGGGA | 87530572 | 87530594 | 0.02 |
| GCAGGGGAGCTGCTGAATCC | 87530577 | 87530599 | 0.01 |
| GTGGCCGGCAGGGGGCGCAG | 87530593 | 87530615 | 2.02 |
| GCTGCGCCCCCTGCCGGCCAC | 87530596 | 87530618 | 0.01 |
| GTGCGTCCGGTGGCCGGCAG | 87530602 | 87530624 | 0.06 |
| GATGCGTGGAGTGCGTCCGG | 87530612 | 87530634 | 2.10 |
| GGCGATGCGTGGAGTGCGTC | 87530615 | 87530637 | 0.01 |
| GAGGGTGGGGCGGCGATGCG | 87530626 | 87530648 | 0.00 |
| GTGCTGAAGCCGGGAGGGTG | 87530639 | 87530661 | 1.63 |
| GAGGGGGTGCTGAAGCCGGG | 87530645 | 87530667 | 0.02 |
| GAGGTAGGTGGTGGGGGAGG | 87530661 | 87530683 | 0.02 |
| GGAGCCGAGGTAGGTGGTGG | 87530667 | 87530689 | 0.01 |
| GATCCCGGGAGCCGAGGTAGG | 87530673 | 87530695 | 0.48 |
| GCCAACCTATCCCGGGAGCCG | 87530680 | 87530702 | 0.01 |
| GCTCCCGGGATAGGTTGGGG | 87530685 | 87530707 | 0.01 |
| GGAGGGAGCCTTCCATTGTT | 87530707 | 87530729 | 0.43 |
| GCCTTCCATTGTTCGGGGAG | 87530714 | 87530736 | 0.01 |
| GTCGGGGAGTGGAGGCGTTTC | 87530726 | 87530748 | 0.01 |
| GTCCGGAAGTTCCCTACCTGA | 87530744 | 87530766 | 1.01 |
| GCCGGTTGTCCCCATCAGGTA | 87530754 | 87530776 | 0.21 |
| GGACAACCGGGGACGTCCCC | 87530767 | 87530789 | 1.04 |
| GTTCTGGCCTGGGGACGTCCC | 87530773 | 87530795 | 0.01 |
| GAAGGGAGAAATTTCTGGCCT | 87530784 | 87530806 | 0.01 |
| GATTCCAAAGGGAGAAATTTC | 87530790 | 87530812 | 0.02 |
| GTTCTCCCTTTGGAATTCCAG | 87530798 | 87530820 | 0.01 |
| GGAATTCCAGAGGGGTGGGG | 87530808 | 87530830 | 0.13 |
| GACGTCACCTCCCCACCCCTC | 87530814 | 87530836 | 0.01 |
| GTGACGTTCTTAACGTTTAT | 87530830 | 87530852 | 0.34 |
| GTAACGTTTATTGGTTATTAA | 87530840 | 87530862 | 0.01 |
| GTATTAATGGGGTGCATTTCC | 87530854 | 87530876 | 0.44 |
| GTGCATTTCCCGGGCGAGCT | 87530864 | 87530886 | 0.01 |
| GTTCTTTCCCCTAGCTCGCC | 87530873 | 87530895 | 0.01 |
| GAAAGAACTTGGAAAGTGTCA | 87530889 | 87530911 | 3.91 |
| GTCATTCATTTTTCTCAGTG | 87530918 | 87530940 | 0.01 |
| GTTAGTCCTCGTGTGTATGTC | 87530949 | 87530971 | 0.01 |
| GATGTCAGGACACGCCTGAAG | 87530964 | 87530986 | 0.01 |
| GGACACGCCTGAAGAGGACT | 87530970 | 87530992 | 0.03 |
| GTTGGCTCCGAGTCCTCTTC | 87530977 | 87530999 | 0.02 |
| GGAGCCAACTGTTGTGCCCT | 87530991 | 87531013 | 9.16 |
| GTTGTGCCCTCGGAGGGCTA | 87531001 | 87531023 | 0.01 |
| GCCCTCGGAGGGCTAAGGAA | 87531006 | 87531028 | 0.09 |
| GTCCGCCCTTCCTTCATCTCT | 87531037 | 87531059 | 0.02 |
| GCTCCACTACCTAGAGATGA | 87531046 | 87531068 | 0.00 |
| GCAGACTTCTTGGAGGATGG | 87531068 | 87531090 | 1.52 |
| GAATAAGGCAGACTTCTTGG | 87531075 | 87531097 | 0.21 |
| GAACAGAGGTAATGAAGAATA | 87531090 | 87531112 | 0.01 |
| GTTCTTCATTACCTCTGTTAC | 87531095 | 87531117 | 3.29 |

| | | | |
|---|---|---|---|
| GACTGACATTGCCTGTAACAG | 87531105 | 87531127 | 0.01 |
| GCAATGTCAGTGCCTCGCTT | 87531117 | 87531139 | 0.13 |
| GTGCCTCGCTTTGGTAGGTC | 87531126 | 87531148 | 0.61 |
| GCAGGATTCTCTGCTCATTTT | 87531145 | 87531167 | 0.01 |
| GTTAAGCCCATCATTGTGTCC | 87531198 | 87531220 | 0.04 |
| GCTGCCCCTGGACACAATGAT | 87531203 | 87531225 | 5.36 |
| GGATAGGGCTTTCTGCCCC | 87531216 | 87531237 | 0.01 |
| GCAGAAAGCCCTATCCGTGTG | 87531223 | 87531245 | 0.01 |
| GATATCAGCCTCACACGGAT | 87531231 | 87531253 | 0.01 |
| GTGAGGCTGATATCTAGGG | 87531240 | 87531261 | 0.58 |
| GCTGATATCTAGGGAGGAGA | 87531245 | 87531267 | 0.01 |
| GTCCCATTTCCTGTCCAGTCA | 87531286 | 87531308 | 0.01 |
| GAGTTTCTCCTTGACTGGAC | 87531294 | 87531316 | 1.93 |
| GACTGGGAGTTTCTCCTTGAC | 87531299 | 87531321 | 0.13 |
| GAAACTCCCAGTATCCCCCA | 87531310 | 87531332 | 0.01 |
| GACGGGGACCGTGGGGGATAC | 87531317 | 87531339 | 2.47 |
| GCCAAATTCACGGGGACCGT | 87531326 | 87531348 | 0.43 |
| GTATGTGGGGCCAAATTCAC | 87531335 | 87531357 | 0.01 |
| GACGAAGATCTTCGGGTATGT | 87531349 | 87531371 | 3.43 |
| GGGTACTCACGAAGATCTTC | 87531357 | 87531379 | 0.01 |
| GTACCCCGAAATCTGAGCCC | 87531374 | 87531396 | 0.01 |
| GTTCAGAGAGCAAACCGTCCA | 87531391 | 87531413 | 0.01 |
| GTTGCTCTCTGAAAGGTATCC | 87531402 | 87531424 | 0.01 |
| GGACGCCACTTAAGATCACG | 87531423 | 87531445 | 0.17 |
| GAAAGCCCTCGTGATCTTAAG | 87531428 | 87531450 | 0.01 |
| GTTGAAAGGGAGAGACTGGCG | 87531454 | 87531476 | 0.00 |
| GAAATAGGGAGCCATTGAAA | 87531468 | 87531490 | 0.00 |
| GCTTTTCTTGTAAGGGAAAT | 87531483 | 87531505 | 0.01 |
| GCAGCTCGGCTTTTCTTGTA | 87531491 | 87531513 | 0.00 |
| GCTTGTGAGTGGCCAGCAGCT | 87531505 | 87531527 | 0.02 |
| GCAGCAGGGAGGCCTTGTGAG | 87531517 | 87531539 | 0.09 |
| GAATGGGGGACGCAGCAGGG | 87531529 | 87531551 | 0.01 |
| GACTAGAGAGAAGTGAATGG | 87531543 | 87531565 | 0.01 |
| GTCGTAGTTAAGGGATAGCA | 87531578 | 87531600 | 0.19 |
| GCTGATTTCCGTCGTAGTTA | 87531588 | 87531610 | 0.01 |
| GACGGAAATCAGCCTGGGCTC | 87531599 | 87531621 | 0.01 |
| GCTGCCTAGGGCCAGAGCCC | 87531610 | 87531632 | 0.12 |
| GGCCCTAGGCAGCAGATCCA | 87531620 | 87531642 | 0.52 |
| GATCCAGGGAGCGAGCCAGAG | 87531635 | 87531657 | 0.00 |
| GTATCACAACACTCGCCACTC | 87531649 | 87531671 | 0.06 |
| GAAAGTCCACGTGAGTGCTCA | 87531671 | 87531693 | 0.15 |
| GGGAGGATAAAAGCCACTC | 87531692 | 87531713 | 0.02 |
| GTTTTTTATTTATTTCCAGAG | 87531705 | 87531727 | 0.03 |
| GGAAGCGTGAGTCAAAGAGA | 87531728 | 87531750 | 0.03 |
| GATGGGTGGGGAGAGAGAA | 87531746 | 87531767 | 0.19 |
| GAGAGAGAAAGGCACTCCAA | 87531756 | 87531778 | 0.01 |
| GGCACTCCAACGGGGAGAAC | 87531766 | 87531788 | 0.01 |
| GCCACTCCCTGTTCTCCCCGT | 87531772 | 87531794 | 0.01 |
| GGGGAGAACAGGGAGTGGCC | 87531777 | 87531799 | 0.01 |
| GACAGGGAGTGGCCTGGAGGC | 87531784 | 87531806 | 0.02 |
| GCTGGGGTGCAGCCGGCCTCC | 87531795 | 87531817 | 0.24 |
| GATCTGGACTGGGGTGCAGC | 87531803 | 87531825 | 0.01 |

| | | | |
|---|---|---|---|
| GCTTGTCTCCGATCTGGACTG | 87531812 | 87531834 | 0.71 |
| GTCCAGATCGGAGACAAGGA | 87531817 | 87531839 | 0.32 |
| GTCAGGCCTCGTGGAGCACTG | 87531892 | 87531914 | 0.27 |
| GTCCACGAGGCCTGAGAAGTC | 87531901 | 87531923 | 0.05 |
| GACTGTTGGCCCTGACTTCTC | 87531910 | 87531932 | 0.35 |
| GGGGTTAGAAGCAGACTGT | 87531925 | 87531946 | 0.51 |
| GTCTGCTTCTAACCCCTGAG | 87531931 | 87531953 | 0.01 |
| GTTCATACTATGCCCTCTCA | 87531944 | 87531966 | 0.01 |
| GAGGGCATAGTATGAACTTC | 87531950 | 87531972 | 0.03 |
| GACAGGGACGACTGGACTGG | 87531986 | 87532008 | 0.01 |
| GGGGGGTGGACAGGGACGAC | 87531994 | 87532016 | 0.01 |
| GTCCACCCCCCACCCCCCAA | 87532006 | 87532028 | 0.15 |
| GTTTCCAGCCTTTGGGGGGTG | 87532013 | 87532035 | 0.01 |
| GCTCTACTATTTCCAGCCTT | 87532022 | 87532044 | 0.01 |
| GGAAATAGTAGAGCACCTCT | 87532031 | 87532053 | 0.13 |
| GCACCTCTGGGTGATCCAAC | 87532043 | 87532065 | 0.01 |
| GGGTGATCCAACTGGGGTCC | 87532051 | 87532073 | 0.01 |
| GTAGGATGCCGGGACCCCAGT | 87532058 | 87532080 | 1.32 |
| GGAGGAGGCAGTAGGATGCC | 87532069 | 87532091 | 0.16 |
| GAAGGGTAGGAGGAGGCAGT | 87532077 | 87532099 | 0.01 |
| GCAACTGGAAGGGTAGGAGG | 87532084 | 87532106 | 0.02 |
| GTTTGAGCAACTGGAAGGGT | 87532090 | 87532112 | 0.01 |
| GCAGGGGTTTGAGCAACTGGA | 87532095 | 87532117 | 0.01 |
| GACCCTCTGCAGCTCAGGCA | 87532113 | 87532135 | 0.00 |
| GTTTCTGACCCTCTGCAGCTC | 87532118 | 87532140 | 0.01 |
| GCAATGGAGATGTGTGACAC | 87532145 | 87532167 | 0.53 |
| GACATCTCCATTGCCTTGACC | 87532155 | 87532177 | 0.00 |
| GTTTCCTCCTGGTCAAGGCAA | 87532161 | 87532183 | 5.27 |
| GCTTGGTTTTCCTCCTGGTCA | 87532167 | 87532189 | 0.02 |
| GTTGAAACTTGGTTTTCCTCC | 87532173 | 87532195 | 0.01 |
| GGAAAACCAAGTTTCAAAA | 87532179 | 87532200 | 0.00 |
| GAATAAGGCCATTTTGAAACT | 87532185 | 87532207 | 0.01 |
| GTTGAGTCAGATTGCAAAATA | 87532201 | 87532223 | 0.02 |
| GCAATCTGACTCAAAAGGGTG | 87532211 | 87532233 | 0.01 |
| GACTCAAAAGGGTGTGGAG | 87532217 | 87532238 | 0.03 |
| GATTATAGACAGCAGCCACCA | 87532242 | 87532264 | 3.57 |
| GCAGCAGCCACCAAGGGACTT | 87532250 | 87532272 | 0.03 |
| GATTAAGCCAAAGTCCCTTGG | 87532256 | 87532278 | 0.00 |
| GGACTTTGGCTTAATATAAC | 87532264 | 87532286 | 0.02 |
| GCTTAATATAACTGGTGACC | 87532272 | 87532294 | 0.02 |
| GATAACTGGTGACCTGGGAAA | 87532279 | 87532301 | 0.42 |
| GGTGACCTGGGAAACGGCAG | 87532285 | 87532307 | 0.01 |
| GTTCTGCCTCTGCCGTTTCCC | 87532290 | 87532312 | 0.70 |
| GAAAATAATTTCTGCCTTCT | 87532310 | 87532332 | 0.01 |
| GATTTCTGCCTTCTCGGATTG | 87532317 | 87532339 | 0.02 |
| GCCTTCTCGGATTGAGGGAC | 87532323 | 87532345 | 0.31 |
| GTAAAGACTTCCTTCGAGCCG | 87532372 | 87532394 | 0.08 |
| GTTCGAGCTTCCTCGGCTCGA | 87532381 | 87532403 | 0.01 |
| GCTGCTTCCTTCGAGCTTCCT | 87532389 | 87532411 | 0.01 |
| GCTCGAAGGAAGCAGGTCAG | 87532397 | 87532419 | 0.01 |
| GGAGGCGCAAGGAAGTGGGA | 87532424 | 87532446 | 0.02 |
| GAGAGGGAGGCGCAAGGAAG | 87532429 | 87532451 | 0.01 |

| | | | |
|---|---|---|---|
| GAAGCTGGAGAGGGAGGCGCA | 87532435 | 87532457 | 0.01 |
| GCATGAAGAAAAGCTGGAGA | 87532445 | 87532467 | 2.46 |
| GCAGAAAGCATGAAGAAAAGC | 87532451 | 87532473 | 0.15 |
| GTAGGATGGGAAGGGGGCGA | 87532476 | 87532498 | 0.02 |
| GCCAAGAGGGTAGGATGGGA | 87532485 | 87532507 | 0.01 |
| GAAAGCCAAGAGGGTAGGAT | 87532489 | 87532511 | 0.01 |
| GACTGGAAAGCCAAGAGGGT | 87532494 | 87532516 | 0.20 |
| GAAAAGACTGGAAAGCCAAG | 87532499 | 87532521 | 0.01 |
| GAAGCGGAGATAAGAAAAGAC | 87532511 | 87532533 | 0.01 |
| GGGGGTGGGGTGGAAGAAG | 87532528 | 87532549 | 0.93 |
| GGGGCGGCATGGGGGTGGGG | 87532537 | 87532559 | 0.02 |
| GCAGGGGCGGGGGCGGCATG | 87532546 | 87532568 | 0.01 |
| GAAGCGAGCAGGGGCGGGGG | 87532553 | 87532575 | 0.03 |
| GGAGGGAGGGAAGCGAGCAG | 87532562 | 87532584 | 0.05 |
| GCAGATCAAAGGGAGGAGGGA | 87532575 | 87532597 | 0.03 |
| GAAGAACAGATCAAAGGGAGG | 87532580 | 87532602 | 0.01 |
| GAAGCTGAAAGAACAGATCAA | 87532587 | 87532609 | 0.08 |
| GTTCGCTGTTAATAAGTGGTT | 87532630 | 87532652 | 0.01 |
| GATTTTTCGCTGTTAATAAG | 87532635 | 87532657 | 0.01 |
| GGTTACAGACAGTCGCTGAC | 87532687 | 87532709 | 0.59 |
| GCTGTAACCCATCAATTAGTG | 87532702 | 87532724 | 0.11 |
| GCTGGATCCCTCACTAATTGA | 87532709 | 87532731 | 3.05 |
| GTTCTGTTTGTAGCGTGTTTC | 87532728 | 87532750 | 0.04 |
| GTCGCATCAGAAGGTCTGCTT | 87532756 | 87532778 | 0.11 |
| GCAAATTGTATCGCATCAGA | 87532766 | 87532788 | 0.01 |
| GATTTGCCACTTGAGCCCTGT | 87532783 | 87532805 | 0.04 |
| GTGGACCAACAGGGCTCAAG | 87532788 | 87532810 | 0.28 |
| GTTACTGCCCGTGGACCAACA | 87532797 | 87532819 | 0.88 |
| GCAAATGTGCTTACTGCCCG | 87532807 | 87532829 | 0.01 |
| GCACATTTGCTGCGTTGTGT | 87532820 | 87532842 | 1.82 |
| GTTGTGTGGGAGCGTTCCTGA | 87532834 | 87532856 | 0.51 |
| GAAGTTCTGGATCTGTCCTTC | 87532849 | 87532871 | 0.29 |
| GCCAGTATAATGAAAAAGTTC | 87532863 | 87532885 | 0.01 |
| GTTATCGTCTTGATGAGTA | 87532895 | 87532916 | 0.00 |
| GACTCTCCATGCCCCACCCTC | 87532915 | 87532937 | 0.01 |
| GAGTACCAGAGGGTGGGGCA | 87532920 | 87532942 | 0.01 |
| GTCTGTGAGTACCAGAGGGTG | 87532925 | 87532947 | 0.01 |
| GAAGTCATCTGTGAGTACCAG | 87532931 | 87532953 | 0.01 |
| GTCTTTACATTTGCATATGAG | 87532954 | 87532976 | 0.02 |
| GATTATCTTCAAAGGTTTTTA | 87532980 | 87533002 | 0.02 |
| GTTTCTTTCTATTATCTTCAA | 87532989 | 87533011 | 0.01 |
| GATAGAAAGAAAAAACCCTCA | 87533001 | 87533023 | 0.06 |
| GAACCCTCATGGTGTTAGTTT | 87533013 | 87533035 | 0.01 |
| GCATGGTGTTAGTTTTGGTTA | 87533019 | 87533041 | 0.01 |
| GTTTTGAATACAGAAAGAA | 87533100 | 87533121 | 0.01 |
| GAATACAGAAAGAAAGGACC | 87533105 | 87533127 | 0.01 |
| GAAAGGACCAGGCAGTGGTG | 87533116 | 87533138 | 0.08 |
| GTATGTCCCCACCACTGCC | 87533123 | 87533144 | 0.77 |
| GTGGTGGGGACATACACAGT | 87533130 | 87533152 | 0.02 |
| GTCTAAAACATTTTGAGTCGA | 87533155 | 87533177 | 0.01 |
| GTGCCCTTGGCATTATCTGT | 87533188 | 87533210 | 2.82 |
| GCCAAGGGCACACACATTCT | 87533200 | 87533222 | 0.02 |

| | | | |
|---|---|---|---|
| GCACACACATTCTCGGATGA | 87533207 | 87533229 | 0.54 |
| GCAGCCATGCACACACTGAG | 87533232 | 87533254 | 0.23 |
| GCCTGCATTTGGGACATTGAC | 87533267 | 87533289 | 0.59 |
| GATGGGTGAGGGACCTGCATT | 87533279 | 87533301 | 0.13 |
| GTTGGTCAAGTCCATGGGTGA | 87533291 | 87533313 | 0.04 |
| GCCAATATTGGTCAAGTCCAT | 87533297 | 87533319 | 0.62 |
| GTTGACCAATATTGGATATTA | 87533306 | 87533328 | 0.51 |
| GATATTGGATATTAAGGTTTT | 87533313 | 87533335 | 0.02 |
| GTTAATATTGTCAGTGTTAAC | 87533344 | 87533366 | 0.05 |
| GTGCCCAGAGGTTCGTCTAC | 87533378 | 87533400 | 0.95 |
| GAACCTCTGGGCACGCCTGTG | 87533388 | 87533410 | 0.01 |
| GTGAGGATTTTTCTTCACTA | 87533405 | 87533427 | 0.01 |
| GTTCTTCACTAGGGTAGCCTC | 87533415 | 87533437 | 1.05 |
| GGGTAGCCTCTGGGTGCATC | 87533425 | 87533447 | 0.96 |
| GTTCCTCCCAGATGCACCCAG | 87533431 | 87533453 | 0.10 |
| GATCTTGATTCTGTTTTTCTC | 87533456 | 87533478 | 0.00 |
| GTCTGGCCTTACCTGAAGAGA | 87533474 | 87533496 | 0.01 |
| GCCAGACCATCTCTTCAGGTA | 87533479 | 87533501 | 0.17 |
| GGTCTGGATTCAATTAGTTG | 87533495 | 87533517 | 1.44 |
| GCTGTAGCTTATTTTTAAGAT | 87533526 | 87533548 | 0.02 |
| GATAAGCTACAGTAAACTGAG | 87533538 | 87533560 | 0.01 |
| GTACAGTAAACTGAGCGGTGG | 87533544 | 87533566 | 0.01 |
| GACTGTTTTATTTTTCAAGCA | 87533678 | 87533700 | 0.01 |
| GACTGCACTATTCACTGCCC | 87533702 | 87533724 | 0.42 |
| GTATTCACTGCCCTGGGTCTT | 87533710 | 87533732 | 0.01 |
| GGGTCTTAGGCTACACAAGA | 87533723 | 87533745 | 0.01 |
| GGCTACACAAGAAGGAAGGC | 87533731 | 87533753 | 0.02 |
| GAAGGAAGGCAGGGCGTGGA | 87533741 | 87533763 | 0.12 |
| GAAGGCAGGGCGTGGAAGGA | 87533745 | 87533767 | 0.34 |
| GGGGCATGAAATAAAGACA | 87533769 | 87533790 | 0.03 |
| GACACTCCAGAGACAAGCAGA | 87533800 | 87533822 | 0.01 |
| GAGATCCGTCTGCTTGTCTC | 87533805 | 87533827 | 0.21 |
| GATCTCTCTCTGTGAGATCC | 87533822 | 87533844 | 0.00 |
| GCTGTGAGATCCAGGCCAACC | 87533831 | 87533853 | 0.02 |
| GTAGGACAGGCAGGGATATGT | 87533870 | 87533892 | 0.84 |
| GAAAAAGGAAGCTAACTGTGC | 87533922 | 87533944 | 0.07 |
| GGAAGCTAACTGTGCTGGGC | 87533927 | 87533949 | 0.09 |
| GTGCTGGGCAGGTTGTGTG | 87533938 | 87533959 | 0.06 |
| GGTCACACTGCATGAGCAG | 87533967 | 87533988 | 0.64 |
| GGCAGGAGCTTGAGGTAAC | 87533987 | 87534008 | 0.01 |
| GCAGGAGAGGCAGGAGCTTG | 87533994 | 87534016 | 0.06 |
| GGGAAGGAAGCAGGAGAGGC | 87534003 | 87534025 | 0.03 |
| GCATGATGATGGGAAGGAAGC | 87534012 | 87534034 | 0.01 |
| GTGGTCCATGATGATGGGA | 87534019 | 87534040 | 0.01 |
| GAAGGGTGTGGTCCATGATGA | 87534024 | 87534046 | 0.02 |
| GGCTCACAGTTCAAGGGTG | 87534037 | 87534058 | 0.01 |
| GGAGCGAGTGAGTTTATTT | 87534057 | 87534078 | 4.55 |
| GCTAAGTTGCTTTTGTCAAGG | 87534078 | 87534100 | 0.01 |
| GATTGATCGATCGATTGAT | 87534114 | 87534135 | 0.02 |
| GTTGATTGATTACATATACAA | 87534136 | 87534158 | 0.01 |
| GTCGACAAGGGCTGGAGAGA | 87534292 | 87534314 | 0.04 |
| GTAAAACATGTCGACAAGGGC | 87534300 | 87534322 | 0.02 |

| | | | |
|---|---|---|---|
| GTCGACATGTTTTAATCGAG | 87534309 | 87534331 | 0.04 |
| GTAATCGAGAGGAAGAAATTG | 87534321 | 87534343 | 0.34 |
| GAGGAAGAAATTGAGGAGT | 87534328 | 87534349 | 0.51 |
| GCCTCAGCAGTTGAGAGCAT | 87534356 | 87534378 | 0.02 |
| GTCGAGCGGCCAGGTGGGTGC | 87534409 | 87534431 | 0.27 |
| GACAGTTTCGAGCGGCCAGGT | 87534415 | 87534437 | 0.01 |
| GACTGAAGTTACAGTTTCGAG | 87534424 | 87534446 | 0.58 |
| GTGAGGGTGTTGGATGTCG | 87534448 | 87534469 | 0.01 |
| GATATATTTGTGTGAGGGTGT | 87534457 | 87534479 | 0.01 |
| GCTGCACATATATTTGTGTGA | 87534463 | 87534485 | 0.02 |
| GTTTAATTACTTTAAGTGCAT | 87534494 | 87534516 | 0.00 |
| GCTTAAAGTAATTAAAAAAAG | 87534502 | 87534524 | 0.03 |
| GGCAAGAAGAGAGATGGAGG | 87534542 | 87534564 | 0.01 |
| GATAATGGCAAGAAGAGAGA | 87534548 | 87534570 | 0.01 |
| GTGTTATTTAGGAAGATAA | 87534563 | 87534584 | 0.01 |
| GATAGCAATACTGTGTTATTT | 87534573 | 87534595 | 0.10 |
| GCATAGCATGTATATTGCATT | 87534599 | 87534621 | 0.02 |
| GTATTCTAAATAACATAGAGA | 87534622 | 87534644 | 0.04 |
| GATGGTTTAGTATATATGGA | 87534640 | 87534662 | 0.00 |
| GCTCACATCTCTTATATAAAA | 87534688 | 87534710 | 0.02 |
| GATGTGAGCACCCCAGAGTTT | 87534704 | 87534726 | 0.02 |
| GTCTTAGACACCAAAACTCTG | 87534713 | 87534735 | 0.03 |
| GTTTTGGTGTCTAAGAATCC | 87534720 | 87534742 | 0.02 |
| GAATCCTGGAACTAATCCCCT | 87534735 | 87534757 | 0.03 |
| GCTAATCCCCTTGGTCACCAA | 87534745 | 87534767 | 0.01 |
| GACTTCCTTTGGTGACCAAG | 87534750 | 87534772 | 0.06 |
| GTTAAAATACAGACTTCCTT | 87534761 | 87534783 | 0.02 |
| GAATGTCTTCAGTTATACAAA | 87534783 | 87534805 | 0.02 |
| GAATCAGTGTTTGAGTGTGCC | 87534806 | 87534828 | 0.01 |
| GTCTCAAACACGTTGGTCTCC | 87534824 | 87534846 | 0.01 |
| GGCGGGATTCTCAAACACGT | 87534832 | 87534854 | 0.02 |
| GCCCTAGACTCTAGGGAAGG | 87534850 | 87534872 | 0.00 |
| GCTTAATGGCCCTAGACTCTA | 87534857 | 87534879 | 0.23 |
| GCTAGGGCCATTAAGATAGCC | 87534866 | 87534888 | 0.01 |
| GCTCTGCCCAGGCTATCTTAA | 87534872 | 87534894 | 0.03 |
| GCACGGGCTCAACTCTGCCC | 87534884 | 87534906 | 0.02 |
| GAGCCCGTGCAGGGGAAAAT | 87534897 | 87534919 | 0.05 |
| GTGTGTGCACATAAGTGACT | 87534925 | 87534947 | 3.43 |
| GCATTGAGGAATAGTGGACAT | 87534947 | 87534969 | 1.79 |
| GCTTTCACAGTCCTACATTG | 87534962 | 87534984 | 0.30 |
| GAAGCTATAGAGTTTCTATCC | 87534981 | 87535003 | 0.01 |
| GCTAGAGCACTAACTCAGCC | 87534999 | 87535021 | 0.02 |
| GTCTAGCTGGGGCTCAGAAGC | 87535016 | 87535038 | 0.01 |
| GGGGCTCAGAAGCAGGCATG | 87535023 | 87535045 | 1.34 |
| GAAGCAGGCATGGGGTCACC | 87535031 | 87535053 | 0.19 |
| GACCTGGGAGATAACACTTGC | 87535048 | 87535070 | 0.01 |
| GAACACTTGCTGGAGATATGT | 87535059 | 87535081 | 1.48 |
| GCTGGAGATATGTAGGGCCA | 87535066 | 87535088 | 1.41 |
| GTCTGAATAGCGTCAATCCC | 87535083 | 87535105 | 0.13 |
| GATTGACGCTATTCAGACTCA | 87535089 | 87535111 | 0.01 |
| GCAGACTCAAGGTCAGCCTTA | 87535101 | 87535123 | 0.00 |
| GTATCTAGAAACTGACCCTTA | 87535116 | 87535138 | 0.27 |

| | | | |
|---|---|---|---|
| GGGTCAGTTTCTAGATAGGT | 87535122 | 87535144 | 0.03 |
| GGTAGGGAGCTGCTGCAAC | 87535139 | 87535160 | 0.01 |
| GCTGCTGCAACAGGAAGAAT | 87535147 | 87535169 | 0.01 |
| GACAGGAAGAATGGGGAGGGT | 87535156 | 87535178 | 0.13 |
| GAGGGTTGGCATCACAGAAC | 87535170 | 87535192 | 0.33 |
| GAACTGGCATCAGCAGCTG | 87535186 | 87535207 | 0.15 |
| GCTGTGGTGTCAGCAGCTGT | 87535201 | 87535223 | 0.03 |
| GCTGTGGGCTCAGCAGCTGT | 87535216 | 87535238 | 0.67 |
| GTCAGACTCAGCTGTGGACTG | 87535252 | 87535274 | 0.00 |
| GCTCACCTCAGACTCAGCTG | 87535259 | 87535281 | 0.00 |
| GCTGAGTCTGAGGTGAGCTGG | 87535265 | 87535287 | 0.00 |
| GCCTGGTCAAGAGGACTTTAG | 87535298 | 87535320 | 0.00 |
| GATGAACTCTTCCTGGTCAAG | 87535308 | 87535330 | 0.01 |
| GACCAGGAAGAGTTCATATC | 87535314 | 87535336 | 0.01 |
| GACAGTGGAGTTTTCATTAGG | 87535353 | 87535375 | 0.84 |
| GATGAAAACTCCACTGTTCTC | 87535360 | 87535382 | 0.02 |
| GCTTCTAACCAGAGAACAG | 87535369 | 87535390 | 0.01 |
| GAAGCAAAAAATTACTGCATG | 87535387 | 87535409 | 0.02 |
| GACTGCATGTGGTCAGTTCAC | 87535399 | 87535421 | 0.05 |
| GCAGGGGTTGTCTGAGACCAC | 87535418 | 87535440 | 0.02 |
| GATTACAGTTGCAAAGTAGCA | 87535482 | 87535504 | 0.03 |
| GTAGCAAGGAAAAATTTTAT | 87535496 | 87535518 | 0.03 |
| GTTGAGAACCACTGTCATTC | 87535570 | 87535592 | 0.01 |
| GAACAAACCGGAATGACAG | 87535578 | 87535599 | 0.00 |
| GTTTGTTTGTTTTTATCTACT | 87535601 | 87535623 | 0.01 |
| GTTTATCTACTTGGCACAAAC | 87535611 | 87535633 | 0.01 |
| GGCACAAACTGGCATTACCT | 87535622 | 87535644 | 0.01 |
| GCAGTTGAGGTTCTTCTTCCA | 87535639 | 87535661 | 0.01 |
| GAGGCAGCTTTTTTCAGTTG | 87535653 | 87535675 | 0.03 |
| GATCTATAGGCAGTCTGGTGG | 87535672 | 87535694 | 1.70 |
| GCCTATAGATATGTCTGTAG | 87535685 | 87535707 | 9.79 |
| GCTGTCTAAAAACAAACAGAA | 87535826 | 87535848 | 0.03 |
| GTTAGACAGGGTTTCTTTGTG | 87535841 | 87535863 | 0.02 |
| GAAAGCAGGCCTCTGAGTTTG | 87535894 | 87535916 | 0.26 |
| GTAGTAGAGGCAGAGGAAAGC | 87535909 | 87535931 | 0.01 |
| GTAGCACTCTAGTAGAGGCAG | 87535917 | 87535939 | 0.02 |
| GTACTAGAGTGCTAGGATCAA | 87535927 | 87535949 | 0.02 |
| GTGCTAGGATCAAAGGTAT | 87535934 | 87535955 | 0.01 |
| GGCCACCATGCCCAAGTGTA | 87535954 | 87535976 | 0.48 |
| GAAATTCCCTACACTTGGGCA | 87535959 | 87535981 | 0.09 |
| GAAAAAAATTCCCTACACTT | 87535964 | 87535986 | 0.03 |
| GTAGGGAATTTTTTTCATTAG | 87535972 | 87535994 | 0.03 |
| GTTGTTGTCTAGCCCTCTAGA | 87535995 | 87536017 | 0.02 |
| GCCAGGGTGGTGCCGTCTAGA | 87536006 | 87536028 | 0.01 |
| GTAGACGGCACCACCCTGGGC | 87536011 | 87536033 | 0.14 |
| GAGCAGGCTGAGAAAGCTGT | 87536054 | 87536076 | 0.03 |
| GCTCCTGCCTCCAGCCCTGCC | 87536120 | 87536142 | 0.31 |
| GGAGCTCCAGGCAGGGCTGG | 87536126 | 87536148 | 3.41 |
| GCAGGTCAGGAGCTCCAGGCA | 87536133 | 87536155 | 0.56 |
| GGAGTCAGGTCAGGAGCTCC | 87536138 | 87536160 | 0.06 |
| GACCTGACTCCCCACATAAA | 87536150 | 87536172 | 0.00 |
| GGTTGATTTCCATTTATGTG | 87536159 | 87536181 | 0.07 |

| | | | |
|---|---|---|---|
| GCAAAACAACTAGGGAGGAAA | 87536180 | 87536202 | 0.02 |
| GACTGACCAAAACAACTAGGG | 87536186 | 87536208 | 0.01 |
| GTCACAGCAATAGAACCCTAT | 87536215 | 87536237 | 0.00 |
| GAACCCTATAGGCAGACAAT | 87536226 | 87536248 | 0.02 |
| GGCAGACAATGGGGTGACTC | 87536236 | 87536258 | 0.04 |
| GTCACTGTTTAACACAGAGTT | 87536261 | 87536283 | 0.11 |
| GCACAGATAGATAGAGACCTA | 87536297 | 87536319 | 0.00 |
| GACCTATGGCTGACAACGTT | 87536311 | 87536333 | 0.01 |
| GATGGCTGACAACGTTGGGCT | 87536316 | 87536338 | 0.01 |
| GTAAGTAAAGTCTTCTTGA | 87536346 | 87536367 | 0.99 |
| GAAGAAGACTTTACTTACAAG | 87536351 | 87536373 | 0.00 |
| GACTTACAAGAGGAAAGACAA | 87536362 | 87536384 | 0.01 |
| GCTCCAAACTCTACATTTCTC | 87536391 | 87536413 | 1.15 |
| GCTGGAAACTCTGCCTTTCAC | 87536410 | 87536432 | 0.01 |
| GAGGTCACACAGCCTGTGAA | 87536422 | 87536444 | 0.01 |
| GAACAGGTAGTAACTTACCTG | 87536441 | 87536463 | 0.02 |
| GGTAAGTTACTACCTGTTCC | 87536446 | 87536468 | 0.72 |
| GAGGAAATGGGCCCAGGAAC | 87536458 | 87536480 | 0.01 |
| GCATATGGAGGAAATGGGCCC | 87536464 | 87536486 | 0.02 |
| GTCATTTCATATGGAGGAAAT | 87536470 | 87536492 | 0.02 |
| GCAAAAGCCCTCATTTCATA | 87536480 | 87536502 | 0.02 |
| GAAATGAGGGCTTTTGCTTG | 87536486 | 87536508 | 0.00 |
| GGCTTTTGCTTGAGGCCTCT | 87536494 | 87536516 | 3.66 |
| GCACAAGCACGCTAGCCTAG | 87536509 | 87536531 | 0.12 |
| GGGCTGCTCTCTAGCTATGA | 87536531 | 87536553 | 0.00 |
| GTAGAGAGCAGCCCTTCAGTG | 87536541 | 87536563 | 0.01 |
| GGGCTGCTTGCCTCACTGAA | 87536551 | 87536573 | 0.01 |
| GCAGTGAGGCAAGCAGCCCTC | 87536556 | 87536578 | 0.01 |
| GGCAAGCAGCCCTCAGGCAA | 87536562 | 87536584 | 0.03 |
| GTCAGAGCTGCCCTTGCCTGA | 87536571 | 87536593 | 0.01 |
| GCAGCTCTGAGAGCTTCACTG | 87536585 | 87536607 | 0.49 |
| GTGGGACCTGTCAACTCCTC | 87536604 | 87536626 | 0.01 |
| GAAGTTCCCGGAGGAGTTGAC | 87536610 | 87536632 | 0.01 |
| GGAACACTCTCTGAAGTTCC | 87536623 | 87536645 | 0.23 |
| GTGTTCCTGTGAAAAATGCA | 87536639 | 87536661 | 0.02 |
| GCCTTCCCCTGCATTTTTCAC | 87536644 | 87536666 | 0.01 |
| GCAGGGGAAGGAACTTTGTC | 87536656 | 87536678 | 0.37 |
| GGATGCTAGTCTGCCCTTAA | 87536677 | 87536699 | 0.04 |
| GATTTATTTCTAGCCTTTAA | 87536690 | 87536712 | 0.01 |
| GAAATAAATCCAGATGCCAG | 87536703 | 87536725 | 0.01 |
| GTAATTCTGCCCCTGGCATC | 87536712 | 87536734 | 0.02 |
| GCTTCCTGGTAATTCTGCCCC | 87536719 | 87536741 | 0.05 |
| GGGTGACTTTTCTAGCTTCC | 87536734 | 87536756 | 0.02 |
| GTTGTGACTGTATGTATGGCT | 87536755 | 87536777 | 0.01 |
| GAAAGGTTGTGACTGTATGTA | 87536760 | 87536782 | 0.01 |
| GGATGTCTTCTATAAGGAAA | 87536778 | 87536800 | 0.02 |
| GATTTGGGGATGTCTTCTATA | 87536784 | 87536806 | 0.41 |
| GTGTGTAGTGTACACAATTT | 87536800 | 87536822 | 20.69 |
| GTCTAGGTTCTGTGAAGCATG | 87536822 | 87536844 | 0.24 |
| GCAGCTAGCACGGCTACATCT | 87536839 | 87536861 | 0.05 |
| GTAAGTTACAGGCAGCTAGCA | 87536850 | 87536872 | 0.01 |
| GCAACAATGGCCGTAAGTTAC | 87536862 | 87536884 | 0.01 |

| | | | |
|---|---|---|---|
| GCTTACGGCCATTGTTGTAGC | 87536869 | 87536891 | 0.01 |
| GTCTGCCTCCTGCTACAACAA | 87536876 | 87536898 | 0.01 |
| GCAGGAGGCAGACACAGAC | 87536887 | 87536908 | 0.27 |
| GACAGGTCTCCCCAGTGAGA | 87536903 | 87536925 | 0.01 |
| GAGTCATATCCCTCTCACTG | 87536912 | 87536934 | 1.72 |
| GATAGACAGCTACAGCTTTGA | 87536939 | 87536961 | 0.00 |
| GAGACTGGGTCAGGTGTGGA | 87537003 | 87537025 | 1.19 |
| GCAGGAAGGCGAGACTGGGTC | 87537012 | 87537034 | 0.01 |
| GGGATCAGGAAGGCGAGACT | 87537017 | 87537039 | 0.02 |
| GGACTCACTGGGATCAGGA | 87537027 | 87537048 | 7.75 |
| GGTTGTAGGGTGGACTCACT | 87537037 | 87537059 | 0.51 |
| GCTGTCTTGTAGGTTGTAGGG | 87537047 | 87537069 | 0.02 |
| GGTAAGACTACCTGTCTTGT | 87537058 | 87537080 | 0.77 |
| GAGAAGACTTCTCTCTCAGT | 87537083 | 87537105 | 3.85 |
| GACTGAGAGAGAAGTCTTCTC | 87537086 | 87537108 | 0.24 |
| GCTTCTCAGGTTTGCAGAAGC | 87537100 | 87537122 | 0.02 |
| GGAAGGAGGAAGGAGAGGG | 87537141 | 87537162 | 0.02 |
| GTGGAGGGAGGAAGGAAGGA | 87537169 | 87537191 | 0.07 |
| GAAGGAATAGTGGAGGGAGGA | 87537177 | 87537199 | 0.13 |
| GAAGTAGGAAAGGAATAGTGG | 87537185 | 87537207 | 4.73 |
| GGAAAGACAGAAAGTAGGAA | 87537196 | 87537218 | 0.17 |
| GTCTTGTTTCAAAAAATGGA | 87537217 | 87537239 | 0.01 |
| GAAGACACTGATATGTAGCCC | 87537235 | 87537257 | 0.16 |
| GCTGGAAGTCCAGGGCTAGCC | 87537253 | 87537275 | 0.01 |
| GCAGAAGGACTGGAAGTCCA | 87537262 | 87537284 | 0.03 |
| GGAGGCAGTGGCAGAAGGAC | 87537272 | 87537294 | 0.08 |
| GATTTGGGAGGCAGTGGCAGA | 87537277 | 87537299 | 0.02 |
| GTTAGAAACTCAGCATTTGGG | 87537290 | 87537312 | 0.01 |
| GTAAGCATGTGCTGCCAAGCC | 87537310 | 87537332 | 0.00 |
| GCTGCCAAGCCTGGCTTCCC | 87537319 | 87537341 | 1.18 |
| GAAGAAAGTCCTGGGAAGCC | 87537328 | 87537350 | 0.01 |
| GAAAAGGTAGAAGAAAGTCC | 87537337 | 87537359 | 0.02 |
| GAAACCAAAGAGCCTGGAAA | 87537353 | 87537375 | 0.00 |
| GGCTCTTTGGTTTCCTTACT | 87537362 | 87537384 | 0.01 |
| GACTGGACTTGAACCCAAGTA | 87537375 | 87537397 | 0.01 |
| GGGTTCAAGTCCAGTCATGC | 87537383 | 87537405 | 0.02 |
| GCCACTTTGCTCCAGCATGAC | 87537393 | 87537415 | 0.01 |
| GAATGAGATTCACATCTCTG | 87537415 | 87537437 | 1.06 |
| GTTCACATCTCTGTGGCTGAG | 87537423 | 87537445 | 0.01 |
| GCCCTGAGACCCCAGCACCC | 87537445 | 87537467 | 0.02 |
| GCTGCTTGGTCCCTGGGTGCT | 87537455 | 87537477 | 0.01 |
| GGGACCAAGCAGGCCCAATG | 87537466 | 87537488 | 3.77 |
| GATGTCCCCATTGGGCCTGCT | 87537470 | 87537492 | 0.00 |
| GCAGCACTTTATGTCCCCATT | 87537479 | 87537501 | 1.23 |
| GATAAAGTGCTGGGCAGCACC | 87537491 | 87537513 | 0.42 |
| GGGCAGCACCTGGAATCACA | 87537501 | 87537523 | 0.00 |
| GTTTTTCTGCCCTGTGATTCC | 87537509 | 87537531 | 0.31 |
| GAAAAAGTCCTAAAAACTGA | 87537526 | 87537548 | 0.04 |
| GGGGGGAACCCTCAGTTTTT | 87537534 | 87537556 | 0.02 |
| GAAGAAAAAGGGGGCCCGGG | 87537551 | 87537573 | 0.87 |
| GACTTGAGAACAGAAGAAAA | 87537563 | 87537585 | 0.01 |
| GAGTGCTGAATTTTACATTT | 87537602 | 87537624 | 0.77 |

| | | | |
|---|---|---|---|
| GATTCAGCACTCAACCACAGC | 87537614 | 87537636 | 0.66 |
| GCAGAGAAATCTTACCAGCTG | 87537627 | 87537649 | 0.01 |
| GATTTCTCTGCCATGTCTA | 87537640 | 87537661 | 0.38 |
| GTCTATGGTTCAAAAAGAGC | 87537654 | 87537676 | 0.01 |
| GGCTTGTGGGCTTGTCTGTA | 87537686 | 87537708 | 0.05 |
| GATGTGACACTTCCTGGCTTG | 87537700 | 87537722 | 0.01 |
| GGGAACATGTGACACTTCC | 87537707 | 87537728 | 0.46 |
| GAAAAGAGTCTGGTGTCACCA | 87537726 | 87537748 | 0.01 |
| GCCAACACACAAAAAAGAGTC | 87537737 | 87537759 | 0.01 |
| GAGTCTGGTGTTGCCTGGCG | 87537893 | 87537915 | 0.00 |
| GTTAATGAGTCTGGTGTTGCC | 87537898 | 87537920 | 0.02 |
| GACTGTGGCTTAATGAGTC | 87537908 | 87537929 | 0.04 |
| GCCTGTGACTGTGTGTGACTG | 87537922 | 87537944 | 0.13 |
| GACACACAGTCACAGGAAAGA | 87537930 | 87537952 | 0.15 |
| GATCTAGCTCAGTTTTGGGG | 87537964 | 87537986 | 0.01 |
| GAAAGATCTAGCTCAGTTTTG | 87537967 | 87537989 | 0.01 |
| GTGCCTACTGTTGCCTAGTG | 87537990 | 87538012 | 0.01 |
| GCTGGGTGGGGCTCCCTCACT | 87538003 | 87538025 | 0.01 |
| GAGCCCCACCCAGGAGAGCT | 87538013 | 87538035 | 0.03 |
| GTTTGGTCCCAAGCTCTCCT | 87538021 | 87538043 | 0.01 |
| GTTAGGTTTTGTCGTTAGGTT | 87538038 | 87538060 | 1.44 |
| GTTTCGTTAGGTTTTGTCGTT | 87538043 | 87538065 | 0.01 |
| GAAACCTAACGAAACAAGCTC | 87538053 | 87538075 | 0.05 |
| GTCTGGGAAAACAGCTGCAAG | 87538071 | 87538093 | 0.01 |
| GCAGCTGCAAGCGGAATCGAT | 87538081 | 87538103 | 0.07 |
| GAATCGATTGGATTCCTGCAG | 87538094 | 87538116 | 0.03 |
| GCAGTGGCTATGTCCTAATG | 87538110 | 87538132 | 3.19 |
| GTATGTCCTAATGAGGCCACC | 87538118 | 87538140 | 0.34 |
| GGATCCCTGGTGGCCTCATT | 87538123 | 87538145 | 0.01 |
| GCCTTGTCAAGCTGGGATCCC | 87538136 | 87538158 | 0.11 |
| GATCCCAGCTTGACAAGGACT | 87538142 | 87538164 | 0.01 |
| GCACAAAAGAAACTGTGCATG | 87538168 | 87538190 | 0.01 |
| GACTGTGCATGTGGCACCTCT | 87538178 | 87538200 | 0.00 |
| GCATGTGGCACCTCTAGGACC | 87538184 | 87538206 | 0.02 |
| GGTGTGGCACCAGGTCCTAG | 87538193 | 87538215 | 0.01 |
| GGACCTGGTGCCACACCTAG | 87538199 | 87538221 | 0.01 |
| GCTTGACAGTCCCCCTAGGTG | 87538209 | 87538231 | 0.00 |
| GGGACTGTCAAGAAACAGTG | 87538220 | 87538242 | 0.00 |
| GAAACAGTGTGGAGGGGTGA | 87538231 | 87538253 | 0.01 |
| GGAGGGGTGATGGCTTGACT | 87538241 | 87538263 | 0.02 |
| GCTTGACTGGGGGCTCATTG | 87538253 | 87538275 | 1.09 |
| GATGATGCTGTATATATCCT | 87538275 | 87538297 | 0.46 |
| GCTAGGTCCAGATCCTTCCTC | 87538293 | 87538315 | 0.02 |
| GCTGACCCCGGAGGAAGGATC | 87538299 | 87538321 | 0.03 |
| GTGAATCTGACCCCGGAGGA | 87538305 | 87538327 | 0.01 |
| GTCCGGGGTCAGATTCACACC | 87538311 | 87538333 | 0.01 |
| GATTCACACCTGGGTAGCT | 87538321 | 87538342 | 0.01 |
| GTCAGATTCCTAGCTACCC | 87538329 | 87538350 | 0.00 |
| GAGAAGACCCACTCCATCC | 87538356 | 87538377 | 0.01 |
| GAGAGTAGCCAGGATGGAGT | 87538363 | 87538385 | 0.38 |
| GCTACTGGAGAGTAGCCAGGA | 87538369 | 87538391 | 0.27 |
| GGCTACTCTCCAGTAGCTC | 87538376 | 87538397 | 0.05 |

| | | | |
|---|---|---|---|
| GCTCAATTTCCTGAGCTAC | 87538385 | 87538406 | 0.01 |
| GTTTGTGGATCAGGAATGGA | 87538408 | 87538430 | 0.01 |
| GATGATCGCAGTTTGTGGATC | 87538417 | 87538439 | 0.01 |
| GAGGCCATGATCGCAGTTTG | 87538423 | 87538445 | 0.01 |
| GTCAGGGCATAAAGGAGGGGG | 87538442 | 87538464 | 0.01 |
| GCAATCCTCAGGGCATAAAGG | 87538448 | 87538470 | 0.11 |
| GCAGATTCAGACCCAATCCTC | 87538460 | 87538482 | 0.00 |
| GTCTCAGACTATGTATTCT | 87538482 | 87538503 | 0.29 |
| GCTATGTATTCTCGGTGTATC | 87538490 | 87538512 | 0.02 |
| GTAATTGCTGCTCGTTCCTC | 87538513 | 87538535 | 0.01 |
| GCCTCTTTGCAGAAGGTCCAG | 87538529 | 87538551 | 1.25 |
| GTTGGTGCACCTCTTTGCAGA | 87538537 | 87538559 | 0.01 |
| GTAACATTGCAAAGGAATCT | 87538556 | 87538578 | 0.02 |
| GGCTGGTTGTAACATTGCAA | 87538564 | 87538586 | 0.01 |
| GCAATGTTACAACCAGCCTT | 87538569 | 87538591 | 1.30 |
| GCCAACACAAACCCCAAAGGC | 87538581 | 87538603 | 0.00 |
| GGGGTTTGTGTTGGGGTTTC | 87538590 | 87538612 | 0.02 |
| GTCAGCTGCAATGAAATTATG | 87538622 | 87538644 | 0.01 |
| GATTTCATTGCAGCTGAACTG | 87538629 | 87538651 | 0.01 |
| GAGTGTCCCCATGTCCCCAT | 87538663 | 87538685 | 0.16 |
| GTTTGAGCCTATGGGGACATG | 87538669 | 87538691 | 0.02 |
| GCCAAATCATTTGAGCCTATG | 87538677 | 87538699 | 0.04 |
| GTCAAATGATTTGGATATGGA | 87538687 | 87538709 | 0.01 |
| GAATGATTTGGATATGGAAGG | 87538690 | 87538712 | 0.04 |
| GATGGGACAACTTTCAGGAGT | 87538716 | 87538738 | 0.01 |
| GAGATCATGGGACAACTTTC | 87538722 | 87538744 | 0.01 |
| GGCATGCATGTGGAGATCAT | 87538734 | 87538756 | 0.02 |
| GTGTAATGTGGCATGCATG | 87538744 | 87538765 | 0.10 |
| GCTCTCTCTTTCTGTGTAATG | 87538755 | 87538777 | 0.16 |
| GCTGTGTGTGTGTGTGTAATG | 87538834 | 87538856 | 0.03 |
| GAGACAGGACAGACAGAAAG | 87538920 | 87538942 | 1.65 |
| GAGGTGGAGACAGAGAGAGT | 87538939 | 87538961 | 0.06 |
| GGCCTTAAGTGAAGACAGAT | 87538960 | 87538982 | 4.58 |
| GTAAGTGAAGACAGATTGGCA | 87538965 | 87538987 | 0.01 |
| GGCATGGTTGTCCCCAGGAC | 87538981 | 87539003 | 0.01 |
| GTATCTGCTGGCCCTGTCCTG | 87538992 | 87539014 | 0.01 |
| GCCTTGGAGGCCCATATCTGC | 87539005 | 87539027 | 0.06 |
| GACAGCCATTGACACCCTTGG | 87539019 | 87539041 | 0.00 |
| GACGACAGCCATTGACACCCT | 87539022 | 87539044 | 0.01 |
| GTATTTTATATTCCTCCTTCA | 87539077 | 87539099 | 0.03 |
| GGAAGGACGTTCCTTGAAGG | 87539088 | 87539110 | 0.06 |
| GATAGAGTCATTAACATAAAA | 87539109 | 87539131 | 0.08 |
| GATGACTCTATGATAATCAGA | 87539122 | 87539144 | 0.00 |
| GGCATGAGTCCAGTAGCTGA | 87539143 | 87539165 | 0.01 |
| GAGTCCAGTAGCTGAAGGTG | 87539148 | 87539170 | 0.00 |
| GTTGAACAGCCACTAAGACTG | 87539198 | 87539220 | 0.02 |
| GTTCTTTTCCACAGTCTTAG | 87539206 | 87539228 | 0.06 |
| GTCACAAATCTAAAGAAACAG | 87539309 | 87539331 | 0.01 |
| GTCAAGGTCAGCACGGGACAC | 87539389 | 87539411 | 0.01 |
| GACAGTACAGTGTCCAAACCC | 87539414 | 87539436 | 0.01 |
| GACAGTGTCCAAACCCAGGTG | 87539419 | 87539441 | 0.06 |
| GCTTTCTACCACACCTGGGTT | 87539426 | 87539448 | 0.02 |

| | | | |
|---|---|---|---|
| GTTACCCTTTCTACCACACCT | 87539431 | 87539453 | 0.13 |
| GAAAGGGTAATTTCAGGACA | 87539444 | 87539466 | 0.01 |
| GATAGGATAAGCTAGCTGGCT | 87539468 | 87539490 | 0.01 |
| GATCACATAGGATAAGCTAGC | 87539473 | 87539495 | 0.01 |
| GTTATCCTATGTGATTCACAG | 87539482 | 87539504 | 0.25 |
| GCTGTCTCCTAAGCATTAAG | 87539554 | 87539576 | 0.26 |
| GACCCCAGCCCCTTAATGCTT | 87539561 | 87539583 | 0.03 |
| GCATTAAGGGGCTGGGGTTG | 87539566 | 87539588 | 0.00 |
| GTTGTGGGATGCTGAGTCTT | 87539582 | 87539604 | 0.01 |
| GAGTCTTAGGATAATCAAAA | 87539595 | 87539617 | 0.01 |
| GTCACTCCAGTCATTTATTCC | 87539620 | 87539642 | 0.02 |
| GACTGGTCTTATGATCTGC | 87539657 | 87539678 | 0.00 |
| GAATAGCGAGAGAGAACTACT | 87539708 | 87539730 | 0.04 |
| GTCTAGAGATCTCCAGAGA | 87539763 | 87539784 | 0.01 |
| GTCTCTCTGATCTTCCTTCTC | 87539775 | 87539797 | 0.04 |
| GAGAAAGAACACAGAACAA | 87539794 | 87539815 | 0.01 |
| GACAGAACAAAGGAAGTAGAC | 87539804 | 87539826 | 0.01 |
| GATCTGTCTCTAGCAGAGCGC | 87539829 | 87539851 | 12.82 |
| GCAGAGCGCAGGCCGTCAGCT | 87539841 | 87539863 | 1.07 |
| GACAGCTAGAAGTCAAATCAT | 87539865 | 87539887 | 0.02 |
| GTTCTGAGAAAGAGGTGTCT | 87539902 | 87539924 | 0.03 |
| GGAGAGCGGTTCTGAGAAAG | 87539910 | 87539932 | 0.08 |
| GCAGAACCGCTCTCCAAGTTG | 87539919 | 87539941 | 0.14 |
| GCTAAGGACCAGCCTCAACT | 87539931 | 87539953 | 0.03 |
| GCTCTTTAGTTACGTTTGCTA | 87539947 | 87539969 | 0.01 |
| GACTAAAGAGAATCATCATGC | 87539961 | 87539983 | 0.01 |
| GCATCATGCAGGAGCTAGAGA | 87539973 | 87539995 | 0.01 |
| GACCTATACTGTGAACACTCT | 87540008 | 87540030 | 0.67 |
| GAACACTCTCGGGGCCGAAG | 87540019 | 87540041 | 12.18 |
| GTTTGTGCTATCTTCCCTCTT | 87540033 | 87540055 | 0.01 |
| GGGAAGATAGCACAAACCCA | 87540040 | 87540062 | 0.21 |
| GCACAAACCCAAGGGCACCC | 87540049 | 87540071 | 0.01 |
| GTGTGGACCAGGGTGCCCTT | 87540056 | 87540078 | 0.01 |
| GGCACCCTGGTCCACACAGC | 87540062 | 87540084 | 0.64 |
| GTAGAACCTGCTGTGTGGACC | 87540067 | 87540089 | 4.00 |
| GGGTCCTAGAACCTGCTGTG | 87540073 | 87540095 | 0.03 |
| GCTTTTTGTTTTTGTGGGGCA | 87540093 | 87540115 | 0.01 |
| GTTTTACTTTTTGTTTTTGT | 87540099 | 87540121 | 0.01 |
| GAATAAATAAAAAAGTTATC | 87540123 | 87540145 | 0.04 |
| GAAAAAGTTATCTGGTGACTT | 87540132 | 87540154 | 0.39 |
| GGTGACTTCGGGAGAAGTG | 87540144 | 87540165 | 0.70 |
| GTGGCCACTGTCGCTAATTT | 87540162 | 87540184 | 0.00 |
| GGTGGCAAGGGAAGTGTAAA | 87540190 | 87540212 | 0.01 |
| GCAGTGGGGTGTGGGGTGGCA | 87540203 | 87540225 | 0.01 |
| GTACTGACACAGTGGGGTGT | 87540212 | 87540234 | 0.01 |
| GACATGAGTACTGACACAGT | 87540219 | 87540241 | 0.00 |
| GGGTTGGCAGGGTCAGAGGA | 87540256 | 87540278 | 0.01 |
| GTTTGGGGTTGGCAGGGTCAG | 87540260 | 87540282 | 0.01 |
| GAAGTGCATTTGGGGTTGGCA | 87540267 | 87540289 | 0.02 |
| GACAGAAAGTGCATTTGGGGT | 87540272 | 87540294 | 0.11 |
| GTAGAGACAGAAAGTGCATT | 87540278 | 87540300 | 0.48 |
| GTTACATGATGTGTTCAGAAC | 87540308 | 87540330 | 0.03 |

| | | | |
|---|---|---|---|
| GAATACAACCATATATTTTGC | 87540330 | 87540352 | 0.01 |
| GCACAAAACCTGCAAAATATA | 87540337 | 87540359 | 0.02 |
| GGCACAGTATGAATGAGTC | 87540374 | 87540395 | 0.17 |
| GAACTGGAGTAACACTACGTA | 87540394 | 87540416 | 0.02 |
| GTTCTTTTTCTTGATGAAGA | 87540414 | 87540436 | 0.02 |
| GAAATGTGGCTATTAATATAA | 87540439 | 87540461 | 0.23 |
| GAGTGGATAAAAGAAAATG | 87540454 | 87540475 | 0.02 |
| GAATGTCTATCAACTAATGAG | 87540470 | 87540492 | 0.25 |
| GTTTTGATCATAGTATAGATT | 87540510 | 87540532 | 0.02 |
| GCTATACTATGATCAAAATCA | 87540516 | 87540538 | 0.18 |
| GAACTATCAACACTTAGGTTT | 87540588 | 87540610 | 0.02 |
| GCACATAAACTATCAACACTT | 87540594 | 87540616 | 1.31 |
| GTTTATGTGGCCTACACATT | 87540608 | 87540630 | 0.01 |
| GTCACCAAGGACCAAATGTGT | 87540618 | 87540640 | 0.01 |
| GTCCTTGGTGAGAACACTTCC | 87540631 | 87540653 | 0.01 |
| GAACACTTCCTGGTGTGTTG | 87540641 | 87540663 | 0.23 |
| GTCAACAGCCCTCAACACACC | 87540649 | 87540671 | 0.01 |
| GACAAGTGTTCTGTGTAGCC | 87540746 | 87540768 | 0.01 |
| GTCTGTGTAGCCCGGTCATTC | 87540755 | 87540777 | 0.18 |
| GATTAAGTTCCAGAATGACC | 87540764 | 87540786 | 2.98 |
| GACTTAATCTATAGACCAGGC | 87540779 | 87540801 | 0.03 |
| GACTTGGGTGACAACAGAGGC | 87540821 | 87540843 | 0.02 |
| GAAAAATAAGGGCAGGCATGG | 87540864 | 87540886 | 0.00 |
| GAAGAACATAAAAATAAGGGC | 87540872 | 87540894 | 0.01 |
| GTATCTGTGTATCTGAGAGT | 87540902 | 87540924 | 0.02 |
| GTATCTGAGAGTAGGCAACTG | 87540911 | 87540933 | 0.02 |
| GCAACTGTGGAGGCCAGACA | 87540924 | 87540946 | 0.00 |
| GACAGATCTAAAGTCCATGTC | 87540937 | 87540959 | 0.05 |
| GACTTTAGATCTGTCAGAGG | 87540946 | 87540968 | 0.42 |
| GCAGTTGTAAATAGCCAGATG | 87540978 | 87541000 | 0.04 |
| GAAATAGCCAGATGTGGGTGC | 87540985 | 87541007 | 0.01 |
| GTTTTCCAGAGGACATGAATT | 87541011 | 87541033 | 0.00 |
| GAGATGAAATAGCTAATAA | 87541044 | 87541065 | 0.26 |
| GATTAATTATATTTAGCACT | 87541079 | 87541101 | 0.02 |
| GATAGAAAGGAAAAATGTAAA | 87541139 | 87541161 | 0.03 |
| GACACATTGTCTTGTAGTATA | 87541188 | 87541210 | 0.15 |
| GCACTTTTTAAGTCAAAATAA | 87541235 | 87541257 | 0.02 |
| GTAAGTCAAAATAATGGTTGA | 87541242 | 87541264 | 0.03 |
| GAAAATAATGGTTGATGGCCC | 87541248 | 87541270 | 1.25 |
| GCAAATAGAAAGAAAAGTCCT | 87541265 | 87541287 | 12.52 |
| GATGAGAAATCTTTGGTTTCT | 87541318 | 87541340 | 0.00 |
| GTTTAATTAATGAGAAATCTT | 87541326 | 87541348 | 0.02 |
| GTAAATGTTCTATCCTAGTCT | 87541349 | 87541371 | 0.02 |
| GGACTTTCAGACCCAAGACT | 87541361 | 87541383 | 0.02 |
| GTCTTGGGTCTGAAAGTCCTT | 87541366 | 87541388 | 2.10 |
| GAAATTCTCAAGATTCTCCAA | 87541382 | 87541404 | 0.02 |
| GATTGCAATTGTGAGACAATA | 87541412 | 87541434 | 0.02 |
| GTTAAAAGTTTGCCAGACAAA | 87541447 | 87541469 | 0.03 |
| GAATTCAAATTGCCATTTGTC | 87541458 | 87541480 | 0.01 |
| GTTGAATTTTTCCATGATCAT | 87541474 | 87541496 | 0.01 |
| GACTTGACTTCCAATGATCA | 87541484 | 87541506 | 0.09 |
| GATGAACAAGGAGGGAAAAAT | 87541557 | 87541579 | 0.75 |

| | | | |
|---|---|---|---|
| GCAGCAAATACATGAACAAGG | 87541567 | 87541589 | 0.01 |
| GTTCTAGTAACTTTAAACATT | 87541623 | 87541645 | 0.02 |
| GTTGGTAGTGGTTTAAGGATT | 87541657 | 87541679 | 0.45 |
| GCTAGTGTTGGTAGTGGTTTA | 87541663 | 87541685 | 0.00 |
| GAATTTCCTAGTGTTGGTAG | 87541670 | 87541692 | 0.14 |
| GAATTTGAATTTCCTAGTGT | 87541676 | 87541698 | 0.02 |
| GCTAGGAAATTCAAATTCACT | 87541682 | 87541704 | 0.03 |
| GTAATGTACATCTTATTACTT | 87541714 | 87541736 | 0.02 |
| GACAGTCCCTATGACAGAATC | 87541736 | 87541758 | 0.01 |
| GTTTAGCCTGATTCTGTCATA | 87541741 | 87541763 | 0.03 |
| GAAAATCAAAATTACTTCATG | 87541818 | 87541840 | 0.01 |
| GAAATTACTTCATGTGGGGGC | 87541825 | 87541847 | 0.02 |
| GAACAAAAGACAACTACTTCA | 87542023 | 87542045 | 2.29 |
| GACAACTACTTCATGGGGCC | 87542030 | 87542052 | 0.09 |
| GCTACTTCATGGGGCCAGGAA | 87542035 | 87542057 | 1.32 |
| GAAGGCATGCATTACCATTCC | 87542048 | 87542070 | 0.01 |
| GTCTTAAATTCTGGGGTTAAA | 87542067 | 87542089 | 0.02 |
| GCCTCTGCCTCTTAAATTCTG | 87542075 | 87542097 | 0.02 |
| GAGGTATGTAGATCTCTGTG | 87542094 | 87542116 | 0.02 |
| GTCTGGACTACAGAGTTCCA | 87542126 | 87542148 | 0.03 |
| GTCTCTCTATATAACTGCCC | 87542143 | 87542165 | 0.00 |
| GCTTCTTTTGCTTTTGAGACA | 87542165 | 87542187 | 0.01 |
| GAAGCAAAGAGCCTTAATGTG | 87542185 | 87542207 | 0.01 |
| GAAGAGCCTTAATGTGAGGAC | 87542190 | 87542212 | 0.03 |
| GCACACCCAGTCCTCACATTA | 87542195 | 87542217 | 0.03 |
| GTAGAGCACTTGTCTAGCC | 87542229 | 87542250 | 0.44 |
| GAAGGATTGAAGTTGGACTCC | 87542246 | 87542268 | 0.03 |
| GTGATGCTAAGGATTGAAGT | 87542254 | 87542276 | 0.09 |
| GTCCTTAGCATCACATGGATC | 87542264 | 87542286 | 0.01 |
| GCATCACATGGATCAGGGTGC | 87542271 | 87542293 | 0.01 |
| GCCTTTGCGTCCTCAGTGCT | 87542308 | 87542330 | 39.70 |
| GACTGAGGACGCAAAGGCACG | 87542314 | 87542336 | 0.01 |
| GCAAAGGCACGAGGAACAGA | 87542323 | 87542345 | 0.01 |
| GCACGAGGAACAGAAGGCCA | 87542329 | 87542351 | 0.02 |
| GCAGAAGGCCAAGGGGATTCC | 87542339 | 87542361 | 1.08 |
| GGTATAACCAGGAATCCCCT | 87542346 | 87542368 | 0.01 |
| GTCAAATGTCCAGGTATAACC | 87542357 | 87542379 | 0.02 |
| GCAGGCTGGTCTCAAATGTCC | 87542367 | 87542389 | 1.12 |
| GATGTCTATTATATCCCAGGC | 87542382 | 87542404 | 0.01 |
| GCACAGCACATGAAGCTGGCT | 87542425 | 87542447 | 0.53 |
| GCTGTGTGACAGTTTGTACTT | 87542443 | 87542465 | 0.06 |
| GACAGTTTGTACTTAGGGTCC | 87542450 | 87542472 | 0.24 |
| GTAGGGTCCCGGTCACTTGAG | 87542462 | 87542484 | 0.08 |
| GCTTCATCCTCTCAAGTGAC | 87542469 | 87542491 | 0.04 |
| GAAGCAAGAGGATTACGTG | 87542487 | 87542508 | 0.01 |
| GCAACCTTATAGAAAGACCA | 87542520 | 87542542 | 0.09 |
| GTATACGAAAAGAAAAGTCCA | 87542537 | 87542559 | 0.02 |
| GTTTTCTTTTCGTATAAAAGA | 87542545 | 87542567 | 0.02 |
| GTATAAAAGATGGCTTATTTA | 87542556 | 87542578 | 0.02 |
| GAAGATGGCTTATTTAAGGCC | 87542561 | 87542583 | 0.30 |
| GAAGGCTTGTGCTACTGTGCC | 87542579 | 87542601 | 0.01 |
| GCCTACTGAGTGCGGGGGTG | 87542622 | 87542644 | 0.05 |

| | | | |
|---|---|---|---|
| GTATTTGCCTACTGAGTGCGG | 87542627 | 87542649 | 0.34 |
| GTAGGCAAATAAGATACATCT | 87542640 | 87542662 | 0.01 |
| GATACATCTTGGTGAGTTCA | 87542651 | 87542673 | 0.01 |
| GTAGGATTTGCTACAAAGACC | 87542678 | 87542700 | 0.00 |
| GTAGCAAATCCTAAGCCAGTC | 87542689 | 87542711 | 0.78 |
| GTATATATCCCTGACTGGCTT | 87542697 | 87542719 | 0.01 |
| GTCTCAGTATATATCCCTGAC | 87542703 | 87542725 | 0.00 |
| GTAAACATGTTTTTGAGAC | 87542727 | 87542748 | 0.02 |
| GTACTGTTGTGAGACTTGTGC | 87542746 | 87542768 | 0.02 |
| GCATGCCTGTGTAAATACTTG | 87542777 | 87542799 | 0.60 |
| GTAAATACTTGAGGCCAGAAA | 87542787 | 87542809 | 0.02 |
| GTCAAACTTGATGGCCATTTC | 87542800 | 87542822 | 0.01 |
| GGTAGATTATCAAACTTGA | 87542810 | 87542831 | 0.01 |
| GTCATAGGAATATGTAGACTG | 87542830 | 87542852 | 0.31 |
| GGGAGAGACTTTGCTTCAT | 87542846 | 87542867 | 0.01 |
| GAAACACAAGTCTAGGTTCA | 87542865 | 87542887 | 19.55 |
| GAAGAGGAAACACAAGTCT | 87542872 | 87542893 | 1.45 |
| GTTGTGTTTCCTCTTCTAGGA | 87542879 | 87542901 | 1.64 |
| GCTGACTACCATCCTAGAAG | 87542887 | 87542909 | 2.30 |
| GAAGGCAGGAGGATTGCTGG | 87542912 | 87542934 | 0.05 |
| GTCTAAGAGTGAGAAGGCAGG | 87542923 | 87542945 | 0.00 |
| GCCTTCTCACTCTTAGATCT | 87542929 | 87542951 | 0.09 |
| GACTCTTAGATCTGGGGCCAC | 87542937 | 87542959 | 0.01 |
| GGAGTCTCACACATGCCAG | 87542953 | 87542974 | 0.75 |
| GAGACTCCTGTTTTGTTGTT | 87542967 | 87542989 | 0.12 |
| GCAACACCCAAACAACAAAAC | 87542973 | 87542995 | 0.02 |
| GGGTGTTGAGATCTGCTCTC | 87542988 | 87543010 | 0.01 |
| GGTCTTTGCGACTGTAGAGT | 87543009 | 87543031 | 1.97 |
| GGAGGGGTGGTTCAGTGGTT | 87543036 | 87543058 | 0.05 |
| GGGTTGGAGGGGTGGTTCAG | 87543041 | 87543063 | 0.04 |
| GATGAAGACTAAGGGGTTGGA | 87543053 | 87543075 | 0.02 |
| GAAAATGAAGACTAAGGGGT | 87543057 | 87543079 | 0.01 |
| GCCTAGCAAAACAGGGGGAAA | 87543092 | 87543114 | 0.01 |
| GTCAAGCCCTAGCAAAACAG | 87543099 | 87543121 | 0.08 |
| GAAGGGCCTAGCATGAGGTTT | 87543123 | 87543145 | 0.01 |
| GCAGAGTAAGGGCCTAGCATG | 87543129 | 87543151 | 0.05 |
| GGGCATGTAGCTCAGAGTAA | 87543141 | 87543163 | 0.33 |
| GAACATGGGGGGTGGGGGGC | 87543162 | 87543184 | 0.02 |
| GTATAGAACATGGGGGGTGG | 87543167 | 87543189 | 0.02 |
| GTTAAGTGTATAGAACATGGG | 87543173 | 87543195 | 0.01 |
| GTTCTATACACTTAATTTTC | 87543181 | 87543203 | 0.01 |
| GACACTTAATTTTCTGGCATT | 87543188 | 87543210 | 0.03 |
| GGCATTTGGGGCTTTCATGT | 87543202 | 87543224 | 3.22 |
| GTAGGAAAGATTTCTCCTTC | 87543220 | 87543242 | 0.01 |
| GAAAAACTACTGAGGCCCAGA | 87543235 | 87543257 | 0.01 |
| GGCCTCAGTAGTTTTTAGAG | 87543242 | 87543264 | 0.13 |
| GCAACTTCCCACATTGAGCTT | 87543268 | 87543290 | 0.02 |
| GTATATCCAAAGCTCAATGT | 87543274 | 87543296 | 0.01 |
| GTACTTGGGGGTATGGCATC | 87543309 | 87543331 | 0.04 |
| GATGCCATACCCCCAAGTAC | 87543312 | 87543334 | 0.33 |
| GTTGGGTTCTCCAGTACTTGG | 87543321 | 87543343 | 0.04 |
| GAACTGGCATTAGGCTATTTT | 87543339 | 87543361 | 0.01 |

| | | | |
|---|---|---|---|
| GAAACAGAGCAAACTGGCATT | 87543349 | 87543371 | 0.00 |
| GAGGCAAAACAGAGCAAAC | 87543356 | 87543377 | 0.01 |
| GGAGGGAAATGTTATTTAG | 87543374 | 87543395 | 0.01 |
| GCTATTGAAGACAGGGACTC | 87543394 | 87543416 | 0.01 |
| GCAAAGTGGCTATTGAAGACA | 87543401 | 87543423 | 0.01 |
| GCAATAGCCACTTTGGCTTTT | 87543410 | 87543432 | 0.01 |
| GATTATTCCTAAAAGCCAAAG | 87543416 | 87543438 | 0.02 |
| GCTATATACATCTTTATGATA | 87543448 | 87543470 | 0.00 |
| GCATCTTTATGATAAGGAACT | 87543455 | 87543477 | 0.01 |
| GGAACTTGGTACTACCCTGT | 87543469 | 87543491 | 0.51 |
| GTCCCATGATGGCTCCCGAC | 87543484 | 87543506 | 0.02 |
| GATTATTAGCTTGTCCCATGA | 87543495 | 87543517 | 0.12 |
| GGTTATATAAAATCTGAAGC | 87543542 | 87543564 | 0.02 |
| GCATAAAAGCGAATCTGTCGC | 87543563 | 87543585 | 0.01 |
| GATTCGCTTTTATGAAGCA | 87543572 | 87543593 | 0.01 |
| GTTTATGAAGCAAGGCCGTAA | 87543580 | 87543602 | 0.18 |
| GCCGTAAAGGAATTCATTTT | 87543593 | 87543615 | 0.04 |
| GAAAGGAATTCATTTTAGGAA | 87543598 | 87543620 | 0.01 |
| GTAAGTAATAGCACGCCCCTT | 87543675 | 87543697 | 0.03 |
| GAAGACAGTACTGCTCCAAAG | 87543689 | 87543711 | 1.40 |
| GTAAAAAAACTTAATAATCAC | 87543787 | 87543809 | 0.03 |
| GTAAATAATTGAACTGCTATT | 87543808 | 87543830 | 0.02 |
| GTTACACCTTCAGTAAACCTG | 87544072 | 87544094 | 0.09 |
| GTAAACCTGTGGAGCTGAGAG | 87544084 | 87544106 | 0.03 |
| GAAAGATGTATAGGTATTG | 87544242 | 87544263 | 0.18 |
| GTTGAGGACAAAGAGATGAGA | 87544258 | 87544280 | 0.02 |
| GAAAAATCTGTAAGCAGGGAA | 87544301 | 87544323 | 0.03 |
| GTGGGGAAAAATCTGTAAGC | 87544307 | 87544329 | 0.01 |
| GAAAGCAGAAAGATACAAGT | 87544325 | 87544347 | 0.01 |
| GCTATTTGAAGGAAGAAAAA | 87544417 | 87544439 | 0.03 |
| GACTTTCAACTTGCTATTTGA | 87544428 | 87544450 | 0.02 |
| GCTTTCTCAGCAGGAACTGTG | 87544456 | 87544478 | 0.48 |
| GTCTTTGTACTTTCTCAGC | 87544466 | 87544487 | 0.01 |
| GAACTGCAGTTTCTTGCACTT | 87544523 | 87544545 | 0.03 |
| GTGCAAGAAACTGCAGTTTC | 87544528 | 87544550 | 2.66 |
| GTCTGGCCTCAGGATCACAGA | 87544546 | 87544568 | 0.02 |
| GATCTGCCTTCTGTGATCCTG | 87544551 | 87544573 | 0.12 |
| GTTTGTCTTACAAGGTATTTA | 87544632 | 87544654 | 0.00 |
| GGGCAAGACTTTGTCTTACA | 87544641 | 87544663 | 0.01 |
| GACAAAGTCTTGCCCTCGG | 87544649 | 87544670 | 0.15 |
| GATGGGGAAGAGTGTCCGCCG | 87544662 | 87544684 | 0.22 |
| GTTCTTATTGAGGCAGCAAA | 87544681 | 87544703 | 0.02 |
| GTTTCTTGTTGGTTCTTATTG | 87544691 | 87544713 | 0.01 |
| GGGGGATCTTCTTTCTTGT | 87544703 | 87544724 | 0.01 |
| GCCAGGGGCTAGCTGCAGTG | 87544721 | 87544743 | 1.79 |
| GTCACTTTGGGGTTGTGCCA | 87544737 | 87544759 | 0.02 |
| GGGGTGATCTTGTCACTTTG | 87544748 | 87544770 | 0.00 |
| GTTTTGTGTGGTCTCACTGT | 87544768 | 87544790 | 0.01 |
| GGAATAGCTCTTGTTTTGTG | 87544780 | 87544802 | 0.01 |
| GAAAACAAGAGCTATTCCCCA | 87544786 | 87544808 | 2.32 |
| GCAGCCACTGCTCCCATCCT | 87544803 | 87544825 | 0.00 |
| GAGGTGATGGCTGATGCTCT | 87544828 | 87544850 | 0.03 |

| | | | |
|---|---|---|---|
| GCCATCACCTCAGGAGAACC | 87544840 | 87544862 | 0.01 |
| GGAGCAGCCTGGTTCTCCTG | 87544847 | 87544869 | 0.01 |
| GACCAGGCTGCTCCCTTCAAG | 87544857 | 87544879 | 0.00 |
| GTACCACTGGCTCCACTTGA | 87544869 | 87544891 | 0.00 |
| GTCTGGAGAAGTGGGTACCAC | 87544882 | 87544904 | 0.01 |
| GAAGACACGCTTCTGGAGAAG | 87544892 | 87544914 | 0.00 |
| GCTGGTTCTAAGACACGCTTC | 87544900 | 87544922 | 0.01 |
| GCATGGAGCATTTGTTCAGTC | 87544919 | 87544941 | 0.00 |
| GAAATGCTCCATGAGACTCAC | 87544930 | 87544952 | 0.01 |
| GCTCAGACCAGTGAGTCTCA | 87544937 | 87544959 | 1.98 |
| GAGCAAGGCCAGCTGCCCTG | 87544956 | 87544978 | 0.00 |
| GCATATGTCCCACAGGGCAGC | 87544964 | 87544986 | 0.01 |
| GAAGGTTACATATGTCCCAC | 87544972 | 87544994 | 0.01 |
| GGGACATATGTAACCTTCTG | 87544977 | 87544999 | 0.00 |
| GAGGACTCATGACCCACAGA | 87544990 | 87545012 | 0.27 |
| GGGTCATGAGTCCTCCCTGT | 87544998 | 87545020 | 0.01 |
| GGGAGCACACAGGTCCCACA | 87545012 | 87545034 | 0.01 |
| GGGACCTGTGTGCTCCCTGT | 87545018 | 87545040 | 0.45 |
| GCTGGGGTGCACATGTCCCAC | 87545033 | 87545055 | 0.01 |
| GAGAACCAAAGGAGATAACT | 87545051 | 87545073 | 0.68 |
| GTTATCTCCTTTGGTTCTCTC | 87545056 | 87545078 | 0.01 |
| GGAAATGTTGAAGATCTGT | 87545081 | 87545102 | 0.02 |
| GACGGCAGTTCAGTCTTCAG | 87545103 | 87545125 | 0.01 |
| GATCATATCGGGAAGACAGGA | 87545121 | 87545143 | 0.01 |
| GTTCATAAATGTGATCATATC | 87545133 | 87545155 | 0.01 |
| GATCAATGGTGAGCAAGAGAA | 87545158 | 87545180 | 0.14 |
| GCCAATGAAAGGGACAATCAA | 87545173 | 87545195 | 0.01 |
| GTCCCTTTCATTGGCATAAT | 87545182 | 87545204 | 0.01 |
| GCATTGGCATAATGGGATGGT | 87545190 | 87545212 | 0.01 |
| GCCTAATCCATTGAGAGACT | 87545217 | 87545239 | 0.01 |
| GCCTGCATCCAAGTCTCTCAA | 87545224 | 87545246 | 0.01 |
| GCTTATGCTGTTAGGATTTT | 87545254 | 87545276 | 0.01 |
| GAAGGAGATGCTTATGCTGTT | 87545262 | 87545284 | 0.03 |
| GAAGAGTGTGAAGATTGATAA | 87545281 | 87545303 | 0.01 |
| GGGATGGTATCTGGTTGGAA | 87545308 | 87545330 | 0.03 |
| GTCTAGGGATGGTATCTGGT | 87545313 | 87545335 | 0.01 |
| GATACCATCCCTAGACCCAG | 87545320 | 87545342 | 0.02 |
| GGGAAGGGCCTCTGGGTCTA | 87545328 | 87545350 | 0.63 |
| GAAGAACCCGGGAAGGGCCTC | 87545336 | 87545358 | 0.11 |
| GTAGATGGAAGAACCCGGGAA | 87545343 | 87545365 | 0.59 |
| GTAGGATTAGATGGAAGAACC | 87545349 | 87545371 | 0.02 |
| GTCAGTCATCTTAGGATTAGA | 87545359 | 87545381 | 0.01 |
| GGCTGCACTCAGTCATCTT | 87545368 | 87545389 | 1.23 |
| GCAGGAATGGGTGATGCAGGC | 87545388 | 87545410 | 1.58 |
| GGGTCTTCCCAGGCAGGAAT | 87545401 | 87545423 | 0.02 |
| GTTTGTGGGGTCTTCCCAGGC | 87545407 | 87545429 | 0.10 |
| GGAAGACCCCACAAAGTGGC | 87545415 | 87545437 | 0.01 |
| GACCCCACAAAGTGGCTGGAC | 87545420 | 87545442 | 0.03 |
| GAAGGCATCAAGGTGGGCAGG | 87545455 | 87545477 | 0.05 |
| GCCACCGGAAGGCATCAAGGT | 87545462 | 87545484 | 1.60 |
| GATGCCTTCCGGTGGGGTTC | 87545470 | 87545492 | 0.01 |
| GGCTCTTTCCAGAACCCCAC | 87545478 | 87545500 | 0.07 |

| | | | |
|---|---|---|---|
| GAGCCTCGTCTCCTGTTTTT | 87545496 | 87545518 | 0.01 |
| GATTCACGGATCCCTAAAAAC | 87545507 | 87545529 | 0.02 |
| GTAGAAGGAAGCTTATATTCA | 87545522 | 87545544 | 0.05 |
| GCATGAAGGATTATCTAGA | 87545538 | 87545559 | 0.10 |
| GGATGGGAGACACAGCATGA | 87545551 | 87545573 | 0.58 |
| GTGTTTGTTTTAATCAGGAT | 87545567 | 87545589 | 0.03 |
| GACTTAGTGTTTGTTTTAATC | 87545572 | 87545594 | 0.01 |
| GCAAACGATCGATGTAATTTC | 87545614 | 87545636 | 0.04 |
| GTTTGCTCTCGAGAGGAGAAA | 87545646 | 87545668 | 0.02 |
| GATCCCCGTTTGCTCTCGAG | 87545654 | 87545676 | 0.07 |
| GCAAACGGGGATCCAGCAGGC | 87545665 | 87545687 | 0.01 |
| GTGGAGCATTCCGGCCTGC | 87545676 | 87545697 | 0.00 |
| GTGTTCAGAGTGGAGCATTC | 87545684 | 87545706 | 0.02 |
| GCCAAAGTATCGTGTTCAGAG | 87545694 | 87545716 | 0.00 |
| GAACACGATACTTTGGCAGA | 87545701 | 87545723 | 0.01 |
| GATTAAGTGTCAGACTGCAG | 87545743 | 87545765 | 0.75 |
| GATGCACTGACTTAATGATGA | 87545780 | 87545802 | 0.44 |
| GACCTAGGAAAGAGATAAAT | 87545829 | 87545851 | 0.21 |
| GAAGGGGGAAAAGGGAGACCT | 87545844 | 87545866 | 0.02 |
| GCTTCTTTCCAAGGGGGAAAA | 87545853 | 87545875 | 0.02 |
| GTAGCCCCTTCTTTCCAAGG | 87545860 | 87545882 | 0.15 |
| GCTGAGACCATTTTTCCCAG | 87545892 | 87545914 | 0.58 |
| GCAAGTTTCCACTGGGAAAAA | 87545899 | 87545921 | 0.01 |
| GTTGCAGACTCAAGTTTCCAC | 87545908 | 87545930 | 0.24 |
| GCATATAGGGCTGTTTTATA | 87545937 | 87545959 | 0.23 |
| GGGAGAGGTAAAGGCATATA | 87545950 | 87545972 | 0.06 |
| GTATATATGGGGAGAGGTAA | 87545959 | 87545981 | 0.01 |
| GGGATTGTATATATGGGGAG | 87545965 | 87545987 | 0.36 |
| GATTCAGGGATTGTATATATG | 87545970 | 87545992 | 0.02 |
| GCTATGAAAGGCACTAATTC | 87545986 | 87546008 | 0.52 |
| GAAAAGAGTTCAGGCTATGAA | 87545998 | 87546020 | 0.02 |
| GTTTTTAAAGGAAAAGAGTTC | 87546008 | 87546030 | 0.01 |
| GCACATAATTTTTTTTTTAA | 87546021 | 87546043 | 0.02 |
| GATACCTTTAATTCTAGCATT | 87546060 | 87546082 | 0.05 |
| GACCAGGCAGACCTTGAACT | 87546104 | 87546126 | 0.06 |
| GAGTGAGTTCCAGCAAAATC | 87546131 | 87546153 | 0.06 |
| GCTGTGTAACCCTGATTTTGC | 87546140 | 87546162 | 0.01 |
| GTGTCTGTGTGTTTGAGACA | 87546168 | 87546190 | 0.02 |
| GACACAGGTACACACACACAT | 87546241 | 87546263 | 0.01 |
| GACATGATTTTATATACACAC | 87546257 | 87546279 | 0.02 |
| GTATATAAAATCATGTATCAG | 87546265 | 87546287 | 0.03 |
| GAATCATGTATCAGAGGACAA | 87546272 | 87546294 | 0.01 |
| GATGACTGCTTGTTACTAAAA | 87546301 | 87546323 | 0.22 |
| GCAGTCATGAGCTGTTGAGTG | 87546317 | 87546339 | 0.79 |
| GAGCTGTTGAGTGTGGGTTC | 87546324 | 87546346 | 0.01 |
| GTTCAGCAGGTACTGCTCATG | 87546365 | 87546387 | 0.35 |
| GATGGTTAATCACATTCAGC | 87546379 | 87546401 | 0.01 |
| GTCTGGGGAGATGGTTTGAGA | 87546397 | 87546419 | 0.01 |
| GAAAAAGGGGGTCTGGGGAGA | 87546407 | 87546429 | 3.29 |
| GAAATATAAAAAGGGGGTCT | 87546414 | 87546436 | 0.06 |
| GTTGTAAAGAAATATAAAAAG | 87546421 | 87546443 | 0.04 |
| GATTACACACATATTGTGTTG | 87546443 | 87546465 | 0.00 |

| | | | |
|---|---|---|---|
| GCACATATTGTGTTGTGGGTG | 87546449 | 87546471 | 0.01 |
| GGGTGTTTTCAGCTACACCA | 87546473 | 87546495 | 1.12 |
| GGACCTCTACACACACGCCA | 87546490 | 87546512 | 0.02 |
| GCGTGTGTGTAGAGGTCCAA | 87546495 | 87546517 | 0.02 |
| GAGGTCCAAAGGCGACCTTC | 87546506 | 87546528 | 0.03 |
| GCCAATCCCGAAGGTCGCCTT | 87546511 | 87546533 | 0.06 |
| GAAGGAAGAACCCAATCCCGA | 87546521 | 87546543 | 1.88 |
| GGTTCTTCCTTCCACAGTGC | 87546533 | 87546555 | 0.03 |
| GCCAGGGACCTGCACTGTGGA | 87546540 | 87546562 | 0.27 |
| GCAGTGCAGGTCCCTGGGTCT | 87546547 | 87546569 | 0.60 |
| GCTGTTACTGAGCCTAGACCC | 87546558 | 87546580 | 0.00 |
| GTACATGCTGAGCCTTCTCTC | 87546588 | 87546610 | 0.00 |
| GCAAGAAGGAGCCCTGAGAGA | 87546599 | 87546621 | 0.08 |
| GTTTTAACAGAGGACTCAAGA | 87546614 | 87546636 | 0.20 |
| GTTTTCACACCATTTTAACAG | 87546625 | 87546647 | 0.01 |
| GTAGGGTCGTTAGGGTCGTTA | 87546648 | 87546670 | 0.62 |
| GTAGGGTCATTAGGGTCGTTA | 87546657 | 87546679 | 6.40 |
| GAAGAGGTCGTTAGGGTCATT | 87546667 | 87546689 | 0.01 |
| GAAACTCCGAAGAGGTCGTT | 87546676 | 87546698 | 1.98 |
| GAAATAAGTGAAACTCCGAAG | 87546684 | 87546706 | 0.03 |
| GACCAAATGAACAGGTATAC | 87546749 | 87546771 | 0.01 |
| GAATTAGATGACCAAATGAAC | 87546757 | 87546779 | 0.03 |
| GTCATTTGGTCATCTAATTCA | 87546762 | 87546784 | 0.01 |
| GTCTCCTAGGTTCTGTTTGG | 87546786 | 87546808 | 1.50 |
| GAACTTCTCTCTTGGTCTCCT | 87546799 | 87546821 | 0.02 |
| GGAAAGAAAAACTTCTCTCT | 87546808 | 87546830 | 0.04 |
| GCATAATTTATTAACGGTGCA | 87546829 | 87546851 | 0.02 |
| GTTGGGCACATAATTTATTAA | 87546836 | 87546858 | 0.10 |
| GTAAATTATGTGCCCAACAAC | 87546843 | 87546865 | 0.02 |
| GAGCTTTTATGCCTGTTGTT | 87546854 | 87546876 | 1.40 |
| GTTACAAAGTATCTTTATAAA | 87546879 | 87546901 | 0.03 |
| GAACGAAGCATAAAATTTGG | 87546936 | 87546958 | 1.80 |
| GGAGGTACGACTTTAGCCC | 87546954 | 87546975 | 0.27 |
| GTCTGCTCTTGTATTTGACC | 87546971 | 87546993 | 0.51 |
| GCTTTATTTGTGTTCTTCTGA | 87547006 | 87547028 | 0.00 |
| GAATAAAGGCAAGCAAACTGT | 87547022 | 87547044 | 0.01 |
| GCATGCTATGCCAGAAACAT | 87547047 | 87547069 | 0.00 |
| GCATGTGTCCGCCTATGTTTC | 87547057 | 87547079 | 3.83 |
| GACAATACATTCTAACACTG | 87547089 | 87547111 | 2.06 |
| GCAGCTAGGTATCAGGGGGAT | 87547155 | 87547177 | 0.01 |
| GTTTCCGACAGCTAGGTATCA | 87547162 | 87547184 | 0.13 |
| GACCTAGCTGTCGGAAAACAC | 87547169 | 87547191 | 0.01 |
| GCTGATATTGGTAATTAAGAT | 87547202 | 87547224 | 0.01 |
| GTGATCTATGAGCTGATAT | 87547215 | 87547236 | 0.01 |
| GCTCATAGATCACACATCAA | 87547224 | 87547246 | 0.01 |
| GAACGTAGTTATATGTGTATA | 87547250 | 87547272 | 0.02 |
| GACCACAGCAGACAAAGTTCA | 87547288 | 87547310 | 0.01 |
| GCAGTCTGAACAAGCCACG | 87547320 | 87547341 | 0.11 |
| GCTACTGACTTGCTCCTCG | 87547334 | 87547355 | 0.01 |
| GATGCAGAAGTAAGTCATGG | 87547361 | 87547383 | 0.10 |
| GTCTGCATCTGCTCCTGCCTA | 87547376 | 87547398 | 0.07 |
| GAAGGGCAGGAACCCGTAGGC | 87547388 | 87547410 | 0.02 |

| | | | |
|---|---|---|---|
| GGACAGGAACTCAAAGGGC | 87547402 | 87547423 | 0.01 |
| GCAGCCAGGACAGGAACTCAA | 87547407 | 87547429 | 0.07 |
| GTCACTGAAGCCAGCCAGGAC | 87547417 | 87547439 | 0.01 |
| GTTCCTCACTGAAGCCAGCC | 87547422 | 87547444 | 0.00 |
| GAGGAAAGGAAGGGTTTACT | 87547464 | 87547486 | 0.01 |
| GCAAGTTGAGGAGGAAAGGA | 87547474 | 87547496 | 0.01 |
| GTAACCCCAACTAAATCATC | 87547527 | 87547549 | 1.28 |
| GAAATCATCAGGACAGAGTCC | 87547539 | 87547561 | 0.01 |
| GTCCAGGTCTGCTTAGAAAA | 87547555 | 87547577 | 0.94 |
| GCTTAGAAAATGGAGTCTCT | 87547565 | 87547587 | 0.02 |
| GAAAATGGAGTCTCTCGGGGC | 87547571 | 87547593 | 0.06 |
| GAGTCTCTCGGGGCAGGGGA | 87547577 | 87547599 | 0.01 |
| GCACAAAACACTGGTTTTTC | 87547600 | 87547622 | 0.03 |
| GAACCAGTGTTTTGTGCAGGA | 87547607 | 87547629 | 0.01 |
| GTTTTGTGCAGGATGGAGGC | 87547614 | 87547636 | 0.16 |
| GCCTTTCTGACAGCTGAGGGG | 87547643 | 87547665 | 0.19 |
| GCTGTCAGAAAGGGGATTGA | 87547653 | 87547675 | 0.01 |
| GCTTTTGAACTCTTAGCTTC | 87547675 | 87547697 | 1.58 |
| GTCAGGTAGATTAAGCATTTC | 87547693 | 87547715 | 0.00 |
| GCCTGGCTTGCTTTCATCTGA | 87547726 | 87547748 | 2.04 |
| GCAAGCCAGGGTGGCAGTTT | 87547739 | 87547761 | 0.01 |
| GGGATCATTCTAGTTACCTC | 87547760 | 87547782 | 0.01 |
| GATTCTAGTTACCTCTGGATC | 87547766 | 87547788 | 0.01 |
| GCAGATGCTTTCCAGATCCAG | 87547776 | 87547798 | 0.01 |
| GAAGCATCTGTTTGTTGAGAC | 87547790 | 87547812 | 0.02 |
| GCTTATACTGTAGCCTAGGTC | 87547815 | 87547837 | 0.01 |
| GTACTGTAGCCTAGGTCAGGA | 87547819 | 87547841 | 0.01 |
| GTTTGAGGCCATCCTGACCT | 87547827 | 87547849 | 0.01 |
| GATCTTCCTATCTCAGAGTGC | 87547858 | 87547880 | 0.04 |
| GCAACCCCAGCACTCTGAGAT | 87547863 | 87547885 | 0.01 |
| GCAACGTCTGAGGTGCTGGTG | 87547896 | 87547918 | 1.82 |
| GACTAGCAACGTCTGAGGTGC | 87547901 | 87547923 | 0.90 |
| GTAAACGACTAGCAACGTCTG | 87547907 | 87547929 | 0.17 |
| GCTAGTCGTTTATGTTGTCTG | 87547919 | 87547941 | 0.43 |
| GTCTGTGGTTGGGTGTTGCA | 87547934 | 87547956 | 0.84 |
| GACAGCCTGCTTGACCATTC | 87547956 | 87547978 | 1.48 |
| GCTTGACCATTCAGGAGTGA | 87547964 | 87547986 | 0.01 |
| GTCAGTCCCTTCACTCCTGAA | 87547970 | 87547992 | 0.45 |
| GAACAGCTTTTGACTGCGACT | 87547993 | 87548015 | 0.01 |
| GATATCCCGTGACTGAGAGAT | 87548016 | 87548038 | 0.01 |
| GTTCCCCGATCTCTCAGTCA | 87548021 | 87548043 | 0.01 |
| GATCGGGGAACAGATAGGCA | 87548033 | 87548055 | 0.05 |
| GATAGGCAAGGTCATCACAG | 87548045 | 87548067 | 0.12 |
| GGTCATCACAGTGGTCAGC | 87548054 | 87548075 | 0.01 |
| GTATTTTAATCAGATTACAGC | 87548077 | 87548099 | 0.01 |
| GATCAACACATCTCAAGAAAG | 87548099 | 87548121 | 0.00 |
| GCTCAAGAAAGAGGAGGCCAT | 87548109 | 87548131 | 0.01 |
| GAAAGAGGAGGCCATTGGGG | 87548114 | 87548136 | 1.81 |
| GAACTCAGCCCTCCTCCCCAA | 87548125 | 87548147 | 0.01 |
| GACTATGTAAAGACTTGGGAG | 87548150 | 87548172 | 1.11 |
| GTGACAACTATGTAAAGACT | 87548156 | 87548178 | 0.00 |
| GACATAGTTGTCACCTGACCC | 87548166 | 87548188 | 0.24 |

| | | | |
|---|---|---|---|
| GTTGCTTTAATGACCAGGGTC | 87548178 | 87548200 | 0.78 |
| GAAGTTTGCTTTAATGACCA | 87548183 | 87548205 | 0.30 |
| GACTTCCCATAAATGTTCATG | 87548201 | 87548223 | 0.00 |
| GTAAATGTTCATGAGGTTGGA | 87548209 | 87548231 | 0.01 |
| GACTAGCATATGTTCAATAAA | 87548253 | 87548275 | 0.03 |
| GTGTGGACTGACTCTTTCCA | 87548274 | 87548296 | 0.09 |
| GCCTAACAGAAACTGAGTCCC | 87548291 | 87548313 | 4.19 |
| GTTTCTGTTAGGGTCAGAGAG | 87548303 | 87548325 | 0.02 |
| GTAAACTCGTCCACGCAGTGT | 87548333 | 87548355 | 0.01 |
| GTATCACCAACCTACACTGCG | 87548342 | 87548364 | 0.19 |
| GCAGTGTAGGTTGGTGATACA | 87548347 | 87548369 | 0.02 |
| GGTTGGTGATACATGGTTTT | 87548354 | 87548376 | 0.41 |
| GATGGTTTTTGGGTGACTGTG | 87548366 | 87548388 | 0.87 |
| GGGTGACTGTGAGGACTGTA | 87548375 | 87548397 | 0.01 |
| GCTGTGAGGACTGTAGGGGAA | 87548381 | 87548403 | 0.04 |
| GGCTGACTAACTCATGGATT | 87548409 | 87548431 | 0.25 |
| GCAGCAGGGCTGACTAACTCA | 87548415 | 87548437 | 0.25 |
| GTAGTCAGCCCTGCTGACAGA | 87548423 | 87548445 | 0.01 |
| GTAACTGCCCATCTGTCAGCA | 87548430 | 87548452 | 0.15 |
| GACAGATGGGCAGTTAGCTT | 87548437 | 87548459 | 0.01 |
| GAAAGAGCTTGCTGCTCTTCC | 87548471 | 87548493 | 0.01 |
| GAATTGCCTGACATGAGTGC | 87548511 | 87548533 | 0.02 |
| GTTCACAACCACCAGTAACTC | 87548531 | 87548553 | 0.62 |
| GCATGAAGCCAGAGTTACTGG | 87548538 | 87548560 | 0.17 |
| GATCTGATGTTCTCTCTCCTC | 87548563 | 87548585 | 0.01 |
| GAGCAATCTGCAGAAGCCAG | 87548579 | 87548601 | 0.08 |
| GGCACGCATGTGCACACATG | 87548601 | 87548623 | 0.01 |
| GTGTGGTCTGTGTGAACACA | 87548622 | 87548644 | 0.03 |
| GCATCTCTATTTATGTACGTG | 87548639 | 87548661 | 1.76 |
| GCTTAAAGACATGAACGAGAC | 87548674 | 87548696 | 0.01 |
| GCATGAACGAGACTGGAGAAC | 87548682 | 87548704 | 1.39 |
| GCCAGACACTGCACTAGAGCT | 87548728 | 87548750 | 0.01 |
| GCAATATCACAGGGGTGGGAA | 87548760 | 87548782 | 0.36 |
| GCCACCTCAATATCACAGGGG | 87548766 | 87548788 | 0.77 |
| GTGATATTGAGGTGGGGGTG | 87548774 | 87548796 | 0.01 |
| GAGGGGTAGGGCATACAGTG | 87548800 | 87548822 | 0.00 |
| GTGAGGGTAGAATTAGCAAA | 87548817 | 87548839 | 0.21 |
| GCAAATGGAAGAAACCAAAA | 87548832 | 87548854 | 0.28 |
| GAAATGGAGGCTCTCTCGCCA | 87548849 | 87548871 | 0.09 |
| GAAGGCTGAGTTGTAGCATGC | 87548868 | 87548890 | 0.15 |
| GCATGCAGGACAACTTATAA | 87548882 | 87548904 | 0.03 |
| GACAACTTATAAAGGTTCAA | 87548890 | 87548912 | 0.01 |
| GATAAAGGTTCAAAGGCCTAG | 87548898 | 87548920 | 0.00 |
| GAGGTGCTCTGACAGCCTCT | 87548913 | 87548935 | 0.10 |
| GCTGTCAGAGCACCTCGCCTT | 87548921 | 87548943 | 1.78 |
| GAGGATGGCTCCCTAAGGCG | 87548932 | 87548954 | 1.17 |
| GCTAAGAGGATGGCTCCCTA | 87548937 | 87548959 | 0.01 |
| GCCATCCTCTTAGCTCCTGA | 87548946 | 87548968 | 0.86 |
| GCCATAGCAATGGTTCCTTC | 87548961 | 87548983 | 0.08 |
| GACCATTGCTATGGCCCTGAG | 87548970 | 87548992 | 0.04 |
| GCTATGGCCCTGAGTGGGC | 87548976 | 87548997 | 0.01 |
| GGCCCTGAGTGGGCTGGATG | 87548981 | 87549003 | 0.01 |

| | | | |
|---|---|---|---|
| GTGGGCTGGATGTGGGGACT | 87548989 | 87549011 | 0.64 |
| GTGGGGACTGGGCAGTGAA | 87549000 | 87549021 | 0.01 |
| GCTCTTGCTTACTCTGTACA | 87549023 | 87549045 | 0.01 |
| GTAGTGGTGGAAATCAAATTT | 87549047 | 87549069 | 0.05 |
| GTCCACCACTACATAGATAGA | 87549060 | 87549082 | 0.01 |
| GTAGATGGATAGGTAGATGAA | 87549123 | 87549145 | 0.02 |
| GGTGGATGGATAAAGTGTGG | 87549283 | 87549305 | 0.01 |
| GTATCAGCACTTGTGATGAAT | 87549320 | 87549342 | 0.09 |
| GGAAGGAGATCAGAAATTTA | 87549341 | 87549363 | 0.01 |
| GATTTGTCGTATAGCTGAGTC | 87549364 | 87549386 | 0.01 |
| GACAAATTTGAAGCCAGCCT | 87549380 | 87549402 | 0.01 |
| GCTTGCTTGTTTGTTTGAGA | 87549415 | 87549437 | 0.01 |
| GAACTAAATAGTACAGCTGTT | 87549467 | 87549489 | 4.10 |
| GAACCAGAGAATTACAGAGA | 87549503 | 87549525 | 1.13 |
| GAATTCTCTGGTTCAGTAAAA | 87549513 | 87549535 | 0.01 |
| GGTTCAGTAAAAAGGGCAAA | 87549521 | 87549543 | 0.01 |
| GTGTAGCATCAGAATTGTGA | 87549546 | 87549568 | 0.01 |
| GAATTGTGAAGGAGAGAGGT | 87549557 | 87549579 | 0.04 |
| GAGAGGTGGGACCTTCACTC | 87549570 | 87549592 | 1.41 |
| GGGCAGGAAACCCTGAGTGA | 87549581 | 87549603 | 0.02 |
| GAAATAGTTCTGTCACTGGGC | 87549597 | 87549619 | 5.20 |
| GAACTATTTTTAGGCTTGAT | 87549611 | 87549633 | 0.00 |
| GGATCTCTCATGCATGTTGC | 87549633 | 87549655 | 0.00 |
| GCATGTTGCAGGATGGTTTTC | 87549645 | 87549667 | 0.21 |
| GATGACAGTTTACTTGAGAG | 87549673 | 87549695 | 0.45 |
| GCTGTCATCTAATCCCAGCCC | 87549688 | 87549710 | 1.19 |
| GCCGGGTAACTGTCCTGGGCT | 87549700 | 87549722 | 0.01 |
| GTCTATCCGGGTAACTGTCCT | 87549705 | 87549727 | 0.01 |
| GCCAGGCTGCTACTCTATCC | 87549718 | 87549740 | 0.10 |
| GCAGCCTGGCCCCCACTACAC | 87549732 | 87549754 | 0.02 |
| GTCGAATAGCCAGTGTAGTGG | 87549740 | 87549762 | 0.10 |
| GCTTTGACCCAGCTATGTAAC | 87549768 | 87549790 | 0.19 |
| GTTGAAGGCCTGTTACATAGC | 87549775 | 87549797 | 0.01 |
| GCAGTCAGCGCAGGTCTTTGA | 87549791 | 87549813 | 0.01 |
| GACCTGCGCTGACTGGGTGT | 87549799 | 87549821 | 0.01 |
| GCTGACTGGGTGTTGGGAG | 87549806 | 87549827 | 0.01 |
| GAGCAGTCCTAGTCTATGAC | 87549827 | 87549849 | 1.05 |
| GAACTCTGCCTGTCATAGACT | 87549834 | 87549856 | 0.01 |
| GCAAATGCATGAACAGACGAC | 87549860 | 87549882 | 0.02 |
| GTGTGATTCAGCATGCACAC | 87549884 | 87549906 | 0.05 |
| GTCACAGATTGATGTTCTTGC | 87549909 | 87549931 | 0.10 |
| GTCTTGCTGGAGACTGAGCTC | 87549923 | 87549945 | 0.03 |
| GCTCAGGGCTTACGCACCTCT | 87549940 | 87549962 | 0.00 |
| GCCAGCAGTAAACTTGCCTAG | 87549955 | 87549977 | 0.02 |
| GATTACATGCTGAGGGACTAA | 87549987 | 87550009 | 0.01 |
| GATGCACAAAATTACATGCTG | 87549996 | 87550018 | 0.02 |
| GATATGAGCCTACTTCATGCT | 87550017 | 87550039 | 0.01 |
| GCTAAGTTCCTAGCATGAAGT | 87550024 | 87550046 | 0.29 |
| GAATGTTAAGCAAATACAAGC | 87550052 | 87550074 | 0.00 |
| GTCTAAGAGTCTTGATGGCAA | 87550076 | 87550098 | 0.21 |
| GATCAGCTCTAAGAGTCTTGA | 87550082 | 87550104 | 0.01 |
| GTCTTAGAGCTGATCTGGGCT | 87550092 | 87550114 | 0.01 |

| | | | |
|---|---|---|---|
| GCATTCTAGAGTTAGAAGAAT | 87550124 | 87550146 | 0.01 |
| GTGACAGCATAGAAGTCCA | 87550150 | 87550171 | 0.01 |
| GATAGAAGTCCAAGGCCACCA | 87550158 | 87550180 | 0.01 |
| GTGTAGGCCATGGTGGCCT | 87550166 | 87550187 | 0.19 |
| GTCTCACTGTGTAGGCCATGG | 87550172 | 87550194 | 0.04 |
| GTTTTTAGAGTCTCACTGTGT | 87550181 | 87550203 | 0.01 |
| GTGAGACTCTAAAAACAAAT | 87550189 | 87550211 | 0.02 |
| GACTCTAAAAACAAATGGGGG | 87550194 | 87550216 | 0.02 |
| GAAAACAAATGGGGGCGGGGA | 87550200 | 87550222 | 0.01 |
| GGGGCGGGGAAGGAAGGTAA | 87550210 | 87550232 | 0.05 |
| GAAGGAAGGTAAAGGAGGAA | 87550218 | 87550240 | 0.03 |
| GGTAAAGGAGGAAAGGGATG | 87550225 | 87550247 | 0.02 |
| GGAGGAAAGGGATGAGGAAA | 87550231 | 87550253 | 0.06 |
| GGATGAGGAAAGGGGAGAGG | 87550240 | 87550262 | 0.04 |
| GGGGAGAGGAGGGAAAGGAA | 87550251 | 87550273 | 0.01 |
| GAGGAGGGAAAGGAAGGGGT | 87550256 | 87550278 | 0.05 |
| GGAAGGGGTGGGAGGGGAAA | 87550267 | 87550289 | 0.02 |
| GGAAGGAAAGAGGGAAGGGG | 87550294 | 87550316 | 0.08 |
| GGGGAGGAAGGGGAGGGGAA | 87550310 | 87550332 | 0.03 |
| GGGGAAAGGGGAGGAGAGGA | 87550324 | 87550346 | 0.04 |
| GGGGAAAGGGGAGGTGAGGA | 87550344 | 87550366 | 0.12 |
| GAAAGGGGAGGTGAGGAGGGA | 87550348 | 87550370 | 0.03 |
| GAGGAGGGAAGGGAACAGGA | 87550359 | 87550381 | 0.01 |
| GGAAGGGAACAGGAGGGGAA | 87550365 | 87550387 | 0.06 |
| GGAACAGGAGGGGAAAGGGG | 87550370 | 87550392 | 0.07 |
| GAAAGGGGAGGGGAGGAGAGA | 87550383 | 87550405 | 0.12 |
| GGAGGAGAGAAGGGAAGGGG | 87550393 | 87550415 | 0.04 |
| GAGGGGAAGGAGGGAAGGGG | 87550421 | 87550443 | 0.05 |
| GGGAGGGGAGGGAGGGAAAT | 87550466 | 87550488 | 0.03 |
| GGGAGGGAAATAGGGAGGGG | 87550475 | 87550497 | 0.06 |
| GGGGAAAGGGGAGGGGAGCA | 87550496 | 87550518 | 0.02 |
| GGGAGGGGAGCAGGGAAGGG | 87550504 | 87550526 | 0.03 |
| GGAGAGAAGGGGAGGGGACA | 87550549 | 87550571 | 0.08 |
| GGAAGAAGCTTGCTCACTAC | 87550570 | 87550592 | 0.00 |
| GCTAGTGTAAAAGTGTTATAA | 87550629 | 87550651 | 0.02 |
| GTGTTATAAAGGGAAAGTGT | 87550640 | 87550662 | 0.20 |
| GGAAAGTGTGGGAAAGCAGC | 87550651 | 87550673 | 0.04 |
| GCTGAATCACATTCGAGTTGG | 87550684 | 87550706 | 0.01 |
| GAGTGGTCAGTGTCAGCTGG | 87550706 | 87550728 | 0.00 |
| GCTGGAGGGAAAACATTCT | 87550721 | 87550742 | 0.02 |
| GGAAGAAAAAAAAAGAGCTC | 87550741 | 87550763 | 0.02 |
| GAAAAAAGAGCTCTGGGGGGG | 87550749 | 87550771 | 0.53 |
| GTCTGGGGGGGAGGAGGTCTG | 87550759 | 87550781 | 0.08 |
| GGGGAGGAGGTCTGTGGCCA | 87550765 | 87550787 | 0.05 |
| GTTCTGCTGTATCAGCTTCCT | 87550782 | 87550804 | 0.06 |
| GTACAGCAGAACACACGCATA | 87550795 | 87550817 | 0.01 |
| GACACACGCATAAGGAAGTGT | 87550804 | 87550826 | 3.36 |
| GGGCTCAGGAGCAGAAAGTT | 87550831 | 87550853 | 0.15 |
| GTTTTCGTTTGCAGGGGCTC | 87550845 | 87550867 | 0.02 |
| GTTTGTTGTTTTCGTTTGCAG | 87550851 | 87550873 | 0.99 |
| GACAACAAAGGTCAGATTAAC | 87550866 | 87550888 | 0.08 |
| GCTACAAAAGCTTACACGAGA | 87550914 | 87550936 | 0.01 |

| | | | |
|---|---|---|---|
| GCCTGCTAGACAGCTCAGCC | 87550940 | 87550962 | 0.25 |
| GCTCAGCCAGGAAAAGCAC | 87550952 | 87550973 | 2.10 |
| GCTGCACTCCAGTGCTTTTCC | 87550958 | 87550980 | 7.21 |
| GCACTGGAGTGCAGACCAA | 87550967 | 87550988 | 0.71 |
| GGAGTGCAGACCAAAGGAC | 87550972 | 87550993 | 0.01 |
| GGAATTTGACTCCAGTCCTT | 87550982 | 87551004 | 0.28 |
| GCCCTAGCTCTCTGTAGGAT | 87551006 | 87551028 | 3.43 |
| GTCCTACAGAGAGCTAGGGCT | 87551010 | 87551032 | 0.02 |
| GAGCTAGGGCTAGGGAAGTA | 87551019 | 87551041 | 0.01 |
| GCATGAACAATGCATGCGTG | 87551063 | 87551085 | 0.01 |
| GTATGAAAGAAGCTGACTAAA | 87551377 | 87551399 | 0.02 |
| GTCTCAACACTTTAGAGATTG | 87551431 | 87551453 | 0.01 |
| GGAGAAGAGTGTTCAAGACC | 87551456 | 87551478 | 0.02 |
| GAGTGTTCAAGACCAGGCCA | 87551462 | 87551484 | 0.01 |
| GCTGGATTATCTCCCTGGCC | 87551474 | 87551496 | 0.02 |
| GTACTTGCTGGATTATCTCCC | 87551479 | 87551501 | 1.22 |
| GTTACAAGGGCTTTTACTTGC | 87551492 | 87551514 | 0.01 |
| GCTCATCGGGCTTGGTTACA | 87551507 | 87551529 | 0.02 |
| GAACTCAGCTCATCGGGCT | 87551515 | 87551536 | 0.07 |
| GAGTTTGAACTCAGCTCATC | 87551520 | 87551542 | 0.17 |
| GAGCTGAGTTCAAACTCTC | 87551526 | 87551547 | 0.01 |
| GTCAGGCCTACGAAGTAGAAG | 87551543 | 87551565 | 1.45 |
| GCAGAGCCTCTTCTACTTCGT | 87551548 | 87551570 | 0.01 |
| GATGGTATAATGTGAATCTTG | 87551587 | 87551609 | 0.22 |
| GTGGGTGTGTGTGGGAGGA | 87551606 | 87551627 | 0.00 |
| GCATGCATGTGGGTGTGTGT | 87551613 | 87551635 | 0.06 |
| GACATTTGCATGCATGCATGT | 87551623 | 87551645 | 0.01 |
| GATTAAATAACAACATTCTAA | 87551681 | 87551703 | 0.01 |
| GTAACAACATTCTAAAGGGCC | 87551687 | 87551709 | 0.01 |
| GATTCTAAAGGGCCTGGAGAG | 87551694 | 87551716 | 0.01 |
| GACTACGGAGCCCTCTCTCC | 87551705 | 87551727 | 0.01 |
| GCAGTAGAGCTCTTGACTA | 87551720 | 87551741 | 1.81 |
| GAAGAGCTCTACTGCTTTTGC | 87551728 | 87551750 | 0.01 |
| GTACTGCTTTTGCAGGGAACC | 87551736 | 87551758 | 0.00 |
| GTAATTGTGGCAACAAAGCCC | 87551754 | 87551776 | 0.01 |
| GTTGTTGCCACAATTATATGA | 87551762 | 87551784 | 0.00 |
| GTAAGCCATCATATAATTG | 87551768 | 87551789 | 0.28 |
| GCAGAGTCAGAGCCCCAGAAC | 87551804 | 87551826 | 0.04 |
| GACTCTGTCTTCTGATTTCTC | 87551821 | 87551843 | 0.01 |
| GGGTCTGCATGAGCATGCA | 87551847 | 87551868 | 0.00 |
| GTTTTATTTTTATTTGTGTGA | 87551866 | 87551888 | 0.04 |
| GATTAATGTCTTTTTTTAAAA | 87551888 | 87551910 | 0.03 |
| GTTTTAAAACGGCAGCTATCT | 87551900 | 87551922 | 0.04 |
| GGCAGCTATCTTGGCAGTGG | 87551909 | 87551931 | 0.00 |
| GTCAATCTGTCTCTGCCTCCT | 87551953 | 87551975 | 0.39 |
| GATTGATCTTATGAGTTTG | 87551976 | 87551997 | 0.00 |
| GCCAGCCCGGTCTACAGAGC | 87551997 | 87552019 | 0.01 |
| GGTCTACAGAGCAGGTTCCA | 87552005 | 87552027 | 0.03 |
| GCAGGGCTACACAGTAAAACT | 87552023 | 87552045 | 0.02 |
| GTAAAATATCCTTCGTTTTGA | 87552074 | 87552096 | 0.00 |
| GTAATAAAGCCTTCAAAACGA | 87552082 | 87552104 | 0.01 |
| GGCAAGTTCTAGAATAGTCA | 87552110 | 87552132 | 0.03 |

| | | | |
|---|---|---|---|
| GAACTTGCCTAAGCAGACT | 87552124 | 87552145 | 0.01 |
| GTACATAGCCTAGTCTGCTT | 87552131 | 87552153 | 0.95 |
| GTGTACGAGGCTGAGGCACA | 87552170 | 87552192 | 0.01 |
| GCCTCAGCCTCGTACACGCT | 87552176 | 87552198 | 0.19 |
| GCTTTATTCCCAGCGTGTACG | 87552183 | 87552205 | 0.01 |
| GACATGCTTTTAAGCTGGGTG | 87552216 | 87552238 | 0.01 |
| GCAAAAACATGCTTTTAAGCT | 87552221 | 87552243 | 0.01 |
| GAAAAGAATTTGAGTTCAGCC | 87552249 | 87552271 | 0.27 |
| GAGTTCAGCCTGGGCTACA | 87552259 | 87552280 | 0.03 |
| GGTAAGATACTGTTTAAAAG | 87552279 | 87552301 | 0.01 |
| GATACTGTTTAAAAGGGGTG | 87552284 | 87552306 | 0.56 |
| GTTTAAAAGGGGTGGGGGTG | 87552290 | 87552312 | 0.04 |
| GGTGGGACGTGTGCTGCTAC | 87552311 | 87552333 | 0.00 |
| GCTGCTACTGGTTAAAAACAC | 87552324 | 87552346 | 0.01 |
| GAGTATGTCATGTGAGTGCT | 87552376 | 87552398 | 0.02 |
| GCTCAGTTTCTTAGAATGTGA | 87552396 | 87552418 | 0.03 |
| GATGGCCCCTGCTAGCACC | 87552414 | 87552435 | 0.24 |
| GCCTATGCCTGGTGCTAGCA | 87552420 | 87552442 | 0.83 |
| GCTGTGCGCCATGCCTATGCC | 87552431 | 87552453 | 0.00 |
| GGCGCACAGACACATGTGC | 87552445 | 87552466 | 0.03 |
| GTAATTAATTATTGATGTGTT | 87552475 | 87552497 | 0.02 |
| GTTAATTATAATTTTAAAAGT | 87552491 | 87552513 | 0.01 |
| GAATTGTGCACCCAATGTCCC | 87552516 | 87552538 | 0.09 |
| GCTCTCCTTCCCTGGGACATT | 87552525 | 87552547 | 0.01 |
| GGCATGCCCTCTCCTTCCCT | 87552533 | 87552555 | 3.51 |
| GGAAGGAGAGGGCATGCCAG | 87552538 | 87552560 | 0.01 |
| GGCAACACAAACGTTCCAC | 87552554 | 87552575 | 1.35 |
| GCCTGGAAATTCATTTAATGG | 87552574 | 87552596 | 0.01 |
| GTTATCTCACACTCATTTCCC | 87552592 | 87552614 | 0.01 |
| GATTGATACTCCTGCATGTTT | 87552626 | 87552648 | 0.01 |
| GATGCTAACCCTAAACATGC | 87552635 | 87552657 | 0.01 |
| GTTTAGGGTTAGCATCTTTA | 87552642 | 87552664 | 0.01 |
| GAAGAAGCTTGGAAAATGC | 87552685 | 87552706 | 0.01 |
| GATGGCTACTGAAGAAGCT | 87552695 | 87552716 | 7.22 |
| GCCATCAAACTCCCTGTAGA | 87552711 | 87552733 | 0.01 |
| GTCTATGTAAATCCCTCTACA | 87552722 | 87552744 | 0.69 |
| GATTTACATAGATTTACTCT | 87552733 | 87552755 | 0.03 |
| GTCAGGGAAGCTTTTACTGTC | 87552763 | 87552785 | 0.01 |
| GCAGTAAAAGCTTCCCTGAAG | 87552768 | 87552790 | 0.02 |
| GTGTTCACGAACTCCTCTTC | 87552781 | 87552803 | 0.00 |
| GCAGACACATCTGGGGATTG | 87552803 | 87552825 | 0.28 |
| GACTGAAAGCAGACACATCT | 87552811 | 87552833 | 0.01 |
| GTCAGTCAGATGAGAGAAACT | 87552828 | 87552850 | 0.01 |
| GAAGGCTTTGTGTACACCTG | 87552852 | 87552874 | 0.01 |
| GTGGATACTGGGGTTCCAC | 87552868 | 87552889 | 0.01 |
| GATTCTAAGCTGTGGATACTG | 87552877 | 87552899 | 1.74 |
| GTAGTCTACTATTCTAAGCTG | 87552886 | 87552908 | 0.24 |
| GTTAGAATAGTAGACTAACTC | 87552893 | 87552915 | 1.16 |
| GAACTCTGGGGTCCCAGCAAG | 87552908 | 87552930 | 0.01 |
| GCCAGTGGAGACCCTCTTGC | 87552920 | 87552942 | 0.00 |
| GAGGGTCTCCACTGGCGCGG | 87552927 | 87552949 | 0.00 |
| GTCTCCACTGGCGCGGAGGAC | 87552932 | 87552954 | 0.01 |

| | | | |
|---|---|---|---|
| GGCGCGGAGGACAGGGGACG | 87552940 | 87552962 | 0.01 |
| GACAGGGGACGGGGTGGGAG | 87552949 | 87552971 | 0.02 |
| GGAGTGGGGGGACCCACCAC | 87552965 | 87552987 | 0.01 |
| GACCCACCACAGGGTTCAGT | 87552975 | 87552997 | 0.02 |
| GATGAAGCCAACTGAACCCTG | 87552981 | 87553003 | 1.33 |
| GTTGGCTTCATCCCTTTCCT | 87552993 | 87553015 | 0.00 |
| GCTTCATCCCTTTCCTGGGGA | 87552998 | 87553020 | 0.01 |
| GATTCTTCCATCCCCAGGAAA | 87553004 | 87553026 | 9.47 |
| GTCTTTCATTCTTCCATCCCC | 87553010 | 87553032 | 0.08 |
| GAAAGAGCTGAAGTTTTAAGC | 87553028 | 87553050 | 0.17 |
| GTTTTAAGCAGGAGAGCTGC | 87553039 | 87553061 | 0.01 |
| GCTACTTGTGTTCGTTAAGCT | 87553065 | 87553087 | 0.00 |
| GTCGTTAAGCTCGGGGACTCC | 87553075 | 87553097 | 0.00 |
| GAAGCTCGGGGACTCCAGGAC | 87553080 | 87553102 | 0.04 |
| GCCTGTGTTCCGTCCAGTCC | 87553093 | 87553115 | 0.12 |
| GGCAGCCAACTACCCCAGCC | 87553113 | 87553135 | 0.04 |
| GAGTTCCAGGCTGGGGTAGT | 87553118 | 87553140 | 0.13 |
| GTACCCCAGCCTGGAACTCAG | 87553123 | 87553145 | 0.02 |
| GCGCTCTCCTCTGAGTTCC | 87553131 | 87553152 | 0.01 |
| GAGCGCTGACGATTACTGT | 87553147 | 87553168 | 0.83 |
| GCTGTAGGAGTCAGCCACGTA | 87553162 | 87553184 | 0.89 |
| GCTATAGTTACCAGCCATACG | 87553175 | 87553197 | 0.01 |
| GTTCAAATCTCATGCACACAC | 87553199 | 87553221 | 0.06 |
| GAGATTTGAAAGAAAAGATA | 87553212 | 87553234 | 0.11 |
| GTAAATCTATAGGTGGGTAGA | 87553251 | 87553273 | 0.01 |
| GTTTCTTTTAAATCTATAGGT | 87553258 | 87553280 | 0.48 |
| GCAGAAAGCAAGAGCGCTG | 87553295 | 87553316 | 0.14 |
| GCGCTGTGGAGACAGAAGGT | 87553308 | 87553330 | 0.01 |
| GAGACAGAAGGTGGGCTTGC | 87553316 | 87553338 | 0.21 |
| GGTGGGCTTGCAGGAGCCCC | 87553325 | 87553347 | 0.01 |
| GTTGCAGGAGCCCCTGGCACT | 87553332 | 87553354 | 0.08 |
| GCTGCAAACACCAAGTGCCAG | 87553341 | 87553363 | 0.01 |
| GTTGGTGTTTGCAGGAGCCCC | 87553351 | 87553373 | 0.01 |
| GTTGCAGGAGCCCCCGGCACT | 87553358 | 87553380 | 0.70 |
| GATGCAAACACCAAGTGCCGG | 87553367 | 87553389 | 0.03 |
| GCACTTGGTGTTTGCATTTGC | 87553374 | 87553396 | 0.12 |
| GGTGTTTGCATTTGCAGGAA | 87553379 | 87553401 | 0.46 |
| GGAAGGGTCCATGTGAGCG | 87553395 | 87553416 | 1.70 |
| GCAACGTTCCGCGCTCACA | 87553403 | 87553424 | 0.01 |
| GCTGTACAGTGCAAGACTGCG | 87553433 | 87553455 | 0.02 |
| GCACTGTACAGGATCCCCG | 87553445 | 87553466 | 2.59 |
| GATCTGAGCTCGGATCCTCG | 87553459 | 87553481 | 0.00 |
| GCAAACTGAGGATCTGAGCT | 87553469 | 87553491 | 0.01 |
| GTCAGGCGAGAAGGCAAACTG | 87553481 | 87553503 | 0.01 |
| GTTTAAATCCATCAGGCGAGA | 87553491 | 87553513 | 0.01 |
| GAGGATTTTTAAATCCATC | 87553499 | 87553520 | 0.50 |
| GTTTAAAAATCCTCATCTCCC | 87553508 | 87553530 | 0.00 |
| GATCCTCATCTCCCTGGGCAG | 87553515 | 87553537 | 0.52 |
| GCGTGCGCAGCCACTGCCCA | 87553525 | 87553547 | 0.98 |
| GATGAGGATTTGTTGTTGTTT | 87553674 | 87553696 | 0.01 |
| GATAATGCAATGTGTGACATG | 87553691 | 87553713 | 0.01 |
| GTATAGTTTATGGCAACATAT | 87553723 | 87553745 | 0.01 |

| | | | |
|---|---|---|---|
| GTAACAGTAATATATAGTTTA | 87553734 | 87553756 | 0.58 |
| GTATTGAAGACGATGTAGTCC | 87553761 | 87553783 | 0.07 |
| GATGTAGTCCCGGCTAGCC | 87553771 | 87553792 | 0.01 |
| GTAGTAAGTTCCTGGCTAGCC | 87553779 | 87553801 | 0.01 |
| GATAGAGATAAGAAGAGGTCC | 87553817 | 87553839 | 1.03 |
| GGGACATAGAGATAAGAAG | 87553823 | 87553844 | 0.04 |
| GTCTTATCTCTATGTCCCTAG | 87553828 | 87553850 | 0.02 |
| GTCCCTAGTGGTGGCATTAA | 87553840 | 87553862 | 0.02 |
| GTGGTGGCATTAAAGGTGT | 87553847 | 87553868 | 0.02 |
| GGTGTGGGCCACATTTTTAT | 87553861 | 87553883 | 1.36 |
| GCAAACCCTCCTATAAAAATG | 87553869 | 87553891 | 0.01 |
| GAAGGGTCACAACACTGGGAA | 87553931 | 87553953 | 0.23 |
| GCATGAGGGACTGTATCAAA | 87553949 | 87553971 | 0.05 |
| GATACAGTCCCTCATGCTG | 87553955 | 87553976 | 0.01 |
| GTTGGCGTCGCCGCAGCATG | 87553964 | 87553986 | 0.01 |
| GCAGCAATGAAAATAATGTTG | 87553986 | 87554008 | 0.21 |
| GAATCGTAATGTAAACATTT | 87554038 | 87554060 | 0.03 |
| GTAAACATTTTGGAGAGAG | 87554048 | 87554069 | 2.04 |
| GTTTGCCAAAGGAGACCCAC | 87554069 | 87554091 | 0.26 |
| GCTTAACCTGTGGGTCTCCTT | 87554074 | 87554096 | 0.02 |
| GAGCAATGCTTCTTAACCTG | 87554085 | 87554107 | 0.02 |
| GGTTAAGAAGCATTGCTCTA | 87554090 | 87554112 | 0.03 |
| GGATTGTGTCTAAGTGTGTG | 87554112 | 87554134 | 0.00 |
| GACGTATTGTGTGCACATGTG | 87554136 | 87554158 | 0.24 |
| GACGTGTGCTCTCAGGGTCAC | 87554163 | 87554185 | 0.01 |
| GGCTTCTACGTGTGCTCTCA | 87554170 | 87554192 | 3.17 |
| GCACACGTAGAAGCCAGGGT | 87554178 | 87554200 | 0.01 |
| GAGCACCTGGTGTCCCACCC | 87554191 | 87554213 | 0.04 |
| GGAGGGCAGAAGAGAGCACC | 87554204 | 87554226 | 0.08 |
| GGAACCGTGACTAAGGCAGC | 87554225 | 87554247 | 0.02 |
| GCTTAGTAGGAACCGTGACTA | 87554232 | 87554254 | 0.05 |
| GACGGTTCCTACTAAGACAGA | 87554240 | 87554262 | 0.01 |
| GCAGAAACCTTCTGTCTTAGT | 87554246 | 87554268 | 0.02 |
| GTTTCTGAGCTCTTAAAGAA | 87554262 | 87554284 | 0.05 |
| GCTTAAAGAAAGGGTCACTAA | 87554273 | 87554295 | 0.01 |
| GTAAAGGAGTCTAAGCATCGA | 87554290 | 87554312 | 0.38 |
| GATGGAATCCCATCAATAA | 87554308 | 87554329 | 2.89 |
| GTTTTGATGCCTTTATTGAT | 87554316 | 87554338 | 0.00 |
| GTAAAGGCATCAAAACAAAGA | 87554324 | 87554346 | 0.01 |
| GCATCAAAACAAAGATGGGG | 87554329 | 87554351 | 0.02 |
| GAAACAAAGATGGGGAGGTTC | 87554335 | 87554357 | 0.02 |
| GATGGGGAGGTTCTGGGCAG | 87554342 | 87554364 | 0.05 |
| GCAGGGGTGTCTGCTAGACAC | 87554359 | 87554381 | 0.01 |
| GACACTGGTTAGACAGATGTG | 87554375 | 87554397 | 0.01 |
| GACAGATGTGTGGGGATGA | 87554385 | 87554406 | 0.01 |
| GATGTGTGGGGATGATGGTCA | 87554390 | 87554412 | 0.03 |
| GATGGTCAAGGCATCCATGA | 87554402 | 87554424 | 0.04 |
| GCAAGGCATCCATGATGGATG | 87554408 | 87554430 | 0.19 |
| GACAGAACCACATCCATCA | 87554416 | 87554437 | 0.01 |
| GTGGTTCTGTCAAGCCCAC | 87554427 | 87554448 | 0.79 |
| GTCACTTCACAGCAGACCAG | 87554442 | 87554464 | 0.01 |
| GTTTTTGCCCTAGTGTTTATC | 87554469 | 87554491 | 1.06 |

| | | | |
|---|---|---|---|
| GGAAACCCAGATAAACACTA | 87554475 | 87554497 | 1.31 |
| GGCTTCTCTGTAGCACACA | 87554496 | 87554517 | 0.01 |
| GTCTGGAGTCTGACAGGGATT | 87554516 | 87554538 | 0.03 |
| GCCTGGGTCTGGAGTCTGAC | 87554523 | 87554545 | 0.01 |
| GACTCCAGACCCAGGCCTGGG | 87554531 | 87554553 | 1.20 |
| GTAAGCATTCCACCCAGGCCT | 87554539 | 87554561 | 0.20 |
| GGAGAATAAGCATTCCACCC | 87554545 | 87554567 | 0.81 |
| GGGAGAGTAAAAGTAGGGGG | 87554566 | 87554588 | 0.89 |
| GTTCCCAGGGAGAGTAAAAGT | 87554572 | 87554594 | 0.02 |
| GTCTTCTGGTCACATTTCCCA | 87554586 | 87554608 | 0.01 |
| GATGTGACCAGAAGAAAGACC | 87554595 | 87554617 | 0.02 |
| GCTTTCACCTGGTCTTTCTTC | 87554601 | 87554623 | 0.01 |
| GAAGACCAGGTGAAAGGCCAA | 87554609 | 87554631 | 1.02 |
| GCTTGTTAGTTCTTGGCCTT | 87554625 | 87554647 | 0.01 |
| GCCTGAGAGCTTGTTAGTTCT | 87554632 | 87554654 | 0.01 |
| GAATATAAACATCAAGCATGA | 87554661 | 87554683 | 0.01 |
| GAATGGGCTTAGTGTTCAGTA | 87554695 | 87554717 | 0.47 |
| GTAAGCCCATTAGCAGTAAAA | 87554708 | 87554730 | 0.02 |
| GATGGGCCGTTTTACTGCTAA | 87554713 | 87554735 | 0.01 |
| GCAGCTCTGGGACAATCACAA | 87554732 | 87554754 | 0.02 |
| GATTGTCCCAGAGCTGGGCAG | 87554740 | 87554762 | 0.01 |
| GCATGCCACTGCCCAGCTCT | 87554745 | 87554767 | 0.00 |
| GGCTCAAAGCTGGTCTAAT | 87554771 | 87554792 | 0.01 |
| GGCTTAAGTTGGCTCAAAGC | 87554780 | 87554802 | 17.69 |
| GCATTATTCAGGGGCTTAAGT | 87554791 | 87554813 | 0.14 |
| GCCCCTGAATAATGCCCACT | 87554800 | 87554822 | 0.02 |
| GAATGCCCACTGGGATGCCGC | 87554810 | 87554832 | 0.20 |
| GGACAAGAAAATAACCCCTG | 87554826 | 87554848 | 0.01 |
| GATTTTCTTGTCCTCTGTGTC | 87554837 | 87554859 | 0.01 |
| GTCCTCTGTGTCAGGTAGGT | 87554845 | 87554867 | 0.01 |
| GTGTCAGGTAGGTTGGTCC | 87554852 | 87554873 | 0.30 |
| GAAACATTTTCACAGTGTTCC | 87554869 | 87554891 | 0.01 |
| GAAAATGTTTGTCATTATTC | 87554882 | 87554904 | 0.02 |
| GAAACTGTAAACTTTTCTC | 87554926 | 87554947 | 0.02 |
| GCTTTTCTCAGGACTCAGAAG | 87554937 | 87554959 | 0.33 |
| GAAGTGGGTACTAGAGTGTCA | 87554954 | 87554976 | 1.49 |
| GGTACTAGAGTGTCAAGGGC | 87554959 | 87554981 | 0.02 |
| GAGTGTCAAGGGCTGGAGCA | 87554966 | 87554988 | 0.02 |
| GAGTGACCTCAGCTCCCTTC | 87555002 | 87555024 | 0.04 |
| GTCAGCTCCCTTCAGGCCTTC | 87555010 | 87555032 | 0.12 |
| GCAGGTGGCCAGAAGGCCTGA | 87555017 | 87555039 | 1.20 |
| GGAGGGAGCAGGTGGCCAGA | 87555025 | 87555047 | 0.01 |
| GATCAGAAGGGAGGGAGCAGG | 87555033 | 87555055 | 0.01 |
| GCTAGGCAGCATCAGAAGGGA | 87555042 | 87555064 | 0.00 |
| GATGCTGCCTAGAAGGAAA | 87555053 | 87555074 | 0.02 |
| GTCTCATGACCTTTTCCTTCT | 87555060 | 87555082 | 0.01 |
| GGTCATGAGATGTCCATTG | 87555073 | 87555094 | 0.09 |
| GATGTCCATTGTGGAGCCT | 87555081 | 87555102 | 0.00 |
| GACTGTTCCTAGGCTCCACAA | 87555086 | 87555108 | 0.41 |
| GGAAATTTTGACTGTTCCT | 87555097 | 87555118 | 0.01 |
| GACAGTCAAAATTTCCAAGAC | 87555104 | 87555126 | 0.00 |
| GCCTGGTGAATAACCCAGTCT | 87555117 | 87555139 | 0.02 |

| | | | |
|---|---|---|---|
| GCTTGTCATGGAGGGTTTGCC | 87555135 | 87555157 | 0.02 |
| GACCCTCCATGACAAGGTAGA | 87555143 | 87555165 | 0.01 |
| GACAAGGTAGATGGGAAGGT | 87555152 | 87555174 | 0.03 |
| GAAGAGCATCTGTGAGGTTCT | 87555177 | 87555199 | 0.01 |
| GTGAGCTAAGAGCATCTGTG | 87555184 | 87555206 | 0.02 |
| GGCCTGATGGCTGAAAACTG | 87555213 | 87555235 | 0.02 |
| GTTAGGTGTGCACAGGCCTGA | 87555226 | 87555248 | 0.02 |
| GGCCTGTGCACACCTAACAC | 87555232 | 87555254 | 1.03 |
| GCACCTAACACAGGGTGCTCA | 87555242 | 87555264 | 0.01 |
| GAGGGTTTTGGAGTGTTGGA | 87555268 | 87555290 | 0.06 |
| GCTAAAGGAGGCAGAGGGTTT | 87555280 | 87555302 | 0.01 |
| GTGCAGGCTAAAGGAGGCAG | 87555287 | 87555309 | 0.10 |
| GTCCTTTAGCCTGCACTGCAC | 87555295 | 87555317 | 0.01 |
| GTTCGCAGCCCAGTGCAGTGC | 87555303 | 87555325 | 0.02 |
| GCACTGGGCTGCGAAGTTCG | 87555311 | 87555333 | 0.01 |
| GGGATGCTTGCCTAGCATGT | 87555332 | 87555354 | 0.00 |
| GGCTCTGGGCATCAGATCCA | 87555358 | 87555380 | 0.02 |
| GCAGCTAGTTTTTGTGGCTCT | 87555372 | 87555394 | 0.01 |
| GCTGCCACAGCTAGTTTTTG | 87555379 | 87555401 | 0.00 |
| GCTGCAATCCCAGCAGCTCTC | 87555407 | 87555429 | 0.01 |
| GCTTGCCTCCTGAGAGCTGCT | 87555414 | 87555436 | 0.01 |
| GTCTCAGGAGGCAAGAGCAGC | 87555423 | 87555445 | 0.00 |
| GTCAGTAGTTCAAGAACAACC | 87555450 | 87555472 | 0.01 |
| GGGCTACATAGCAAGATAC | 87555471 | 87555492 | 0.01 |
| GATACTGGAAAAAGAGAGAG | 87555485 | 87555507 | 0.02 |
| GGGGGGAGAGAGGAAGGGGG | 87555560 | 87555582 | 0.10 |
| GAGAGAGATCCTAATGAGTA | 87555603 | 87555625 | 0.01 |
| GATTTCCCTCCCATACTCATT | 87555612 | 87555634 | 0.06 |
| GAATCTTTTTAAATAATGTGA | 87555672 | 87555694 | 0.02 |
| GCACATGTAACGTAATTCTGC | 87555703 | 87555725 | 0.01 |
| GTCGGGCGCTGGTAGAGGGC | 87555728 | 87555750 | 0.01 |
| GCAGGAGTCGGGCGCTGGTAG | 87555733 | 87555755 | 0.43 |
| GCACGCAGGAGTCGGGCGC | 87555739 | 87555760 | 0.01 |
| GCAAGCAGCACGCAGGAGTC | 87555745 | 87555767 | 0.24 |
| GCTCGAGTGCAAGCAGCACGC | 87555752 | 87555774 | 0.01 |
| GCTTCTGCTATTGGCTTCT | 87555787 | 87555808 | 0.19 |
| GTAAGCTGTTGCTTCTGCTAT | 87555795 | 87555817 | 0.07 |
| GCCTCAACCTTCTAGAGCAG | 87555818 | 87555840 | 0.01 |
| GTACTAAAGGTCTCAGGCTT | 87555852 | 87555874 | 0.01 |
| GGAACTGTACTAAAGGTCTC | 87555858 | 87555880 | 0.02 |
| GCAATAGTTTTGTGTTTGGG | 87555898 | 87555920 | 0.04 |
| GAATGCAATAGTTTTGTGTTT | 87555901 | 87555923 | 0.01 |
| GCATAGCAGAGGCAAAATTAC | 87555935 | 87555957 | 0.01 |
| GTTACATCTGATTCATAGCAG | 87555947 | 87555969 | 0.01 |
| GATGTCTGATATACGATCCAC | 87555986 | 87556008 | 0.11 |
| GTTCTAGAAGCTACCCCTCC | 87556021 | 87556043 | 0.01 |
| GAAGCTACCCCTCCTGGACT | 87556027 | 87556049 | 3.82 |
| GAGAAAGTCCCAGTCCAGGA | 87556035 | 87556057 | 0.01 |
| GCTGGGACTTTCTCTCCCCAG | 87556045 | 87556067 | 0.09 |
| GCCCAGTAAAGATTCCACTG | 87556059 | 87556081 | 0.02 |
| GGCTGGACAATAGTAGGGTG | 87556081 | 87556103 | 0.01 |
| GAAGTCAGGCTGGACAATAGT | 87556087 | 87556109 | 0.01 |

| | | | |
|---|---|---|---|
| GAACTTCTGGCTTTCAAGTC | 87556102 | 87556124 | 0.01 |
| GCACAATTTACTCTGAACTTC | 87556115 | 87556137 | 0.02 |
| GTTGATCCAGTTCATAGCAG | 87556146 | 87556168 | 0.14 |
| GTGTCCCACTGCTATGAAC | 87556152 | 87556173 | 1.98 |
| GCATTTAGGATGAGTTCAGGG | 87556179 | 87556201 | 0.02 |
| GTCCTAAATGTAATCATGATT | 87556193 | 87556215 | 0.26 |
| GATTTGGTTCCAATCTGAATC | 87556210 | 87556232 | 0.01 |
| GTCTGAATCTGGTTCTATCTG | 87556222 | 87556244 | 0.60 |
| GATCTGGTTCTATCTGAGGAC | 87556227 | 87556249 | 0.01 |
| GTCAGGCACACGCAGGCTGTT | 87556257 | 87556279 | 0.01 |
| GATGATGTCAGGCACACGC | 87556265 | 87556286 | 0.48 |
| GGATACTTACTGATGATGTC | 87556275 | 87556297 | 0.01 |
| GGCTGCGGAGGAGGTGGCC | 87556296 | 87556317 | 0.01 |
| GGTCTAGCAGGCTGCGGAGG | 87556304 | 87556326 | 0.09 |
| GATTGGTCTAGCAGGCTGCGG | 87556307 | 87556329 | 0.96 |
| GACAGTAACATTGGTCTAGC | 87556316 | 87556338 | 0.01 |
| GTAGTGTTCCGACAGTAACAT | 87556325 | 87556347 | 0.02 |
| GACACTACCATGTGAACACAT | 87556342 | 87556364 | 0.01 |
| GATGCGACCTATGTGTTCACA | 87556348 | 87556370 | 0.09 |
| GTCGCATTCAACTTTCTAGA | 87556364 | 87556386 | 0.03 |
| GCACATGTGAGAAAAGAAAA | 87556386 | 87556408 | 0.04 |
| GAGAAAAGAAAATGGAGGCC | 87556394 | 87556416 | 0.03 |
| GAAGAAAATGGAGGCCGGGCA | 87556399 | 87556421 | 0.01 |
| GAAGGAAGGAAGGAAATGGGC | 87556641 | 87556663 | 0.47 |
| GGAAGGAAATGGGCTGGAG | 87556647 | 87556668 | 0.01 |
| GTCTTCCTCCTGTGTGTGTG | 87556687 | 87556709 | 0.05 |
| GTCCCAGCCCCACACACAC | 87556695 | 87556716 | 0.13 |
| GCAAATCTCAGCTCATATGTC | 87556718 | 87556740 | 0.00 |
| GCAGCTCATATGTCAGGTGAC | 87556725 | 87556747 | 0.01 |
| GTGACTGGAAATCCAGCTCC | 87556740 | 87556762 | 0.19 |
| GAAGGACGGATCTCCTGGAGC | 87556752 | 87556774 | 0.02 |
| GACAACAAAGGACGGATCTCC | 87556758 | 87556780 | 0.00 |
| GCAGGAGCGAACAACAAAGGA | 87556767 | 87556789 | 2.53 |
| GTTGTTCGCTCCTGGCGTCCA | 87556777 | 87556799 | 0.05 |
| GTACAGTGCCCGTGGACGCC | 87556786 | 87556808 | 0.02 |
| GCACATGTGTACAGTGCCCG | 87556794 | 87556816 | 0.02 |
| GCACTGTACACATGTGCTGTG | 87556801 | 87556823 | 0.00 |
| GGTTTGAATCTGCTTGGCAA | 87556825 | 87556847 | 0.42 |
| GAATCTGCTTGGCAAGGGAG | 87556830 | 87556852 | 0.01 |
| GGCAAGGGAGTGGCACTACT | 87556840 | 87556862 | 0.06 |
| GTACTAGGAGATGTGGCCTTG | 87556856 | 87556878 | 0.01 |
| GATGTGGCCTTGTGGGAGGTG | 87556865 | 87556887 | 0.02 |
| GTCAAGGCCACACCTCCCACA | 87556871 | 87556893 | 0.04 |
| GCAATGGGAGCTTTTTCCTAG | 87556920 | 87556942 | 0.36 |
| GGAGAAGACTGGCATCCTCT | 87556935 | 87556957 | 0.54 |
| GTAAAGCAGTCAGGAGAAGAC | 87556946 | 87556968 | 0.02 |
| GATCTTCATCTTAAAGCAGTC | 87556956 | 87556978 | 0.02 |
| GACAGTCATGGTATTGGAGA | 87556994 | 87557016 | 0.00 |
| GGCATGACAGTCATGGTAT | 87557000 | 87557021 | 0.01 |
| GGTGGGAGGCATGACAGTCA | 87557006 | 87557028 | 0.20 |
| GGTTCCGTCCATTATCACCA | 87557027 | 87557049 | 0.08 |
| GATTAATTGGGTCTGGCTTCC | 87557056 | 87557078 | 0.01 |

| | | | |
|---|---|---|---|
| GTGTGTGGTGTGTCTTAGTT | 87557134 | 87557156 | 0.01 |
| GATGATTGCAGTGCATGTGTG | 87557149 | 87557171 | 0.00 |
| GCTTGAGCATGTACACACAG | 87557172 | 87557194 | 0.01 |
| GAAATAAATAAAAATCGTCT | 87557212 | 87557234 | 0.06 |
| GATCGTCTTGGAAAAGACAAA | 87557225 | 87557247 | 0.02 |
| GAAAAGGTGAACACGTGAGTA | 87557242 | 87557264 | 0.01 |
| GACGTCTGTGCACGTATGTGC | 87557266 | 87557288 | 0.22 |
| GATGCTTTCCTGATGTGTGTG | 87557290 | 87557312 | 0.00 |
| GCACTGCACCACACACACATC | 87557297 | 87557319 | 0.01 |
| GTGTGTGTGGTGCAGTGGC | 87557303 | 87557324 | 0.00 |
| GGCAGAGAGTGATAGGGGA | 87557327 | 87557348 | 0.01 |
| GTTTTATTTTATTTTCAAGT | 87557351 | 87557373 | 0.05 |
| GATTTTCAAGTAGGATCTCTG | 87557361 | 87557383 | 0.39 |
| GTAGGATCTCTGAGGGACCC | 87557369 | 87557391 | 0.01 |
| GACCCTGGAGTTCACTGGTC | 87557384 | 87557406 | 0.12 |
| GGCCCTGACCAGTGAACTCC | 87557387 | 87557409 | 0.11 |
| GTTCACTGGTCAGGGCCGC | 87557393 | 87557414 | 0.00 |
| GGTCAGGGCCGCTGGCCTCC | 87557400 | 87557422 | 0.02 |
| GGGCCGCTGGCCTCCAGGAT | 87557405 | 87557427 | 2.38 |
| GAGGAGACAGCCAATCCTGG | 87557415 | 87557437 | 0.30 |
| GTCTCCTCTCTATCAGTGCT | 87557430 | 87557452 | 0.02 |
| GACAAAGAACCCCACCACAGC | 87557456 | 87557478 | 0.14 |
| GACGTGAAAGCCAGCTGTGGT | 87557465 | 87557487 | 0.01 |
| GGGTACTACAGATCTGAATT | 87557488 | 87557510 | 0.01 |
| GATCTGAATTCGGGTACTT | 87557498 | 87557519 | 0.04 |
| GCAGTAGGTAAAATGCTTGCT | 87557524 | 87557546 | 0.01 |
| GCACTGGGGAGGTAGCTCAGT | 87557540 | 87557562 | 0.10 |
| GATTAATTTTGATCACTGGGG | 87557552 | 87557574 | 0.24 |
| GATGCAATATTTTGACATTT | 87557594 | 87557616 | 0.01 |
| GTTGCTGCTGCTGCTCCTT | 87557651 | 87557672 | 0.01 |
| GCTGCTCCTTTGGTTTTCTG | 87557660 | 87557682 | 1.05 |
| GATCGCCTCAGAAAACCAA | 87557666 | 87557687 | 0.01 |
| GTTTTCTGAGGCGATCTCACG | 87557673 | 87557695 | 0.01 |
| GAGGCGATCTCACGTGGTTC | 87557679 | 87557701 | 0.90 |
| GGCCATTCTCAGAAAATTCA | 87557706 | 87557728 | 0.00 |
| GCGAGAGGAGCAGAAGTTTA | 87557727 | 87557749 | 0.02 |
| GATGCAAGAGATGGAGGCGAG | 87557742 | 87557764 | 0.01 |
| GTTCCTACATGCAAGAGATGG | 87557749 | 87557771 | 0.00 |
| GCTCTTGCATGTAGGAAACAC | 87557756 | 87557778 | 0.30 |
| GAACACAGGCACGTACCACCA | 87557771 | 87557793 | 0.02 |
| GCACGTACCACCATGGCCAGA | 87557779 | 87557801 | 0.01 |
| GTTTTTTCCATCTGGCCATGG | 87557785 | 87557807 | 0.01 |
| GACTCCAATATTTTTTCCATC | 87557794 | 87557816 | 0.03 |
| GTTTAGTTTAGTGTCTTAGTC | 87557820 | 87557842 | 0.01 |
| GAAAACTAAACTAAACTGTAC | 87558203 | 87558225 | 0.01 |
| GTTTAGTTTTTTTTTTTTTTT | 87558216 | 87558238 | 0.02 |
| GAATTTCTTTGTGTAGACT | 87558256 | 87558277 | 0.01 |
| GCTCGCTCTGTAGACCAGGT | 87558288 | 87558310 | 0.01 |
| GTTCTGGATTCAAGACCAACC | 87558302 | 87558324 | 0.01 |
| GATATTTTAGCTGGGCAGTGG | 87558370 | 87558392 | 0.01 |
| GTTATTAACAATATTTTAGCT | 87558379 | 87558401 | 0.04 |
| GAAAGACTCACTGGGAAAAAA | 87558413 | 87558435 | 0.02 |

| | | | |
|---|---|---|---|
| GAAGGATTTCAAAGACTCACT | 87558422 | 87558444 | 0.03 |
| GTAAGGGTGAAAGACACAA | 87558441 | 87558462 | 0.01 |
| GCACCCTTACAATACCACCCA | 87558454 | 87558476 | 0.01 |
| GCTTTGACTGTTCCCATGGG | 87558467 | 87558489 | 0.07 |
| GGGAACAGTCAAAGCATGC | 87558475 | 87558496 | 0.00 |
| GTCAAAGCATGCAGGTGACT | 87558482 | 87558504 | 0.00 |
| GCATGCAGGTGACTGGGATGC | 87558489 | 87558511 | 0.01 |
| GGTGACTGGGATGCTGGACT | 87558495 | 87558517 | 0.12 |
| GGATGCTGGACTTGGGTTTC | 87558503 | 87558525 | 0.02 |
| GCAGGCAAACGTTAGCTTCAG | 87558522 | 87558544 | 0.01 |
| GTAGCTTCAGTGGTTAACCCC | 87558533 | 87558555 | 0.02 |
| GATGAGTCAGGAGGCCTCCT | 87558550 | 87558572 | 0.04 |
| GAAAGGGCATAAGATGAGTC | 87558562 | 87558584 | 0.63 |
| GACTCATCTTATGCCCTTTCT | 87558566 | 87558588 | 0.13 |
| GCTGAGAAACAATCCAAGAA | 87558579 | 87558601 | 1.33 |
| GCTGGGCCCAACAGCTCAG | 87558600 | 87558621 | 0.21 |
| GTTCTTTACCACTGAGCTGTT | 87558606 | 87558628 | 0.00 |
| GCTCAGTGGTAAAGAAATGG | 87558613 | 87558635 | 0.01 |
| GGGCTATTTGCACATGAAAA | 87558637 | 87558659 | 0.01 |
| GCAACTGGTTTGCTGAAGGGA | 87558660 | 87558682 | 0.01 |
| GAACCAGTTGCCAAGACAGAC | 87558674 | 87558696 | 0.00 |
| GACTGCTTTTCCTGTCTGTCT | 87558683 | 87558705 | 0.00 |
| GTTCTTACTCCTCATAAATGA | 87558705 | 87558727 | 0.00 |
| GACTCCTCATAAATGAAGGCC | 87558710 | 87558732 | 0.01 |
| GAAGGTATACACTGCCATGCC | 87558728 | 87558750 | 0.01 |
| GCTGGGTGCTGCTGGGATTAA | 87558747 | 87558769 | 0.02 |
| GTTGTGCCTCCTGGGTGCTGC | 87558756 | 87558778 | 0.20 |
| GACCCAGGAGGCACAAGCAGA | 87558764 | 87558786 | 0.47 |
| GTAGACTAATCTAGAACTGAC | 87558790 | 87558812 | 0.02 |
| GTTTGGAGGGGGGCAGGGGCA | 87558857 | 87558879 | 0.01 |
| GTTAGATTTGGAGGGGGGCAG | 87558862 | 87558884 | 0.03 |
| GAATCTATTAGATTTGGAGG | 87558869 | 87558891 | 0.01 |
| GCACTCAGAATCTATTAGATT | 87558875 | 87558897 | 0.02 |
| GAGAGTTTATCATTGGTGTT | 87558929 | 87558951 | 0.02 |
| GAGTGAGAGAGTTTATCAT | 87558936 | 87558957 | 0.51 |
| GTTAACTACTGTCAGCAGAGA | 87558962 | 87558984 | 1.00 |
| GAAAGCTAAGAAAAATGAGTG | 87558983 | 87559005 | 0.01 |
| GCCTGTGTGTGCATGGCTAT | 87559008 | 87559030 | 0.01 |
| GCACACAAGCCTGTGTGTGCA | 87559015 | 87559037 | 0.01 |
| GTGTCATTACTCAGACACAA | 87559148 | 87559170 | 0.01 |
| GTTACTCAGACACAAGGGACA | 87559154 | 87559176 | 0.01 |
| GACAAAATCACTCTCAGACTA | 87559186 | 87559208 | 0.01 |
| GCAGACTAAGGGAGACAACGA | 87559199 | 87559221 | 0.29 |
| GCTTCACTCTCTAGAGAAGTT | 87559222 | 87559244 | 0.02 |
| GTTCTCTAGAGAGTGAAGTGG | 87559228 | 87559250 | 0.01 |
| GTTGAACACCTACTACCTGCC | 87559256 | 87559278 | 0.03 |
| GCATGTACCAGGCAGGTAGT | 87559263 | 87559285 | 0.64 |
| GAAGTACGCATGTACCAGGC | 87559270 | 87559292 | 0.38 |
| GCATGATGACACTGGATGATA | 87559320 | 87559342 | 0.01 |
| GTAGCTGACATGATGACAC | 87559329 | 87559350 | 0.16 |
| GAACTGCATCATCTACTAAAT | 87559362 | 87559384 | 0.07 |
| GATGATGCAGTTAACTCGAA | 87559373 | 87559395 | 0.55 |

| | | | |
|---|---|---|---|
| GTTAACTCGAAAGGGGATGA | 87559382 | 87559404 | 0.01 |
| GATGAAGGATGGCAAGATGC | 87559397 | 87559419 | 0.01 |
| GGCAAGATGCTGGGACCCTG | 87559407 | 87559429 | 0.08 |
| GATGCTGGGACCCTGGGGCAC | 87559413 | 87559435 | 0.98 |
| GTTTGTGTGCCCAGTGCCCCA | 87559422 | 87559444 | 0.01 |
| GGGCACACAAAAAAAGAAGA | 87559434 | 87559456 | 0.01 |
| GAAAAAAGAAGAAGGAACGAG | 87559443 | 87559465 | 0.00 |
| GAAGAAGGAACGAGAGGGGT | 87559449 | 87559471 | 0.01 |
| GGAACGAGAGGGGTGGGAGG | 87559455 | 87559477 | 0.01 |
| GGGTGGGAGGTGGGGGGAGC | 87559465 | 87559487 | 0.02 |
| GGGAGCAGGATGGAGAGAGA | 87559479 | 87559501 | 0.06 |
| GGAGAGAGATGGAAGTTCCT | 87559490 | 87559512 | 0.00 |
| GGAAGTTCCTCGGAGAGAGC | 87559500 | 87559522 | 0.00 |
| GTCTCCACCCTGCTCTCTCCG | 87559507 | 87559529 | 0.43 |
| GCAGGGTGGAGACAAGTTCCA | 87559519 | 87559541 | 0.02 |
| GAACTGGGCACAGTGGCTCCG | 87559536 | 87559558 | 0.01 |
| GCTGAGCGAAACTGGGCACAG | 87559544 | 87559566 | 4.77 |
| GTCTGAACCTGAGCGAAACT | 87559552 | 87559574 | 0.38 |
| GCTCAGGTTCAGACGGGCTT | 87559561 | 87559583 | 4.74 |
| GACGGGCTTTGGCTCTTCCC | 87559572 | 87559594 | 1.32 |
| GCTCCAGCTCTTATCTATCC | 87559590 | 87559612 | 0.01 |
| GTAGATAAGAGCTGGAGCTTC | 87559596 | 87559618 | 1.31 |
| GTTCCTTGTTCATTCTGCAAT | 87559632 | 87559654 | 0.01 |
| GTTCATTCTGCAATAGGTGTG | 87559639 | 87559661 | 0.00 |
| GCAATAGGTGTGAGGATGAG | 87559647 | 87559669 | 0.01 |
| GAGCGGCCTGCAGTGACTGT | 87559664 | 87559686 | 0.44 |
| GGAGCCCAACAGTCACTGC | 87559670 | 87559691 | 0.65 |
| GTGACTGTTGGGCTCCACTG | 87559676 | 87559698 | 0.01 |
| GTTGGGCTCCACTGAGGTTCC | 87559683 | 87559705 | 0.01 |
| GGAGGCCCAGGAACCTCAG | 87559690 | 87559711 | 0.01 |
| GGCCTCCTCCTCTGCACCTT | 87559705 | 87559727 | 0.02 |
| GGAAGCCAAAGGTGCAGAGG | 87559710 | 87559732 | 0.04 |
| GATTGCCAAGCAGGAAGCCAA | 87559721 | 87559743 | 0.01 |
| GCTGATTGAGGATTGCCAAGC | 87559731 | 87559753 | 0.03 |
| GGCAATCCTCAATCAGGTGA | 87559738 | 87559760 | 0.01 |
| GACTGTGCCTTCACCTGATTG | 87559744 | 87559766 | 0.03 |
| GTCAGGTGAAGGCACAGTCGC | 87559750 | 87559772 | 0.71 |
| GCTGGCTTCCTTTTTCCACTT | 87559769 | 87559791 | 0.00 |
| GCTCACCCTAAGTGGAAAA | 87559776 | 87559797 | 0.02 |
| GAAAGGAGCTCACCCTAAG | 87559783 | 87559804 | 0.44 |
| GTAGGGTGAGCTCCTTTCAAT | 87559788 | 87559810 | 0.17 |
| GAGCAATTACACCTATTGAA | 87559799 | 87559821 | 11.90 |
| GGTCTTGCTCAGTGAACAGC | 87559821 | 87559843 | 0.01 |
| GCCGCATTCAGCTCAGTCGCC | 87559842 | 87559864 | 0.01 |
| GCTCTCAAGTGTAAGGATTAT | 87559874 | 87559896 | 0.01 |
| GTCCTTACACTTGAGAGGGTC | 87559881 | 87559903 | 0.84 |
| GTTGAGAGGGTCAGGAGTTCA | 87559890 | 87559912 | 0.01 |
| GCAGGAGTTCAAGGGCAATCT | 87559900 | 87559922 | 0.07 |
| GATCTTGGCCACATGTGAGTT | 87559916 | 87559938 | 0.08 |
| GCTGGCTCCAAACTCACATG | 87559923 | 87559945 | 0.03 |
| GCATATTTATTTGTCCCAGGC | 87559941 | 87559963 | 0.01 |
| GGGACAAATAAATATGGAAA | 87559948 | 87559970 | 0.03 |

| | | | |
|---|---|---|---|
| GAAAGGAAGCAAGTTAATTCT | 87559966 | 87559988 | 0.02 |
| GATAATTTATGTTCTCAGCC | 87559997 | 87560019 | 0.01 |
| GTTTATGTTCTCAGCCGGGCG | 87560002 | 87560024 | 0.01 |
| GAATTATCTTTTTTTGAGACT | 87560139 | 87560161 | 0.00 |
| GTATAGATCAACTTGCACATA | 87560173 | 87560195 | 1.07 |
| GATCAACTTGCACATAAGGAA | 87560178 | 87560200 | 0.38 |
| GAATGAAGACGAGTAAACGC | 87560200 | 87560222 | 0.48 |
| GACTCAGGAGAGAAGCAGCAT | 87560223 | 87560245 | 0.56 |
| GCTGCTTCTCTCCTGAGTGGC | 87560229 | 87560251 | 0.00 |
| GCTCTCCTGAGTGGCTGGTAC | 87560235 | 87560257 | 1.29 |
| GAGTGGCTGGTACAGGCGC | 87560242 | 87560263 | 0.09 |
| GGTACAGGCGCTGGTGTTTT | 87560250 | 87560272 | 0.02 |
| GAATGATAAGAACTACATAAC | 87560274 | 87560296 | 0.26 |
| GACATGTAACCAGTCATCCAG | 87560298 | 87560320 | 0.01 |
| GAGATTAACCACTGGATGAC | 87560306 | 87560328 | 2.07 |
| GACATGAAGAGATTAACCAC | 87560314 | 87560336 | 0.00 |
| GAATCTCTTCATGTCTAAAGC | 87560323 | 87560345 | 0.10 |
| GTTGCATCACAGCTTCCTCAA | 87560359 | 87560381 | 0.29 |
| GCCTATTGGAGTTTCCCTTTG | 87560373 | 87560395 | 0.69 |
| GTCCAATAGGACCCTTGACAA | 87560387 | 87560409 | 0.38 |
| GTAGGACCCTTGACAAAGGAA | 87560392 | 87560414 | 0.45 |
| GTTTTTCCTTTCCTTTGTCAA | 87560397 | 87560419 | 0.00 |
| GCAAAGGAAAGGAAAAATTTC | 87560404 | 87560426 | 0.05 |
| GCTGTCTAAATAAAGAAGCAT | 87560432 | 87560454 | 0.02 |
| GCATCCCAAAAAGCTGAGAT | 87560456 | 87560478 | 0.04 |
| GAGCAAGGGCACTTAGCTGC | 87560491 | 87560513 | 0.01 |
| GACGTTCATGATTTCAGAGCA | 87560506 | 87560528 | 0.01 |
| GAACGTTCAGAGTTCAAATCC | 87560524 | 87560546 | 1.74 |
| GTGAAGTCAGACTTCTTGTG | 87560550 | 87560572 | 0.15 |
| GTATCAACTTGTAACCTAGT | 87560574 | 87560596 | 0.01 |
| GTAACCTAGTTGGGGTTTGC | 87560584 | 87560606 | 0.47 |
| GGGGTTTGCTGGTGTGCAGC | 87560595 | 87560617 | 0.01 |
| GCTGGTGTGCAGCAGGTAGT | 87560602 | 87560624 | 0.73 |
| GCTGAGGTGCACAACTTGCTA | 87560632 | 87560654 | 0.09 |
| GAAGTTGTGCACCTCAGGAAG | 87560639 | 87560661 | 0.00 |
| GCAGTCTCTCCCTCTTCCTG | 87560649 | 87560671 | 1.18 |
| GAGGGAGAGACTGCCACAG | 87560658 | 87560679 | 0.03 |
| GAGACTGCCACAGAGGAAC | 87560664 | 87560685 | 0.01 |
| GATTCTCTGCCTGTTCCTCTG | 87560671 | 87560693 | 0.57 |
| GCACACAATGTGCCTTCTTC | 87560700 | 87560722 | 0.01 |
| GTTGATCTTGAAGCCAGAAGA | 87560712 | 87560734 | 0.01 |
| GGTTGGAGGCATAGTTCGTC | 87560740 | 87560762 | 0.01 |
| GAAAGTGGGGGGGAAGGTTGG | 87560754 | 87560776 | 0.13 |
| GTTGGAAAAAAGTGGGGGGGA | 87560761 | 87560783 | 0.02 |
| GACCATGTTTGGAAAAAAGT | 87560769 | 87560791 | 0.01 |
| GTTTTTTCCAAACATGGTCTC | 87560774 | 87560796 | 0.01 |
| GGGCTACCTGAGACCATGTT | 87560780 | 87560802 | 0.00 |
| GAGTGAGGGTGAGGTCAGCA | 87560801 | 87560823 | 0.01 |
| GTTTGTTACAGAGTGAGGGTG | 87560810 | 87560832 | 0.42 |
| GCCACCCTTTGTTACAGAGTG | 87560816 | 87560838 | 0.01 |
| GAAAGGGTGGCCTCCGTTTCC | 87560830 | 87560852 | 0.34 |
| GCAAGGGAGACCGGGAAACGG | 87560839 | 87560861 | 0.01 |

| | | | |
|---|---|---|---|
| GAAGGTAGAGACAAGGGAGAC | 87560849 | 87560871 | 0.09 |
| GCACTTGGAAGGTAGAGACAA | 87560856 | 87560878 | 0.01 |
| GCCTTGGTCTCAACACTTGGA | 87560868 | 87560890 | 0.01 |
| GGTATGGTGAGGCATGCCCT | 87560885 | 87560907 | 0.01 |
| GAAAATAAGTTGGGTATGGTG | 87560896 | 87560918 | 0.01 |
| GATAACAAAATAAGTTGGGTA | 87560901 | 87560923 | 0.02 |
| GAACTAATAACAAAATAAGTT | 87560906 | 87560928 | 0.01 |
| GAATTTTTTATTTTAATTTG | 87560931 | 87560953 | 0.04 |
| GTTTCTGGTCTCTTCAGAAAC | 87560993 | 87561015 | 0.01 |
| GGGGGTCTGATGCTCTTTC | 87561009 | 87561030 | 0.02 |
| GATCAGACCCCCTGGCACTGG | 87561020 | 87561042 | 0.58 |
| GTCTAGCCCTCCAGTGCCAGG | 87561026 | 87561048 | 0.01 |
| GTGAGATGCCACGAGGGTTC | 87561062 | 87561084 | 0.01 |
| GTTTGGTTCCCAGAACCCTCG | 87561070 | 87561092 | 0.50 |
| GGCAGCGAGGACCTAGGTT | 87561088 | 87561109 | 0.04 |
| GACTCGGCAGCGAGGACCT | 87561093 | 87561114 | 0.00 |
| GCTGGAGATGACTCGGCAGCG | 87561100 | 87561122 | 0.07 |
| GATGGGATGCTGGAGATGACT | 87561108 | 87561130 | 7.73 |
| GTGGTGGGGGATGGGATGC | 87561119 | 87561140 | 0.02 |
| GCTGGGAAGTGGTGGGGGAT | 87561126 | 87561148 | 0.02 |
| GAACCCAAGCTGGGAAGTGGT | 87561133 | 87561155 | 0.02 |
| GCCAAAACCAAAACCCAAGCT | 87561143 | 87561165 | 0.10 |
| GTAAATAGTTCACATACTA | 87561176 | 87561197 | 0.71 |
| GAAGCAAATTTTTGCTGCCCG | 87561210 | 87561232 | 0.01 |
| GATTTTTGCTGCCCGAGGTTG | 87561216 | 87561238 | 0.00 |
| GTTTAATTTGCCCTCAACCTC | 87561226 | 87561248 | 0.22 |
| GCAGCACAGGCTGCTATCA | 87561272 | 87561293 | 0.01 |
| GCTAGGAATGCTCAGCAGCAC | 87561284 | 87561306 | 0.07 |
| GATTCCTAGGGTTAAAGTAAA | 87561299 | 87561321 | 0.01 |
| GGAGATTTTGTTTCCTGCT | 87561320 | 87561341 | 0.02 |
| GCTGCACAGGTTAAACCGAGC | 87561333 | 87561355 | 0.08 |
| GTCTTGCACACAGATCTGCAC | 87561347 | 87561369 | 1.45 |
| GCAGATCTGTGTGCAAGACC | 87561352 | 87561374 | 0.02 |
| GTGCAAGACCTGGAATCTG | 87561362 | 87561383 | 0.01 |
| GCTTTAAGGTCCTCAGATTCC | 87561370 | 87561392 | 0.00 |
| GTCTGAGGACCTTAAAGGTCG | 87561377 | 87561399 | 0.01 |
| GTGGTACGCCTCGACCTTTA | 87561385 | 87561407 | 0.00 |
| GTCGAGGCGTACCACAGTCT | 87561393 | 87561415 | 0.01 |
| GCTGTCTGCAGCCTAGACTG | 87561404 | 87561426 | 33.75 |
| GGCTGCAGACAGCCTTACTT | 87561414 | 87561436 | 0.01 |
| GAACACACTGACTCCAAAGTA | 87561426 | 87561448 | 12.22 |
| GACACAGCAAGCAGTGATCCA | 87561465 | 87561487 | 0.01 |
| GCAAGCAGTGATCCAAGGAA | 87561470 | 87561492 | 0.01 |
| GCCTGAACTCAAACCCTTCCT | 87561482 | 87561504 | 0.46 |
| GCCTGACGAAGTTCTGGGATA | 87561562 | 87561584 | 3.07 |
| GTCTGTCCTGACGAAGTTCT | 87561568 | 87561590 | 0.99 |
| GACTTCGTCAGGACAGACCCA | 87561574 | 87561596 | 0.45 |
| GCCAGGCAACTGTGCTTCCGT | 87561590 | 87561612 | 0.01 |
| GACGTACTATAGATTCTATC | 87561612 | 87561634 | 0.11 |
| GATTCTATCTGGGAAAGCA | 87561623 | 87561644 | 0.44 |
| GTCTGGGAAAGCATGGAAGCA | 87561630 | 87561652 | 0.01 |
| GAAGCATGGAAGCAAGGCAGA | 87561637 | 87561659 | 0.01 |

| | | | |
|---|---|---|---|
| GCAGAAGGCCGTATCAGATTC | 87561653 | 87561675 | 0.17 |
| GCGAACCCTGAATCTGATA | 87561660 | 87561681 | 0.01 |
| GTCAGGGTTCGCTGACCAGGC | 87561671 | 87561693 | 0.01 |
| GTCATACGGTAGAACCCAGCC | 87561685 | 87561707 | 0.02 |
| GTTGATGGATGTCAGATCATA | 87561700 | 87561722 | 0.01 |
| GAAATTTCAATTTATTCTTGA | 87561716 | 87561738 | 0.01 |
| GTGCCTCCTGCAACAAACAT | 87561744 | 87561766 | 0.48 |
| GATCATGTCTGAGAACAATTC | 87561770 | 87561792 | 0.01 |
| GTGCCCTCTGCCTTTTCTCC | 87561800 | 87561822 | 0.01 |
| GAGCGAGGCTCCAGGAGAAA | 87561810 | 87561832 | 0.01 |
| GGAACTGTGAGCGAGGCTCC | 87561818 | 87561840 | 0.02 |
| GCTGTGGGGAACTGTGAGCG | 87561825 | 87561847 | 0.01 |
| GCCATCGTGAGCAGTGCTGTG | 87561839 | 87561861 | 0.11 |
| GACGATGGTAGTCCTTTGACG | 87561855 | 87561877 | 0.21 |
| GAAGGATTGTTGCCACGTCAA | 87561866 | 87561888 | 0.45 |
| GCACAGGTGCTCATTGCAGGA | 87561885 | 87561907 | 0.45 |
| GAGCACCTGTGACTGTTTCC | 87561897 | 87561919 | 0.04 |
| GCTTACCAGGAAACAGTCAC | 87561902 | 87561924 | 0.01 |
| GATCGTTGGTCTAGGCTTACC | 87561915 | 87561937 | 0.00 |
| GCTTCGTCATCGTTGGTCT | 87561924 | 87561945 | 0.01 |
| GCTTGGCTGCTTCGTCATCGT | 87561930 | 87561952 | 0.01 |
| GCAGCCAAGAAGCTAGTGTTC | 87561945 | 87561967 | 0.01 |
| GCCTAGTTTCATCTCTGTCA | 87561967 | 87561989 | 0.09 |
| GTTTGTCATGTCTCGTGCCTT | 87561994 | 87562016 | 3.84 |
| GCTCGTGCCTTTGGGTATGAG | 87562004 | 87562026 | 0.00 |
| GTTTGGGTATGAGTGGATCCC | 87562012 | 87562034 | 0.02 |
| GATCCCAGGGTGATCTCCTG | 87562026 | 87562048 | 0.41 |
| GGCGTTTGTGGCTTCCCAC | 87562042 | 87562063 | 5.35 |
| GGGAGAGCATGGACGGCGT | 87562271 | 87562292 | 0.02 |
| GTTTATTGGGAGAGCATGGA | 87562277 | 87562299 | 0.02 |
| GAAGTAGTAAAGAGGAGATTG | 87562353 | 87562375 | 0.01 |
| GCAATAAATTAAGTAGTAAAG | 87562362 | 87562384 | 0.02 |
| GTTTATTGATTATTCAGTGTT | 87562378 | 87562400 | 0.01 |
| GTTAAAGTATTTACTTTCTG | 87562417 | 87562439 | 0.01 |
| GAGAGAGAGGAGTGTAGAGC | 87562439 | 87562461 | 0.01 |
| GCTCTCTCTCTCTCGGGAGGG | 87562471 | 87562493 | 0.07 |
| GTCTCGGGAGGGTGGCAAGAC | 87562480 | 87562502 | 0.01 |
| GTAACAGAGCCCTGATTTTTC | 87562514 | 87562536 | 0.02 |
| GAGTCAGTCCAGAAAAATCA | 87562522 | 87562544 | 0.04 |
| GACTGACTCACTTGTAGACC | 87562536 | 87562558 | 0.09 |
| GCTTTAGTTACAGCACTCAGG | 87562594 | 87562616 | 0.04 |
| GGTGGTGGCAGAGGCGTTGG | 87562627 | 87562649 | 0.05 |
| GCTAAGTGGTGGTGGTGGTGG | 87562645 | 87562667 | 0.04 |
| GAACTCAAGCTAAGTGGTGG | 87562654 | 87562676 | 2.01 |
| GCTTTGAGAACTCAAGCTAAG | 87562660 | 87562682 | 0.30 |
| GAGTTCTCAAAGGATCCTGA | 87562671 | 87562693 | 0.51 |
| GATGGCAGGAAAGAGAAAGA | 87562689 | 87562711 | 0.03 |
| GGCAGGAAAGAGAAAGAAGG | 87562692 | 87562714 | 0.04 |
| GCCTGAACTAATTGGATCTTT | 87562729 | 87562751 | 0.06 |
| GTCCAATTAGTTCAGGGCCAC | 87562737 | 87562759 | 0.52 |
| GTACTGAGAAGTCACTCCTG | 87562753 | 87562775 | 0.01 |
| GCTGGCTCACACATACGGTAA | 87562785 | 87562807 | 0.51 |

| | | | |
|---|---|---|---|
| GTATGTGTGAGCCAGAGGTA | 87562793 | 87562815 | 0.01 |
| GAGGTAAGGTGGATGTCTAA | 87562807 | 87562829 | 0.02 |
| GTGGATGTCTAATGGTAGGC | 87562815 | 87562837 | 2.25 |
| GGTAGGCTGGGCCCTGACTT | 87562828 | 87562850 | 0.01 |
| GCTGGGCCCTGACTTGGGGC | 87562833 | 87562855 | 0.86 |
| GACACTCCAGCCCCAAGTCA | 87562839 | 87562861 | 0.00 |
| GGCTGGAGTGTCTGTTCCCA | 87562850 | 87562872 | 0.01 |
| GTCTGTTCCCATGGGTCAA | 87562859 | 87562880 | 0.24 |
| GGGCTCAACCTTTGACCCAT | 87562866 | 87562888 | 0.46 |
| GCAAAGGTTGAGCCCTTTCCT | 87562875 | 87562897 | 1.76 |
| GAATACGTGATTCCTAGGAAA | 87562886 | 87562908 | 3.54 |
| GAGTAAATACGTGATTCCT | 87562892 | 87562913 | 0.01 |
| GTCAAGGAATGAGATATTTGC | 87562924 | 87562946 | 0.07 |
| GAATAAATATCTGTAGCTTCA | 87562941 | 87562963 | 0.59 |
| GCATCTACTATGTTCTAGTAT | 87562967 | 87562989 | 0.02 |
| GATGTTCTAGTATAGGCACTG | 87562975 | 87562997 | 0.01 |
| GATAGGCACTGAGGATGTAGC | 87562985 | 87563007 | 0.01 |
| GTGTTACTTCTCATGTGTC | 87563010 | 87563031 | 0.01 |
| GCACATGAGAAGTAACACAGA | 87563015 | 87563037 | 0.02 |
| GCAGTAGATTAGACAGTGCTA | 87563048 | 87563070 | 4.14 |
| GAAAAACCAAGTCTAGAGGA | 87563072 | 87563094 | 0.10 |
| GCTGCTACCCTCCTCTAGACT | 87563078 | 87563100 | 0.03 |
| GATGCTTTTCGTAGGATGA | 87563108 | 87563129 | 0.03 |
| GTTATCTGAGATGCTTTTCGT | 87563115 | 87563137 | 0.82 |
| GCTGCAGTGAACACCCAGTTA | 87563149 | 87563171 | 0.02 |
| GAACACCCAGTTAAGGAACTG | 87563157 | 87563179 | 0.39 |
| GAACTGAGGATTGAGTCATGC | 87563172 | 87563194 | 0.01 |
| GATTGAGTCATGCAGGAGTC | 87563179 | 87563201 | 0.00 |
| GTCTGGGGAAATATTTGTTG | 87563196 | 87563218 | 0.34 |
| GCACAGAGGTAGAAATTCTTT | 87563220 | 87563242 | 0.01 |
| GAATTTCTACCTCTGTGGGC | 87563226 | 87563248 | 0.64 |
| GCTACCTCTGTGGGCAGGTGG | 87563232 | 87563254 | 0.00 |
| GTGGGCAGGTGGGGGGTGGG | 87563240 | 87563262 | 0.01 |
| GGGGGGTGGGGAGAGGAGGC | 87563257 | 87563279 | 0.04 |
| GGGGAGAGGAGGCTGGGCCC | 87563264 | 87563286 | 0.01 |
| GCTTTGGCTGGAATTTTGCCA | 87563281 | 87563303 | 0.02 |
| GTTCCAGCCAAAGGCCACCAG | 87563292 | 87563314 | 0.03 |
| GTTCCTTCCTCTGGTGGCCTT | 87563298 | 87563320 | 0.01 |
| GTTAGGTGTTCCTTCCTCTGG | 87563305 | 87563327 | 0.03 |
| GAGGAAGGAACACCTAAGCT | 87563311 | 87563333 | 0.01 |
| GTGCAATGGAAACCTAGCTT | 87563323 | 87563345 | 0.09 |
| GCTAGGTTTCCATTGCACAA | 87563328 | 87563350 | 0.01 |
| GTTAACAGTGCCCTTGTGCAA | 87563337 | 87563359 | 0.00 |
| GAAGTGTGTCGATCCTGTGTT | 87563358 | 87563380 | 0.08 |
| GTGTTAGGAACAAAAGCCGA | 87563373 | 87563395 | 0.01 |
| GCATAGTTTGTGAAGACCCT | 87563389 | 87563411 | 0.02 |
| GGTCTTCACAAACTATGCCC | 87563394 | 87563416 | 0.00 |
| GCACAAACTATGCCCAGGAAA | 87563400 | 87563422 | 0.02 |
| GCAAAACGTCTCCCATTTCCT | 87563411 | 87563433 | 0.51 |
| GAATGGGAGACGTTTTGTGTC | 87563418 | 87563440 | 0.01 |
| GGATCATTTTTCAGCTTCA | 87563439 | 87563460 | 0.53 |
| GAATTTATAAAAATAGTCTTC | 87563477 | 87563499 | 0.02 |

| | | | |
|---|---|---|---|
| GCTGAGCCCTTAGCAACCGTC | 87563517 | 87563539 | 1.12 |
| GAACACCAGACGGTTGCTAA | 87563522 | 87563544 | 0.01 |
| GGAAACCATGAACACCAGA | 87563532 | 87563553 | 0.01 |
| GTGTTCATGGTTTCCAAATG | 87563539 | 87563561 | 0.01 |
| GATTCTTTTTCTCACCACATT | 87563552 | 87563574 | 0.01 |
| GAAAGAATCCTCTGAACTGCT | 87563568 | 87563590 | 0.51 |
| GCCTTCATCCAAGCAGTTCAG | 87563575 | 87563597 | 0.01 |
| GATACTGACTTCAGCCTGCCC | 87563598 | 87563620 | 1.74 |
| GCAGGCGTGTGCCACCGGGGC | 87563611 | 87563633 | 0.31 |
| GATGACAGGCGTGTGCCACC | 87563616 | 87563638 | 0.02 |
| GCCTGTCATCCTAGCTACTG | 87563629 | 87563651 | 0.05 |
| GTCCTAGCTACTGAGGATGCC | 87563637 | 87563659 | 0.01 |
| GCTACTGAGGATGCCAGGGA | 87563642 | 87563664 | 0.01 |
| GAGGATGCCAGGGAGGGAGG | 87563648 | 87563670 | 0.03 |
| GGAGGGAGGTGGTAAGATAA | 87563659 | 87563681 | 0.39 |
| GGTAAGATAAAGGCTCACC | 87563669 | 87563690 | 0.01 |
| GGCTCACCTGGACAACTATG | 87563680 | 87563702 | 1.48 |
| GTCTCTCCACATAGTTGTCC | 87563686 | 87563708 | 0.01 |
| GTTTGTCAGAGTACTTGACT | 87563733 | 87563755 | 0.01 |
| GTATAGGGTCTTTTTTCACTT | 87563792 | 87563814 | 0.96 |
| GAAGGCTGAGGTGGAGTCTAT | 87563809 | 87563831 | 0.95 |
| GAACTTTGACCAAGGCTGAGG | 87563819 | 87563841 | 0.01 |
| GAAGGGCTGGAACTTTGACCA | 87563828 | 87563850 | 0.01 |
| GTCAAAGTTCCAGCCCTTGG | 87563833 | 87563855 | 0.33 |
| GTTCCAGCCCTTGGCGGGAGG | 87563840 | 87563862 | 0.01 |
| GTTCCCCCCACCTCCCGCCAA | 87563846 | 87563868 | 0.02 |
| GGGAGGTGGGGGGAAGGGGT | 87563854 | 87563876 | 0.02 |
| GGGAAGGGGTGGGCTGAGGC | 87563864 | 87563886 | 0.54 |
| GTTGCAGACAGAGTCTATCTC | 87563909 | 87563931 | 0.01 |
| GAATCCAGGTTTGTTTAGTT | 87563940 | 87563962 | 0.00 |
| GGGGTGATGGCTAGAATCC | 87563954 | 87563975 | 0.01 |
| GGGGACAGGGCTGGGGTGA | 87563966 | 87563987 | 0.02 |
| GCCCTCTGGGGACAGGGCTG | 87563972 | 87563994 | 0.00 |
| GCCCTGTCCCCAGAGGGCTC | 87563977 | 87563999 | 0.01 |
| GCCAGAATCCAGAGCCCTCT | 87563985 | 87564007 | 0.01 |
| GAGCCCTCTGGGGGCAGGGC | 87564014 | 87564036 | 0.01 |
| GCCCTGCCCCCAGAGGGCTC | 87564017 | 87564039 | 0.02 |
| GCAGACCCCAGAGCCCTCTG | 87564024 | 87564046 | 0.01 |
| GCATGTTTAGCCAGCACCCT | 87564045 | 87564067 | 0.04 |
| GTGGAGTCACCTAGGGTGC | 87564055 | 87564076 | 0.04 |
| GCCTGGAGTGGAGTCACCTA | 87564061 | 87564083 | 0.05 |
| GACTCCACTCCAGGCTGTTT | 87564069 | 87564091 | 0.22 |
| GTCTGAGCCTCCCAAACAGCC | 87564078 | 87564100 | 1.88 |
| GTCAGAGAGTGTTTACAAACA | 87564096 | 87564118 | 0.07 |
| GTTTACAAACAAGGGAAAGG | 87564105 | 87564127 | 0.02 |
| GCAAACAAGGGAAAGGAGGCC | 87564110 | 87564132 | 0.42 |
| GGGAAAGGAGGCCTGGTGAG | 87564117 | 87564139 | 0.11 |
| GGCAAGACATGCCTCTCACC | 87564128 | 87564150 | 0.03 |
| GAGAGGCATGTCTTGCCCAA | 87564134 | 87564156 | 0.01 |
| GTCTTGCCCAAGGGAGAAC | 87564143 | 87564164 | 0.33 |
| GTTGACTGACCAGTTCTCCCT | 87564150 | 87564172 | 0.05 |
| GGAGAACTGGTCAGTCAAGC | 87564155 | 87564177 | 1.04 |

| | | | |
|---|---|---|---|
| GACTGGTCAGTCAAGCAGGGC | 87564160 | 87564182 | 0.02 |
| GCAGTCACAGACAGAGGGAGT | 87564201 | 87564223 | 0.02 |
| GTAAAAACAGTCACAGACAGA | 87564207 | 87564229 | 0.15 |
| GACAAAGATAGCCTGTGATAT | 87564242 | 87564264 | 0.01 |
| GGCATCTGTTCCAATATCAC | 87564252 | 87564274 | 0.02 |
| GTATTGGAACAGATGCCAGGC | 87564259 | 87564281 | 0.47 |
| GATCTTAACAAATGCCCGGCC | 87564273 | 87564295 | 0.03 |
| GGCATATCTTAACAAATGCC | 87564278 | 87564300 | 0.01 |
| GTTAAGATATGCCAGTAATAA | 87564289 | 87564311 | 0.01 |
| GATTGTTATGTCCTTTATTAC | 87564299 | 87564321 | 0.11 |
| GCTAGGGCACTCAGCTCTCAA | 87564327 | 87564349 | 0.78 |
| GCCCTAGTTACAGTCTGTAA | 87564343 | 87564365 | 0.00 |
| GACAGTCTGTAAGGGAATGCT | 87564352 | 87564374 | 0.00 |
| GGAATGCTTGGTTCTGTTTA | 87564364 | 87564386 | 0.01 |
| GATATTATTCATCATTTTATT | 87564389 | 87564411 | 0.02 |
| GAATATGTGAGCTAACTGATT | 87564407 | 87564429 | 0.06 |
| GTGCTAAGGAGGCAGAAGC | 87564563 | 87564584 | 0.01 |
| GCTTTGCTCACAGTGCTAAGG | 87564573 | 87564595 | 0.01 |
| GTAAAAGGGTGGTATGGTGGT | 87564601 | 87564623 | 0.01 |
| GTCATAAATAAAAGGGTGGTA | 87564608 | 87564630 | 0.02 |
| GTAAATAAATCATAAATAAA | 87564617 | 87564639 | 0.05 |
| GTGTCCACAGAAACCAAACA | 87564686 | 87564708 | 0.01 |
| GCAGAAACCAAACAAGGGCAT | 87564693 | 87564715 | 0.01 |
| GAGGAGCCAATGCCCTTGTT | 87564699 | 87564721 | 0.01 |
| GATGTCTGATTCCAGTTCCAG | 87564718 | 87564740 | 0.00 |
| GTGAACTGCCTCGTGAGTGC | 87564745 | 87564767 | 0.01 |
| GTTAGATCCCCCGCACTCACG | 87564753 | 87564775 | 0.02 |
| GAGTGCGGGGGATCTAATCT | 87564759 | 87564781 | 1.28 |
| GGGATCTAATCTGGGTCCTC | 87564767 | 87564789 | 0.03 |
| GATTATTACCTTTTCATCTTC | 87564838 | 87564860 | 0.00 |
| GCTCACACCCAGAAGATGAAA | 87564845 | 87564867 | 0.03 |
| GCACCAGGGATGTATGTACA | 87564888 | 87564910 | 0.01 |
| GGTGCCCTCAAGTCCAGAAG | 87564906 | 87564928 | 0.02 |
| GACATCCTCTTCTGGACTTGA | 87564910 | 87564932 | 1.62 |
| GACATGGTAACTTTAGTAAC | 87564939 | 87564961 | 2.09 |
| GTAAAGTTACCATGTCGGAGC | 87564947 | 87564969 | 1.01 |
| GTACCATGTCGGAGCTGGGGG | 87564953 | 87564975 | 0.01 |
| GGTGGAACCTCTAGACCCTC | 87564971 | 87564993 | 0.11 |
| GTACTTTACCAGAGGGTCTAG | 87564978 | 87565000 | 0.01 |
| GACCCTCTGGTAAAGTATCC | 87564984 | 87565006 | 0.53 |
| GTATCCAGGGCTCTAAACCA | 87564998 | 87565020 | 0.02 |
| GAGATGGAAGGGTGGATCCG | 87565015 | 87565037 | 0.48 |
| GCTTTTATGGAGATGGAAGGG | 87565023 | 87565045 | 0.58 |
| GATTTACAACTTTTATGGAGA | 87565031 | 87565053 | 0.02 |
| GAATGTAATTTACAACTTTTA | 87565037 | 87565059 | 0.03 |
| GCACCACATAAACACACATGC | 87565122 | 87565144 | 0.01 |
| GCGCGCGTGTGCACTTGCT | 87565156 | 87565177 | 0.01 |
| GATATCAACTTGACACAAACT | 87565183 | 87565205 | 0.01 |
| GAACCGAGAAAATGCCTCTAT | 87565228 | 87565250 | 0.01 |
| GTTCACAGGTCTATCCTATAG | 87565241 | 87565263 | 0.01 |
| GATCACAACAGGTGTGTTCAC | 87565256 | 87565278 | 0.73 |
| GTTGTGATTTTCTTGATTGA | 87565271 | 87565293 | 0.01 |

| | | | |
|---|---|---|---|
| GATATGGGAGGGCCCACTGT | 87565296 | 87565318 | 0.02 |
| GGGAGGGCCCACTGTGGGTG | 87565301 | 87565323 | 0.01 |
| GAGGTGACCTCACCCACAGT | 87565308 | 87565330 | 0.04 |
| GAGGTCACCTCTGGACTGA | 87565320 | 87565341 | 0.01 |
| GACCTCTGGACTGATGGTCCT | 87565326 | 87565348 | 0.01 |
| GAAGAGCAAGCCAGTAGGC | 87565379 | 87565400 | 0.70 |
| GCATGGAGGAGTCCTGCCTAC | 87565389 | 87565411 | 0.01 |
| GAAGTTACAGAGAGCACTTG | 87565431 | 87565453 | 0.01 |
| GCTCTCTGTAACTTCCTTGGA | 87565440 | 87565462 | 0.02 |
| GCTAAATGGTCTACCATCCA | 87565453 | 87565475 | 0.01 |
| GTTAGCTCTCCTTCCCTCCTC | 87565471 | 87565493 | 0.02 |
| GCATCAACACTGTCTTAGTCA | 87565532 | 87565554 | 0.00 |
| GCATGCCCACCGTGTGTGTG | 87565562 | 87565584 | 0.04 |
| GATCTCCACACACACACGGT | 87565567 | 87565589 | 0.02 |
| GTGGAGATCAGAGGATAATA | 87565581 | 87565603 | 0.64 |
| GAGGATAATAAGGGGAATT | 87565591 | 87565612 | 1.10 |
| GACCTATGGGACCCAAATGG | 87565623 | 87565645 | 0.15 |
| GTTTGGGTCCCATAGGTCATC | 87565629 | 87565651 | 1.23 |
| GTCCCATAGGTCATCAGGCC | 87565634 | 87565656 | 0.02 |
| GATCAGGCCTGGTGACTGAGA | 87565646 | 87565668 | 0.01 |
| GCTGAGGCCATCTCAGTCACC | 87565652 | 87565674 | 0.71 |
| GGTCTTTACACTTTGTCTG | 87565669 | 87565690 | 0.62 |
| GCTGTGGATTTGGGTGTTGTC | 87565689 | 87565711 | 1.20 |
| GCTGGTAACTGCTGTGGATTT | 87565699 | 87565721 | 0.00 |
| GTCCACAGCAGTTACCAGAGA | 87565705 | 87565727 | 0.01 |
| GACTCTTCTCTTCCCCTTCTC | 87565718 | 87565740 | 0.13 |
| GGAAGAGAAGAGTGAAGCAA | 87565728 | 87565750 | 0.02 |
| GAGTGAAGCAAAGGACCAG | 87565737 | 87565758 | 0.02 |
| GGGGGAGGGAGGGTCTCCTC | 87565752 | 87565774 | 0.01 |
| GCCTGAGGATGGGGGGAGGGA | 87565762 | 87565784 | 3.77 |
| GAGACATCCCTGAGGATGGG | 87565770 | 87565792 | 2.29 |
| GAACTTACTGAGACATCCCTG | 87565778 | 87565800 | 0.00 |
| GTCTCAGTAAGTTTGCTGCTA | 87565789 | 87565811 | 0.02 |
| GTTTGCTGCTATGGGGGCC | 87565798 | 87565819 | 0.01 |
| GGGGGCCTGGAGATCACGAC | 87565810 | 87565832 | 0.01 |
| GTCATGCCAGTCGTGATCTCC | 87565815 | 87565837 | 0.07 |
| GCACGACTGGCATGAATTTGA | 87565824 | 87565846 | 0.01 |
| GCATGAATTTGAAGGGACCC | 87565832 | 87565854 | 0.03 |
| GAATTTGAAGGGACCCCGGGA | 87565837 | 87565859 | 0.01 |
| GCTGTCTCGCAAGTCCATCCC | 87565850 | 87565872 | 0.01 |
| GAGACGTGCATCAGAGAGCT | 87565890 | 87565912 | 0.01 |
| GCTGTGTGTGTGTTTATTTG | 87565924 | 87565946 | 0.00 |
| GTGTGTTTATTTGAGGTGTC | 87565931 | 87565953 | 0.01 |
| GAGGTGTCAGGAATAAACTT | 87565943 | 87565965 | 0.05 |
| GCAGGTCTGAGGGTGTTGTTC | 87565997 | 87566019 | 0.27 |
| GAGCATGTGAGGCAGGTCTG | 87566009 | 87566031 | 0.81 |
| GACCTGCCTCACATGCTCAT | 87566014 | 87566036 | 0.00 |
| GTACCTACCCATGAGCATGTG | 87566020 | 87566042 | 0.70 |
| GCTCATGGGTAGGTACTGTG | 87566028 | 87566050 | 3.31 |
| GGGTAGGTACTGTGAGGCT | 87566034 | 87566055 | 1.20 |
| GTGAGGCTTGGTGTGACCCA | 87566045 | 87566067 | 0.00 |
| GTTGGTGTGACCCATGGCAAG | 87566052 | 87566074 | 0.01 |

| | | | |
|---|---|---|---|
| GAGTGAGCTACCACTTGCCA | 87566062 | 87566084 | 0.00 |
| GTCTGGGTCTATATTGCACCC | 87566083 | 87566105 | 0.01 |
| GCTTGTAACTTCATTTTCCC | 87566101 | 87566123 | 0.02 |
| GAAGCAAGATCCCTCTCCTGA | 87566120 | 87566142 | 0.36 |
| GTCAGTCAGCCCTTCAGGAG | 87566130 | 87566152 | 0.07 |
| GCAGCTATGGCTGGAAGGCTG | 87566160 | 87566182 | 0.01 |
| GCCTTCCAGCCATAGCTGCT | 87566165 | 87566187 | 0.01 |
| GACAGTCCTAGCAGCTATGGC | 87566170 | 87566192 | 18.69 |
| GCTAGGACTGTCCTAATAGC | 87566182 | 87566204 | 0.00 |
| GACTGTCCTAATAGCTGGGC | 87566187 | 87566209 | 0.01 |
| GAATAGCTGGGCAGGAACACT | 87566196 | 87566218 | 0.73 |
| GTGACAGCAGCCCACATGTG | 87566287 | 87566309 | 0.07 |
| GTTCTGGGTTCCCACACATGT | 87566297 | 87566319 | 0.01 |
| GTCAGGAGGAGGGCGGCTTCT | 87566313 | 87566335 | 2.28 |
| GCAACAGGATCAGGAGGAGGG | 87566321 | 87566343 | 0.01 |
| GTCAAACAACAGGATCAGG | 87566328 | 87566349 | 0.00 |
| GCACTGATGTAGTCAAACAAC | 87566337 | 87566359 | 0.01 |
| GATCAGTGCTAAAGCCGCCAA | 87566353 | 87566375 | 0.45 |
| GTCTAGCTTTGCCCGTTGG | 87566366 | 87566387 | 0.53 |
| GGGCAAAGCTAGACTCCTC | 87566374 | 87566395 | 0.03 |
| GAAGGGGAAACTTTTTGCCTG | 87566389 | 87566411 | 0.01 |
| GTGGGGTGTAGGCTTGGAAG | 87566406 | 87566428 | 0.43 |
| GCTTGTTGTGGGGTGTAGGCT | 87566412 | 87566434 | 0.02 |
| GAAAGGCTTGTTGTGGGGTGT | 87566417 | 87566439 | 0.13 |
| GAACAACAAAGGCTTGTTGTG | 87566423 | 87566445 | 0.01 |
| GCTAGGGAGAACAAACAACAA | 87566435 | 87566457 | 0.01 |
| GTTGTTTGTTCTCCCTAGGT | 87566440 | 87566462 | 0.13 |
| GAAGGGCTTTTGTCCCCACCT | 87566453 | 87566475 | 0.01 |
| GAAAAGCCCTTTCAGATGCTG | 87566466 | 87566488 | 0.18 |
| GACATTCCACAGCATCTGAAA | 87566471 | 87566493 | 0.00 |
| GTACCAGAAGTGTCATGAGA | 87566492 | 87566514 | 0.02 |
| GTGTCATGAGAAGGGTTTGC | 87566501 | 87566523 | 0.07 |
| GTTTACAGGATTCTAGGTCA | 87566525 | 87566547 | 0.01 |
| GAATCCTGTAAACCAGGACA | 87566535 | 87566557 | 0.01 |
| GTAAACCAGGACAGGGAGC | 87566542 | 87566563 | 0.14 |
| GAAGGGCCAGCTCCCTGTCC | 87566547 | 87566569 | 0.01 |
| GCCCTTCTGCTTGAAGCTCC | 87566563 | 87566585 | 0.02 |
| GGTCCTGCCATGATTTCTGG | 87566584 | 87566606 | 1.21 |
| GCTGCCACCAGAAATCATGGC | 87566587 | 87566609 | 0.02 |
| GCACTGAAGGTACAGGGGGCA | 87566614 | 87566636 | 0.02 |
| GTCATTGCCACTGAAGGTAC | 87566622 | 87566644 | 0.23 |
| GGGGTGTCATTGCCACTGA | 87566628 | 87566649 | 4.53 |
| GCTGGGCTGTGGTGAGGACTG | 87566646 | 87566668 | 0.01 |
| GCAGTTTCCTGGGCTGTGGTG | 87566653 | 87566675 | 0.01 |
| GGCTGCAGTTTCCTGGGCTG | 87566658 | 87566680 | 0.01 |
| GTTCTCAGGCTGCAGTTTCCT | 87566664 | 87566686 | 0.30 |
| GCAGCCTGAGAAGGCCAGCC | 87566675 | 87566697 | 0.01 |
| GAGAAGGCCAGCCTGGTGCA | 87566682 | 87566704 | 0.01 |
| GGGTACGCCTTGCACCAGGC | 87566689 | 87566711 | 0.12 |
| GTACCCTCATTTGACTTTCCC | 87566707 | 87566729 | 0.01 |
| GAAGTGATATTGCATTCACC | 87566725 | 87566747 | 0.10 |
| GTCTGTCAGGCAGAAGCAGTG | 87566747 | 87566769 | 0.01 |

| | | | |
|---|---|---|---|
| GTCTGCCTGACAGATCTGCAG | 87566757 | 87566779 | 0.31 |
| GCTGCAGAGGGAAGGAGAACT | 87566771 | 87566793 | 4.90 |
| GTTAAAGATCATGACGACCCG | 87566814 | 87566836 | 0.01 |
| GTGGCCCCGCCCTTTATCAT | 87566833 | 87566855 | 0.54 |
| GCAGCCCCCATGATAAAGGGC | 87566838 | 87566860 | 0.01 |
| GTGTTCAGCCCCCATGATAA | 87566843 | 87566865 | 0.38 |
| GCTTGCTTTGTTCGCACTGT | 87566870 | 87566892 | 0.10 |
| GACAAAGCAAGCCGACTTTGG | 87566882 | 87566904 | 1.12 |
| GACTTTGGTGGTCCTGAGGA | 87566894 | 87566916 | 0.04 |
| GTTCACAGTGGCTCCTTCCTC | 87566906 | 87566928 | 0.01 |
| GGTGCATGACGATTCACAG | 87566919 | 87566940 | 0.04 |
| GGACAGACACGGCAGAGGAA | 87566939 | 87566961 | 0.02 |
| GACAGAGGACAGACACGGCAG | 87566944 | 87566966 | 0.01 |
| GTCAAGGACAGAGGACAGACA | 87566950 | 87566972 | 0.01 |
| GGGGAGACACTCAAGGACAG | 87566960 | 87566982 | 1.08 |
| GCACGCAAGGGGAGACACTCA | 87566967 | 87566989 | 0.26 |
| GTCTCCCCTTGCGTGGGGCC | 87566975 | 87566997 | 0.01 |
| GCTGTTCCTGGCCCCACGCAA | 87566980 | 87567002 | 0.01 |
| GGGGCCAGGAACAGAACAAA | 87566989 | 87567011 | 0.02 |
| GCTCCCTGCCAGACTCTACA | 87567014 | 87567036 | 0.01 |
| GACTGTAGCCATGTAGAGTC | 87567022 | 87567044 | 0.00 |
| GCTACATGGCTACAGTCTCAG | 87567029 | 87567051 | 0.59 |
| GTCTCAGAGGGAACACTTCC | 87567042 | 87567064 | 0.01 |
| GAAGAAGCAACAGTAGCAACC | 87567060 | 87567082 | 2.05 |
| GCTTCTTCTCTAACTCAGGTC | 87567077 | 87567099 | 0.01 |
| GAACTCAGGTCAGGCAACTCC | 87567087 | 87567109 | 0.07 |
| GTCAGGCAACTCCAGGGCGC | 87567094 | 87567116 | 0.42 |
| GGTGGCTGGTTCCAGCGCCC | 87567105 | 87567127 | 0.01 |
| GCTGGAACCAGCCACCGTGT | 87567112 | 87567134 | 0.71 |
| GCAAAGGTCCACCCACACGG | 87567123 | 87567145 | 3.42 |
| GCATTTTGCTAGCAGAGCAA | 87567139 | 87567161 | 3.57 |
| GCTAGCAAAATGCAAATGAGC | 87567150 | 87567172 | 0.00 |
| GTACCTGAAGTAAAACTGCAC | 87567169 | 87567191 | 0.01 |
| GCAGTTTTACTTCAGGTATAG | 87567175 | 87567197 | 0.04 |
| GTTACTTCAGGTATAGAGGTG | 87567180 | 87567202 | 1.53 |
| GGTATAGAGGTGAGGGAGGG | 87567188 | 87567210 | 0.01 |
| GTACCCTTGAGAGCAATGTGC | 87567224 | 87567246 | 0.01 |
| GAGCAATGTGCTGGCGGGGA | 87567233 | 87567255 | 1.78 |
| GTGCTGGCGGGGATGGAGA | 87567240 | 87567261 | 0.02 |
| GTCCAATCTTACACGCCAAAG | 87567271 | 87567293 | 0.02 |
| GCTTACACGCCAAAGCGGGGG | 87567277 | 87567299 | 0.08 |
| GCCAAAGCGGGGGAGGGAGA | 87567284 | 87567306 | 0.02 |
| GAGAGGGGACTGAAGAGTGA | 87567300 | 87567322 | 0.00 |
| GACTGAAGAGTGAAGGGTACG | 87567308 | 87567330 | 0.77 |
| GAAGGGTACGAGGATCAGAC | 87567318 | 87567340 | 0.00 |
| GTAGTATTACGCTCAGATAAG | 87567368 | 87567390 | 0.79 |
| GTTCTTATTACCCAAGCTCCC | 87567402 | 87567424 | 0.03 |
| GTACCCAAGCTCCCTGGGGAA | 87567409 | 87567431 | 0.01 |
| GGGGAATGGGTTTATGACTT | 87567423 | 87567445 | 0.02 |
| GACTTTGGATTCCATGAAGG | 87567438 | 87567460 | 0.01 |
| GGCAATTTAATCCTCCTTCA | 87567449 | 87567471 | 0.01 |
| GATTTGGGGCTCTTATCTGTA | 87567473 | 87567495 | 0.01 |

| | | | |
|---|---|---|---|
| GCCAGCCTATTTGGCTATTTG | 87567488 | 87567510 | 2.69 |
| GTAGCCAAATAGGCTGGTAGA | 87567495 | 87567517 | 0.40 |
| GGTAGAAGGAAACGAGAGAG | 87567509 | 87567531 | 0.01 |
| GGAAACGAGAGAGGGGTTGG | 87567516 | 87567538 | 0.01 |
| GGGGTTGGGGGGGGGTTCA | 87567528 | 87567550 | 0.02 |
| GGGGGTTCATGGTTGTCCTG | 87567539 | 87567561 | 0.87 |
| GCAGAGCAGAGACTGCCCCAC | 87567555 | 87567577 | 0.00 |
| GTGAAGAGAGAACACAGCAC | 87567582 | 87567604 | 0.00 |
| GCAAAATGTACACCAAGTCTG | 87567604 | 87567626 | 12.81 |
| GATGTCATCTGACCACAGACT | 87567615 | 87567637 | 0.02 |
| GTCATCACAGAGTGACATCAA | 87567639 | 87567661 | 2.05 |
| GAATGGTGTGCCTCCTTTGCC | 87567657 | 87567679 | 1.35 |
| GTCACTGGGGCCAGGCAAAGG | 87567666 | 87567688 | 0.28 |
| GGCCCCAGTGACAGGTGCTG | 87567678 | 87567700 | 0.58 |
| GACAGGTGCTGAGGACAGGGT | 87567688 | 87567710 | 0.01 |
| GAGGACAGGGTAGGATCTCA | 87567697 | 87567719 | 0.01 |
| GAAGGGCAAAACTGGAATCTC | 87567750 | 87567772 | 2.92 |
| GAACATCTGAAGGGCAAAAC | 87567759 | 87567781 | 2.91 |
| GTTATGGTGGGAACATCTGAA | 87567768 | 87567790 | 0.01 |
| GTTCGTTTCTCCTTTATGGT | 87567781 | 87567803 | 0.01 |
| GAAAGGTGGTGTTGTATTTC | 87567813 | 87567835 | 0.02 |
| GCTAGTGATACCCAGGAAAGG | 87567827 | 87567849 | 0.47 |
| GCACTTCTGCTAGTGATACCC | 87567835 | 87567857 | 1.04 |
| GTTTTAATGACTGTTGCTTTA | 87567887 | 87567909 | 0.01 |
| GAAGCAACAGTCATTAAAAAA | 87567892 | 87567914 | 0.01 |
| GTTAAAAAAAGGACTCAAGCC | 87567904 | 87567926 | 0.02 |
| GAAAGTATGATACATTATACC | 87567922 | 87567944 | 0.01 |
| GACTTCCTTGCAACGTGTCCT | 87567947 | 87567969 | 0.00 |
| GCAACGTGTCCTTGGCAAAG | 87567955 | 87567977 | 2.44 |
| GTCCTTGGCAAAGTGGGAGGC | 87567963 | 87567985 | 1.18 |
| GTGAGTCTCTTGAGCTGTAA | 87568015 | 87568037 | 0.01 |
| GCGAGTCTCTTGAGCTGCAG | 87568059 | 87568081 | 0.02 |
| GTTGAGTCTCTTGAGCTGTA | 87568192 | 87568214 | 0.01 |
| GTGAGTCTCTTGAGCTACAG | 87568367 | 87568389 | 0.01 |
| GACTCACCCCTACAGCTCAA | 87568405 | 87568427 | 0.68 |
| GTCAATACACTCTAGGTTAGC | 87568428 | 87568450 | 0.01 |
| GCTCTTATCTCAATACACTCT | 87568436 | 87568458 | 0.01 |
| GAGACCCTATGTCACAAACA | 87568458 | 87568480 | 0.01 |
| GCACAGACGTGGATGTCAAA | 87568504 | 87568526 | 0.01 |
| GACATCCACGTCTGTGCTG | 87568510 | 87568531 | 0.03 |
| GAGTACCACAGCACAGACG | 87568515 | 87568536 | 0.01 |
| GCATAAAATAATACATTTTAT | 87568560 | 87568582 | 0.03 |
| GGGAACTAGAGAGATCACTT | 87568581 | 87568603 | 0.04 |
| GAAGCGCTTGTTGTGCAAGTG | 87568610 | 87568632 | 0.00 |
| GCAAGTGTGGGAATTTGAGTT | 87568624 | 87568646 | 0.03 |
| GCTGACTTTTGTTGGGGTGCT | 87568650 | 87568672 | 0.01 |
| GAACACATCTGACTTTTGTTG | 87568657 | 87568679 | 0.00 |
| GCAATGATGGGGTTATGGTAT | 87568682 | 87568704 | 0.01 |
| GACCATAACCCCATCATTGAC | 87568687 | 87568709 | 0.00 |
| GTCTTTACCTGTCAATGATG | 87568694 | 87568716 | 0.01 |
| GCATTGACAGGTAAAGACAGC | 87568700 | 87568722 | 0.07 |
| GGTAAAGACAGCTGGATCCT | 87568708 | 87568730 | 0.34 |

| | | | |
|---|---|---|---|
| GCTGGATCCTGGGGACTCAG | 87568718 | 87568740 | 0.23 |
| GGGTTACCACTGAGTCCCC | 87568725 | 87568746 | 0.01 |
| GCTCACACTTTGATTAGGCA | 87568744 | 87568766 | 0.01 |
| GCCTGAGCTCACACTTTGATT | 87568749 | 87568771 | 0.00 |
| GTGTGAGCTCAGGGTTCAGT | 87568759 | 87568781 | 0.44 |
| GGGAGAACTTGTCTCAAAAA | 87568779 | 87568801 | 0.02 |
| GTAGGTAAAGGTCCTTGCCAC | 87568823 | 87568845 | 0.04 |
| GAGCTCAGACTCCTGTGGCA | 87568834 | 87568856 | 1.03 |
| GTATGGAGCTCAGACTCCTG | 87568839 | 87568861 | 0.00 |
| GCTCCATACCAGACCTCACAG | 87568854 | 87568876 | 0.60 |
| GGTTCTCCACTGTGAGGTC | 87568861 | 87568882 | 0.08 |
| GGGCTGGTTCTCCACTGTG | 87568866 | 87568887 | 0.01 |
| GTCAGAGAAGCTTTTGAGGGC | 87568881 | 87568903 | 0.01 |
| GTAGAGTCAGAGAAGCTTTTG | 87568886 | 87568908 | 1.75 |
| GAAAAACACATGCATGAGCAG | 87568917 | 87568939 | 0.02 |
| GTTTTAAACAGTGTATTCCTT | 87568936 | 87568958 | 0.02 |
| GAGCCATCTATCCCACCCGA | 87568952 | 87568974 | 0.07 |
| GGTGGGATAGATGGCTCAGC | 87568958 | 87568980 | 0.02 |
| GGCTCAGCAGGACCTATGTT | 87568970 | 87568992 | 0.02 |
| GGCGTTGGGAACCTAACAT | 87568982 | 87569003 | 0.02 |
| GTTAGGTTCCCAACGCCTGTA | 87568988 | 87569010 | 0.47 |
| GTGAGCTGCCATACAGGCGT | 87568996 | 87569018 | 0.01 |
| GTGTTTGTGAGCTGCCATAC | 87569002 | 87569024 | 0.96 |
| GCACCCACCCAGAGAGAGTGA | 87569022 | 87569044 | 0.01 |
| GTGACCCCATCACTCTCTCT | 87569028 | 87569050 | 5.87 |
| GGTTTATTTTATGCATGTGA | 87569067 | 87569089 | 0.01 |
| GCACTGCTTAATATTTGTTTT | 87569088 | 87569110 | 0.02 |
| GTGTAATCCTTTATAAAAAT | 87569108 | 87569130 | 0.02 |
| GGCTGCACCTATTTTTATAA | 87569115 | 87569137 | 0.02 |
| GTAGGTGCAGCCAGGTGATGA | 87569127 | 87569149 | 0.25 |
| GATTAAAGGCCATCATCACC | 87569136 | 87569158 | 0.01 |
| GATCTGAGTTTGAATCCAGCC | 87569193 | 87569215 | 0.01 |
| GACAAACAAACAAACTAAAAT | 87569281 | 87569303 | 0.04 |
| GCTGTTAGAAGAAGATTATAT | 87569305 | 87569327 | 0.01 |
| GAAACAGAATCTCAGAGATGC | 87569332 | 87569354 | 0.27 |
| GCAGGTTCACTCAGCAATA | 87569350 | 87569371 | 0.07 |
| GACTCAGCAATAAGGAGCAGA | 87569358 | 87569380 | 0.01 |
| GAAGGACTTAAGTGCTCCA | 87569376 | 87569397 | 0.01 |
| GCTCCAAGGTCTCTCAGCCC | 87569389 | 87569411 | 0.01 |
| GATAGTGAAGTCTGGTAGCC | 87569407 | 87569429 | 0.00 |
| GCTACCAGACTTCACTATCCC | 87569412 | 87569434 | 0.04 |
| GATCCCTGGTTAGACACTTGC | 87569427 | 87569449 | 0.02 |
| GTTAGACACTTGCAGGGATC | 87569434 | 87569456 | 0.01 |
| GCTTGCAGGGATCAGGGTATT | 87569442 | 87569464 | 0.04 |
| GGATCAGGGTATTTGGGAAC | 87569449 | 87569471 | 0.02 |
| GAACAGGAAGCTCTCTTCCC | 87569465 | 87569487 | 0.05 |
| GCATTGGGACTTTGGGGCCCT | 87569482 | 87569504 | 0.51 |
| GCCCCAAAGTCCCAATGTGG | 87569488 | 87569510 | 0.01 |
| GAAAACAGCTACCACCACAT | 87569499 | 87569521 | 0.01 |
| GTGGTAGCTGTTTTCCACCC | 87569507 | 87569529 | 0.11 |
| GCCATGGAGAGAGCTCCTGGG | 87569521 | 87569543 | 0.40 |
| GCTCTCTCCATGGGGTGCC | 87569531 | 87569552 | 0.01 |

| | | | |
|---|---|---|---|
| GGGTGTGGCCAGGCACCCCA | 87569538 | 87569560 | 0.00 |
| GTGAGCAAGTGGGTGTGGCC | 87569548 | 87569570 | 0.02 |
| GCAGAAAGCATGTGAGCAAGT | 87569558 | 87569580 | 0.02 |
| GCTCACATGCTTTCTGGGGG | 87569565 | 87569587 | 0.62 |
| GTCATCCTTGCTAGCTGCAGC | 87569601 | 87569623 | 0.01 |
| GACATTTACATGTAACCTTCT | 87569627 | 87569649 | 0.28 |
| GCTAGGTAATAGCCCTAGA | 87569641 | 87569662 | 0.13 |
| GCTATTACCTAGCATGACCCA | 87569651 | 87569673 | 0.59 |
| GACCCAAGGCTGGGATCTCA | 87569665 | 87569687 | 0.01 |
| GGTGTTGACAGAAGACCCAG | 87569686 | 87569708 | 0.09 |
| GTCTAATAAGGTGAGACCTC | 87569702 | 87569724 | 0.01 |
| GTCACTCTGCAAGGTCTAATA | 87569714 | 87569736 | 2.42 |
| GTAGACCTTGCAGAGTGACCT | 87569720 | 87569742 | 0.01 |
| GACACCAGAGATAGACTTCCG | 87569737 | 87569759 | 0.01 |
| GGTGTGAATAGTCACAATC | 87569755 | 87569776 | 0.02 |
| GTCACAATCAGGTTCCCTG | 87569765 | 87569786 | 0.00 |
| GTCCCTGTGGAACAAGAGACA | 87569778 | 87569800 | 0.02 |
| GCATTCACAGTGAGCGGTCTT | 87569803 | 87569825 | 0.47 |
| GTGTGTCATTCACAGTGAG | 87569810 | 87569831 | 0.00 |
| GAAAATAATGCCCCTGAACA | 87569837 | 87569859 | 1.89 |
| GAATGCCCCTGAACAAGGTAA | 87569843 | 87569865 | 0.01 |
| GATGCATGTGACATGCCCAC | 87569875 | 87569897 | 0.89 |
| GACATGCCCACGGGGTTTAA | 87569884 | 87569906 | 0.01 |
| GCAGATGTCCTTTAAACCCCG | 87569891 | 87569913 | 0.00 |
| GACAGCAGTCTCTGTTCTTG | 87569926 | 87569948 | 0.01 |
| GGGGCACTTCGGTCATCAGA | 87569952 | 87569974 | 0.02 |
| GACTCACGACTGGGGCACTT | 87569963 | 87569985 | 0.44 |
| GCCTATTCTAGACTCACGAC | 87569973 | 87569995 | 0.31 |
| GCTGCTTGGGATTTGCATACT | 87570021 | 87570043 | 0.15 |
| GTATGCAAATCCCAAGCAGGT | 87570026 | 87570048 | 0.05 |
| GCACACCCAACCTACCTGCTT | 87570035 | 87570057 | 0.01 |
| GGTTGGGTGTGGCAGCAGAT | 87570047 | 87570069 | 0.01 |
| GGCAGCAGATAGGGAAAATC | 87570057 | 87570079 | 0.38 |
| GTAGGGAAAATCTGGAACCCC | 87570066 | 87570088 | 0.02 |
| GAACCCCTGGTGTGACTGAG | 87570079 | 87570101 | 0.01 |
| GGTGTGACTGAGAGGGCGT | 87570087 | 87570108 | 0.01 |
| GACTGAGAGGGCGTTGGAGTC | 87570093 | 87570115 | 0.50 |
| GTCAGGAGAGTCCCAGCCAC | 87570110 | 87570132 | 0.01 |
| GCATGAACTCTGCCCGTGGC | 87570122 | 87570144 | 0.01 |
| GTCATGCTTGCCCGACTTCCC | 87570139 | 87570161 | 0.68 |
| GTTGCCCGACTTCCCTGGAAC | 87570145 | 87570167 | 0.01 |
| GTTGGAGCTCAGCCTGTTCCA | 87570156 | 87570178 | 0.01 |
| GCAGGCTGAGCTCCAAGCTCC | 87570164 | 87570186 | 0.71 |
| GACTAGAGCGGCCGGGAGCT | 87570175 | 87570197 | 0.01 |
| GTACTCCAGACTAGAGCGGCC | 87570182 | 87570204 | 0.02 |
| GCCAGATACTCCAGACTAGAG | 87570187 | 87570209 | 0.01 |
| GCTAGTCTGGAGTATCTGGAA | 87570192 | 87570214 | 0.01 |
| GCGATTAGGCAGAAACTATG | 87570222 | 87570244 | 0.01 |
| GCAGGAGAGTAAATAGCGATT | 87570236 | 87570258 | 0.02 |
| GTCTCCTGGGGTTTCAGAGAA | 87570252 | 87570274 | 0.01 |
| GTTTCAGAGAAAGGGTGAATC | 87570262 | 87570284 | 0.43 |
| GAAAGGGTGAATCAGGGAGC | 87570269 | 87570291 | 0.01 |

| | | | |
|---|---|---|---|
| GAATCAGGGAGCTGGGAAGAA | 87570278 | 87570300 | 0.17 |
| GGGAGCTGGGAAGAAAGGGT | 87570283 | 87570305 | 0.05 |
| GGGTAGGACAACCGCGCTTG | 87570299 | 87570321 | 2.05 |
| GCGGCTCTGAACCCCAAGCG | 87570310 | 87570332 | 0.02 |
| GAAATCTATACAACACTGCTG | 87570329 | 87570351 | 0.01 |
| GTAGATTTTCTTGTCTGAGCA | 87570345 | 87570367 | 0.01 |
| GTTGCGGGCTGAGGACTGGTT | 87570372 | 87570394 | 0.01 |
| GCAGCTGATTTTGCGGGCTG | 87570382 | 87570404 | 0.01 |
| GATGGACAGCAGCTGATTTTG | 87570389 | 87570411 | 0.01 |
| GTCAGCTGCTGTCCATTGCGC | 87570397 | 87570419 | 0.08 |
| GAAGGCAAGGCCCCAGCGCAA | 87570408 | 87570430 | 0.01 |
| GCAGGACTCAGACAAGGCA | 87570422 | 87570443 | 2.10 |
| GCAAACTGCAGGACTCAGACA | 87570427 | 87570449 | 0.01 |
| GTCCTGCAGTTTGAAACAGC | 87570437 | 87570459 | 0.01 |
| GCAGGTTCCACATCCGAGAAA | 87570456 | 87570478 | 0.01 |
| GCATCCGAGAAAAGGGAGTCT | 87570465 | 87570487 | 0.02 |
| GGCCCAAGACTCCCTTTTCT | 87570468 | 87570490 | 0.31 |
| GAGGAAGCACAGCAGAAACA | 87570489 | 87570511 | 0.02 |
| GCTGTGCTTCCTCGTGCTC | 87570499 | 87570520 | 0.93 |
| GTCGGCATTGTCCAGAGCACG | 87570508 | 87570530 | 0.00 |
| GACAATGCCGAGTCAAATCAT | 87570521 | 87570543 | 1.47 |
| GCAGGGTCCTATGATTTGACT | 87570527 | 87570549 | 0.16 |
| GTCATAGGACCCTGGAATGAC | 87570537 | 87570559 | 0.01 |
| GAGTACGGGCCGGTCATTCC | 87570546 | 87570568 | 0.38 |
| GGGAAGCGGGGAGTACGGGC | 87570556 | 87570578 | 0.84 |
| GATTGGCATAGACAGGGAAG | 87570570 | 87570592 | 0.59 |
| GATCATGATTGGCATAGACA | 87570576 | 87570598 | 0.01 |
| GCTAGCTGGTCGATCATGAT | 87570587 | 87570609 | 0.01 |
| GCATCTTTAGAACGCTAGC | 87570601 | 87570622 | 0.02 |
| GTTCTAAAGATGCTACTGC | 87570610 | 87570631 | 1.04 |
| GCACAGGTTGACGCTAACGTT | 87570632 | 87570654 | 0.10 |
| GATCATCTGGTTGTCTCCAC | 87570649 | 87570671 | 0.01 |
| GCCACAGAGTTATGATCATC | 87570662 | 87570684 | 1.67 |
| GATCATAACTCTGTGGCCAG | 87570668 | 87570690 | 0.03 |
| GAAACTCTGGATGCTCGCCAC | 87570684 | 87570706 | 0.01 |
| GGCGAGCATCCAGAGTTTAC | 87570689 | 87570711 | 0.02 |
| GATAAATACCCTGTAAACTC | 87570698 | 87570720 | 0.03 |
| GGGTATTTATCCACTTTTCA | 87570710 | 87570732 | 0.04 |
| GCCTCTCACATCCCTGAAAAG | 87570720 | 87570742 | 0.01 |
| GATGTGAGAGGAATAAAGTCT | 87570733 | 87570755 | 0.01 |
| GAGGAATAAAGTCTAGGAAA | 87570739 | 87570761 | 0.09 |
| GATGTCCCTGTTTGCTGTGCC | 87570793 | 87570815 | 0.01 |
| GATCCTTGGGCAAATACTTCC | 87570811 | 87570833 | 0.02 |
| GTACACAGTGCTGTTATCCTT | 87570825 | 87570847 | 0.01 |
| GTGTAGAACATGCTGCCACG | 87570843 | 87570865 | 0.01 |
| GCCTACAAAGGGCAGTCCACG | 87570858 | 87570880 | 0.01 |
| GATGCTTGTACGACCTACAAA | 87570870 | 87570892 | 0.02 |
| GGTCGTACAAGCATGAGGGA | 87570879 | 87570901 | 0.01 |
| GCATGAGGGAAGGGAAGACA | 87570889 | 87570911 | 0.03 |
| GTACAGCTGGCACACAGCAA | 87570924 | 87570946 | 0.02 |
| GCTGTGTGCCAGCTGTACGT | 87570929 | 87570951 | 0.01 |
| GGAGCCACCAACGTACAGC | 87570937 | 87570958 | 0.01 |

| | | | |
|---|---|---|---|
| GGCTCCATCTCGCTAACAGC | 87570953 | 87570975 | 0.02 |
| GGATGCTGCTGTCCCACAC | 87570972 | 87570993 | 0.07 |
| GACAGCAGCATCCTCCATCTC | 87570982 | 87571004 | 0.01 |
| GCATCCTCCATCTCTGGGAT | 87570988 | 87571010 | 0.05 |
| GCCTGCAGCCCATCCCAGAGA | 87570995 | 87571017 | 0.01 |
| GCACAGCTCTTCAGCACTTCT | 87571019 | 87571041 | 0.01 |
| GTGCTGAAGAGCTGTGGCCC | 87571026 | 87571048 | 0.00 |
| GCCATTGGCTGGCAAGCACCT | 87571043 | 87571065 | 0.02 |
| GTTCTCTGCTTCCCCATTGGC | 87571055 | 87571077 | 0.01 |
| GAAGCAGAGAAGCGACAAAGC | 87571068 | 87571090 | 1.03 |
| GAGAAGCGACAAAGCCGGTT | 87571073 | 87571095 | 0.58 |
| GCGACAAAGCCGGTTGGGGG | 87571078 | 87571100 | 2.30 |
| GCCTAAGCTCCCTCCCCCAAC | 87571087 | 87571109 | 0.01 |
| GGAGTGCAACTTTCCCCCC | 87571108 | 87571129 | 0.01 |
| GGCTCCTGTAGCTTCCAGGG | 87571121 | 87571143 | 0.02 |
| GAATGGCTCCTGTAGCTTCCA | 87571124 | 87571146 | 0.39 |
| GAAGCTACAGGAGCCATTGAA | 87571130 | 87571152 | 0.92 |
| GCAGGAGCCATTGAACGGCTG | 87571136 | 87571158 | 0.12 |
| GCACAGGCCACAGCCGTTCAA | 87571142 | 87571164 | 0.16 |
| GGCTGTGGCCTGTGGCGAAA | 87571151 | 87571173 | 0.12 |
| GTACAGGAGCCATTTCGCCAC | 87571159 | 87571181 | 0.01 |
| GAAATGGCTCCTGTAGCGAA | 87571167 | 87571189 | 0.08 |
| GCTCCTGTAGCGAAAGGCAT | 87571173 | 87571195 | 0.01 |
| GACAGCTGCTGGAGGGCTGTC | 87571206 | 87571228 | 0.01 |
| GTCACGGCTTGCACAGCTGC | 87571218 | 87571240 | 0.01 |
| GCTCTGGTTTTCATTATGTCA | 87571234 | 87571256 | 0.01 |
| GAAAACCAGAGAGCGTCGTC | 87571246 | 87571268 | 0.45 |
| GGACAGAGGTATGCCCTTC | 87571265 | 87571286 | 0.15 |
| GTTCTGTATGAGCAAGGACAG | 87571278 | 87571300 | 0.06 |
| GTCCTTGCTCATACAGAAGAG | 87571284 | 87571306 | 0.01 |
| GTCATACAGAAGAGAGGTCTC | 87571291 | 87571313 | 0.03 |
| GAGGTCTCTGGGCTCCAGGC | 87571303 | 87571325 | 0.01 |
| GGGTTATTGAACTGCCAGCC | 87571317 | 87571339 | 0.01 |
| GTTGGATCTAAAATTGTAAAA | 87571338 | 87571360 | 0.02 |
| GCAATTTTAGATCCAAGCCAG | 87571346 | 87571368 | 0.01 |
| GAAAACTGGGGGCCACTGGCT | 87571357 | 87571379 | 0.03 |
| GAAATTAAAACTGGGGGCCAC | 87571362 | 87571384 | 5.55 |
| GATAATCTAAATTAAAACTGG | 87571369 | 87571391 | 0.00 |
| GTAATTTAGATTATTGAAAGA | 87571379 | 87571401 | 0.03 |
| GATTATTGAAAGATGGTAAAT | 87571387 | 87571409 | 0.05 |
| GAAAGATGGTAAATTGGGCC | 87571393 | 87571415 | 0.01 |
| GAATTGGGCCAGGCGAGTGAG | 87571404 | 87571426 | 1.16 |
| GCTAGTGCCTCTCACTCGCC | 87571411 | 87571433 | 0.28 |
| GTGAGAGGCACTAGCGGCGT | 87571419 | 87571441 | 0.01 |
| GTAGCGGCGTGGGCACAGCGC | 87571430 | 87571452 | 0.01 |
| GCGCTGGCAAGCTGCTGCT | 87571446 | 87571467 | 0.00 |
| GTTCAGTCATGACATTGAGGT | 87571473 | 87571495 | 0.01 |
| GATCTAAGTCTGAATTTAGCT | 87571504 | 87571526 | 0.00 |
| GTAGCTCGGCCGTTCACTCAC | 87571519 | 87571541 | 0.01 |
| GACTCATGACCAGTGAGTGAA | 87571527 | 87571549 | 0.01 |
| GACTCACTGGTCATGAGTCAC | 87571533 | 87571555 | 0.02 |
| GTGTGCTTTTATTTCACCAT | 87571577 | 87571599 | 0.04 |

| | | | |
|---|---|---|---|
| GCTATGAAACATATTCCAA | 87571593 | 87571614 | 0.76 |
| GGCATTTCTTTCGTTGTTTC | 87571634 | 87571656 | 0.04 |
| GCATACAAGAGAGGGGCGGTG | 87571655 | 87571677 | 0.87 |
| GCAACGATCATACAAGAGAG | 87571663 | 87571685 | 0.01 |
| GCCTACTGTTTATATAAGC | 87571684 | 87571705 | 0.25 |
| GACTGAACGGCAAGAATGTGC | 87571702 | 87571724 | 0.01 |
| GCTTGCCGTTCAGTAAATGCT | 87571712 | 87571734 | 0.01 |
| GGTGATGCAATCAGGCTCAC | 87571745 | 87571767 | 0.01 |
| GCCTGATTGCATCACCGGCA | 87571752 | 87571774 | 0.01 |
| GATCACCGGCATGGCATCATC | 87571762 | 87571784 | 0.01 |
| GCATCATCTGGTTGTCCCCTG | 87571775 | 87571797 | 0.00 |
| GCGGCAGTATTGCTCCACAG | 87571789 | 87571811 | 0.01 |
| GGAGCAATACTGCCGCTCAG | 87571796 | 87571818 | 0.01 |
| GCCGCTCAGTGGATGAATGT | 87571807 | 87571829 | 0.02 |
| GTGGATGAATGTGGGATGGC | 87571815 | 87571837 | 0.03 |
| GCCCCACCCTTCATTCCACA | 87571853 | 87571875 | 0.02 |
| GACCCTTCATTCCACAAGGAC | 87571858 | 87571880 | 1.00 |
| GTCGGAGTTTCCCTGTCCTTG | 87571868 | 87571890 | 0.82 |
| GGGAAACTCCGAGCCTGCTC | 87571879 | 87571901 | 0.70 |
| GAACAGCCTCCTGAGCAGGCT | 87571887 | 87571909 | 0.01 |
| GACAAAACAGCCTCCTGAGC | 87571892 | 87571914 | 0.89 |
| GTTGTTTTTGTTTTAGTATCA | 87571914 | 87571936 | 0.03 |
| GCAAAACTCACAGGGATTGAA | 87571947 | 87571969 | 0.01 |
| GCTCAAGGTTCAAAACTCAC | 87571957 | 87571979 | 0.21 |
| GGTGTTCTTTCTTTAGCTCA | 87571972 | 87571994 | 0.01 |
| GTATTTTTAAATTTTTGAGAT | 87572018 | 87572040 | 0.02 |
| GATAGGTCTCACAATACAGGT | 87572036 | 87572058 | 0.01 |
| GACAATACAGGTCGGTCCATC | 87572045 | 87572067 | 0.01 |
| GATTGAGTGTTTAAAGCCAGA | 87572060 | 87572082 | 0.02 |
| GATTTTGGAAGCGGGGGCAGG | 87572083 | 87572105 | 0.01 |
| GTTCCAGCATTTTGGAAGCGG | 87572090 | 87572112 | 0.03 |
| GCATCTCTAATTCCAGCATTT | 87572099 | 87572121 | 0.01 |
| GAGATGTGTGCCAAAACTTG | 87572116 | 87572138 | 0.00 |
| GTGATTTCACCACAAGTTT | 87572126 | 87572147 | 0.01 |
| GAATCACAATCTTAACTAGTC | 87572142 | 87572164 | 0.01 |
| GGGAACAACTTTCTAGACT | 87572163 | 87572184 | 0.06 |
| GCAGGGGGCACTGCAGGACA | 87572185 | 87572207 | 1.22 |
| GCATGGGGCAGGGGGCACTGC | 87572191 | 87572213 | 0.01 |
| GTTAGTGGCACATGGGGCAG | 87572201 | 87572223 | 0.04 |
| GTTCTTGTTAGTGGCACATG | 87572207 | 87572229 | 0.03 |
| GCTCTCAGAGGTTCTTGTTAG | 87572216 | 87572238 | 2.21 |
| GTTTCATGTTCAACCTCTCAG | 87572229 | 87572251 | 0.01 |
| GAACATGAAATGACAAGAACC | 87572243 | 87572265 | 0.16 |
| GTGTTTGCTTTAGAATGCC | 87572261 | 87572282 | 0.14 |
| GCTAAAGCAAACACAGAAGAG | 87572270 | 87572292 | 0.03 |
| GCAAACACAGAAGAGAGGTAT | 87572276 | 87572298 | 0.03 |
| GCACTGCTAAGAAACGTTGG | 87572303 | 87572325 | 0.22 |
| GAAACGTTGGTGGGAACCATC | 87572314 | 87572336 | 0.01 |
| GGGAACCATCAGGTCTCAGT | 87572324 | 87572346 | 0.01 |
| GTGTTCCAACTGAGACCTGA | 87572329 | 87572351 | 0.01 |
| GTTGTTGTTGCTCTCTTCTC | 87572357 | 87572379 | 0.01 |
| GCCCACCAACTATCTACCTG | 87572392 | 87572414 | 0.01 |

| | | | |
|---|---|---|---|
| GAACTATCTACCTGGGGGCGG | 87572399 | 87572421 | 0.01 |
| GCCCTCCCTCCTCCGCCCCC | 87572408 | 87572430 | 0.02 |
| GTCTGAGTCTGAGGGCCATCC | 87572476 | 87572498 | 12.78 |
| GGGCTTTTCTGTGTGGCCC | 87572523 | 87572544 | 0.01 |
| GTAAGACAGGGCTTTTCTGTG | 87572529 | 87572551 | 0.01 |
| GTTGGTTGTTGTTTTAAGACA | 87572542 | 87572564 | 5.80 |
| GTTAAAACAACAACCAAAGCC | 87572549 | 87572571 | 0.87 |
| GCAACAACCAAAGCCAGGCTG | 87572555 | 87572577 | 0.03 |
| GTATTTCTTTTGTTTTATGCT | 87572722 | 87572744 | 0.01 |
| GATAAAACAAAAGAAATAAGA | 87572728 | 87572750 | 0.02 |
| GAAGAAATAAGAAGGGAGAAA | 87572737 | 87572759 | 0.01 |
| GATATTAACAAAATCACCTA | 87572759 | 87572781 | 1.34 |
| GCAGACTGCAATATATCCTT | 87572775 | 87572797 | 0.49 |
| GATATATTGCAGTCTGCAGGA | 87572782 | 87572804 | 0.22 |
| GCAGGAAGGGCCTGAAGTTG | 87572796 | 87572818 | 0.82 |
| GGGCCTGAAGTTGAGGTTAC | 87572803 | 87572825 | 0.42 |
| GTACAGGTGCTAGCCCCACCC | 87572820 | 87572842 | 0.00 |
| GGCTTATCTAGTCCTGGGTG | 87572832 | 87572854 | 0.78 |
| GACTGTGGCTTATCTAGTCC | 87572838 | 87572860 | 0.01 |
| GACTAGATAAGCCACAGTCCT | 87572843 | 87572865 | 1.21 |
| GCCACAGTCCTTGGTGCTTC | 87572852 | 87572874 | 0.15 |
| GCAGGGCCTCCGGAAGCACCA | 87572860 | 87572882 | 0.00 |
| GTCCGGAGGCCCTGGTCTCTA | 87572870 | 87572892 | 0.81 |
| GTTTGGGAACCATAGAGACCA | 87572878 | 87572900 | 0.02 |
| GTCTCTATGGTTCCCAAAAG | 87572883 | 87572905 | 0.64 |
| GCCTCTGCTGCCCCCACTTTT | 87572895 | 87572917 | 0.01 |
| GGGGCAGCAGAGGGCCCACA | 87572905 | 87572927 | 0.01 |
| GCAGAGGGCCCACAAGGAT | 87572911 | 87572932 | 1.57 |
| GTTGTGGTGTCCTATCCTTGT | 87572919 | 87572941 | 0.02 |
| GTTTGCTCAAAGATAGTTTG | 87572936 | 87572958 | 0.05 |
| GAGTAGTACATAAAAACCAC | 87572961 | 87572983 | 0.56 |
| GTACATAAAAACCACAGGGCA | 87572967 | 87572989 | 0.01 |
| GTTGTCTTCATCCTTGCCCTG | 87572977 | 87572999 | 0.01 |
| GCAAGGATGAAGACAAGAGCT | 87572985 | 87573007 | 0.40 |
| GACAAGAGCTCGGCCAGTAA | 87572995 | 87573017 | 0.02 |
| GTAAAGGCTCTTGTCGCCTG | 87573011 | 87573033 | 1.12 |
| GGTGTCTAACGCTGGTCCTC | 87573027 | 87573049 | 0.01 |
| GGGTTATGGTGTCTAACGC | 87573035 | 87573056 | 0.01 |
| GTTAGACACCATAACCCACA | 87573040 | 87573062 | 0.01 |
| GTCTGTTCCCCATGTGGGTTA | 87573048 | 87573070 | 0.71 |
| GTCCCCCTCTGTTCCCCATG | 87573055 | 87573077 | 0.67 |
| GGGACCAACCAAGTCCTGCC | 87573073 | 87573095 | 0.01 |
| GAGGGCACCTGGCAGGACT | 87573081 | 87573102 | 0.61 |
| GAAGTCAGAGGGCACCTGGC | 87573087 | 87573109 | 0.01 |
| GTAGTACACGTGTGAAGTCAG | 87573099 | 87573121 | 0.17 |
| GCTTGTTTGAGATGAAACTTT | 87573205 | 87573227 | 0.02 |
| GTAGGAAAACACTGCTTCTGT | 87573224 | 87573246 | 0.05 |
| GACTTCTTCAGTTGTGTTA | 87573254 | 87573275 | 0.02 |
| GACTCATTTTTCTCAAAAAA | 87573316 | 87573338 | 0.02 |
| GATCAAAGATGTTATAGAA | 87573371 | 87573392 | 0.02 |
| GTTTGCCACAGTCTTTCACCC | 87573401 | 87573423 | 0.03 |
| GGTTGGTGGCAATTGTGCCG | 87573418 | 87573440 | 0.00 |

| | | | |
|---|---|---|---|
| GGACTCTGTAAAGCGGTTGG | 87573432 | 87573454 | 0.01 |
| GGGGTGGGGACTCTGTAAAG | 87573439 | 87573461 | 0.01 |
| GCTGATTGGGGTGGGGGGTG | 87573453 | 87573475 | 0.02 |
| GTAAAACTGATTGGGGTGGG | 87573459 | 87573481 | 0.00 |
| GGCCTCTGTAAAACTGATTG | 87573466 | 87573488 | 1.06 |
| GAAGAGACAAGAGGAATGGGT | 87573487 | 87573509 | 0.02 |
| GCAATGTCACCAAGAGACAAG | 87573497 | 87573519 | 1.93 |
| GACATTGACCTTAATTAGGT | 87573513 | 87573535 | 0.03 |
| GCACCAAGCCAACCTAATTA | 87573521 | 87573543 | 0.10 |
| GCACCATCTTGACATATGT | 87573549 | 87573570 | 0.01 |
| GCTTGACATATGTAGGCTCAT | 87573556 | 87573578 | 0.06 |
| GGATGCGAGCACACAGAGAA | 87573577 | 87573599 | 0.04 |
| GAGCACACAGAGAAGGGGC | 87573583 | 87573604 | 0.02 |
| GCACAGAGAAGGGGCTGGCGA | 87573588 | 87573610 | 0.01 |
| GAAGGGGCTGGCGATGGTTAA | 87573595 | 87573617 | 0.01 |
| GGCGATGGTTAAAGGGTTT | 87573603 | 87573624 | 0.05 |
| GAAAGGGTTTTGGCAGCTTTG | 87573613 | 87573635 | 7.69 |
| GTTTCACACTCAATGCCGTCG | 87573637 | 87573659 | 0.67 |
| GACTCAATGCCGTCGTGGACA | 87573643 | 87573665 | 0.17 |
| GACAACCACCCTTGTCCACGA | 87573651 | 87573673 | 0.09 |
| GACATTCACGTTGTTTTAT | 87573686 | 87573707 | 0.02 |
| GATGTCTATCACGCCTCCAGC | 87573703 | 87573725 | 0.50 |
| GCTGAGGAAGGCACCTGCTGG | 87573715 | 87573737 | 0.00 |
| GGTGCCTTCCTCAGCCATGT | 87573724 | 87573746 | 0.14 |
| GATAATCCACCAACATGGCTG | 87573732 | 87573754 | 0.80 |
| GTTACTCATAATCCACCAACA | 87573738 | 87573760 | 0.60 |
| GCAATCTCCTTGCAACTTGG | 87573776 | 87573798 | 0.37 |
| GTCCTTGCAACTTGGTGGCAT | 87573782 | 87573804 | 0.84 |
| GCAACTTGGTGGCATAGGGT | 87573787 | 87573809 | 0.02 |
| GGTGGCATAGGGTGGGGCTC | 87573794 | 87573816 | 1.39 |
| GGGGAGTGTGTTGAGCATGG | 87573814 | 87573836 | 0.01 |
| GCATGGTGGCTCGAATAGAAA | 87573829 | 87573851 | 0.01 |
| GCATTCGAATATGAACTTAC | 87573854 | 87573876 | 0.01 |
| GTATTCGAATGCTTGGTCACT | 87573866 | 87573888 | 0.00 |
| GACTAGGGGTAGCCTTGTCAG | 87573883 | 87573905 | 0.00 |
| GTCAGAGGACTGTGTCACTA | 87573898 | 87573920 | 8.13 |
| GCAGAGACTCTCAAGGGCC | 87573949 | 87573970 | 0.01 |
| GCAGAGAAGCAGAGACTCTCA | 87573955 | 87573977 | 4.10 |
| GCAAGCAGATGTACAGGCAAC | 87573978 | 87574000 | 0.02 |
| GTTCTACAAGCAGATGTAC | 87573985 | 87574006 | 0.05 |
| GCATGTAGGCAGACCTGGTGC | 87574020 | 87574042 | 0.02 |
| GCAGAGCATGGTGGCATGT | 87574035 | 87574056 | 0.02 |
| GTTATGGCAGGGAGCAGAGCA | 87574046 | 87574068 | 0.01 |
| GTATCCATTATCATTATGGCA | 87574058 | 87574080 | 0.01 |
| GCTTTTATCCATTATCATTA | 87574063 | 87574085 | 0.03 |
| GAAAGAAAGAATCTAATTG | 87574104 | 87574125 | 0.93 |
| GTGAAGGGACATTATAACTA | 87574136 | 87574158 | 0.02 |
| GATAATGTCCCTTCACAGCAA | 87574144 | 87574166 | 0.01 |
| GCAGTGTCCCATTGCTGTGAA | 87574151 | 87574173 | 0.01 |
| GGGACACTGAGATAGAAGC | 87574165 | 87574186 | 0.01 |
| GCTGAGATAGAAGCTGGTACC | 87574171 | 87574193 | 0.62 |
| GATAGAAGCTGGTACCAGGAT | 87574176 | 87574198 | 0.01 |

| | | | |
|---|---|---|---|
| GTTGACCATGCTTTTGTTCAG | 87574217 | 87574239 | 0.01 |
| GCTTTTGTTCAGAGGAATG | 87574225 | 87574246 | 0.07 |
| GCAGAGGAATGTGGAACGTTT | 87574234 | 87574256 | 0.00 |
| GTGGAACGTTTTGGGACTT | 87574243 | 87574264 | 0.01 |
| GGGACTTTGGTCTAGAAAAG | 87574255 | 87574277 | 0.21 |
| GTGGTTGGACACTTTGAGCA | 87574274 | 87574296 | 0.48 |
| GACACTTTGAGCAGGGCTTAA | 87574282 | 87574304 | 0.01 |
| GAGCAGGGCTTAATGGAGCA | 87574289 | 87574311 | 0.03 |
| GCATGGAAGACAGTCGAGCTG | 87574307 | 87574329 | 0.45 |
| GCTGAGGAAGCTTTGAACTG | 87574323 | 87574345 | 0.01 |
| GTGGAAGAATATTAATATG | 87574368 | 87574389 | 0.01 |
| GTATCCACAGGAAAGGTCTCT | 87574390 | 87574412 | 0.20 |
| GCTAAAATATCCACAGGAA | 87574398 | 87574419 | 3.76 |
| GTTCTTTGCTAAAATATCCAC | 87574403 | 87574425 | 0.02 |
| GGATATTTTAGCAAAGAATG | 87574408 | 87574430 | 0.04 |
| GCAAAGAATGTGGCTGGGTT | 87574418 | 87574440 | 0.03 |
| GCAGGTTGGTTTTTTAGACTA | 87574441 | 87574463 | 0.02 |
| GTCTTCAATTTAGCCACAGGT | 87574456 | 87574478 | 0.14 |
| GTGGCTAAATTGAAGAGTTT | 87574463 | 87574485 | 0.03 |
| GAGTTTTGGATTCATGGCTT | 87574477 | 87574499 | 0.01 |
| GGATTCATGGCTTTGGCCA | 87574484 | 87574505 | 0.01 |
| GACACTGCGGTGAAATCTCCT | 87574500 | 87574522 | 1.57 |
| GACAGAGTAAATACTACACTG | 87574514 | 87574536 | 0.46 |
| GTAGTATTTACTCTGTTGTG | 87574521 | 87574543 | 0.01 |
| GTTACTCTGTTGTGTGGTCAT | 87574528 | 87574550 | 0.23 |
| GTTGTGTGGTCATTGGCGA | 87574535 | 87574556 | 0.01 |
| GCTCTTACGCAGCTCTACAA | 87574556 | 87574578 | 0.01 |
| GTACGCAGCTCTACAATGGAA | 87574561 | 87574583 | 0.00 |
| GGAAAGGAACAAGCCAAGCA | 87574577 | 87574599 | 0.01 |
| GCATTTTGTATTTTCCTTGCT | 87574590 | 87574612 | 0.01 |
| GAATGTATAGTTTGAGAAGAA | 87574609 | 87574631 | 0.01 |
| GTTTAACTTCATTACACATCT | 87574637 | 87574659 | 0.01 |
| GAAGTTAAATCCTGTGCTCA | 87574651 | 87574673 | 0.05 |
| GCTATTCATCTCCTTGAGCAC | 87574661 | 87574683 | 1.38 |
| GAATAGATTAAAGAGAAGCC | 87574678 | 87574700 | 0.03 |
| GACTTTGTTCTGTGTAGCACC | 87574696 | 87574718 | 0.02 |
| GACAAAGTGAGCGCTGACCTC | 87574712 | 87574734 | 0.50 |
| GCTGACTGGCATCTTGCCTG | 87574728 | 87574750 | 0.01 |
| GAATTGGGAAGCTTAGCTGAC | 87574742 | 87574764 | 8.16 |
| GAAGCTTCCCAATTTGTGAAG | 87574752 | 87574774 | 0.27 |
| GTTAATTCCACTTCACAAATT | 87574758 | 87574780 | 0.03 |
| GTGGAATTAAAGAAAAGCCC | 87574771 | 87574793 | 0.02 |
| GAAAGCCCAGGGCCAATGTGA | 87574784 | 87574806 | 0.01 |
| GTGAGCCATCACATTGGCCC | 87574789 | 87574811 | 0.13 |
| GAAAGGTGTGAGCCATCACAT | 87574795 | 87574817 | 10.34 |
| GCTTTGTAGATCAGGCAGC | 87574867 | 87574888 | 0.00 |
| GCTGTTTAAGCTTGGCTGTCC | 87574894 | 87574916 | 0.01 |
| GTTTCTTCACTGTTTAAGCT | 87574903 | 87574925 | 0.01 |
| GTTAAACAGTGAAGAAACCAT | 87574909 | 87574931 | 0.03 |
| GTGAAGAAACCATAGGAAAT | 87574916 | 87574938 | 0.02 |
| GCAGCAGCTTCCTATTTCCTA | 87574925 | 87574947 | 0.66 |
| GCTGAAAATGTATTTGAACT | 87574944 | 87574966 | 0.81 |

| | | | |
|---|---|---|---|
| GCTCTGCTGCTAGCTGGAAC | 87574978 | 87575000 | 0.35 |
| GCTAGCAGCAGAGCTAGGC | 87574987 | 87575008 | 0.01 |
| GCTAGGCAGGTTCAACCATG | 87574999 | 87575021 | 0.01 |
| GAACCATGTGGTTCTGTCTTA | 87575012 | 87575034 | 0.60 |
| GCTGTCTTAAGGATATAAGAA | 87575024 | 87575046 | 0.02 |
| GGTTGTAGAATCTCCCTCTG | 87575046 | 87575068 | 0.05 |
| GTGGCTAAGAAAAGCTGCTG | 87575065 | 87575087 | 0.01 |
| GCTGAGGCCAACCATGTGTC | 87575081 | 87575103 | 1.22 |
| GGTCACCCCTGACACATGGT | 87575088 | 87575110 | 0.13 |
| GTGTCAGGGGTGACCTTGCA | 87575096 | 87575118 | 0.00 |
| GTCTCCTTGGGTCCCCATGCA | 87575109 | 87575131 | 8.10 |
| GCTTCACACAATGGTCTCCT | 87575123 | 87575145 | 0.00 |
| GAAGCTGTGAAGGTGGAGCT | 87575141 | 87575163 | 0.05 |
| GATTGCCGAGACCCCAATGG | 87575163 | 87575185 | 0.01 |
| GTCTCCACCATTGGGGTCT | 87575168 | 87575189 | 0.01 |
| GCTTTGGTGTCTCCACCATT | 87575175 | 87575197 | 0.01 |
| GGAGACACCAAAGCTGTAGA | 87575184 | 87575206 | 0.00 |
| GCAGCAGACCCTCTACAGCTT | 87575191 | 87575213 | 1.12 |
| GCTGAGGAAAGCTGTTCACT | 87575210 | 87575232 | 0.01 |
| GGAAAGCTGTTCACTGGGAG | 87575215 | 87575237 | 0.04 |
| GCATGACTAAAAACCAGCT | 87575326 | 87575347 | 0.11 |
| GAACAGAGGTAGGAAATACT | 87575353 | 87575375 | 0.61 |
| GGAAAGAAAGGAACAGAGGT | 87575363 | 87575385 | 0.02 |
| GTCTGTTCCTTTCTTTCCTTC | 87575369 | 87575391 | 0.03 |
| GTCCTTTCTTTCCTTCTGGAA | 87575374 | 87575396 | 0.09 |
| GATATACATGACCATTCCAGA | 87575384 | 87575406 | 0.41 |
| GACATATATATATCTTGGCAC | 87575407 | 87575429 | 0.21 |
| GCTTTCTTATTTTGATTTTAC | 87575468 | 87575490 | 0.02 |
| GTTTACAGGGGGTTATAGTGA | 87575483 | 87575505 | 0.01 |
| GTTGAAACTGTTATAGATTA | 87575557 | 87575579 | 0.01 |
| GATAATGCAAAAATACATTAA | 87575595 | 87575617 | 0.06 |
| GGCTACAAGCCTACTGAGT | 87575623 | 87575644 | 0.03 |
| GCAAGCCTACTGAGTAGGAAG | 87575628 | 87575650 | 0.34 |
| GGGAATGCAGTGACTTGAAT | 87575649 | 87575671 | 0.03 |
| GCAGTGACTTGAATAGGAA | 87575655 | 87575676 | 0.01 |
| GCCTTCAAATCTATGAGCCTC | 87575680 | 87575702 | 0.01 |
| GATTTGAAGGCTTGGTTATT | 87575693 | 87575715 | 0.00 |
| GAAGGCTTGGTTATTTGGGAG | 87575699 | 87575721 | 0.01 |
| GTGGTCCTACTTGACAGATT | 87575718 | 87575740 | 0.77 |
| GACTTGACAGATTAGGAGGTG | 87575726 | 87575748 | 1.76 |
| GTTACTGAGGGTAGGATCC | 87575767 | 87575788 | 2.42 |
| GGCTTGAGCATTTGAAATCC | 87575784 | 87575806 | 0.01 |
| GAGAGCAAGACACTGGGGC | 87575805 | 87575826 | 0.58 |
| GCAGGAAGAGAGCAAGACAC | 87575811 | 87575833 | 0.03 |
| GTCATCATATTCATAGGCAGC | 87575829 | 87575851 | 0.29 |
| GAGTTCCTCATCATATTCAT | 87575836 | 87575858 | 0.01 |
| GTCTCTAGCATCGTTCCAAAC | 87575863 | 87575885 | 0.53 |
| GTAAGGAATGGGGTTCCAGTT | 87575877 | 87575899 | 0.01 |
| GGAACCCCATTCCTTATCTC | 87575884 | 87575906 | 2.21 |
| GTGGACCAGAGATAAGGAAT | 87575889 | 87575911 | 0.04 |
| GATGCATGTGGACCAGAGATA | 87575895 | 87575917 | 0.00 |
| GGATTGAGTCTGATGCATG | 87575908 | 87575929 | 0.47 |

| | | | |
|---|---|---|---|
| GCATTCACTGATTTCTGGTGA | 87575928 | 87575950 | 0.00 |
| GGGATCATTCACTGATTTC | 87575934 | 87575955 | 0.03 |
| GTCAGTGAATGATCCCTGTGT | 87575941 | 87575963 | 0.00 |
| GATGATCCCTGTGTTGGCATC | 87575948 | 87575970 | 0.21 |
| GCAGTTCCTGATGCCAACACA | 87575953 | 87575975 | 0.17 |
| GCCTGATGTGTAGGAAAAGG | 87575995 | 87576017 | 0.02 |
| GAACTTCTAGCCTGATGTGT | 87576004 | 87576026 | 1.92 |
| GTTGCCAGACATCCCAGGAA | 87576026 | 87576048 | 0.00 |
| GATCAAGTTGCCAGACATCCC | 87576031 | 87576053 | 0.03 |
| GACGTTGGACAGTGAGCATGT | 87576058 | 87576080 | 0.70 |
| GCTGGAGGTACTTGGCAACGT | 87576074 | 87576096 | 3.92 |
| GCATATGTACCTGGAGGTACT | 87576083 | 87576105 | 0.02 |
| GTACCTCCAGGTACATATGCT | 87576088 | 87576110 | 2.68 |
| GAGATCCTAGCATATGTACC | 87576093 | 87576115 | 0.18 |
| GTTATTGATAGAAGAAGTGAT | 87576119 | 87576141 | 0.01 |
| GGAACTACATACAAGACCCA | 87576387 | 87576409 | 0.18 |
| GTCTCCCAACTGGGATTCCA | 87576404 | 87576426 | 1.70 |
| GACAACTCTTGTCTCCCAACT | 87576413 | 87576435 | 1.42 |
| GCTCTACCATAAAAACCAAGC | 87576443 | 87576465 | 1.19 |
| GGGACCCAGCTTGGTTTTTA | 87576448 | 87576470 | 0.02 |
| GCACTTACTGGGGACCCAGCT | 87576457 | 87576479 | 0.04 |
| GCATACTGATGGCACTTACTG | 87576468 | 87576490 | 0.00 |
| GTTCCTCTGGATACATACTGA | 87576480 | 87576502 | 2.75 |
| GCAATTGAGACATATTTCCTC | 87576494 | 87576516 | 0.30 |
| GTTTTCTGATATCTAAAGATT | 87576517 | 87576539 | 0.01 |
| GTAAGGAAGTGAGAAAGTCAG | 87576559 | 87576581 | 0.01 |
| GCATTTATGAGGGGGATGTTA | 87576577 | 87576599 | 0.01 |
| GAAGAAAAAGGTGAAATGACA | 87576606 | 87576628 | 0.01 |
| GTTTTCTTCAGACCTTAACCT | 87576622 | 87576644 | 0.01 |
| GAGTTGGTACACCAAGGTTA | 87576633 | 87576655 | 1.13 |
| GATTAAGGAGTTGGTACACCA | 87576639 | 87576661 | 0.01 |
| GTCTGTTGACATTAAGGAGT | 87576649 | 87576671 | 0.04 |
| GACCAGGTCTGTTGACATTA | 87576655 | 87576677 | 0.00 |
| GCAGCAATTTTTTAAGTGACC | 87576671 | 87576693 | 0.01 |
| GCTGGCCACTTCAGCCTCTC | 87576690 | 87576712 | 22.54 |
| GTATCACCGGAGAGGCTGAAG | 87576695 | 87576717 | 0.41 |
| GACTTGACAGTATCACCGGAG | 87576704 | 87576726 | 0.01 |
| GGGCAGGTGTCAAAGGGTG | 87576734 | 87576755 | 0.06 |
| GAGTCAGGGCAGGTGTCAAA | 87576739 | 87576761 | 0.01 |
| GGCAACATCAGAGTCAGGGC | 87576749 | 87576771 | 0.01 |
| GTTATGGCAACATCAGAGTC | 87576754 | 87576776 | 0.01 |
| GAGGAAAGGTATGGAGTTA | 87576770 | 87576791 | 0.01 |
| GACAGGGATAGAGGAAAGGTA | 87576778 | 87576800 | 0.11 |
| GCAAAAACAGGGATAGAGGAA | 87576783 | 87576805 | 0.44 |
| GTACGTCAAAAACAGGGATAG | 87576788 | 87576810 | 0.06 |
| GCAGCTTGCTACGTCAAAAAC | 87576796 | 87576818 | 0.02 |
| GACGTAGCAAGCTGTTTTA | 87576805 | 87576826 | 0.01 |
| GCAAGCTGTTTTAAGGCCTCC | 87576812 | 87576834 | 0.51 |
| GCCTCCAGGACACGTTCTGT | 87576826 | 87576848 | 0.01 |
| GTTGTGGCTCTATTGCAGTCT | 87576861 | 87576883 | 0.02 |
| GATGAAGTATCAAGGGTTG | 87576878 | 87576899 | 1.03 |
| GTGTCAGAGATGAAGTATCA | 87576885 | 87576907 | 0.01 |

| | | | |
|---|---|---|---|
| GCTACAGTGTTTTGGACTGC | 87576913 | 87576935 | 0.01 |
| GCTGGACAGCTACAGTGTTT | 87576921 | 87576943 | 0.51 |
| GTCCAGCAGCCATTGATGAAC | 87576938 | 87576960 | 0.42 |
| GATGAACTGGGTACCTGAAT | 87576951 | 87576973 | 0.02 |
| GGGTACCTGAATGGGGGAC | 87576959 | 87576980 | 0.01 |
| GTCATTTCCAGTCCCCCATTC | 87576964 | 87576986 | 0.47 |
| GACTGGAAATGAAAACAGAC | 87576975 | 87576997 | 0.03 |
| GATTCAGCTCTGTGTTTATTT | 87577056 | 87577078 | 0.19 |
| GTTTATTTAGGGAGAGCCCA | 87577068 | 87577090 | 0.08 |
| GTTGCAAAACACACTCAGC | 87577142 | 87577163 | 0.00 |
| GCAGGTGGTCTGCACCTGT | 87577159 | 87577180 | 0.05 |
| GCTGCACCTGTAGGAAATTGC | 87577168 | 87577190 | 0.49 |
| GAAGGTGGAAGATGCCACCCT | 87577197 | 87577219 | 0.09 |
| GAAGATGCCACCCTAGGTTGG | 87577204 | 87577226 | 0.80 |
| GGTCTGCCACCAACCTAGGG | 87577210 | 87577232 | 1.67 |
| GGTTGGTGGCAGACCAACTG | 87577218 | 87577240 | 0.04 |
| GTTGTTTTTTAAAGATGAGAC | 87577303 | 87577325 | 0.01 |
| GTTTTCCATGATACCATCTA | 87577342 | 87577364 | 0.15 |
| GCTAGGCCCTAGATGGTATCA | 87577347 | 87577369 | 14.63 |
| GATACCATCTAGGGCCTAGAG | 87577352 | 87577374 | 1.93 |
| GCATGGCTGCCTAGCCACTCT | 87577365 | 87577387 | 0.01 |
| GCAGCCATGTCTGTCTTAGGC | 87577380 | 87577402 | 0.01 |
| GACGGAGCAATATAGACAC | 87577399 | 87577420 | 1.47 |
| GTCTATATTGCTCCGTCTTAC | 87577405 | 87577427 | 0.09 |
| GTTGCTCCGTCTTACTGGTCA | 87577411 | 87577433 | 0.00 |
| GTCCTCCATGACCAGTAAGA | 87577416 | 87577438 | 2.98 |
| GGAGGACAAGCACAGCTTTT | 87577432 | 87577454 | 0.00 |
| GTTGGTGTTTGTCTGTGACTT | 87577451 | 87577473 | 0.04 |
| GTCTGTGACTTAGGTCAAT | 87577460 | 87577481 | 0.54 |
| GTCTCACCTGTCCCTGACCAC | 87577495 | 87577517 | 0.01 |
| GAAGGCCGGTGGTCAGGGAC | 87577500 | 87577522 | 0.01 |
| GCTATAGAAGGCCGGTGGTC | 87577506 | 87577528 | 0.07 |
| GTATGTGCTATAGAAGGCCGG | 87577511 | 87577533 | 1.08 |
| GGGAAAGTATGTGCTATAGA | 87577518 | 87577540 | 0.01 |
| GTTCGTGGCTCTGGTCTGAAT | 87577538 | 87577560 | 0.01 |
| GTCATATGTCCTTCGTGGCTC | 87577548 | 87577570 | 0.01 |
| GATATCAATGAACAACTGCCA | 87577594 | 87577616 | 0.24 |
| GTTGGTTGTCTTCACACAGGA | 87577640 | 87577662 | 0.02 |
| GACCAATCTGTTTAAAATATG | 87577658 | 87577680 | 0.01 |
| GATCTCATTGCTTTTAACTTT | 87577682 | 87577704 | 0.02 |
| GAATGAGATTAAAGTTGTCTG | 87577697 | 87577719 | 0.01 |
| GTTGTCTGTGGGAATGTCCC | 87577709 | 87577731 | 0.12 |
| GTAGCAGCTGAATTGAACACC | 87577727 | 87577749 | 0.00 |
| GTTCAATTCAGCTGCTAAT | 87577733 | 87577754 | 4.19 |
| GCCACCAGGTTTATGGGGGG | 87577777 | 87577799 | 0.04 |
| GAAAACACTTGTGCTCCAGT | 87577815 | 87577837 | 0.02 |
| GCACTTGTGCTCCAGTAGGAG | 87577820 | 87577842 | 0.00 |
| GCATAGTCTCCACTCCTAC | 87577830 | 87577851 | 0.01 |
| GGAGTGGAGACTATGCTCC | 87577836 | 87577857 | 0.01 |
| GATGCTCCAGGCTAACTCAGC | 87577848 | 87577870 | 0.00 |
| GGCTAACTCAGCTGGCTGGT | 87577856 | 87577878 | 0.03 |
| GAAATTTTCACAAAAAGAAAA | 87577975 | 87577997 | 0.01 |

| | | | |
|---|---|---|---|
| GTTTTTGCTTTTTGTTTTTCG | 87578101 | 87578123 | 0.01 |
| GCTTTTTGTTTTTCGAGGCA | 87578106 | 87578128 | 0.02 |
| GTGGGGTTCCAGGATAGCCA | 87578142 | 87578164 | 0.14 |
| GATCTACAGAGTGGGGTTCC | 87578152 | 87578174 | 0.01 |
| GCAAAAAGGACTGGGCAGTGG | 87578241 | 87578263 | 0.01 |
| GACAAAAAACCAAAAAGGACT | 87578250 | 87578272 | 0.02 |
| GCAGGACCTTCGGAGAGCAGT | 87578405 | 87578427 | 0.05 |
| GAGCACCGACTGCTCTCCGA | 87578410 | 87578432 | 0.03 |
| GAAAAAACCAAAAAGGGGGTC | 87578453 | 87578475 | 0.02 |
| GAACAAAAAAAACCAAAAAGG | 87578458 | 87578480 | 0.49 |
| GAGATTCTTTGTAATTCTCA | 87578529 | 87578551 | 0.01 |
| GTACAAAGAATCTCAATAATA | 87578539 | 87578561 | 0.04 |
| GTAAGCATTTGAGATGTTTTG | 87578577 | 87578599 | 0.01 |
| GAATGCTTAACTGCAATAGCC | 87578592 | 87578614 | 0.01 |
| GTACATTTGCAATGTTTGATT | 87578793 | 87578815 | 0.01 |
| GATTAGGTCTAAATCCTCG | 87578809 | 87578830 | 0.05 |
| GAGGATTAACACTATTATG | 87578827 | 87578848 | 0.02 |
| GTAGAGAATGTGAAAGTCAAA | 87578887 | 87578909 | 0.02 |
| GTCTCTACAAATACCAAATTT | 87578904 | 87578926 | 0.01 |
| GAAATACCAAATTTTGGTCTC | 87578911 | 87578933 | 0.02 |
| GAATAGCCTGAGACCAAAATT | 87578916 | 87578938 | 0.00 |
| GTATATATATAGGCCTTACAC | 87578983 | 87579005 | 0.04 |
| GTATATATTTGTATAGCTGCC | 87579028 | 87579050 | 0.00 |
| GAACTCATATTAGATTGCC | 87579089 | 87579110 | 0.16 |
| GATGAGTTCTTAAGTGTCCAA | 87579103 | 87579125 | 0.00 |
| GTTTGAAATTGCTCCTTTTTT | 87579207 | 87579229 | 0.01 |
| GTTTCAACATTTTAAACACAG | 87579287 | 87579309 | 0.02 |
| GCTGAAGTGGAGGGAAGCTCA | 87579337 | 87579359 | 0.01 |
| GGGAGAGAAACATATGTAAT | 87579375 | 87579397 | 0.02 |
| GCTTATAAACAGCTCATCTAT | 87579395 | 87579417 | 0.02 |
| GGATGCATGCATAACTTTGC | 87579440 | 87579462 | 0.02 |
| GAATTGTTACAACTTGTCTTT | 87579489 | 87579511 | 0.00 |
| GGATAATGAGTATCAGTTT | 87579510 | 87579531 | 0.01 |
| GAATACTGATATTTATTACA | 87579544 | 87579566 | 0.04 |
| GATAAGGAATAAATGTTACAT | 87579596 | 87579618 | 0.05 |
| GCACAGAAACAGCTTCATAAT | 87579678 | 87579700 | 0.01 |
| GTAGGATGTTAAAACCTTACT | 87579697 | 87579719 | 2.01 |
| GTCTTTTTTCATCTCCAAGTA | 87579710 | 87579732 | 0.01 |
| GAAAAGATGAGTCCACATTAA | 87579727 | 87579749 | 3.51 |
| GACAGCAAATCTTTGGCAAAA | 87579751 | 87579773 | 0.01 |
| GAAGTGCCCACAGCAAATCTT | 87579759 | 87579781 | 0.01 |
| GGGACAAAAGCTCAGGCAAT | 87579803 | 87579825 | 0.01 |
| GCCTGAGCTTTTGTCCCCAC | 87579809 | 87579831 | 0.01 |
| GCAGCATCATTTTTCCGGTTC | 87579904 | 87579926 | 0.00 |
| GTATAAGCAGCATCATTTTTC | 87579910 | 87579932 | 0.01 |
| GATGCTGCTTATATAGCCCGC | 87579921 | 87579943 | 0.01 |
| GCGGCGTAGTAATGAGGTGA | 87579995 | 87580017 | 0.01 |
| GATCAACTGTTGGCTGTTG | 87580159 | 87580180 | 0.01 |
| GATTATATATAGATCAACTGT | 87580168 | 87580190 | 0.01 |
| GATATATAATACATCTGTTGT | 87580182 | 87580204 | 0.00 |
| GCAATGGGGATGCAAATTAAC | 87580246 | 87580268 | 0.02 |
| GTCTGTTTGATATTCTCAATG | 87580261 | 87580283 | 0.06 |

| | | | |
|---|---|---|---|
| GCATCTTAAGCTCACCATAGG | 87580293 | 87580315 | 0.01 |
| GTCACACTTTTTTCTTTTTT | 87580401 | 87580423 | 0.01 |
| GTCTTTTTTAGGATATTAGTG | 87580413 | 87580435 | 1.63 |
| GACAATGGGTTTTTAAATTTT | 87580491 | 87580513 | 0.02 |
| GAAGCATTTGCTCTTATACAA | 87580507 | 87580529 | 0.03 |
| GTATAGTAAAACCCCTTTAGT | 87580529 | 87580551 | 0.03 |
| GTTAGCTGATCCTACTAAAG | 87580539 | 87580561 | 1.04 |
| GCTGCTTCAACACAAGAAGCC | 87580609 | 87580631 | 0.39 |
| GCATTTTTTTTTCATAATGTA | 87580635 | 87580657 | 0.02 |
| GGCTTTTAGCCAATCCTTG | 87580656 | 87580677 | 1.62 |
| GAAAGTTTTGCCTCAAGGAT | 87580665 | 87580687 | 0.01 |
| GTTTCTAATGATATTGCTTCA | 87580684 | 87580706 | 0.01 |
| GTTACACTCACCAGAATGCAA | 87580744 | 87580766 | 0.00 |
| GTATAGAAACTCTCCTCCAAC | 87580772 | 87580794 | 0.01 |
| GCCTAGAAAGTGTCTACCGGT | 87580787 | 87580809 | 0.03 |
| GGTAGACACTTTCTAGGGGC | 87580793 | 87580815 | 0.02 |
| GTCAGTCTGACAAAGACGAA | 87580815 | 87580837 | 0.00 |
| GTAACGTGGTTAAACTGCCAG | 87580854 | 87580876 | 1.84 |
| GAGTAACGTGAAGATTAACG | 87580869 | 87580891 | 0.00 |
| GTTACTCTTGTAACACCAGAG | 87580886 | 87580908 | 0.01 |
| GCCAAAGTTGTGGTTCCGCTC | 87580900 | 87580922 | 0.01 |
| GATACACTTGGTGTTGATTT | 87580957 | 87580979 | 0.01 |
| GCATGGGGGGGTGATACACT | 87580969 | 87580991 | 0.41 |
| GCCAAAAACTCTGTAGCATG | 87580984 | 87581006 | 0.01 |
| GTTGGCTGCCAGTTCCTGTGC | 87581002 | 87581024 | 0.00 |
| GAACAATCCTGCACAGGAAC | 87581009 | 87581031 | 0.26 |
| GAGGCTGAACAATCCTGCAC | 87581015 | 87581037 | 0.01 |
| GAGACACTGCCCTAAATCC | 87581050 | 87581071 | 0.11 |
| GTAATTCTTACCAGGATTTA | 87581059 | 87581081 | 0.01 |
| GTATTTTATGTAATTCTTACC | 87581067 | 87581089 | 0.02 |
| GAGTTATTTTAAACAACAGG | 87581143 | 87581165 | 0.02 |
| GCCAAACTGGTAACTCAGGGT | 87581165 | 87581187 | 0.03 |
| GCTGGGCTCGAACTCAGAAAC | 87581335 | 87581357 | 0.01 |
| GAACTGGTAACTTGCCGGGCA | 87581401 | 87581423 | 0.01 |
| GCTTAAAACTGGTAACTTGC | 87581407 | 87581429 | 0.01 |
| GCAATTAAAAACTTGTAACTT | 87581444 | 87581466 | 0.01 |
| GACATCTTGGCTATAGGGTAT | 87581485 | 87581507 | 0.01 |
| GTTCTGCACATCTTGGCTAT | 87581492 | 87581514 | 0.02 |
| GTTTTTTCTGACTGGTTTTAA | 87581524 | 87581546 | 0.01 |
| GTCAGAAAAAAAAAATGAAG | 87581536 | 87581558 | 0.03 |
| GAAAGAATAAGAAAAATTTT | 87581585 | 87581607 | 0.01 |
| GGAACACCTTGAGCCAGGTG | 87581627 | 87581649 | 0.01 |
| GACACCTTGAGCCAGGTGTGG | 87581630 | 87581652 | 0.01 |
| GCTCCTTTAGTCCCAGCTATC | 87581657 | 87581679 | 0.01 |
| GTTGTGCTTATTGGGTTCAGA | 87581927 | 87581949 | 0.19 |
| GTTGCAGAGAATTTTTTTTCT | 87581950 | 87581972 | 0.05 |
| GGAGAAAACTTGCTTTTAAA | 87582022 | 87582044 | 0.03 |
| GAAGTTGCCCTGCACTGTAGC | 87582060 | 87582082 | 0.00 |
| GTTTAGTTTAACTTATTTAGA | 87582112 | 87582134 | 0.02 |
| GAAATAAGTTAAACTAAATCA | 87582118 | 87582140 | 0.02 |
| GAAAAACAGACATGAATCGAC | 87582223 | 87582245 | 0.00 |
| GTGGACAGATTGATGCACCA | 87582245 | 87582267 | 0.04 |

| | | | |
|---|---|---|---|
| GAACTAAGGATATCTCCAGC | 87582309 | 87582331 | 0.03 |
| GCTTCCTCTTTGGCTCCTGC | 87582324 | 87582346 | 0.22 |
| GCAGGAGCCAAAGAGGAAGC | 87582327 | 87582349 | 0.14 |
| GCCTAGGGAAAGGAAGAAATC | 87582356 | 87582378 | 0.02 |
| GTAATCAAAAGAGAAGGAAAA | 87582392 | 87582414 | 0.02 |
| GGACGTGTAATCAAAAGAGA | 87582399 | 87582421 | 0.01 |
| GTTTGATTACACGTCCCAGAT | 87582407 | 87582429 | 0.01 |
| GTACACGTCCCAGATAGGAGA | 87582413 | 87582435 | 0.12 |
| GTCAGCTACCATCTCCTATCT | 87582420 | 87582442 | 0.01 |
| GATGGTAGCTGAAAATGACCA | 87582432 | 87582454 | 0.02 |
| GAAGGACTGGAGACATTGTGC | 87582545 | 87582567 | 0.01 |
| GTAAGGGACAGACTTAAGGAC | 87582559 | 87582581 | 0.04 |
| GACATTAAGGGACAGACTTA | 87582564 | 87582586 | 0.07 |
| GCTGTCCCTTAATGTCACTAA | 87582572 | 87582594 | 0.02 |
| GTTAGCCTTTAGTGACATTA | 87582577 | 87582599 | 0.01 |
| GCCCCCCCCCCCCAAATGAA | 87582612 | 87582634 | 0.15 |
| GCACACACCTTTCATTTGGGG | 87582618 | 87582640 | 1.53 |
| GGTGTGTGATTTAGCTACTC | 87582633 | 87582655 | 0.20 |
| GTTGCTTCTATCCCGATTTC | 87582656 | 87582678 | 0.03 |
| GCCTCTGTGTCTCTGTGAGT | 87582731 | 87582753 | 0.03 |
| GTCTCTGTGAGTAGGACATTC | 87582740 | 87582762 | 0.01 |
| GTAGGACATTCTGGACGTA | 87582749 | 87582770 | 0.01 |
| GATTCTGGACGTAAGGCTTTT | 87582756 | 87582778 | 0.02 |
| GATGCATTGGGCGAGAGGAGC | 87582857 | 87582879 | 0.23 |
| GCAAGAGATGCATTGGGCGAG | 87582863 | 87582885 | 0.41 |
| GCCGCAGCCAAGAGATGCATT | 87582870 | 87582892 | 0.34 |
| GTGACAGTGCATTGGGATGG | 87582908 | 87582930 | 0.00 |
| GCTCAGGGGTGACAGTGCATT | 87582915 | 87582937 | 0.02 |
| GCTGTCACCCCTGAGTGAATT | 87582924 | 87582946 | 0.01 |
| GCCAACACCGAATTCACTCAG | 87582930 | 87582952 | 0.01 |
| GGATACAACTAGGCTGAGT | 87582960 | 87582981 | 0.39 |
| GTTGCAAAGAGGATACAACT | 87582969 | 87582991 | 0.01 |
| GGAGACTGTTGTTGCAAAG | 87582980 | 87583001 | 0.72 |
| GTCTCCAAAAAATAAAACTTA | 87582997 | 87583019 | 0.08 |
| GATAAAACTTATGGCTAGTGT | 87583007 | 87583029 | 0.01 |
| GTTTGCTGCTTACCACCGTGC | 87583041 | 87583063 | 0.01 |
| GTTGCAGTATCCTGCACGG | 87583052 | 87583073 | 0.01 |
| GCAACTTCTTTCATTTTCTG | 87583069 | 87583091 | 0.01 |
| GCTGTGGAGCTCCACCAAGAC | 87583086 | 87583108 | 0.01 |
| GAGGGTGTAACCTGTCTTGG | 87583096 | 87583118 | 0.00 |
| GGTTGTGGGGGATGTGAGGA | 87583114 | 87583136 | 0.02 |
| GCTCACATCCCCCACAACCCC | 87583119 | 87583141 | 0.01 |
| GATTGAACCCGGGGGTTGTG | 87583127 | 87583149 | 0.01 |
| GAATAAGAGGATTGAACCCGG | 87583135 | 87583157 | 0.01 |
| GACAGGCAGTGCCCAAATAAG | 87583149 | 87583171 | 0.01 |
| GTGGCTGCTAGACATCTAC | 87583167 | 87583188 | 1.26 |
| GTCTAGCAGCCACAGAGAAC | 87583176 | 87583198 | 0.01 |
| GTTCAGGTATCCAGTTCTCTG | 87583185 | 87583207 | 0.08 |
| GTCATATTTAGCCTCCCTTTC | 87583202 | 87583224 | 0.01 |
| GGCTAAATATGAAAAAGGAC | 87583213 | 87583235 | 0.02 |
| GAAGGACAGGTGAGCAAGAGA | 87583227 | 87583249 | 0.01 |
| GAATATGAAAATTTGAGCCG | 87583485 | 87583507 | 0.01 |

| | | | |
|---|---|---|---|
| GAGAGAGAGGATGGGTCTGT | 87583532 | 87583554 | 0.01 |
| GAGACAGTTCCAGCACAGCC | 87583602 | 87583624 | 0.01 |
| GACTGTCTCCGTTGACCAGGC | 87583617 | 87583639 | 0.01 |
| GGTCTGGGGCCGGGCAGTGG | 87583699 | 87583721 | 0.04 |
| GCAAAGGATGGGTCTGGGGCC | 87583708 | 87583730 | 0.01 |
| GACATAATCCTGCTGAAACAA | 87583725 | 87583747 | 0.00 |
| GCAGGCATTCTGCTCCTGT | 87583763 | 87583784 | 0.04 |
| GCAGTACCTGCAATTTCCTAC | 87583777 | 87583799 | 0.02 |
| GGAAATTGCAGGTACTGCA | 87583783 | 87583804 | 0.43 |
| GGCAGAAGACTCCACCTTGT | 87583803 | 87583825 | 0.02 |
| GACTCCACCTTGTGGGGCTGA | 87583811 | 87583833 | 0.50 |
| GTGGGGCTGAAGGAGTGTTG | 87583821 | 87583843 | 0.01 |
| GCTGAAGGAGTGTTGGGGGG | 87583826 | 87583848 | 0.01 |
| GGAGTGTTGGGGGGTGGGGG | 87583832 | 87583854 | 0.01 |
| GAGGGACTCTGGCTGGACGT | 87583860 | 87583882 | 0.02 |
| GTCCCTCCTGTGCTCTGGGC | 87583876 | 87583898 | 0.10 |
| GATCCCAGATGCTTTCCACT | 87583912 | 87583934 | 1.05 |
| GTTCCACTTGGCCCCACTCGA | 87583925 | 87583947 | 0.00 |
| GTTGGCCCCACTCGACGGAGG | 87583931 | 87583953 | 0.02 |
| GCACTCGACGGAGGTGGGAGG | 87583938 | 87583960 | 0.01 |
| GGAGGTGGGAGGGGGGACAA | 87583946 | 87583968 | 0.07 |
| GCCCCCATACCAGGGCCCCG | 87583972 | 87583994 | 0.44 |
| GGGCCCCGGGGCTACACCCT | 87583984 | 87584006 | 0.01 |
| GTGCCAAGGGTGTAGCCCCG | 87583987 | 87584009 | 0.01 |
| GCACCCTTGGCACAGGAGATA | 87583998 | 87584020 | 0.01 |
| GCACAGGAGATACGGGAGAG | 87584006 | 87584028 | 0.01 |
| GATACGGGAGAGAGGGTGGA | 87584014 | 87584036 | 0.02 |
| GAGAGAGGGTGGAGGGAGAG | 87584021 | 87584043 | 0.04 |
| GTCCTTAGTCGGGTCTGTGGC | 87584062 | 87584084 | 0.58 |
| GAGCACAGGAAGGCATTCTG | 87584084 | 87584106 | 0.05 |
| GCCTATCCCATCGTCCAATA | 87584131 | 87584153 | 0.01 |
| GCTAAAGCCATATTGGACGAT | 87584137 | 87584159 | 0.01 |
| GATAAAGCTCTAAAGCCATAT | 87584145 | 87584167 | 0.01 |
| GACCCACCATGGCCAAGAGG | 87584245 | 87584267 | 0.01 |
| GGGAAAGGAGAGAGAGAAGA | 87584273 | 87584295 | 0.06 |
| GAGAGAGAGAAGAAGGTAAG | 87584280 | 87584302 | 0.01 |
| GGTAAGAGGTGAGAGAGAGA | 87584294 | 87584316 | 0.05 |
| GAGGTGAGAGAGAGAGGGGG | 87584299 | 87584321 | 0.07 |
| GTTGAGAGTAAGAGAGTGAG | 87584323 | 87584345 | 0.04 |
| GATAACAGTCTACCATAAGA | 87584394 | 87584416 | 0.03 |
| GTAGCCACAGCTTCTCCTGT | 87584483 | 87584505 | 0.08 |
| GTCAACAGAAGCCAAGTGTCC | 87584525 | 87584547 | 0.01 |
| GCCTCATGTCTCCTGGACACT | 87584535 | 87584557 | 0.01 |
| GACCATTCACCTACATGGGA | 87584567 | 87584589 | 0.06 |
| GTGGTGACCATTCACCTACA | 87584572 | 87584594 | 0.01 |
| GGTCACCACTGGGTTTCCCC | 87584586 | 87584608 | 0.07 |
| GGAACCCCGGGGAAACCCAG | 87584591 | 87584613 | 0.05 |
| GGCCAGGTGTGGGGGGCAAT | 87584654 | 87584676 | 0.01 |
| GTAAAGGTGGGCCAGGTGTG | 87584663 | 87584685 | 0.03 |
| GCATGGAGGTAAAGGTGGGCC | 87584670 | 87584692 | 0.38 |
| GAATCTCATGGAGGTAAAGGT | 87584675 | 87584697 | 0.05 |
| GCTGTGACGTAATCTCATGG | 87584685 | 87584707 | 1.40 |

| | | | |
|---|---|---|---|
| GAAAGAGTGAAGGTCAGGAG | 87584724 | 87584746 | 0.01 |
| GAAAGAGAAAGAGTGAAGGTC | 87584729 | 87584751 | 0.04 |
| GGGGGAAAGAGAAAGAGTGA | 87584734 | 87584756 | 0.05 |
| GGGGGAAATGAAGATGAAGG | 87584752 | 87584774 | 0.01 |
| GTGGGGGGAAAGGGCAAAAG | 87584771 | 87584793 | 0.55 |
| GTCTCTCCTGTGGAAGTGTAC | 87584802 | 87584824 | 0.15 |
| GCCAGGCCAGTACACTTCCAC | 87584807 | 87584829 | 0.01 |
| GTCGTCGAAAAAGATCACACC | 87584825 | 87584847 | 0.01 |
| GGGTGCTGTGTTAGAATTG | 87584849 | 87584870 | 0.01 |
| GCACAGCACCCTGAGTGCTCT | 87584861 | 87584883 | 0.01 |
| GACCCTGAGTGCTCTTGGAGG | 87584867 | 87584889 | 0.02 |
| GTCCTCCTGACCCTTTCCCTT | 87584897 | 87584919 | 0.01 |
| GCCTCATCTTCCTAAGGGAAA | 87584906 | 87584928 | 0.00 |
| GAAGCCTACCTCATCTTCCTA | 87584913 | 87584935 | 0.01 |
| GTAGGCTTTGTTTTTTACCT | 87584928 | 87584950 | 0.02 |
| GTTACCTTGGTTTTCACCTTT | 87584942 | 87584964 | 0.01 |
| GACTTTCAAATAAAGCCAAA | 87584957 | 87584979 | 0.02 |
| GTATTTGAAAGTCCTGGTACC | 87584968 | 87584990 | 0.01 |
| GGAGACGGGGACCTGGTACC | 87584979 | 87585001 | 0.01 |
| GCTGTAAAGGCCAAGGAGAC | 87584993 | 87585015 | 0.01 |
| GGCCAGAGCTGTAAAGGCCA | 87585000 | 87585022 | 0.01 |
| GATTGGGGGCCAGAGCTGTAA | 87585006 | 87585028 | 0.91 |
| GCTCTGGCCCCCAATCCTGTG | 87585015 | 87585037 | 0.00 |
| GTTTATTGCCTCACAGGATT | 87585023 | 87585045 | 0.01 |
| GTTGATGGTTTATTGCCTCAC | 87585029 | 87585051 | 0.07 |
| GAGGAAGGCACAAGCATTGA | 87585045 | 87585067 | 0.00 |
| GATGCTTGTGCCTTCCTCGTT | 87585051 | 87585073 | 0.31 |
| GGACCCAGGGGGACCAAACG | 87585064 | 87585086 | 0.07 |
| GTTTGGTCCCCCTGGGTCCTA | 87585069 | 87585091 | 0.05 |
| GAAGGTGTCCTTAGGACCCAG | 87585076 | 87585098 | 0.16 |
| GCTGAAGTCTAAGGTGTCCTT | 87585085 | 87585107 | 0.66 |
| GACACCTTAGACTTCAGGCCT | 87585092 | 87585114 | 0.01 |
| GCCATGGATGAGGGAAGTCCA | 87585109 | 87585131 | 0.01 |
| GCACGCAGTTCCCATGGATG | 87585120 | 87585142 | 0.12 |
| GATGATGCACGCAGTTCCCA | 87585126 | 87585148 | 0.01 |
| GTACTCCAAGAAGTGGAGTG | 87585154 | 87585176 | 0.01 |
| GTCAGGGGTACTCCAAGAAG | 87585161 | 87585183 | 0.01 |
| GCACTACGTAGAGAGGTCAG | 87585176 | 87585198 | 0.00 |
| GAAATTAGGCACTACGTAGAG | 87585183 | 87585205 | 0.01 |
| GCATTTAGAGGCCCATAAATT | 87585198 | 87585220 | 0.01 |
| GCAGAGGTGGATACATTTAG | 87585211 | 87585233 | 0.01 |
| GAGACCATATTTGACTGCAG | 87585227 | 87585249 | 0.01 |
| GACATTTGAATTGCTTTCTAA | 87585255 | 87585277 | 0.01 |
| GTGGCTGAGCGCTCGAGCGA | 87585276 | 87585298 | 0.00 |
| GCGCTCGAGCGATGGGGTTT | 87585284 | 87585306 | 0.01 |
| GCGATGGGGTTTGGGGAGAG | 87585292 | 87585314 | 0.01 |
| GGGTTTGGGGAGAGTGGGAC | 87585298 | 87585320 | 0.05 |
| GAGTGGGACAGGATGGAAA | 87585309 | 87585330 | 0.50 |
| GAAAGACACAGGAACGGAACG | 87585334 | 87585356 | 0.01 |
| GAGGCAAAAAGACACAGGAA | 87585341 | 87585363 | 1.26 |
| GTTGAAGAGGCAAAAAGACAC | 87585346 | 87585368 | 0.01 |
| GAATACGATCAGGGCTTGAAG | 87585360 | 87585382 | 0.01 |

| | | | |
|---|---|---|---|
| GCAGACAAAAAATACGATCA | 87585370 | 87585392 | 0.02 |
| GTTATTTACACAGTGTCAGCT | 87585396 | 87585418 | 0.01 |
| GCTTTGCAACAAATGTAGGGC | 87585422 | 87585444 | 0.01 |
| GAGACAGAGACAGACCATAG | 87585448 | 87585470 | 0.01 |
| GAGACAGACCATAGAGGAGC | 87585454 | 87585476 | 0.01 |
| GACCATAGAGGAGCGGGGCTG | 87585461 | 87585483 | 0.01 |
| GGAGCGGGGCTGCGGTGAG | 87585469 | 87585490 | 0.01 |
| GGGCTGCGGTGAGAGGAGCA | 87585475 | 87585497 | 0.02 |
| GGTGAGAGGAGCAAGGCAGC | 87585482 | 87585504 | 0.03 |
| GGCTGCAGCCTGCACACAC | 87585507 | 87585528 | 0.36 |
| GCAGTGTGTGCCGGTGTGTGC | 87585515 | 87585537 | 0.02 |
| GGTGTGTGCCAGTGTGTGC | 87585525 | 87585546 | 1.26 |
| GTAGCTTGCTGAGGGGAGAGC | 87585545 | 87585567 | 0.00 |
| GCAGCAGCTTAGCTTGCTGAG | 87585553 | 87585575 | 0.01 |
| GCTGCTGATCTAACCTGTCC | 87585569 | 87585591 | 0.00 |
| GATCCAGATGGACCCTGGAC | 87585582 | 87585604 | 0.01 |
| GCAGATGATCCAGATGGACCC | 87585587 | 87585609 | 1.16 |
| GACACTCACAGATGATCCAGA | 87585594 | 87585616 | 0.00 |
| GTGATTCCTGCTGTGCCTC | 87585615 | 87585636 | 0.01 |
| GTGGGTGCCTGAGGCACAGC | 87585621 | 87585643 | 2.49 |
| GAAGTCCACGTGGGTGCCTG | 87585630 | 87585652 | 6.50 |
| GCAGAAGGGAGAAGTCCACG | 87585640 | 87585662 | 1.01 |
| GACTTCTCCCTTCTGCCCTCC | 87585648 | 87585670 | 0.01 |
| GAGACACCCAGGAGGGCAGA | 87585655 | 87585677 | 0.01 |
| GAAGCCAGAGACACCCAGGA | 87585662 | 87585684 | 0.89 |
| GCAGCGTCATCTCTCCCTCT | 87585698 | 87585720 | 0.03 |
| GTGTTCTTGCAAGCTCCAAG | 87585713 | 87585735 | 0.45 |
| GCTTGCAAGAACACACCTGTG | 87585723 | 87585745 | 0.06 |
| GCACAAAATGGAACACCACAC | 87585737 | 87585759 | 0.57 |
| GAGAGAGGGCGTCACAAAA | 87585750 | 87585771 | 0.01 |
| GAGTGTGTGGAGGAAGAGAG | 87585764 | 87585786 | 0.01 |
| GAATGAAATCTGAGTGTGTGG | 87585774 | 87585796 | 0.13 |
| GCAGATTTCATTAGTACAGCT | 87585786 | 87585808 | 0.01 |
| GTTGGCCTGAGTTCAGAGCAA | 87585805 | 87585827 | 0.01 |
| GCAGAGCAATGGCCAAAATAT | 87585817 | 87585839 | 0.01 |
| GGCCAAAATATTGGCAAATT | 87585826 | 87585848 | 0.00 |
| GTTATGATCTGTCAGAATT | 87585852 | 87585873 | 0.02 |
| GATCATAACAACAGCTACTG | 87585865 | 87585887 | 0.09 |
| GTTTATATATAAGATATTTG | 87585897 | 87585919 | 0.05 |
| GAATTAGAGAAAATATAGGG | 87585925 | 87585947 | 0.02 |
| GATTAATAAAAGAACAGGAGT | 87585947 | 87585969 | 0.00 |
| GAAATCAATTAATAAAAGAAC | 87585953 | 87585975 | 0.02 |
| GAGGTTGTGATTGGTACGTA | 87585989 | 87586011 | 2.81 |
| GTAGTGGTGAGAGGTTGTGAT | 87585998 | 87586020 | 0.06 |
| GAGGAGGGGTTAGTGGTGAG | 87586008 | 87586030 | 0.14 |
| GGGGGGAGAGGAGGGGTTAG | 87586015 | 87586037 | 0.04 |
| GTGGGCCTGGGGGGAGAGGA | 87586023 | 87586045 | 0.12 |
| GATTGTGAGGGTGGGCCTGG | 87586033 | 87586055 | 0.30 |
| GGGGAGGGGATTGTGAGGGT | 87586041 | 87586063 | 0.04 |
| GTAAGGGGGAGGGGATTGTG | 87586046 | 87586068 | 0.01 |
| GAGGTGGGAGTAAGGGGGAG | 87586055 | 87586077 | 0.02 |
| GCTGAAGAGGTGGGAGTAAGG | 87586060 | 87586082 | 0.02 |

| | | | |
|---|---|---|---|
| GTACTCCCACCTCTTCAGAGA | 87586066 | 87586088 | 0.01 |
| GAAGGGGAAGCCTCCCCCTT | 87586084 | 87586106 | 0.01 |
| GTAGGTTGGTACCCAAGGGGG | 87586094 | 87586116 | 0.03 |
| GCAATTTTATGTGCCAGGGT | 87586113 | 87586135 | 0.01 |
| GACCCTGGCACATAAAATTGC | 87586116 | 87586138 | 0.01 |
| GTAAAATTGCAGGAGAACTAA | 87586127 | 87586149 | 0.00 |
| GTTGTCTGGTCTCAGAGGGAG | 87586153 | 87586175 | 0.03 |
| GTGGGGGGCTGCTCTCCTGC | 87586182 | 87586204 | 0.64 |
| GAAAACATGTGGTCTTCATGT | 87586221 | 87586243 | 0.46 |
| GAAGACCACATGTTTTGTTC | 87586228 | 87586250 | 0.01 |
| GTTCACCCTGAACAAAACATG | 87586233 | 87586255 | 0.01 |
| GAGAAAAACAATTTCAGATA | 87586260 | 87586282 | 0.00 |
| GCAGATATGGAAACTTAAAAA | 87586274 | 87586296 | 0.01 |
| GCTCAGGGAGGCGACCAGCC | 87586315 | 87586337 | 0.02 |
| GATGAGGTTCAGTTCCCAGGC | 87586329 | 87586351 | 0.62 |
| GGAACTGAACCTCATCCCTG | 87586337 | 87586359 | 1.10 |
| GCCATCTCCCCTCAGGGATG | 87586346 | 87586368 | 0.03 |
| GATGTTGTGCCATCTCCCCTC | 87586353 | 87586375 | 0.08 |
| GATGGCACAACATGTTTGA | 87586363 | 87586384 | 0.01 |
| GAAGGATGAGAACACAAATCG | 87586381 | 87586403 | 0.01 |
| GCACAAATCGAGGAGAAGCTG | 87586392 | 87586414 | 0.15 |
| GGAGAAGCTGCGGGGAGCTG | 87586402 | 87586424 | 3.13 |
| GCCATGACGTCAAAATACAAT | 87586431 | 87586453 | 1.14 |
| GACGTCATGGATTAAGCAAG | 87586444 | 87586466 | 0.04 |
| GTTAAGCAAGGGGGTACCCGT | 87586455 | 87586477 | 22.18 |
| GAAGGGGGTACCCGTTGGAGA | 87586461 | 87586483 | 0.56 |
| GCCCTTGAAGCCATCTCCAA | 87586471 | 87586493 | 0.00 |
| GAGATGGCTTCAAGGGCACC | 87586477 | 87586499 | 0.34 |
| GTTCAAGGGCACCTGGCTTCA | 87586485 | 87586507 | 0.01 |
| GAATTAAGGGCCTTGAAGCC | 87586495 | 87586517 | 0.01 |
| GAATTCATTCTCCTAGACATC | 87586513 | 87586535 | 0.02 |
| GCTCCTAGACATCAGGTCCAA | 87586521 | 87586543 | 0.01 |
| GTCATCACTCTGAGCTCCCTT | 87586537 | 87586559 | 1.22 |
| GTCAGAGTGATGAGGGGACAG | 87586548 | 87586570 | 0.01 |
| GTGATGAGGGGACAGAGGAG | 87586553 | 87586575 | 0.01 |
| GGGACAGAGGAGAGGGTCAG | 87586561 | 87586583 | 0.19 |
| GTCAGTGGCATGCAGTTAACT | 87586577 | 87586599 | 0.05 |
| GCAGTTAACTAGGGCAGAAC | 87586587 | 87586609 | 8.86 |
| GACTAGGGCAGAACAGGCGAT | 87586594 | 87586616 | 0.04 |
| GTTTTTACAACTTATGTACCG | 87586624 | 87586646 | 0.06 |
| GAATTGTCGTTATCTGAACCA | 87586641 | 87586663 | 0.47 |
| GTATTAACTGGTAAAGAATAT | 87586664 | 87586686 | 0.05 |
| GTTTCTTAATCATCTATTAAC | 87586677 | 87586699 | 0.04 |
| GTAGATGATTAAGAAAACATT | 87586685 | 87586707 | 0.01 |
| GTAAGAAAACATTTGGAGAAC | 87586693 | 87586715 | 0.01 |
| GAACATTTGGAGAACTGGGAG | 87586699 | 87586721 | 0.02 |
| GAGGTGTTCATTCCTAGGCC | 87586718 | 87586740 | 0.02 |
| GAAGTTCATGTTCCCGGGCCT | 87586730 | 87586752 | 0.01 |
| GTCAGACAAGTTCATGTTCCC | 87586736 | 87586758 | 0.70 |
| GTCTGAGCCTTCCAGCAAGA | 87586753 | 87586775 | 0.08 |
| GAAGTTGTCTCTGAGATGGC | 87586777 | 87586799 | 0.01 |
| GTCTCTGAGATGGCTGGACTC | 87586784 | 87586806 | 0.93 |

| | | | |
|---|---|---|---|
| GAACAGAAGCCGTTCAGCTCT | 87586818 | 87586840 | 0.02 |
| GGACGTCCCTAGAGCTGAA | 87586826 | 87586847 | 0.01 |
| GGGACGTCCTCAGCTCCCCT | 87586839 | 87586861 | 0.03 |
| GTCCTCAGCTCCCCTGGGGCC | 87586845 | 87586867 | 0.14 |
| GAAAAGGTCCCAGGCCCCAG | 87586854 | 87586876 | 0.00 |
| GGGAGAAAGGAAAAGGTCCC | 87586863 | 87586885 | 0.02 |
| GAATCATAGGGAGAAAGGAAA | 87586870 | 87586892 | 2.20 |
| GCCATTTAATCATAGGGAGAA | 87586876 | 87586898 | 0.01 |
| GAATGGAGTCTGAAAACAAAC | 87586894 | 87586916 | 0.01 |
| GAAGCAAAGGAAACTAAAAAA | 87586917 | 87586939 | 0.01 |
| GCTTGGTTTGGAAACAAGCAA | 87586931 | 87586953 | 0.01 |
| GCAGAGGTGTAGCTTGGTT | 87586944 | 87586965 | 0.01 |
| GATCTTGCACATTTGTAGCAG | 87586959 | 87586981 | 0.01 |
| GTACAAATGTGCAAGATGCCT | 87586966 | 87586988 | 0.00 |
| GGAAAGTAGGGGCTGGACCT | 87586983 | 87587005 | 0.01 |
| GTAAGAGTGGAAAGTAGGGGC | 87586990 | 87587012 | 0.02 |
| GATTTTTAAGAGTGGAAAGT | 87586996 | 87587018 | 0.01 |
| GGAAAAAGATTTTTAAGAG | 87587004 | 87587025 | 0.01 |
| GAGCACAGAGAAATTATAA | 87587024 | 87587045 | 0.01 |
| GCTCTCAAGAGATCTTTTTGA | 87587048 | 87587070 | 0.84 |
| GTTCAGAATCTGTAAACTCAT | 87587087 | 87587109 | 0.02 |
| GATCTGTAAACTCATTGGGTA | 87587093 | 87587115 | 0.02 |
| GTCTACACATGAATATACATA | 87587189 | 87587211 | 0.04 |
| GTATATATGTGTATATACCT | 87587312 | 87587334 | 0.01 |
| GCACATATAGAGTTGAATCCA | 87587329 | 87587351 | 0.01 |
| GATATGTGTGTATATGTAATT | 87587345 | 87587367 | 0.39 |
| GATTTTGTATTATATATTATT | 87587462 | 87587484 | 0.06 |
| GCATGTATATGTATATACA | 87587488 | 87587509 | 0.01 |
| GAAACAGATATAGCAATCATG | 87587537 | 87587559 | 0.01 |
| GAAGTCATCAGCTAAGATGG | 87587564 | 87587586 | 0.01 |
| GTCTTAGCTGATGACTTCATC | 87587570 | 87587592 | 0.02 |
| GAAAGATGGCTACCAGCCATG | 87587603 | 87587625 | 0.01 |
| GCTCTTTTACCCTAGTAAAGA | 87587618 | 87587640 | 0.02 |
| GTATATAGATGTTAACTTACA | 87587647 | 87587669 | 0.02 |
| GTAAGTTAACATCTATATATG | 87587652 | 87587674 | 0.03 |
| GTAAAGTTATAAAGGTTTATT | 87587718 | 87587740 | 0.01 |
| GTTATTTGGATTATCAAAAGC | 87587733 | 87587755 | 5.40 |
| GATCTACATCTTTACCCAA | 87587923 | 87587944 | 0.00 |
| GATCTTTACCCAAAGGTAACC | 87587930 | 87587952 | 0.25 |
| GTCTTCTCCTGGTTACCTTT | 87587937 | 87587959 | 0.33 |
| GCCTAGAAGCCAGTCTTCTCC | 87587948 | 87587970 | 0.03 |
| GATGATGGTTTGAATATGCT | 87588059 | 87588081 | 0.05 |
| GATTCAAACCATCATCACAGC | 87588067 | 87588089 | 0.01 |
| GATCTTAACCAGCTGTGATGA | 87588074 | 87588096 | 0.01 |
| GGTGTTGAAAAGTTAAGCAA | 87588116 | 87588138 | 0.04 |
| GACCAAACTGCCAGAAAATTT | 87588136 | 87588158 | 0.03 |
| GTAACTTACCTAAATTTTC | 87588145 | 87588166 | 0.02 |
| GCAGCATATATTTGTTAAATG | 87588166 | 87588188 | 0.01 |
| GATAAACTCCTAAGGTCTGCG | 87588210 | 87588232 | 0.00 |
| GTGAACCCGCGCAGACCTT | 87588217 | 87588238 | 0.17 |
| GTGAGTGACTACAAAGAGGT | 87588251 | 87588273 | 0.01 |
| GTATGGCTTAGTTTATCGGAA | 87588292 | 87588314 | 0.01 |

| | | | |
|---|---|---|---|
| GTTTCATATGGCTTAGTTTAT | 87588297 | 87588319 | 0.01 |
| GCGTACTGCAGTTTTTCATA | 87588310 | 87588332 | 0.01 |
| GCTACAACGACACACTGTAAA | 87588332 | 87588354 | 0.01 |
| GAATCGTGAATACAGCAAGGC | 87588368 | 87588390 | 0.06 |
| GTCAATTCAAAAATACTGTTA | 87588420 | 87588442 | 0.01 |
| GAATTACAACATGCAAGTTAA | 87588445 | 87588467 | 0.01 |
| GAAAATGTTTGATCGTTTATA | 87588474 | 87588496 | 0.01 |
| GACTAGTGCAATAGATTGC | 87588507 | 87588528 | 0.01 |
| GTAGCTTTGAAAACACACAGA | 87588545 | 87588567 | 0.01 |
| GGTTCTGGTTTCTTATTTTA | 87588569 | 87588591 | 0.02 |
| GACAAACTTTGCTTGGTTC | 87588584 | 87588605 | 0.01 |
| GTAAACTAGACAAACTTTGCT | 87588590 | 87588612 | 0.01 |
| GCTAGTTTAGATATACTTAAT | 87588605 | 87588627 | 0.01 |
| GTAGATATACTTAATAGGTAA | 87588611 | 87588633 | 0.03 |
| GAACAGCTCTCTGAAAAGCTT | 87588636 | 87588658 | 0.00 |
| GTAAAAAGCTGTCCATGAGAG | 87588685 | 87588707 | 0.01 |
| GAGGGCACTTCCCTCTCTCA | 87588696 | 87588718 | 0.00 |
| GTGCCCTCTCCTGACAGCAG | 87588711 | 87588733 | 0.60 |
| GACCATCATCCTCTGCTGTC | 87588720 | 87588742 | 0.06 |
| GATGGTCGCACAGAAAACTCC | 87588737 | 87588759 | 0.02 |
| GCCATGTTTAAAGCTAGCTCC | 87588755 | 87588777 | 0.01 |
| GAACATGGCAAAGCTAAACCC | 87588771 | 87588793 | 0.02 |
| GAGGGATAGCTTCTCGCCCT | 87588788 | 87588810 | 1.35 |
| GCCTGGCAACGGGTGAGGTGA | 87588806 | 87588828 | 0.01 |
| GCAGGGTCCTGGCAACGGGTG | 87588812 | 87588834 | 0.00 |
| GTTGCTCAGGGTCCTGGCAAC | 87588817 | 87588839 | 0.62 |
| GCCACAGTTTGCTCAGGGTCC | 87588824 | 87588846 | 0.01 |
| GCTTGTCCACAGTTTGCTCA | 87588830 | 87588852 | 0.01 |
| GACTGTGGACAAGCAGGACGC | 87588840 | 87588862 | 0.04 |
| GCAAGCAGGACGCTGGGGAGT | 87588848 | 87588870 | 0.01 |
| GTACCTTGTCTAGGCAAAGT | 87588875 | 87588897 | 0.07 |
| GGGGGAACTGTACCTTGTCT | 87588884 | 87588906 | 0.01 |
| GTCTGTGGTGTATGAACTTGG | 87588902 | 87588924 | 0.12 |
| GATCTAATGGCTTTTTCTG | 87588918 | 87588939 | 0.01 |
| GGTTCCTCTGTGAGATCTAA | 87588930 | 87588952 | 0.02 |
| GTAGATCTCACAGAGGAACCT | 87588934 | 87588956 | 0.01 |
| GGAACCTAGGATAACCGTCC | 87588947 | 87588969 | 0.02 |
| GCAGATACTTACTGGCTACC | 87588965 | 87588987 | 0.01 |
| GTAAAAATGGCAGATACTTAC | 87588973 | 87588995 | 0.01 |
| GCTTCCGAATCTATTAAAAA | 87588987 | 87589009 | 0.10 |
| GCTACCCTGTACTGCTTACT | 87589011 | 87589033 | 0.01 |
| GGGTGAAATGTGTCCTTCTC | 87589032 | 87589054 | 2.91 |
| GTCCTTCTCAGGTCTGATGG | 87589043 | 87589065 | 0.00 |
| GGTCTGATGGTGGTGATAC | 87589053 | 87589074 | 0.01 |
| GCAGTGTGACACCAGCAGCCA | 87589092 | 87589114 | 0.01 |
| GACAGCTGAGGCCATGGCTGC | 87589102 | 87589124 | 0.11 |
| GAGGAGACAGCTGAGGCCA | 87589109 | 87589130 | 0.00 |
| GTAGAATTGAGGAGACAGCTG | 87589115 | 87589137 | 0.02 |
| GTTTACACAGGAATAGAATTG | 87589127 | 87589149 | 0.01 |
| GTCTATTCCTGTGTAAAAATC | 87589134 | 87589156 | 0.01 |
| GCTGCAGCCTGATTTTTACAC | 87589140 | 87589162 | 0.02 |
| GTAGTAGTTCTAGGAAAGTG | 87589164 | 87589186 | 0.04 |

| | | | |
|---|---|---|---|
| GCTAGACCTAGTAGTAGTTCT | 87589173 | 87589195 | 0.01 |
| GTATGAGTTTGTCTCGTAGCA | 87589206 | 87589228 | 0.02 |
| GTTTATTAATCCTTTATTTAA | 87589250 | 87589272 | 0.01 |
| GAAGTGGGTTCCTTTAAATAA | 87589259 | 87589281 | 0.09 |
| GCAGGTGCAATCATTGTAAAG | 87589276 | 87589298 | 0.01 |
| GCTTTTTACAGTGAGGCAC | 87589295 | 87589316 | 0.00 |
| GCTGGACTGCTTTTTACAGTG | 87589301 | 87589323 | 0.52 |
| GACTGCACTGCTGGCATAGCC | 87589320 | 87589342 | 0.01 |
| GCAGGCAGGCCACTGCACTGC | 87589330 | 87589352 | 0.00 |
| GCCTGCCTGTTCTCATCTCG | 87589344 | 87589366 | 0.33 |
| GTCTCATCTCGTGGAAACTCT | 87589354 | 87589376 | 0.11 |
| GCTCGTGGAAACTCTTGGACC | 87589360 | 87589382 | 0.45 |
| GGTAGAGAGATTTCAGCACC | 87589378 | 87589400 | 0.80 |
| GCAGTGAGAACTTATCGAGCT | 87589399 | 87589421 | 0.04 |
| GCTCGATAAGTTCTCACTGGC | 87589404 | 87589426 | 0.00 |
| GTTGGAAGCATTGGGATGGCG | 87589433 | 87589455 | 0.02 |
| GGAATTTGGAAGCATTGGGA | 87589438 | 87589460 | 0.68 |
| GTTGTGGGAATTTGGAAGCAT | 87589443 | 87589465 | 0.02 |
| GAAGTGCACCCCAAAAGGTTG | 87589460 | 87589482 | 0.01 |
| GAAGGTGCAGTGGCAAAGCTT | 87589491 | 87589513 | 0.00 |
| GCTCCAGAGAGAAAGGTGCAG | 87589502 | 87589524 | 0.04 |
| GCAGCTGTGCTCCAGAGAGAA | 87589510 | 87589532 | 0.01 |
| GACAGCTGAGTTGCCGTGAAA | 87589526 | 87589548 | 0.08 |
| GTTTGTGTGTTTTCCTTTTCA | 87589538 | 87589560 | 0.39 |
| GTCAACAGATAACTCAATCGT | 87589573 | 87589595 | 0.02 |
| GTTGCAAAATTAGGATGCTAG | 87589602 | 87589624 | 0.01 |
| GTTAGTATATCTTGCAAAATT | 87589612 | 87589634 | 0.01 |
| GACTCCTCCAGTGGTGAGTCC | 87589640 | 87589662 | 0.01 |
| GATCTGCCAGGACTCACCAC | 87589646 | 87589668 | 0.02 |
| GGCAGATCCACCATCTAAAG | 87589661 | 87589683 | 1.30 |
| GAGGCCCCCACTTTAGATGG | 87589668 | 87589690 | 0.00 |
| GCAAGAGGCCCCCACTTTAGA | 87589671 | 87589693 | 0.00 |
| GTCAAAATGTAGCTATCAAG | 87589687 | 87589709 | 0.09 |
| GCTTTTGGATGGCAGACAGA | 87589712 | 87589734 | 0.01 |
| GAAGGATACTGGCTTTTGGA | 87589723 | 87589745 | 0.01 |
| GTTGAAGGAGGAGAAGGATAC | 87589734 | 87589756 | 0.04 |
| GATCCTTCTCCTCCTTCAACC | 87589739 | 87589761 | 0.02 |
| GGTACTTCCAGGTTGAAGG | 87589747 | 87589768 | 0.27 |
| GAGGAGTAGGTGGTACTTCC | 87589757 | 87589779 | 0.32 |
| GGGAACCAATCACTGGGTG | 87589776 | 87589797 | 0.27 |
| GATATCAGGGAACCAATCAC | 87589782 | 87589804 | 0.07 |
| GCCTAGTGAATAAAGATATCA | 87589795 | 87589817 | 0.01 |
| GTATCTTTATTCACTAGGAAA | 87589801 | 87589823 | 0.02 |
| GAAAAGGTTCTGCCACATTTG | 87589818 | 87589840 | 0.01 |
| GTTATACTTTCCCCTCAAATG | 87589829 | 87589851 | 0.03 |
| GTAAAGTTTATGTTAAGTCTG | 87589849 | 87589871 | 0.13 |
| GATCTCAAGAGATGTTTTA | 87589886 | 87589907 | 0.01 |
| GACATGCAAGTTATGAAAGTG | 87589913 | 87589935 | 0.00 |
| GATGTGAAAACTTAAGTTGTG | 87589936 | 87589958 | 0.91 |
| GAACTTAAGTTGTGAGGGTCT | 87589943 | 87589965 | 0.05 |
| GGGTCTAGGAAGGTGTTTAA | 87589957 | 87589979 | 0.19 |
| GAAAGTGTCTCAGATAGTGC | 87590036 | 87590058 | 0.01 |

| | | | |
|---|---|---|---|
| GCTCAGATAGTGCTGGAGAGA | 87590044 | 87590066 | 0.01 |
| GACTTTCAAACTCAGGACCTC | 87590096 | 87590118 | 0.01 |
| GATTGTGGAACTTTCAAACTC | 87590104 | 87590126 | 0.02 |
| GTTTGAAAGTTCCACAATGTG | 87590110 | 87590132 | 0.01 |
| GTTTTTATTCAAGGGCGCTCT | 87590214 | 87590236 | 0.02 |
| GCAAGGGCGCTCTTGGGCCCA | 87590222 | 87590244 | 0.08 |
| GCATCAAACCCCATTTCAGA | 87590662 | 87590684 | 0.01 |
| GATGTAGCAATATCACAGAGA | 87590782 | 87590804 | 0.01 |
| GAGTTTTAACATTCATCAGT | 87590823 | 87590845 | 0.01 |
| GTTCTGATAAGACATTAGCC | 87590846 | 87590868 | 0.00 |
| GATAAGACATTAGCCTGGCTC | 87590852 | 87590874 | 0.01 |
| GTCAGTGCTTACCAGAGCC | 87590864 | 87590885 | 0.00 |
| GTCAAGATTTTCAATTTCTTT | 87590906 | 87590928 | 0.02 |
| GATCTATACACTCAGACTTC | 87590984 | 87591006 | 0.05 |
| GACACTCAGACTTCTGGACAG | 87590991 | 87591013 | 0.28 |
| GATGATTTGGGGTTAAATGCA | 87591023 | 87591045 | 0.00 |
| GTTGGGGTTCCAAAATGATTT | 87591036 | 87591058 | 0.02 |
| GCTGAGTCATAGTAGTAGCTT | 87591054 | 87591076 | 0.00 |
| GTCAGTACAGTTTACCAACAG | 87591080 | 87591102 | 0.02 |
| GACTGTTAGCATTGCCACTGT | 87591093 | 87591115 | 1.12 |
| GTAAAAAAGTCTTGTTGCTTT | 87591195 | 87591217 | 0.01 |
| GCTTGAATCCACCTCTTAT | 87591224 | 87591245 | 1.47 |
| GTCCACCTCTTATTGGTCAAA | 87591231 | 87591253 | 0.01 |
| GGAAACAGGTTGTTGACTGA | 87591268 | 87591290 | 0.01 |
| GAATAATTGGAATAGGCAGG | 87591291 | 87591313 | 0.01 |
| GCCTATTCCAATTATTCCTG | 87591297 | 87591319 | 0.12 |
| GTTCCAATTATTCCTGAGGGC | 87591302 | 87591324 | 0.01 |
| GTTGCTGGAGATCCAGCCCTC | 87591313 | 87591335 | 1.42 |
| GGGCAGGTGGTGGTGGTTGC | 87591329 | 87591351 | 0.03 |
| GATCAGAGAAAGGGCAGGTGG | 87591339 | 87591361 | 1.88 |
| GAAGATCAGAGAAAGGGCAGG | 87591342 | 87591364 | 0.00 |
| GAGTCCCAAGATCAGAGAAA | 87591349 | 87591371 | 2.58 |
| GTAGTTCTGGTAGGTGGGGGA | 87591372 | 87591394 | 0.01 |
| GCCCATAGTTCTGGTAGGTG | 87591377 | 87591399 | 0.02 |
| GTTTAGGCCCATAGTTCTGGT | 87591382 | 87591404 | 0.01 |
| GGTTTCAAATGTACACCTAA | 87591405 | 87591427 | 0.01 |
| GGGGGAATTTTTCCCCCTT | 87591420 | 87591441 | 0.14 |
| GATAAAGTTGAGTAGTTTAG | 87591438 | 87591460 | 0.01 |
| GAACTTTATCTTCAACCTTCC | 87591452 | 87591474 | 0.09 |
| GAAGGAACATCTGGACCTGGA | 87591466 | 87591488 | 0.01 |
| GTAGATTTTGAAGGAACATC | 87591476 | 87591498 | 0.02 |
| GTCCAGGATGTAGATTTTGA | 87591485 | 87591507 | 0.01 |
| GTAGTTGCTTCGAATCTGTCC | 87591501 | 87591523 | 0.02 |
| GATTCGAAGCAACTAGCCCC | 87591509 | 87591531 | 0.01 |
| GTTGTGAGGCTGGACCGCCTG | 87591525 | 87591547 | 0.11 |
| GCAGCAGTTGGAGCCTTTGTG | 87591540 | 87591562 | 0.02 |
| GGTACATGCAGGCAGCAGT | 87591553 | 87591574 | 0.12 |
| GTTTGGAGCTAGGTACATGC | 87591563 | 87591585 | 0.48 |
| GCTGCGGGACCGTTTGGAGCT | 87591573 | 87591595 | 1.32 |
| GCTAGGCTCTGCGGGACCGTT | 87591580 | 87591602 | 0.01 |
| GACTTACTGCCTAGGCTCTGC | 87591589 | 87591611 | 0.01 |
| GTCATTGTACTTACTGCCT | 87591598 | 87591619 | 0.01 |

| | | | |
|---|---|---|---|
| GTACAATGACATCCTCTTCC | 87591610 | 87591632 | 0.53 |
| GACATCCTCTTCCAGGCCT | 87591617 | 87591638 | 0.02 |
| GGCTGGCCAAGGCCTGGAAG | 87591622 | 87591644 | 0.01 |
| GATGCTGGCTGGCCAAGGCC | 87591628 | 87591650 | 0.01 |
| GCTGTGATGCTGGCTGGCCA | 87591633 | 87591655 | 0.01 |
| GAAGAAAGCTGTGATGCTGGC | 87591639 | 87591661 | 0.01 |
| GTTAGGCCCCCAGAGTTGTCA | 87591660 | 87591682 | 0.35 |
| GGGTTCCTTGACAACTCTGG | 87591665 | 87591687 | 0.60 |
| GTCAAGGAACCCAGACCGA | 87591676 | 87591697 | 0.01 |
| GCAGTCTGTTTCCTTCGGTCT | 87591685 | 87591707 | 0.12 |
| GTCTCCACAGTCTGTTTCCTT | 87591691 | 87591713 | 0.01 |
| GACAGACTGTGGAGAGCCTTT | 87591700 | 87591722 | 0.01 |
| GAGCCTTTTGGGGTGCAGCT | 87591712 | 87591734 | 0.00 |
| GGGTGCAGCTTGGCAGGACA | 87591722 | 87591744 | 0.08 |
| GCTTGGCAGGACAAGGACA | 87591729 | 87591750 | 0.02 |
| GCAGGACAAGGACAAGGAAA | 87591734 | 87591756 | 0.03 |
| GTTCAGGCAGGGACTTGACAT | 87591800 | 87591822 | 0.01 |
| GCAGGGACTTGACATTGGCCA | 87591806 | 87591828 | 0.01 |
| GATCCTTACTTCCTTGTCCT | 87591823 | 87591845 | 0.05 |
| GGAAGTAAGGATCTGAGTTT | 87591833 | 87591855 | 0.10 |
| GTCCTTAGAAACAGCTATCAG | 87591891 | 87591913 | 0.01 |
| GCTATCAGCGGAGTGATCAC | 87591903 | 87591925 | 0.00 |
| GTAATCAAGGAACAAGCAAGG | 87591938 | 87591960 | 0.01 |
| GTCTTGCTTGATCCCTCAT | 87591963 | 87591984 | 0.52 |
| GCAGGGTGAGTAGTCCAATGA | 87591975 | 87591997 | 0.01 |
| GAAATGGTATAAAAAGTTAAT | 87592016 | 87592038 | 0.03 |
| GGGAAAATGAAATTTGCCTT | 87592037 | 87592059 | 0.01 |
| GATTTGCCTTCGGTCTCAGAA | 87592048 | 87592070 | 0.00 |
| GCCAGTCCATTCTGAGACCGA | 87592053 | 87592075 | 0.01 |
| GATTGACTGACTGAGCGGGCA | 87592142 | 87592164 | 0.01 |
| GATTGGTGACACAGGAATA | 87592188 | 87592209 | 0.16 |
| GAAGGGGTGATTGGTGACAC | 87592195 | 87592217 | 0.02 |
| GAAGATATATGAAGGGGTGAT | 87592204 | 87592226 | 0.02 |
| GACAGGTGAAGATATATGAAG | 87592211 | 87592233 | 0.02 |
| GTTCACCTGTTATTTAAAAAA | 87592225 | 87592247 | 0.03 |
| GAAAAAGGAATATTAGAAACT | 87592241 | 87592263 | 0.05 |
| GGCGCTGAGTCACTAAAGGC | 87592275 | 87592297 | 0.02 |
| GTGGCTGGTGATGATGATGC | 87592296 | 87592318 | 0.24 |
| GGACCTTTTAAAGCACTTGG | 87592315 | 87592337 | 0.01 |
| GCAAGGACCTTTTAAAGCACT | 87592318 | 87592340 | 0.01 |
| GAGAGCTGTGTGTGATGGCA | 87592336 | 87592358 | 0.02 |
| GTAAAAAAAGAACAAGAGACA | 87592376 | 87592398 | 1.52 |
| GATCTGACACTCCCTCTTT | 87592470 | 87592491 | 0.01 |
| GAACAAGATGGGCTGGAGATA | 87592581 | 87592603 | 0.01 |
| GAAAGAGAATAAGAACAAGAT | 87592593 | 87592615 | 0.02 |
| GTTGTTCTGGTTTTTCTAGAC | 87592692 | 87592714 | 0.01 |
| GTACTGAGTTCGAGGCCAGAC | 87592759 | 87592781 | 0.01 |
| GTTCCAACACGTGGGAGGCAG | 87592791 | 87592813 | 0.01 |
| GCTTTAATTCCAACACGTGGG | 87592797 | 87592819 | 0.05 |
| GCAGAGACAGAGAATAAGTGG | 87592827 | 87592849 | 0.01 |
| GCTCTTGTTTCCACTCTGAGA | 87592887 | 87592909 | 0.01 |
| GTGAAAGGCCATCTCAGAG | 87592896 | 87592917 | 3.71 |

| | | | |
|---|---|---|---|
| GTATCGGGGATGGGGAGTGAA | 87592910 | 87592932 | 0.00 |
| GCAAGAGATTTATCGGGGAT | 87592920 | 87592942 | 0.01 |
| GCTCATGCAAGAGATTTATCG | 87592925 | 87592947 | 0.01 |
| GCTTGCATGAGATCTGCTGCA | 87592938 | 87592960 | 0.01 |
| GTGATCTCTGCAGCATTCCT | 87592962 | 87592984 | 0.02 |
| GCCTGTGGCAGATCAGAGCCA | 87592979 | 87593001 | 0.01 |
| GTTCATGCAGTGGAGTCCTG | 87592995 | 87593017 | 0.36 |
| GAGTGTTAGGGTTCATGCAG | 87593005 | 87593027 | 0.43 |
| GGGCTACACAGTGAGTGTTA | 87593017 | 87593039 | 0.01 |
| GTTTTGTAGATCAAGTTCATC | 87593058 | 87593080 | 0.01 |
| GTAGATCAAGTTCATCTGGCC | 87593063 | 87593085 | 0.01 |
| GCAAGTTCATCTGGCCTGGCC | 87593068 | 87593090 | 0.01 |
| GATCTCTGGGAGTGCCAGGCC | 87593081 | 87593103 | 0.22 |
| GAACATTCTGGAGACAGAAGC | 87593107 | 87593129 | 0.02 |
| GTTAAAAAATCTCTCTTTGT | 87593157 | 87593179 | 0.01 |
| GTTAGAAGAGAAGAGAATAGA | 87593183 | 87593205 | 0.01 |
| GAAAACATTGATCAGCAAGCA | 87593204 | 87593226 | 0.01 |
| GTTGGTTCAAACTCAGTTGTC | 87593229 | 87593251 | 1.10 |
| GAACCAAGACCCATGAGCAGA | 87593246 | 87593268 | 0.02 |
| GGTTTTCTCCTTCTGCTCAT | 87593254 | 87593276 | 0.06 |
| GTTTGACAACTTAATGTGATT | 87593275 | 87593297 | 0.01 |
| GATTTATAATGTGGAGGTCAA | 87593302 | 87593324 | 0.02 |
| GTCTATTTATTTATAATGTGG | 87593309 | 87593331 | 0.01 |
| GCAATGCATTTGCAAGTCAA | 87593353 | 87593375 | 2.99 |
| GTCTGGATTTGTTACAGAAAA | 87593393 | 87593415 | 0.01 |
| GCAAATCCAGAAAGGAAGAAA | 87593406 | 87593428 | 0.01 |
| GCTTTTCCATTTCTTCCTTTC | 87593411 | 87593433 | 0.88 |
| GAAATGGAAAAGTACCTACT | 87593422 | 87593444 | 0.02 |
| GTACCTACTAGGCTGAAGCC | 87593433 | 87593455 | 0.01 |
| GTAGGCTGAAGCCAGGGAGTG | 87593441 | 87593463 | 0.01 |
| GCCAGGGAGTGTGGAAGGGT | 87593450 | 87593472 | 0.01 |
| GGAGTGTGGAAGGGTTGGGT | 87593455 | 87593477 | 0.14 |
| GGGTTGGGTTGGGGGTGAG | 87593466 | 87593487 | 0.01 |
| GAGTGGTGTATGTGACTGTG | 87593482 | 87593504 | 1.50 |
| GTGACTGTGTGGGGGTAAG | 87593493 | 87593514 | 0.89 |
| GAATGTGTGTGTAGTGTGT | 87593548 | 87593569 | 0.68 |
| GTGTGTGACTGTGTGTGTAA | 87593583 | 87593605 | 0.03 |
| GTATGTGAGTATGTATGTGAG | 87593628 | 87593650 | 0.01 |
| GATGTATGTGAGTGGCAGTGG | 87593637 | 87593659 | 0.01 |
| GATTGTGTGTTGTTGTGTC | 87593669 | 87593690 | 0.01 |
| GTGTGATTGTGAGTATGTGT | 87594021 | 87594043 | 0.01 |
| GTTGTGAGTATGTGTGGGAGT | 87594027 | 87594049 | 3.53 |
| GGAGTAGGTGATTGTGTGTG | 87594042 | 87594064 | 0.38 |
| GGTGATTGTGTGTGTGGGGG | 87594048 | 87594070 | 0.01 |
| GTTATATTCTAATGTTAATAC | 87594085 | 87594107 | 3.44 |
| GTCCTCAAGATCTATTTGCCT | 87594109 | 87594131 | 0.01 |
| GCAGACTCTCTTGTCTCCG | 87594126 | 87594147 | 0.03 |
| GTAAGGTCATAGAGTGTCAC | 87594156 | 87594178 | 0.21 |
| GAAGAGACATAGGTGAGTCTT | 87594398 | 87594420 | 1.15 |
| GGTGAGTCTTAGGGTTCCC | 87594408 | 87594429 | 0.02 |
| GTCTTAGGGTTCCCAGGTTT | 87594413 | 87594435 | 0.01 |
| GTTCCCAGGTTTGGGGTCAG | 87594421 | 87594443 | 0.09 |

| | | | |
|---|---|---|---|
| GAGGAAAACCACAGAGAACA | 87594440 | 87594462 | 0.05 |
| GCTTCTTCTCCTTGTTCTCTG | 87594448 | 87594470 | 4.18 |
| GAACAAGGAGAAGAAGGAGA | 87594455 | 87594477 | 6.18 |
| GAGTTATGAAAACATGGCCC | 87594506 | 87594528 | 0.01 |
| GAACATGGCCCTGGGGGCAAT | 87594516 | 87594538 | 0.03 |
| GATTAACTCCAATTGCCCCCA | 87594523 | 87594545 | 0.08 |
| GTTACTTAGTATGTTTCATC | 87594552 | 87594574 | 0.03 |
| GTTGGTAGATATCTACCCTCT | 87594611 | 87594633 | 0.01 |
| GTAAATATATGGTTGCGTG | 87594659 | 87594680 | 0.03 |
| GTTGCGTGTGGCTCTTATTC | 87594670 | 87594692 | 0.01 |
| GATTCAGGAACTGAATGATGA | 87594686 | 87594708 | 0.03 |
| GGAACTGAATGATGAAGGTG | 87594691 | 87594713 | 0.02 |
| GAAATTTTTAATCACAATCT | 87594721 | 87594743 | 0.03 |
| GCAACATGTGTGAGTTTTCAG | 87594746 | 87594768 | 0.01 |
| GGAAAAATTCTAGTTCCTC | 87594767 | 87594788 | 0.01 |
| GACTCTGAAGCTGTGTCCTG | 87594782 | 87594804 | 0.54 |
| GAGTCCCTTCCTGTAGAAGT | 87594800 | 87594822 | 0.61 |
| GCATCAGAACCAACTTCTAC | 87594809 | 87594831 | 1.24 |
| GTTGGTTCTGATGCTAATA | 87594818 | 87594839 | 0.01 |
| GATAAGGTCCTGTTCCTCAAT | 87594834 | 87594856 | 0.02 |
| GCATCCTCAGACATTGATTGC | 87594880 | 87594902 | 0.00 |
| GTCAATGTCTGAGGATGGAGC | 87594887 | 87594909 | 0.65 |
| GCTGGACACGTAATGTTTCG | 87594905 | 87594927 | 0.01 |
| GGGAGGATGCAGCAAGAATC | 87594926 | 87594948 | 0.58 |
| GCAGCAAGAATCTGGAGAAC | 87594934 | 87594956 | 0.01 |
| GAATCTGGAGAACTGGGGAAG | 87594942 | 87594964 | 0.01 |
| GGAAGGAGAGAGTAGCAGC | 87594963 | 87594984 | 1.35 |
| GCAACCAGTCACCTGAGCTTT | 87594994 | 87595016 | 0.26 |
| GCCACTCCAGCCGAAAGCTC | 87595004 | 87595026 | 0.01 |
| GCTTTCGGCTGGAGTGGCCAC | 87595010 | 87595032 | 0.01 |
| GTAATCGGACAGAATGGCCAG | 87595026 | 87595048 | 0.08 |
| GTCTGGCATAATCGGACAGAA | 87595033 | 87595055 | 0.00 |
| GATTATGCCAGAGGAAGTTT | 87595044 | 87595066 | 0.01 |
| GGGCATTCCTAAACTTCCTC | 87595051 | 87595073 | 0.01 |
| GTCATCCATCGAGTTAGCAA | 87595075 | 87595097 | 0.02 |
| GAAATGCCTTTGCTAACTCGA | 87595080 | 87595102 | 0.00 |
| GCAAAGGCATTTTAAGATTAA | 87595092 | 87595114 | 0.03 |
| GATAAAGATCAACACACACAC | 87595116 | 87595138 | 0.01 |
| GTGTGTTGATCTTTATCTG | 87595123 | 87595144 | 0.02 |
| GATCTTTATCTGTGGATCCAA | 87595131 | 87595153 | 0.01 |
| GCCCCCATCCAGATTCCTT | 87595147 | 87595168 | 0.46 |
| GCTGCCCAGAGCTTAAAGTG | 87595167 | 87595189 | 0.01 |
| GCAAAAACTAAGCAGTACAGA | 87595193 | 87595215 | 0.02 |
| GCAGATGGTACACAGTGTGGC | 87595209 | 87595231 | 0.00 |
| GAGAATCTACTATTGATTG | 87595233 | 87595254 | 0.14 |
| GTTGATTGAGGAATTCTCAGA | 87595245 | 87595267 | 0.01 |
| GAAATGATACAGTTTAAGAGC | 87595298 | 87595320 | 0.02 |
| GAACTGTTTTTTTCATCAGAG | 87595324 | 87595346 | 0.01 |
| GAGTCACATGACTCTGATGC | 87595350 | 87595372 | 0.00 |
| GATGCTGGAAAACTTGAACAA | 87595366 | 87595388 | 0.01 |
| GAAGCTCAGCTCAGAGATGC | 87595399 | 87595421 | 0.02 |
| GCCTGTCTCTCTGCACAGCA | 87595426 | 87595448 | 0.02 |

| | | | |
|---|---|---|---|
| GAGACAGGCTGTGACTCTA | 87595440 | 87595461 | 0.05 |
| GATTACCTGCTGTGGGAAGC | 87595488 | 87595510 | 0.00 |
| GTCCTTTGATTACCTGCTGT | 87595495 | 87595517 | 0.01 |
| GTTTTGATTTAATTGCTTTTA | 87595529 | 87595551 | 0.02 |
| GATTTTGCCCGAGCCACATG | 87595567 | 87595589 | 0.05 |
| GCTCAGGAAGAAATCCCCATG | 87595580 | 87595602 | 0.03 |
| GGGGATTTCTTCCTGAGGTT | 87595586 | 87595608 | 0.01 |
| GTTGTATCAGTTCCGAACCTC | 87595597 | 87595619 | 0.16 |
| GAGCTTAAGATGAGAGGCAG | 87595621 | 87595643 | 0.01 |
| GTAAGATGAGAGGCAGGGGGC | 87595626 | 87595648 | 0.15 |
| GAGATGGCACTTGTGTTCC | 87595650 | 87595671 | 0.50 |
| GCACTTGTGTTCCAGGAATC | 87595656 | 87595678 | 0.55 |
| GTGTTCCAGGAATCAGGCTC | 87595662 | 87595684 | 0.03 |
| GATCACCTGAGCCTGATTCC | 87595667 | 87595689 | 0.68 |
| GCAGGCTCAGGTGATCAGGTT | 87595675 | 87595697 | 0.01 |
| GATTGGTGGCTGATGAGACAG | 87595704 | 87595726 | 0.02 |
| GCTCAAAAAGAAAAATATTGG | 87595719 | 87595741 | 0.01 |
| GGATAGATATGCATGGATT | 87595772 | 87595793 | 0.01 |
| GCAAAAATGGATAGATATGCA | 87595778 | 87595800 | 0.01 |
| GAAAAATGCAAATTACAAAAA | 87595792 | 87595814 | 0.00 |
| GACACACAATGACAATTTACT | 87595823 | 87595845 | 0.29 |
| GATGATGTATTTATCCACTCA | 87595869 | 87595891 | 0.00 |
| GCTTAACTCAGTGTCCATGAG | 87595882 | 87595904 | 0.01 |
| GAATTTATTTCAAGCATTAAA | 87595906 | 87595928 | 0.02 |
| GTCAAGCATTAAAAGGAGATT | 87595914 | 87595936 | 0.02 |
| GTTAAAAGGAGATTTGGGGGC | 87595921 | 87595943 | 0.01 |
| GACAAGATTTGATTCTGTCTC | 87596037 | 87596059 | 0.01 |
| GTAAAATAAATCTTTGTTTTA | 87596102 | 87596124 | 0.03 |
| GATTAAGATTTAAACAGTCCA | 87596127 | 87596149 | 0.02 |
| GCTTCAGCTGTCAGACACCT | 87596144 | 87596166 | 0.01 |
| GTCTGACAGCTGAAGCAGAAA | 87596152 | 87596174 | 0.04 |
| GTCTTTGAGTCTTTGTTCTAC | 87596183 | 87596205 | 0.02 |
| GTCCTAAACTTGATTCTATTT | 87596227 | 87596249 | 0.13 |
| GCCAGTACGAGAATGAATTGA | 87596258 | 87596280 | 0.02 |
| GCAAAGAGAAGGTAGTATTG | 87596290 | 87596312 | 26.93 |
| GAGAAGGTAGTATTGTGGAA | 87596295 | 87596317 | 0.01 |
| GATAAGATTATGTTAGTTTTC | 87596396 | 87596418 | 0.02 |
| GTAAGAGGTACTACAATCT | 87596430 | 87596451 | 0.01 |
| GCAATCTCAGCATCTGTAAG | 87596444 | 87596466 | 0.01 |
| GTATGAATGACCAATGAAATT | 87596472 | 87596494 | 0.04 |
| GCTCTTTACCAAATTTCAT | 87596481 | 87596502 | 0.01 |
| GAAATTTGGTAAAGAGCTTG | 87596486 | 87596508 | 0.01 |
| GTTCTTCTGGTGTGGGGGGAG | 87596558 | 87596580 | 0.01 |
| GGTGTGGTGGCGCTGACTTA | 87596641 | 87596663 | 0.01 |
| GTTGGTGGTATCAGGGTGTGG | 87596654 | 87596676 | 0.01 |
| GCCTGTGCAATGAACGACACG | 87596821 | 87596843 | 0.01 |
| GACCTGCTGGATGGGTCATG | 87596911 | 87596933 | 0.00 |
| GGGTCATGTGGTAATTACTC | 87596923 | 87596945 | 5.53 |
| GAACTCATTGGATTTTCCC | 87596996 | 87597017 | 0.01 |
| GAATTGCTGTTCTCTCTACC | 87597013 | 87597035 | 0.01 |
| GTATAAGATGTTGGTGTTTC | 87597035 | 87597057 | 0.03 |
| GTTTGCATCTGTATAAGATGT | 87597044 | 87597066 | 0.01 |

| | | | |
|---|---|---|---|
| GTTATACAGATGCAAACAAGA | 87597052 | 87597074 | 0.00 |
| GACTTTTGAACTGAGATATA | 87597122 | 87597144 | 0.01 |
| GAATTACATGCAATCTTTA | 87597146 | 87597167 | 0.01 |
| GTGATTATGTTAAGAGATTT | 87597182 | 87597204 | 0.01 |
| GTTGCAACTGTATAAACAATA | 87597279 | 87597301 | 4.60 |
| GGAATATGAGTAATATATAG | 87597326 | 87597348 | 0.01 |
| GCCTAGCAAACCTGTATGAGA | 87597347 | 87597369 | 1.39 |
| GTTGCTAGGTCCATTAGTTCA | 87597362 | 87597384 | 0.10 |
| GTCATCATGTCCTTGAACTAA | 87597371 | 87597393 | 0.01 |
| GAAACTGACCAATTACTAAT | 87597392 | 87597414 | 0.01 |
| GATACATTTCCAATTAGTAAT | 87597400 | 87597422 | 0.03 |
| GAAAAAAAACATCATATTAAT | 87597443 | 87597465 | 0.03 |
| GAAACATCATATTAATAGGAG | 87597448 | 87597470 | 0.02 |
| GACAAAAGTTTCTATCCCT | 87597477 | 87597498 | 0.01 |
| GTTTCTTTGGCTTGTTGCCAA | 87597492 | 87597514 | 0.01 |
| GACGTTTTAGAATTATTTCTT | 87597506 | 87597528 | 0.01 |
| GTATGTGTTTTCTATACAACC | 87597531 | 87597553 | 0.02 |
| GCAACCAGGTCTGTCACCTCC | 87597546 | 87597568 | 0.00 |
| GTCACCTCCTGGTAGTAACCC | 87597558 | 87597580 | 0.01 |
| GTGTCCCCGGGGTTACTACC | 87597564 | 87597586 | 0.64 |
| GTAACCCCGGGGACACCAAA | 87597571 | 87597593 | 0.02 |
| GTCATTCCTTTTGGTGTCCC | 87597577 | 87597599 | 0.00 |
| GTTATCAGATGTCATTCCTTT | 87597586 | 87597608 | 0.01 |
| GTTTGCAGAATTTGAAAAACA | 87597623 | 87597645 | 0.28 |
| GCATATCGTTGACATGTACTG | 87597656 | 87597678 | 0.99 |
| GACATGTACTGAGGGTTCCAG | 87597666 | 87597688 | 0.22 |
| GTAAAGCAGATATCTGCCCAC | 87597682 | 87597704 | 0.01 |
| GTGTGTTCTGACACACTTAT | 87597722 | 87597744 | 0.01 |
| GATTGGACATAATGACAATTA | 87597740 | 87597762 | 0.01 |
| GTTATCAGTTTTAATTTGTAC | 87597768 | 87597790 | 0.01 |
| GTTTTATTGGATAAATATGT | 87597795 | 87597817 | 0.03 |
| GAAAGAATTGTTTAGTTTTAT | 87597808 | 87597830 | 0.02 |
| GAATATGTTTTATATTGTAAT | 87597832 | 87597854 | 0.01 |
| GGTATACCGCACAATCCTAC | 87597858 | 87597880 | 0.03 |
| GGCTCACTGCTTGTCCTGT | 87597873 | 87597894 | 0.00 |
| GTGAGCCAATTGTACCTTAA | 87597888 | 87597910 | 0.01 |
| GTTTAGTAAGCATCCTTTA | 87597902 | 87597923 | 0.01 |
| GGATGCTTACTAAACAAAG | 87597909 | 87597930 | 1.37 |
| GACTAACCCCCCTCCCCCCA | 87597933 | 87597955 | 0.04 |
| GATCTGTCCCTGGGGGGAGGG | 87597939 | 87597961 | 1.23 |
| GAAGAAAACTTAGAGTTAACA | 87597971 | 87597993 | 0.04 |
| GCAACAGAAGCTGAGAGATAC | 87598013 | 87598035 | 5.95 |
| GAGATACTGGGTTGTCTTTA | 87598026 | 87598048 | 0.00 |
| GCTTTGGAGAAGCAGGCAAA | 87598051 | 87598073 | 0.01 |
| GTTTCTCTGCTTTGGAGAAGC | 87598058 | 87598080 | 0.00 |
| GTTCTCCAAAGCAGAGAAAGG | 87598063 | 87598085 | 0.04 |
| GGAGGGGGTGTCTGCTTGCC | 87598081 | 87598103 | 0.01 |
| GCTGCTTGCCTGGCAGATTTA | 87598092 | 87598114 | 0.08 |
| GCTGACTCCCTTAAATCTGCC | 87598099 | 87598121 | 0.02 |
| GTTAAGGGAGTCAGGATCTGT | 87598109 | 87598131 | 1.69 |
| GAAGCACAGATGATAAGAGG | 87598131 | 87598153 | 1.21 |
| GATAAGAGGAGGTACATTTG | 87598142 | 87598164 | 0.01 |

| | | | |
|---|---|---|---|
| GGAGGTACATTTGAGGCTCA | 87598149 | 87598171 | 0.00 |
| GTACATTTGAGGCTCAAGGCG | 87598154 | 87598176 | 0.10 |
| GGAAGGAACAGGGAGGGTTT | 87598202 | 87598224 | 0.06 |
| GGAGGGTTTTGGATGTAAAC | 87598213 | 87598235 | 0.02 |
| GCAATTGCTGAAATATACACA | 87598238 | 87598260 | 0.03 |
| GCAATTGTTTCTATTAGCTTG | 87598255 | 87598277 | 0.00 |
| GAAGTGCCAAGAGTTAGATTT | 87598280 | 87598302 | 0.01 |
| GTTGAGGCTTAAAGATAGGAA | 87598320 | 87598342 | 0.36 |
| GAGGATTGAGGCTTAAAGAT | 87598325 | 87598347 | 2.14 |
| GAGTCTCTGAGGGAGGATTG | 87598337 | 87598359 | 0.25 |
| GCTGCAGGAGTTGAGTCTCTG | 87598348 | 87598370 | 0.01 |
| GAGACTCAACTCCTGCAGGT | 87598353 | 87598375 | 0.01 |
| GACTGCTAGAGCCGACCTGC | 87598364 | 87598386 | 0.01 |
| GAAATAAAATTTAAAAACCTG | 87598406 | 87598428 | 0.03 |
| GATCGGTTGGTCTAGTTCCTC | 87598422 | 87598444 | 0.00 |
| GTAGCCTTATAGTATATCGGT | 87598436 | 87598458 | 0.01 |
| GTATAAGGCTATGTTGACATA | 87598449 | 87598471 | 0.02 |
| GGTTGTGCTTATGTGTCTAC | 87598502 | 87598524 | 0.01 |
| GATGTGTCTACAGGGAAAACA | 87598512 | 87598534 | 0.00 |
| GCTGATCTTTCTAAGTTTTGT | 87598538 | 87598560 | 0.01 |
| GAAAACTTAGAAAGATCAGAT | 87598543 | 87598565 | 0.01 |
| GTAGAAAGATCAGATCGGACC | 87598549 | 87598571 | 0.01 |
| GTTGATAAGAGGTTTTTCC | 87598567 | 87598588 | 0.02 |
| GCATGTCTCAGTTGATAAG | 87598577 | 87598598 | 0.01 |
| GTCACAAAGCAAACAGTGC | 87598604 | 87598625 | 0.01 |
| GTCACAGACTGTCTCAATTAC | 87598636 | 87598658 | 0.00 |
| GTTGATATGTGTGCATATTT | 87598664 | 87598686 | 0.01 |
| GCACACATATCAACTTCAAA | 87598673 | 87598695 | 0.01 |
| GACTTCTCCTGACATCCCAAC | 87598792 | 87598814 | 0.01 |
| GCCAGGACCAGTTGGGATGTC | 87598798 | 87598820 | 0.02 |
| GACCAGGACCAGGACCAGTT | 87598806 | 87598828 | 0.10 |
| GTCAGCCTGGACCAGGACC | 87598816 | 87598837 | 0.01 |
| GTCCTGGTCCAGGCTGACTCA | 87598821 | 87598843 | 0.01 |
| GAATCCCCGTGAGTCAGCC | 87598828 | 87598849 | 0.01 |
| GACGGGGATTCTGCGCAAGCC | 87598840 | 87598862 | 0.01 |
| GCAAGCCTGGCAGTTTCTGC | 87598853 | 87598875 | 0.73 |
| GTCCTCCTGACACTATTGAAC | 87598908 | 87598930 | 0.05 |
| GGTATCCTGATAACAGAGAT | 87598937 | 87598959 | 0.03 |
| GCTTAGAAGGAGTTCTTGGG | 87598969 | 87598991 | 0.02 |
| GCGCTTAGAAGGAGTTCTTG | 87598971 | 87598993 | 0.00 |
| GGATTCAAGATGCGCTTAGA | 87598982 | 87599004 | 0.04 |
| GAAGAGGGGGCTAGAAGAGG | 87599055 | 87599077 | 0.04 |
| GAAGCCTACACACCTCTGCTT | 87599123 | 87599145 | 0.29 |
| GATATGGGAAGCCTAAGCAG | 87599134 | 87599156 | 0.03 |
| GACATTTGATCACAATGATA | 87599150 | 87599172 | 0.01 |
| GCAAAAAAAAAATTTAAACT | 87599172 | 87599194 | 0.07 |
| GTTCCTTTCTGTTGCATGTA | 87599220 | 87599242 | 0.00 |
| GAGCCAGAGCAAGAGAGAGA | 87599452 | 87599474 | 0.05 |
| GAGTTGCACATGAAACTGAC | 87599625 | 87599647 | 0.01 |
| GACGATAGCAGGCACTAAAGC | 87599651 | 87599673 | 0.33 |
| GGAGGACCCTAACGATAGC | 87599663 | 87599684 | 0.56 |
| GTGAGTGACAGACACTGTGG | 87599680 | 87599702 | 0.08 |

| | | | |
|---|---|---|---|
| GTCAGCTCTGCTCAGAACTGC | 87599705 | 87599727 | 0.01 |
| GTTCTGAGCAGAGCTGAGAC | 87599711 | 87599733 | 55.68 |
| GGTCTTCAGACATTACCACT | 87599732 | 87599754 | 2.87 |
| GCTGATGCTCTTAGAACCTAG | 87599747 | 87599769 | 0.02 |
| GAAGAGCATCAGTGAAGTCGC | 87599759 | 87599781 | 0.39 |
| GTCTGAGCATGCTCAAGAATT | 87599782 | 87599804 | 0.01 |
| GCATGCTCAGATCCAGAGCA | 87599794 | 87599816 | 0.69 |
| GCCTAGGAGGTGGCCGTGCTC | 87599806 | 87599828 | 0.01 |
| GGCCACCTCCTAGGGGTTCT | 87599815 | 87599837 | 0.02 |
| GAAGTGCCAAGAACCCCTAGG | 87599820 | 87599842 | 0.10 |
| GTTCTTGGCACTTGAGTTGT | 87599830 | 87599852 | 0.00 |
| GACTTGAGTTGTTGGTGTCAC | 87599839 | 87599861 | 0.01 |
| GCACAGGGAATAAACAGCATT | 87599856 | 87599878 | 0.02 |
| GATTTGGCTTACTTTTTACAA | 87599873 | 87599895 | 0.54 |
| GTCTAACTCAGAGGTGACAAA | 87599904 | 87599926 | 0.15 |
| GAACTAGGCTCTAACTCAG | 87599914 | 87599935 | 0.06 |
| GAGCCTAGTTCAATAAACCC | 87599925 | 87599947 | 0.01 |
| GAGATAAAGGAAGGTGTTCC | 87599943 | 87599965 | 0.02 |
| GTCGGCTTAGGAGATAAAGGA | 87599952 | 87599974 | 0.01 |
| GTCTGGGTGAAGCTTCGGCTT | 87599965 | 87599987 | 0.00 |
| GTCAGTGTCTGGGTGAAGCTT | 87599971 | 87599993 | 0.01 |
| GCACCCAGACACTGACACTGA | 87599980 | 87600002 | 2.06 |
| GCACTGACACTGATGGCTTCA | 87599988 | 87600010 | 0.28 |
| GCTGATGGCTTCATGGTTGTG | 87599996 | 87600018 | 0.31 |
| GGTCTGACTCAAGGTCTGGA | 87600029 | 87600051 | 0.00 |
| GTTAGGGTCTGACTCAAGGTC | 87600033 | 87600055 | 0.70 |
| GAGCTTTAGGGTCTGACTCA | 87600038 | 87600060 | 0.01 |
| GCGCACTCACCGAGAGCTTT | 87600051 | 87600073 | 0.02 |
| GCAACCTGAGAACTTTATCA | 87600072 | 87600094 | 0.02 |
| GTATTTATTTAGTTTATTTAT | 87600137 | 87600159 | 3.59 |
| GTCTCTCCAGCCCATCAGGGG | 87600299 | 87600321 | 0.09 |
| GTAAAACCTCCCCTGATGGGC | 87600304 | 87600326 | 0.47 |
| GCTTTGCTTTCATGGTATCTA | 87600332 | 87600354 | 0.01 |
| GAATAATAAACTTTGCTTTCA | 87600341 | 87600363 | 0.01 |
| GTGTGTCCTGCGAATTGTA | 87600395 | 87600416 | 0.05 |
| GCATTGTGCCATACAATTCGC | 87600401 | 87600423 | 0.04 |
| GGCACAATGCATATCACCAC | 87600415 | 87600437 | 0.00 |
| GCTCTGACATGCACATACCTG | 87600431 | 87600453 | 0.19 |
| GATGTGCATGTCAGAGGACAG | 87600439 | 87600461 | 0.40 |
| GGCCTGAAAACTGTACCAAA | 87600460 | 87600482 | 0.66 |
| GAAAACTGTACCAAAAGGTCA | 87600466 | 87600488 | 0.34 |
| GCAGCACGATCCGTGACCTTT | 87600475 | 87600497 | 0.66 |
| GTCACGGATCGTGCTGAGTGC | 87600483 | 87600505 | 0.50 |
| GCAGGGTGACCCCAACACAT | 87600501 | 87600523 | 0.02 |
| GAATCAGTGCAAAAAGTAAG | 87600535 | 87600557 | 0.01 |
| GCACTGATTCCTGGCTCCACG | 87600549 | 87600571 | 0.00 |
| GTGGCGAGCTGTAGCTTACC | 87600587 | 87600609 | 0.02 |
| GCTCGCCACAGACTTCCAGC | 87600601 | 87600623 | 0.01 |
| GAAGGAGAGGATGATTCCTGC | 87600616 | 87600638 | 0.41 |
| GACCCAGAAGGTGGAAGGAG | 87600630 | 87600652 | 0.02 |
| GAGCATTAAGACCCAGAAGG | 87600639 | 87600661 | 1.48 |
| GATGGAGCATTAAGACCCAGA | 87600642 | 87600664 | 0.58 |

| | | | |
|---|---|---|---|
| GGCCCAACCTTTAACTGCTG | 87600665 | 87600687 | 0.01 |
| GATGCCTCAGCAGTTAAAGGT | 87600668 | 87600690 | 0.03 |
| GTTTAACTGCTGAGGCATCTC | 87600674 | 87600696 | 3.75 |
| GAGTTTCCTCTGTTTAGCCC | 87600719 | 87600741 | 0.01 |
| GGTCAACAGAGCAAGTTCCA | 87600745 | 87600767 | 0.01 |
| GACCTTGAATTCCTGAGTTT | 87600773 | 87600795 | 0.81 |
| GTTCCTGAGTTTTGGGAATAA | 87600782 | 87600804 | 0.00 |
| GAAATGCCTGCATGCCAGAAG | 87600871 | 87600893 | 0.05 |
| GCATCTATAATTTCATTTCCA | 87600903 | 87600925 | 0.04 |
| GAAATTATAGATGGTCATG | 87600913 | 87600934 | 0.00 |
| GATAGATGGTCATGTGGATAT | 87600919 | 87600941 | 0.00 |
| GCTGCTCTTTGTAGAGGATC | 87600951 | 87600973 | 6.63 |
| GGACTGGCTGCTCTTTGTAG | 87600957 | 87600979 | 0.01 |
| GGCTTGATAGTTAAGAGGAC | 87600973 | 87600995 | 0.02 |
| GAGATGGCTTGATAGTTAAG | 87600978 | 87601000 | 0.01 |
| GGTAGGTGGGTCTAGAGAGA | 87600994 | 87601016 | 0.03 |
| GTCACGAGAATGAAGGTAGGT | 87601007 | 87601029 | 0.01 |
| GTTCCAATTCACGAGAATGA | 87601015 | 87601037 | 0.01 |
| GACACTGATAAAATTTATAC | 87601051 | 87601073 | 0.14 |
| GCTCTAAAATGCTTGGTTAGC | 87601073 | 87601095 | 0.03 |
| GCTTGGTTAGCAGGGCATGG | 87601082 | 87601104 | 0.01 |
| GCTGGAACTTTGAGAACAGTT | 87601152 | 87601174 | 0.00 |
| GAAAGATGGTGTCTTGATATG | 87601176 | 87601198 | 1.62 |
| GTTTTTTTAAAGAATAAAAGA | 87601191 | 87601213 | 0.03 |
| GAAAAAAAAAAGCCTAGACAT | 87601214 | 87601236 | 0.05 |
| GAAAAAGCCTAGACATTGGGC | 87601219 | 87601241 | 0.02 |
| GTTAGCCCCAGCCCAATGTCT | 87601225 | 87601247 | 0.01 |
| GCATTGGGCTGGGGCTAAGAG | 87601231 | 87601253 | 0.00 |
| GGGCTAAGAGTGGCTTTCAG | 87601241 | 87601263 | 0.11 |
| GACACACAAATTAAAATAAAA | 87601330 | 87601352 | 0.03 |
| GAAAAAGGCAATAAATATGTT | 87601346 | 87601368 | 0.03 |
| GCACCAAAAGCCGGGCGGTGG | 87601510 | 87601532 | 0.01 |
| GAAAAGCCACCAAAAGCCGGG | 87601516 | 87601538 | 6.80 |
| GTAATACTCTAGCATAGCATC | 87601541 | 87601563 | 0.03 |
| GCATAGCATCAGGCAACTTGA | 87601552 | 87601574 | 0.16 |
| GTTTATGTAGCTGCACTTGGA | 87601596 | 87601618 | 0.68 |
| GACATAAATTCTAATCTCTGC | 87601612 | 87601634 | 0.02 |
| GCAGTGCCAGTTTCTGTAGAT | 87601637 | 87601659 | 0.01 |
| GCTGCTTTGCTTAGCAACTGT | 87601657 | 87601679 | 0.00 |
| GAACTGTAGGGAAATCTCCTT | 87601671 | 87601693 | 0.02 |
| GATCTCCTTTGGAGAGATTCT | 87601683 | 87601705 | 0.01 |
| GGAGAGATTCTAGGAGTGGT | 87601692 | 87601714 | 0.01 |
| GTGGTAGGGAACAAGACAA | 87601707 | 87601728 | 0.02 |
| GGAGACTCTCACCACGCCC | 87601727 | 87601748 | 1.80 |
| GGCTGCGGCCTCCAGGGCG | 87601738 | 87601759 | 0.01 |
| GCCGGTGAGGCTGCGGCCTCC | 87601744 | 87601766 | 0.57 |
| GTTCTCTCCGGTGAGGCTG | 87601752 | 87601773 | 0.01 |
| GTTCTGCAGTTCTCTCCGGTG | 87601758 | 87601780 | 0.01 |
| GCAATCTTCTGCAGTTCTCTC | 87601763 | 87601785 | 0.07 |
| GAGAACTGCAGAAGATTGCC | 87601768 | 87601790 | 0.01 |
| GCAGAAGATTGCCCGGGAACC | 87601776 | 87601798 | 0.01 |
| GGAACCTGGAATCCAGGAGG | 87601790 | 87601812 | 0.16 |

| | | | |
|---|---|---|---|
| GCCACCCACCTCCTGGATTCC | 87601794 | 87601816 | 0.00 |
| GATCCAGGAGGTGGGTGGAGC | 87601800 | 87601822 | 0.04 |
| GCTTGAATGTTCAAAGAACAA | 87601826 | 87601848 | 0.14 |
| GTCTTTGAACATTCAAGGCCC | 87601833 | 87601855 | 0.01 |
| GTTTAGCAAATTAGGTTCCA | 87601850 | 87601872 | 0.27 |
| GAGCTCTAGTTTAGCAAATT | 87601858 | 87601880 | 0.00 |
| GTTTGGCTTTGAATTTAAACA | 87601894 | 87601916 | 1.66 |
| GCAAAGCCAAAAGACACCCAG | 87601907 | 87601929 | 0.17 |
| GAAAAACCTCTGGGTGTCTTT | 87601912 | 87601934 | 0.01 |
| GAACAACAACAAAAAACCTCT | 87601922 | 87601944 | 0.04 |
| GGGAACATCGCTTAAGAAAC | 87601990 | 87602012 | 0.01 |
| GTTAAGCGATGTTCCCGCCAT | 87601998 | 87602020 | 0.26 |
| GTTCCCGCCATAGGCTTCCT | 87602007 | 87602029 | 0.01 |
| GCCATAGGCTTCCTAGGTAG | 87602013 | 87602035 | 0.33 |
| GGGACTCTGACCCCCTACCT | 87602024 | 87602046 | 0.15 |
| GGCACGGGGGTGAGGGGTGG | 87602044 | 87602066 | 0.13 |
| GCTGAAAGGCACGGGGGTGA | 87602051 | 87602073 | 0.02 |
| GTATAGCGGCTGAAAGGCAC | 87602059 | 87602081 | 0.00 |
| GTTGCGGTATAGCGGCTGAA | 87602065 | 87602087 | 0.02 |
| GCTGGCCATGTTGCGGTATAG | 87602073 | 87602095 | 0.02 |
| GCCTCCTGCTGGCCATGTTG | 87602081 | 87602103 | 0.00 |
| GTATGTAGTGCTGCCTCCTGC | 87602092 | 87602114 | 0.00 |
| GGCAGCACTACATATCAGAA | 87602101 | 87602123 | 0.01 |
| GTTCTTCTTATTTTTTTACGT | 87602134 | 87602156 | 0.01 |
| GATAAGAAGAATTGAAGCCTT | 87602147 | 87602169 | 0.01 |
| GAAGCCTTTGGAGCAGCTCTC | 87602160 | 87602182 | 6.87 |
| GTCTGGTTTCATAATGTTGTC | 87602178 | 87602200 | 0.00 |
| GTTTCATAATGTTGTCTGGGG | 87602183 | 87602205 | 0.01 |
| GTTGTCTGGGGAGGGGGGAG | 87602192 | 87602214 | 0.01 |
| GTTTCTTCTGCTGCCTAAATC | 87602225 | 87602247 | 0.01 |
| GCTGGGAAACCTAAAGCCAGT | 87602244 | 87602266 | 0.02 |
| GAATTATTCCCAACTGGCTTT | 87602252 | 87602274 | 0.01 |
| GCAGCAGAATTATTCCCAAC | 87602259 | 87602281 | 0.01 |
| GTCTGCTGCTTTTTGTGATAG | 87602274 | 87602296 | 0.00 |
| GATTTATTCCCTCATGCGCA | 87602302 | 87602324 | 0.01 |
| GCTCATGCCCATGCGCATGA | 87602310 | 87602332 | 0.01 |
| GAACATGACCACCGTACAAGA | 87602335 | 87602357 | 0.00 |
| GCGCGCATGCCCTTCTTGTA | 87602345 | 87602367 | 0.01 |
| GAGGAAGTGTGTGTGTGTTG | 87602389 | 87602411 | 0.01 |
| GAAACTCTAGACAGAGGAAG | 87602408 | 87602430 | 0.01 |
| GGAAGAGAAACTCTAGACAG | 87602414 | 87602436 | 0.01 |
| GAGCAAATTTAAAGTGGCTC | 87602435 | 87602457 | 0.15 |
| GTTACAGAGCAAATTTAAAG | 87602441 | 87602463 | 0.18 |
| GTTGCTCTGTAACGCAAGTCT | 87602452 | 87602474 | 0.24 |
| GAAGTCTTGGCTCCCTGTTAA | 87602466 | 87602488 | 0.01 |
| GCTCCCTGTTAAAGGAATGCT | 87602475 | 87602497 | 0.01 |
| GAATGCTAGGGATCAGTGAGT | 87602489 | 87602511 | 0.01 |
| GTAGGGATCAGTGAGTAGGGT | 87602494 | 87602516 | 0.01 |
| GTAGGGAAAACGTTATTTTC | 87602512 | 87602534 | 0.02 |
| GTAGGTATGTTGGGATGGTT | 87602540 | 87602562 | 0.01 |
| GGATTGACCGTAGGTATGTT | 87602549 | 87602571 | 0.01 |
| GTTCAAAGTAGGATTGACCGT | 87602558 | 87602580 | 2.54 |

| | | | |
|---|---|---|---|
| GTCAATCCTACTTTGAAGGC | 87602564 | 87602586 | 0.01 |
| GCTTGACCTGCCTTCAAAGT | 87602570 | 87602592 | 0.04 |
| GGAACTCAGAAATCAGATAC | 87602592 | 87602614 | 0.01 |
| GCTGCTTGTCTTTCTGGATC | 87602613 | 87602635 | 0.01 |
| GTTAGAGCTGCTTGTCTTTC | 87602619 | 87602641 | 0.05 |
| GACAAGCAGCTCTAACTGC | 87602626 | 87602647 | 0.01 |
| GCAGGCCCAGTCTCCCTGCC | 87602643 | 87602665 | 0.01 |
| GGGATCCAGGCAGGGAGACT | 87602648 | 87602670 | 0.02 |
| GACGGTGGGGGGATCCAGGCA | 87602656 | 87602678 | 0.01 |
| GATTCCACGGTGGGGGGATCC | 87602661 | 87602683 | 0.02 |
| GCTCGGTGAGATTCCACGGT | 87602671 | 87602693 | 0.06 |
| GGGAGCTCGGTGAGATTCCA | 87602675 | 87602697 | 0.03 |
| GTACTGAGAAGAGTGGGAGCT | 87602688 | 87602710 | 0.02 |
| GCAGTTAGTACTGAGAAGAGT | 87602695 | 87602717 | 0.02 |
| GACTAACTGTAGAGACTTTCC | 87602710 | 87602732 | 0.01 |
| GAGCTCTCTGACTACAGGCC | 87602728 | 87602750 | 0.32 |
| GACTTCTTTGGCATTTATGT | 87602762 | 87602784 | 0.14 |
| GATGCCAAAGAAGTCCCTGTG | 87602771 | 87602793 | 0.65 |
| GCAAAGACAGCATCCCGCAC | 87602785 | 87602807 | 0.01 |
| GCTGTCTTTGCCCTGCCCCAA | 87602798 | 87602820 | 3.06 |
| GTTGGACATGCCCATTGGGGC | 87602808 | 87602830 | 0.01 |
| GTAGCTTGGACATGCCCATT | 87602813 | 87602835 | 0.10 |
| GGCATGTCCAAGCTACTGTT | 87602820 | 87602842 | 0.00 |
| GAAGTCTCCAAACAGTAGCT | 87602827 | 87602849 | 0.01 |
| GTTGGAGACTTCGAGTTCACC | 87602839 | 87602861 | 0.01 |
| GCAAAGGGCAGAGGGAGGGCC | 87602857 | 87602879 | 0.01 |
| GAAGGTTCAAAGGGCAGAGGG | 87602863 | 87602885 | 0.62 |
| GCAGGCTCCAGGAAGGTTCAA | 87602874 | 87602896 | 0.04 |
| GAAAAGCCACAGGCTCCAGGA | 87602882 | 87602904 | 0.07 |
| GAAGAACCAGAGAAAAGCCAC | 87602893 | 87602915 | 1.76 |
| GCTTTTCTCTGGTTCTTGGA | 87602899 | 87602921 | 0.01 |
| GTCTGGTTCTTGGATGGTTAA | 87602906 | 87602928 | 0.02 |
| GGTGAGGAAAGCAGGCGGAA | 87602936 | 87602958 | 0.03 |
| GCCAACGGTGAGGAAAGCAGG | 87602941 | 87602963 | 0.22 |
| GACCGTTGGCCTCTGTGTCTC | 87602956 | 87602978 | 0.01 |
| GTTGGCCTCTGTGTCTCAGG | 87602959 | 87602981 | 0.00 |
| GTGTCTCAGGTGGCAGGGTG | 87602969 | 87602991 | 0.94 |
| GCAGGGTGTGGTCCCAGCTAG | 87602982 | 87603004 | 0.07 |
| GTTTCATTTAGGCCTCTAGCT | 87602993 | 87603015 | 0.01 |
| GTTTGTCTTTCCTTTTCATTT | 87603005 | 87603027 | 0.01 |
| GATCGTCATGAATGATGAAT | 87603068 | 87603090 | 0.00 |
| GAATGGTCACATTCATTTGT | 87603090 | 87603112 | 0.02 |
| GACCATTCACATTTTAACAA | 87603105 | 87603127 | 0.05 |
| GGGGAGCCCCCACACCCCA | 87603125 | 87603146 | 0.01 |
| GTTCAATGCCTTGGGGTGTG | 87603132 | 87603154 | 0.27 |
| GATGTACAGTTCAATGCCTTG | 87603139 | 87603161 | 0.02 |
| GATTGAACTGTACATGTACAG | 87603148 | 87603170 | 3.47 |
| GCTGATTTAGTCTCTAAAAGC | 87603174 | 87603196 | 2.22 |
| GCTAGTATCTATCTACCCAG | 87603196 | 87603218 | 0.01 |
| GATCTACCCAGAGGGCAGCTG | 87603206 | 87603228 | 0.50 |
| GCCTACCACAGCTGCCCTCT | 87603211 | 87603233 | 0.56 |
| GGCAGCTGTGGTAGGCTGGC | 87603218 | 87603240 | 0.88 |

| | | | |
|---|---|---|---|
| GTAGGCTGGCCGGCTGTGGT | 87603228 | 87603250 | 0.34 |
| GAGGGCTGCCCTACCACAGC | 87603237 | 87603259 | 0.11 |
| GAGAAGGAAACGGTGGTTGG | 87603259 | 87603281 | 0.08 |
| GCTGAGAAGGAAACGGTGGT | 87603262 | 87603284 | 0.01 |
| GGTCACTGCATGAGCTGAGA | 87603275 | 87603297 | 0.02 |
| GCATGCAGTGACCTCACTCTC | 87603286 | 87603308 | 0.01 |
| GTCACTCTCTGGTCTGTTCAC | 87603298 | 87603320 | 0.07 |
| GCTCTCCCACGAAGCCTTTG | 87603320 | 87603342 | 0.08 |
| GACGAACCTCAAAGGCTTCG | 87603326 | 87603348 | 0.53 |
| GCGAAGCTGACGAACCTCAA | 87603334 | 87603356 | 9.12 |
| GTGTACACTCGGAGCGGCTG | 87603356 | 87603378 | 0.09 |
| GTGTACACTGGCCTGAGTTC | 87603371 | 87603393 | 0.24 |
| GAGGTAGCTCCCCAGAACTC | 87603382 | 87603404 | 1.55 |
| GCTGGGGAGCTACCTCAGCTC | 87603390 | 87603412 | 1.11 |
| GGAGAGAAAACCAGAGCTG | 87603401 | 87603422 | 0.02 |
| GTCTGGTTTTCTCTCCATCCA | 87603408 | 87603430 | 0.01 |
| GTTTCTCTCCATCCATGGGCG | 87603414 | 87603436 | 0.02 |
| GGCCTTGGGCCCCACGCCCA | 87603425 | 87603447 | 1.34 |
| GAAGTGAGGTATCATGGCCTT | 87603439 | 87603461 | 0.00 |
| GCCATGATACCTCACTTTTA | 87603445 | 87603467 | 0.01 |
| GATACCTCACTTTTATGGTG | 87603450 | 87603472 | 0.42 |
| GCGGATAACACCTTTGTCCT | 87603469 | 87603491 | 0.80 |
| GCCAGAAAGTTCCTAGGACAA | 87603479 | 87603501 | 0.25 |
| GGTGGAACCAGAAAGTTCCT | 87603486 | 87603508 | 0.02 |
| GAGGTACTGGAAGGAGTGGG | 87603504 | 87603526 | 0.01 |
| GAAGCAAGGAGAGGTACTGGA | 87603513 | 87603535 | 0.01 |
| GTACCTCTCCTTGCTTTTCT | 87603520 | 87603542 | 23.62 |
| GCTTTTCTCGGGGACATGAA | 87603532 | 87603554 | 0.03 |
| GCAGCATGCAGCGTTGCTCAA | 87603558 | 87603580 | 0.32 |
| GAGCAACGCTGCATGCTGGG | 87603563 | 87603585 | 0.03 |
| GAGAAGTGAATAGCAGGAGC | 87603605 | 87603627 | 0.00 |
| GCACACTGAGAAGTGAATAGC | 87603611 | 87603633 | 0.02 |
| GTATATAAGCACAGACTCAAC | 87603639 | 87603661 | 2.12 |
| GCCACGAATGGCTGGGAGTTC | 87603658 | 87603680 | 0.02 |
| GAGATGGGTCCACGAATGGC | 87603667 | 87603689 | 0.05 |
| GCCTGTGGAAGCCAAAGAGAT | 87603682 | 87603704 | 0.02 |
| GGCTTCCACAGGAACCCTGA | 87603693 | 87603715 | 0.01 |
| GACAGGAACCCTGAAGGTAGG | 87603700 | 87603722 | 0.02 |
| GAACCCTGAAGGTAGGAGGGA | 87603705 | 87603727 | 0.01 |
| GAAGGCTTGCCATTCTGTAAA | 87603724 | 87603746 | 0.07 |
| GTTGCCATTCTGTAAAAGGGC | 87603729 | 87603751 | 0.01 |
| GTAAAAGGGCAGGCAGGCTAA | 87603740 | 87603762 | 2.47 |
| GGCAGGCTAAAGGAACAGCC | 87603750 | 87603772 | 0.10 |
| GTTTCTTCTGCGGAGAAGGCC | 87603768 | 87603790 | 0.87 |
| GGCTATTTCTTCTGCGGAGA | 87603773 | 87603795 | 0.01 |
| GCACATGAGTGAATACTGGCC | 87603794 | 87603816 | 0.19 |
| GAAGTCACATGAGTGAATAC | 87603799 | 87603821 | 0.03 |
| GACTTCTATCTGTGACTGAGA | 87603817 | 87603839 | 4.33 |
| GTGACTGAGAAGGCCTTTCC | 87603827 | 87603849 | 0.40 |
| GACTGCAGCTTCCCTGGAA | 87603840 | 87603861 | 1.74 |
| GACTACAGACTGCAGCTTCCC | 87603845 | 87603867 | 0.05 |
| GTCAAATTCGTTGTCTGAAAC | 87603874 | 87603896 | 0.01 |

| | | | |
|---|---|---|---|
| GCAACGAATTTGAGGCCAGCC | 87603885 | 87603907 | 0.00 |
| GTGAGACAGGATCTCATGT | 87603908 | 87603929 | 0.07 |
| GCCTCTCTGTTTTTGTGAGAC | 87603920 | 87603942 | 0.00 |
| GCTCAGAACCCAGAGAAAGG | 87603951 | 87603973 | 0.04 |
| GCCAGACATCCTCCTTTCTCT | 87603959 | 87603981 | 0.01 |
| GCCCTGATCTGATCATTATC | 87604000 | 87604022 | 0.10 |
| GTAGTATCTCATATAGCCC | 87604087 | 87604108 | 0.01 |
| GACTAGAGAGTTAGGAGTTCA | 87604113 | 87604135 | 0.01 |
| GGCTTCTGACTAGAGAGTT | 87604122 | 87604143 | 0.21 |
| GTCAGAAGCCTTATGAGTTC | 87604134 | 87604156 | 0.01 |
| GCTATGATCCCAGAACTCATA | 87604142 | 87604164 | 0.19 |
| GACGGTAGTCAGGTGATAG | 87604173 | 87604194 | 0.01 |
| GTCTTGTATATGACGGTAGTC | 87604182 | 87604204 | 0.03 |
| GATTTTAGTTCTTGTATATGA | 87604190 | 87604212 | 0.01 |
| GATGTGTCAGCCAACAAATAA | 87604244 | 87604266 | 0.01 |
| GTCTTGAAAGCCTTTATTTGT | 87604253 | 87604275 | 0.92 |
| GCAAGAACCTCCGTATGTAGC | 87604271 | 87604293 | 0.01 |
| GATCAGTCCTGCTACATACGG | 87604277 | 87604299 | 0.49 |
| GTATGTAGCAGGACTGATGT | 87604282 | 87604304 | 0.01 |
| GCTGATGTGGGGCTAGAGCGC | 87604295 | 87604317 | 0.45 |
| GGCTAGAGCGCTGGCTCAGC | 87604304 | 87604326 | 0.01 |
| GTGTGCTGGGCACTGAACTC | 87604334 | 87604356 | 0.12 |
| GTTCAGTGCCCAGCACACACA | 87604340 | 87604362 | 0.03 |
| GAATTGCCCCATGTGTGTGC | 87604348 | 87604370 | 0.01 |
| GCCTGGAGCTGGAGCTATCGA | 87604375 | 87604397 | 0.01 |
| GAGGACAGATCCCCTGGAGC | 87604387 | 87604409 | 1.73 |
| GTGTGGAGGACAGATCCCC | 87604393 | 87604414 | 0.01 |
| GCCCATGAGCATGGGTGTGTG | 87604409 | 87604431 | 0.02 |
| GTGTTTGTTCCCATGAGCAT | 87604418 | 87604440 | 0.01 |
| GAAAAAATCCAGTAGTGTT | 87604479 | 87604500 | 0.01 |
| GATCCAGTAGTGTTAGGAGTA | 87604485 | 87604507 | 0.30 |
| GTGTTAGGAGTATGGCTCAG | 87604493 | 87604515 | 0.00 |
| GTAGTACTGGATTCAAACTGA | 87604534 | 87604556 | 2.97 |
| GTTTCTTTTTTTCTGTAGTAC | 87604548 | 87604570 | 0.00 |
| GAAAGAAAAAAAAGATTAAG | 87604571 | 87604593 | 0.64 |
| GTAAAACAAATCTTCTAAGC | 87604610 | 87604632 | 0.04 |
| GTTTTACTGAGAGCAGAATA | 87604626 | 87604648 | 0.02 |
| GCAGAATAAGGAGGTTCTCT | 87604638 | 87604660 | 0.02 |
| GGAGGTTCTCTGGGTAGCTC | 87604647 | 87604669 | 0.03 |
| GATCTCTGAGGGTAAATTCT | 87604674 | 87604696 | 0.02 |
| GTTCTTTTAATCTCAGCACTC | 87604712 | 87604734 | 0.03 |
| GACTTTTAGGGCTAGTGAAA | 87604766 | 87604788 | 0.18 |
| GTCTTTAGATCACATTACAGA | 87604865 | 87604887 | 0.01 |
| GATAAATAAAACTTAAAAAAA | 87604915 | 87604937 | 0.03 |
| GAAAAAAATGGCTTTTAATGT | 87604928 | 87604950 | 0.03 |
| GCTTTTAATGTAGGGCATAA | 87604937 | 87604959 | 0.01 |
| GTAGGGCATAATGGTGCAC | 87604946 | 87604967 | 0.42 |
| GGTGCACAGGAGGCAGAGGC | 87604958 | 87604980 | 0.03 |
| GGCAGACTGCTATGAGTTTG | 87604979 | 87605001 | 0.01 |
| GAATCACTATGTAGATCTGGC | 87605002 | 87605024 | 0.00 |
| GACATAGTGATTTATAGCCCA | 87605014 | 87605036 | 0.02 |
| GTTTATAGCCCATGGAGAAAC | 87605023 | 87605045 | 0.02 |

| | | | |
|---|---|---|---|
| GTTCAGACCCAGTTTCTCCAT | 87605030 | 87605052 | 0.20 |
| GCAAAATATCTCCTCCATGAG | 87605085 | 87605107 | 4.13 |
| GGACGTGCCTCCGCTCATGG | 87605095 | 87605117 | 1.89 |
| GACGCAGATGGGCAGCTGTTT | 87605116 | 87605138 | 2.72 |
| GTCTCAGGACTGAACGCAGA | 87605129 | 87605151 | 0.01 |
| GCGTTCAGTCCTGAGACTC | 87605135 | 87605156 | 0.01 |
| GTCCTGAGACTCAGGTGACA | 87605142 | 87605164 | 0.63 |
| GACTCAGGTGACAAGGGGG | 87605149 | 87605170 | 0.01 |
| GGGGGAGGACAGACTCCTA | 87605163 | 87605184 | 0.01 |
| GAGGTCAAATGACAGTCCGT | 87605178 | 87605200 | 0.01 |
| GGTATGCATGTGTTACTGTG | 87605200 | 87605222 | 0.03 |
| GTATTTATTTTGTATGGGTTG | 87605221 | 87605243 | 0.01 |
| GTTTGCTTATTTATTTTGTAT | 87605227 | 87605249 | 0.01 |
| GAACAATAAAATCATTAATTA | 87605253 | 87605275 | 0.02 |
| GTAAAATCATTAATTAAGGGG | 87605258 | 87605280 | 0.07 |
| GTAATTAAGGGGCGGGAGAGA | 87605267 | 87605289 | 0.02 |
| GGGCGGGAGAGACGGCTCAT | 87605275 | 87605297 | 0.07 |
| GTATTAAAAATCAATTTTTAT | 87605462 | 87605484 | 0.01 |
| GTGGGAGTGGGTGGATAGTG | 87605640 | 87605662 | 0.03 |
| GTGGGTGGATAGTGGGGTGG | 87605646 | 87605668 | 0.12 |
| GTTTAAAACGTTAATTAAAGC | 87605751 | 87605773 | 0.02 |
| GACGTTAATTAAAGCTGGGCG | 87605757 | 87605779 | 0.01 |
| GTATCCTGGAACTCACTTGGT | 87605857 | 87605879 | 0.04 |
| GCAAAAAAGACAAGCTGTATG | 87605934 | 87605956 | 0.02 |
| GTCTCAGCATTCCAGAGGCAC | 87605966 | 87605988 | 0.04 |
| GCAGCTGCCTGCCTGTGCCTC | 87605976 | 87605998 | 0.01 |
| GTCTGGTCTACAGGACAACC | 87606021 | 87606043 | 0.04 |
| GAAAGCCAAATACATAAAGGA | 87606068 | 87606090 | 0.09 |
| GTAGTTCAACTTAGTCTACAA | 87606230 | 87606252 | 0.03 |
| GTAGTCTACAAAGGAAGTTCC | 87606240 | 87606262 | 0.08 |
| GTAACCCTGGCTGGCTGGCC | 87606258 | 87606280 | 0.01 |
| GATTGAGTAACCCTGGCTGGC | 87606263 | 87606285 | 0.62 |
| GAAGGTCTCATTGAGTAACCC | 87606271 | 87606293 | 0.00 |
| GTGGTTTTCTTTTTGAAACA | 87606290 | 87606312 | 0.01 |
| GTGTGTGCTATTTGTTTTG | 87606309 | 87606330 | 0.01 |
| GAATAGTTTCTCTCTTTTTAA | 87606362 | 87606384 | 0.01 |
| GATTTGTCAATGCATCCGTGA | 87606382 | 87606404 | 0.00 |
| GAAGCATATTCAAACCATCA | 87606396 | 87606418 | 0.37 |
| GTTTGAGAAAACTTCCACCTA | 87606486 | 87606508 | 0.02 |
| GAACTTCCACCTAAGGTCCTG | 87606494 | 87606516 | 1.77 |
| GACTGGCTCCTCAGGACCTT | 87606502 | 87606524 | 0.07 |
| GGTCCTGAGGAGCCAGTCTT | 87606507 | 87606529 | 1.54 |
| GGAGCCAGTCTTAGGGCCCA | 87606515 | 87606537 | 16.15 |
| GATGGAAAGCAGGCCTGGCCC | 87606532 | 87606554 | 0.01 |
| GATCATGATGGAAAGCAGGCC | 87606538 | 87606560 | 0.18 |
| GATATCATCATGATGGAAAGC | 87606543 | 87606565 | 0.09 |
| GTTCAATCCATATCATCATGA | 87606551 | 87606573 | 0.15 |
| GATGGATTGAACTTCAGAGCC | 87606564 | 87606586 | 0.01 |
| GATGAGGGAAATATTTAATTG | 87606594 | 87606616 | 2.50 |
| GACCAAGGCAGCTGCTATGA | 87606610 | 87606632 | 0.01 |
| GTTGCTTCGGTTGCTGTCTT | 87606660 | 87606682 | 0.02 |
| GACAGCAACCGAAGCAACCT | 87606665 | 87606687 | 0.01 |

| | | | |
|---|---|---|---|
| GCAACCGAAGCAACCTGGGTT | 87606670 | 87606692 | 0.46 |
| GTAAGCCATTGCCCTAACCC | 87606682 | 87606704 | 0.03 |
| GTACCTGCCCTTAGAAAAAGA | 87606702 | 87606724 | 0.01 |
| GTATTTTCCTTCTTTTTCTA | 87606709 | 87606731 | 0.01 |
| GCCAGTTGGAGCTGGGAAAAT | 87606731 | 87606753 | 0.64 |
| GTTCCCAGCTCCAACTGGGGC | 87606737 | 87606759 | 0.01 |
| GGTCAGCCTCCAGCCCCAGT | 87606746 | 87606768 | 0.01 |
| GGTTGGTGTGAACTTTACTC | 87606767 | 87606789 | 1.95 |
| GAAGTCTTGCTCTTTGTTCA | 87606788 | 87606810 | 0.02 |
| GAATAAGATGAATAACCATCC | 87606823 | 87606845 | 0.01 |
| GATGAATAACCATCCAGGGGC | 87606829 | 87606851 | 0.01 |
| GCCATCTTACCAGCCCCTGGA | 87606837 | 87606859 | 0.01 |
| GTAAGATGGGTGTACTGATGA | 87606852 | 87606874 | 0.55 |
| GAATCACACTTGAGTCATA | 87606889 | 87606910 | 0.10 |
| GTGATTCTTGAACCCCACA | 87606904 | 87606925 | 0.43 |
| GCTTGAACCCCACATGGTAGA | 87606910 | 87606932 | 0.52 |
| GGCATCAGAGGACAACTTAC | 87606945 | 87606967 | 0.01 |
| GTTGTGGGGAGAGGGCATCAG | 87606957 | 87606979 | 0.01 |
| GATTTATGTTTGTGGGGAGA | 87606966 | 87606988 | 0.02 |
| GTTTTTAGATTTATGTTTGTG | 87606972 | 87606994 | 0.02 |
| GTTTTCTTTTAATTTTTTTGT | 87607019 | 87607041 | 0.01 |
| GATTAAGATCAAAATACAAAC | 87607043 | 87607065 | 0.09 |
| GCTTAATTCTGCTTCCTCGAG | 87607060 | 87607082 | 0.01 |
| GTTGGGGGCAGCCCCTCTCG | 87607073 | 87607095 | 2.55 |
| GGGCTGCCCCCAACCAGGGG | 87607082 | 87607104 | 0.01 |
| GTAAGTGATCCACCCCTGGT | 87607091 | 87607113 | 2.08 |
| GCTGTGTAAGTGATCCACCCC | 87607095 | 87607117 | 0.70 |
| GACCAGTCAGCATAGGGAGA | 87607132 | 87607154 | 0.01 |
| GTTATTTGACCAGTCAGCATA | 87607138 | 87607160 | 0.01 |
| GCTAGAGCCTCTGATTAGGC | 87607163 | 87607185 | 0.52 |
| GCCTCTGATTAGGCAGGGGA | 87607169 | 87607191 | 0.01 |
| GTAGGCAGGGGAAGGGAAAGA | 87607178 | 87607200 | 0.01 |
| GGGGAAGGGAAAGATGGAAC | 87607184 | 87607206 | 0.05 |
| GAAGATGGAACGGGATGTCTG | 87607194 | 87607216 | 0.03 |
| GGAACGGGATGTCTGAGGGA | 87607199 | 87607221 | 0.01 |
| GAAAAGTGGAGTAGAACCACA | 87607370 | 87607392 | 10.51 |
| GCATGGCCTACAGAAGCCACA | 87607388 | 87607410 | 0.01 |
| GACCCCTTGTGGCTTCTGT | 87607393 | 87607414 | 1.18 |
| GCCACAAGGGGTCTCATAGC | 87607402 | 87607424 | 0.03 |
| GAAATGTAGTAACTGAGTTGT | 87607476 | 87607498 | 0.01 |
| GACTGAGTTGTTGGGTCTATA | 87607486 | 87607508 | 0.01 |
| GGGTCTATATGGGCACTTTG | 87607497 | 87607519 | 0.49 |
| GCATGAAAAGCAGAAAAAAAG | 87607549 | 87607571 | 0.01 |
| GAAAAAGTGGTTTGTTCAATG | 87607563 | 87607585 | 0.01 |
| GTTTGTTCAATGTGGCTGCAT | 87607572 | 87607594 | 0.01 |
| GAATGTGGCTGCATTGGGAAG | 87607579 | 87607601 | 0.03 |
| GCATTGGGAAGAGGTACAGAG | 87607589 | 87607611 | 0.00 |
| GAAGAGGTACAGAGAGGGCC | 87607595 | 87607617 | 0.01 |
| GGTGAATTTTCAGGGTTCCC | 87607613 | 87607635 | 0.03 |
| GAAAAGGAAGGTGAATTTTCA | 87607621 | 87607643 | 0.02 |
| GTAAGCACTTGTTTAAAAGGA | 87607634 | 87607656 | 3.16 |
| GAACAAGTGCTTAGAGAGCTG | 87607645 | 87607667 | 0.31 |

| | | | |
|---|---|---|---|
| GAGAGCTGAGGAGACTCAAT | 87607657 | 87607679 | 0.97 |
| GACTCAATTGGTGTTTGCCT | 87607669 | 87607691 | 0.67 |
| GCAAGGACATTGTGCATCCC | 87607686 | 87607708 | 1.20 |
| GCAATGCTGCAGATGGAAGCA | 87607703 | 87607725 | 1.66 |
| GCTTTTTGGCAATGCTGCAGA | 87607711 | 87607733 | 0.00 |
| GATCACAATTAAAACTCTTTT | 87607726 | 87607748 | 0.01 |
| GTTAATTGTGATAATAAACAG | 87607738 | 87607760 | 0.03 |
| GATAATAAACAGAGGGAGC | 87607746 | 87607767 | 0.03 |
| GTCTCAATAGCTAAGAGTGT | 87607774 | 87607796 | 0.01 |
| GAAGAGTGTTGGCTCTCCCAG | 87607786 | 87607808 | 0.19 |
| GTTGGCTCTCCCAGAGGACC | 87607792 | 87607814 | 0.04 |
| GGTGTTGAGCACCCTGGAAC | 87607861 | 87607883 | 1.35 |
| GTAAGGGTGTTGAGCACCC | 87607867 | 87607888 | 0.03 |
| GCTGCATACATGTTTGTGTAA | 87607882 | 87607904 | 0.00 |
| GATTTTATTTTATACATAT | 87607913 | 87607934 | 0.07 |
| GTCATTTTTTTAAAAAATGGG | 87607933 | 87607955 | 0.03 |
| GTTAAAAAATGGGAGGTAGTT | 87607941 | 87607963 | 0.02 |
| GGGAGGTAGTTTGGATATAT | 87607950 | 87607972 | 0.01 |
| GATGACCTACAAGCCAAGAAA | 87607990 | 87608012 | 0.02 |
| GAGACTTCATGCCCTTTTCT | 87608002 | 87608024 | 0.07 |
| GAAAGGGCATGAAGTCTCTTT | 87608008 | 87608030 | 0.01 |
| GCTTTAGGGCCTTGAAAAGAG | 87608024 | 87608046 | 0.50 |
| GCTGACAGGCCTCTCTTTTCA | 87608032 | 87608054 | 0.01 |
| GAGAGGCCTGTCAGTGCCT | 87608041 | 87608062 | 0.01 |
| GCCTGTCAGTGCCTTGGTTT | 87608046 | 87608068 | 0.00 |
| GTGCCTTGGTTTAGGGCATC | 87608054 | 87608076 | 0.01 |
| GTCTCTTATGGTTGAGAAGGC | 87608073 | 87608095 | 0.01 |
| GAAAGGGAATTTACTCTCTTA | 87608086 | 87608108 | 0.01 |
| GGATAGTTGCCGGGCAGTGG | 87608255 | 87608277 | 0.02 |
| GACAAGCCTGGATAGTTGCC | 87608264 | 87608286 | 0.04 |
| GAAAGGCACTCACGACAAGCC | 87608276 | 87608298 | 0.01 |
| GACTGAATATGATATAGGAAA | 87608294 | 87608316 | 0.01 |
| GCACCACTGAATATGATAT | 87608300 | 87608321 | 0.03 |
| GTATCCATTTTATACTAGGCC | 87608333 | 87608355 | 0.01 |
| GATTTTATACTAGGCCTGGCC | 87608338 | 87608360 | 0.01 |
| GCCAGGCAAAATCTGCTCAT | 87608355 | 87608377 | 2.06 |
| GAAAATCTGCTCATTGGTGCA | 87608362 | 87608384 | 0.01 |
| GGGCAGCAAGTCTGTTTAT | 87608383 | 87608404 | 0.01 |
| GGAGAAGCCAACTCCACAGA | 87608403 | 87608425 | 0.02 |
| GCTGAATTCCCTCTGTGGAGT | 87608410 | 87608432 | 0.04 |
| GCCAGATCTGAATTCCCTCTG | 87608416 | 87608438 | 0.04 |
| GTCAGATCTGGTATTGTAAAC | 87608429 | 87608451 | 0.03 |
| GTAAACCGGGTCCGAAGCCCA | 87608444 | 87608466 | 0.01 |
| GGGTCCGAAGCCCATGGCTG | 87608450 | 87608472 | 0.00 |
| GGGTATGACCCCACAGCCAT | 87608460 | 87608482 | 0.02 |
| GCATTTAGGAAAACACGTGAA | 87608480 | 87608502 | 0.01 |
| GCTTTGACATCATGCCCATTT | 87608495 | 87608517 | 0.30 |
| GGAGACAGACATTTTCTGAA | 87608520 | 87608542 | 0.02 |
| GAAATGTCTGTCTCCATGGGT | 87608529 | 87608551 | 0.66 |
| GCTGACAGTAGGACCAACCCA | 87608541 | 87608563 | 0.02 |
| GCTTTTAGAAAAGCTGACAGT | 87608553 | 87608575 | 0.01 |
| GAATGTGTATAACATAAGAG | 87608653 | 87608675 | 2.39 |

| | | | |
|---|---|---|---|
| GCAGAAGCCCATCAGATCCCC | 87608677 | 87608699 | 0.02 |
| GCGGTTCCTGGGGATCTGAT | 87608683 | 87608705 | 0.01 |
| GATCCCCAGGAACCGCAGCTA | 87608691 | 87608713 | 0.00 |
| GCTCACATCCACCCATAGCTG | 87608702 | 87608724 | 0.31 |
| GGATGTGAGTCACCTGAGAC | 87608716 | 87608738 | 0.29 |
| GAGTCACCTGAGACGGGTGT | 87608722 | 87608744 | 0.48 |
| GGTTCCCAACACCCGTCTC | 87608728 | 87608749 | 0.06 |
| GAAGCTAAAGGTAGCACTGGC | 87608804 | 87608826 | 0.01 |
| GTTTCTTTGAGCAAAGCTAA | 87608817 | 87608839 | 0.02 |
| GCACACATGGAAACAAAGAAA | 87608839 | 87608861 | 1.16 |
| GAAGGTTTGCGCCACCACACA | 87608853 | 87608875 | 0.12 |
| GTTAATCCCAGCACTCCTAAA | 87608875 | 87608897 | 0.00 |
| GCACTCCTAAATGGGCATGA | 87608884 | 87608906 | 0.02 |
| GCTCTGCCCTCATGCCCATTT | 87608889 | 87608911 | 0.13 |
| GGCATGAGGGCAGAGGCAGG | 87608897 | 87608919 | 0.01 |
| GTGGATCTCTGTTAAGTCCA | 87608916 | 87608938 | 0.01 |
| GTTTGTAGATCTGGATGGCCT | 87608933 | 87608955 | 0.01 |
| GCTTTTTGTTTTTGTAGATC | 87608943 | 87608965 | 0.02 |
| GAACCAGCCTCTATTTGCAGT | 87608968 | 87608990 | 0.01 |
| GCAGCTACCTACTGCAAATAG | 87608974 | 87608996 | 0.01 |
| GTAACTGCACAGACCACAAAC | 87608997 | 87609019 | 0.02 |
| GTTACACAGACATCCTGTTTG | 87609009 | 87609031 | 0.11 |
| GATGTCTGTGTAATCCCTCCA | 87609020 | 87609042 | 0.28 |
| GCTTTATATTGTTACCTTGGA | 87609033 | 87609055 | 0.01 |
| GATTCAGCGTGCGTGTTCTTT | 87609062 | 87609084 | 0.01 |
| GAACACGCACGCTGAATCACA | 87609069 | 87609091 | 0.30 |
| GCACAAGGTGGACTGTTGTAG | 87609085 | 87609107 | 0.04 |
| GTTGTAGAGGTTGGCCTTCC | 87609098 | 87609120 | 0.01 |
| GTGTCCTATGGCCACCTGGA | 87609112 | 87609134 | 0.03 |
| GGCTGTGTCCTATGGCCACC | 87609116 | 87609138 | 0.09 |
| GCGGACAGGCTGTGTCCTA | 87609124 | 87609145 | 1.18 |
| GACACAGCCTGTCCGCATCC | 87609130 | 87609152 | 0.86 |
| GCCTGTCCGCATCCTGGGGT | 87609136 | 87609158 | 0.08 |
| GTCCGCATCCTGGGGTGGGGC | 87609141 | 87609163 | 0.01 |
| GCTCCTCCCTGCCCCACCCC | 87609148 | 87609170 | 0.01 |
| GGACGCCTCCACCCACCCTG | 87609174 | 87609196 | 0.01 |
| GTAGCTCCTCAGGGTGGGTGG | 87609179 | 87609201 | 0.01 |
| GAACCTATAGCTCCTCAGGGT | 87609185 | 87609207 | 0.01 |
| GAGCTATAGGTTGCTGGAAG | 87609196 | 87609218 | 0.01 |
| GGAAGAGGGATTTTCTTTCG | 87609211 | 87609233 | 0.01 |
| GTCTTTCGTGGGGTAGCCATA | 87609224 | 87609246 | 0.00 |
| GCCTGAGCGTGGGCCACCTTA | 87609239 | 87609261 | 0.13 |
| GTGGGTTATTTACCTGAGCG | 87609251 | 87609273 | 0.02 |
| GTCACTCACAGGAACATGGA | 87609273 | 87609295 | 0.81 |
| GTTCCTGTGAGTGACCCTGA | 87609281 | 87609303 | 0.02 |
| GTTCCATCAGGGTCACTCAC | 87609284 | 87609306 | 0.00 |
| GAGGCTCAGTGAGTTCCATC | 87609296 | 87609318 | 0.23 |
| GCACTGAGCCTCACAGAGCAT | 87609308 | 87609330 | 4.99 |
| GCGTGTCCCAATGCTCTGTG | 87609315 | 87609337 | 1.14 |
| GCTGGTTCTGGCTAATTGCTG | 87609362 | 87609384 | 0.22 |
| GAATTAGCCAGAACCAGAAAA | 87609369 | 87609391 | 0.04 |
| GCTGGTCCTTTTTCTGGTTC | 87609375 | 87609397 | 0.01 |

| | | | |
|---|---|---|---|
| GTAGTAGCTGGTCCTTTTTC | 87609381 | 87609403 | 0.01 |
| GAAAGGACCAGCTACTACAGG | 87609387 | 87609409 | 0.30 |
| GTCTCGCCCTCCTGTAGTAGC | 87609393 | 87609415 | 0.01 |
| GACTGGGAACATCTGGGGAAA | 87609553 | 87609575 | 0.01 |
| GTAAAGACTGGGAACATCTG | 87609559 | 87609581 | 0.01 |
| GTCTTTAACCCGGTAAAGACT | 87609570 | 87609592 | 0.01 |
| GACCGGGTTAAAGATGCGCCA | 87609580 | 87609602 | 0.06 |
| GCCATGGTCACGTGACGTGA | 87609596 | 87609618 | 0.16 |
| GGTCACGTGACGTGAGGGGC | 87609601 | 87609623 | 1.86 |
| GGGGCTGGCAGCCGTGTTG | 87609616 | 87609637 | 0.04 |
| GAGCTGATGTTTCCTCAACA | 87609627 | 87609649 | 0.25 |
| GATCAGCTCAGGCGCTGACCA | 87609642 | 87609664 | 0.09 |
| GACCAAGGAAAAAGTATACA | 87609657 | 87609679 | 0.16 |
| GTATACAAGGTGCAACTGGT | 87609670 | 87609692 | 0.17 |
| GCAAGGTGCAACTGGTTGGAC | 87609675 | 87609697 | 0.93 |
| GTTAAGTTACTGAGGTCTGTG | 87609701 | 87609723 | 0.01 |
| GTCTTAGGGATTAAGTTACTG | 87609710 | 87609732 | 0.01 |
| GTTTTAAAATTCAGTCTCTTA | 87609725 | 87609747 | 0.01 |
| GAATTTTAAAAGTAATTGATT | 87609738 | 87609760 | 0.03 |
| GTAAAAGTAATTGATTCGGTG | 87609743 | 87609765 | 0.01 |
| GACGCGCGTACGCGAGCGAGC | 87609784 | 87609806 | 0.02 |
| GCGAGCAGGGCTTGTCTTGC | 87609798 | 87609820 | 0.76 |
| GGGCTTGTCTTGCCGGCCTG | 87609805 | 87609827 | 0.02 |
| GTCTTGCCGGCCTGGGGAAGA | 87609812 | 87609834 | 0.01 |
| GCTCATCCGTCTTCCCCAGGC | 87609817 | 87609839 | 0.00 |
| GTCAGCCCTCTTCCCTGCCC | 87609839 | 87609861 | 0.49 |
| GAAACGGTCCCTCCCGGGCA | 87609851 | 87609873 | 0.26 |
| GTCATAGAAACGGTCCCTCCC | 87609856 | 87609878 | 0.01 |
| GCAGGTGAATCTTCATAGAAA | 87609867 | 87609889 | 0.01 |
| GTATGAAGATTCACCTGTGCA | 87609874 | 87609896 | 0.32 |
| GTCACCTGTGCAAGGTGTAAT | 87609883 | 87609905 | 0.01 |
| GCAGCCTGTTACTCACACAG | 87609917 | 87609939 | 1.79 |
| GTGTGAGTAACAGGCTGCGA | 87609922 | 87609944 | 0.07 |
| GAGAATGATTCATTCCATCT | 87609956 | 87609978 | 0.01 |
| GCACGATACTCTCCCCCCAGA | 87609970 | 87609992 | 0.09 |
| GTATCGTGCCACTTCGCTGC | 87609985 | 87610007 | 0.01 |
| GACATCGCCCCCGCAGCGAAG | 87609993 | 87610015 | 0.01 |
| GATGTCTCACTAAGCAATGTC | 87610012 | 87610034 | 16.07 |
| GCAATGTCTGGCCAGAGTCCT | 87610025 | 87610047 | 0.01 |
| GTCTCTCAGTGCCGAGGACTC | 87610035 | 87610057 | 0.04 |
| GATGCTGCTCTCTCAGTGCCG | 87610042 | 87610064 | 0.07 |
| GTCTAGAGAAAAGCACCTACC | 87610074 | 87610096 | 0.01 |
| GGGGAAAACTCGAACTTTCC | 87610092 | 87610114 | 0.03 |
| GATTATCAACGAAACACTCTG | 87610111 | 87610133 | 0.00 |
| GAATGGATTTGTGCTCTCTCC | 87610131 | 87610153 | 0.01 |
| GTTTGTGCTCTCTCCTGGGTG | 87610137 | 87610159 | 0.01 |
| GCTCCTGGGTGAGGACAAAGA | 87610147 | 87610169 | 0.09 |
| GCAAAGAAGGCTCTCCATTCT | 87610161 | 87610183 | 0.02 |
| GTCCATTCTTGGCATGCGCAG | 87610173 | 87610195 | 0.04 |
| GGTCAGAGGCACAGGAGTC | 87610200 | 87610221 | 0.01 |
| GAGTGGATGGTCAGAGGCAC | 87610207 | 87610229 | 0.01 |
| GTCCTGGAGTGGATGGTCAG | 87610213 | 87610235 | 0.40 |

| | | | |
|---|---|---|---|
| GAGTTCTGTCCTGGAGTGGA | 87610220 | 87610242 | 0.63 |
| GCAAAAGTGAGTTCTGTCC | 87610229 | 87610250 | 0.00 |
| GAAATGTATATAGAAGATG | 87610271 | 87610292 | 0.27 |
| GAAGATGTGGACCTGCTAAT | 87610283 | 87610305 | 0.00 |
| GTTTGGGAAGAGCCTATTAGC | 87610294 | 87610316 | 2.71 |
| GTATTTTAAAAGACTGTATTT | 87610311 | 87610333 | 0.04 |
| GCATGCGTGACGTATCCTCAG | 87610398 | 87610420 | 0.79 |
| GTATCCTCAGAGGCCGGAAG | 87610408 | 87610430 | 0.04 |
| GATGCCCTAGAATTAGAATTT | 87610438 | 87610460 | 0.03 |
| GGTAGCTGTTAGCTATAATG | 87610460 | 87610482 | 0.07 |
| GTTAGCTATAATGTGGGTGC | 87610467 | 87610489 | 0.04 |
| GTATAATGTGGGTGCTGGGAA | 87610473 | 87610495 | 0.16 |
| GACTGAGGTAGTTGAGGGGGC | 87610549 | 87610571 | 0.01 |
| GACATCTAACTGAGGTAGTTG | 87610556 | 87610578 | 0.01 |
| GACTACCTCAGTTAGATGTCT | 87610561 | 87610583 | 0.08 |
| GTTAGATGTCTTGGGGGTGA | 87610570 | 87610592 | 0.01 |
| GATGTCTTGGGGGTGAGGGAC | 87610575 | 87610597 | 0.06 |
| GACTCTGAAAGTGATAGACAA | 87610598 | 87610620 | 0.01 |
| GCTGGTTTTGGTGACAGAA | 87610641 | 87610662 | 0.01 |
| GTTGTTGTTTATTGCTGGTTT | 87610652 | 87610674 | 0.01 |
| GTAGTGTTTGTTGTTTATTGC | 87610658 | 87610680 | 0.89 |
| GTAACACCCAGTGACCCCATT | 87610679 | 87610701 | 5.25 |
| GAAGGCCCAAATGGGGTCAC | 87610685 | 87610707 | 0.01 |
| GCCTCAAGGAAGGCCCAAAT | 87610693 | 87610715 | 0.11 |
| GTATGGGCTATGCCTCAAGGA | 87610703 | 87610725 | 0.01 |
| GCATAGCCCATACTCAAGAG | 87610714 | 87610736 | 0.01 |
| GTGTTGCCGCTCTTGAGTAT | 87610720 | 87610742 | 0.01 |
| GAGCGGCAACACCAAGAGA | 87610731 | 87610752 | 0.41 |
| GGACTTACATGGCCTTCTCT | 87610742 | 87610764 | 0.01 |
| GTCTGTTTTCAGGACTTACA | 87610753 | 87610775 | 0.19 |
| GAAAGAGTCAAGTCTGTTTTC | 87610763 | 87610785 | 0.03 |
| GTTGACTCTTTGGTGCTTGTG | 87610776 | 87610798 | 0.12 |
| GAGCTCTTGTGTCCAGAATG | 87610798 | 87610820 | 0.00 |
| GCCTTTCTCTAGGCCGCATTC | 87610810 | 87610832 | 0.01 |
| GCAGTCTGTGCCCTTTCTCT | 87610821 | 87610843 | 0.00 |
| GGCACAGACTGCAGCGTGCA | 87610832 | 87610854 | 1.92 |
| GACTGCAGCGTGCATGGTCC | 87610838 | 87610860 | 0.01 |
| GCTCTCCTGGGAGGATTCGCC | 87610856 | 87610878 | 0.54 |
| GCTATGCTACCTCTCCTGGG | 87610866 | 87610888 | 0.31 |
| GAGGTAGCATAGCCAGTCC | 87610876 | 87610897 | 0.02 |
| GTCGGCTTTGTACCTGGAC | 87610888 | 87610909 | 1.02 |
| GGTACAAAGCCGACTCTCTG | 87610896 | 87610918 | 0.17 |
| GCAAAGCCGACTCTCTGAGGC | 87610900 | 87610922 | 2.14 |
| GGTCCCCAGCCTCAGAGAGT | 87610905 | 87610927 | 0.01 |
| GCTCTGAGGCTGGGGACCCAA | 87610911 | 87610933 | 0.01 |
| GGGGAAGTGGGGTCTCCTTT | 87610926 | 87610948 | 0.01 |
| GTTCTACGACATGGGGAAGTG | 87610937 | 87610959 | 0.89 |
| GTCCCCATGTCGTAGAATTTC | 87610944 | 87610966 | 1.43 |
| GAGCTAAGCTCCACAGAAGC | 87610973 | 87610995 | 0.48 |
| GGGACTGCTGCCAGCTTCTG | 87610983 | 87611005 | 0.01 |
| GTTGAGTGACTGGTGGTAAAC | 87611004 | 87611026 | 0.00 |
| GCTCTACTCTTGAGTGACTGG | 87611012 | 87611034 | 0.01 |

| | | | |
|---|---|---|---|
| GAATTCCCCAGAGCAAGACT | 87611034 | 87611056 | 0.02 |
| GCTGCCTCCAAGTCTTGCTCT | 87611040 | 87611062 | 0.01 |
| GAGGCAGGACTTCTCAATCG | 87611056 | 87611078 | 0.13 |
| GCTTCTCAATCGAGGGACATC | 87611065 | 87611087 | 0.01 |
| GCCTTATGTTTTATTTGTGA | 87611106 | 87611128 | 0.01 |
| GTTACTTCTGAGTCATTCTGC | 87611128 | 87611150 | 0.45 |
| GACTCAGAAGTAAAAGCACT | 87611138 | 87611160 | 1.34 |
| GAAGTAAAAGCACTAGGCACC | 87611145 | 87611167 | 0.29 |
| GTGCAGTGTCATGTCAGCC | 87611163 | 87611184 | 0.01 |
| GTTGACCTCTGTGTCATGCCG | 87611195 | 87611217 | 0.00 |
| GTTTGTTTATGTGTGTATGT | 87611234 | 87611256 | 0.01 |
| GATGTATCTTTAGAGAATGTA | 87611267 | 87611289 | 0.01 |
| GTCTTTAGAGAATGTATGGAG | 87611272 | 87611294 | 0.19 |
| GGAGTGGGTGGGTAGTAGTC | 87611288 | 87611310 | 0.02 |
| GATATTGTAGGATATTTTTCA | 87611313 | 87611335 | 0.01 |
| GTAGGATTGCGTGTATATTGT | 87611326 | 87611348 | 0.01 |
| GCACTGTGGGGACTTCTCTCT | 87611345 | 87611367 | 0.01 |
| GCCAGGAACAAACTCACTGTG | 87611358 | 87611380 | 0.02 |
| GGGACCCGAGCAGACGTAAG | 87611380 | 87611402 | 0.00 |
| GCTCTCCTCTTACGTCTGCTC | 87611384 | 87611406 | 0.02 |
| GCAGACGTAAGAGGAGAGAGA | 87611390 | 87611412 | 18.80 |
| GATGGCTTCTGATGCACTTTT | 87611420 | 87611442 | 0.01 |
| GTCAGAAGCCATAGCAACGCC | 87611432 | 87611454 | 0.07 |
| GCACCTGCCTGGCGTTGCTA | 87611439 | 87611461 | 0.17 |
| GCAACGCCAGGCAGGTGCTGC | 87611445 | 87611467 | 0.33 |
| GCAGGCCGGCAGCACCTGCC | 87611450 | 87611472 | 0.01 |
| GGCCTGCCATCAGCCCATCA | 87611466 | 87611488 | 0.12 |
| GCCATCAGCCCATCAAGGGG | 87611471 | 87611493 | 0.03 |
| GCCCATCAAGGGGAGGGGAG | 87611478 | 87611500 | 0.01 |
| GGGGAGGGGAGGGAGGGGTT | 87611497 | 87611519 | 0.03 |
| GGGGAGGGAGGGGTTGGGGC | 87611502 | 87611524 | 0.03 |
| GGGAGGGGTTGGGGCTGGGG | 87611507 | 87611529 | 0.02 |
| GGCTGGGGAGGAACAGAGGG | 87611519 | 87611541 | 0.06 |
| GGAGGAACAGAGGGAGGGGT | 87611525 | 87611547 | 0.13 |
| GAAGACGTTTAAAGAGTTAA | 87611553 | 87611575 | 0.21 |
| GTTAAAGGTCATCCTAAAC | 87611568 | 87611589 | 0.20 |
| GAACTCAAACTACTCCAGTTT | 87611580 | 87611602 | 0.01 |
| GACTGGAGTAGTTTGAGTTCT | 87611585 | 87611607 | 0.02 |
| GTTTGAGTTCTAGGCTAGTC | 87611594 | 87611616 | 0.01 |
| GAAGGCAATAAAACAATTTAC | 87611661 | 87611683 | 0.02 |
| GAAACAATTTACTGGCGAACT | 87611670 | 87611692 | 0.03 |
| GTCAGACTAAACAAATGATCT | 87611703 | 87611725 | 0.01 |
| GATGATCTTGGGGCCCAGCCA | 87611716 | 87611738 | 0.52 |
| GCTTGGAACAGACCATGGCT | 87611728 | 87611750 | 0.03 |
| GCTGGCTAAGCTTTGGGAGCT | 87611745 | 87611767 | 0.05 |
| GCTCTAGACTGGCTAAGCTTT | 87611752 | 87611774 | 0.43 |
| GGGGATGGAAACTCTAGAC | 87611764 | 87611785 | 0.03 |
| GAGTTGTATAAGAAAGGGGA | 87611778 | 87611800 | 0.01 |
| GTAGAGAATAGCAGGTGTGG | 87611807 | 87611829 | 0.01 |
| GCATGTAGAGAATAGCAGGTG | 87611810 | 87611832 | 0.01 |
| GTAGTCCATGTAGAGAATAGC | 87611815 | 87611837 | 0.03 |
| GCAGTCAGTCGGTCGATCGAT | 87611843 | 87611865 | 0.32 |

| | | | |
|---|---|---|---|
| GCAATCTATAAACCAGTCAGT | 87611855 | 87611877 | 0.25 |
| GTAGATTGACTTTTGCAGTGA | 87611871 | 87611893 | 0.01 |
| GCAGTGATGGAGATGAACCC | 87611884 | 87611906 | 0.02 |
| GACTAGTGTGCAAAAAGTCC | 87611902 | 87611924 | 1.25 |
| GCTCTGGGGATATAGCTTAG | 87611934 | 87611956 | 0.01 |
| GCTAAGCTATATCCCCAGAGC | 87611937 | 87611959 | 0.02 |
| GAAAAACATTTCCCGCTCTG | 87611948 | 87611970 | 0.29 |
| GTTTTTCAATGAAGCAGAGT | 87611964 | 87611986 | 0.01 |
| GCAATGAAGCAGAGTTGGAAG | 87611970 | 87611992 | 0.01 |
| GAAGAGGTTATTAACACAGA | 87611986 | 87612008 | 0.06 |
| GTATTAACACAGATGGAAGCA | 87611994 | 87612016 | 0.01 |
| GATGGAAGCACGGGTGTTGTA | 87612005 | 87612027 | 0.01 |
| GATTCTTGTCCCGAGTTGGTT | 87612030 | 87612052 | 0.01 |
| GAAATCTAACCAAACCAACTC | 87612038 | 87612060 | 0.01 |
| GTTTTTTTTAGTCTTTGAGGT | 87612067 | 87612089 | 0.00 |
| GTCTTTGAGGTAGGGTGTC | 87612076 | 87612097 | 0.01 |
| GTTGTCACCACAGCACTGGAG | 87612123 | 87612145 | 0.01 |
| GCATGCTTGTCACCACAGCAC | 87612128 | 87612150 | 0.01 |
| GCAGTGTTAGGAGTAGGACGT | 87612156 | 87612178 | 1.09 |
| GCTTACTGCAGTGTTAGGAGT | 87612163 | 87612185 | 0.06 |
| GCTTTCTTACTGCAGTGTT | 87612169 | 87612190 | 0.01 |
| GTTTTTTGTTTTGTGTTTGTT | 87612201 | 87612223 | 0.01 |
| GGTTTTGTTTTCTCGAGGCA | 87612222 | 87612244 | 0.83 |
| GAGCTTTCAGCCGAGCGTGG | 87612356 | 87612378 | 0.00 |
| GCTCACTTTTCTAGTTCTCTT | 87612376 | 87612398 | 0.01 |
| GTAAGTTTTAAAAAGTAGA | 87612413 | 87612434 | 0.01 |
| GAAAACTTACATTATGCATAT | 87612426 | 87612448 | 0.02 |
| GTAAATTATCAGCGCACTGCT | 87612462 | 87612484 | 0.02 |
| GTATCAGCGCACTGCTTGGCA | 87612467 | 87612489 | 0.01 |
| GGCATGGTAGGTACCTGCAG | 87612483 | 87612505 | 0.02 |
| GTACCTGCAGGGGAGAGAAG | 87612493 | 87612515 | 0.03 |
| GAAGAGGTCAACAGATGTCC | 87612509 | 87612531 | 0.56 |
| GTCAACAGATGTCCTGGAAC | 87612515 | 87612537 | 2.42 |
| GTTGTGAGCTGCTGTGTAGG | 87612549 | 87612571 | 0.01 |
| GCTGTGTAGGTGGTGGAGAC | 87612559 | 87612581 | 1.37 |
| GGTGGTGGAGACTGGACCC | 87612567 | 87612588 | 0.03 |
| GAGACTGGACCCAGGTGCTC | 87612574 | 87612596 | 0.01 |
| GGTGCTCTGGAAGAACAGC | 87612587 | 87612608 | 0.01 |
| GGCTGGGCAGTTGAGAACAC | 87612605 | 87612627 | 0.06 |
| GCTGGGAAGGTAGTTTAGGGC | 87612634 | 87612656 | 0.02 |
| GTAACATAGGACAACCTGGGA | 87612648 | 87612670 | 0.14 |
| GGTTGTCCTATGTTAGACAG | 87612656 | 87612678 | 0.24 |
| GAATTCTCCCCTGTCTAACAT | 87612662 | 87612684 | 0.01 |
| GACAGGGGAGAATTTAGACTT | 87612672 | 87612694 | 0.02 |
| GACCTTGGATATGACAGGTC | 87612698 | 87612720 | 0.01 |
| GAAAATGTACATCCCAGACCT | 87612713 | 87612735 | 0.01 |
| GCAAGTGCTGTGACCTCCCTC | 87612747 | 87612769 | 0.01 |
| GTGACCTCCCTCTGGCCTA | 87612755 | 87612776 | 0.22 |
| GATCTGCGGCCATAGGCCAGA | 87612762 | 87612784 | 0.02 |
| GAACACCCATCTGCGGCCAT | 87612770 | 87612792 | 0.01 |
| GATGTTTAGAACACCCATCTG | 87612777 | 87612799 | 0.01 |
| GTTCTAAACATGAGCCCTCC | 87612789 | 87612811 | 1.14 |

| | | | |
|---|---|---|---|
| GCGCCTGGGGATTGGCCGGG | 87612804 | 87612826 | 0.01 |
| GAATGTTAGGCGCCTGGGGAT | 87612812 | 87612834 | 0.01 |
| GTTTCAAATGTTAGGCGCCT | 87612818 | 87612840 | 0.27 |
| GATGCTCCGTTTCAAATGTT | 87612826 | 87612848 | 0.01 |
| GTTACAGACGCTTAATTCGTC | 87612849 | 87612871 | 0.01 |
| GACTGGCTATTTTTTAATGCC | 87612868 | 87612890 | 0.02 |
| GTCAGAGCGGGTCGCAGCTAC | 87612886 | 87612908 | 0.00 |
| GATATGGCACAGGCTCAGAG | 87612900 | 87612922 | 0.48 |
| GCCACACTAGTGATATGGCAC | 87612910 | 87612932 | 0.01 |
| GATCACTAGTGTGGAGATCAG | 87612920 | 87612942 | 0.30 |
| GAGATCAGAGGACGATGTAC | 87612932 | 87612954 | 0.12 |
| GTACAGGAGCCAGTTCAATT | 87612948 | 87612970 | 0.50 |
| GCCAGTTCAATTGGGCTTCC | 87612956 | 87612978 | 0.01 |
| GGGCTTCCGGGATTCTAAAA | 87612968 | 87612990 | 0.02 |
| GCTGATCCCGTTTTAGAATCC | 87612974 | 87612996 | 0.14 |
| GGGATCAGGCTTGTCAGATT | 87612989 | 87613011 | 0.18 |
| GTCAGATTTGGTGGCACCTGC | 87613002 | 87613024 | 0.00 |
| GATGGCTCAGGGCATTCCGGC | 87613017 | 87613039 | 0.01 |
| GATGGTGGTGACATGGCTCA | 87613029 | 87613051 | 0.04 |
| GTAAGGCCGATGGTGGTGACA | 87613036 | 87613058 | 1.08 |
| GCCTATTTCATTAAGGCCGA | 87613047 | 87613069 | 0.03 |
| GGCCTTAATGAAATAGGCCA | 87613052 | 87613074 | 0.01 |
| GAATGAAATAGGCCAGGGCGG | 87613058 | 87613080 | 0.73 |
| GCCTGGCAAGAGCCCCCGCCC | 87613069 | 87613091 | 0.00 |
| GAGTGCTGGGATGGATTCC | 87613087 | 87613108 | 0.37 |
| GCTGTAGCCTGAGTGCTGGGA | 87613095 | 87613117 | 0.01 |
| GTACAGAGTGAATTCCAGCCT | 87613113 | 87613135 | 0.20 |
| GCTTTTCTTTATTCCCGAGGC | 87613126 | 87613148 | 0.01 |
| GAAAAAAAAAAGGGGCACGT | 87613148 | 87613170 | 0.01 |
| GCAAGCACGTATTCCTAAACA | 87613178 | 87613200 | 0.54 |
| GTCAAAGAGCGGGCCATGTTT | 87613190 | 87613212 | 0.28 |
| GGCCCGCTCTTTGACCATCT | 87613199 | 87613221 | 0.05 |
| GTTGACCATCTGGGAACTACA | 87613209 | 87613231 | 0.01 |
| GAACTACAAGGCAAGAGTGC | 87613221 | 87613243 | 0.49 |
| GTGCAGGCTTTACAAAGAAC | 87613237 | 87613259 | 0.01 |
| GACTTGCAAAAGACTTTGTCC | 87613256 | 87613278 | 0.54 |
| GTTTCCACTGACCTTAAAAC | 87613313 | 87613335 | 0.01 |
| GCCTTAGATCTCCCTGTTTTA | 87613324 | 87613346 | 0.02 |
| GAACAGGGAGATCTAAGGAAC | 87613330 | 87613352 | 0.26 |
| GATCTAAGGAACTGGCGTT | 87613338 | 87613359 | 0.04 |
| GAACTGGCGTTTGGAGTTAG | 87613346 | 87613368 | 0.01 |
| GTTAGAGGGAAGAAAATCCC | 87613361 | 87613383 | 0.07 |
| GGAAGAAAATCCCAGGGCTG | 87613368 | 87613390 | 0.01 |
| GGCTGTGTGACCGCAGCCCT | 87613378 | 87613400 | 0.05 |
| GCTTTGTCTTGGTTACTGATG | 87613399 | 87613421 | 0.06 |
| GTTGTCATTTACTCTTTGTCT | 87613411 | 87613433 | 0.35 |
| GTAAATGACAAAAAAAAAAA | 87613423 | 87613445 | 0.05 |
| GCCGCCTGCAAAACTCACCC | 87613447 | 87613469 | 0.58 |
| GACTCACCCTGGTTCCCAGCT | 87613459 | 87613481 | 0.00 |
| GGGCTCCGAGCTGGGAACCA | 87613464 | 87613486 | 0.01 |
| GCGGTTCCTGGGCTCCGAGC | 87613473 | 87613495 | 0.00 |
| GCGGTGCTCCTGGCGGTTCC | 87613485 | 87613507 | 0.06 |

| | | | |
|---|---|---|---|
| GGCAGGAGGGGCGGTGCTCC | 87613495 | 87613517 | 0.02 |
| GGATGATCACGTGGCAGGAG | 87613507 | 87613529 | 0.02 |
| GCTGGAGGATGATCACGTGGC | 87613512 | 87613534 | 0.02 |
| GTTGTCTGGGAGGAAGGCTGG | 87613528 | 87613550 | 0.28 |
| GCTCTTCTTTGTCTGGGAGGA | 87613535 | 87613557 | 1.57 |
| GTCCCAGACAAAGAAGAGGGG | 87613541 | 87613563 | 0.02 |
| GAAAGAAGAGGGGAGGAGACT | 87613549 | 87613571 | 0.00 |
| GAAGAGCGAGAGCTGTAGAAG | 87613577 | 87613599 | 0.82 |
| GAGCTGTAGAAGCGGGTCGT | 87613585 | 87613607 | 0.01 |
| GTAGAAGCGGGTCGTGGGCTC | 87613591 | 87613613 | 0.02 |
| GGTAAGGTAAAAGGGAAGAC | 87613617 | 87613639 | 0.04 |
| GATTCTTTGGGTAAGGTAAAA | 87613625 | 87613647 | 0.05 |
| GTTTTTAATATTCTTTGGGTA | 87613633 | 87613655 | 0.01 |
| GTTAATTTTTAATATTCTTT | 87613638 | 87613660 | 0.04 |
| GAAATTAACCCGAGCTGCAAA | 87613653 | 87613675 | 0.01 |
| GCTCGGCTCCTTTTGCAGCTC | 87613660 | 87613682 | 0.01 |
| GCCGAGGCACCTGATCATCT | 87613677 | 87613699 | 0.01 |
| GATCATCTCGGCCTAACCCAG | 87613690 | 87613712 | 0.01 |
| GCCTAACCCAGAGGAGAGGG | 87613699 | 87613721 | 0.72 |
| GGAGAGCCTCCCTCTCCTCT | 87613705 | 87613727 | 0.02 |
| GTATTTGGGTTTGAGGGGGCT | 87613726 | 87613748 | 1.57 |
| GAACATATTTGGGTTTGAGG | 87613731 | 87613753 | 0.01 |
| GTCAAAAGGAGGAACATATTT | 87613741 | 87613763 | 0.02 |
| GAAGGCAACACTCAAAAGG | 87613753 | 87613774 | 0.18 |
| GAGTGTTGCCTTCTACTTC | 87613762 | 87613783 | 0.01 |
| GCAGATCTGTCCAGAAGTAGA | 87613770 | 87613792 | 1.84 |
| GGGGTGGGGAGGAGGGAGGA | 87613806 | 87613828 | 0.05 |
| GAGGATGGGGGTGGGGAGGA | 87613813 | 87613835 | 0.04 |
| GCTGAACGAGGATGGGGGTG | 87613820 | 87613842 | 0.00 |
| GTAAACGCTGAACGAGGATGG | 87613825 | 87613847 | 0.01 |
| GAAATACATAAACGCTGAACG | 87613832 | 87613854 | 0.00 |
| GCGACGTTCATTTGTCAGAG | 87613865 | 87613887 | 0.01 |
| GACAAATGAACGTCGCCAA | 87613872 | 87613893 | 0.17 |
| GACGTCGCCAACGGAAAAACT | 87613881 | 87613903 | 0.01 |
| GCCTTTCCCAAGTTTTTCCGT | 87613887 | 87613909 | 0.49 |
| GACTTCTATTTAGTGTTGTTT | 87613930 | 87613952 | 0.14 |
| GACAATTATAGCCACTCTAGA | 87613962 | 87613984 | 0.00 |
| GTTTTCAACTACCTTCTAGAG | 87613972 | 87613994 | 0.04 |
| GAAGGTAGTTGAAAACGGGAC | 87613981 | 87614003 | 0.03 |
| GAAAACGGGACAGGGATTC | 87613990 | 87614011 | 0.01 |
| GCAGGACCCCCGCCCCAACAG | 87614008 | 87614030 | 0.01 |
| GTCTGCCGCTGTTGGGGCGG | 87614013 | 87614035 | 1.85 |
| GTCACTGTCTGCCGCTGTTG | 87614019 | 87614041 | 0.01 |
| GACAGAAGCAGATAGAGTGAA | 87614054 | 87614076 | 0.02 |
| GAATGGATATTAACAAGTAA | 87614071 | 87614093 | 0.02 |
| GTAAGGGCCTGCATTCACA | 87614087 | 87614108 | 7.32 |
| GCATGGCGTCCATGTGAATGC | 87614094 | 87614116 | 0.00 |
| GAGGCAGTAGTTAGAGGGCA | 87614112 | 87614134 | 1.60 |
| GAAGTCAGAGGCAGTAGTTAG | 87614118 | 87614140 | 0.03 |
| GAATCGTATTTGTGAAGTCAG | 87614131 | 87614153 | 0.30 |
| GCCACATATACTGTACATGA | 87614155 | 87614177 | 0.22 |
| GTTTCTTTTCAGATTTGCTTA | 87614235 | 87614257 | 0.01 |

| | | | |
|---|---|---|---|
| GCTTATGGAGATGAGTGTT | 87614250 | 87614271 | 0.04 |
| GTGTGTACTACTTGCATGCT | 87614319 | 87614341 | 0.02 |
| GACTACTTGCATGCTTGGTGC | 87614325 | 87614347 | 0.01 |
| GGTGCTGGTGGGAGTCAGAG | 87614340 | 87614362 | 0.01 |
| GGTGGGAGTCAGAGAGGGCC | 87614346 | 87614368 | 0.02 |
| GAGAGGGCCTGGGATCCCC | 87614357 | 87614378 | 0.04 |
| GTTAAGTCCAGGGGATCCC | 87614364 | 87614385 | 3.10 |
| GCTGAACTCTAGTTAAGTCCA | 87614373 | 87614395 | 0.03 |
| GTGAGCCACCACGGAGTGCT | 87614403 | 87614425 | 0.01 |
| GTTCAGATCCCAGCACTCCG | 87614411 | 87614433 | 0.39 |
| GGAGTGCTGGGATCTGAACT | 87614415 | 87614437 | 0.06 |
| GAACTTGGGTCCTCTACAAA | 87614430 | 87614452 | 0.04 |
| GACATTTTGTTCCTTTTGTAG | 87614440 | 87614462 | 0.02 |
| GAACAAAATGTTCTTACCAGC | 87614453 | 87614475 | 0.01 |
| GGCTGGAGAGATAGCCCAGC | 87614468 | 87614490 | 0.02 |
| GAACAAGAACACAAGGCCAG | 87614489 | 87614511 | 0.01 |
| GGCCTTGTGTTCTTGTTCTC | 87614494 | 87614516 | 0.01 |
| GCTGAAGCAGAGACAAGCTGA | 87614521 | 87614543 | 0.02 |
| GCGGCCCAGTGTTCTCCTC | 87614543 | 87614564 | 0.01 |
| GCAGCAGCCAGAGGAGAACAC | 87614548 | 87614570 | 0.04 |
| GCATGGGTCTCCAGCAGCCAG | 87614558 | 87614580 | 0.09 |
| GCTGGAGACCCATGTTGAAAG | 87614568 | 87614590 | 2.91 |
| GTGCTTTACCACTTTCAACA | 87614576 | 87614598 | 0.01 |
| GTATTAGATGTGATAGGAGTC | 87614602 | 87614624 | 0.02 |
| GAAGGGTGTATTAGATGTGAT | 87614609 | 87614631 | 0.01 |
| GACTGTGTGTGTGATGATGA | 87614628 | 87614650 | 0.05 |
| GTCTCTTCAAAATGACCAGCT | 87614649 | 87614671 | 0.17 |
| GTCTTTGCAGTCCTCCTAGC | 87614663 | 87614685 | 0.02 |
| GGACTGCAAAGACAGCTCAG | 87614673 | 87614695 | 0.03 |
| GACAGCTCAGAGGTGGAGTGC | 87614684 | 87614706 | 0.01 |
| GTGTGCAGGGAATTAAATTC | 87614723 | 87614745 | 0.02 |
| GTGGTTGTAAGCTGTGTGCA | 87614736 | 87614758 | 0.01 |
| GCTGGGTATTGGAGCTATGGG | 87614755 | 87614777 | 0.00 |
| GTTTCAGATCCCTGGGTAT | 87614767 | 87614788 | 0.00 |
| GACTGAAGTTTCAGATCCCT | 87614773 | 87614795 | 0.22 |
| GTCCTCTGTAGACACCATGTG | 87614794 | 87614816 | 0.00 |
| GTATGGCTGCACCACCTCACA | 87614807 | 87614829 | 0.51 |
| GTTACATTTACTTTGTGTGTA | 87614825 | 87614847 | 0.01 |
| GACTCACCTGAGCCAGACG | 87614859 | 87614880 | 0.14 |
| GAGCAACCACGTCTGGCTC | 87614865 | 87614886 | 0.03 |
| GAAGGCACGAGCAACCACGTC | 87614871 | 87614893 | 2.67 |
| GTATTATTATTATGTTAGACA | 87614949 | 87614971 | 0.01 |
| GATGATAATTCCAGCCCAGTT | 87615011 | 87615033 | 0.92 |
| GCTGTGTGGCCCTAACTGGGC | 87615020 | 87615042 | 0.01 |
| GTAAGATGAGGTCTCACTGTG | 87615035 | 87615057 | 0.01 |
| GACCTCATCTTAAAACAACAG | 87615047 | 87615069 | 0.05 |
| GAACAACAGTGGAGCTGAGTG | 87615059 | 87615081 | 0.11 |
| GTCTACAACCCCAGTACTTG | 87615090 | 87615112 | 0.02 |
| GATCTTTCTGCCTCAAGTAC | 87615100 | 87615122 | 0.01 |
| GGGCATCACATGACACAAGA | 87615138 | 87615160 | 0.01 |
| GTGTTCTTGTTTTGGGGACA | 87615158 | 87615180 | 0.00 |
| GTAGGGAAGTGTTCTTGTTTT | 87615165 | 87615187 | 0.00 |

| | | | |
|---|---|---|---|
| GAACACTTCCCTAATATATGC | 87615176 | 87615198 | 0.01 |
| GCTAGGGCCCTGCATATATTA | 87615183 | 87615205 | 1.21 |
| GATGCAGGGCCCTAGGTTCTA | 87615192 | 87615214 | 0.16 |
| GGGCCCTAGGTTCTATGGCT | 87615197 | 87615219 | 0.09 |
| GGTTGGGTTGGGTATCAGAA | 87615232 | 87615254 | 0.02 |
| GTACCCAACCCAACCCCCAGA | 87615241 | 87615263 | 0.01 |
| GTCAGCTTCCTTCTGGGGGTT | 87615248 | 87615270 | 0.02 |
| GTTTTTGTTCAGCTTCCTTC | 87615256 | 87615278 | 0.02 |
| GCTCCGCAGTTCTTGCTCGCC | 87615289 | 87615311 | 0.04 |
| GACAGGGAACTAAGTAACC | 87615307 | 87615328 | 0.02 |
| GACAAATGAACAAAAGTGACA | 87615322 | 87615344 | 0.06 |
| GTTATTGTTACTGTATTCTT | 87615343 | 87615365 | 0.67 |
| GTTACTGTATTCTTTGGAGAC | 87615350 | 87615372 | 0.02 |
| GAGACAGGATCTTACTGTGT | 87615365 | 87615387 | 0.01 |
| GTCTTACTGTGTAGGTAGCCC | 87615374 | 87615396 | 0.14 |
| GCTGGTTCTGTAGTGAGTTCT | 87615401 | 87615423 | 0.01 |
| GAACTCACTACAGAACCAGGT | 87615406 | 87615428 | 0.39 |
| GCTACAGAACCAGGTTGGCTT | 87615412 | 87615434 | 0.01 |
| GCTATGAGTCCAAAGCCAACC | 87615420 | 87615442 | 6.62 |
| GATTCAGCCCTGGGAAGTCAG | 87615454 | 87615476 | 0.01 |
| GCATGCTTTAATTCAGCCCT | 87615464 | 87615486 | 0.66 |
| GAAAAAAAATCAAGACAGTGT | 87615488 | 87615510 | 0.03 |
| GCAACAAAACAAATAAACAAA | 87615516 | 87615538 | 0.03 |
| GTTTTGTTGAGATAGAGTC | 87615530 | 87615551 | 0.00 |
| GAGATAGAGTCTGGTTGTG | 87615538 | 87615559 | 0.01 |
| GAGTCTGGTTGTGTGGCTC | 87615544 | 87615565 | 0.99 |
| GATGTGCCCAACCCAACTTGA | 87615591 | 87615613 | 0.01 |
| GAAGTACCTTCAAGTTGGGTT | 87615596 | 87615618 | 0.03 |
| GATTTAAAGTACCTTCAAGTT | 87615601 | 87615623 | 0.02 |
| GATATATGTGTAATTTAGAGC | 87615646 | 87615668 | 0.01 |
| GTAATTTAGAGCTGGAGAGA | 87615654 | 87615676 | 0.01 |
| GGCTCACACCCCTCTGTAAT | 87615744 | 87615766 | 0.68 |
| GACACTGGATCCCATTACAG | 87615754 | 87615776 | 0.01 |
| GACATACCAGAAGAGGACAC | 87615769 | 87615791 | 0.03 |
| GAAAAATATTTAATTTTTAAT | 87615840 | 87615862 | 0.05 |
| GCTACTAGACTACGAGAGCAA | 87615886 | 87615908 | 0.01 |
| GCTCTCGTAGTCTAGTAGAA | 87615891 | 87615913 | 0.08 |
| GAACCACCCATCATGTATGCT | 87615918 | 87615940 | 2.42 |
| GTTGGTTCCTAGCATACATGA | 87615924 | 87615946 | 0.01 |
| GTATGCTAGGAACCAAACTG | 87615931 | 87615953 | 0.10 |
| GTCTTATAGAAGACCCCAGTT | 87615943 | 87615965 | 0.57 |
| GTTAAGAGCAAGTACTGGCC | 87616148 | 87616170 | 0.01 |
| GCAGTAGTTAAGAGCAAGTAC | 87616153 | 87616175 | 3.31 |
| GCAATCTCTCCAGTCCATTGC | 87616178 | 87616200 | 0.01 |
| GCAAAAGTACCTGCAATGGAC | 87616186 | 87616208 | 0.01 |
| GTCTATCAAAAGTACCTGCAA | 87616191 | 87616213 | 0.00 |
| GCACAGGACTTAAAAACTTGT | 87616218 | 87616240 | 0.02 |
| GTAGAAACAGACGCTGCACAC | 87616235 | 87616257 | 0.05 |
| GCAGCGTCTGTTTCTAAGTAC | 87616243 | 87616265 | 2.91 |
| GTTTCTAAGTACCGGTTAGTG | 87616252 | 87616274 | 0.01 |
| GTCTATCCCCTCCACACTAAC | 87616262 | 87616284 | 0.01 |
| GGGATAGAGTAACTCTCTGC | 87616277 | 87616299 | 0.01 |

| | | | |
|---|---|---|---|
| GTATACAGGGATGGGGATTCT | 87616329 | 87616351 | 0.49 |
| GACTTAGGATATACAGGGATG | 87616337 | 87616359 | 0.54 |
| GTAACTAACTTAGGATATACA | 87616343 | 87616365 | 0.02 |
| GGATGGCTTTAACTAACTT | 87616353 | 87616374 | 0.01 |
| GTTAGTTAAAGCCATCCTG | 87616358 | 87616379 | 0.00 |
| GTAAAGCCATCCTGTGGTGTT | 87616364 | 87616386 | 0.01 |
| GTCTTCCAAACACCACAGGA | 87616369 | 87616391 | 0.02 |
| GCTTCAGAGCTTTCAAAAGTC | 87616408 | 87616430 | 0.01 |
| GAAGCTCTGAAGGTAAGCTAT | 87616420 | 87616442 | 0.05 |
| GAAGGTAAGCTATAGGCAGT | 87616427 | 87616449 | 0.01 |
| GCTATAGGCAGTAGGCCCCA | 87616435 | 87616457 | 0.01 |
| GCAAGGCTCATGTGTAGCCC | 87616452 | 87616474 | 0.01 |
| GCTACACATGAGCCTTGCTC | 87616457 | 87616479 | 0.01 |
| GGGCTTGCATCCCCGGAGCA | 87616469 | 87616491 | 1.86 |
| GCTTATGGGCTTGCATCCC | 87616476 | 87616497 | 0.01 |
| GATGCAAGCCCATAAGCATT | 87616481 | 87616503 | 0.03 |
| GCTGGTGTTGCCGAATGCTTA | 87616490 | 87616512 | 0.27 |
| GGTGGAGGAATTGTGGGCC | 87616509 | 87616530 | 0.01 |
| GTTGGAAAGGTGGAGGAATTG | 87616515 | 87616537 | 0.15 |
| GTCCTCCACCTTTCCAATGAC | 87616522 | 87616544 | 0.01 |
| GCAAGGCCCTGTCATTGGAA | 87616529 | 87616551 | 0.00 |
| GTTCTTTGGCAAGTGTGTGCA | 87616546 | 87616568 | 0.01 |
| GCAGCAGATGCGGACCTTCTT | 87616561 | 87616583 | 0.02 |
| GAAGGTGTCTTGCAGCAGATG | 87616572 | 87616594 | 0.45 |
| GCAAGACACCTTCAGCATC | 87616583 | 87616604 | 0.01 |
| GACCTTCAGCATCAGGTTCCG | 87616590 | 87616612 | 0.96 |
| GCATCAGGTTCCGAGGGAAC | 87616597 | 87616619 | 0.04 |
| GAGGGAACCGGAGTCAGACC | 87616609 | 87616631 | 3.67 |
| GTTCAAGGCCAGGTCTGACTC | 87616616 | 87616638 | 0.01 |
| GCCTTGAATCAATCCTCGTC | 87616631 | 87616653 | 0.00 |
| GTTCACAGGGACAACCAGACG | 87616644 | 87616666 | 0.06 |
| GAAGGCCATATGGGAATTCAC | 87616659 | 87616681 | 0.00 |
| GGATGGGAGCAAGGCCATAT | 87616669 | 87616691 | 0.05 |
| GCCTTGCTCCCATCCACGTC | 87616677 | 87616699 | 0.58 |
| GAAAGACAGCCAGACGTGGA | 87616686 | 87616708 | 0.00 |
| GGCTGTCTTTCTTGGTTGC | 87616698 | 87616719 | 0.01 |
| GCTTTCTTGGTTGCCGGTGTG | 87616704 | 87616726 | 1.03 |
| GTCGCTGTTGGTCCTCACAC | 87616716 | 87616738 | 0.02 |
| GAGGACCAACAGCGACCTC | 87616723 | 87616744 | 0.01 |
| GTTTGGCCTGAGGTCGCTGT | 87616728 | 87616750 | 5.76 |
| GTTGCTTCTGTTTGGCCTG | 87616738 | 87616759 | 0.01 |
| GAACAAGGTGTTGCTTCTGTT | 87616745 | 87616767 | 2.89 |
| GGAACTGGTGGAAAGCAACA | 87616761 | 87616783 | 0.08 |
| GCACAACACTGAGGGAACTGG | 87616773 | 87616795 | 0.01 |
| GCTGTGGAATCACAACACTGA | 87616782 | 87616804 | 3.79 |
| GAGCACTTGTCAGCGCTCTG | 87616799 | 87616821 | 0.50 |
| GCTCCAGTCTCTGCTCACCA | 87616818 | 87616840 | 0.21 |
| GGATCGGAGCGGCAGAGCCA | 87616835 | 87616857 | 0.26 |
| GCATCTGCAATAGGATCGGAG | 87616846 | 87616868 | 0.36 |
| GCCTCTTGAGTCATCTGCAAT | 87616856 | 87616878 | 1.03 |
| GCAGAAGCCAGCAGCCATGTG | 87616879 | 87616901 | 0.03 |
| GCAGTGTCCACACATGGCTGC | 87616885 | 87616907 | 0.01 |

| | | | |
|---|---|---|---|
| GCTATGCAGTGTCCACACA | 87616892 | 87616913 | 0.01 |
| GACACTGCATAGCTCCTTAGG | 87616902 | 87616924 | 0.01 |
| GCTGCTGTGGTTTTCCACCTA | 87616915 | 87616937 | 0.01 |
| GGAAAACCACAGCAGTCCC | 87616923 | 87616944 | 0.00 |
| GGTTCTGCCGGGGACTGCTG | 87616929 | 87616951 | 0.10 |
| GCTTTGGGTGTGGTTCTGCC | 87616940 | 87616962 | 0.07 |
| GCAGAACCACACCCAAAGCGC | 87616945 | 87616967 | 0.14 |
| GCTGTGAGTTCCCAGCGCTTT | 87616955 | 87616977 | 0.01 |
| GACTCTGTGTGGGAGAAGCTG | 87616988 | 87617010 | 0.01 |
| GGAGGAACAAGACTCTGTGT | 87616999 | 87617021 | 0.37 |
| GGTCCAGGGAGGAAGAAAAC | 87617020 | 87617042 | 0.01 |
| GCTTCCTCCCTGGACCACGTC | 87617028 | 87617050 | 0.12 |
| GCGGAGGCCAGACGTGGTCC | 87617035 | 87617057 | 0.01 |
| GGAGAAGCGGAGGCCAGACG | 87617041 | 87617063 | 39.00 |
| GGGGTAAAAAGGAGAAGCGG | 87617051 | 87617073 | 0.04 |
| GATTCAAGAAATGGGGTAAAA | 87617062 | 87617084 | 0.01 |
| GCAGACGAGATTCAAGAAATG | 87617070 | 87617092 | 0.10 |
| GCGTACACTGGAGAGGAGAG | 87617097 | 87617119 | 0.01 |
| GTCTCTGGGCGTACACTGGAG | 87617104 | 87617126 | 0.15 |
| GATGTTCTCTGGGCGTACAC | 87617109 | 87617131 | 6.78 |
| GGAAACAGGTGATGTTCTCT | 87617119 | 87617141 | 0.01 |
| GCACTGAGACTGTGGGGAAAC | 87617133 | 87617155 | 0.01 |
| GTTTACAGCACTGAGACTGTG | 87617140 | 87617162 | 0.64 |
| GGCAGGTAGTCATTTTGTTT | 87617172 | 87617194 | 0.00 |
| GACTACCTGCCTTCTCAATGT | 87617185 | 87617207 | 0.01 |
| GCAGGCTAACCTACATTGAGA | 87617193 | 87617215 | 0.01 |
| GTAGGTTAGCCTGGTTGAGC | 87617203 | 87617225 | 0.04 |
| GCCTGGGAAGCCTGCTCAACC | 87617212 | 87617234 | 0.02 |
| GTTGTAGACGTGCAATAGACC | 87617230 | 87617252 | 0.01 |
| GCAACAAAGAATCTACTGTCT | 87617250 | 87617272 | 0.00 |
| GCAGATTCAAGAATCACAGGA | 87617275 | 87617297 | 0.01 |
| GACATGTAACATATTGTATCT | 87617315 | 87617337 | 0.06 |
| GTATGTTACATGTTAATTTTC | 87617326 | 87617348 | 0.01 |
| GTTTTCAGGTTGTTGTCCAAT | 87617341 | 87617363 | 0.45 |
| GAAAAAAAAAAGTTTTCCTAT | 87617356 | 87617378 | 0.00 |
| GCAACTCAACTACAGAGTA | 87617388 | 87617409 | 0.23 |
| GAAATTATTTGTATCGTGCT | 87617423 | 87617445 | 0.64 |
| GTACAAATAATTTCTATCTGG | 87617433 | 87617455 | 0.02 |
| GTTTCTATCTGGTGGAAATAT | 87617442 | 87617464 | 0.01 |
| GTAGAAACAGAGCCTAGGGC | 87617664 | 87617686 | 0.01 |
| GAGCAGTAGAAACAGAGCCT | 87617669 | 87617691 | 0.03 |
| GCTAGGTTAGATACAAAAT | 87617691 | 87617712 | 0.01 |
| GAACGGTATAGCGCATTTGCT | 87617707 | 87617729 | 0.01 |
| GAAGAAACTGGGTATTTAGAA | 87617725 | 87617747 | 0.01 |
| GTTCCGAGACACTAAGAAAC | 87617738 | 87617760 | 0.02 |
| GAACTAGCTTTACAGTCTTT | 87617757 | 87617779 | 0.02 |
| GTTTCGGGTTCTCTCACTAAT | 87617774 | 87617796 | 0.88 |
| GTAGGAAAACTGAATAAAAAT | 87617793 | 87617815 | 0.08 |
| GAAAATAGGTTCTCAGCCTTC | 87617808 | 87617830 | 0.02 |
| GGTTCTCAGCCTTCTGGCTA | 87617814 | 87617836 | 0.01 |
| GCCTTCTGGCTAAGGTCATG | 87617822 | 87617844 | 0.01 |
| GCTAAGGTCATGTGGAAAGTT | 87617831 | 87617853 | 0.01 |

| | | | |
|---|---|---|---|
| GACATGTGTAACCATACTTTT | 87617854 | 87617876 | 0.01 |
| GAATAAAGTAACCGAAAAGTA | 87617864 | 87617886 | 0.02 |
| GAAAGGGATAAGTGTGGAGC | 87617907 | 87617929 | 0.05 |
| GAGATTGAAAGGGATAAGTG | 87617913 | 87617935 | 0.98 |
| GCCTAATGTCAGAGATTGAAA | 87617923 | 87617945 | 0.02 |
| GTAGGTAGGAGAGAAGAATAG | 87617942 | 87617964 | 0.01 |
| GAGAAGAATAGAGGGAAAGA | 87617951 | 87617973 | 0.63 |
| GACCTCTTCCTGCTGATTAG | 87617988 | 87618010 | 0.05 |
| GCTCATCACCCCTAATCAGC | 87617996 | 87618018 | 0.03 |
| GCAAGTCTGACCTTCATCTTC | 87618023 | 87618045 | 0.18 |
| GATTGGATATCCTGAAGATGA | 87618032 | 87618054 | 0.21 |
| GAAGAGACTAGAAGAAGACAT | 87618050 | 87618072 | 2.06 |
| GCTTAGGTTTGGTTGTCAAG | 87618082 | 87618104 | 0.00 |
| GACAACCAAACCTAAGCAGC | 87618088 | 87618110 | 0.25 |
| GCTGCTGGTTCCTGCTGCTT | 87618098 | 87618120 | 0.44 |
| GCAGCAGGAACCAGCAGCTGC | 87618104 | 87618126 | 0.01 |
| GCTGCTCTCCCTGCAGCTGC | 87618113 | 87618135 | 0.02 |
| GCCAGTGCAGGGAGTGGCTG | 87618138 | 87618160 | 0.28 |
| GAAAAACGCCAGTGCAGGGAG | 87618144 | 87618166 | 0.01 |
| GATATAAAAACGCCAGTGCA | 87618149 | 87618171 | 0.01 |
| GCCAGCTGAAGAGTTTATGTT | 87618193 | 87618215 | 0.01 |
| GCTGGCAAAATCACACCCCT | 87618211 | 87618233 | 0.01 |
| GAAAATCACACCCCTGGGGGC | 87618217 | 87618239 | 0.01 |
| GTGGGCAAGAGACTGTCAG | 87618252 | 87618273 | 0.01 |
| GTTACATGTTCATTTGCACAC | 87618343 | 87618365 | 0.02 |
| GCATATTTTTGTTTTAATCCC | 87618478 | 87618500 | 0.02 |
| GATATGTTCATATAATACAAC | 87618496 | 87618518 | 4.01 |
| GTTTGTCTATAGTGAGATTTT | 87618531 | 87618553 | 0.03 |
| GCAGCAGGTAAGAGACACAGA | 87618622 | 87618644 | 0.94 |
| GGAAGAGGGGCTGACGAGA | 87618647 | 87618668 | 0.17 |
| GGGGAAGAGCTTAGGAAGAG | 87618659 | 87618681 | 0.01 |
| GTAGACTGAGGGGAAGAGCTT | 87618667 | 87618689 | 0.01 |
| GTAGCAGAGTTGTAGACTGAG | 87618678 | 87618700 | 0.00 |
| GCCTGTGATTTTTCTTCAAGG | 87618710 | 87618732 | 0.01 |
| GACTTGGACATGGATGTTCTC | 87618738 | 87618760 | 0.59 |
| GGATGACAGCACTTGGACA | 87618749 | 87618770 | 1.32 |
| GTCCAAGTGCTGTCATCCTGC | 87618754 | 87618776 | 0.13 |
| GATCCTGCTGGAGTTATCCTG | 87618767 | 87618789 | 0.59 |
| GTTATCCTGTGGTAACTTGA | 87618778 | 87618800 | 0.26 |
| GAAACCCCCTCAAGTTACCAC | 87618783 | 87618805 | 0.00 |
| GACTTATTTATTTTTATTTGG | 87618820 | 87618842 | 0.00 |
| GCCATGCCACAGTGTGTAAG | 87618953 | 87618975 | 2.63 |
| GACTCCTCCACTTACACACTG | 87618959 | 87618981 | 0.50 |
| GTGTGTAAGTGGAGGAGTGG | 87618964 | 87618986 | 0.06 |
| GTGGAGGAGTGGAGGTCAG | 87618972 | 87618993 | 0.01 |
| GAGGAGAACTTCTCTGAGT | 87618990 | 87619011 | 0.07 |
| GGCTCTCTCCTCCTAGTCTA | 87619010 | 87619032 | 0.01 |
| GTCTCCTCCTAGTCTATGGTT | 87619015 | 87619037 | 0.17 |
| GTCTTCTCCAAACCATAGACT | 87619021 | 87619043 | 0.00 |
| GGGGAAAGAATTGTCAAGCT | 87619058 | 87619080 | 0.92 |
| GTCAGTGAGATGGCTGAGCG | 87619078 | 87619100 | 0.12 |
| GTCTGTCTTGTCAGTGAGA | 87619088 | 87619109 | 0.01 |

| | | | |
|---|---|---|---|
| GTTCTGTACCTTTCAGTACAC | 87619112 | 87619134 | 0.81 |
| GTCAGTACACTGGCTAAACAC | 87619123 | 87619145 | 0.11 |
| GGCTAAACACAGGGGAAAAC | 87619133 | 87619155 | 0.18 |
| GACAGGGGAAAACAGGTAGCC | 87619141 | 87619163 | 0.01 |
| GTTGTATCTAATCAAAAGACC | 87619159 | 87619181 | 0.02 |
| GATACAGATTTCAAGATCTCC | 87619190 | 87619212 | 0.02 |
| GTATGCACTATACTATTCTCC | 87619208 | 87619230 | 0.76 |
| GACTTTTTTTTTAATTTTA | 87619236 | 87619257 | 0.01 |
| GTTTTAATTTTAAGGAAAAAA | 87619244 | 87619266 | 0.00 |
| GACATAAAATAAAACAAATAA | 87619272 | 87619294 | 0.01 |
| GCTGGGTTGCTTCTCAGGCTC | 87619344 | 87619366 | 0.00 |
| GCTGACTGGGTTGCTTCTC | 87619350 | 87619371 | 2.72 |
| GACAGAGGATCACTGCTGAC | 87619363 | 87619385 | 0.75 |
| GTGATCCTCTGTCACTGTG | 87619373 | 87619394 | 0.87 |
| GCAGGAGCCTCACAGTGACAG | 87619378 | 87619400 | 0.01 |
| GTCACTGTGAGGCTCCTGCCT | 87619384 | 87619406 | 0.02 |
| GTCACTAAGGGTAGCCAAGGC | 87619397 | 87619419 | 0.02 |
| GCTTACGGACTCTGTCACTAA | 87619410 | 87619432 | 0.01 |
| GCCATGATCGGAAACAACTTA | 87619426 | 87619448 | 0.02 |
| GCTGTGATAAACACCATGAT | 87619439 | 87619461 | 0.02 |
| GTTTAATCAGTCTTGCTGTCT | 87619475 | 87619497 | 0.01 |
| GTAAAGGCTAAGTGTCACAGC | 87619494 | 87619516 | 0.01 |
| GCTAAGTGTCACAGCCGGCG | 87619499 | 87619521 | 0.03 |
| GCACAGCCGGCGCGGCATCCC | 87619508 | 87619530 | 0.01 |
| GAGCTCCGGGGATGCCGCGC | 87619513 | 87619535 | 0.01 |
| GTGAAGTAGTTGTGAGCTCC | 87619526 | 87619548 | 0.57 |
| GTGCAGAGGTCAGAGGGTGA | 87619548 | 87619570 | 0.04 |
| GATTCTGTGCAGAGGTCAGA | 87619554 | 87619576 | 0.01 |
| GACCTCTGCACAGAATCCAT | 87619560 | 87619582 | 0.06 |
| GCACAGAATCCATGGGGGGG | 87619567 | 87619589 | 0.02 |
| GTGCGTACCCACCCCCCCA | 87619576 | 87619597 | 0.03 |
| GTTTGTTAGTGATTACAAACT | 87619664 | 87619686 | 0.02 |
| GAAATTTAAAAGACTATTGAA | 87619699 | 87619721 | 0.43 |
| GTTTTTTGCATTTATTTAGAG | 87619743 | 87619765 | 0.01 |
| GACATACACGCATGCACACAC | 87619767 | 87619789 | 0.02 |
| GAGAGAGAGAGGATACCCTG | 87619831 | 87619853 | 0.79 |
| GTAAGAGAATCAACTCCCACA | 87619846 | 87619868 | 0.03 |
| GATTCTCTTATTTCACCACAT | 87619860 | 87619882 | 0.29 |
| GTATTTCACCACATAGGTTCC | 87619867 | 87619889 | 0.70 |
| GTTTGATTCCTGGAACCTATG | 87619874 | 87619896 | 0.02 |
| GACGTCCTGAGTTTGATTCC | 87619885 | 87619907 | 0.02 |
| GGCAAAGGTGCTCATTGTCA | 87619910 | 87619932 | 0.81 |
| GGGATGGCTCTGAGGGCAA | 87619925 | 87619946 | 0.04 |
| GGCTGCTGGGATGGCTCTGA | 87619931 | 87619953 | 0.01 |
| GTAATTACGGGGCTGCTGGGA | 87619940 | 87619962 | 0.92 |
| GCCCCGTAATTATGATTATT | 87619951 | 87619973 | 0.22 |
| GTAATTATGATTATTAGGTG | 87619956 | 87619978 | 2.66 |
| GTTGCTTACTTGTAGCCGATC | 87619984 | 87620006 | 0.01 |
| GTGTATAATTTGTCCCGGAT | 87619998 | 87620020 | 2.41 |
| GCTGAGGTGCGGTAGAGGTGT | 87620020 | 87620042 | 0.43 |
| GGCTGACTGAGGTGCGGTAG | 87620026 | 87620048 | 0.21 |
| GAAGGAAGGCTGACTGAGGTG | 87620032 | 87620054 | 0.01 |

| | | | |
|---|---|---|---|
| GACACTAAGGAAGGCTGACTG | 87620037 | 87620059 | 0.99 |
| GAAACACACAAACACTAAGGA | 87620047 | 87620069 | 0.00 |
| GTGTTTGTGTGTTTACTGTG | 87620056 | 87620078 | 0.01 |
| GTGGACAAAGCGGCATTGTC | 87620080 | 87620102 | 0.25 |
| GCAATGCCGCTTTGTCCACGG | 87620085 | 87620107 | 0.01 |
| GCCTGGCCACCGTGGACAAAG | 87620090 | 87620112 | 0.02 |
| GATTAGCTTCCCTGGCCACCG | 87620099 | 87620121 | 6.61 |
| GGCCAGGGAAGCTAATGCTT | 87620106 | 87620128 | 2.37 |
| GTTGGGTGTTGAGAAAGAATC | 87620124 | 87620146 | 0.03 |
| GCTTTCCCCCTGAGCTCAGT | 87620147 | 87620169 | 0.00 |
| GGTTTCCAACTGAGCTCAGG | 87620152 | 87620174 | 0.01 |
| GAGCTCAGTTGGAAACCTCT | 87620158 | 87620180 | 0.00 |
| GTTGGAAACCTCTCGGCTGT | 87620165 | 87620187 | 0.31 |
| GATGTCATTCCCACAGCCGAG | 87620173 | 87620195 | 0.09 |
| GACATCACCACACTTCCCTC | 87620191 | 87620213 | 0.73 |
| GACAGTGGCCAGAGGGAAGTG | 87620198 | 87620220 | 0.05 |
| GTTTTTGGCACAGTGGCCAGA | 87620206 | 87620228 | 0.01 |
| GCCAGCTCCTTTTTGGCACAG | 87620214 | 87620236 | 0.01 |
| GCTGGCCTACCAGCTCCTTTT | 87620222 | 87620244 | 0.03 |
| GCTGGTAGGCCAGAAGCAG | 87620232 | 87620253 | 2.06 |
| GCCAGAAGCAGAGGAGGAAA | 87620240 | 87620262 | 0.02 |
| GCAGAGGAGGAAAAGGGTG | 87620247 | 87620268 | 0.02 |
| GAGGAAAAGGGTGTGGCCAC | 87620253 | 87620275 | 0.01 |
| GAAAGGGTGTGGCCACAGGTG | 87620258 | 87620280 | 0.02 |
| GTATCTGTGGCACCTCACCTG | 87620269 | 87620291 | 0.04 |
| GCATATTTCGTCAGGTATCTG | 87620283 | 87620305 | 0.02 |
| GAGGAAGTCATATTTCGTC | 87620292 | 87620313 | 11.94 |
| GGGGGTGTCAAGGGTGTAG | 87620310 | 87620331 | 0.00 |
| GGGGCGGATGGGGGTGTCAA | 87620318 | 87620340 | 0.02 |
| GCTCAGGGGGAGGGGGCGGA | 87620330 | 87620352 | 0.01 |
| GTTAAGCTCAGGGGGAGGGGG | 87620334 | 87620356 | 0.02 |
| GTAAAACTTAAGCTCAGGGGG | 87620340 | 87620362 | 0.01 |
| GTACTCTAAAACTTAAGCTC | 87620346 | 87620368 | 0.58 |
| GATCACACAAAACTCTGCTCT | 87620395 | 87620417 | 0.01 |
| GTGTGTGTGTACCGCATGCA | 87620466 | 87620488 | 0.01 |
| GCATGCATGGCTGTTGCCTG | 87620479 | 87620501 | 0.30 |
| GAGGATCTAACGGCCTCTTC | 87620505 | 87620527 | 0.03 |
| GGCCGTTAGATCCTCTGAAC | 87620513 | 87620535 | 0.02 |
| GCTCTGAACTGGCATTTCAGA | 87620525 | 87620547 | 0.01 |
| GACGGTTGTGAGCCTGCCTGT | 87620544 | 87620566 | 1.33 |
| GTGAGCCTGCCTGTAGGTAC | 87620550 | 87620572 | 0.00 |
| GTCAGTTCCCAGTACCTAC | 87620559 | 87620580 | 0.03 |
| GGTACTGGGAACTGACTCC | 87620565 | 87620586 | 0.04 |
| GTTACTCTTCTAGAGGGTCC | 87620582 | 87620604 | 0.03 |
| GTACTTTGTTACTCTTCTAG | 87620589 | 87620611 | 0.01 |
| GAAGCAGGAGCTGAAGAGA | 87620626 | 87620647 | 0.03 |
| GAAAAACAACAGCAAAGAAGC | 87620640 | 87620662 | 0.65 |
| GTTGCTGTTGTTTTTAAGCCA | 87620649 | 87620671 | 0.01 |
| GCTGAGCTATTTGAGACCT | 87620666 | 87620687 | 0.18 |
| GCTCACCTTCTAACTCACCA | 87620686 | 87620708 | 2.30 |
| GGAAGAAAATGAGTTGCCA | 87620703 | 87620724 | 1.75 |
| GTATAAATTCCAGTGTTTAGG | 87620733 | 87620755 | 1.52 |

| | | | |
|---|---|---|---|
| GAGTACTTCTTATCTGGGTG | 87620763 | 87620785 | 0.00 |
| GCGAAGGAGTACTTCTTATC | 87620769 | 87620791 | 0.01 |
| GATATTAATAAAAGGGGCGA | 87620785 | 87620807 | 0.86 |
| GTAATAAAGATATTAATAAA | 87620793 | 87620815 | 0.15 |
| GTATTACTTTTATTTATTTCA | 87620810 | 87620832 | 0.02 |
| GCTGTCTCAATACACACACTG | 87620911 | 87620933 | 0.01 |
| GCAGAAGTCTGTTCTCTGCCC | 87620945 | 87620967 | 0.02 |
| GAACCCAAGTGGCATTCCCA | 87620962 | 87620984 | 0.10 |
| GGGAATGCCACTTGGGTTCT | 87620966 | 87620988 | 0.00 |
| GACTCATTCCCAGAACCCAAG | 87620973 | 87620995 | 0.02 |
| GAGGCATCTGCTGTAAGCTG | 87621001 | 87621023 | 0.01 |
| GATTGTTCAGCGGTAAGGAG | 87621020 | 87621042 | 0.01 |
| GTGGTGATTGTTCAGCGGTA | 87621025 | 87621047 | 0.53 |
| GGTCTGTGGTGATTGTTCAG | 87621030 | 87621052 | 0.01 |
| GATAATAATACTAAGGGTCTG | 87621044 | 87621066 | 0.01 |
| GTTAAAAAATAATAATACTA | 87621052 | 87621074 | 0.05 |
| GTATTTTTTAACTTTACTATT | 87621064 | 87621086 | 0.01 |
| GTATCTAAAGAGAGTCAGGTA | 87621105 | 87621127 | 0.05 |
| GTAATATATCTAAAGAGAGTC | 87621110 | 87621132 | 0.04 |
| GTTTAGATATATTAGTTTTCT | 87621120 | 87621142 | 0.02 |
| GTTGTTTGTTTGTTTGCAGTT | 87621169 | 87621191 | 0.01 |
| GTTAGGGTCTCCTATAGCCC | 87621186 | 87621208 | 0.02 |
| GCCAAGAGAATCAGGAGGTCA | 87621242 | 87621264 | 0.01 |
| GATACAACCAAGAGAATCAGG | 87621248 | 87621270 | 0.02 |
| GTCTTGGTTGTATGCTAAGTT | 87621259 | 87621281 | 0.01 |
| GAGACATGTGTCACCACACC | 87621281 | 87621303 | 0.02 |
| GACAAATAAGCAGGCCAGGTG | 87621294 | 87621316 | 0.35 |
| GAAACCACAAATAAGCAGGCC | 87621299 | 87621321 | 0.01 |
| GCTTATTTGTGGTTTCATTT | 87621307 | 87621329 | 0.07 |
| GTGAATGTATATCATGCGTG | 87621377 | 87621399 | 1.06 |
| GCATGCGTGTGGGGGTGCCTA | 87621389 | 87621411 | 0.01 |
| GTGCCTATGGGTGCCAGAAG | 87621402 | 87621424 | 0.05 |
| GCCAAGTAGCTCTAGCTCCAG | 87621434 | 87621456 | 0.01 |
| GCTAGAGCTACTTGGGTTCT | 87621442 | 87621464 | 1.03 |
| GGGTTCTAGGAACTGAGTGT | 87621455 | 87621477 | 0.02 |
| GAACTGAGTGTGGGTCCTCC | 87621464 | 87621486 | 0.31 |
| GTGGGTCCTCCAGGGGAGC | 87621473 | 87621494 | 0.33 |
| GGTGCTTCCTGCTCCCCTGG | 87621479 | 87621501 | 0.02 |
| GGAAGCACCCTTGACCTCTT | 87621493 | 87621515 | 1.65 |
| GAGATGGCCAAAGAGGTCAA | 87621500 | 87621522 | 0.01 |
| GGGGCTGGAGATGGCCAAAG | 87621507 | 87621529 | 0.01 |
| GCAAATAAAGGGGGCTGGAGA | 87621516 | 87621538 | 0.12 |
| GAAAATCCAAATAAAGGGGGC | 87621522 | 87621544 | 0.02 |
| GCTTTATAAAATCCAAATAA | 87621529 | 87621551 | 0.55 |
| GTCTCAGTGTATATCCCAGAC | 87621552 | 87621574 | 0.01 |
| GTAGGAGTTGCCGGCCAGTC | 87621566 | 87621588 | 0.36 |
| GCCGGCAACTCCTACTGTGC | 87621574 | 87621596 | 0.03 |
| GTCAAGGTCAGCCGGCACAGT | 87621584 | 87621606 | 0.01 |
| GATTGTGAGTTCAAGGTCAGC | 87621593 | 87621615 | 0.01 |
| GCAGGAGATATTGTGAGTTCA | 87621601 | 87621623 | 0.00 |
| GTACTTGGGAGGATGGCTC | 87621620 | 87621641 | 0.02 |
| GAATCTCTGTACTTGGGAGGA | 87621626 | 87621648 | 0.01 |

| | | | |
|---|---|---|---|
| GCAGGCATGAGTCACCTCACC | 87621650 | 87621672 | 5.51 |
| GCTCTGGATGAAAGCCAGGTG | 87621663 | 87621685 | 0.01 |
| GAAAACCTCTGGATGAAAGCC | 87621668 | 87621690 | 0.01 |
| GTTCATCCAGAGGTTTTCCTA | 87621675 | 87621697 | 0.01 |
| GCTAGACCGTAGGAAAACCTC | 87621680 | 87621702 | 0.01 |
| GGAAGATCATTCTAGACCGT | 87621691 | 87621713 | 0.01 |
| GAACCCAAAATCTGAAGAAA | 87621712 | 87621734 | 0.10 |
| GAAAAAGAAGAAAACTGAGAT | 87621743 | 87621765 | 0.00 |
| GTTTAGGGAATTAATTCATTC | 87621764 | 87621786 | 0.00 |
| GTCATTAATATTTAAGATTG | 87621800 | 87621822 | 0.02 |
| GATACAAACATAGATACAT | 87621894 | 87621915 | 0.02 |
| GATTGTTAGGGCCTGGCGTGG | 87622135 | 87622157 | 0.08 |
| GTTAAGAATATTGTTAGGGCC | 87622143 | 87622165 | 0.01 |
| GAAGTTTAAGAATATTGTTA | 87622148 | 87622170 | 0.59 |
| GCTAAATGTTGGTGCTCAGG | 87622170 | 87622192 | 0.50 |
| GAGGTCGCTGAGCTAAATGT | 87622181 | 87622203 | 0.33 |
| GGAGCTCATATCTGAACATG | 87622200 | 87622222 | 0.49 |
| GTTCAGATATGAGCTCCCTCT | 87622207 | 87622229 | 0.00 |
| GCCTGGCTGAAGACTCCAAG | 87622222 | 87622244 | 0.01 |
| GTGGGGACTGGAGCAGAGCC | 87622239 | 87622261 | 0.02 |
| GCAAAGACAGGAAGTGGGGAC | 87622251 | 87622273 | 0.69 |
| GTTCACAAAGACAGGAAGTG | 87622256 | 87622278 | 0.08 |
| GATTGTCCCGTTCACAAAGAC | 87622264 | 87622286 | 0.01 |
| GCAATCTACAGTTCCATCAGA | 87622283 | 87622305 | 0.01 |
| GTTCCATCAGATGGGAAAAGT | 87622293 | 87622315 | 0.01 |
| GAGCTCAGGGAAGAGTAATG | 87622323 | 87622345 | 0.65 |
| GCCACAGAACGTAGAGCTCA | 87622336 | 87622358 | 0.78 |
| GACGAGAGGTGTTTGGGAATA | 87622358 | 87622380 | 0.25 |
| GAACAGGCACGAGAGGTGTT | 87622366 | 87622388 | 0.10 |
| GACGAGGAACAGGCACGAG | 87622373 | 87622394 | 2.31 |
| GGTCATGGAGACGAGGAAC | 87622382 | 87622403 | 0.04 |
| GAGAGATGGTCATGGAGACG | 87622388 | 87622410 | 0.23 |
| GACACAGTGGAGAGATGGTCA | 87622396 | 87622418 | 1.37 |
| GCTCTCCACTGTGTTTAGCTC | 87622406 | 87622428 | 0.03 |
| GGACCTGAGCTAAACACAG | 87622410 | 87622431 | 0.01 |
| GTGTGAGCTTTCCTGTCACG | 87622433 | 87622455 | 0.00 |
| GTTTCCTGTCACGTGGCTTCC | 87622441 | 87622463 | 0.05 |
| GGCTTCCAGGCCTCCTCCTC | 87622454 | 87622476 | 0.01 |
| GTAGAGCCAGAGGAGGAGGCC | 87622459 | 87622481 | 0.12 |
| GATTGCTTTAGAGCCAGAGG | 87622467 | 87622489 | 0.02 |
| GATCTTCCATATTGCTACCAA | 87622487 | 87622509 | 0.01 |
| GAAATTCCTTTGGTAGCAATA | 87622492 | 87622514 | 10.16 |
| GATGGATGGAGAAATTCCTT | 87622503 | 87622525 | 0.02 |
| GATTGTGAGTGATCAGATGGA | 87622517 | 87622539 | 0.01 |
| GATCACTCACAATCACTCCCC | 87622528 | 87622550 | 0.03 |
| GTCTTGCTGATGACTAACCTG | 87622544 | 87622566 | 10.85 |
| GATAACATCCAAATTTATCC | 87622565 | 87622587 | 0.03 |
| GCTTATCACCCTGGATAAATT | 87622573 | 87622595 | 0.01 |
| GCAAAGTGATCCTTATCACCC | 87622583 | 87622605 | 0.02 |
| GATGGCTGATCTGACGGAAA | 87622606 | 87622628 | 0.21 |
| GGAAAGATGGCTGATCTGA | 87622612 | 87622633 | 0.01 |
| GTTATTATAGTGTAGGAAAGA | 87622624 | 87622646 | 0.09 |

| | | | |
|---|---|---|---|
| GGCTAGATTATTATAGTGT | 87622632 | 87622653 | 0.03 |
| GAAGCTAAACATAGTTGGCTG | 87622652 | 87622674 | 1.19 |
| GTTTGGGAAGCTAAACATAGT | 87622658 | 87622680 | 0.02 |
| GTCAACTCTATGACAACTTT | 87622675 | 87622697 | 8.62 |
| GAAAAACAAAAACAAAAGCT | 87622697 | 87622719 | 0.53 |
| GAGACAGAGTATTGCTGAG | 87622729 | 87622750 | 0.00 |
| GTATTGCTGAGTGGCAGAGTC | 87622738 | 87622760 | 0.08 |
| GTAGTAAATAGCTTGTGCC | 87622778 | 87622799 | 0.06 |
| GCACGGGATCAGCATGGTGCC | 87622795 | 87622817 | 0.21 |
| GAATCACTCACGGGATCAGCA | 87622802 | 87622824 | 0.35 |
| GGCTCTGCCCAATCACTCAC | 87622812 | 87622834 | 0.68 |
| GTGATTGGGCAGAGCCTGG | 87622819 | 87622840 | 0.01 |
| GAATTACGGGTGTGATCCACC | 87622833 | 87622855 | 0.01 |
| GATCACACCCGTAATTAAGAG | 87622841 | 87622863 | 0.02 |
| GTAATTAAGAGTGGAGGCAG | 87622850 | 87622872 | 0.01 |
| GAGTGGAGGCAGAGGAAGC | 87622858 | 87622879 | 0.02 |
| GCAGAGGAAGCAGGCCAACCC | 87622867 | 87622889 | 0.01 |
| GTCTTGCTTGGTAACCTGGGT | 87622880 | 87622902 | 0.01 |
| GTTCTTAAGCAAGGTCTTGCT | 87622893 | 87622915 | 0.02 |
| GTTGTTTGTTTCTTAAGCA | 87622903 | 87622924 | 0.02 |
| GACAAACAACAATAAAAATAG | 87622916 | 87622938 | 0.03 |
| GAACAATAAAAATAGAGGGCT | 87622922 | 87622944 | 0.04 |
| GTTAAGTAAAAAGTTTAATCA | 87622945 | 87622967 | 0.03 |
| GAAGTTTTCTGCCTAACCCAG | 87622973 | 87622995 | 2.98 |
| GAAAGAACTAGCCGCTGGGTT | 87622983 | 87623005 | 0.01 |
| GATTAAAAAGAACTAGCCGC | 87622989 | 87623011 | 0.01 |
| GTTCTTTTTAATCTCTGCGCT | 87623000 | 87623022 | 0.06 |
| GTCTCTGCGCTTGGGAGGCAG | 87623010 | 87623032 | 0.10 |
| GGGTCTCTTTACTCAGCTC | 87623084 | 87623105 | 0.23 |
| GTGTGTAGAGGTGTAGAGAC | 87623104 | 87623126 | 1.10 |
| GCTTGAGTCAGAGTCTTCCC | 87623184 | 87623206 | 0.03 |
| GTCTTCCCAGGGTCTCTCC | 87623196 | 87623217 | 0.14 |
| GGTCAGCCTGGAGAGACCCT | 87623201 | 87623223 | 0.02 |
| GTAGTTTATGAAGTGAGTTCT | 87623222 | 87623244 | 0.06 |
| GACTCACTTCATAAACTAGAC | 87623228 | 87623250 | 0.02 |
| GTCTCAGTACTCTAGAAGCTG | 87623276 | 87623298 | 0.01 |
| GAAGTTTCTAAAAGTGGGCAT | 87623313 | 87623335 | 0.01 |
| GTTGTAGTAAGTTTCTAAAAG | 87623320 | 87623342 | 0.02 |
| GTAATAAAATGTCATTAGACT | 87623402 | 87623424 | 0.04 |
| GTCAGAGCGTCTTAACCAGAT | 87623426 | 87623448 | 0.04 |
| GTTTTAAATCAGAAGCCTATC | 87623440 | 87623462 | 0.01 |
| GAAAAATCACGTTTATTTAAT | 87623463 | 87623485 | 0.01 |
| GCATGTGACATGATGCACATG | 87623594 | 87623616 | 0.01 |
| GATGCACATGTGGAAGTCAA | 87623604 | 87623626 | 0.01 |
| GAAGTCAAAGGGCAACTTGC | 87623616 | 87623638 | 0.03 |
| GTAGTTGCCTGTTCATCATGT | 87623641 | 87623663 | 0.01 |
| GCTGTTCATCATGTAGGGCCT | 87623648 | 87623670 | 0.01 |
| GGAGACTGAGTTTAATTCCG | 87623665 | 87623687 | 0.01 |
| GAATTAAACTCAGTCTCCTC | 87623670 | 87623692 | 0.01 |
| GAAACTCAGTCTCCTCTGGCT | 87623675 | 87623697 | 0.05 |
| GGCACTTGCCACCAAGCCAG | 87623686 | 87623708 | 0.01 |
| GCAAAATGGTTCGGCAGGTA | 87623708 | 87623730 | 0.01 |

| | | | |
|---|---|---|---|
| GGGCAGCAAAATGGTTCGGC | 87623713 | 87623735 | 0.69 |
| GGGGGGGGGGGGCAGCAAAA | 87623722 | 87623744 | 0.12 |
| GATATCATTTGGCGGGGGGG | 87623739 | 87623761 | 0.01 |
| GATCAAAATATCATTTGGCGG | 87623745 | 87623767 | 0.02 |
| GCCAAATGATATTTTGATAT | 87623750 | 87623772 | 0.02 |
| GTATTATATAATGTCTGAATC | 87623777 | 87623799 | 0.01 |
| GATAAATGAGAATGTTTGAGG | 87623813 | 87623835 | 1.39 |
| GCAAACATTCTCATTTATTTG | 87623819 | 87623841 | 2.50 |
| GTGGAAAGCTTTTAAATGTT | 87623859 | 87623881 | 0.13 |
| GCAGACTGGAGTTAGTTACAG | 87623878 | 87623900 | 0.49 |
| GTAACTAACTCCAGTCTGAG | 87623883 | 87623905 | 0.01 |
| GCTGGCTGCATCCTCTCAGAC | 87623893 | 87623915 | 0.91 |
| GATGTATGCGCATCACAAAGC | 87623912 | 87623934 | 0.01 |
| GATGCGCATACATGCATGC | 87623922 | 87623943 | 0.02 |
| GTCTAATTGTATGTGCATACA | 87623948 | 87623970 | 0.03 |
| GAATGATCATGTGCTGCTGTT | 87623979 | 87624001 | 0.08 |
| GCCTACATAAGAGAGTGATTG | 87624004 | 87624026 | 0.42 |
| GTAGGAAAACATCAGAATAT | 87624022 | 87624044 | 0.37 |
| GACAAGAAATAACGCTAACAT | 87624049 | 87624071 | 0.14 |
| GCGTTATTTCTTGTGGGGTC | 87624058 | 87624080 | 0.20 |
| GATTTCTTGTGGGGTCTGGGC | 87624063 | 87624085 | 0.50 |
| GGGTCTGGGCAGGAATCCCA | 87624073 | 87624095 | 0.04 |
| GCTCTGTGTGCAAAAGGCCTT | 87624089 | 87624111 | 0.00 |
| GAGCATACTCTGTGTGCAAA | 87624096 | 87624118 | 0.14 |
| GTTGAGGAATGCAGTCAGTG | 87624121 | 87624143 | 0.12 |
| GTAAACAATGAATGGGTTG | 87624137 | 87624158 | 0.89 |
| GAATGAATGTAAACAATGAAT | 87624143 | 87624165 | 0.77 |
| GATGTCCATCTTGTGTGTATG | 87624219 | 87624241 | 0.00 |
| GTATGTGGGTGGACACGGCA | 87624234 | 87624256 | 0.14 |
| GGTGGACACGGCATGGTTAG | 87624241 | 87624263 | 0.01 |
| GGTTAGAGGGGAGATTTGTG | 87624255 | 87624277 | 1.29 |
| GTCTCTTCTTTCATCTTCATG | 87624283 | 87624305 | 0.80 |
| GGCAGTTGTCATGCAAGAC | 87624327 | 87624348 | 0.01 |
| GTTTAAATGCTTTGCAGGTAA | 87624347 | 87624369 | 0.01 |
| GACTTTGTTTAAATGCTTTGC | 87624353 | 87624375 | 0.02 |
| GAACTAGGAATAAACGTACAG | 87624402 | 87624424 | 0.01 |
| GTATTTTAAGACAGCAAACT | 87624418 | 87624440 | 0.02 |
| GAAACAAACAAACTAAAGATA | 87624466 | 87624488 | 0.07 |
| GCCAGAGCAAGAGGGAAAAG | 87624492 | 87624514 | 0.01 |
| GACGAGTGGGGCCAGAGCAAG | 87624501 | 87624523 | 0.01 |
| GCCTATGGGCTGGAACGAGTG | 87624514 | 87624536 | 0.06 |
| GAAAAAAAAAACCCTATGGGC | 87624525 | 87624547 | 0.34 |
| GTTTTTGTTTCTCATTATGTA | 87624558 | 87624580 | 0.01 |
| GAAAAATAAGGAATGGTGAGA | 87624657 | 87624679 | 0.02 |
| GTTCCTTATTTTTAGTTTTTA | 87624668 | 87624690 | 0.03 |
| GTTTAAGGTTTATTAGTTATA | 87624684 | 87624706 | 0.01 |
| GTCACTGTTCCAGGACAGCCA | 87624766 | 87624788 | 0.01 |
| GAACAGTGAGTAGACCAAGC | 87624780 | 87624802 | 0.02 |
| GTCTCTGCCTTGGGAGTGCT | 87624825 | 87624847 | 0.05 |
| GAAAATAGGGCCGGGCGGTGG | 87624862 | 87624884 | 0.01 |
| GAATATAAAATAGGGCCGGG | 87624868 | 87624890 | 0.05 |
| GATCAAATAGAATATAAAATA | 87624876 | 87624898 | 1.73 |

| | | | |
|---|---|---|---|
| GGTTGTAAAAAAGGATAATC | 87624901 | 87624923 | 0.05 |
| GGCGGAACTGGTTGTAAAAA | 87624910 | 87624932 | 0.01 |
| GAGGTCTGACAGGCGGAAC | 87624922 | 87624943 | 0.71 |
| GTGATACGAGGTCTGACAGG | 87624928 | 87624950 | 0.05 |
| GCTATATATGAAAGTGATACG | 87624940 | 87624962 | 0.32 |
| GAAGATCTCCTATCCACAC | 87624962 | 87624983 | 0.01 |
| GGTCAGAGCCTGTGTGGAT | 87624970 | 87624991 | 0.41 |
| GCTACGTGGTCAGAGCCTGTG | 87624975 | 87624997 | 0.01 |
| GTCTGACCACGTAGTCCTGAC | 87624985 | 87625007 | 0.45 |
| GCCAAGCCAGTCAGGACTACG | 87624990 | 87625012 | 0.04 |
| GGAAGTCACTACCCAGAGC | 87625011 | 87625032 | 0.01 |
| GACGTGTAAGCCAGCCGGCTC | 87625023 | 87625045 | 0.42 |
| GCTGGCTTACACGTAGTTTA | 87625032 | 87625054 | 0.01 |
| GAAGAGGTCATCATTACAGA | 87625149 | 87625171 | 0.00 |
| GAAGAGTACTGACGGCTCTTC | 87625215 | 87625237 | 0.02 |
| GTCAGAGGGTAAGAGTACTGA | 87625224 | 87625246 | 0.16 |
| GAATTAAAATAAATAAGGGGC | 87625256 | 87625278 | 0.32 |
| GTTTTAAAACAGTTTTCTACC | 87625283 | 87625305 | 0.01 |
| GCAATTCTCATTCTAACTTCC | 87625301 | 87625323 | 2.40 |
| GATTGTGTGATACTAAAGGAT | 87625320 | 87625342 | 0.01 |
| GCTAGTTTCAGCATCTTTGTC | 87625346 | 87625368 | 0.00 |
| GAGGGTGTTAAATGGTTAT | 87625382 | 87625403 | 0.01 |
| GTCCTAAAGAGGGTGTTAAA | 87625389 | 87625411 | 0.03 |
| GTGTCCGTGGAGTCCTAAAG | 87625400 | 87625422 | 0.01 |
| GCACCATATCTTTTTTTCTTA | 87625438 | 87625460 | 0.01 |
| GGAAAAAAAAAGTCACAGC | 87625459 | 87625480 | 0.03 |
| GAAGTCACAGCTGGACATTGA | 87625468 | 87625490 | 0.46 |
| GAATGCTGGATCTAAAGCTAA | 87625497 | 87625519 | 0.01 |
| GCTTTAGATCCAGCATTCAGG | 87625504 | 87625526 | 0.02 |
| GGAGGATTACTTGAGTCAAG | 87625535 | 87625557 | 0.11 |
| GAAGGGGTTTGAGACCAGCCT | 87625552 | 87625574 | 0.07 |
| GTCTCTTTGAATTGCCTAGGC | 87625565 | 87625587 | 0.01 |
| GAAAAAGAAAAGAAATCCCA | 87625603 | 87625625 | 0.69 |
| GAAAAGAAATCCCATGGGAAC | 87625610 | 87625632 | 0.01 |
| GCCTTTGAACCAGTTCCCAT | 87625619 | 87625641 | 0.56 |
| GAGGCACTACTGGGATCAAA | 87625645 | 87625667 | 0.44 |
| GATCCCAGTAGTGCCTCTGTG | 87625652 | 87625674 | 0.27 |
| GCATACATCTAATCCACACAG | 87625664 | 87625686 | 0.43 |
| GCCGTGTAACACAGACAGACA | 87625698 | 87625720 | 0.01 |
| GTCTGTCTGTGTTACACGGTA | 87625703 | 87625725 | 0.01 |
| GAAAAAAAAACACAGGGAAGG | 87625727 | 87625749 | 0.01 |
| GTTTTCTAAGAACTGCCTTCT | 87625752 | 87625774 | 0.04 |
| GACTTCTCTTCACCCTAGA | 87625766 | 87625787 | 0.06 |
| GCTGCAGTCAACCCAAGTGTG | 87625794 | 87625816 | 0.01 |
| GCCGTCAGACACCCACACACT | 87625805 | 87625827 | 0.03 |
| GTGGGTGTCTGACGGCCACC | 87625813 | 87625835 | 0.01 |
| GGACAGGAGATCTTGCCTGG | 87625828 | 87625850 | 0.01 |
| GCCAAGAGTTGTAGGAGGAC | 87625844 | 87625866 | 0.02 |
| GCTTCCTGCCAAGAGTTGT | 87625852 | 87625873 | 1.08 |
| GACTCTTGGCAGGAAGCAGAG | 87625858 | 87625880 | 0.02 |
| GGCAGGAAGCAGAGAGGGAG | 87625864 | 87625886 | 0.02 |
| GCAGAGAGGGAGAGGTCCTG | 87625872 | 87625894 | 0.04 |

| | | | |
|---|---|---|---|
| GGAGAGGTCCTGTGGCTACC | 87625880 | 87625902 | 0.29 |
| GAACAGCCCTGGTAGCCAC | 87625888 | 87625909 | 0.01 |
| GCAATAAACTAGAACAGCCC | 87625898 | 87625920 | 0.60 |
| GAAGCAAAGTACAACTATTTA | 87625953 | 87625975 | 0.01 |
| GTACAACTATTTATGGCCATG | 87625961 | 87625983 | 0.13 |
| GTATCAGTTTCTCTATCCACA | 87625976 | 87625998 | 0.57 |
| GACTGATACCCAATGCCCGTG | 87625992 | 87626014 | 0.73 |
| GTATTCTTCCACACGGGCATT | 87625999 | 87626021 | 0.02 |
| GATACTTTATTCTTCCACAC | 87626006 | 87626028 | 0.02 |
| GAAGCTGAGCCCTGATCCATA | 87626035 | 87626057 | 0.01 |
| GCCACAGAGCCTTATGGATCA | 87626043 | 87626065 | 0.33 |
| GCTGATCCCCACAGAGCCTTA | 87626050 | 87626072 | 0.01 |
| GCTCTGTGGGGATCAGCTGG | 87626057 | 87626079 | 0.01 |
| GTGGGGATCAGCTGGAGGCT | 87626062 | 87626084 | 0.03 |
| GATCAGCTGGAGGCTGGGAAC | 87626068 | 87626090 | 1.96 |
| GCTTATATCCGACATGCAAA | 87626092 | 87626114 | 0.01 |
| GTTAGCGAGCCATTTGCATGT | 87626100 | 87626122 | 0.01 |
| GACAGTTGACTACTAAGACAC | 87626122 | 87626144 | 1.22 |
| GCTTCCATGTTGACTGCAGTG | 87626145 | 87626167 | 0.17 |
| GTTGACTGCAGTGAGGCCATA | 87626153 | 87626175 | 0.00 |
| GCAGTGAGGCCATATGGGC | 87626159 | 87626180 | 0.01 |
| GCCTATAATCACCAGCCCATA | 87626168 | 87626190 | 0.01 |
| GCTTTACAGCCCACAGACCT | 87626190 | 87626212 | 0.09 |
| GAGACCTGATCCTAGGTCTG | 87626200 | 87626222 | 0.00 |
| GGACTACGAGACCTGATCCT | 87626207 | 87626229 | 0.34 |
| GCAAAGCTATCTTAGTGTAT | 87626228 | 87626250 | 0.04 |
| GCCAGTGACATGGGGGAAACT | 87626253 | 87626275 | 0.04 |
| GTTTTGTGTTTCCAGTGACA | 87626264 | 87626286 | 0.01 |
| GCAAAACATCTGTTCATACTC | 87626281 | 87626303 | 0.04 |
| GAGATTCAGTGAGAAAAAGA | 87626303 | 87626325 | 0.01 |
| GAGAAAAAGAAGGAAGCACA | 87626313 | 87626335 | 0.01 |
| GACTTCACTAGCAGCTGGTA | 87626341 | 87626363 | 0.03 |
| GCCCAGACTTCACTAGCAGC | 87626346 | 87626368 | 1.76 |
| GCTAGTGAAGTCTGGGCAG | 87626352 | 87626373 | 0.00 |
| GTTTTTTTTTTCAGGGTCTTA | 87626374 | 87626396 | 0.02 |
| GACCCTGAAAAAAAAAATCC | 87626380 | 87626402 | 0.09 |
| GAAAAAAAAATCCCGGGTGAG | 87626388 | 87626410 | 0.02 |
| GCAACAAAAATCCTCTCACCC | 87626398 | 87626420 | 0.11 |
| GAAAATAATCCTAGCCGGGCG | 87626588 | 87626610 | 0.07 |
| GCTTAAAAAATAATCCTAGCC | 87626593 | 87626615 | 0.01 |
| GGGTGTGTGCACTGGAGTGC | 87626662 | 87626684 | 0.00 |
| GCAGGTGTCCCCAGAAGCCAG | 87626681 | 87626703 | 0.01 |
| GCCTGACACCTCTGGCTTCTG | 87626688 | 87626710 | 0.79 |
| GCCACGGGCACCTGACACCTC | 87626697 | 87626719 | 0.32 |
| GTGTCAGGTGCCCGTGGAAC | 87626703 | 87626725 | 0.84 |
| GCCCGTGGAACTGGAACCCC | 87626712 | 87626734 | 0.01 |
| GACTGGAACCCCAGGCAGCTG | 87626721 | 87626743 | 4.22 |
| GCGGCACCCCACAGCTGCCT | 87626729 | 87626751 | 0.12 |
| GGGGTGCCGCTCAACATGGG | 87626742 | 87626764 | 0.46 |
| GTCAACATGGGTGGTCTGCAT | 87626752 | 87626774 | 1.79 |
| GGGTGGTCTGCATAGGTAAC | 87626759 | 87626781 | 1.05 |
| GCATAGGTAACAGGTCCTA | 87626768 | 87626789 | 0.19 |

| | | | |
|---|---|---|---|
| GTCCTAAGGAAGAAAAGAAC | 87626781 | 87626803 | 0.02 |
| GTATTCTAAGAGCTGGAGAGA | 87626819 | 87626841 | 0.01 |
| GAAAAATATATTCTAAGAGC | 87626827 | 87626849 | 0.36 |
| GAGAGAGAACTGACTCTCAC | 87626939 | 87626961 | 0.01 |
| GTTCTCTCTCTCCACCATAA | 87626952 | 87626974 | 0.23 |
| GCTCTCCACCATAAGGGTCTC | 87626959 | 87626981 | 4.01 |
| GTTCAATTCCTGAGACCCTTA | 87626966 | 87626988 | 0.16 |
| GGGTCTCAGGAATTGAACTT | 87626972 | 87626994 | 0.03 |
| GTTGAACTTAGGTTTCAAGCT | 87626984 | 87627006 | 0.03 |
| GAAGCTTGGTGGCAAGTGCCT | 87626999 | 87627021 | 0.02 |
| GAGGTGGCTCAGTAGGCCA | 87627016 | 87627037 | 0.02 |
| GAGCAGTGAGGTGGCTCAGT | 87627022 | 87627044 | 0.06 |
| GAATGCTTGGGAGCAGTGAGG | 87627031 | 87627053 | 1.06 |
| GTTATAATTCTCTTAATGCTT | 87627044 | 87627066 | 0.04 |
| GTAAGAGAATTATAATGAACG | 87627053 | 87627075 | 0.02 |
| GAATTATAATGAACGTGGGGC | 87627059 | 87627081 | 0.01 |
| GAACGTGGGGCTGGAAAGAT | 87627068 | 87627090 | 1.10 |
| GCACGGGTTGCCTCTTCCA | 87627105 | 87627126 | 0.01 |
| GTTGCCTCTTCCAAGGAACC | 87627111 | 87627133 | 0.01 |
| GGAATCGAACCCAGGTTCCT | 87627121 | 87627143 | 0.02 |
| GTCGGCGCCATGTGGGTGTT | 87627142 | 87627164 | 0.01 |
| GTGGTTGGTCGGCGCCATGT | 87627149 | 87627171 | 0.67 |
| GAAACTGGAATGACAGGTGGT | 87627164 | 87627186 | 0.01 |
| GCCTGAAACTGGAATGACAGG | 87627168 | 87627190 | 0.43 |
| GCCATAGGATGTCCTGAAAC | 87627180 | 87627202 | 0.03 |
| GTGTATGCAGAAGAGGCCAT | 87627195 | 87627217 | 0.01 |
| GTAGCATGTGTATGCAGAAG | 87627202 | 87627224 | 0.73 |
| GTTGTATGTACATAGGTGCTT | 87627241 | 87627263 | 0.01 |
| GATTAATTATTGTATGTACAT | 87627249 | 87627271 | 9.06 |
| GAATGATCTAGAACTGAATAT | 87627290 | 87627312 | 0.57 |
| GGCATGACTGGTATCAGTC | 87627313 | 87627334 | 0.32 |
| GCTGCATAAAGCAGGCATGAC | 87627324 | 87627346 | 0.01 |
| GATAACCCTGCTGCATAAAGC | 87627333 | 87627355 | 0.02 |
| GATGCAGCAGGGTTATCAATC | 87627341 | 87627363 | 0.01 |
| GTCTTTATGTATTTTTCTTG | 87627382 | 87627404 | 0.01 |
| GACGACTAGGATTCAACAAGA | 87627412 | 87627434 | 0.01 |
| GCCGGCTCTGTGGAGACGACT | 87627426 | 87627448 | 0.01 |
| GTAAGCAATTAACCGGCTCTG | 87627437 | 87627459 | 0.29 |
| GCACTTTAATAAGCAATTAAC | 87627445 | 87627467 | 0.09 |
| GTATTAAAGTGGACGCATTTC | 87627458 | 87627480 | 2.43 |
| GCAGACCCGTAACTTAGAACT | 87627489 | 87627511 | 0.32 |
| GCTGCCCGAGTTCTAAGTTA | 87627494 | 87627516 | 0.01 |
| GACTCGGGCAGCAGAGATAGG | 87627506 | 87627528 | 0.03 |
| GCAGCAGAGATAGGAGGATGA | 87627513 | 87627535 | 0.01 |
| GTTTTGCCAAGAAAAAAGGA | 87627548 | 87627570 | 0.03 |
| GCTTTTTCCCTCCTTTTTTCT | 87627554 | 87627576 | 0.01 |
| GAAGGAGGGAAAAAGGAATTT | 87627563 | 87627585 | 0.01 |
| GAATTTTGGGCTGAGTGTGG | 87627577 | 87627599 | 1.69 |
| GACATGTTTGTAATGTCAGT | 87627600 | 87627622 | 0.02 |
| GTTTGTAATGTCAGTAGGGT | 87627605 | 87627627 | 0.01 |
| GAATCAGAGAGCGTCACGTC | 87627641 | 87627663 | 1.79 |
| GAGAGCGTCACGTCAGGGTG | 87627647 | 87627669 | 0.15 |

| | | | |
|---|---|---|---|
| GGGGAGCCACCTTCAACTCC | 87627668 | 87627690 | 0.02 |
| GTCTTACCCTGGAGTTGAAGG | 87627674 | 87627696 | 1.78 |
| GTCCAGGGTAAGACCGTGTCT | 87627685 | 87627707 | 0.00 |
| GTTTTGTCAGTTTCCGAGACA | 87627697 | 87627719 | 0.01 |
| GCACATCTCTCCAGACTCC | 87627722 | 87627743 | 0.01 |
| GAAAGGAAACTTCCGGGAGTC | 87627732 | 87627754 | 1.52 |
| GAAAGGGAAAGGAAACTTCC | 87627739 | 87627761 | 0.02 |
| GTTCTGGTTGAGAAAGGGAA | 87627750 | 87627772 | 0.01 |
| GTCTTGGTTCTGGTTGAGAAA | 87627755 | 87627777 | 0.01 |
| GCAACCAGAACCAAGAAAGGC | 87627763 | 87627785 | 0.01 |
| GGAGGTAGCCTGCCTTTCT | 87627772 | 87627793 | 0.01 |
| GAAAATCAAGCACAGGGGTG | 87627792 | 87627814 | 0.10 |
| GAAAGAAGAAAATCAAGCAC | 87627799 | 87627821 | 0.01 |
| GGAAGAGTATAAAGAATTAG | 87627829 | 87627851 | 0.06 |
| GTTGGCAGTCATCAGGACTG | 87627850 | 87627872 | 0.01 |
| GAACACCAGTTGGCAGTCATC | 87627857 | 87627879 | 0.22 |
| GACTGCCAACTGGTGTTACA | 87627863 | 87627885 | 0.01 |
| GCATCACCCTGTAACACCAGT | 87627868 | 87627890 | 0.01 |
| GATGACGGGCAGACCCCCG | 87627887 | 87627908 | 0.92 |
| GCCAGCTTGACAGTGTCCTC | 87627902 | 87627924 | 0.75 |
| GAAGCTGGCACATCCAGAGAC | 87627917 | 87627939 | 0.76 |
| GACAAACATCATCCCAGTCTC | 87627929 | 87627951 | 0.01 |
| GACTGGGATGATGTTTGTGTG | 87627935 | 87627957 | 0.10 |
| GATGTTTGTGTGTGGTTCAT | 87627943 | 87627965 | 0.00 |
| GATGTATAGCTCAGCCTCCAG | 87627973 | 87627995 | 0.01 |
| GCTCAGCCTCCAGAGGAGA | 87627980 | 87628001 | 1.54 |
| GCAACGCTCCTTCTCCTCTGG | 87627986 | 87628008 | 0.02 |
| GTTCTGAACATGTTGATATAG | 87628036 | 87628058 | 0.00 |
| GATAGAGGGACACTCAAGAGA | 87628052 | 87628074 | 5.12 |
| GAGACGGGACAGATCATTCC | 87628068 | 87628090 | 0.02 |
| GCTTAGACGGCTGGCCTTCC | 87628086 | 87628108 | 0.01 |
| GAAAACTCATGCTTAGACGGC | 87628095 | 87628117 | 0.02 |
| GTTTATTTTGTCTCCTTATCA | 87628145 | 87628167 | 0.00 |
| GCTGTTTGCAATCCATGATA | 87628157 | 87628179 | 0.20 |
| GATTAGCTCTCAACCAGCTTC | 87628186 | 87628208 | 0.01 |
| GCTACGGTTTCTCCTGAAGC | 87628198 | 87628220 | 0.18 |
| GCAAACAGATCTATTGCTA | 87628214 | 87628235 | 0.00 |
| GTAGATCTGTTTGCTGCTGAG | 87628223 | 87628245 | 0.06 |
| GCTGAGTGGTTACTGAGAG | 87628237 | 87628258 | 0.01 |
| GGCATATGTAGGTGACTACA | 87628265 | 87628287 | 0.01 |
| GCCTCACTGCCTGGCATATGT | 87628276 | 87628298 | 0.02 |
| GTTTAATGACCTCACTGCC | 87628286 | 87628307 | 1.72 |
| GCATGAAAGCCGCCATGTCTG | 87628307 | 87628329 | 1.26 |
| GACCTGACGCACCTCAGACA | 87628318 | 87628340 | 0.31 |
| GAGGTGCGTCAGGTCTCTTC | 87628326 | 87628348 | 0.01 |
| GAGAGGGTGGGTCTTTATG | 87628367 | 87628388 | 0.50 |
| GATCACAGGATCAGAGAGGGT | 87628378 | 87628400 | 0.01 |
| GCTGATCCTGTGATAAGGTAG | 87628388 | 87628410 | 3.16 |
| GCTTCACCTCTACCTTATCAC | 87628393 | 87628415 | 0.04 |
| GTAGGAATTGGCAGACAGGAT | 87628418 | 87628440 | 0.03 |
| GGAAGTAGGAATTGGCAGAC | 87628423 | 87628445 | 0.01 |
| GACTTAGAAGGAAGTAGGAAT | 87628431 | 87628453 | 0.01 |

| | | | |
|---|---|---|---|
| GCTGTAACTTAGAAGGAAGT | 87628437 | 87628459 | 0.03 |
| GATCACGCTGTAACTTAGA | 87628444 | 87628465 | 0.50 |
| GCTGAAACCAACTGTTTCAGC | 87628465 | 87628487 | 0.01 |
| GTTTGAGCCAGCTGAAACAGT | 87628471 | 87628493 | 4.77 |
| GCTTTGCCTGCATCTATGTT | 87628550 | 87628572 | 0.01 |
| GTGGCCCAAACATAGATGC | 87628556 | 87628577 | 0.01 |
| GTTGGGCCACATGTGTGTACC | 87628569 | 87628591 | 0.24 |
| GGACACCAGGTACACACATG | 87628574 | 87628596 | 0.90 |
| GTCTCTAACTCTGGTTTTAGG | 87628621 | 87628643 | 0.32 |
| GTTCATAACCATCTCTAACTC | 87628631 | 87628653 | 0.02 |
| GATGGTTATGAACCACCACGC | 87628643 | 87628665 | 0.02 |
| GAACCACCACGCAGGCCTT | 87628651 | 87628672 | 0.09 |
| GCCAAGTTCCTAAGGCCTGCG | 87628657 | 87628679 | 0.10 |
| GCCAGAAGATCCAAGTTCCTA | 87628666 | 87628688 | 0.01 |
| GAGGAAAGAAAGGGGGAGGC | 87628718 | 87628740 | 0.19 |
| GGGGTTTGAGGAAAGAAAGG | 87628725 | 87628747 | 0.05 |
| GACTCTTCAATCCGGGGTTTG | 87628737 | 87628759 | 0.10 |
| GTAATTTAACTCTTCAATCCG | 87628744 | 87628766 | 0.26 |
| GTTTTGAAACAGGATCTTACT | 87628806 | 87628828 | 0.00 |
| GTCTTACTAGGGAGCCCTGGC | 87628819 | 87628841 | 0.01 |
| GACCAGTTTCAGACCAGCCA | 87628832 | 87628854 | 0.02 |
| GACTTAGGAGGCAGAAACAGC | 87628854 | 87628876 | 0.52 |
| GTCTGCCTCCTAAGTGCAAAG | 87628863 | 87628885 | 0.02 |
| GCTTTAAGCCTCTTTGCACTT | 87628870 | 87628892 | 0.01 |
| GAAGGCTTGTTCTGCCACATC | 87628890 | 87628912 | 0.11 |
| GATTAATCGAAGCCAGATG | 87628903 | 87628924 | 0.23 |
| GATTCCTTTTTAATTTCTTCG | 87628925 | 87628947 | 0.01 |
| GTTTAATTTCTTCGAGGCCTT | 87628932 | 87628954 | 0.02 |
| GTCGAGGCCTTTGGTAACTCC | 87628942 | 87628964 | 0.00 |
| GCAGGACCTGGAGTTACCAA | 87628948 | 87628970 | 0.02 |
| GAAGTATGGTGGTGCAGGACC | 87628960 | 87628982 | 0.01 |
| GTTACGTAAGTATGGTGGTGC | 87628966 | 87628988 | 0.02 |
| GATCCTGAGTTACGTAAGTA | 87628975 | 87628997 | 0.01 |
| GCCTCTGGCTTGTGACGGTG | 87629031 | 87629053 | 3.83 |
| GGACAGCCTCTGGCTTGTGA | 87629036 | 87629058 | 0.05 |
| GGAGGAAAAGGACAGCCTC | 87629046 | 87629067 | 0.28 |
| GACAAAAGAGGAGGAGAAGA | 87629099 | 87629121 | 0.03 |
| GAAAGAGAAGACAAAAGAGG | 87629108 | 87629130 | 0.02 |
| GTTGATCTAGCCCTGTAGCCC | 87629144 | 87629166 | 0.47 |
| GCAGGATTGTCTTGAGTTCA | 87629171 | 87629193 | 0.70 |
| GCTAGAATCCTAACATTCAGG | 87629199 | 87629221 | 0.95 |
| GGTGTTACCGCTCCCAGCTA | 87629223 | 87629245 | 0.23 |
| GACGTTTTCCATAGCTGGGAG | 87629230 | 87629252 | 0.01 |
| GCAGAACGTTTTCCATAGCT | 87629235 | 87629257 | 1.88 |
| GTGAATGTAAAGAAATATTT | 87629267 | 87629289 | 0.00 |
| GTTGAGAAGTAATCAGGGAGG | 87629322 | 87629344 | 0.01 |
| GATTCTTTTGAGAAGTAATC | 87629329 | 87629351 | 0.01 |
| GTCTCAAAAGAATCCACTGAG | 87629339 | 87629361 | 0.22 |
| GAATCCACTGAGTGGGGGAT | 87629347 | 87629369 | 0.06 |
| GAGTGGGGGATGGGGGCCC | 87629356 | 87629377 | 0.01 |
| GGGATGGGGGCCCAGGCCTG | 87629362 | 87629384 | 0.02 |
| GTAGGGGTGGGACCACAGGCC | 87629373 | 87629395 | 0.01 |

| | | | |
|---|---|---|---|
| GCCTCCTAGGGGTGGGACCAC | 87629378 | 87629400 | 0.00 |
| GTCCCACCCCTAGGAGGCAG | 87629384 | 87629406 | 0.01 |
| GCTGCTGCCTCTGCCTCCTAG | 87629390 | 87629412 | 0.07 |
| GGACTGATTCAAACTCCATC | 87629412 | 87629434 | 0.02 |
| GGAACTCATCTGGTCCCAGA | 87629427 | 87629449 | 0.41 |
| GATGAGTTCCAGGTCAACCT | 87629440 | 87629462 | 0.01 |
| GCACTGTAGCCGAGGTTGACC | 87629448 | 87629470 | 5.59 |
| GAAGGTCTCACACTGTAGCCG | 87629457 | 87629479 | 0.00 |
| GTCTTTTGTTTTACAGAGTCA | 87629476 | 87629498 | 0.01 |
| GAGAAAAGTTTGAAGGAGGA | 87629514 | 87629536 | 0.01 |
| GCAGTTGTGAGAAAAGTTTGA | 87629521 | 87629543 | 0.02 |
| GTTCTCACAACTGATGGACTC | 87629532 | 87629554 | 0.33 |
| GACTCTGGTATCTTCACCTA | 87629547 | 87629569 | 0.65 |
| GTAGAACACAGTCAGGGCCTT | 87629563 | 87629585 | 0.02 |
| GTTGGGCCTTAGAACACAGTC | 87629571 | 87629593 | 0.02 |
| GGGAAAGCAGATGGAAGGTT | 87629589 | 87629611 | 0.09 |
| GTTTTGGGAAAGCAGATGGA | 87629594 | 87629616 | 0.01 |
| GAAGGTAGTTGAGGGAGGTTT | 87629610 | 87629632 | 1.25 |
| GAAGGGAAAGGTAGTTGAGGG | 87629616 | 87629638 | 0.01 |
| GGGCAGAAAAGGAAGGGAA | 87629629 | 87629650 | 0.06 |
| GCCAATAGGGCAGAAAAGGAA | 87629634 | 87629656 | 0.03 |
| GATGGACCAATAGGGCAGAAA | 87629639 | 87629661 | 0.01 |
| GCTGAATATCAATGGACCAAT | 87629649 | 87629671 | 0.02 |
| GCAGGACATCCTGAATATCAA | 87629658 | 87629680 | 0.34 |
| GCACTTGGGATCAAGTTTAGC | 87629677 | 87629699 | 0.01 |
| GCTGTGACTGGGCATGCACTT | 87629692 | 87629714 | 0.01 |
| GCCTAGGTGCAGCCTGTGAC | 87629705 | 87629727 | 0.01 |
| GTGGGTGGCACAGCTGCCT | 87629721 | 87629742 | 0.02 |
| GATCTGTCATTTACATGTGT | 87629738 | 87629760 | 0.06 |
| GTAAATGACAGATCTTCCT | 87629747 | 87629768 | 0.01 |
| GACAGATCTTCCTTGGAAGT | 87629753 | 87629775 | 0.00 |
| GCTGCAGGGTTCCCACTTCCA | 87629763 | 87629785 | 0.04 |
| GTGGGAACCCTGCAGCAAC | 87629771 | 87629792 | 0.00 |
| GTGGTAGTGCCGGTTGCTGC | 87629779 | 87629801 | 0.38 |
| GAGAAATGTAGTGGTAGTGC | 87629789 | 87629811 | 0.16 |
| GGGTAGCAGGAGAAATGTAG | 87629798 | 87629820 | 0.01 |
| GCTGGGGGTGGGGTGGGTAGC | 87629811 | 87629833 | 0.05 |
| GCTGAGCGGGGGCTGGGGGT | 87629823 | 87629845 | 0.01 |
| GAGTCGCTGAGCGGGGGCTG | 87629828 | 87629850 | 0.07 |
| GTACGGGAGTCGCTGAGCGG | 87629834 | 87629856 | 0.01 |
| GAAAAAAAAAATTTGTAGTAC | 87629850 | 87629872 | 0.00 |
| GATTTGTATTAAGCGGGGCGG | 87629998 | 87630020 | 0.01 |
| GAAGATTTGTATTAAGCGGGG | 87630001 | 87630023 | 4.46 |
| GTACAAATCTTTAAGGAATAA | 87630014 | 87630036 | 0.01 |
| GATGGAAATTTTTGAGGCTAA | 87630042 | 87630064 | 0.04 |
| GCCTCAAAAATTTCCATCAG | 87630048 | 87630070 | 3.49 |
| GAAATTTCCATCAGTGGGGAC | 87630055 | 87630077 | 0.01 |
| GTGTCACCAGTCCCCACTGA | 87630061 | 87630083 | 0.73 |
| GCAGCCTGTGCTCTGAATCAC | 87630087 | 87630109 | 0.03 |
| GAACCAGATTGAATCAACAA | 87630110 | 87630132 | 0.01 |
| GTTGTTGATTCAATCTGGTTC | 87630113 | 87630135 | 0.02 |
| GGGAATCCAATGCCTATTC | 87630134 | 87630155 | 0.14 |

| | | | |
|---|---|---|---|
| GTCCAATGCCTATTCTGGCCT | 87630139 | 87630161 | 0.01 |
| GTATCCCCCCAAGGCCAGAAT | 87630146 | 87630168 | 0.64 |
| GGGGGGATACTATGTTCATA | 87630160 | 87630182 | 0.02 |
| GGCACGTTTACATATAAGA | 87630181 | 87630202 | 0.01 |
| GAAATCATAAAAAGAAAGCTC | 87630230 | 87630252 | 0.03 |
| GATAAAAAGAAAGCTCTGGGC | 87630235 | 87630257 | 1.54 |
| GAAGCTCTGGGCCGGGTTTGG | 87630244 | 87630266 | 0.78 |
| GAAAAAAAAAGAAAGCTCTGC | 87630411 | 87630433 | 0.02 |
| GTCTGCAGGGTATGACTCTAG | 87630426 | 87630448 | 0.01 |
| GATGACTCTAGTGGTTTTAAC | 87630436 | 87630458 | 0.01 |
| GTTAAAACCTCTGCGTGTGC | 87630463 | 87630485 | 0.01 |
| GCTGTTCTCCGGCACACGCAG | 87630470 | 87630492 | 0.96 |
| GCCATACTCAAGACTGTTCTC | 87630482 | 87630504 | 0.02 |
| GCTTGAGTATGGTCTCTCTTC | 87630494 | 87630516 | 0.10 |
| GCATTTAAACAACAGCGAGAT | 87630522 | 87630544 | 0.02 |
| GTAGATAGAGTCTGGAAATTA | 87630546 | 87630568 | 0.00 |
| GTTTTTTTCTTTGTCTGAGAC | 87630631 | 87630653 | 0.01 |
| GCTGTGTAGCCCAGCATGGCC | 87630661 | 87630683 | 0.02 |
| GAGCGAGTTCCAGGCCATGC | 87630670 | 87630692 | 0.00 |
| GCTGTGAACTTGAAGCCAACC | 87630699 | 87630721 | 0.44 |
| GCAACTACTCTTGGGAGGTAG | 87630731 | 87630753 | 0.44 |
| GCAATCCCAACTACTCTTGGG | 87630737 | 87630759 | 0.00 |
| GTGTAATTAAGCTGTGTTAG | 87630773 | 87630795 | 1.49 |
| GATATATATATACCCAGGGCA | 87630840 | 87630862 | 0.02 |
| GTTGAATGCTTACCCTTGCCC | 87630852 | 87630874 | 0.01 |
| GTAAGCATTCAAAACTGCTCT | 87630864 | 87630886 | 0.02 |
| GACTGCTCTAGGTTCTGTGCC | 87630876 | 87630898 | 0.21 |
| GTGCCTGGCTTTTAAAAGAA | 87630891 | 87630913 | 0.03 |
| GAAAAGAAAGGGTGACTTGCT | 87630904 | 87630926 | 0.04 |
| GGGTGACTTGCTAGGGTCTG | 87630912 | 87630934 | 0.10 |
| GGGTCTGGGGTTCACCAGTT | 87630925 | 87630947 | 0.01 |
| GTTCACCAGTTAGGCCAGGT | 87630934 | 87630956 | 0.01 |
| GCCAGCCAACCTGGCCTAAC | 87630939 | 87630961 | 0.02 |
| GTTGGCTGGCCAGACAGTGC | 87630952 | 87630974 | 0.00 |
| GAAAGAACCCTGCACTGTC | 87630961 | 87630982 | 0.05 |
| GTCTAGCACTGAACAGCAGTA | 87630986 | 87631008 | 0.31 |
| GCATCTGCTTCTTCCACATC | 87631016 | 87631038 | 0.06 |
| GCTTCCACATCTGGCTTTTCA | 87631026 | 87631048 | 0.01 |
| GGCTTTTCATGGATGCAGGG | 87631037 | 87631059 | 0.10 |
| GGAATTAAAGCCCCCATGCA | 87631059 | 87631081 | 0.01 |
| GAAAAAAGTGCTTGCCATGCA | 87631072 | 87631094 | 0.00 |
| GAATGCATGAGACTGGAGAGA | 87631265 | 87631287 | 0.05 |
| GCATGGCAAAATGCATGAGAC | 87631273 | 87631295 | 0.29 |
| GCTGGAGAGATAGCTGAGCA | 87631291 | 87631313 | 0.01 |
| GAAAAATTATGTGAGGGAGGC | 87631309 | 87631331 | 8.47 |
| GAAAAAAAAAAAATTATGTGA | 87631316 | 87631338 | 0.02 |
| GATTATGTGAGCCGGGCGTGG | 87631478 | 87631500 | 0.01 |
| GTATTAAAAATTATGTGAGC | 87631487 | 87631509 | 0.03 |
| GCAACAGTGTGTTGAAGGGAG | 87631509 | 87631531 | 0.01 |
| GTTAAACAACAGTGTGTTGAA | 87631514 | 87631536 | 0.03 |
| GTTTAATTTCCCTTCCGTC | 87631531 | 87631552 | 0.11 |
| GCCAAGGGTAACCGGACGGAA | 87631540 | 87631562 | 0.01 |

| | | | |
|---|---|---|---|
| GAATGAGTCACCAAGGGTAAC | 87631549 | 87631571 | 0.01 |
| GCAGAGAAATGAGTCACCAA | 87631556 | 87631578 | 2.59 |
| GGTGACTCATTTCTCTGCGT | 87631561 | 87631583 | 0.00 |
| GCTGCGTGGGGAGTGTGTATA | 87631575 | 87631597 | 0.01 |
| GGGGAGTGTGTATATGGTGC | 87631581 | 87631603 | 1.12 |
| GGCCCTGGGTTTTGGGTTTT | 87631623 | 87631645 | 0.02 |
| GATGCACAAGGCCCTGGGTTT | 87631631 | 87631653 | 0.16 |
| GTAATGCATGCACAAGGCCCT | 87631637 | 87631659 | 0.02 |
| GATCTGGCTAATGCATGCACA | 87631644 | 87631666 | 0.01 |
| GGACTGGAGTGTAGTCCATC | 87631661 | 87631683 | 0.02 |
| GAAAAGAAATAATTAGGGAC | 87631677 | 87631699 | 0.02 |
| GAGAAGAAAAGAAATAATTA | 87631682 | 87631704 | 0.10 |
| GAATAGAAAAGAAGGAAAAGA | 87631722 | 87631744 | 0.01 |
| GTTTCCTTCTTTTCTATTCTG | 87631728 | 87631750 | 0.01 |
| GATTCTGAGGTCTCAAAACAC | 87631742 | 87631764 | 0.77 |
| GCTCAAAACACTGGTTAAGAG | 87631752 | 87631774 | 0.01 |
| GCGGGTCCGCCCGCTGACAT | 87631771 | 87631793 | 0.01 |
| GGCTTCACGCCCATGTCAGC | 87631780 | 87631802 | 0.01 |
| GACATGGGCGTGAAGCCTCTG | 87631787 | 87631809 | 0.79 |
| GAAGTATGGAGATGCCACAG | 87631801 | 87631823 | 0.01 |
| GGGTCGATGGGATGAAGTA | 87631815 | 87631836 | 0.03 |
| GTCCTCAGCAGTGGGTCGAT | 87631826 | 87631848 | 0.00 |
| GCAGACACGAGTCCTCAGCAG | 87631835 | 87631857 | 0.07 |
| GGACTCGTGTCTGAAACACT | 87631845 | 87631867 | 0.02 |
| GTTGATGGTTTTGAACAGCGC | 87631868 | 87631890 | 0.24 |
| GAAAACCATCAAGATCAGGAA | 87631880 | 87631902 | 0.01 |
| GCTATCCTTTCCTGATCTTGA | 87631884 | 87631906 | 0.00 |
| GGATAGAGAGAGACTGAGC | 87631901 | 87631922 | 0.01 |
| GAGACTGAGCTGGACTCCCA | 87631910 | 87631932 | 0.56 |
| GAGCTGGACTCCCATGGGTT | 87631916 | 87631938 | 0.01 |
| GGACTCCCATGGGTTGGGGA | 87631921 | 87631943 | 0.01 |
| GTTGCCACCATCCCCAACCCA | 87631927 | 87631949 | 1.19 |
| GTAATAACTAATATATAATAT | 87631952 | 87631974 | 0.14 |
| GCTTAGCATGTGCCAAGCTG | 87631985 | 87632007 | 0.01 |
| GCTGGATGTGAACCCACAGCT | 87631997 | 87632019 | 0.00 |
| GTCACATCCAGCCTCATCCTC | 87632010 | 87632032 | 0.01 |
| GCTACTGGTGGCCGGAGGATG | 87632020 | 87632042 | 0.12 |
| GCCCCCCTACTGGTGGCCGG | 87632026 | 87632048 | 0.01 |
| GCATGCCCCCCTACTGGTGGC | 87632029 | 87632051 | 0.02 |
| GAACCACCATGCCCCCCTAC | 87632036 | 87632058 | 0.13 |
| GAATAAACAACAAATAAAGGC | 87632058 | 87632080 | 0.06 |
| GTTATTTTCTTGTTTTGAGAC | 87632076 | 87632098 | 0.01 |
| GGGGTTCTTTGTTTAGCCC | 87632097 | 87632118 | 0.01 |
| GTTGTTTAGCCCAGGCTGTCC | 87632105 | 87632127 | 0.03 |
| GATTCGGAAAGCAGAGGCAGA | 87632170 | 87632192 | 0.03 |
| GACGCTCTTAGCCCCAGCATT | 87632187 | 87632209 | 0.02 |
| GAGCGTGCAGCACCACAGCA | 87632204 | 87632226 | 1.15 |
| GCAGCACCACAGCACGGTTG | 87632210 | 87632232 | 0.42 |
| GCATGAACCACAACCGTGCTG | 87632216 | 87632238 | 0.01 |
| GCTTTTAATGATAACAATT | 87632238 | 87632259 | 0.02 |
| GAACAATTAGGATGCTGAGGT | 87632250 | 87632272 | 0.44 |
| GATTAGGATGCTGAGGTAGGA | 87632254 | 87632276 | 0.01 |

| | | | |
|---|---|---|---|
| GCTGAGGTAGGAAGGTTACC | 87632262 | 87632284 | 0.01 |
| GACCAGGAGTTGAGTCCAATC | 87632279 | 87632301 | 0.01 |
| GTTGAGTCCAATCTGGTACA | 87632286 | 87632308 | 0.01 |
| GGGGTCTCCCTGTACCAGAT | 87632293 | 87632315 | 0.01 |
| GTTGGTTGGCTTTTGAGGAG | 87632312 | 87632334 | 0.12 |
| GTTTTGGTTGGTTGGCTTTTG | 87632317 | 87632339 | 0.17 |
| GCTAGGGTTTTTGGTTTTGGT | 87632330 | 87632352 | 0.02 |
| GTTTGGCTGACCTAGGGTTTT | 87632340 | 87632362 | 0.01 |
| GTAGAACACTTTGGCTGACCT | 87632348 | 87632370 | 0.01 |
| GACTACAGGCATAGAACACTT | 87632358 | 87632380 | 0.03 |
| GTTCTATGCCTGTAGTCTC | 87632365 | 87632386 | 0.01 |
| GCTTGCATAGCCAGAGACTAC | 87632373 | 87632395 | 0.04 |
| GTTTTGCCTCTGGCTGGCAAG | 87632399 | 87632421 | 0.00 |
| GTTGATTCTTTTGCCTCTGGC | 87632406 | 87632428 | 0.00 |
| GTTGTTTTTGAGACAAGTCTC | 87632481 | 87632503 | 0.04 |
| GAACAAGCGACAACAAAAAAC | 87632499 | 87632521 | 0.01 |
| GCAACAAAAAACTGGGCAATA | 87632508 | 87632530 | 0.02 |
| GGCAATATGGGATTCCAGAT | 87632521 | 87632543 | 0.25 |
| GATGGGATTCCAGATTGGTCT | 87632527 | 87632549 | 1.19 |
| GATTGGTCTCGGTGATACAA | 87632538 | 87632560 | 0.02 |
| GCTCGGTGATACAAGGGAAAC | 87632545 | 87632567 | 0.03 |
| GAAACTGGGGTCCCCTTTTG | 87632560 | 87632582 | 0.29 |
| GGGTCCCCTTTTGAGGTTCT | 87632567 | 87632589 | 0.01 |
| GAATCAAAGAATGTCCAAAAA | 87632597 | 87632619 | 0.03 |
| GTTCTGACTGAGTCCCTTTTT | 87632610 | 87632632 | 0.02 |
| GGGACTCAGTCAGAAGCTCA | 87632618 | 87632640 | 0.01 |
| GTCAGAAGCTCAAGGACTTAG | 87632627 | 87632649 | 0.01 |
| GAGGTGATAAGAAAACATCC | 87632646 | 87632668 | 0.02 |
| GATAAGAAAACATCCAGGGC | 87632651 | 87632673 | 1.80 |
| GATTTAAAGCTTACCTGCCC | 87632664 | 87632686 | 0.06 |
| GAAATCCCAGCAGCTTCGCAG | 87632682 | 87632704 | 0.01 |
| GCTTCGCAGAGGAGGAGAC | 87632693 | 87632714 | 0.15 |
| GTGCCCTTGAGTCAAGCCAG | 87632731 | 87632753 | 0.02 |
| GCCTGTAGCTGACCTCTCCAC | 87632747 | 87632769 | 0.01 |
| GAGGTCAGCTACAGGACAGC | 87632755 | 87632777 | 0.00 |
| GGACAGCAGGCAGCCACAAG | 87632768 | 87632790 | 0.01 |
| GGCAGCCACAAGGGGGGAAG | 87632776 | 87632798 | 0.03 |
| GGGCCTCAGAAGAACGAACA | 87632796 | 87632818 | 0.03 |
| GTCTGCTTCTTATGATTCTTG | 87632822 | 87632844 | 0.06 |
| GTAAGAAGCAGAGTTAGATTC | 87632834 | 87632856 | 0.02 |
| GCACCAGAAAGCACAACAGAA | 87632861 | 87632883 | 0.39 |
| GAGAAGCTTGCACTTTGAGC | 87632891 | 87632913 | 7.47 |
| GAGCAGGCAGCATCTCATCA | 87632907 | 87632929 | 0.00 |
| GCAGCATCTCATCATGGCTGC | 87632914 | 87632936 | 0.01 |
| GTCATGGCTGCTGGCCTAGTA | 87632924 | 87632946 | 0.01 |
| GCTGGCCTAGTAAGGACTGA | 87632932 | 87632954 | 0.01 |
| GACAATCCTTCAGTCCTTACT | 87632937 | 87632959 | 0.02 |
| GGACTGAAGGATTGTGGGA | 87632945 | 87632966 | 6.00 |
| GAAGGAAGAAAAGCTCATTAA | 87632963 | 87632985 | 0.11 |
| GTTCCACCTGTCTGCTTTGCA | 87632994 | 87633016 | 0.01 |
| GTTAAACCTTGCAAAGCAGAC | 87632999 | 87633021 | 0.01 |
| GTTAATGAACCCACCTTCCTA | 87633018 | 87633040 | 0.01 |

| | | | |
|---|---|---|---|
| GGGATCAGCCTTAGGAAGGT | 87633026 | 87633048 | 0.23 |
| GCCGGCCAAGGGATCAGCCTT | 87633034 | 87633056 | 0.01 |
| GCTGGGTCAGTCGCCCGGCCA | 87633047 | 87633069 | 0.11 |
| GCTGGGACTGGGTCAGTCGCC | 87633053 | 87633075 | 0.00 |
| GAGAATCAGGGGCCTGGGAC | 87633066 | 87633088 | 0.80 |
| GACAAAGAGAATCAGGGGCCT | 87633071 | 87633093 | 0.02 |
| GAATACCAACAAAGAGAATC | 87633079 | 87633101 | 9.71 |
| GCAAGTGTCTGTGTCTGTGAC | 87633107 | 87633129 | 1.08 |
| GCAGACACAGACACTTGAACT | 87633114 | 87633136 | 0.01 |
| GAGGACAGGTCAGAAGGAAC | 87633137 | 87633159 | 0.07 |
| GCCATCCGAGGACAGGTCAGA | 87633143 | 87633165 | 0.02 |
| GCTGTGGAGCCATCCGAGGAC | 87633151 | 87633173 | 0.01 |
| GTCGGATGGCTCCACAGATGC | 87633158 | 87633180 | 0.00 |
| GTCCACAGATGCTGGACAGCA | 87633167 | 87633189 | 0.01 |
| GTTGTCCAGTCTTAGGAGCGT | 87633196 | 87633218 | 0.05 |
| GATCCTCACACCCACTCCTCT | 87633221 | 87633243 | 0.01 |
| GTCTGGGGAACCTAGAGGAGT | 87633230 | 87633252 | 0.04 |
| GACGTCTCTGGGGAACCTAG | 87633236 | 87633258 | 0.02 |
| GACGTTGGGGCGACGTCTCT | 87633247 | 87633269 | 0.12 |
| GCTGCTTCATGCTGACGTTG | 87633260 | 87633282 | 0.51 |
| GTAGGCTCATCATGGTATC | 87633282 | 87633303 | 0.00 |
| GAAAGAGCAGTAGGCTCATCA | 87633289 | 87633311 | 0.01 |
| GGTGATGGCAAAAGAGCAGT | 87633299 | 87633321 | 2.54 |
| GAAAAAATTAGAAAAGGTGA | 87633314 | 87633336 | 0.01 |
| GAAAAAAGAAAAAATTAGAAA | 87633320 | 87633342 | 0.03 |
| GTTTTTTAAACTAAACCAAAA | 87633341 | 87633363 | 0.02 |
| GTAAGAGAGGAGGGAAGAGG | 87633453 | 87633475 | 0.07 |
| GAGAAAAGCAGGAGTAAGAG | 87633466 | 87633488 | 0.01 |
| GGGGATGGGGGAAGAGGCAA | 87633488 | 87633510 | 0.02 |
| GATGTAGAGAGAGGGGGGAAG | 87633507 | 87633529 | 0.02 |
| GCCATGACCATGTAGAGAGA | 87633516 | 87633538 | 0.04 |
| GGAGAGGAGAGGTAGAGGC | 87633538 | 87633559 | 0.04 |
| GGTGAGAGGAGAGAGGAGAG | 87633699 | 87633721 | 0.03 |
| GGAGAGGGGGGAGAGGTGAG | 87633713 | 87633735 | 0.07 |
| GGCGTGGGAGAGGGTAGTAG | 87633999 | 87634021 | 0.01 |
| GATTATTCAGGGCGTGGGAGA | 87634008 | 87634030 | 0.01 |
| GAGTTTATTATTCAGGGCGT | 87634014 | 87634036 | 2.88 |
| GTAGAATAGAGTTTATTATTC | 87634021 | 87634043 | 0.02 |
| GTACTATACTATACTGTTGTG | 87634069 | 87634091 | 0.01 |
| GCAAAGCACCCCTTCCCCTGA | 87634097 | 87634119 | 0.11 |
| GGGGGTGTGGTGAGGTGTCG | 87634146 | 87634168 | 0.02 |
| GACCAGAAGTTTCCCTAACT | 87634219 | 87634241 | 1.08 |
| GAACAACAAAGGCCTAGTTA | 87634231 | 87634253 | 0.02 |
| GCTGTGTTAGAGGAACAACAA | 87634242 | 87634264 | 0.04 |
| GTTCCTCTAACACAGGGATA | 87634250 | 87634272 | 0.10 |
| GTTTAAAAAGAAACAAGAGC | 87634283 | 87634305 | 0.00 |
| GAAACAAGAGCCGGGCATGG | 87634292 | 87634314 | 0.02 |
| GAATAAAAAGAAACAAGAAGC | 87634465 | 87634487 | 0.01 |
| GACTTTAGTCTAGTAACAC | 87634502 | 87634523 | 0.01 |
| GTAGTCTAGTAACACTGGCCA | 87634507 | 87634529 | 0.01 |
| GCTTTGTCAAGACATCGGCCT | 87634524 | 87634546 | 0.01 |
| GCCATGTCTTTGTCAAGACAT | 87634530 | 87634552 | 0.01 |

| | | | |
|---|---|---|---|
| GACAAAGACATGGTTTGTA | 87634540 | 87634561 | 4.19 |
| GAATAACTCCATAATCACAT | 87634574 | 87634596 | 0.80 |
| GATAATCACATGGGGACCTTC | 87634584 | 87634606 | 0.05 |
| GGGACCTTCAGGTCTATCCT | 87634595 | 87634617 | 0.01 |
| GTCTATCCTTGGATCTCTGC | 87634606 | 87634628 | 0.01 |
| GATGTAACCAGCAGAGATCCA | 87634612 | 87634634 | 0.02 |
| GGCATGGGGTGAGGAGGAG | 87634644 | 87634665 | 0.01 |
| GAAGATGGCATGGGGTGAGG | 87634649 | 87634671 | 0.03 |
| GCCAGCTGAGAAGATGGCATG | 87634657 | 87634679 | 0.02 |
| GTATTTCCCAGCTGAGAAGA | 87634664 | 87634686 | 0.01 |
| GGGAAATACTTTGATACACA | 87634678 | 87634700 | 0.03 |
| GATACACAAGGCATCGTGCA | 87634690 | 87634712 | 0.10 |
| GGCATCGTGCAAGGCATTAT | 87634699 | 87634721 | 0.02 |
| GCCTCTCAGGGTCTGGGATAT | 87634729 | 87634751 | 0.26 |
| GATCCCAGACCCTGAGAGGAA | 87634734 | 87634756 | 0.93 |
| GACCCTGAGAGGAAAGGGGG | 87634740 | 87634762 | 0.01 |
| GGGAGGGCTGCATAAGACAC | 87634757 | 87634779 | 0.01 |
| GCATAAGACACTGGAGAGA | 87634766 | 87634787 | 5.72 |
| GATGGCTCAGTGACTAAGCCC | 87634784 | 87634806 | 0.06 |
| GCTGGTCTTCTAAGAGCAGCC | 87634802 | 87634824 | 0.26 |
| GAGTGTCAGAAACTGAACCC | 87634821 | 87634843 | 0.01 |
| GTTCAGTTTCTGACACTCACA | 87634827 | 87634849 | 0.01 |
| GCCTGCAGCTCCCGTTTCAG | 87634861 | 87634883 | 0.58 |
| GAGCATTGATCCTCTGAAAC | 87634871 | 87634893 | 0.89 |
| GCAGAGGATCAATGCTCTCTT | 87634878 | 87634900 | 2.00 |
| GAATGCTCTCTTTGGCCTGTA | 87634887 | 87634909 | 0.00 |
| GCATGCATGCAGTTCCCATAC | 87634901 | 87634923 | 0.06 |
| GTGATGCATAGACATACATG | 87634923 | 87634945 | 0.12 |
| GAAGATACCGTCTTTAAAAAA | 87634991 | 87635013 | 0.02 |
| GGCTTGCCTTTTTTTAAAGA | 87634997 | 87635019 | 0.15 |
| GGAAAGGATCAAATGGGTGA | 87635018 | 87635040 | 0.02 |
| GATGCATGGGAAAGGATCAAA | 87635025 | 87635047 | 14.23 |
| GAAGTCACTGATGCATGGGAA | 87635034 | 87635056 | 0.01 |
| GCTGTTAAGTCACTGATGCAT | 87635039 | 87635061 | 0.07 |
| GAACACAAAATTCTTCAAGGA | 87635069 | 87635091 | 0.01 |
| GCTTCAAGGAAGGAGAAGCCA | 87635080 | 87635102 | 0.05 |
| GGAGAAGCCATGGTGGTCAT | 87635090 | 87635112 | 0.01 |
| GACTCCACCAATGACCACCA | 87635097 | 87635119 | 0.03 |
| GGTCTCCAGAGAGCCCAAGT | 87635122 | 87635144 | 0.02 |
| GCTGCAGTTAGGGTCCAACTT | 87635135 | 87635157 | 0.00 |
| GCTCGGCTAGCTCTGCAGTTA | 87635146 | 87635168 | 0.57 |
| GACTGCAGAGCTAGCCGAGGA | 87635151 | 87635173 | 0.01 |
| GCAGAAGTTGAAGACCATCCT | 87635164 | 87635186 | 0.98 |
| GCTTTCACCTTCCCAGTGC | 87635194 | 87635215 | 0.15 |
| GCCGCAATGCCAGCACTGGGA | 87635201 | 87635223 | 0.01 |
| GAGGCCCGCAATGCCAGCAC | 87635206 | 87635228 | 0.20 |
| GTAAACTGGGCTTGGTGGCAG | 87635225 | 87635247 | 0.09 |
| GATCTCCTAAACTGGGCTTGG | 87635231 | 87635253 | 0.02 |
| GAAGCCCAGTTTAGGAGATGC | 87635236 | 87635258 | 0.01 |
| GTTGAGCACACTTGAAAACC | 87635268 | 87635290 | 0.68 |
| GGGGATGTAGATCAGTTAGA | 87635297 | 87635319 | 0.10 |
| GTAACTGATCTACATCCCCAG | 87635302 | 87635324 | 0.97 |

| | | | |
|---|---|---|---|
| GATCTACATCCCCAGAGGGC | 87635307 | 87635329 | 1.50 |
| GCACACATTTCCAGCCCTCT | 87635317 | 87635339 | 0.00 |
| GTGAGTTGACAGAGATATTG | 87635355 | 87635377 | 0.00 |
| GACAGAGATATTGGGGCAGG | 87635362 | 87635384 | 0.42 |
| GGGGCAGGAGGGGAGTTAG | 87635374 | 87635395 | 0.01 |
| GATATTCTTTATAGCATCTCA | 87635403 | 87635425 | 0.01 |
| GATAGAGCACAGATAGAATTT | 87635431 | 87635453 | 0.01 |
| GTAGAATTGGGCGGGGGGGG | 87635488 | 87635510 | 0.06 |
| GCCCCACTAGAATTGGGCGGG | 87635494 | 87635516 | 0.06 |
| GATGACACTCCCCACTAGAAT | 87635502 | 87635524 | 0.02 |
| GTCATGCTATTTCATGAAGA | 87635520 | 87635542 | 0.01 |
| GTTCATGAAGATGGAGGTCAG | 87635530 | 87635552 | 0.01 |
| GAGGTCAGAGGATTAACTAG | 87635542 | 87635564 | 0.13 |
| GAGGATTAACTAGTGGGGGT | 87635549 | 87635571 | 0.01 |
| GCCTAGAACCCACAGGGTAGA | 87635578 | 87635600 | 0.01 |
| GGGTTCTAGGGACTGAATGC | 87635591 | 87635613 | 0.03 |
| GGGACTGAATGCTGGACATC | 87635599 | 87635621 | 0.01 |
| GAATGCTGGACATCAGGAAGT | 87635606 | 87635628 | 0.01 |
| GCATCAGGAAGTAGGGGTCAG | 87635615 | 87635637 | 1.49 |
| GGGGTCAGAGGATCCCTAAC | 87635627 | 87635649 | 0.02 |
| GCTCTAAATGTCTGCCTGTTA | 87635640 | 87635662 | 0.00 |
| GACAGGCAGACATTTAGAGAT | 87635645 | 87635667 | 0.01 |
| GAGATAGGGCCACAAAAAT | 87635660 | 87635681 | 0.01 |
| GATCTTCATCTCCTATTTTTG | 87635669 | 87635691 | 0.00 |
| GCAGCAGGGTGAGGGAGAAGC | 87635701 | 87635723 | 0.02 |
| GTCTCAGATCAGCAGGGTGA | 87635710 | 87635732 | 0.01 |
| GCTTTTGTCTCAGATCAGCA | 87635716 | 87635738 | 0.01 |
| GATCTGAGACAAAAGCCTGG | 87635723 | 87635745 | 0.01 |
| GGCCTTCTATTTAAGCCTCC | 87635738 | 87635760 | 0.31 |
| GGCGGGAGAAAAAGCAGGCC | 87635759 | 87635781 | 0.02 |
| GCTACTGGCGGGAGAAAAAGC | 87635764 | 87635786 | 0.01 |
| GCAGCAGGTGGGCCCTACTGG | 87635777 | 87635799 | 0.49 |
| GGGCCCACCTGCTGGAGGAC | 87635786 | 87635808 | 8.82 |
| GCAGTCATGAACTTGTCAGAC | 87635805 | 87635827 | 0.31 |
| GACAAGTTCATGACTGAATG | 87635812 | 87635834 | 0.01 |
| GATGAGGACTGTTTATCCCAC | 87635829 | 87635851 | 0.02 |
| GACTGTTTATCCCACCGGTAT | 87635835 | 87635857 | 0.00 |
| GCCGATAGGCCGATACCGGT | 87635844 | 87635866 | 0.01 |
| GACTAAGACAGCGCAGCCGAT | 87635858 | 87635880 | 0.02 |
| GTCAAACTGACCAACCCAAAC | 87635881 | 87635903 | 1.82 |
| GTTCAGCCCCCAGTTTGGGT | 87635890 | 87635912 | 0.00 |
| GGAGGGGTGGGAGTGGGGGG | 87635920 | 87635942 | 0.02 |
| GTCTTTGGAGGGGTGGGAGT | 87635926 | 87635948 | 0.02 |
| GGTTTTATGGGTCTTTGGAG | 87635936 | 87635958 | 0.03 |
| GTCCAAAGACCCATAAAACCC | 87635940 | 87635962 | 0.01 |
| GCCTCCACACCTGGGTTTTAT | 87635948 | 87635970 | 0.01 |
| GCTGCTTCTCCTCCACACCT | 87635957 | 87635979 | 0.41 |
| GCAGCACTTCTTCCCTCTGA | 87635975 | 87635997 | 0.01 |
| GTGCAGAATCAGTCCATCAG | 87635988 | 87636010 | 0.00 |
| GATTCTGCACGTGAGACCTG | 87636001 | 87636023 | 1.20 |
| GCACGTGAGACCTGCGGCGG | 87636007 | 87636029 | 0.00 |
| GAGACCTGCGGCGGCGGCGG | 87636013 | 87636035 | 0.01 |

| | | | |
|---|---|---|---|
| GCAGCTGCATGTTTCACTAG | 87636050 | 87636072 | 0.07 |
| GACAAGAGTTTCTCTTGTGT | 87636118 | 87636140 | 0.05 |
| GAGTTTCTCTTGTGTGGGCC | 87636123 | 87636145 | 0.01 |
| GCTGTGAGTTCGAATACAGCC | 87636141 | 87636163 | 0.01 |
| GACGACGAAAGCAGAGGCAGG | 87636168 | 87636190 | 0.55 |
| GCCTCTGCTTTCGTCGTGCT | 87636174 | 87636196 | 0.01 |
| GTTTCGTCGTGCTAGGATTGA | 87636182 | 87636204 | 0.01 |
| GAAGGTGTGTGCTACCCGCCC | 87636201 | 87636223 | 0.01 |
| GCTCGAAATGGAACCAGGGC | 87636214 | 87636236 | 0.01 |
| GCAACTGGTGAGTGCTCGAAA | 87636226 | 87636248 | 0.15 |
| GATGGATGGCTAAGAGCCAAC | 87636242 | 87636264 | 0.06 |
| GTGCAGGAGTTATGTATGGA | 87636257 | 87636279 | 0.34 |
| GGCTTTGGGTCCCCTCGTGC | 87636273 | 87636295 | 0.25 |
| GATTAGTCAGAAGAGGGCTTT | 87636287 | 87636309 | 1.44 |
| GCCCTCTTCTGACTAATAAG | 87636293 | 87636315 | 0.01 |
| GTTCTGACTAATAAGAGGACC | 87636299 | 87636321 | 0.02 |
| GAATAAGAGGACCAGGCATGC | 87636307 | 87636329 | 0.01 |
| GTTGTGCACCACCTGCATGCC | 87636317 | 87636339 | 0.00 |
| GCAGGTGGTGCACAAGCATGC | 87636326 | 87636348 | 0.01 |
| GTAATTGTAAAAGTGTGAAA | 87636371 | 87636393 | 0.01 |
| GTAAAAGTGTGAAAAGGTAT | 87636377 | 87636399 | 0.00 |
| GTATTGGAAAGTAAGCTGCC | 87636393 | 87636415 | 0.02 |
| GACTTGACTTTGCGCTGCGCC | 87636411 | 87636433 | 0.13 |
| GTTGTCTATTGTCTTTCGTCC | 87636435 | 87636457 | 0.45 |
| GTCTTTCGTCCAGGAAGTTCT | 87636445 | 87636467 | 0.01 |
| GAAGTCAAACCGAGAACTTCC | 87636453 | 87636475 | 0.04 |
| GTTTGACTTGGTATCCCTTT | 87636467 | 87636489 | 0.02 |
| GACTGGACGTGAAAGCCGAA | 87636482 | 87636504 | 1.20 |
| GCGGACACACGGACGAGAC | 87636499 | 87636520 | 0.02 |
| GTCCGTGTGTCCGCGACATC | 87636507 | 87636529 | 0.01 |
| GTCCGCGACATCCGGTCCTTC | 87636516 | 87636538 | 0.00 |
| GATCCGGTCCTTCCGGGGTAC | 87636524 | 87636546 | 0.25 |
| GGGGATTCCAGTACCCCGGA | 87636531 | 87636553 | 0.01 |
| GGTACTGGAATCCCCGCCTC | 87636539 | 87636561 | 0.02 |
| GGAATCCCCGCCTCTGGTCC | 87636545 | 87636567 | 0.03 |
| GCTGTCCCGGACCAGAGGCG | 87636550 | 87636572 | 0.01 |
| GGTCCGGGACAGCGCACACC | 87636560 | 87636582 | 0.05 |
| GGGACAGCGCACACCTGGAG | 87636565 | 87636587 | 0.00 |
| GCACACCTGGAGAGGGGGCG | 87636573 | 87636595 | 0.01 |
| GGAGAGGGGGCGAGGTGGGG | 87636581 | 87636603 | 0.01 |
| GTGGGGCGGGTGAGTCACCG | 87636595 | 87636617 | 0.01 |
| GGTGAGTCACCGCGGGCGAG | 87636603 | 87636625 | 0.01 |
| GTCACCGCGGGCGAGCGGGC | 87636608 | 87636630 | 0.01 |
| GGGCGAGCGGGCGGGTGGGC | 87636616 | 87636638 | 0.01 |
| GCGGGTGGGCGGGCGAGCGG | 87636626 | 87636648 | 0.56 |
| GGGCGGGCGAGCGGAGGTGA | 87636632 | 87636654 | 0.02 |
| GAGCGGAGGTGAGGGCGGGG | 87636640 | 87636662 | 0.05 |
| GGTGAGGGCGGGGAGGGGCG | 87636647 | 87636669 | 0.01 |
| GGAGGGGCGTGGCCGGCCGC | 87636658 | 87636680 | 0.01 |
| GGATCTGCTGCCCCGGCGGC | 87636670 | 87636692 | 3.56 |
| GACCTGCGGATCTGCTGCCC | 87636677 | 87636699 | 0.00 |
| GCGAGGCGGGAGCGGACCTG | 87636691 | 87636713 | 0.03 |

| | | | |
|---|---|---|---|
| GCGCGCGGCGAGGCGGGAG | 87636699 | 87636720 | 2.71 |
| GCGCTGTGCGCGCGGCGAGG | 87636705 | 87636727 | 0.01 |
| GGACTGAGCGCTGTGCGCG | 87636713 | 87636734 | 0.01 |
| GTCGCGCTGCGCGGCGGCGGA | 87636733 | 87636755 | 0.38 |
| GGAGGACAGTCGCGCTGCG | 87636743 | 87636764 | 0.24 |
| GCGGCGCGGGACGGCTCGG | 87636760 | 87636781 | 0.01 |
| GACTCGCGGAGGTGCGGCGC | 87636772 | 87636794 | 0.00 |
| GAGGGCGACTCGCGGAGGTG | 87636778 | 87636800 | 0.00 |
| GCGGCGAGGGCGACTCGCGG | 87636783 | 87636805 | 0.01 |
| GATGCTGCGCGCGGAGCGGCG | 87636797 | 87636819 | 0.01 |
| GCCGCCATGCTGCGCGCGGAG | 87636802 | 87636824 | 0.01 |
| GGGACCCGCCATGCTGCGCG | 87636807 | 87636829 | 0.26 |
| GGTCCCCAGGCCCTCGCGTT | 87636824 | 87636846 | 1.47 |
| GAGCAGGAGCCCGAACGCGA | 87636834 | 87636856 | 0.11 |
| GTTCGGGCTCCTGCTCGCGG | 87636841 | 87636863 | 0.07 |
| GTCGCTGTGACCACCGCGAGC | 87636850 | 87636872 | 0.60 |
| GCGGTGGTCACAGCGACGC | 87636857 | 87636878 | 0.01 |
| GGTCACAGCGACGCTGGCCG | 87636862 | 87636884 | 4.87 |
| GACGCTGGCCGCGGCTCAGAG | 87636872 | 87636894 | 0.00 |
| GGCCGCGGCTCAGAGAGGTG | 87636877 | 87636899 | 0.25 |
| GGCTCAGAGAGGTGAGGCG | 87636883 | 87636904 | 0.12 |
| GAGAGGTGAGGCGTGGACCT | 87636889 | 87636911 | 0.03 |
| GAGGCGTGGACCTGGGGCGG | 87636896 | 87636918 | 1.08 |
| GTGGACCTGGGGCGGAGGTG | 87636901 | 87636923 | 0.02 |
| GTGGTGGGGTCGTGTCTGGC | 87636920 | 87636942 | 1.05 |
| GGGTCGTGTCTGGCCGGTGA | 87636926 | 87636948 | 0.30 |
| GTCTGGCCGGTGATGGGGAG | 87636933 | 87636955 | 0.00 |
| GGTGATGGGGAGCGGACAGC | 87636941 | 87636963 | 0.17 |
| GACAGCGGGGCTGCCGTCTC | 87636955 | 87636977 | 0.00 |
| GTCGGGTCTCGGGGCCAGAGA | 87636968 | 87636990 | 0.02 |
| GGCCCCGAGACCCGACGGTG | 87636976 | 87636998 | 4.20 |
| GACCCGACGGTGCGGGCGGG | 87636984 | 87637006 | 0.04 |
| GGGCGGGCGGGACGAGCGCT | 87636997 | 87637019 | 0.00 |
| GCGGGACGAGCGCTCGGATG | 87637003 | 87637025 | 1.15 |
| GAGCGCTCGGATGAGGCGG | 87637010 | 87637031 | 0.04 |
| GGCGGTGTGCTGTGCCCCGC | 87637027 | 87637049 | 1.69 |
| GTGCTGTGCCCCGCGGGGAC | 87637033 | 87637055 | 0.00 |
| GCCCCGCGGGGACGGGCGGC | 87637040 | 87637062 | 0.03 |
| GCGGGGACGGGCGGCCGGGA | 87637045 | 87637067 | 0.02 |
| GGGCGGCCGGGATGGCTGAG | 87637053 | 87637075 | 0.19 |
| GGTGGCCGCTCAGCCATCC | 87637059 | 87637080 | 0.01 |
| GGCTGAGCGGCCACCGCCCC | 87637066 | 87637088 | 0.01 |
| GCGGCCACCGCCCCAGGTC | 87637072 | 87637093 | 0.12 |
| GAAGTTGGAGTCGCCAGACCT | 87637083 | 87637105 | 0.01 |
| GGTCTGGCGACTCCAACTTG | 87637087 | 87637109 | 0.17 |
| GACTTGGGGTTCCAAGTTCCA | 87637102 | 87637124 | 0.22 |
| GGTTCCAAGTTCCAAGGCGC | 87637108 | 87637130 | 0.33 |
| GCAACGAAGGCCAGCGCCT | 87637119 | 87637140 | 0.41 |
| GCTGGTAGGTCGCAGCAACGA | 87637131 | 87637153 | 0.53 |
| GGGAGGGAGGGCTGACTGGT | 87637146 | 87637168 | 0.02 |
| GGCAGGTACGGAGGGAGGGA | 87637158 | 87637180 | 0.03 |
| GGGACCGGGGCAGGTACGGA | 87637166 | 87637188 | 0.12 |

| | | | |
|---|---|---|---|
| GAGCCCACATCCGGGGACCG | 87637179 | 87637201 | 0.02 |
| GGTCCCCGGATGTGGGCTCC | 87637183 | 87637205 | 0.06 |
| GTGGGCTCCGGGAGGGGAC | 87637194 | 87637215 | 4.96 |
| GGGAGCTACCTGTCCCCTCC | 87637201 | 87637223 | 0.07 |
| GGGGACAGGTAGCTCCCCCG | 87637207 | 87637229 | 0.03 |
| GCAGGTGCATAGCGGCCTCG | 87637222 | 87637244 | 0.00 |
| GCCGCTATGCACCTGCGCGT | 87637229 | 87637251 | 0.65 |
| GATGCACCTGCGCGTCGGAGG | 87637235 | 87637257 | 0.94 |
| GCGTCGGAGGCGGGCCCGCT | 87637245 | 87637267 | 0.42 |
| GCGGGCCCGCTGGGACTTAC | 87637254 | 87637276 | 0.02 |
| GGCGGCCAGTAAGTCCCAGC | 87637259 | 87637281 | 0.00 |
| GGGACTTACTGGCCGCCGG | 87637265 | 87637286 | 0.30 |
| GAAAAGGAAAAACACACCTC | 87637280 | 87637302 | 0.01 |
| GTTAGTCATGATTCTTAGAAA | 87637296 | 87637318 | 0.19 |
| GATGAATGCAGTGTTTATTGT | 87637318 | 87637340 | 0.04 |
| GATTATGCGATTTTTAGAA | 87637371 | 87637392 | 0.01 |
| GCACGATTTTTAATTAGAG | 87637396 | 87637417 | 0.01 |
| GAATCGTGCATATTTGTGTTT | 87637410 | 87637432 | 0.01 |
| GATATTTGTGTTTCGGGATAC | 87637418 | 87637440 | 0.84 |
| GATCTTACCAACATTTCATGA | 87637456 | 87637478 | 0.33 |
| GAAACTTCCTTCATGAAATGT | 87637462 | 87637484 | 0.23 |
| GAAGGAAGTTTTCTACTTT | 87637474 | 87637495 | 0.04 |
| GTAGGCTTTTATTTTTAAAAC | 87637492 | 87637514 | 2.13 |
| GAAACTGGGTTTGCATGAAGA | 87637508 | 87637530 | 0.01 |
| GCATGAAGAAGGACGATTTG | 87637519 | 87637541 | 0.18 |
| GTCACTGTTAAAGAATAGTT | 87637546 | 87637568 | 0.02 |
| GTTGAGTGATCTGTAGAGAGA | 87637569 | 87637591 | 0.14 |
| GATCTGTAGAGAGAAGGATG | 87637575 | 87637597 | 0.02 |
| GCTTAGAAATAAATCAAAAAT | 87637638 | 87637660 | 0.03 |
| GTTTATTTCTAAGATGGTGTC | 87637649 | 87637671 | 0.00 |
| GTCTAAGATGGTGTCTGGAAG | 87637655 | 87637677 | 0.94 |
| GAAGTGGAGACTTGAACAAAG | 87637672 | 87637694 | 0.00 |
| GAGACTTGAACAAAGCGGTC | 87637677 | 87637699 | 0.01 |
| GAAATTGAGAAAGAAACAAAA | 87637713 | 87637735 | 0.99 |
| GTATAATTTAGTTCTGTTTTC | 87637747 | 87637769 | 0.02 |
| GTTTAGTTCTGTTTTCTGGGA | 87637752 | 87637774 | 0.02 |
| GGAAGGATTTCATTTCCAAC | 87637769 | 87637791 | 0.00 |
| GTCATTTCCAACTGGGAGACG | 87637778 | 87637800 | 0.00 |
| GTATAGGCCACGTCTCCCAGT | 87637784 | 87637806 | 0.00 |
| GAAAATGTAATTAATGGATAT | 87637801 | 87637823 | 0.36 |
| GCTGTATTAAAATGTAATTAA | 87637808 | 87637830 | 0.03 |
| GCTATAGTTTTCTGATCTCT | 87637840 | 87637862 | 0.03 |
| GTTGGGCTAAATTGTAACTTG | 87637859 | 87637881 | 0.01 |
| GCTAAATTGTAACTTGAGGGC | 87637864 | 87637886 | 0.02 |
| GGATGTAGAGTCCCTGACCT | 87637886 | 87637908 | 0.01 |
| GTGCTAGGGAACCAAGGTCA | 87637897 | 87637919 | 0.01 |
| GTATGGGGTGCTAGGGAACCA | 87637903 | 87637925 | 2.94 |
| GCCTTGTTTATGGGGTGCTA | 87637911 | 87637933 | 3.01 |
| GTTACAGGTGCCTTGTTTAT | 87637920 | 87637942 | 0.96 |
| GCACCTGTAACCCCAGACCT | 87637932 | 87637954 | 0.10 |
| GTTCCACTTCCCAAGGTCTG | 87637942 | 87637964 | 0.01 |
| GCCTCCAGGTTCCACTTCCCA | 87637949 | 87637971 | 0.01 |

| | | | |
|---|---|---|---|
| GCCTTGAATTGCTAATCCTCC | 87637964 | 87637986 | 0.06 |
| GAATTCAAGGTCATCTTGAAT | 87637978 | 87638000 | 0.01 |
| GATCTTGAATTGGCGTAGCAC | 87637989 | 87638011 | 0.01 |
| GTAGCACCGGAGGCCAGCCT | 87638002 | 87638024 | 0.03 |
| GCTTTAGCCCAGGCTGGCCTC | 87638008 | 87638030 | 0.01 |
| GACACAGGGCTCCTTTAGCCC | 87638019 | 87638041 | 0.02 |
| GAAGCTTTTCTTCATTACACA | 87638034 | 87638056 | 0.01 |
| GTAATGAAGAAAAGCTTGAAG | 87638041 | 87638063 | 0.43 |
| GAAGCTTGAAGAGGAACCTAC | 87638051 | 87638073 | 0.03 |
| GAACCTACTGGGCCCCAAGT | 87638063 | 87638085 | 0.02 |
| GCCCCAAGTAGGCATTCCTT | 87638074 | 87638096 | 1.46 |
| GCCTGGACAAGTCACTTCCTA | 87638090 | 87638112 | 1.83 |
| GCTGCCATCTATGGCTCCTCC | 87638108 | 87638130 | 0.01 |
| GGAGCCATAGATGGCAGATC | 87638114 | 87638136 | 0.02 |
| GATCTGGTGTTGTTGACTTC | 87638130 | 87638152 | 0.19 |
| GACAGATAAGTGTGTAGTCGT | 87638173 | 87638195 | 0.30 |
| GTGTAGTCGTAGGTAAAGAG | 87638183 | 87638205 | 0.11 |
| GTAGGTAAAGAGAGGGACAC | 87638191 | 87638213 | 20.33 |
| GAGAGGGACACTGGAAAGTG | 87638200 | 87638222 | 0.01 |
| GGAAAGTGAGGGGAACAGAC | 87638212 | 87638234 | 0.85 |
| GTGAGGGGAACAGACAGGTG | 87638217 | 87638239 | 0.03 |
| GGTGCTGGGCTTACAGACTG | 87638243 | 87638265 | 0.04 |
| GCTTCTCAAGTGCTGGTGCT | 87638257 | 87638279 | 0.01 |
| GCACCAGCACTTGAGAAGCTG | 87638262 | 87638284 | 0.01 |
| GAGAAGCTGAGGCAGCCGGA | 87638273 | 87638295 | 0.01 |
| GCCAAGCTCACTACAGCCCTC | 87638288 | 87638310 | 0.04 |
| GAATCCAGAAATATCCAGAAA | 87638318 | 87638340 | 0.28 |
| GAAATATCCAGAAAAGGCGGG | 87638325 | 87638347 | 0.00 |
| GAAAAGGCGGGAGGGAGGGA | 87638334 | 87638356 | 0.01 |
| GCGGGAGGGAGGGAGGGACT | 87638340 | 87638362 | 0.01 |
| GGAGGGAGGGACTCGGAAGA | 87638347 | 87638369 | 0.01 |
| GAGGGACTCGGAAGAAGGAA | 87638352 | 87638374 | 0.02 |
| GTTTAATGACATGACCATTTC | 87638408 | 87638430 | 0.02 |
| GCATGACCATTTCTGGCTCAG | 87638416 | 87638438 | 0.00 |
| GTTTACCCACTGAGCCAGAAA | 87638421 | 87638443 | 0.01 |
| GCCTTGGGACTCTAACTCAG | 87638448 | 87638470 | 0.01 |
| GTTAGAGTCCCAAGGCCCAC | 87638455 | 87638477 | 0.02 |
| GTCCCAAGGCCCACAGGCAGA | 87638462 | 87638484 | 0.01 |
| GCCATTCCCCCTTCTGCCTGT | 87638470 | 87638492 | 1.66 |
| GGGGGAATGGACTCCCACGT | 87638483 | 87638505 | 0.02 |
| GTGGAAGTCAGAGGCCAACG | 87638497 | 87638519 | 0.03 |
| GTTCAAATGTGGAAGTCAG | 87638506 | 87638527 | 0.02 |
| GACTTCCACATTTGAACTG | 87638511 | 87638532 | 0.44 |
| GCACATGCCACAGTTCAAATG | 87638516 | 87638538 | 0.02 |
| GCGCAGAGTAAATGAGTGA | 87638579 | 87638600 | 0.05 |
| GTGAAGGTTTAACTTCAGAA | 87638594 | 87638616 | 0.01 |
| GCCCAGTAGTGCAGCCGCAA | 87638620 | 87638642 | 0.01 |
| GCAGCCGCAAGGGTTTATTTC | 87638631 | 87638653 | 0.20 |
| GCAAGGGTTTATTTCCGGCT | 87638636 | 87638658 | 0.05 |
| GTTTATTTCCGGCTGGGCTGG | 87638643 | 87638665 | 0.01 |
| GCTTGCACCACCAGCCCAGC | 87638650 | 87638672 | 0.00 |
| GTTTATTGAGTCCTCGCACTC | 87638674 | 87638696 | 0.05 |

| | | | |
|---|---|---|---|
| GTCGCACTCTGGCTGAAGATC | 87638686 | 87638708 | 0.00 |
| GGCTGAAGATCTGGCAGGT | 87638695 | 87638716 | 0.03 |
| GATCTGGCAGGTAGGTCTC | 87638702 | 87638723 | 0.01 |
| GCAGGTAGGTCTCTGGTTGA | 87638708 | 87638730 | 0.01 |
| GTAGGTCTCTGGTTGAAGGCC | 87638713 | 87638735 | 0.02 |
| GGCCTGGTCTATGCAGAGCC | 87638729 | 87638751 | 1.91 |
| GTATGCAGAGCCAGGCCGGCC | 87638738 | 87638760 | 0.49 |
| GATATGTAGCCCTGGCCGGCC | 87638747 | 87638769 | 0.24 |
| GACATAATATGTAGCCCTGGC | 87638752 | 87638774 | 0.02 |
| GTCTGTGATTCCAGACCTT | 87638799 | 87638820 | 0.08 |
| GACCTTAGGATTGTGAGCTT | 87638812 | 87638834 | 0.01 |
| GTTGTGAGCTTGGGGCTAATC | 87638822 | 87638844 | 0.01 |
| GTGGACAACTTCGTTGTTGC | 87638901 | 87638923 | 0.01 |
| GAGGTGTAGATTACTACCCG | 87638920 | 87638942 | 0.01 |
| GCACCTCTTAGCCCAGCACCG | 87638937 | 87638959 | 0.02 |
| GCCCAGCACCGAGGAGACCA | 87638946 | 87638968 | 0.02 |
| GTTCTTTGTCCTTGGTCTCCT | 87638954 | 87638976 | 1.50 |
| GGACAAAGAATCTCTAGTTT | 87638967 | 87638989 | 2.92 |
| GAATCTCTAGTTTGGGGCCA | 87638974 | 87638996 | 0.08 |
| GCTAGTTTGGGGCCAGGGAAA | 87638980 | 87639002 | 0.00 |
| GGAAATGGCTCAGTGACTGA | 87638995 | 87639017 | 0.04 |
| GTCAGTGACTGAAGGTACTTG | 87639004 | 87639026 | 0.77 |
| GAAGGTACTTGTGGATCAGG | 87639013 | 87639035 | 0.08 |
| GGCTTAGTGACCAGAGTCCC | 87639034 | 87639056 | 0.02 |
| GAGTCCCAGGACCCAGGAAG | 87639047 | 87639069 | 0.03 |
| GCTTCCCCTTCCTGGGTCCT | 87639051 | 87639073 | 1.07 |
| GCTTCTCGCTTCCCCTTCCT | 87639058 | 87639080 | 0.01 |
| GGGTAAGTACAGAGGTCAGA | 87639093 | 87639115 | 0.14 |
| GACGCTGAGGGGTAAGTACAG | 87639101 | 87639123 | 1.21 |
| GCTGGAGCAGGGCACGCTGA | 87639114 | 87639136 | 0.01 |
| GTTTACTCCCTGGCTGGAGCA | 87639125 | 87639147 | 0.00 |
| GCCTTGCATTTACTCCCTGGC | 87639132 | 87639154 | 0.02 |
| GAGAGAGAGAGACACGACAA | 87639209 | 87639231 | 0.01 |
| GCAAGGGAAGAAATACCCCAC | 87639226 | 87639248 | 0.03 |
| GACAGGTTAGCACACCTGTG | 87639240 | 87639262 | 0.01 |
| GCTTTGGACTGTGCTCATGAC | 87639257 | 87639279 | 0.04 |
| GACAGTCCAAAGCTCTCTCCT | 87639269 | 87639291 | 0.01 |
| GCAGTGCCAAGGAGAGAGCTT | 87639274 | 87639296 | 0.15 |
| GCTCTCTCCTTGGCACTGCCA | 87639280 | 87639302 | 0.01 |
| GGGGTGCCTTGGCAGTGCCA | 87639286 | 87639308 | 0.01 |
| GAGGAAGACTGGGGGTGCCT | 87639297 | 87639319 | 0.01 |
| GCCCCAAGGGAGGAAGACTG | 87639306 | 87639328 | 0.02 |
| GGTGGGACTGGCCCCAAGGG | 87639316 | 87639338 | 1.96 |
| GTCCCACCCCGCAGAGTTCT | 87639331 | 87639353 | 3.93 |
| GATGTGGCCAAGAACTCTGCG | 87639337 | 87639359 | 0.01 |
| GAAGTCTGTTAAGGAAATG | 87639354 | 87639375 | 0.01 |
| GAGTGAATCGAAGTCTGTTA | 87639362 | 87639384 | 0.01 |
| GCCTGTATGTTGTTAATTTTC | 87639406 | 87639428 | 0.93 |
| GAAATTAACAACATACAGGAA | 87639411 | 87639433 | 8.99 |
| GAACATACAGGAAAGGGGAAG | 87639419 | 87639441 | 0.05 |
| GGAAAGGGGAAGTGGGAAC | 87639427 | 87639448 | 0.50 |
| GGGAACTGGCCAAGATTAGA | 87639440 | 87639462 | 0.01 |

| | | | |
|---|---|---|---|
| GATATCACACCGTCTAATCT | 87639449 | 87639471 | 0.00 |
| GACGGTGTGATATCACAAAG | 87639458 | 87639480 | 0.02 |
| GATCACAAAGTGGCCAGTGTG | 87639469 | 87639491 | 0.35 |
| GAGTGTGAGTCGCCTCACAC | 87639481 | 87639503 | 0.01 |
| GACTCACACTCAAATTATAGC | 87639494 | 87639516 | 0.01 |
| GTCTCTTTTGTCATTTAAGAG | 87639535 | 87639557 | 0.00 |
| GTCATTTAAGAGAGGGGGGA | 87639543 | 87639565 | 0.00 |
| GGGTCTATCTACATAGTTT | 87639563 | 87639584 | 0.04 |
| GCTACATAGTTTTGGCTCTCC | 87639571 | 87639593 | 0.02 |
| GTTGGCTCTCCTGGAACTTAC | 87639581 | 87639603 | 0.00 |
| GGTCAACACCGGTAAGTTCC | 87639589 | 87639611 | 0.02 |
| GCTTACCGGTGTTGACCAGGC | 87639596 | 87639618 | 0.02 |
| GCCGACAGAGGTAGAGGCTGG | 87639637 | 87639659 | 0.12 |
| GCCTCTACCTCTGTCGGGCT | 87639643 | 87639665 | 0.51 |
| GCTTTAATCCCAGCCCGACAG | 87639650 | 87639672 | 0.00 |
| GAGATAGCATTTGTATGACA | 87639724 | 87639746 | 0.00 |
| GATTTGTATGACAAGGCACAT | 87639732 | 87639754 | 0.00 |
| GATGACAAGGCACATAGGTGG | 87639738 | 87639760 | 0.01 |
| GCACATAGGTGGAGGACTAA | 87639746 | 87639768 | 0.03 |
| GAAGGCGTGCGCCAGCATTTC | 87639765 | 87639787 | 0.01 |
| GGTCTTGAGCCAGAAATGC | 87639775 | 87639796 | 0.01 |
| GGCTCAAGACCACTTCTTAA | 87639786 | 87639808 | 3.61 |
| GTCAATCATGCCTTTAAGAAG | 87639795 | 87639817 | 1.13 |
| GACAGAATGAGTTGACAGT | 87639816 | 87639837 | 0.01 |
| GTCTGTGACAACTACAAGC | 87639900 | 87639921 | 1.06 |
| GTTGCTCTCTGAATGAATA | 87639928 | 87639949 | 0.03 |
| GAATGCCAGTGTACTTCCTA | 87639951 | 87639973 | 0.01 |
| GTGTACCATAGGAAGTACAC | 87639956 | 87639978 | 0.01 |
| GACAGTATTCTGTGTACCAT | 87639967 | 87639989 | 0.01 |
| GAAGATTGATTTACTCACGTT | 87639997 | 87640019 | 0.43 |
| GAAATCAATCTTCTTATTGCC | 87640009 | 87640031 | 0.01 |
| GATTATCGAGATTAAACTTCC | 87640027 | 87640049 | 0.01 |
| GATAATACTTTTCAAATCG | 87640044 | 87640065 | 0.00 |
| GGCTCCTCGAGTTGTTTTAT | 87640064 | 87640086 | 0.01 |
| GGCTCTAATCTGTTATTTTG | 87640085 | 87640107 | 0.01 |
| GCTTCAGCCAGTAATTCAGTG | 87640115 | 87640137 | 0.01 |
| GCCAGTAATTCAGTGCGGGG | 87640120 | 87640142 | 0.10 |
| GATTCAGTGCGGGGGGGGGGG | 87640127 | 87640149 | 0.04 |
| GGGGGGGGGGAATAGAAGGG | 87640143 | 87640165 | 0.14 |
| GAATAGAAGGGCGGGTAGAA | 87640152 | 87640174 | 0.09 |
| GAAGGGCGGGTAGAATGGAA | 87640157 | 87640179 | 0.11 |
| GAAGGGCGCGTGAATAATCCT | 87640175 | 87640197 | 1.69 |
| GCGTGAATAATCCTTGGCC | 87640181 | 87640202 | 0.02 |
| GTTCTGCTGCCATCCAGGCCA | 87640192 | 87640214 | 0.01 |
| GTTTTTATTCTGCTGCCATCC | 87640198 | 87640220 | 0.01 |
| GCAGAATAAAAAGCTGTGCTC | 87640210 | 87640232 | 0.04 |
| GCTCTGGGCACGTGAAGCCG | 87640226 | 87640248 | 0.93 |
| GAAGCCGCGGCTGTCTCTCAT | 87640240 | 87640262 | 0.10 |
| GTACTGTCATCGCCTTTCCAG | 87640267 | 87640289 | 0.07 |
| GCATTTAGACGCCACTGGAA | 87640278 | 87640300 | 1.10 |
| GCCAAGCATTTAGACGCCAC | 87640283 | 87640305 | 0.62 |
| GAAATGACTCACAGCAAGTC | 87640316 | 87640338 | 0.18 |

| | | | |
|---|---|---|---|
| GCTCACAGCAAGTCTGGGAGG | 87640323 | 87640345 | 0.02 |
| GGGAGGAGGATAAAGCCCGA | 87640337 | 87640359 | 1.40 |
| GTTGTTCTGGATCGCCCCTT | 87640353 | 87640375 | 0.39 |
| GGGGCGATCCAGAACAACGA | 87640358 | 87640380 | 0.94 |
| GTACAGCCCATCGTTGTTC | 87640366 | 87640387 | 0.13 |
| GACCCCGACTGCGACGAGCA | 87640388 | 87640410 | 0.03 |
| GTTCAAAGCCAAGCAGTGCAA | 87640415 | 87640437 | 0.01 |
| GGCGGTGCCGTTGCACTGCT | 87640422 | 87640444 | 1.75 |
| GCAACGGCACCGCCACGTGC | 87640431 | 87640453 | 0.01 |
| GTTGACACACCAGCACGTGG | 87640440 | 87640462 | 0.00 |
| GCTGGTGTGTCAACACCGC | 87640449 | 87640470 | 0.00 |
| GTCGGTTCTTCGGACTCCGG | 87640464 | 87640486 | 0.11 |
| GCCGTGTCCTTGTCGGTTCTT | 87640474 | 87640496 | 0.01 |
| GTGATCTCCGTGTCCTTGT | 87640482 | 87640503 | 0.01 |
| GTCACGTGCTCCGAGCGCGTG | 87640500 | 87640522 | 0.43 |
| GTCACTAGGTCCTCACGCGCT | 87640509 | 87640531 | 0.01 |
| GCACTGGAAAGCTCACTCACT | 87640524 | 87640546 | 0.01 |
| GAAAGCTAAACTTAAGCGCAC | 87640541 | 87640563 | 0.01 |
| GCAAATAAGATTTTTGTGCCT | 87640570 | 87640592 | 0.01 |
| GCCTCGGAATTCAAAAAACC | 87640586 | 87640608 | 0.01 |
| GTTCAAAAAACCGGGAAAAGC | 87640595 | 87640617 | 0.01 |
| GCTACTACGCCCGGCTTTTCC | 87640604 | 87640626 | 0.08 |
| GAAAGCCGGGCGTAGTAGTTC | 87640610 | 87640632 | 0.03 |
| GGTCTCAAACTCACTGTGGC | 87640634 | 87640656 | 0.26 |
| GGGCTACTTGAGATCCCTTT | 87640662 | 87640684 | 0.06 |
| GGACACTTGTCACTCCAAAA | 87640676 | 87640698 | 0.16 |
| GCATGAGGCACGGAGACAGGA | 87640697 | 87640719 | 0.35 |
| GACTGCCATTTCATGAGGCA | 87640708 | 87640730 | 0.10 |
| GCTGGTGACTGCCATTTCATG | 87640713 | 87640735 | 0.24 |
| GGCAGTCACCAGCCTTGAGT | 87640724 | 87640746 | 0.02 |
| GGCCAATAAAGCCCAACTCA | 87640736 | 87640758 | 0.47 |
| GAAACAAATATAACTGGATTA | 87640757 | 87640779 | 0.02 |
| GCCAAGAAAACAAATATAAC | 87640764 | 87640786 | 0.01 |
| GAATCCAGCTGTGTTGTTTTG | 87640790 | 87640812 | 0.00 |
| GTTGCCGAAGCTTTTTATCCT | 87640816 | 87640838 | 0.13 |
| GTCACACTTAATGACTTGCCG | 87640833 | 87640855 | 0.01 |
| GAAGTCATTAAGTGTGAGGAA | 87640840 | 87640862 | 0.00 |
| GTAAGTGTGAGGAATGGAAGG | 87640847 | 87640869 | 0.51 |
| GTGAGGAATGGAAGGAGGCC | 87640852 | 87640874 | 0.02 |
| GGAGGCCTGGAATGATCTC | 87640865 | 87640886 | 0.02 |
| GCTGAGGCCAGAGATCATTCC | 87640870 | 87640892 | 0.14 |
| GTCTGGAAATGTCACAACCTG | 87640887 | 87640909 | 1.60 |
| GGTTGTGACATTTCCAGAGC | 87640892 | 87640914 | 0.03 |
| GCAGGACTCAGAAGCCAGCTC | 87640905 | 87640927 | 0.04 |
| GCTTCTGAGTCCTGTGTAGAC | 87640915 | 87640937 | 0.10 |
| GAGTCCTGTGTAGACAGGCT | 87640920 | 87640942 | 0.56 |
| GTGTAGACAGGCTGGGTCTT | 87640927 | 87640949 | 0.01 |
| GGCTGGGTCTTAGGTTTTA | 87640936 | 87640957 | 0.02 |
| GGTCTTAGGTTTTACGGAAA | 87640941 | 87640963 | 0.01 |
| GGTTTTACGGAAACGGTAGT | 87640948 | 87640970 | 0.01 |
| GAAACGGTAGTGGGCGTGACA | 87640958 | 87640980 | 2.47 |
| GACATGGGTTACTGAACCTC | 87640974 | 87640996 | 0.00 |

| | | | |
|---|---|---|---|
| GAACTCCAAAACGGGGACCAG | 87640990 | 87641012 | 0.26 |
| GACTAATCCAACTCCAAAACG | 87640998 | 87641020 | 13.76 |
| GTAGTGTAAAGTGCGTGTGAA | 87641017 | 87641039 | 0.01 |
| GCTTAAACTGAACGCACTCA | 87641039 | 87641061 | 1.78 |
| GTCATGGATTGAGTCCTAAAG | 87641056 | 87641078 | 3.00 |
| GTAAAGCGGGGCTGTTTGTGC | 87641071 | 87641093 | 0.01 |
| GCTGTTTGTGCTGGAGACAAG | 87641081 | 87641103 | 0.02 |
| GAGACAAGTGGTAGAGAAGC | 87641093 | 87641115 | 0.01 |
| GGCTCTCTCCCCAAGCACAG | 87641116 | 87641138 | 0.02 |
| GCTCTCTGCCCTCTGTGCTTG | 87641124 | 87641146 | 0.01 |
| GAGATGGCTGAGAGCAGAC | 87641144 | 87641165 | 0.00 |
| GAACAGTGGCTTCGGCTTGAC | 87641169 | 87641191 | 0.69 |
| GGTTCTTCCAACAGTGGCTT | 87641178 | 87641200 | 0.03 |
| GTTCCCAGGTTCTTCCAACAG | 87641184 | 87641206 | 0.02 |
| GTTGGAAGAACCTGGGAAGG | 87641189 | 87641211 | 0.04 |
| GCTGGCCTGCCGCCTTCCC | 87641199 | 87641220 | 0.10 |
| GCTACAGCACACGAGGCCAGC | 87641216 | 87641238 | 0.01 |
| GCTACGGCTACAGCACACG | 87641224 | 87641245 | 0.06 |
| GTCTGACACTGACTGCTGCTA | 87641239 | 87641261 | 0.00 |
| GCAGCAGTCAGTGTCAGAAA | 87641244 | 87641266 | 0.01 |
| GTCAGAAAGGGCGTTTCCTTG | 87641257 | 87641279 | 0.23 |
| GTTGTGGGTTCACTTTCTCGC | 87641274 | 87641296 | 0.02 |
| GTTCACTTTCTCGCAGGATA | 87641280 | 87641302 | 0.30 |
| GTCTCGCAGGATATGGGAGGC | 87641288 | 87641310 | 0.99 |
| GGTTCACCACAACGCTTAC | 87641309 | 87641330 | 0.01 |
| GAACAGAACCTGTAAGCGTTG | 87641315 | 87641337 | 0.01 |
| GCATCCCTCCCTGTCCAGAAT | 87641344 | 87641366 | 0.01 |
| GCAGAATTCCAATTCTGGACA | 87641351 | 87641373 | 0.01 |
| GAATTGGAATTCTGGCCTCTT | 87641361 | 87641383 | 0.33 |
| GTTTTACGCTGTTTTCCAAAG | 87641375 | 87641397 | 0.02 |
| GATCCAGCTGTAAGACAAGA | 87641417 | 87641439 | 0.05 |
| GCAAGCTCTGATGGTCGTAG | 87641472 | 87641494 | 0.37 |
| GCTCACGTCTGCAAGCTCTGA | 87641481 | 87641503 | 0.03 |
| GTGCATAAATACAGTAGTAT | 87641514 | 87641536 | 0.08 |
| GCTGTATTTATGCACCTTGAC | 87641523 | 87641545 | 0.04 |
| GCTTATATAGTTTTCCTGTCA | 87641536 | 87641558 | 0.03 |
| GTGATCATGTGTCTTTCCC | 87641558 | 87641579 | 0.04 |
| GGCCCAGATCACATCACGAT | 87641578 | 87641600 | 0.01 |
| GAAGCCCATCGTGATGTGATC | 87641581 | 87641603 | 0.01 |
| GCTGGCAGACATAGCTTGTAA | 87641608 | 87641630 | 0.04 |
| GAATTTCACAATAGCGAAACC | 87641627 | 87641649 | 0.02 |
| GAAATTACCATTTTCCTTTT | 87641644 | 87641666 | 0.01 |
| GATAAACTCCAAAAAGGAAAA | 87641651 | 87641673 | 0.01 |
| GATTATTCATAAACTCCAAAA | 87641658 | 87641680 | 0.01 |
| GATAATCCATTGACAGATACG | 87641676 | 87641698 | 2.12 |
| GAATACCCACGTATCTGTCAA | 87641681 | 87641703 | 0.02 |
| GATCGGAGTGTCAGTCTGATT | 87641720 | 87641742 | 0.00 |
| GACACTCCGATGGCTGATGC | 87641732 | 87641754 | 2.03 |
| GTTCAAGCCAGCATCAGCCAT | 87641738 | 87641760 | 0.01 |
| GTAATTTTAAATCACAGTAAG | 87641761 | 87641783 | 0.01 |
| GACTTGGGACTTGGTGTGCAA | 87641885 | 87641907 | 0.03 |
| GCCCTAGCTGACTTGGGACT | 87641895 | 87641917 | 1.78 |

| | | | |
|---|---|---|---|
| GTAGCTAGCCCTAGCTGACT | 87641902 | 87641924 | 0.76 |
| GTTTTTTTTCTTTAAAGAAGA | 87641933 | 87641955 | 0.01 |
| GCATCTTAAGTAGATAGCTCA | 87642023 | 87642045 | 0.55 |
| GAAGATGTAGAGATTCTATTA | 87642040 | 87642062 | 0.03 |
| GTATTTGCAGTGCGCTTCAAG | 87642092 | 87642114 | 0.00 |
| GAAATTTATCAAAAACATTA | 87642143 | 87642165 | 0.05 |
| GAAACATTATGGTAAGTTTTG | 87642155 | 87642177 | 0.01 |
| GTTGTTTTTTGTTTTGTTTGT | 87642196 | 87642218 | 0.01 |
| GTTTTAGCAGCAAGTTGGTA | 87642252 | 87642274 | 0.42 |
| GCAAGTTGGTAGGGGAGTG | 87642261 | 87642282 | 0.02 |
| GGTGTGGGAGAGAGGAGCAG | 87642301 | 87642323 | 0.12 |
| GCCTGTCTGGGTGTGGGAGAG | 87642309 | 87642331 | 2.19 |
| GAGAGACCCTGTCTGGGTGT | 87642316 | 87642338 | 0.03 |
| GGCATGTTCCATCACTGCCC | 87642436 | 87642458 | 0.01 |
| GCTATTCGCCAGGGCAGTGA | 87642444 | 87642466 | 0.01 |
| GAAATCACTGGCTATTCGCCA | 87642453 | 87642475 | 0.08 |
| GAATAGCCAGTGATTTTTAA | 87642460 | 87642482 | 0.02 |
| GTAGCTACCATTAAAAATCAC | 87642466 | 87642488 | 1.07 |
| GTTTAGTATCAATTTAAATCC | 87642496 | 87642518 | 0.02 |
| GTAAATCCTGGGCACTTAATA | 87642509 | 87642531 | 3.93 |
| GGATTCCATATTAAGTGCCC | 87642514 | 87642536 | 0.04 |
| GTCGATGGAAGGAAAACTACT | 87642535 | 87642557 | 0.16 |
| GAGCTTGTGAGGTCGATGGA | 87642547 | 87642569 | 0.68 |
| GGGTACACTGGAGCTTGTG | 87642558 | 87642579 | 0.02 |
| GCAAGCTCCAGTGTACCCTTA | 87642563 | 87642585 | 0.04 |
| GACGTGTCCTTAAGGGTACAC | 87642569 | 87642591 | 0.01 |
| GTACCCTTAAGGACACGTAA | 87642574 | 87642596 | 0.00 |
| GAAGTAGTGTCTCTTCTTATG | 87642599 | 87642621 | 0.15 |
| GTTACACCTCGAAGAGTGCTC | 87642635 | 87642657 | 0.01 |
| GCAATCCCAGAGCACTCTTCG | 87642640 | 87642662 | 3.23 |
| GTTCTCTGCGCGTCCTGCCAC | 87642665 | 87642687 | 0.01 |
| GGGTAAGGACAGCCTGTGGC | 87642677 | 87642699 | 0.06 |
| GCTGTCCTTACCCAGTGTCT | 87642687 | 87642709 | 0.01 |
| GGAAACCAAGACACTGGGTA | 87642692 | 87642714 | 0.01 |
| GGAAGGGAAACCAAGACACT | 87642697 | 87642719 | 0.01 |
| GCCTTATCACAGCAACAGGAA | 87642713 | 87642735 | 0.01 |
| GGGCACCTTATCACAGCAAC | 87642718 | 87642740 | 0.07 |
| GCTCCTAACTCGATTTCTTCA | 87642738 | 87642760 | 0.26 |
| GAAATCGAGTTAGGAGAGAG | 87642745 | 87642767 | 0.27 |
| GGAGAGAGAGGGTTTATTGG | 87642757 | 87642779 | 0.01 |
| GGTTTATTGGTGGTAGTTTG | 87642767 | 87642789 | 0.09 |
| GTAAAAACGTGATGCACACGC | 87642817 | 87642839 | 0.06 |
| GTGTGCATCACGTTTTTACC | 87642822 | 87642844 | 0.01 |
| GACGTTTTTACCTGGTGCTCC | 87642831 | 87642853 | 0.01 |
| GACCTGGTGCTCCAGGTGTTC | 87642839 | 87642861 | 0.00 |
| GTGCTCCAGGTGTTCAGGAA | 87642844 | 87642866 | 0.01 |
| GAATGGCCTTTCCTGAACACC | 87642849 | 87642871 | 0.13 |
| GACGGACACTAGGAGGTCCAA | 87642867 | 87642889 | 0.47 |
| GTCTGTAACACGGACACTAGG | 87642875 | 87642897 | 3.78 |
| GCCGCTAACCATCTGTAACA | 87642886 | 87642908 | 0.33 |
| GAATTAAATCCCTGTCCTCA | 87642912 | 87642934 | 0.01 |
| GACTTGTTGCCCTTGAGGACA | 87642921 | 87642943 | 0.46 |

| | | | |
|---|---|---|---|
| GTATAGCACTTGTTGCCCTTG | 87642927 | 87642949 | 0.02 |
| GACCATGTCTTCAGCCCCAAC | 87642959 | 87642981 | 0.09 |
| GCTGGAATTGTAAACCCTGTT | 87642973 | 87642995 | 16.81 |
| GTCCAGATTAGAGTCTCCTTG | 87642991 | 87643013 | 0.01 |
| GCTGCAGTTGACTCCCACA | 87643006 | 87643027 | 0.65 |
| GCAGCAGAAACTCACTGTG | 87643023 | 87643044 | 0.38 |
| GGATGTTGCTAGCTGCTTAA | 87643048 | 87643070 | 0.01 |
| GATTCTCTCTGCTCTTGATGG | 87643069 | 87643091 | 0.00 |
| GAAAAACAGAAAGCTGGGCA | 87643109 | 87643131 | 0.01 |
| GATAAAGAAAAACAGAAAGCT | 87643114 | 87643136 | 0.02 |
| GTCTGTTTTTCTTTATAACCC | 87643122 | 87643144 | 0.01 |
| GTATAACCCAGGCCCCAGCCT | 87643134 | 87643156 | 0.01 |
| GCACTTCCTAGGCTGGGGCCT | 87643139 | 87643161 | 0.00 |
| GCCTGCACCACTTCCTAGGC | 87643147 | 87643169 | 0.06 |
| GTAATTAAGGTGGGAAGATTA | 87643169 | 87643191 | 0.13 |
| GTCTATTATGTTAATTAAGGT | 87643179 | 87643201 | 0.01 |
| GAATCCTCACAGATTTGCCAT | 87643205 | 87643227 | 1.01 |
| GCCATAGGTCAACCCAATAT | 87643220 | 87643242 | 0.01 |
| GATGAGGGACTGCCTATATT | 87643232 | 87643254 | 0.04 |
| GAAGCATTTGAGTCTCGATG | 87643248 | 87643270 | 0.01 |
| GAACTAACCATCATACCTAGT | 87643292 | 87643314 | 0.01 |
| GAAAGGACCAACTAGGTATGA | 87643298 | 87643320 | 0.01 |
| GTTAAGTAAAGGACCAACT | 87643306 | 87643327 | 0.03 |
| GTTTTTTTAAGAGTTAAGTAA | 87643316 | 87643338 | 0.18 |
| GTAATTGAAAAATCTTTTAG | 87643381 | 87643403 | 0.12 |
| GTGATAAATTATTCATTAA | 87643406 | 87643427 | 0.02 |
| GATTCATTAATGGATGCATCC | 87643416 | 87643438 | 0.01 |
| GTCGTGTAATCTACACCCACC | 87643434 | 87643456 | 0.01 |
| GATAACATTATTCTCATACT | 87643536 | 87643558 | 4.27 |
| GAGTTTTGCATCAGATCAA | 87643560 | 87643581 | 0.01 |
| GTCAGAAAACACAAGACGACG | 87643585 | 87643607 | 0.31 |
| GACGTGGACATAGCTGATG | 87643601 | 87643622 | 0.00 |
| GATTAAGCTTTAAAAATTACA | 87643654 | 87643676 | 1.82 |
| GATCTCAATATATATATCCC | 87643705 | 87643727 | 0.03 |
| GCTTGAGTGCCAGGACAGCCA | 87643722 | 87643744 | 0.11 |
| GTGTGTGTATCTTGAGTGCC | 87643732 | 87643754 | 0.01 |
| GTATCTATCTATCTATATCCC | 87643874 | 87643896 | 0.03 |
| GTGTGACTGCAGGGACAGCC | 87643892 | 87643914 | 0.03 |
| GGCAGATCTGTGTGACTGCA | 87643901 | 87643923 | 1.53 |
| GAACCATGTCCTTTTTGAGAC | 87643963 | 87643985 | 0.01 |
| GTACAAGAACCTGTCTCAAAA | 87643971 | 87643993 | 0.01 |
| GAGACAGGTTCTTGTAGGC | 87643978 | 87643999 | 1.46 |
| GGTTCTTGTAGGCTGGCCT | 87643984 | 87644005 | 0.01 |
| GGGCCCAGAACTTGGGAGA | 87644025 | 87644046 | 0.02 |
| GTAGACAGGGCCCAGAACTT | 87644031 | 87644053 | 0.01 |
| GCTACTCTCTCTTTGTAGACA | 87644044 | 87644066 | 1.87 |
| GACAAAGAGAGAGTAGTAATT | 87644052 | 87644074 | 0.01 |
| GTTGTAAAGCTCATCTTTGGA | 87644098 | 87644120 | 0.01 |
| GATGAGCTTTACAAGGAGAA | 87644107 | 87644129 | 1.44 |
| GAAAGGGCCTTGCAGTCCCA | 87644124 | 87644146 | 0.01 |
| GCAGTCCCAGGGGCTGTGGT | 87644135 | 87644157 | 0.27 |
| GTGGTCCAACCACAGCCCCT | 87644140 | 87644162 | 0.23 |

| | | | |
|---|---|---|---|
| GCTGTGGTTGGACCACTAGGT | 87644148 | 87644170 | 0.03 |
| GCATAAAGCTCTCCTACCTAG | 87644159 | 87644181 | 0.56 |
| GTAGGAGAGCTTTATGAGGGA | 87644167 | 87644189 | 0.24 |
| GGTGCATCATTCCAGAGCCT | 87644188 | 87644210 | 0.49 |
| GATTCCAGAGCCTGGGGGTCA | 87644196 | 87644218 | 0.00 |
| GATGTCCTCCCCTTGACCCCC | 87644205 | 87644227 | 0.26 |
| GCATCTACACTTTGTGTAAAG | 87644230 | 87644252 | 0.66 |
| GCACTTTGTGTAAAGTGGAGC | 87644236 | 87644258 | 0.24 |
| GTAAAGTGGAGCAGGAAGA | 87644244 | 87644265 | 1.74 |
| GACCATGTCCTGTTCTTTTAG | 87644281 | 87644303 | 0.01 |
| GCACAGGTCCTCTAAAAGAAC | 87644288 | 87644310 | 0.61 |
| GCGGCCTGGGGATCAAACAC | 87644305 | 87644327 | 0.02 |
| GCTTCAGGTGATAGCGGCCTG | 87644317 | 87644339 | 0.00 |
| GTTCTGAACTTCAGGTGATAG | 87644324 | 87644346 | 0.39 |
| GCATCTGTTATTCTGAACTTC | 87644333 | 87644355 | 1.06 |
| GTCAGAATAACAGATGGCTGG | 87644341 | 87644363 | 0.57 |
| GGTGGGTGGGCACTGCCACT | 87644359 | 87644381 | 0.01 |
| GCACTGCCACTTGGGAGGCAG | 87644369 | 87644391 | 0.00 |
| GGATTTCGTTTTTCAAGACA | 87644464 | 87644486 | 0.03 |
| GTATTGTCTTCAATTGTTGTT | 87644485 | 87644507 | 0.00 |
| GATAGAGATGTCTACCATGAC | 87644547 | 87644569 | 0.82 |
| GATGTCTACCATGACAGGAGC | 87644553 | 87644575 | 0.49 |
| GTCGCCAGCCTGCTCCTGTCA | 87644560 | 87644582 | 1.18 |
| GCTGAGAAAGCTACGAAAGAA | 87644589 | 87644611 | 0.58 |
| GTTCGTAGCTTTCTCAGAGAC | 87644596 | 87644618 | 0.01 |
| GTTCTCAGAGACTGGGGAGCT | 87644605 | 87644627 | 0.03 |
| GGCCATAGTCAGATCATGTT | 87644626 | 87644648 | 0.64 |
| GTCATGTTTGGGAGGGTGTCC | 87644639 | 87644661 | 0.01 |
| GTCCAGGTATCAGCATTGTT | 87644655 | 87644677 | 0.01 |
| GTTGTTAGGAGCAGAGAGATG | 87644670 | 87644692 | 0.01 |
| GAAGCACAAATCCTGCTAGC | 87644692 | 87644714 | 0.09 |
| GAATCCTGCTAGCAGGTGATA | 87644700 | 87644722 | 0.03 |
| GGTGATATGGAAACCAAAGC | 87644713 | 87644735 | 1.58 |
| GCATGCATTTCCTACCAGCTT | 87644726 | 87644748 | 0.24 |
| GAATGCATGGCCTGCTATCTA | 87644741 | 87644763 | 0.00 |
| GCCTCCTTCTCCTTAGATAGC | 87644750 | 87644772 | 0.06 |
| GATCTAAGGAGAAGGAGGGAC | 87644756 | 87644778 | 0.01 |
| GACACTTGTAGTCTAGCACT | 87644798 | 87644820 | 0.01 |
| GACTAGTTTATGTCATACTG | 87644826 | 87644848 | 0.33 |
| GTTTATGTCATACTGAGGAC | 87644831 | 87644853 | 0.56 |
| GTCATACTGAGGACTGGTACC | 87644838 | 87644860 | 0.01 |
| GACTGGTACCTGGACATGCT | 87644848 | 87644870 | 11.02 |
| GAATGCTTCCCTAGCATGTCC | 87644856 | 87644878 | 0.00 |
| GAGTCAGAGGGTCTAGAAAC | 87644882 | 87644904 | 0.31 |
| GAAGATTAAAACAGGAGTCAG | 87644895 | 87644917 | 0.00 |
| GTTTTAATCTTTGCTGTGTT | 87644907 | 87644929 | 0.01 |
| GCAGCATACTGGAATACATAC | 87644938 | 87644960 | 0.03 |
| GCTAGACTGTTCCCAGCATAC | 87644950 | 87644972 | 0.00 |
| GTCTAGAACAGTCTACTACT | 87644967 | 87644989 | 0.03 |
| GTGTAAGAAAAGAGAAAACT | 87645043 | 87645065 | 0.02 |
| GAATTATTAGAATATTGGATG | 87645141 | 87645163 | 0.00 |
| GCATAGAAATTATTAGAATAT | 87645147 | 87645169 | 0.05 |

| | | | |
|---|---|---|---|
| GTTCTAATAATTTCTATGAAT | 87645153 | 87645175 | 0.00 |
| GAGTCAGTCCCCCATTTTAA | 87645175 | 87645197 | 0.17 |
| GAGAATCAGCCTTTAAAATG | 87645184 | 87645206 | 0.41 |
| GGCTGATTCTCATTCTGTGT | 87645196 | 87645218 | 0.08 |
| GATCTATGTGCATGTTAGCAC | 87645236 | 87645258 | 0.01 |
| GCACAGGTGCCTTGAGACCAG | 87645253 | 87645275 | 5.32 |
| GCGCTTAGCCTCTGGTCTCA | 87645261 | 87645283 | 0.23 |
| GCTGGGGAAGCGCTTAGCCTC | 87645269 | 87645291 | 0.03 |
| GTAACTGTAATCGAGGTCCT | 87645287 | 87645309 | 0.01 |
| GACAAACTGTAACTGTAATCG | 87645294 | 87645316 | 0.00 |
| GCTATGAAGAGGATTTGAGTT | 87645346 | 87645368 | 0.02 |
| GTTGACATCTCAGCTATGAAG | 87645358 | 87645380 | 0.39 |
| GACATGGAGAAACTCTGTCAC | 87645402 | 87645424 | 0.54 |
| GCTATAAATTTTAGGCCAGCC | 87645457 | 87645479 | 0.02 |
| GGTGAATCTCTATAAATTTT | 87645466 | 87645488 | 0.02 |
| GCTTCAATCCCATCTCTCGGG | 87645497 | 87645519 | 0.20 |
| GAAATGGAATCTGGGCAGTGG | 87645527 | 87645549 | 7.10 |
| GATGTTTTTAAAATGGAATCT | 87645536 | 87645558 | 0.03 |
| GTGCTCACATGTTTTTAAAA | 87645544 | 87645566 | 0.01 |
| GTAAAAACATGTGAGCACCCG | 87645550 | 87645572 | 0.00 |
| GACAGTGTATTCTACCACCTC | 87645566 | 87645588 | 0.01 |
| GCACTGTAATTCTGACACTGA | 87645583 | 87645605 | 0.90 |
| GAATTCTGACACTGAAGGCTG | 87645589 | 87645611 | 0.36 |
| GACACTGAAGGCTGAGGCAG | 87645595 | 87645617 | 0.01 |
| GAAGACTGTCTCAAAACAAAG | 87645682 | 87645704 | 0.03 |
| GACAGTCTTACGTTAGTCT | 87645696 | 87645717 | 0.09 |
| GCTAGGTTGACCTAGAATTCA | 87645713 | 87645735 | 0.01 |
| GACCTAGAATTCATGGTAGA | 87645720 | 87645742 | 0.08 |
| GCTTCAGCCTGAGTTCTCCGA | 87645747 | 87645769 | 0.01 |
| GCCTGAGTTCTCCGATGGTA | 87645752 | 87645774 | 0.39 |
| GTCCGATGGTAGGGTTACAGA | 87645762 | 87645784 | 0.35 |
| GCAGATGGAAGATACTGCACC | 87645778 | 87645800 | 0.01 |
| GTAATAAACACACGCTTAGCC | 87645796 | 87645818 | 0.01 |
| GCGTGTGTTTATTACAGTG | 87645805 | 87645826 | 0.03 |
| GCAATTTACTTCAGAATACTT | 87645834 | 87645856 | 0.03 |
| GACTTCAGAATACTTTGGGCA | 87645840 | 87645862 | 0.00 |
| GACTTTGGGCATGGAAGATGA | 87645850 | 87645872 | 0.00 |
| GAAGAAAGAATGAGACTGAAT | 87645878 | 87645900 | 0.15 |
| GTTGTCATGTTTTAGTCTGG | 87645907 | 87645929 | 0.01 |
| GTTCATTTCTCCTTATGTGGA | 87645944 | 87645966 | 0.02 |
| GGAAAATGCCATCCACATA | 87645953 | 87645974 | 0.01 |
| GTTAAGGCAGGGATTTATTAC | 87645973 | 87645995 | 0.02 |
| GTCCCTGCCTTAAAGTTTCCC | 87645984 | 87646006 | 0.01 |
| GCTGATCCAGGGAAACTTTA | 87645990 | 87646012 | 0.02 |
| GCATGCTAACAAGCTGATCCA | 87646001 | 87646023 | 0.02 |
| GAAACCAACCGTGAAGATTCT | 87646025 | 87646047 | 0.03 |
| GAACGGCCAAGAATCTTCA | 87646032 | 87646053 | 0.02 |
| GATTCTTGGCCGTTCTCTTC | 87646039 | 87646061 | 0.01 |
| GTTCTCTTCTGGCCCGCAGT | 87646050 | 87646072 | 0.00 |
| GGCGTCTGCATACCAACTGC | 87646062 | 87646084 | 0.00 |
| GCTAGACAGTCTGCAAGGCAC | 87646083 | 87646105 | 0.01 |
| GACTGTTCTAGACAGTCTGCA | 87646089 | 87646111 | 0.02 |

| | | | |
|---|---|---|---|
| GCTACTCTCCTTCTCCTCTGT | 87646114 | 87646136 | 0.16 |
| GTCCTTCTCCTCTGTAGGTGA | 87646120 | 87646142 | 0.12 |
| GACTCCCCCTTCACCTACAG | 87646127 | 87646149 | 0.01 |
| GCTCTTAGAAGAATGGAACA | 87646149 | 87646171 | 0.02 |
| GGTCCATGCTCTTAGAAGAA | 87646156 | 87646178 | 0.01 |
| GCATGGACCTGAGAGTGAA | 87646170 | 87646191 | 0.02 |
| GCGGCTCTCCGTTCACTCTC | 87646177 | 87646199 | 0.00 |
| GAACGGAGAGCCGCTCGATC | 87646186 | 87646208 | 0.00 |
| GAGCCGCTCGATCTGGACCC | 87646193 | 87646215 | 0.01 |
| GTAGTAAATCAGAGTCTGCC | 87646212 | 87646234 | 3.26 |
| GATTTACTACGTTGATGAAA | 87646225 | 87646247 | 0.01 |
| GGCACCCGAGTTCTCCATGC | 87646246 | 87646268 | 0.18 |
| GAGGCCCTGCATGGAGAACT | 87646251 | 87646273 | 8.13 |
| GTCCATGCAGGGCCTCACGGC | 87646259 | 87646281 | 0.01 |
| GCAGCGATGATCCCGGCCGTG | 87646270 | 87646292 | 0.36 |
| GATCATCGCTGTCATTGTGG | 87646282 | 87646304 | 0.67 |
| GGTGTCATTAGCAGTCATCG | 87646303 | 87646325 | 1.03 |
| GCATCGCGGGGATTGTTGTCC | 87646318 | 87646340 | 0.04 |
| GATTGTTGTCCTGGTGAGTAC | 87646328 | 87646350 | 0.14 |
| GACTCATCCCTGTACTCACC | 87646336 | 87646358 | 0.00 |
| GACAGGGATGAGTCAGGGCTC | 87646346 | 87646368 | 0.01 |
| GTCAGGGCTCAGGGAAAGAT | 87646356 | 87646378 | 0.01 |
| GGAAAGATGGGTTCTTATAT | 87646368 | 87646390 | 0.01 |
| GCCAAACACGGGTAGTTTTTC | 87646391 | 87646413 | 0.02 |
| GTGTTTGGAATCTTCAGTGA | 87646406 | 87646428 | 0.00 |
| GAAGGACTGTATTGCGTTTC | 87646424 | 87646446 | 0.01 |
| GAAATGACTATTTTGTAAAG | 87646449 | 87646471 | 0.01 |
| GAAATAGTCATTTCCTAAAGC | 87646459 | 87646481 | 0.02 |
| GGTCAATCTTTTCCAGCTTT | 87646471 | 87646493 | 0.00 |
| GCTGAGGTGTTTTCTCAGGAG | 87646492 | 87646514 | 0.01 |
| GATCAGCTGAGGTGTTTTCTC | 87646497 | 87646519 | 0.01 |
| GGGCTGTGTTCATCAGCTG | 87646509 | 87646530 | 0.78 |
| GACACAGCCCAGTTCCCTGCA | 87646522 | 87646544 | 0.01 |
| GGAATTCCTTGCAGGGAACT | 87646528 | 87646550 | 0.04 |
| GGAGCACGGAATTCCTTGCA | 87646535 | 87646557 | 0.01 |
| GAAGGAATTCCGTGCTCCTGC | 87646541 | 87646563 | 0.01 |
| GCTGTGCTCCGGCAGGAGCA | 87646549 | 87646571 | 0.02 |
| GCTAGTCTGCTGTGCTCCGGC | 87646556 | 87646578 | 0.01 |
| GTGCATCTCAGTGACTGATC | 87646584 | 87646606 | 0.01 |
| GCACCACACTTATCATTTGTT | 87646609 | 87646631 | 0.01 |
| GACTTATCATTTGTTTGGTTT | 87646615 | 87646637 | 0.02 |
| GCATTTGTTTGGTTTTGGGTT | 87646621 | 87646643 | 0.01 |
| GGTGCTGCAAACTGAGCTC | 87646645 | 87646666 | 0.03 |
| GTTAAGCTCTGTGTTGTTTTG | 87646667 | 87646689 | 0.51 |
| GTATGTAGAATCACTGCTACA | 87646715 | 87646737 | 0.08 |
| GATAAATAACAAATTTGCACC | 87646735 | 87646757 | 0.02 |
| GCACCAGGCACGTATAATGAA | 87646751 | 87646773 | 0.01 |
| GATGCTCCTAATCCCAGCACT | 87646777 | 87646799 | 0.02 |
| GTAAAGAAGTCTGCTTTATCC | 87646827 | 87646849 | 0.00 |
| GTCTGCTTTATCCTGGGAAAG | 87646835 | 87646857 | 0.01 |
| GTTTATCCTGGGAAAGAGGGA | 87646840 | 87646862 | 0.02 |
| GAAATTCCTTCCCTCTTTCCC | 87646845 | 87646867 | 0.00 |

| | | | |
|---|---|---|---|
| GGGAAGGAATTTCTGAGTGT | 87646856 | 87646878 | 0.02 |
| GTCTGAGTGTGGGAGGGTAGA | 87646867 | 87646889 | 0.01 |
| GGGTAGAAGGGGCTCCAAG | 87646880 | 87646901 | 0.01 |
| GATAAAGGATATTTCTCCTCT | 87646894 | 87646916 | 0.01 |
| GTAAAACATAAAAATAAATAA | 87646910 | 87646932 | 0.06 |
| GTTCTTCCATATATGTGTGTG | 87646939 | 87646961 | 0.00 |
| GATATACCACACACACATATA | 87646944 | 87646966 | 0.02 |
| GTATATGTGTTCACATGTGT | 87646975 | 87646997 | 0.02 |
| GTGTGTGCAAGTACCCATAG | 87647005 | 87647027 | 0.01 |
| GCAAGTACCCATAGAGGCCTA | 87647012 | 87647034 | 0.01 |
| GCATTACCCTTAGGCCTCTA | 87647019 | 87647041 | 0.02 |
| GATTCCCAGCATTACCCTT | 87647028 | 87647049 | 0.28 |
| GAATCTTGCTCTCTAGCTTTC | 87647046 | 87647068 | 0.80 |
| GTTGAGAGAGTCTATCTTAAA | 87647073 | 87647095 | 0.01 |
| GAAACCCAGAGCAGACTGATA | 87647098 | 87647120 | 0.01 |
| GCAGACTGATATGGTGAGGT | 87647107 | 87647129 | 0.01 |
| GGCTAGTCAGCTCGCTTTC | 87647128 | 87647149 | 2.49 |
| GTCAGCTCGCTTTCAGGCTG | 87647133 | 87647155 | 0.06 |
| GTCATCTCTGCCTTCTAAAGA | 87647160 | 87647182 | 0.01 |
| GCCTGAAATGCCATCTTTAGA | 87647169 | 87647191 | 0.02 |
| GCAGTGGGTAAGTGTGAAAA | 87647193 | 87647215 | 0.06 |
| GACACTTACCCACTGCTTTTG | 87647201 | 87647223 | 0.04 |
| GCTAGAACCCACAAAAGCAGT | 87647208 | 87647230 | 0.48 |
| GACGAGACACTCACACATT | 87647235 | 87647256 | 0.02 |
| GAGTGTCTCGTCTTGTATGG | 87647245 | 87647267 | 0.00 |
| GTCTCGTCTTGTATGGAGGTC | 87647250 | 87647272 | 0.01 |
| GGAGGTCAGGGGTCAATGCC | 87647263 | 87647285 | 0.03 |
| GAGGCACAGAGGAAGACTCC | 87647281 | 87647303 | 0.02 |
| GAAAAAGATGGAGAGGCACAG | 87647292 | 87647314 | 0.00 |
| GAACAAAATAAAAAGATGGAG | 87647300 | 87647322 | 0.02 |
| GAAAATAACAAAATAAAAAGA | 87647305 | 87647327 | 0.35 |
| GATTTATTTATTTGGGCTTGT | 87647328 | 87647350 | 0.01 |
| GTTATTTGGGCTTGTTGGGAC | 87647334 | 87647356 | 0.01 |
| GCTCAAGGCCAATGAGTTCC | 87647381 | 87647403 | 0.01 |
| GTCTCTGGCTGGCCAGGCTCA | 87647396 | 87647418 | 0.45 |
| GCCAGAGCTCTCTGGCTGGCC | 87647403 | 87647425 | 0.01 |
| GACAGAGTTCCCAGAGCTCTC | 87647412 | 87647434 | 0.01 |
| GTCACTGCGTCCCAGCACT | 87647438 | 87647459 | 0.01 |
| GTGTCTGCAAGCCTAGTGCT | 87647448 | 87647470 | 0.36 |
| GCAGACACATGGCTACACA | 87647463 | 87647484 | 0.01 |
| GTTTTTTTACAGGGACACGGG | 87647506 | 87647528 | 0.02 |
| GTGTGTGTTCCCCTGCCTA | 87647579 | 87647600 | 0.94 |
| GCTGGGTAGACTCCATAGGCA | 87647589 | 87647611 | 0.47 |
| GCAGCCTGGGTAGACTCCAT | 87647594 | 87647616 | 0.76 |
| GTACCCAGGCTGCTGGATTCC | 87647605 | 87647627 | 0.05 |
| GTCAGGTGATCAGCTGAATCC | 87647623 | 87647645 | 0.28 |
| GCTGATCACCTGACTTTGCA | 87647633 | 87647655 | 1.41 |
| GCCTAGTGTCCCTGCAAAGTC | 87647641 | 87647663 | 0.01 |
| GTTAATTTTAGTTGGACATAA | 87647670 | 87647692 | 0.04 |
| GTTAAAATGCTTAATTTTAGT | 87647679 | 87647701 | 0.01 |
| GAAATTAAGCATTTTAACATT | 87647686 | 87647708 | 0.04 |
| GCATTTTAACATTTGGTGCT | 87647693 | 87647715 | 0.02 |

| | | | |
|---|---|---|---|
| GACATTTGGTGCTGGGGTTCG | 87647701 | 87647723 | 0.00 |
| GGTTCGTGGCTCACACCTGC | 87647715 | 87647737 | 0.00 |
| GCTTCTGAGGGTTAGGCCTGC | 87647730 | 87647752 | 0.01 |
| GCTCAGCCTTCTGAGGGTT | 87647738 | 87647759 | 0.01 |
| GTTGCTGAGCTCAGCCTTCTG | 87647744 | 87647766 | 0.01 |
| GAGAGTTTCAAGTTGCCTT | 87647774 | 87647795 | 0.02 |
| GTTCCTTTTGGTAGAAGCCTA | 87647789 | 87647811 | 0.01 |
| GATGAAAATATGTTTTCCTTT | 87647802 | 87647824 | 0.01 |
| GCAAAAAATAGAAATAAGCT | 87647842 | 87647864 | 0.03 |
| GTATGATGTGTAAAATAAAAG | 87647869 | 87647891 | 0.01 |
| GAATTCTTACAATTTGATG | 87647913 | 87647934 | 0.76 |
| GTGGCTGTCATTCTTTCTCT | 87647938 | 87647960 | 0.01 |
| GAAAGAATGACAGCCACTGGC | 87647945 | 87647967 | 3.38 |
| GACAGCCACTGGCAGGTAG | 87647952 | 87647973 | 0.84 |
| GACAAGGCCTCTACCTGCCAG | 87647957 | 87647979 | 0.49 |
| GCTCAACCAGGCTCGAGCACA | 87647974 | 87647996 | 0.30 |
| GTGCTCGAGCCTGGTTGAG | 87647978 | 87647999 | 0.00 |
| GACTAGGATCCCCTCAACC | 87647987 | 87648008 | 0.00 |
| GATAACTACACTTAGCTGACT | 87648002 | 87648024 | 0.01 |
| GTCAGCTAAGTGTAGTTATTA | 87648007 | 87648029 | 0.01 |
| GTAAGTGTAGTTATTATGGCC | 87648012 | 87648034 | 0.01 |
| GTTATTATGGCCAGGTGAGCG | 87648021 | 87648043 | 5.30 |
| GCCAGGTGAGCGTGGCCAAC | 87648029 | 87648051 | 0.06 |
| GAAGAGAAGGCCTGCCAGT | 87648044 | 87648065 | 0.06 |
| GCAGAGGGAATTGAGAAGAGA | 87648056 | 87648078 | 0.03 |
| GACAGTTTTTGACACGGCAGA | 87648072 | 87648094 | 0.04 |
| GTAAACTAACAGTTTTTGACA | 87648079 | 87648101 | 0.01 |
| GCAAAAACTGTTAGTTTAGCC | 87648085 | 87648107 | 0.03 |
| GCTGTTAGTTTAGCCAGGCAG | 87648091 | 87648113 | 0.01 |
| GATTCCTGAAACTAGCCCCCA | 87648289 | 87648311 | 0.01 |
| GAAAAAAGGAACAGGCCTTGG | 87648303 | 87648325 | 0.05 |
| GTAGCTACATAATTCTTCCAA | 87648343 | 87648365 | 0.09 |
| GAAAAAAATGCTAAGAGTTGA | 87648373 | 87648395 | 0.01 |
| GAGGGTGTCAGATCTTCTTA | 87648461 | 87648483 | 0.07 |
| GTACCGACTGTTCTTCTGA | 87648528 | 87648549 | 0.02 |
| GTAGGCCTTGGCCCAGTACAA | 87648709 | 87648731 | 0.00 |
| GGTCAAATAGGAATAGGCCT | 87648722 | 87648744 | 0.01 |
| GAAAATGGTCAAATAGGAAT | 87648728 | 87648750 | 0.01 |
| GAAGTGAGAAAATGGTCAAAT | 87648734 | 87648756 | 0.02 |
| GCAAGGTGGCAAGTGAGAAAA | 87648743 | 87648765 | 0.10 |
| GTTCTCACTTGCCACCTTGTG | 87648748 | 87648770 | 0.86 |
| GACTTGCCACCTTGTGAGGTT | 87648753 | 87648775 | 0.16 |
| GAAAATCCTAACCTCACAAGG | 87648758 | 87648780 | 0.64 |
| GCAAAGCTAGACACAATGGCA | 87648802 | 87648824 | 0.01 |
| GCTAGACACAATGGCAAGG | 87648806 | 87648827 | 2.20 |
| GACACAATGGCAAGGTGGCTG | 87648811 | 87648833 | 0.24 |
| GCAGGAGAATTGTGGCATCA | 87648833 | 87648855 | 0.01 |
| GAAGATAAGACTATGCTATGT | 87648867 | 87648889 | 0.74 |
| GCTTTTTTTGATTGTTTTTTG | 87648887 | 87648909 | 0.00 |
| GATTCTACTAAGCCGGGCAG | 87649029 | 87649051 | 0.01 |
| GAAGATAGGATTCTACTAAGC | 87649036 | 87649058 | 0.00 |
| GGTTGTTGCTGTTTTAAGAT | 87649051 | 87649073 | 0.35 |

| | | | |
|---|---|---|---|
| GCAATCTTATATTGAGGGAAG | 87649072 | 87649094 | 0.01 |
| GTACTACCAATCTTATATTGA | 87649078 | 87649100 | 0.26 |
| GATATAAGATTGGTAGTAAGT | 87649084 | 87649106 | 0.44 |
| GGTAGTAAGTAGGTACAGA | 87649094 | 87649115 | 0.01 |
| GTACAGATGGCTTATAGGAG | 87649106 | 87649128 | 0.01 |
| GTTATAGGAGAGGATCTTCAG | 87649117 | 87649139 | 0.09 |
| GAAACCCAAGTAGAAGCTCG | 87649174 | 87649196 | 0.36 |
| GTCTTCCCCGAGCTTCTACT | 87649179 | 87649201 | 0.51 |
| GAAGCTCGGGGAAGACGCT | 87649186 | 87649207 | 0.01 |
| GACTTTCAATCCCCGTGCTCA | 87649220 | 87649242 | 0.01 |
| GCCTCTCTCTCCTTGAGCACG | 87649229 | 87649251 | 1.61 |
| GCAAGGAGAGAGAGGATAGCT | 87649238 | 87649260 | 0.02 |
| GAGGATAGCTCGGGCCCACC | 87649248 | 87649270 | 0.05 |
| GACTCACTATGGAAACCAGGT | 87649262 | 87649284 | 0.01 |
| GATACATAGCCCTAGCTGGCC | 87649286 | 87649308 | 0.17 |
| GCTTGCATACATAGCCCTAGC | 87649291 | 87649313 | 0.02 |
| GTCATGTGTTGATGTCTGTG | 87649318 | 87649340 | 0.01 |
| GTCTGTGAGGATAAACGAG | 87649331 | 87649352 | 0.01 |
| GTGTGAGTATTCATTATCTG | 87649356 | 87649378 | 0.01 |
| GATTCATTATCTGAGGATGAG | 87649364 | 87649386 | 0.00 |
| GAGTGGTAAGTGAATGTGTG | 87649381 | 87649403 | 0.01 |
| GAAAAATTGTTAACTAAACGT | 87649411 | 87649433 | 0.07 |
| GTAACAATTTTTGTTTCAGCA | 87649423 | 87649445 | 0.01 |
| GTTGTTTCAGCAAGGTACCTC | 87649432 | 87649454 | 2.53 |
| GAACAGCTCTATTAGTACCAG | 87649448 | 87649470 | 0.01 |
| GAATAGAGCTGTTCAGAGAAT | 87649459 | 87649481 | 0.01 |
| GTTCAGAGAATTGGTACCGT | 87649468 | 87649490 | 2.91 |
| GTGTCTAAAAACTGGTCCCA | 87649484 | 87649506 | 0.01 |
| GTACCAGTTTTTTTTTTTTT | 87649511 | 87649533 | 0.01 |
| GAAACTGGTACACACGCACTT | 87649524 | 87649546 | 0.01 |
| GTTACTGTTAATATTCAAAAG | 87649575 | 87649597 | 0.07 |
| GACAGTAATGGTTTTTCTTTC | 87649591 | 87649613 | 0.01 |
| GTCTTTCAGGTTATATCTACA | 87649605 | 87649627 | 0.01 |
| GAAATCAGCAAAATATGAGA | 87649630 | 87649652 | 0.01 |
| GCAAAATATGAGAAGGCTG | 87649637 | 87649658 | 0.23 |
| GTATGAGAAGGCTGAGGTAAG | 87649643 | 87649665 | 0.02 |
| GGCTGAGGTAAGTGGATAAA | 87649651 | 87649673 | 0.01 |
| GATGCTGAGTGTCTTATTCTG | 87649676 | 87649698 | 0.73 |
| GCAGTGCCCCTTTCAAGACAC | 87649702 | 87649724 | 0.02 |
| GTAACTGCCAGTGTCTTGAA | 87649709 | 87649731 | 1.71 |
| GTTACACTGAAGTCCTGCACA | 87649728 | 87649750 | 0.03 |
| GCCTAAGAGTCTACCGTGTGC | 87649740 | 87649762 | 1.67 |
| GACTCTTAGGAACAGTCTTG | 87649753 | 87649775 | 0.76 |
| GCTTGGGGTGAGTTCTGTGAC | 87649769 | 87649791 | 0.01 |
| GAAACAGAAAGTAGTATGAAG | 87649792 | 87649814 | 0.01 |
| GGCTTCTGCGCCACTTGTCA | 87649815 | 87649837 | 0.01 |
| GACAAGTGGCGCAGAAGCCTC | 87649820 | 87649842 | 0.01 |
| GAAGCCTCTGGCTTTAGATTT | 87649833 | 87649855 | 0.02 |
| GCTTTAGATTTTGGAGTCTTG | 87649843 | 87649865 | 0.01 |
| GGGTTTCTCTAGAATATACA | 87649884 | 87649906 | 0.02 |
| GATCAGGTCAGCACTCATACT | 87649904 | 87649926 | 0.24 |
| GTTGTAGAGACACGGGAAATC | 87649921 | 87649943 | 0.14 |

| | | | |
|---|---|---|---|
| GTTATAAACTTGTAGAGACAC | 87649929 | 87649951 | 0.38 |
| GTTTATAATAATCGTTTGAGT | 87649945 | 87649967 | 0.02 |
| GTAATCGTTTGAGTTGGGCAT | 87649952 | 87649974 | 2.81 |
| GACCTTTAACCCCCCAGTACT | 87649981 | 87650003 | 0.00 |
| GTTTGTCTCCCTAGTACTGGG | 87649989 | 87650011 | 0.62 |
| GTAGGGAGACAAAGGCCAGCT | 87650000 | 87650022 | 0.01 |
| GAAATAGCAGGGCTATGTAAA | 87650073 | 87650095 | 0.01 |
| GCTGGATCTAAATAAATAGCA | 87650085 | 87650107 | 0.03 |
| GGGTCCCTTTACATAGCCC | 87650107 | 87650128 | 0.05 |
| GCTCTAAGTTTGCGGCCAGCC | 87650153 | 87650175 | 0.01 |
| GAAGATGGTTCTCTAAGTTTG | 87650162 | 87650184 | 0.23 |
| GTTACGTTTTCAGCTGGATG | 87650224 | 87650246 | 0.25 |
| GCTTAAAGTTACGTTTTCAGC | 87650230 | 87650252 | 0.02 |
| GTTTAAGACCCCCAAACATTG | 87650247 | 87650269 | 0.23 |
| GACCCCCAAACATTGAGGGGG | 87650253 | 87650275 | 0.02 |
| GAAACATTGAGGGGGTGGTGT | 87650259 | 87650281 | 0.01 |
| GCCTTTAATCCCCAGCGCTC | 87650287 | 87650309 | 0.14 |
| GCTTCTGCATCCTGAGCGCT | 87650297 | 87650319 | 0.02 |
| GCAGGGGATCGCTGAGTTCA | 87650318 | 87650340 | 0.01 |
| GACTCAGCCAGTAGACCAAGC | 87650341 | 87650363 | 1.51 |
| GTTGGTCTACTGGCTGAGTTC | 87650346 | 87650368 | 0.35 |
| GGCTGAGTTCTGGGACAGC | 87650356 | 87650377 | 0.02 |
| GCTTAGAAGCAACAAAGAAC | 87650374 | 87650396 | 0.03 |
| GCTGCAACAGAGGAGGAAACA | 87650404 | 87650426 | 0.00 |
| GTCCTCCTCTGTTGCAGATAA | 87650411 | 87650433 | 0.45 |
| GCTGTTGCAGATAAAGGAGAT | 87650418 | 87650440 | 0.02 |
| GCTAGGCATTAAGCTCTCTG | 87650447 | 87650469 | 0.04 |
| GTTCAGCACTCAGCACGGCT | 87650464 | 87650486 | 3.13 |
| GCTGAGTGCTGAACTGAGGA | 87650473 | 87650495 | 0.01 |
| GCTGAGGAGGGGCCGCCCGAC | 87650486 | 87650508 | 0.13 |
| GTTCTGCCACTTCCGGTCGGG | 87650497 | 87650519 | 0.01 |
| GCTCTTCTGCCACTTCCGGT | 87650501 | 87650523 | 1.35 |
| GGACTGCAGATGTATAAACC | 87650526 | 87650548 | 0.52 |
| GGGAAGATGAAGACCTGCGA | 87650548 | 87650570 | 8.56 |
| GTCAAAGCAGTAACCCTTCGC | 87650561 | 87650583 | 1.76 |
| GTACTCGTAAATACAAGCAGC | 87650625 | 87650647 | 0.84 |
| GAAATACAAGCAGCTGGACAC | 87650632 | 87650654 | 0.35 |
| GAATTTTACGATCGGTAATGC | 87650651 | 87650673 | 0.20 |
| GTTCGTCTAATTTTACGAT | 87650660 | 87650681 | 0.02 |
| GAAATTAGACGAACGTCTTAT | 87650669 | 87650691 | 0.02 |
| GACGAACGTCTTATAGGTGC | 87650675 | 87650697 | 1.09 |
| GTTATAGGTGCAGGTCCAGTG | 87650685 | 87650707 | 0.01 |
| GCTAAGTTCTGAGTACCACAC | 87650699 | 87650721 | 1.51 |
| GATCAACTCTCTTAACTTTGC | 87650722 | 87650744 | 0.11 |
| GAACATTCCAACAGTAGAATG | 87650772 | 87650794 | 8.72 |
| GTAGTCACCGCATTCTACTGT | 87650778 | 87650800 | 0.01 |
| GCAAGACTCAGGTATATAGAA | 87650836 | 87650858 | 0.42 |
| GTTTATTATGTACAAGACTC | 87650848 | 87650870 | 0.01 |
| GATCTTAATTATGCACATGCT | 87650932 | 87650954 | 0.01 |
| GTAATAGATGTCTAAATGCTC | 87650954 | 87650976 | 0.02 |
| GAATGCTCTGGCACTGCTAAC | 87650967 | 87650989 | 0.06 |
| GCAAACCTGTAATTCTGTACT | 87650992 | 87651014 | 0.01 |

| | | | |
|---|---|---|---|
| GATTCTGTACTTGGGAGGTAG | 87651002 | 87651024 | 0.52 |
| GTAGGAGGGTTAGCGCTTCCG | 87651025 | 87651047 | 0.18 |
| GCAGATACACAGCAGCTACCT | 87651042 | 87651064 | 0.25 |
| GTATCTGCTCTGCCACTGAC | 87651058 | 87651080 | 0.01 |
| GGATAGTCAAGGCCAGTCAG | 87651070 | 87651092 | 2.26 |
| GATAGGGTGTTGGATAGTCA | 87651081 | 87651103 | 0.07 |
| GATTTCTTTCAGATAGGGTGT | 87651091 | 87651113 | 0.01 |
| GATTTTTATTTCTTTCAGAT | 87651098 | 87651120 | 0.00 |
| GAAAATCAAACTTAAGAAACG | 87651115 | 87651137 | 0.03 |
| GTGGGTAAGTCTTGTGTTAT | 87651134 | 87651156 | 0.01 |
| GTCTTGTGTTATGGGAGGCT | 87651142 | 87651164 | 0.02 |
| GGGAGGCTAGGGAATGAGTG | 87651154 | 87651176 | 0.00 |
| GGGAATGAGTGTGGTTTTGA | 87651163 | 87651185 | 0.03 |
| GTGGTTTTGAAGGCAGCCC | 87651173 | 87651194 | 0.05 |
| GACTCTGTGTCTACATAGCCT | 87651189 | 87651211 | 0.05 |
| GCTATGTAGACACAGAGTTCC | 87651195 | 87651217 | 0.14 |
| GCAGGGTCTCGCTGGGTAGCC | 87651213 | 87651235 | 0.01 |
| GCTGATGGTCAGGGTCTCGCT | 87651221 | 87651243 | 0.01 |
| GCAATATGTTACTGATGGTCA | 87651231 | 87651253 | 0.01 |
| GCTCTCACAATATGTTACTGA | 87651237 | 87651259 | 0.01 |
| GTAACATATTGTGAGAGTTG | 87651243 | 87651265 | 0.41 |
| GTGAGAGTTGGGGTAGTGTG | 87651253 | 87651275 | 0.01 |
| GGTAGTGTGGGGTGGTTGGG | 87651264 | 87651286 | 0.41 |
| GGTTGGGAGGGTTGTTCAG | 87651277 | 87651298 | 0.23 |
| GCTATGTGCTTACCATGTGAG | 87651301 | 87651323 | 0.00 |
| GGACACAGGGCCTCTCACA | 87651312 | 87651333 | 0.01 |
| GGTAGTGCAAATTGGACACA | 87651324 | 87651346 | 0.01 |
| GAAGACACTGGTAGTGCAAAT | 87651332 | 87651354 | 0.82 |
| GCACTACCAGTGTCTTAGTC | 87651339 | 87651361 | 0.01 |
| GATAGAGGGATGTTCTTTAAC | 87651510 | 87651532 | 0.01 |
| GTTTAACTGGCTTGCTTCCCC | 87651524 | 87651546 | 0.04 |
| GTTTCCCGGCTGTACTGGC | 87651732 | 87651753 | 0.01 |
| GATAATACAAAGAGTGTTTCC | 87651745 | 87651767 | 0.58 |
| GATTATACCCTTAGTACAAAC | 87651763 | 87651785 | 0.02 |
| GAAAGCTCCAGTTTGTACTAA | 87651769 | 87651791 | 0.02 |
| GTGAGTACTTAATTCATAC | 87651794 | 87651815 | 0.53 |
| GCCCTTCATGTAGAACTTCA | 87651826 | 87651848 | 0.01 |
| GCTTTCTGTGTGTGCAGTCAC | 87651856 | 87651878 | 0.42 |
| GCTGCACACACAGAAAGTGTC | 87651863 | 87651885 | 0.01 |
| GCACACTGCATCTGGAGTCAG | 87651901 | 87651923 | 0.88 |
| GCAGCGGCTCACACTGCATC | 87651910 | 87651932 | 0.28 |
| GTGAGCCGCTGCCACCCCAC | 87651921 | 87651943 | 0.10 |
| GCCATCCCGGTGGGGTGGCAG | 87651926 | 87651948 | 0.01 |
| GCCAGAAGCCCATCCCGGTG | 87651935 | 87651957 | 0.43 |
| GAAGGGCCAGAAGCCCATCC | 87651940 | 87651962 | 1.17 |
| GAGAGCAGCTTACAGAGGGA | 87651958 | 87651980 | 0.07 |
| GCTGAGGAGAGCAGCTTACAG | 87651963 | 87651985 | 0.88 |
| GTCCTCAGCATCGTAGCAACG | 87651979 | 87652001 | 0.00 |
| GCACTTGTGTGTTAATGCTAC | 87652013 | 87652035 | 0.02 |
| GCTATGAACACAGCGACAG | 87652072 | 87652093 | 0.17 |
| GTATGCTGTGCGATACAGCTG | 87652100 | 87652122 | 3.68 |
| GTAAGTGATAGTAGCCTTTAC | 87652126 | 87652148 | 0.34 |

| | | | |
|---|---|---|---|
| GTAGCCTTTACAGGAAAAGT | 87652135 | 87652157 | 0.01 |
| GTTGGCTGACTTCTAAGATCC | 87652154 | 87652176 | 2.72 |
| GCTTCTAAGATCCTGGCAAAG | 87652162 | 87652184 | 0.00 |
| GGTCTGATTTTAAAGGGGGG | 87652198 | 87652220 | 0.02 |
| GATCGAGGTCTGATTTTAAA | 87652204 | 87652226 | 0.01 |
| GAAGCGGGATTGACCCGATCG | 87652219 | 87652241 | 0.01 |
| GGGTCAATCCCGCTTGCTC | 87652227 | 87652248 | 0.01 |
| GCACTGACAGCTCTTATGA | 87652397 | 87652418 | 0.61 |
| GCTGAGCTTGGGCTGGTGAGA | 87652433 | 87652455 | 0.03 |
| GAAGAATGCTGAGCTTGGGC | 87652441 | 87652463 | 0.01 |
| GAAGAAGAAGAATGCTGAGCT | 87652446 | 87652468 | 0.03 |
| GCATTCTTCTTCTTTTTTT | 87652455 | 87652476 | 0.01 |
| GAGCTTGGAGCTGGGTGTGG | 87652603 | 87652625 | 0.03 |
| GAAGAATGCTGAGCTTGGAGC | 87652612 | 87652634 | 0.01 |
| GAGTTAAGAATGCTGAGCT | 87652618 | 87652639 | 0.05 |
| GATCATCTAATTACAAAATAT | 87652641 | 87652663 | 0.03 |
| GAAAAACAGCTGTGACCATTG | 87652684 | 87652706 | 0.01 |
| GGGGGTAGCAGGACCACAA | 87652698 | 87652719 | 0.03 |
| GGTCCTGCTACCCCCTTTAT | 87652705 | 87652727 | 0.39 |
| GATCACTGTATCCAATAAAGG | 87652715 | 87652737 | 0.01 |
| GACACCCCGCAAAGATTAGAA | 87652760 | 87652782 | 0.04 |
| GATTAGAAAGGAGATTTCAG | 87652772 | 87652794 | 0.06 |
| GAAATTTATTTGTTATCAAAC | 87652795 | 87652817 | 0.45 |
| GATTTGTTATCAAACAGGCTC | 87652801 | 87652823 | 0.16 |
| GCTCCGGATGAACTGTTTCA | 87652817 | 87652839 | 0.01 |
| GAACTGTTTCAAGGAGTCTG | 87652826 | 87652848 | 0.01 |
| GTTTCAAGGAGTCTGTGGAT | 87652831 | 87652853 | 0.01 |
| GTTCAGAGAAACCCCTCAAGC | 87652880 | 87652902 | 0.02 |
| GAAAAGAAACCCCTGCTTGAG | 87652890 | 87652912 | 0.01 |
| GAACGCTAATGAAGCTCACTG | 87652934 | 87652956 | 0.00 |
| GAAGACGAATATAATATATCC | 87652989 | 87653011 | 0.03 |
| GCCAAACACTGATTGTATGA | 87653027 | 87653049 | 0.02 |
| GATACAATCAGTGTTTGGCTA | 87653032 | 87653054 | 0.01 |
| GTTGTACTGGAGTCCCTTGCA | 87653061 | 87653083 | 0.00 |
| GTGAAAGCATTACTTGTAC | 87653075 | 87653096 | 0.61 |
| GTTCACAGCGCCAAAGTTATA | 87653092 | 87653114 | 0.00 |
| GTAAAATATTCCTTATAACTT | 87653101 | 87653123 | 0.01 |
| GAATTGCTCCCCTGGAATC | 87653180 | 87653201 | 0.01 |
| GCAGTGAGGCCAGATTCCAG | 87653188 | 87653210 | 0.07 |
| GAATCTGGCCTCACTGCAGG | 87653194 | 87653216 | 0.03 |
| GTTCAGAGCCTACTCAGTG | 87653262 | 87653283 | 2.20 |
| GCCACACAAACCTCACTGAGT | 87653270 | 87653292 | 0.11 |
| GGTTTGTGTGGACCCTGCCT | 87653282 | 87653304 | 0.01 |
| GCCTCAAAATTTCCCTAGGCA | 87653294 | 87653316 | 0.48 |
| GAGGCCCTCAAAATTTCCCT | 87653299 | 87653321 | 0.00 |
| GAGGGCCTCACCTTGCCTCC | 87653313 | 87653335 | 0.21 |
| GAATTCCCTGGAGGCAAGGTG | 87653318 | 87653340 | 1.33 |
| GACTTTCTTTTGAACAATACC | 87653376 | 87653398 | 0.00 |
| GAGTCTGTGAAACTTGTAG | 87653427 | 87653448 | 0.32 |
| GATAATAATTCTATATAGAGT | 87653466 | 87653488 | 0.24 |
| GAATTATTATGGATTTGTAA | 87653479 | 87653501 | 0.03 |
| GATCATTGAAAGACTTTTGCC | 87653575 | 87653597 | 0.01 |

| | | | |
|---|---|---|---|
| GAAAGACTTTTGCCTGGGC | 87653581 | 87653602 | 0.02 |
| GAAAAAAAAAAAGGCGAGCAG | 87653780 | 87653802 | 0.01 |
| GAGGACAGCCTGGTCTACTA | 87653861 | 87653883 | 0.01 |
| GACAAAAAAGATTTTGCCTTT | 87653933 | 87653955 | 0.00 |
| GCCTTTAGGAATAGCTAGTA | 87653947 | 87653969 | 0.83 |
| GGAATAGCTAGTAGGGGGC | 87653954 | 87653975 | 0.74 |
| GCTAGTAGGGGGCTGGTGAGA | 87653961 | 87653983 | 0.02 |
| GGGATTGAACTAAGGACCTT | 87654014 | 87654036 | 0.05 |
| GGTTGCTGGGATTGAACTA | 87654022 | 87654043 | 0.04 |
| GTTGTCATTTTACATAATGTA | 87654172 | 87654194 | 0.01 |
| GTGGTAAATTTCCCTTCAGA | 87654283 | 87654305 | 0.01 |
| GGGTAACAGGAAAGGTTTG | 87654302 | 87654323 | 0.02 |
| GCTCGTTGGGTAACAGGAA | 87654309 | 87654330 | 0.00 |
| GTTTAAAGCTCGTTGGGTAAC | 87654314 | 87654336 | 0.01 |
| GCAAATGTTTTAAAGCTCGTT | 87654321 | 87654343 | 0.02 |
| GTTGTCCTAGGATATAACTG | 87654400 | 87654422 | 0.29 |
| GTTCTATCTCACAGTTGTCCT | 87654412 | 87654434 | 0.00 |
| GCTCACCTTAAGCTCACCAG | 87654511 | 87654533 | 0.00 |
| GTCCTCCTCTGGTGAGCTTA | 87654516 | 87654538 | 0.01 |
| GCAAAGGTTTAAGTCCTCCTC | 87654527 | 87654549 | 0.91 |
| GGGGGGGATGCAAAATAAC | 87654564 | 87654585 | 0.03 |
| GATGCAAAATAACTGGCTAG | 87654570 | 87654592 | 0.57 |
| GCTAGAGGCAGATAGCTTTGC | 87654586 | 87654608 | 0.01 |
| GATAGCTTTGCAGGAAGTAG | 87654595 | 87654617 | 0.01 |
| GTTGTTATCCATTGAAACCAA | 87654634 | 87654656 | 0.01 |
| GTTGGTTACCATTGGTTTCAA | 87654641 | 87654663 | 3.35 |
| GCTAATGTTTGGTTACCAT | 87654650 | 87654671 | 0.93 |
| GTAACATAAGCAGCTAATGTT | 87654660 | 87654682 | 0.01 |
| GTGCGCCCACAGCAATTCTT | 87654697 | 87654719 | 0.05 |
| GTCCTAATTATGAAGCTGTTG | 87654777 | 87654799 | 0.01 |
| GGTGTTAATGAAAAAATTGT | 87654798 | 87654820 | 0.01 |
| GATAGAATCATGAAAGCATT | 87654820 | 87654842 | 0.04 |
| GTCTTAAAAACCTGATGAGC | 87654850 | 87654872 | 3.86 |
| GAAACCTGATGAGCTGGGCAG | 87654857 | 87654879 | 0.01 |
| GCACGGAATAGATGTCTCGTC | 87655046 | 87655068 | 0.43 |
| GTTTCAAACAGCAGTTACAT | 87655068 | 87655090 | 0.03 |
| GTATTGCATGGTAAACCTTTT | 87655117 | 87655139 | 0.01 |
| GAATTATCAATTTTGCCTAAA | 87655131 | 87655153 | 0.01 |
| GAATTGATAATTATTATCCAA | 87655143 | 87655165 | 0.02 |
| GATGCATACATAGACTTAC | 87655207 | 87655228 | 0.01 |
| GATGTACTTCTCTCCCATAGA | 87655264 | 87655286 | 0.01 |
| GGGAGAGAAGTACATGTGAT | 87655272 | 87655294 | 0.02 |
| GCATGAGTTTCCTTCTACTTC | 87655311 | 87655333 | 0.01 |
| GATTATTTGGCCTGAAGTAGA | 87655320 | 87655342 | 0.14 |
| GCAGCACATAGTTCGATTATT | 87655334 | 87655356 | 0.02 |
| GTTTTTAAAGTGCTAAAATC | 87655365 | 87655387 | 0.01 |
| GCTAAAATCAGGCTGGAGAGA | 87655377 | 87655399 | 0.01 |
| GTCTGAAAGACCCACCAAGTG | 87655401 | 87655423 | 0.34 |
| GTGGGAGAGTCCTCACTTGG | 87655411 | 87655433 | 0.01 |
| GATACCCCCCAAATCACTCAC | 87655454 | 87655476 | 0.02 |
| GCCTTGATGCAAACCGCTAG | 87655482 | 87655504 | 0.02 |
| GAGGACTTTTTATTCCAGGC | 87655501 | 87655523 | 0.02 |

| | | | |
|---|---|---|---|
| GTTTTTATTCCAGGCGGGCTC | 87655507 | 87655529 | 0.86 |
| GTGGGCCCCAGAGCCCGCC | 87655515 | 87655536 | 0.01 |
| GGGGCCCACAGTCATACACC | 87655528 | 87655550 | 0.02 |
| GCACAGTCCTCTACCCCTGCC | 87655546 | 87655568 | 0.00 |
| GTGGAACCCCGAGTAGCTGG | 87655566 | 87655588 | 0.02 |
| GAACCCCGAGTAGCTGGGGG | 87655569 | 87655591 | 0.01 |
| GCTGGGGGAGGGGGTATTTAA | 87655582 | 87655604 | 0.12 |
| GGAGGGCGTTATTGGAAAAT | 87655631 | 87655653 | 0.02 |
| GATGAGGAAGTGGAAGTCACA | 87655674 | 87655696 | 0.28 |
| GACATTTGCATAGCAGGTTTC | 87655760 | 87655782 | 0.01 |
| GCATAGCAGGTTTCAGGAA | 87655766 | 87655787 | 0.01 |
| GCAGGTTTCAGGAAAGGTCA | 87655771 | 87655793 | 0.57 |
| GAAAAACGGAAAAACGAAAAA | 87655797 | 87655819 | 0.02 |
| GAACCCAGGAAGCAGGTTGG | 87655858 | 87655880 | 0.02 |
| GTAAAAAATAATAAAAGACAA | 87656175 | 87656197 | 0.06 |
| GACATGTGGGCGCACACGCAC | 87656276 | 87656298 | 0.02 |
| GTGCGCCCACATGTGTATCA | 87656285 | 87656307 | 0.01 |
| GTAAGCCCTGATACACATGT | 87656290 | 87656312 | 0.04 |
| GTACTGTTCCTTAAAAGAC | 87656395 | 87656416 | 0.08 |
| GTCTAAAGCCAGTCTTTTA | 87656403 | 87656424 | 0.00 |
| GACGTTTAAGAACTCATCC | 87656422 | 87656443 | 0.02 |
| GGGTCTTGTTCAGGGAGTCC | 87656439 | 87656461 | 0.01 |
| GCCTTAGGGGGTCTTGTTCA | 87656447 | 87656469 | 0.01 |
| GATCTGCTGTGGCATTGCCTT | 87656462 | 87656484 | 0.04 |
| GCCTGGGATAAAAATCTGCTG | 87656474 | 87656496 | 0.02 |
| GTAAGGCTTATACTCTACCT | 87656491 | 87656513 | 0.02 |
| GAAGCCTTACACGAGAACAAT | 87656505 | 87656527 | 0.00 |
| GTTACATCATCAAAATAGTTA | 87656545 | 87656567 | 0.01 |
| GTATGGCTTGAGATGATAATT | 87656563 | 87656585 | 0.03 |
| GGACACTGAGAACACTGTG | 87656635 | 87656656 | 0.08 |
| GTTCTACCTGTACCACATAA | 87656659 | 87656681 | 0.07 |
| GCTTAACCAATTATTGCAAA | 87656703 | 87656725 | 0.01 |
| GCTAGCTGCAACTACTTTAAC | 87656794 | 87656816 | 0.14 |
| GAATGCTTGACTGTAATAGCC | 87656902 | 87656924 | 0.00 |
| GAATCCTCAAAGACAAGGTAT | 87656977 | 87656999 | 0.01 |
| GTTTTCTTTCAAAAACAAATA | 87657017 | 87657039 | 0.02 |
| GGGGGAATTATACCCTTCA | 87657038 | 87657059 | 0.52 |
| GTGCTTTTGGTGACCTTGAA | 87657050 | 87657072 | 0.00 |
| GACAAAGTCAAGATGTGCTTT | 87657063 | 87657085 | 0.01 |
| GTTAAAAAAAAATTTTTTGTG | 87657091 | 87657113 | 0.02 |
| GAAAAAATTTTTTGTGTGGGG | 87657096 | 87657118 | 0.02 |
| GTTTTTTGTGTGGGGCGGTGG | 87657102 | 87657124 | 0.00 |
| GGGTTTCTCTGTTTCAGCC | 87657217 | 87657238 | 0.02 |
| GTTTTTGCAAACTTACACTAA | 87657264 | 87657286 | 0.02 |
| GCCTGCAGCGCATTGTCAC | 87657290 | 87657311 | 0.01 |
| GGGATTCCACCATTTAACCA | 87657337 | 87657359 | 0.03 |
| GGAATCCCGTAACTAATAAG | 87657352 | 87657374 | 0.16 |
| GTCATTCCACTTATTAGTTAC | 87657357 | 87657379 | 0.02 |
| GATCCAGATCCAGTTTCAATA | 87657379 | 87657401 | 0.01 |
| GCCTCTCCCCCATATTGAAAC | 87657387 | 87657409 | 0.00 |
| GCAATCTTTCAAGCAACCATC | 87657445 | 87657467 | 0.02 |
| GCATACTTACTAGAAGGATTC | 87657488 | 87657510 | 0.01 |

| | | | |
|---|---|---|---|
| GTCTTTAGCCATCTTCATAAT | 87657514 | 87657536 | 0.01 |
| GATGATTCAAACTAACTCAGC | 87657547 | 87657569 | 0.00 |
| GTTACTTCTCAAGTGTAGTCG | 87657572 | 87657594 | 0.45 |
| GGCCTCCTGGTGTGGGGAGG | 87657619 | 87657641 | 0.01 |
| GCAATGATCAGACACAAGGCC | 87657640 | 87657662 | 0.01 |
| GTACAACTGAATAGAGTGCAC | 87657669 | 87657691 | 0.01 |
| GGTTGTATGCAGGGAATGTC | 87657688 | 87657710 | 2.32 |
| GACGGGATTAAGGTTGTATGC | 87657698 | 87657720 | 0.01 |
| GTTAAGAGCAACGGGATTA | 87657709 | 87657730 | 0.37 |
| GAAGTCAAAAGTTAAGAGCAA | 87657717 | 87657739 | 0.74 |
| GCAGATCTGTTACGTTCATAC | 87657751 | 87657773 | 0.01 |
| GATACAGGCTCCCTTCAGTCA | 87657767 | 87657789 | 0.03 |
| GAATAACTGCCATGACTGAA | 87657776 | 87657798 | 1.59 |
| GACCACTATGAAGAGATGCAC | 87657799 | 87657821 | 0.01 |
| GATGCACTGGGTGCATATTA | 87657812 | 87657834 | 0.00 |
| GTATTATGGCTTTAGTCTGAA | 87657827 | 87657849 | 0.01 |
| GCTTTAGTCTGAATGGGAAAA | 87657835 | 87657857 | 0.21 |
| GGGAAAAAGGTAAGCTATGA | 87657848 | 87657870 | 0.01 |
| GAGGGTTGGCAGTCAAAGCC | 87657866 | 87657888 | 4.39 |
| GTCATAGAACATCAGTTGCC | 87657884 | 87657906 | 0.03 |
| GACTCTGAAAGATCCCCGTTT | 87657905 | 87657927 | 0.02 |
| GCAATGTTCTCCCCAAAACG | 87657917 | 87657939 | 0.08 |
| GAGAACATTGCTCAAGTCCC | 87657929 | 87657951 | 0.37 |
| GTCAGGGTGTTGGCTCATCCT | 87657946 | 87657968 | 0.01 |
| GCTCTCGAGTTATCAGGGTGT | 87657957 | 87657979 | 0.01 |
| GACGGTTTCTCTCGAGTTATC | 87657964 | 87657986 | 0.01 |
| GATTCGATATTGCATCAAGAA | 87657983 | 87658005 | 0.02 |
| GATTTGAAAATCAGCATGCTG | 87658013 | 87658035 | 0.01 |
| GCCGAGCTCGTAACCCTCAC | 87658035 | 87658057 | 0.22 |
| GATCTTCTCTGCCCCTGTGA | 87658048 | 87658070 | 0.01 |
| GCCGTCGACTGCAACTGCTGG | 87658073 | 87658095 | 0.48 |
| GAAAGAATAAACCATAAATTG | 87658101 | 87658123 | 0.02 |
| GATAAACCATAAATTGAGGGG | 87658106 | 87658128 | 0.42 |
| GCCTATCCTCCCCTCAATTTA | 87658111 | 87658133 | 0.15 |
| GAGGATAGGACCACCAAGAA | 87658125 | 87658147 | 0.01 |
| GCTCAGCTTCCCCATTCTTGG | 87658135 | 87658157 | 0.01 |
| GACTATTTACTAGAAGGAGT | 87658288 | 87658310 | 0.13 |
| GATTAGGACTATTTACTAGA | 87658294 | 87658316 | 0.02 |
| GATTTTTCTAGTTCTATGATT | 87658310 | 87658332 | 0.01 |
| GTACATGCTGTAATGAGTCAG | 87658337 | 87658359 | 0.07 |
| GTTGCTTCTTTCATTTACTAT | 87658462 | 87658484 | 0.01 |
| GAAAGAAGCAAAACTAATAA | 87658474 | 87658496 | 0.01 |
| GCAAAACTAATAAAGGGCCC | 87658481 | 87658503 | 0.02 |
| GCCCAGGAGGGCAGATAGTA | 87658497 | 87658519 | 0.04 |
| GCTAATGGAGGATGGCTTGA | 87658549 | 87658571 | 0.00 |
| GAACAAGAAGCTAATGGAGGA | 87658557 | 87658579 | 0.03 |
| GAAACAAGAACAAGAAGCTAA | 87658564 | 87658586 | 0.00 |
| GTTTTAACCTTTCTCTGAGTT | 87658592 | 87658614 | 0.01 |
| GGGACAATTAGAGACTCTA | 87658615 | 87658636 | 0.02 |
| GTCTCTAATTGTCCCTGAA | 87658622 | 87658643 | 0.98 |
| GGACTGTGTGCAGCCCTAA | 87658666 | 87658687 | 0.03 |
| GCTGCACACAGTCCTCCTTTG | 87658675 | 87658697 | 2.19 |

| | | | |
|---|---|---|---|
| GATTTATTATTCCTCAAAGG | 87658686 | 87658708 | 0.11 |
| GCCAAAGTAGTCTTACAGAAT | 87658718 | 87658740 | 0.03 |
| GTAAGACTACTTTGGAGAGCG | 87658727 | 87658749 | 0.00 |
| GAAAAAAGAAAGTAGACATTA | 87658785 | 87658807 | 0.02 |
| GTAGACATTGCCAGGCGTGG | 87658948 | 87658970 | 0.93 |
| GCAATGAAAGTAGACATTGCC | 87658956 | 87658978 | 0.03 |
| GACAAGGTCTAGAAGGACTGT | 87658982 | 87659004 | 0.13 |
| GGGTCCAGTTACTAAGGCAG | 87659016 | 87659038 | 0.00 |
| GCTAGCAGCTGGAATTGGTAC | 87659041 | 87659063 | 0.01 |
| GTTGGGACTAGCAGCTGGAAT | 87659047 | 87659069 | 1.12 |
| GATGAGTTTGGGACTAGCAGC | 87659053 | 87659075 | 0.01 |
| GCTCGCAATGGTCATGAGTTT | 87659065 | 87659087 | 0.01 |
| GTCATGACCATTGCGAGGGTG | 87659072 | 87659094 | 2.03 |
| GCTTAAGATTTCAAAAATAC | 87659116 | 87659138 | 0.02 |
| GTTTTGAAATCTTAAGCATCT | 87659123 | 87659145 | 0.02 |
| GCATCTAGGACCTCTTTGTC | 87659137 | 87659159 | 0.01 |
| GATAATACCACCCTGACAAAG | 87659147 | 87659169 | 0.26 |
| GTCAGGGTGGTATTATATCCT | 87659155 | 87659177 | 0.01 |
| GTTAATGCAGTTAGTCCCT | 87659172 | 87659193 | 0.01 |
| GTTGCTACTGCTGTTTTCGAT | 87659211 | 87659233 | 0.00 |
| GAAAACAGCAGTAGCAAACAC | 87659218 | 87659240 | 0.02 |
| GCAAACACAGGGAGTCAATC | 87659230 | 87659252 | 1.12 |
| GTATTGGTGGGTTTGCAGCTC | 87659249 | 87659271 | 1.74 |
| GAAACCAATATCTTTGGCC | 87659279 | 87659300 | 0.11 |
| GCTTCCAGAAACCAATATCTT | 87659284 | 87659306 | 0.01 |
| GTATTGGTTTCTGGAAGAAGC | 87659291 | 87659313 | 1.32 |
| GGAAGAAGCAGGAATAGTCC | 87659302 | 87659324 | 0.02 |
| GAAGCAGGAATAGTCCAGGTG | 87659307 | 87659329 | 0.02 |
| GACACCTCTGCAACCACACC | 87659320 | 87659342 | 0.50 |
| GCTCGCCTTAGCCGAAATTTC | 87659381 | 87659403 | 0.01 |
| GCCGCTCCTTCATAATAAGG | 87659471 | 87659493 | 0.01 |
| GCCGGGCCCCCTTATTATGA | 87659477 | 87659499 | 1.09 |
| GACCAGTAGTAGGAGGCCAGT | 87659590 | 87659612 | 1.95 |
| GCCAGCGCAGAAACCTCCAAC | 87659605 | 87659627 | 0.85 |
| GCTGGGAACGATCGTCCCCT | 87659623 | 87659645 | 0.72 |
| GAACGATCGTCCCCTAGGGT | 87659628 | 87659650 | 0.03 |
| GTCCCCTAGGGTTGGGGCTC | 87659636 | 87659658 | 0.02 |
| GGGTTGGGGCTCGGGAATCG | 87659644 | 87659666 | 0.00 |
| GCTCGGGAATCGTGGGGAGA | 87659652 | 87659674 | 0.01 |
| GGAATCGTGGGGAGAAGGGG | 87659657 | 87659679 | 0.01 |
| GGTGGTAGTACTCATCGGG | 87659678 | 87659699 | 0.01 |
| GTACTGGGCTGGGAAAAGTAC | 87659705 | 87659727 | 0.63 |
| GTCCCAGCCCAGTAAGAATAG | 87659715 | 87659737 | 0.02 |
| GTCTCAACCCTGACACAAATG | 87659744 | 87659766 | 0.26 |
| GCATTTTCCACATTTGTGTCA | 87659750 | 87659772 | 0.01 |
| GTACATGCAAGGATGGAATAA | 87659776 | 87659798 | 0.00 |
| GGCCTAAGGGGCGTACCTGG | 87659808 | 87659830 | 0.01 |
| GTAGCTAAGCAGTTTACTGAT | 87659829 | 87659851 | 0.01 |
| GTCAGTCTTGCCTACCAAGGA | 87659863 | 87659885 | 0.01 |
| GCAAGACTGAGTGACCATCT | 87659876 | 87659898 | 0.02 |
| GTGACAGTAAAAACCCAAGA | 87659890 | 87659912 | 0.01 |
| GGTTTTTACTGTCACGATGA | 87659898 | 87659920 | 0.00 |

| | | | |
|---|---|---|---|
| GTTGGAAAGAGACTGGGGGCA | 87659942 | 87659964 | 0.28 |
| GCAAGCTTGGAAAGAGACTGG | 87659947 | 87659969 | 0.01 |
| GTTGGGGATATCTCTCTAACC | 87659967 | 87659989 | 0.07 |
| GCATCGAGAAAAGTACTGGAA | 87659991 | 87660013 | 0.01 |
| GACTTTTCTCGATGGGGCTGG | 87660001 | 87660023 | 0.06 |
| GGGGCATTGAGGGATGACAA | 87660161 | 87660183 | 0.01 |
| GAGGGATGACAAGGGAGGAA | 87660169 | 87660191 | 0.01 |
| GCAAGGGAGGAATGGGTTACC | 87660178 | 87660200 | 0.00 |
| GAGCCTGACAGTATCCAGGC | 87660421 | 87660443 | 0.02 |
| GTTAGAGCCTGACAGTATCC | 87660425 | 87660447 | 0.01 |
| GGCTCTAACCTGGATGATAA | 87660439 | 87660461 | 0.12 |
| GACTAATAACCTCCTATCAG | 87660557 | 87660579 | 0.23 |
| GCAAGAAAACCATAGTGATTG | 87660607 | 87660629 | 0.03 |
| GTCGGTCCTGAAAGAGCCACT | 87660642 | 87660664 | 0.03 |
| GATAGTGGAGCAGAATTTT | 87660661 | 87660682 | 0.01 |
| GATTCTGCTCCACTATCAACA | 87660667 | 87660689 | 0.03 |
| GATGGATTTCCTTGTTGATAG | 87660675 | 87660697 | 0.02 |
| GAAGTGTGGAGGAGACCCCTA | 87660694 | 87660716 | 0.00 |
| GCCTAGGGTAACCCTAAGTG | 87660709 | 87660731 | 0.03 |
| GCTTAGGGTTACCCTAGGCTC | 87660714 | 87660736 | 0.01 |
| GATACCCCCTCCCTGAGCCTA | 87660724 | 87660746 | 0.05 |
| GATGAGGATTAGGGGAGACA | 87660750 | 87660772 | 3.15 |
| GTCCCCTAATCCTCATCCTCT | 87660757 | 87660779 | 1.31 |
| GATTCTCACCCTAGAGGATG | 87660766 | 87660788 | 0.21 |
| GCCCAAGATTCTCACCCTAG | 87660772 | 87660794 | 0.84 |
| GGTGAGAATCTTGGGCGGCC | 87660779 | 87660801 | 1.45 |
| GCTCTAAAGAAAAGGGACACT | 87660832 | 87660854 | 0.05 |
| GATGTGCATACTCTAAAGAAA | 87660841 | 87660863 | 0.02 |
| GCACATTGATCCTTATTTAG | 87660858 | 87660880 | 0.37 |
| GTTCATGACCACCTCTAAATA | 87660868 | 87660890 | 0.62 |
| GGTGGTCATGAAGGTCTTTC | 87660879 | 87660901 | 0.01 |
| GAAGGTCTTTCAGGATTCCG | 87660888 | 87660910 | 0.03 |
| GACACAGGGTGGAGAAGACCT | 87660905 | 87660927 | 0.03 |
| GTTCTCCACCCTGTGTTGTCC | 87660913 | 87660935 | 0.01 |
| GCAGTGTACCAGGACAACACA | 87660920 | 87660942 | 0.00 |
| GAAATAGACAGACAGTGTACC | 87660931 | 87660953 | 0.09 |
| GGCTGCATTTGTAGGCAATA | 87660976 | 87660998 | 0.03 |
| GAGAAGATAGACAAGATAGA | 87661025 | 87661047 | 0.00 |
| GAGAGAGAAGAAGGAGGCTG | 87661051 | 87661073 | 0.90 |
| GAAAAAAGAGAGAGAGAAGA | 87661060 | 87661082 | 0.02 |
| GCTTCCTCTGCTTCCTTAACC | 87661109 | 87661131 | 0.01 |
| GTTCTTTGTAAGGCCTGGTTA | 87661121 | 87661143 | 0.14 |
| GAACATTTTCTTTGTAAGGCC | 87661127 | 87661149 | 0.01 |
| GCTGCAAACATTTTCTTTGTA | 87661132 | 87661154 | 0.05 |
| GAAAATGTTTGCAGACCTTCT | 87661142 | 87661164 | 0.01 |
| GAAGGCTACAATGCCTAGA | 87661156 | 87661177 | 0.22 |
| GCTGCTTTTGACATTAGGAGA | 87661173 | 87661195 | 0.19 |
| GGCAGTCTGCTTTTGACATT | 87661179 | 87661201 | 0.01 |
| GCAGTTGCCATGGCTTTTAT | 87661201 | 87661223 | 0.01 |
| GCCAACAGGCAGCAGTTGCCA | 87661211 | 87661233 | 0.60 |
| GCCCTCTGATGTAGGATCAT | 87661233 | 87661255 | 0.12 |
| GAGCGCCTCTTAGAAGTAGA | 87661267 | 87661289 | 0.01 |

| | | | |
|---|---|---|---|
| GGCGCTCTACAAACATAGCT | 87661283 | 87661305 | 0.02 |
| GAAATTCTACAAGAGGAAA | 87661310 | 87661331 | 0.00 |
| GGTAAGAGAAATTCTACAAG | 87661316 | 87661338 | 0.02 |
| GACGCCGCACCAATTTGGCCA | 87661337 | 87661359 | 0.00 |
| GGCAGACGCCGCACCAATT | 87661343 | 87661364 | 0.01 |
| GTGGCAGGTCTAAAAGGGGC | 87661363 | 87661385 | 0.56 |
| GCTCTGGTGGCAGGTCTAAA | 87661369 | 87661391 | 0.46 |
| GTAGGGAGTGGCTCTGGTGGC | 87661378 | 87661400 | 0.76 |
| GCAGAAGGTAGGGAGTGGCTC | 87661385 | 87661407 | 0.03 |
| GCATGCCAGAAGGTAGGGAG | 87661391 | 87661413 | 0.01 |
| GGGACCCTAGCATGCCAGA | 87661401 | 87661422 | 0.27 |
| GGCATGCTAGGGTCCCAGTT | 87661407 | 87661429 | 0.01 |
| GGTCCCAGTTAGGACGATTC | 87661417 | 87661439 | 0.14 |
| GCTCCAGAAGAAAATAGATGC | 87661446 | 87661468 | 0.01 |
| GATTTTCTTCTGGAGTTGCGT | 87661455 | 87661477 | 0.01 |
| GAAGAACATGCTGGGGGCTAA | 87661482 | 87661504 | 0.01 |
| GGCACGAAAGAACATGCTGG | 87661489 | 87661511 | 1.69 |
| GCATATTTACCACTGAGGAAA | 87661528 | 87661550 | 0.02 |
| GACAGATCATATTTACCACTG | 87661534 | 87661556 | 0.02 |
| GCTGCTGACATTCATCCCAAG | 87661562 | 87661584 | 0.41 |
| GCATTCATCCCAAGTGGGCTG | 87661569 | 87661591 | 0.01 |
| GAAGTGGGCTGAGGAGAGAAC | 87661579 | 87661601 | 0.05 |
| GATTCCAATTATAGAGATCAG | 87661660 | 87661682 | 0.82 |
| GGAAAAGGGCCAATATTGAA | 87661684 | 87661706 | 0.01 |
| GAACTGTAGCCCCTTCAATAT | 87661693 | 87661715 | 0.01 |
| GAAGGGGCTACAGTTGTCCG | 87661701 | 87661723 | 0.80 |
| GTTGTCCGGGGTCTGCTGGT | 87661713 | 87661735 | 0.45 |
| GCTCCCCACCAGCAGACCC | 87661718 | 87661739 | 0.01 |
| GGGGAGCTGTAAGTACGTAG | 87661733 | 87661755 | 0.64 |
| GGCAATGCAGTAGTTGAATC | 87661754 | 87661776 | 0.00 |
| GTAGTTGAATCTGGTCCCTC | 87661763 | 87661785 | 0.03 |
| GAATCTGGTCCCTCTGGGCTC | 87661770 | 87661792 | 0.02 |
| GCCGACGAGCCCAGAGCCCAG | 87661779 | 87661801 | 0.56 |
| GGCTCTGGGCTCGTCGGCTT | 87661785 | 87661807 | 0.05 |
| GGCTCGTCGGCTTCGGGTTC | 87661792 | 87661814 | 0.01 |
| GGCTTCGGGTTCCGGCAGC | 87661800 | 87661821 | 0.65 |
| GCCATTGAAGGAGCAGCAGA | 87661823 | 87661845 | 0.03 |
| GTCTCAACAGGGTGCCATTGA | 87661835 | 87661857 | 0.24 |
| GCACCCTGTTGAGAGATCTG | 87661844 | 87661866 | 0.01 |
| GAGATCTGAGGGGGTGTTAT | 87661856 | 87661878 | 2.80 |
| GGGGTGTTATTGGGGTTATG | 87661866 | 87661888 | 0.01 |
| GAGATCGAAGAGTCTCAAAA | 87661902 | 87661924 | 0.02 |
| GTCGATCTCAGGATAAATCTT | 87661917 | 87661939 | 7.26 |
| GATAAATCTTAGGAGGAGCCT | 87661928 | 87661950 | 0.01 |
| GGAGCCTAGGGTTGCAGTTT | 87661941 | 87661963 | 0.02 |
| GGCTTATTCTTCTGAATCCA | 87662025 | 87662047 | 0.67 |
| GCTGAATCCAAGGTTTCAACC | 87662036 | 87662058 | 0.03 |
| GGTTTCAACCAGGATGGGGG | 87662046 | 87662068 | 0.01 |
| GCACTGGACCCCCCCCCATCC | 87662054 | 87662076 | 0.02 |
| GCTTGGTCGGGAGAGACCT | 87662106 | 87662127 | 0.02 |
| GAATTTCTGCTGAAGAGACCT | 87662122 | 87662144 | 0.01 |
| GATCAAATCAAGTGTGCCCTC | 87662148 | 87662170 | 0.36 |

| | | | |
|---|---|---|---|
| GAATATTGGTTGGCCTCCAGA | 87662163 | 87662185 | 0.75 |
| GGCCAACTTGCCGGGTGTGG | 87662363 | 87662385 | 0.18 |
| GTTAAGAATGGCCAACTTGCC | 87662371 | 87662393 | 0.29 |
| GAACTTTCTGCTCTTAAGAA | 87662384 | 87662406 | 0.00 |
| GTAAGAGCAGAAAGTTCTCCA | 87662391 | 87662413 | 0.01 |
| GTAGATGTCAAGAAGGGACCA | 87662408 | 87662430 | 0.04 |
| GCTACTTGTAGATGTCAAGAA | 87662415 | 87662437 | 0.01 |
| GGGCTCGGGGCTGGCGAGA | 87662627 | 87662648 | 0.02 |
| GAAATTAAGACTAGGGCTCG | 87662639 | 87662661 | 0.69 |
| GTTGAACTGATTCTTGACCAT | 87662665 | 87662687 | 3.23 |
| GCAGTTCAAGGGGAGTACTAA | 87662680 | 87662702 | 0.01 |
| GACATTGTCCAAGTCACATAC | 87662709 | 87662731 | 0.01 |
| GTTGTTTTCCTGTATGTGACT | 87662716 | 87662738 | 0.01 |
| GACTACAAAGACCGAGACC | 87662755 | 87662776 | 0.64 |
| GACCAGGACCCACCTCCTGC | 87662770 | 87662792 | 0.01 |
| GCTGGATCTATTTCTTGGTGT | 87662797 | 87662819 | 0.01 |
| GGACATCTGGATCTATTTCT | 87662803 | 87662825 | 0.01 |
| GAATAGATCCAGATGTCCGAG | 87662809 | 87662831 | 1.62 |
| GTTTGTGCCCACTCGGACATC | 87662816 | 87662838 | 0.40 |
| GCTCCTCTCCAGGAGGGTTTT | 87662843 | 87662865 | 0.01 |
| GATTGGAACCTAAAACCCTCC | 87662850 | 87662872 | 0.57 |
| GTTCCAATGCAACACCCAGA | 87662865 | 87662887 | 0.01 |
| GGAGGACAGAGGGAGCTCCA | 87662903 | 87662925 | 0.02 |
| GAAGACGTCCCAGGAGGACAG | 87662914 | 87662936 | 0.02 |
| GTCCTCCTGGGACGTCTTCC | 87662919 | 87662941 | 0.05 |
| GCCACCCTGGAAGACGTCCC | 87662924 | 87662946 | 0.04 |
| GACGTCTTCCAGGGTGGCAGC | 87662930 | 87662952 | 0.01 |
| GCAGACATGTAAGGGGATAAC | 87662949 | 87662971 | 0.01 |
| GTTATCTACAGACATGTAAG | 87662957 | 87662979 | 0.12 |
| GATAACCCTCCTTTCCATCC | 87662974 | 87662996 | 0.01 |
| GCTTGTCCGGGATGGAAAGGA | 87662979 | 87663001 | 0.02 |
| GAACGAACTCCTTGTCCGGGA | 87662988 | 87663010 | 0.02 |
| GTCGTTCGTTGCTCTCATGAG | 87663006 | 87663028 | 0.42 |
| GTTACCTCCAAGAGGGGTCTC | 87663099 | 87663121 | 0.03 |
| GCAATTCCCAGAGACCCCTCT | 87663105 | 87663127 | 0.02 |
| GGGGTCTCTGGGAATTGGGG | 87663111 | 87663133 | 0.02 |
| GTTGGGGTGGCCGCAAAATCC | 87663125 | 87663147 | 0.42 |
| GGGGCTCATCCGGGATTTTG | 87663134 | 87663156 | 0.26 |
| GTTTCAGTTGGGGGCTCATCC | 87663143 | 87663165 | 0.93 |
| GAAACGGGGTCTTTCAGTTG | 87663154 | 87663176 | 0.14 |
| GCGAGGGTCTCCCCGGAAAC | 87663169 | 87663191 | 5.57 |
| GTTGGGGCATCAGCTCATCCT | 87663198 | 87663220 | 0.02 |
| GAGCTGATGCCCCAATAAC | 87663206 | 87663227 | 1.02 |
| GATTTGGGAATCAACACGCTG | 87663265 | 87663287 | 0.18 |
| GACCCCCAGCAGTTGCAGTTC | 87663324 | 87663346 | 0.00 |
| GGTCCTTTACACAATGATTC | 87663491 | 87663513 | 0.00 |
| GTACACAATGATTCAGGCGCT | 87663498 | 87663520 | 0.01 |
| GCTTGGGTGGGTCACCCAG | 87663515 | 87663536 | 0.01 |
| GTTAAAAAACAAAGCCCAGCA | 87663577 | 87663599 | 0.02 |
| GTTTTAAGAAACACATTGAGT | 87663594 | 87663616 | 0.01 |
| GTTGGGGTCTTATAGCTTCA | 87663612 | 87663634 | 0.68 |
| GAAATTAAAAAAATAAGACAA | 87663654 | 87663676 | 0.03 |

| | | | |
|---|---|---|---|
| GTTTTTGCAAACTGATGCTTA | 87663684 | 87663706 | 0.01 |
| GCAAACTGATGCTTAAGGTC | 87663689 | 87663711 | 0.11 |
| GCTTAAGGTCAGGCTGCAG | 87663699 | 87663720 | 0.01 |
| GGCTGCAGTGGATCACCAC | 87663710 | 87663731 | 0.05 |
| GCGGTTCCACCATTTAACCA | 87663757 | 87663779 | 0.02 |
| GGAACCGCCTAACTAAAGCG | 87663772 | 87663794 | 1.45 |
| GACCATTCCGCGCTTTAGTT | 87663779 | 87663801 | 0.02 |
| GTCCAGATCCTGTTTCAATAC | 87663800 | 87663822 | 0.11 |
| GCCTCTCCCCGGTATTGAAAC | 87663807 | 87663829 | 0.49 |
| GACACAAACAGAACCTCTCCC | 87663819 | 87663841 | 0.01 |
| GCATACTTACTAGAAGGACTC | 87663909 | 87663931 | 0.02 |
| GATGATTCAAATTAACTCAGC | 87663968 | 87663990 | 0.05 |
| GACAAAAATCTGTTACTTCTC | 87664004 | 87664026 | 0.00 |
| GCAGATTTTTGTAGCTGCCTG | 87664016 | 87664038 | 0.01 |
| GCCTGCGGCCACACTTAACT | 87664031 | 87664053 | 0.01 |
| GCTCAGGAACCCAGTTAAGTG | 87664039 | 87664061 | 0.01 |
| GACAGCTCTGGCTTCTCACTC | 87664056 | 87664078 | 0.00 |
| GAAGCCAGAGCTGTATGGA | 87664065 | 87664086 | 0.12 |
| GGACTCTTAGTAAATTTTGG | 87664134 | 87664156 | 0.01 |
| GAGTCCGTGCTTATAAAGAG | 87664151 | 87664173 | 0.07 |
| GAACAGGCACCAAGAATGGGT | 87664188 | 87664210 | 0.03 |
| GCATCTAGCAGATCACCTAC | 87664216 | 87664238 | 0.05 |
| GATCTGCTAGATGCTTTCTC | 87664225 | 87664247 | 3.47 |
| GCTTTCTCTGGAATATCTCA | 87664237 | 87664259 | 0.01 |
| GAAGTTGGAAGGTTCCACACC | 87664326 | 87664348 | 0.01 |
| GAGAATGAGACCACCTGGTG | 87664339 | 87664361 | 0.01 |
| GCACTGAGAATGAGACCACC | 87664344 | 87664366 | 0.10 |
| GTCTCATTCTCAGTGCTAATG | 87664352 | 87664374 | 0.01 |
| GCCTCCTGGAGTAGGGGAAG | 87664435 | 87664457 | 0.68 |
| GGCCTGGCCTCCTGGAGTAG | 87664441 | 87664463 | 0.52 |
| GAACAATCAGACACAAGGCC | 87664457 | 87664479 | 0.08 |
| GATTAGGAACAATCAGACACA | 87664462 | 87664484 | 0.01 |
| GACTCAATTCAGTTGTAAATT | 87664479 | 87664501 | 0.07 |
| GACAGGATGAAGGCTGTCTTC | 87664513 | 87664535 | 3.00 |
| GCTTTTAAGCAACAGGATGA | 87664524 | 87664546 | 0.01 |
| GAAAGGTTGCTTTTAAGCAAC | 87664531 | 87664553 | 0.03 |
| GCAGATCAGTTACCTTCATAC | 87664570 | 87664592 | 3.70 |
| GATACAGGTTCACCTTCAGCA | 87664586 | 87664608 | 1.13 |
| GCCATGACTGAGCCTTGCTGA | 87664597 | 87664619 | 0.26 |
| GTTGAGTGCATATTGCGGCTT | 87664646 | 87664668 | 0.02 |
| GTATTGCGGCTTTGGTGTGAA | 87664655 | 87664677 | 0.01 |
| GCTTTGGTGTGAATGGGAAAA | 87664663 | 87664685 | 0.13 |
| GAGGGCTGGCAGTCAAAGAC | 87664694 | 87664716 | 0.38 |
| GCTTTTATCAACCTGAGACAG | 87664737 | 87664759 | 1.20 |
| GTTTGAACAAACACGGCCTCT | 87664766 | 87664788 | 0.05 |
| GTGTTTATTTTGAACAAACA | 87664774 | 87664796 | 0.01 |
| GTTCAAAATAAACACTAGA | 87664782 | 87664803 | 0.01 |
| GAAACACAACCAAGCTATACA | 87664820 | 87664842 | 0.01 |
| GCCTGGAGGCCATGTATAGCT | 87664828 | 87664850 | 0.08 |
| GCTATACATGGCCTCCAGGAC | 87664833 | 87664855 | 0.01 |
| GTTCAATCTTACCTGTCCTGG | 87664843 | 87664865 | 1.20 |
| GGACAGGTAAGATTGAACCT | 87664849 | 87664871 | 1.00 |

| | | | |
|---|---|---|---|
| GTGGGTGGTGGTCTGTGCCT | 87664866 | 87664888 | 0.01 |
| GCTGGGAATTATGGTGGGTGG | 87664878 | 87664900 | 0.01 |
| GTTCCAACTGGGAATTATGGT | 87664884 | 87664906 | 0.02 |
| GATTATTATTATTTTCCAACT | 87664896 | 87664918 | 0.01 |
| GCATAAAATTTGCTTATTAT | 87664942 | 87664964 | 0.02 |
| GCTGTTCCCCCATCCTTGAGC | 87665009 | 87665031 | 0.13 |
| GCTCAGACCTTGTCTGCCAGT | 87665036 | 87665058 | 0.01 |
| GCTGGCCAACTGGCAGACA | 87665042 | 87665063 | 0.01 |
| GCCAGTTGGCCAGCTCACAT | 87665050 | 87665072 | 0.02 |
| GACTCCACCCTATGTGAGC | 87665059 | 87665080 | 0.06 |
| GGGTGGAGTCTTCTGATCT | 87665071 | 87665092 | 0.05 |
| GCAGGATGCTGCAGAGGTGAC | 87665106 | 87665128 | 0.01 |
| GTAGTGGCAGGATGCTGCAG | 87665113 | 87665135 | 0.01 |
| GCTGCTCAGCAGGTAGTGGC | 87665125 | 87665147 | 0.03 |
| GACAAGCTGAGCTGCTCAGC | 87665135 | 87665157 | 0.09 |
| GTTGTCTTCCTGACTAGATCC | 87665153 | 87665175 | 0.04 |
| GACTAGATCCTGGCAAAGTG | 87665163 | 87665185 | 0.04 |
| GCTAAGGTCGGATTTGAAAGG | 87665196 | 87665218 | 0.78 |
| GTCAGTGTTTAATTCTAAGGT | 87665209 | 87665231 | 0.03 |
| GCTGGGGTTGGATGGGAAAGA | 87665274 | 87665296 | 0.02 |
| GCTATAACCGCCAGCGGCCAC | 87665320 | 87665342 | 6.08 |
| GCTGGCGGTTATAGATCTCA | 87665329 | 87665351 | 0.43 |
| GGCTGGTGTGGGACTCAGTA | 87665361 | 87665383 | 0.01 |
| GAAGTAGCTCTCTGGCTGGTG | 87665373 | 87665395 | 0.02 |
| GGGAAAAGTAGCTCTCTGGC | 87665378 | 87665400 | 0.02 |
| GTTGTTTGAGAAAGGATTTCT | 87665399 | 87665421 | 2.18 |
| GACAAAACAAAAGCATCCTAT | 87665433 | 87665455 | 0.53 |
| GTCCTATAGGATTGCAGCAGC | 87665447 | 87665469 | 0.02 |
| GATTGCAGCAGCAGGGGTGT | 87665455 | 87665477 | 0.01 |
| GTGTTGGTGGCCCCATGAAC | 87665471 | 87665493 | 0.62 |
| GCAGGATTCCTCCAGTTCATG | 87665481 | 87665503 | 0.01 |
| GTTTCTAGCACTCACAGCCTC | 87665500 | 87665522 | 0.00 |
| GCCACCCATGCTACATCAGC | 87665524 | 87665546 | 2.98 |
| GCTGGTTCTGATAGATGTTTG | 87665559 | 87665581 | 0.01 |
| GGGAACAACTTTCCCTTGCT | 87665581 | 87665603 | 0.00 |
| GTGCTAGAGAGGCCCAGCAA | 87665593 | 87665615 | 1.11 |
| GCCTCTCTAGCACCCTCTGC | 87665603 | 87665625 | 0.00 |
| GTATGAAGCAGTACCAGCAGA | 87665615 | 87665637 | 0.18 |
| GTTCATATCTACGTGCACATT | 87665632 | 87665654 | 0.00 |
| GTCTACGTGCACATTTGGAAT | 87665638 | 87665660 | 0.01 |
| GAACACACTGAGTTCTGTT | 87665667 | 87665688 | 2.05 |
| GTTCGGAAGCTACCCCTGCTA | 87665684 | 87665706 | 0.39 |
| GCATAGGCACACACCTTAGC | 87665697 | 87665719 | 0.10 |
| GCATGTACTGAGCGTGCAT | 87665713 | 87665734 | 0.23 |
| GATGCTCTCCAAAGCCTGTTG | 87665731 | 87665753 | 0.37 |
| GAACATCACCACAACAGGCTT | 87665738 | 87665760 | 0.00 |
| GAAGCAACATCACCACAAC | 87665744 | 87665765 | 0.01 |
| GTTGCTTCAGTCCCCACCT | 87665758 | 87665779 | 0.01 |
| GTACAGAAAGCATCCTAGGT | 87665770 | 87665792 | 0.01 |
| GCTTTCTGTACATTATCTT | 87665782 | 87665803 | 0.01 |
| GACATTATCTTTGGTGTCTCC | 87665791 | 87665813 | 0.82 |
| GATACAGTGACAAATGACC | 87665809 | 87665830 | 0.04 |

| | | | |
|---|---|---|---|
| GTCTGGAAACGGTAGCACTTC | 87665834 | 87665856 | 0.01 |
| GCAAAGAACAGATTCTGGAAA | 87665846 | 87665868 | 0.65 |
| GACGAAACAAAGAACAGATTC | 87665852 | 87665874 | 0.60 |
| GTTTCGTTTCTCTTGCAGGA | 87665868 | 87665890 | 0.01 |
| GATAACTAAGGCAAGATGGAC | 87665892 | 87665914 | 0.01 |
| GTCTAGATAACTAAGGCAAGA | 87665897 | 87665919 | 0.01 |
| GTCTACTCATCTAGATAACTA | 87665905 | 87665927 | 0.71 |
| GTATCTAGATGAGTAGATAGC | 87665912 | 87665934 | 0.07 |
| GCTCAGTATGAAGAGGAGACA | 87665945 | 87665967 | 0.01 |
| GATTGGGAACTCAGTATGAAG | 87665953 | 87665975 | 0.01 |
| GCTACTTATGTGAAACAATT | 87665970 | 87665992 | 0.04 |
| GGAAGGCATGAGATCACTAA | 87665992 | 87666014 | 0.00 |
| GGAAATGGATTTGTGGAGGA | 87666009 | 87666031 | 0.01 |
| GAAGACAGGAAATGGATTTG | 87666016 | 87666038 | 0.00 |
| GCCAAAACTGAAGACAGGAAA | 87666024 | 87666046 | 0.02 |
| GCTAATCCCAAAACTGAAGAC | 87666030 | 87666052 | 0.50 |
| GCTCAGTCTACGCATGCGTAC | 87666054 | 87666076 | 0.33 |
| GGGCAGAGGCTATAGCTCAG | 87666215 | 87666237 | 0.03 |
| GTTGAGGTAAAATGGGGCAG | 87666229 | 87666251 | 0.61 |
| GCAAGCCGTTGAGGTAAAATG | 87666235 | 87666257 | 2.17 |
| GTCTTTGCCACCAAGCCGTTG | 87666245 | 87666267 | 0.34 |
| GGCAAAGACCTTCAGCATTT | 87666260 | 87666282 | 0.02 |
| GCCTACATCCTAAATGCTGA | 87666268 | 87666290 | 0.02 |
| GTTTAGGATGTAGGCACCAGA | 87666277 | 87666299 | 8.52 |
| GTAACTTCACAGGGACCTTC | 87666292 | 87666314 | 0.01 |
| GAAAGGGGCTGTAACTTCACA | 87666301 | 87666323 | 2.41 |
| GTTACAGCCCCTTTGAACTCC | 87666311 | 87666333 | 3.94 |
| GGTAGCCCTGGAGTTCAAAG | 87666317 | 87666339 | 0.01 |
| GAGGTCTCACCAGGTAGCCC | 87666329 | 87666351 | 0.03 |
| GTTGGAGGTGAGGTCTCACC | 87666338 | 87666360 | 1.16 |
| GGGTTTTAGTGTTGGAGGTG | 87666348 | 87666370 | 0.01 |
| GTTTTGCTGGGTTTTAGTGT | 87666356 | 87666378 | 0.00 |
| GATCTTTGATTTTGTTTTGCT | 87666368 | 87666390 | 0.01 |
| GAAACAAAATCAAAGATGTAT | 87666375 | 87666397 | 0.01 |
| GTTGCCTCACCAAGAATCTGT | 87666398 | 87666420 | 0.93 |
| GAATCTGTTGGGAGCCATTA | 87666410 | 87666432 | 0.01 |
| GAAATATTTATCAGTGGGAG | 87666641 | 87666663 | 0.02 |
| GAAGGAGAAATATTTATCAGT | 87666646 | 87666668 | 0.01 |
| GTTGATTCTGACCCGTCCAGC | 87666935 | 87666957 | 0.03 |
| GATGTAAGTCATAGACTATTT | 87666960 | 87666982 | 0.41 |
| GCACTAGCTCCATTCTTCATC | 87666993 | 87667015 | 0.01 |
| GCATGAATTCCTGATGAAGAA | 87667001 | 87667023 | 0.01 |
| GATCAGTGAAGACAGATTAA | 87667026 | 87667048 | 0.02 |
| GAATGTGGAATTTGAGCAAGA | 87667073 | 87667095 | 0.00 |
| GAAATGATTTTGGTGTCAATG | 87667089 | 87667111 | 0.06 |
| GAAGCTTTAGGCAAATGATTT | 87667100 | 87667122 | 0.01 |
| GAGACGGTATGCTAAGCTTT | 87667113 | 87667135 | 0.02 |
| GATACCGTCTCTTGGGAGTTC | 87667126 | 87667148 | 0.08 |
| GTCACGAATTATTCACTATTT | 87667166 | 87667188 | 0.01 |
| GTTAGGTGAGCAGTCTACCAG | 87667184 | 87667206 | 0.45 |
| GGGGTTGAGATGTAGTCCGC | 87667200 | 87667222 | 0.11 |
| GGAAAAAAGTAACTGACAAG | 87667219 | 87667241 | 0.01 |

| | | | |
|---|---|---|---|
| GTTCCCGTGTGTTTTTGAGAC | 87667238 | 87667260 | 0.02 |
| GAGTGAGTTTTAGGCCAGCC | 87667276 | 87667298 | 0.01 |
| GCTGGCCTAAAACTCACTCTG | 87667281 | 87667303 | 0.92 |
| GGCAGATCTCTATGAGGAAG | 87667314 | 87667336 | 0.00 |
| GAGCAACACTCAGGAGGCAG | 87667339 | 87667361 | 0.02 |
| GTTAACTGAGCAACACTCAGG | 87667345 | 87667367 | 0.67 |
| GATTTTAGAAATTTATATCGT | 87667367 | 87667389 | 0.02 |
| GTCTCAAAAATAGAAGGGAAA | 87667390 | 87667412 | 0.03 |
| GATCCTTCTCAAAAATAGAA | 87667396 | 87667418 | 0.02 |
| GGATCGCTTTAAGTTGCAC | 87667414 | 87667435 | 0.00 |
| GACACCTGAAAGCTGAGGCT | 87667449 | 87667471 | 0.02 |
| GGCAGACACCTGAAAGCTG | 87667454 | 87667475 | 0.06 |
| GATATGTAAATCAGGTCTGG | 87667474 | 87667496 | 0.00 |
| GAGATGGGATATGTAAATC | 87667482 | 87667503 | 0.05 |
| GCTCACTACGCAGCATGAGAT | 87667496 | 87667518 | 0.35 |
| GAGTGATCTAGTTGAATGTC | 87667516 | 87667538 | 0.01 |
| GTTGTTTGTTTGTATTGAGAT | 87667552 | 87667574 | 0.00 |
| GATAGGTACAGCATGCCTGA | 87667569 | 87667591 | 0.01 |
| GCATGCCTGAAGGCAGGAAA | 87667579 | 87667601 | 0.18 |
| GTTCAACCATTTCCTGCCTTC | 87667584 | 87667606 | 0.07 |
| GAAATGGTTGAATCTAACTA | 87667595 | 87667617 | 0.01 |
| GTTGTCTGCAGTGGTCGCTT | 87667624 | 87667646 | 0.89 |
| GAAGAGGAGGTTGTCTGCAG | 87667633 | 87667655 | 0.08 |
| GTAGGATTATAATGAAGAGG | 87667646 | 87667668 | 0.02 |
| GTTCATTATAATCCTACCTAC | 87667653 | 87667675 | 0.44 |
| GGACTTGTCACTTAACCTGT | 87667668 | 87667690 | 0.13 |
| GACAAGTCCCCAAGTGTACC | 87667682 | 87667704 | 0.03 |
| GCAACTGTCCTGGTACACTT | 87667690 | 87667712 | 0.01 |
| GATGGCTGTCTGCAACTGTCC | 87667700 | 87667722 | 0.83 |
| GTTGCAGACAGCCATGGCTG | 87667708 | 87667730 | 1.50 |
| GACAGCCATGGCTGGGGCCA | 87667714 | 87667736 | 0.01 |
| GTTCTCCCTGGCCCCAGCCA | 87667719 | 87667741 | 0.56 |
| GTCTTTTCACAAAGTTCTCCC | 87667731 | 87667753 | 0.01 |
| GAGAACTTTGTGAAAAGAGG | 87667736 | 87667758 | 0.05 |
| GTTGTCATTCCCAGAAAATAT | 87667778 | 87667800 | 2.25 |
| GAAGACTATTCCTATATTTTC | 87667787 | 87667809 | 0.01 |
| GGCGAAGGATAGGCAAATGA | 87667812 | 87667834 | 0.25 |
| GAAAAACTATGGGCGAAGGAT | 87667822 | 87667844 | 0.21 |
| GAAAAAAAAAACTATGGGCGA | 87667827 | 87667849 | 0.03 |
| GTTATGGGCAGGCTGGAGAGA | 87668020 | 87668042 | 0.02 |
| GGTTAAAAAAAATTATGGGC | 87668032 | 87668054 | 0.62 |
| GATGGGGTTAAAAAAAATTAT | 87668036 | 87668058 | 0.03 |
| GAAAAAGTAAGTTAAAATATG | 87668053 | 87668075 | 0.43 |
| GAAAAAAAATGTAGCTCTGCA | 87668082 | 87668104 | 0.03 |
| GCTGTGATCTGGAAGCAAAGA | 87668127 | 87668149 | 0.65 |
| GTTGCTTCCAGATCACAGGTG | 87668133 | 87668155 | 0.26 |
| GTGGCCCACACCTGTGATC | 87668139 | 87668160 | 0.01 |
| GTGGGCCACTTGCTATTTGT | 87668152 | 87668174 | 0.34 |
| GTAAAACCTACAAATAGCAAG | 87668157 | 87668179 | 0.01 |
| GTCTGAGGACAGTCTGGGC | 87668216 | 87668237 | 0.05 |
| GTCTTAGTCTGAGGACAGTC | 87668221 | 87668243 | 0.01 |
| GAGGCAGAAGTCTTAGTCTG | 87668230 | 87668252 | 1.24 |

| | | | |
|---|---|---|---|
| GAAGACTTCTGCCTCAGTCTC | 87668239 | 87668261 | 0.18 |
| GCAGCTCCATCCAGAGACTG | 87668249 | 87668271 | 0.01 |
| GCTTACAGATACAGGACAAG | 87668283 | 87668305 | 4.77 |
| GTCCTGTATCTGTAAGCTCC | 87668289 | 87668311 | 0.01 |
| GTTCTCAATCAGCTGGGGGCC | 87668307 | 87668329 | 0.53 |
| GAACCAGTTTCTCAATCAGC | 87668315 | 87668337 | 0.01 |
| GATTGAGAAACTGGTTCAAG | 87668321 | 87668343 | 0.16 |
| GGTTTTTCCTGCTCTACTCT | 87668342 | 87668364 | 0.01 |
| GCAGAAACCCAAGAGTAGAGC | 87668349 | 87668371 | 0.58 |
| GTTTTGTTTGTTTTGAGGCT | 87668371 | 87668393 | 0.08 |
| GGGCACAGATTTGATAAAG | 87668391 | 87668412 | 0.05 |
| GCAGATTTGATAAAGTGGCTG | 87668396 | 87668418 | 0.01 |
| GCCAGGTGGAGCAGGTGTTCA | 87668427 | 87668449 | 0.62 |
| GACGTGGGACCAGGTGGAGC | 87668436 | 87668458 | 0.03 |
| GACTTGGGACGTGGGACCAGG | 87668442 | 87668464 | 0.28 |
| GGTCCCACGTCCCAAGTGCT | 87668448 | 87668470 | 0.03 |
| GCAGGAAGTCATCCCAGCACT | 87668459 | 87668481 | 5.71 |
| GCAGGTGGGTATGGTGACAC | 87668478 | 87668500 | 0.01 |
| GTCACCATACCCACCTGCCT | 87668483 | 87668505 | 0.60 |
| GCCGATTCACACCAAGGCAGG | 87668493 | 87668515 | 0.01 |
| GTCTATCCCGATTCACACCA | 87668500 | 87668522 | 0.38 |
| GTATTCCTTTTCTAGAATAAA | 87668523 | 87668545 | 0.02 |
| GAATCACAAACACGACTGTGG | 87668562 | 87668584 | 0.01 |
| GTGATTCTACAACTTCATG | 87668579 | 87668600 | 2.88 |
| GATGAGGAAAAAGAACCCAGC | 87668595 | 87668617 | 4.74 |
| GATTATGTTTTAACTCCAGCT | 87668609 | 87668631 | 0.01 |
| GTAAAACATAATGATTTATAA | 87668621 | 87668643 | 0.08 |
| GCAACGATATGTATGTATAGA | 87668654 | 87668676 | 0.02 |
| GTTGGCAAGAAAAAAAAATGT | 87668673 | 87668695 | 0.01 |
| GAGCACGGACTGCTCTTCTA | 87668723 | 87668745 | 0.01 |
| GACATAAAATAAAGCCAGGCG | 87668848 | 87668870 | 0.54 |
| GTAAAATAAAGCCAGGCGTGG | 87668851 | 87668873 | 0.61 |
| GAACCTCTCAAAACACACACG | 87669078 | 87669100 | 0.65 |
| GTTGTCATCTTTCTGAAGTGT | 87669109 | 87669131 | 0.00 |
| GCAGAAAGATGACAACAGGGC | 87669118 | 87669140 | 0.01 |
| GACAGGGCAGGAGGTGAGCCC | 87669131 | 87669153 | 0.82 |
| GGTGAGCCCTGGCTATATA | 87669142 | 87669163 | 0.03 |
| GCAGGTTGGCCGTATATAGCC | 87669149 | 87669171 | 1.47 |
| GTCTTAAATAATAGCAGCCC | 87669168 | 87669190 | 2.44 |
| GGGCTGCTATTATTTAAGAC | 87669171 | 87669193 | 1.25 |
| GCTGGCCACTTTTCTATGCCA | 87669190 | 87669212 | 0.01 |
| GCATAAGCTGGCTGTCAGCCT | 87669207 | 87669229 | 0.90 |
| GACAAAATGAAGAACATAAGC | 87669220 | 87669242 | 0.07 |
| GTATCCATTAACTTCAGAAA | 87669241 | 87669263 | 0.00 |
| GTCACATATGGCTACTTCTTG | 87669275 | 87669297 | 0.01 |
| GCAGGTTTTTAACTTCACATA | 87669288 | 87669310 | 0.09 |
| GCATTTACTAGTTTGTCTGAC | 87669307 | 87669329 | 0.32 |
| GAATGTGTGAGTATTCACCCC | 87669356 | 87669378 | 0.01 |
| GTATTCACCCCAGGTCTTTTT | 87669366 | 87669388 | 0.01 |
| GTACACACCTAAAAAGACCTG | 87669372 | 87669394 | 0.02 |
| GTGTAGTAAATAAAGAGAC | 87669390 | 87669411 | 0.01 |
| GAGACAGGTACCCAGACTCC | 87669404 | 87669426 | 0.01 |

| | | | |
|---|---|---|---|
| GTTCTGAGGTCCCAGGAGTCT | 87669414 | 87669436 | 0.17 |
| GCATTTCTTCTGAGGTCCC | 87669422 | 87669443 | 1.57 |
| GAACTAGCTGCATTTCTTCTG | 87669429 | 87669451 | 0.50 |
| GAAATGCAGCTAGTTACAACC | 87669438 | 87669460 | 1.65 |
| GTACAACCCGGGGAACTGAGC | 87669451 | 87669473 | 0.83 |
| GAAAGCCTGCTCAGTTCCCC | 87669456 | 87669478 | 0.01 |
| GCTGAGCAGGCTTTCCAGCGA | 87669465 | 87669487 | 0.03 |
| GGCTTTCCAGCGAAGGGATT | 87669472 | 87669494 | 1.82 |
| GCTCGGTCCAAATCCCTTCGC | 87669478 | 87669500 | 1.04 |
| GTCTGGTCATCTTTCAATCCT | 87669496 | 87669518 | 0.00 |
| GACCAGACATTTCTGACAGAA | 87669513 | 87669535 | 1.47 |
| GTCTGACAGAAAGGTCAAAAG | 87669523 | 87669545 | 0.13 |
| GAGGGCCATTCCAGAGCAGA | 87669542 | 87669564 | 0.17 |
| GCTTGACCTTCTGCTCTGGAA | 87669547 | 87669569 | 0.56 |
| GTACACTTGACCTTCTGCTC | 87669552 | 87669574 | 0.01 |
| GAAGGTCAAGTGTACACAG | 87669560 | 87669581 | 0.16 |
| GCAAGTGTACACAGAGGCCTC | 87669566 | 87669588 | 0.00 |
| GTTAATTTGTCTTGCAACCTG | 87669582 | 87669604 | 0.37 |
| GCAAGACAAATTAAGTTCA | 87669591 | 87669612 | 0.13 |
| GTTAAGTTCATGGTTCCTGAT | 87669601 | 87669623 | 0.01 |
| GCATGGTTCCTGATTGGTGTC | 87669608 | 87669630 | 0.02 |
| GCTATTTCCCTGACACCAATC | 87669615 | 87669637 | 0.24 |
| GTTGGTGTCAGGGAAATAGGC | 87669620 | 87669642 | 2.25 |
| GGCAGGCATGTTCAAGCGTC | 87669637 | 87669659 | 0.01 |
| GCAAGCGTCAGGAAATGTGGC | 87669649 | 87669671 | 0.02 |
| GTCAGGAAATGTGGCTGGGG | 87669654 | 87669676 | 0.29 |
| GTTCTTTGTTTTCCAAGTATG | 87669703 | 87669725 | 0.01 |
| GTATGTGGATATTAGCCTGT | 87669718 | 87669740 | 0.87 |
| GTTTAGACACTACTAACCCAC | 87669733 | 87669755 | 1.18 |
| GGGTTAGTAGTGTCTAAATT | 87669738 | 87669760 | 0.06 |
| GTTTGGCTGTCAGAAATATCC | 87669756 | 87669778 | 7.72 |
| GGAAACTCCTTAAAGACACT | 87669777 | 87669799 | 7.55 |
| GGCCTTTCCCAGTGTCTTTA | 87669784 | 87669806 | 2.70 |
| GACACTGGGAAAGGCCAGGTA | 87669792 | 87669814 | 0.32 |
| GATGCCTGTAACCCCAGTTTA | 87669821 | 87669843 | 0.12 |
| GCTGAGAATGACCATAAACT | 87669832 | 87669854 | 1.06 |
| GACAAGACTACGTGTAGCTC | 87669877 | 87669899 | 3.62 |
| GTTGTTTTGTTTAGTTTTGTT | 87669899 | 87669921 | 0.00 |
| GCAACAAAACCTACAGAGAAA | 87669922 | 87669944 | 0.20 |
| GAAACCTACAGAGAAATGGGG | 87669927 | 87669949 | 0.01 |
| GTGGGGAGTGTCAAGCACAG | 87669946 | 87669968 | 3.86 |
| GTGGACCCTCGCCTCCTTC | 87669965 | 87669986 | 4.80 |
| GTAAAGACCTGAAGGAGGCGA | 87669970 | 87669992 | 1.37 |
| GATGGGATAAAGACCTGAAGG | 87669976 | 87669998 | 0.07 |
| GATCGTGGGTTAAATTCTAT | 87669994 | 87670016 | 0.20 |
| GCTGTGTTTTGTATTGATCG | 87670009 | 87670031 | 4.33 |
| GAAAACACAGCCTCACGTCAA | 87670022 | 87670044 | 0.07 |
| GCTCTTAGTCCCTTTGACGTG | 87670031 | 87670053 | 0.27 |
| GACTAAGAGATAGTTTATTT | 87670045 | 87670067 | 0.22 |
| GTTTATTTTGGTGTCAAATAT | 87670058 | 87670080 | 0.01 |
| GTGTCAAATATAGGCGACCA | 87670067 | 87670089 | 0.03 |
| GCCTACAGTGTTTCTAGGCCA | 87670084 | 87670106 | 2.34 |

| | | | |
|---|---|---|---|
| GTAAATGCCTACAGTGTTTCT | 87670090 | 87670112 | 0.12 |
| GGGAGGGGGAGATGAGTAG | 87670115 | 87670136 | 0.03 |
| GACAGTAGGTGGGGAGAGGG | 87670131 | 87670153 | 0.01 |
| GAGAGACAGTAGGTGGGGAG | 87670135 | 87670157 | 0.01 |
| GACAGAGAGAGACAGTAGGT | 87670141 | 87670163 | 0.04 |
| GAGTTGAAGGAATAGAAAAA | 87670177 | 87670199 | 4.32 |
| GACCTGGGGAGGGGAGTTGA | 87670190 | 87670212 | 0.04 |
| GATACTCTATCACTGTGTGTC | 87670231 | 87670253 | 2.19 |
| GCACTGTGTGTCTGGTCACTC | 87670240 | 87670262 | 0.40 |
| GTCTGGTCACTCAGGGGGAA | 87670248 | 87670270 | 0.98 |
| GTTTGGTGAGGTAAGGTGTG | 87670306 | 87670328 | 0.00 |
| GGAAAATGTTTGGTGAGGTA | 87670313 | 87670335 | 1.17 |
| GAAGATGGAAAATGTTTGGTG | 87670318 | 87670340 | 0.53 |
| GATAAAAAGATGGAAAATGTT | 87670323 | 87670345 | 0.01 |
| GAAACACAGTAAGCACAGTCT | 87670355 | 87670377 | 0.01 |
| GGTATATCATGTTGGAACA | 87670390 | 87670411 | 0.01 |
| GATGAGACTGGTATATCATGT | 87670397 | 87670419 | 1.20 |
| GTTAATTAATAAAAATGAGAC | 87670410 | 87670432 | 0.02 |
| GATGGGACTCGTGTACTTA | 87670475 | 87670496 | 0.11 |
| GTACACGAGTCCCATCGCGT | 87670481 | 87670503 | 0.06 |
| GCTTCCACACCCCCACGCGAT | 87670491 | 87670513 | 0.08 |
| GTGGGGGTGTGGAAGTCTG | 87670499 | 87670520 | 0.02 |
| GGAAGTCTGAGGATAACTTG | 87670509 | 87670531 | 0.06 |
| GGAGTTGGTTCTCTCTACTT | 87670531 | 87670553 | 0.01 |
| GTCTACTTTGGGGCCTGTATT | 87670544 | 87670566 | 0.03 |
| GAAGGTCTAGTGGCCTAATAC | 87670556 | 87670578 | 0.39 |
| GCTTTTATTTTAGTTTTGGC | 87670644 | 87670666 | 0.01 |
| GCTGTGGACTTTTGATGAATT | 87670667 | 87670689 | 0.02 |
| GTCATCAAAAGTCCACAGTTC | 87670673 | 87670695 | 0.19 |
| GTAGAAACTTTACCAGAACTG | 87670684 | 87670706 | 0.21 |
| GTAAAGTTTCTACATGTCCTA | 87670696 | 87670718 | 3.35 |
| GAAAAAAAAAAAAGCAACCTT | 87670712 | 87670734 | 0.02 |
| GTTTTTTTTTAGTTCCACTTC | 87670731 | 87670753 | 0.00 |
| GGTTAACTGTTCTTGTTAAT | 87670752 | 87670774 | 0.01 |
| GTATACATATATTTATATTCT | 87670785 | 87670807 | 0.01 |
| GTAGCACTGAACTTTTCTTG | 87670829 | 87670851 | 0.34 |
| GAAAAGTTCAGTGCTACAAT | 87670835 | 87670857 | 0.02 |
| GCAATGGGATCCTGAATACCC | 87670851 | 87670873 | 0.03 |
| GTAACTACCCTGGGTATTC | 87670860 | 87670881 | 0.15 |
| GACTTGACCAGTAACTACCCT | 87670868 | 87670890 | 0.49 |
| GAAGTAATAAAGCTGTCCTAT | 87670887 | 87670909 | 0.01 |
| GTCCTATTGGCTTAAACCTG | 87670900 | 87670922 | 0.02 |
| GAGAAAACAACTTGCCCAC | 87670916 | 87670937 | 0.74 |
| GGGCAAGTTGTTTTCTCTGG | 87670921 | 87670943 | 0.26 |
| GTCTGGTGGATACCACACAAT | 87670936 | 87670958 | 0.98 |
| GGATACCACACAATAGGTCC | 87670942 | 87670964 | 0.01 |
| GCCGTGCCTGGACCTATTGTG | 87670947 | 87670969 | 0.47 |
| GTAGGTCCAGGCACGGAATCC | 87670955 | 87670977 | 0.00 |
| GATGCCCTGGATTCCGTGCC | 87670960 | 87670982 | 0.09 |
| GGAATCCAGGGCATCGGGCT | 87670968 | 87670990 | 0.01 |
| GTGTACCAAGCCCGATGCCC | 87670973 | 87670995 | 0.02 |
| GACTGAGATGGCTCTGGGTGA | 87670999 | 87671021 | 0.39 |

| | | | |
|---|---|---|---|
| GACGGCCACTGAGATGGCTC | 87671006 | 87671028 | 0.01 |
| GTTTCTCGACGGCCACTGAGA | 87671012 | 87671034 | 10.92 |
| GTTTCTATACAGTTTCTCGA | 87671024 | 87671046 | 5.02 |
| GACAAAGAAAGTTTTAAATCA | 87671050 | 87671072 | 0.41 |
| GAATCAAGGCCGTAGAAACTA | 87671065 | 87671087 | 1.43 |
| GTACAGACCCCTTAGTTTCTA | 87671073 | 87671095 | 9.63 |
| GATTATATTTGATAATCACA | 87671100 | 87671122 | 0.41 |
| GATAATCACAAGGTTGTTA | 87671110 | 87671131 | 0.01 |
| GGTTGTTAAGGTAAACACAG | 87671121 | 87671143 | 0.01 |
| GTGGGCAATAGTAGCTCTTT | 87671143 | 87671165 | 0.27 |
| GACAATTAGCTCAAGATAATT | 87671171 | 87671193 | 0.03 |
| GCTCAAGATAATTTGGCTCA | 87671178 | 87671200 | 0.44 |
| GCTGATTAAGATGTGATTGTA | 87671218 | 87671240 | 0.34 |
| GCAGGGTTATAAACATTCTTA | 87671250 | 87671272 | 0.02 |
| GAACCCTGCAGTTTTTCCTGT | 87671266 | 87671288 | 0.01 |
| GACTAAGAGGTGTAAACCGAC | 87671281 | 87671303 | 6.37 |
| GACAGAAAGATGGAGACTAAG | 87671295 | 87671317 | 0.05 |
| GTAAACAAACACACAGAAAGA | 87671306 | 87671328 | 9.02 |
| GTCTGCATCCATTCACTAGCA | 87671352 | 87671374 | 0.99 |
| GTTTTCTTCCGTGCTAGTGAA | 87671359 | 87671381 | 1.13 |
| GGAGGTAGAGAGGTTGCATC | 87671383 | 87671405 | 0.03 |
| GGAGGCGGGGGAGGTAGAG | 87671393 | 87671414 | 1.70 |
| GAAAAGGGTGGAGGCGGGGG | 87671401 | 87671423 | 0.04 |
| GAATAAAGAAAAGGGTGGAGG | 87671407 | 87671429 | 0.01 |
| GACCCTTTTCTTTATTGAGAC | 87671415 | 87671437 | 0.01 |
| GCTACACAGAAATCCAAAGC | 87671443 | 87671465 | 2.93 |
| GAATAAATAAACATCCTGCTT | 87671456 | 87671478 | 0.07 |
| GTTTATTTAGAACTCCAAAGC | 87671473 | 87671495 | 0.45 |
| GAATAAATAAATAACCTGCTT | 87671486 | 87671508 | 0.05 |
| GTTTATTTAGGATGCAGTCTC | 87671515 | 87671537 | 0.66 |
| GACGCAGCTGGAGGTCAATTT | 87671541 | 87671563 | 0.05 |
| GACCTCCAGCTGCGTAGCCA | 87671549 | 87671571 | 0.10 |
| GATTATCCTTGGCTACGCAGC | 87671554 | 87671576 | 44.29 |
| GACACAGTACAGTATTATCCT | 87671566 | 87671588 | 2.60 |
| GCCAAAAGTTAAAAGTGGGTC | 87671595 | 87671617 | 19.98 |
| GAAGGGCCAAAAGTTAAAAG | 87671601 | 87671623 | 0.40 |
| GCAGCTCTCAGGAGGTCAGAA | 87671618 | 87671640 | 0.01 |
| GTTGAAACCCCAGCTCTCAGG | 87671627 | 87671649 | 0.01 |
| GATTAAATGTGGAAACGAGTA | 87671660 | 87671682 | 0.51 |
| GAAAAATATATATATTAAATG | 87671672 | 87671694 | 0.01 |
| GACCTCCTCCACCCACACCG | 87671722 | 87671744 | 0.56 |
| GCAGATCCTCGGTGTGGGTGG | 87671727 | 87671749 | 1.33 |
| GTAACTCCAGATCCTCGGTG | 87671734 | 87671756 | 1.06 |
| GAACTGGTAACTCCAGATCCT | 87671739 | 87671761 | 4.50 |
| GCTAGTGTAGCTCATCATAAC | 87671756 | 87671778 | 5.18 |
| GATGAGCTACACTAGTGATG | 87671764 | 87671786 | 5.42 |
| GATGGGGACTGAACTCAAGGT | 87671781 | 87671803 | 1.53 |
| GTAGGCGACTATGAAAACATT | 87671800 | 87671822 | 0.09 |
| GTATGAAAACATTAGGAAGTT | 87671808 | 87671830 | 1.25 |
| GACAAATAAAGGACAGATTGG | 87671842 | 87671864 | 0.52 |
| GGGGGCTCAGCACCTTGTC | 87671888 | 87671909 | 0.01 |
| GAATAAATAAAGGACAGATGG | 87671905 | 87671927 | 0.01 |

| | | | |
|---|---|---|---|
| GTCTGGTAAATAAATAAATAA | 87671916 | 87671938 | 0.02 |
| GCTGTGTAGTCCTGACTTCCC | 87671949 | 87671971 | 0.95 |
| GAATCGAGTTCCAGGGAAGTC | 87671958 | 87671980 | 0.82 |
| GGTCTACCAATCGAGTTCCA | 87671966 | 87671988 | 14.53 |
| GACTCGATTGGTAGACCTGGC | 87671973 | 87671995 | 0.23 |
| GTAGACCTGGCTGGCCTCAG | 87671982 | 87672004 | 0.11 |
| GGGGGCCTCTGAGGCCAGCC | 87671987 | 87672009 | 0.01 |
| GCAGAGGCCCCCCGAGTGCT | 87671999 | 87672021 | 0.01 |
| GCCCTTAATCCAAGCACTCG | 87672008 | 87672030 | 0.01 |
| GGGCATGCGCCACCACGCT | 87672027 | 87672048 | 0.02 |
| GACACTTGGCAAGCCGAGCG | 87672039 | 87672061 | 2.07 |
| GTCGGCTTGCCAAGTGTCTAT | 87672045 | 87672067 | 1.79 |
| GAATTCATCCAATAGACACT | 87672053 | 87672075 | 3.16 |
| GGATGAATTCGTCAACCGAT | 87672066 | 87672088 | 0.15 |
| GATGGGCGAAATGCAAAACT | 87672083 | 87672105 | 1.46 |
| GCAAAACTGGGTCAGCAAG | 87672095 | 87672116 | 0.78 |
| GTATACCTGAAATTTTACCTA | 87672127 | 87672149 | 0.02 |
| GCTTGCAGTGCAAACTGCCAT | 87672143 | 87672165 | 0.01 |
| GTATAACGCACACTCAAGGC | 87672167 | 87672189 | 0.01 |
| GCACACTCAAGGCTGGGTGG | 87672174 | 87672196 | 0.28 |
| GATTTCACTCTTACGTTTC | 87672231 | 87672252 | 1.64 |
| GAATTTGTTTTATCAAGTGAC | 87672249 | 87672271 | 0.18 |
| GTGCTAGGCGGGGTCAGCT | 87672272 | 87672293 | 1.72 |
| GTGGGTAGGTGTGCTAGGCG | 87672281 | 87672303 | 0.65 |
| GGTGGGTGGGTAGGTGTGCT | 87672286 | 87672308 | 21.51 |
| GACACCTACCCACCCACCAGG | 87672292 | 87672314 | 2.46 |
| GTTCAAGTCCGCCTGGTGGGT | 87672299 | 87672321 | 7.33 |
| GCGCTTTCAAGTCCGCCTGG | 87672304 | 87672326 | 5.19 |
| GGACTTGAAAGCGCAGCCTC | 87672313 | 87672335 | 0.48 |
| GCAGCCTCTGGAAGGCCTGCA | 87672326 | 87672348 | 1.30 |
| GCCTGCAAGGCTGCAGAAGT | 87672339 | 87672361 | 2.01 |
| GCTAGAGTCCTAGAGTTCGC | 87672376 | 87672398 | 0.91 |
| GTCCTAGAGTTCGCGGGAAGC | 87672383 | 87672405 | 0.00 |
| GGACCTTGCTCGCTTATGAT | 87672405 | 87672427 | 1.81 |
| GTCGCTTATGATTGGCTACTG | 87672414 | 87672436 | 1.52 |
| GCGGTAGACTCCCACCCACC | 87672433 | 87672455 | 0.79 |
| GCAGCGCTGCGCCCCGGTGGG | 87672444 | 87672466 | 0.01 |
| GCACCATGCAGCGCTGCGCCC | 87672451 | 87672473 | 3.37 |
| GCGCAGCGCTGCATGGTGAT | 87672456 | 87672478 | 0.12 |
| GTGATTGGGCGAGCCCACAG | 87672471 | 87672493 | 0.55 |
| GAGCCCACAGAGGCCGGACG | 87672481 | 87672503 | 1.63 |
| GAGGCCGGACGAGGTTTAGG | 87672490 | 87672512 | 8.02 |
| GCTCGCCCCCTAAACCTCGTC | 87672494 | 87672516 | 0.86 |
| GAGGTTTAGGGGGCGAGGTT | 87672500 | 87672522 | 4.29 |
| GGGGGCGAGGTTGGGGAGGC | 87672508 | 87672530 | 2.33 |
| GGGGAGGCGGGAAACGACG | 87672520 | 87672541 | 0.00 |
| GGAAACGACGCGGCCAGCGC | 87672529 | 87672551 | 5.28 |
| GCACAGAGCGACCCTGCGC | 87672542 | 87672563 | 0.95 |
| GGGTCGCTCTGTGCTCTAG | 87672550 | 87672571 | 10.16 |
| GCTCTGTGCTCTAGTGGTGT | 87672555 | 87672577 | 1.73 |
| GTGCTCTAGTGGTGTTGGTG | 87672560 | 87672582 | 1.28 |
| GCTAGTGGTGTTGGTGTGGAG | 87672565 | 87672587 | 3.76 |

| | | | |
|---|---|---|---|
| GGTGTTGGTGTGGAGCGGCT | 87672570 | 87672592 | 0.01 |
| GGTGTGGAGCGGCTCGGAAA | 87672576 | 87672598 | 2.30 |
| GGAAATGGCGGTGCAGCCTA | 87672591 | 87672613 | 4.51 |
| GCCTAAGGAGACGCTGCAGT | 87672606 | 87672628 | 48.84 |
| GACGCTGCAGTTGGAAGGCG | 87672615 | 87672637 | 11.59 |
| GCAGTTGGAAGGCGCGGCCG | 87672621 | 87672643 | 8.51 |
| GAAGCGCACGAAGCCCGCCT | 87672638 | 87672660 | 1.27 |
| GGGCTTCGTGCGCTTCTTTG | 87672645 | 87672667 | 8.46 |
| GCGCTTCTTTGAGGGCATGC | 87672654 | 87672676 | 23.21 |
| GCCGGAGAAGCCGAGCACCA | 87672672 | 87672694 | 5.89 |
| GAAGAGGCGCACCGTGGTGCT | 87672682 | 87672704 | 16.35 |
| GCGGTCGAAGAGGCGCACCG | 87672689 | 87672711 | 22.00 |
| GAAAAGTCGCCGCGGTCGAAG | 87672699 | 87672721 | 6.99 |
| GCGCCGTGTAAAAGTCGCCG | 87672708 | 87672730 | 10.65 |
| GCTTTTACACGGCGCACGGAG | 87672717 | 87672739 | 13.98 |
| GCACGGAGAGGACGCGCTGC | 87672729 | 87672751 | 12.40 |
| GCGCTGCTGGCGGCCCGCG | 87672742 | 87672763 | 3.44 |
| GCTGGGTCTTGAACACCTCGC | 87672755 | 87672777 | 8.07 |
| GCATGTACTTGATCACGCCCT | 87672773 | 87672795 | 6.80 |
| GATCAAGTACATGGGGCCGGC | 87672784 | 87672806 | 3.40 |
| GTACATGGGGCCGGCAGGTG | 87672789 | 87672811 | 8.53 |
| GCAGGTGAGGGCAGCCGCGG | 87672802 | 87672824 | 1.71 |
| GGTGAGGGCAGCCGCGGCGG | 87672805 | 87672827 | 9.82 |
| GCAGCCGCGGCGGCGGGGCC | 87672812 | 87672834 | 0.08 |
| GGCGGGGCCCGGGCCGGCTC | 87672823 | 87672845 | 4.66 |
| GACGCGGCCCCGAGCCGGCCC | 87672830 | 87672852 | 4.98 |
| GCCGGCAACGCGGCCCCGAGC | 87672836 | 87672858 | 3.77 |
| GGGCCGCGTTGCCGGAAACC | 87672844 | 87672866 | 3.24 |
| GCTGCATGCGCCCAGGTTTC | 87672855 | 87672877 | 5.01 |
| GAACCTGGGCGCATGCAGCGG | 87672860 | 87672882 | 2.61 |
| GATGCAGCGGTGGGGCTGCGG | 87672871 | 87672893 | 6.75 |
| GGGGAGGCAGCTCTCGGGCG | 87672898 | 87672920 | 5.43 |
| GGCCCGGGGAGGCAGCTCTC | 87672903 | 87672925 | 2.95 |
| GCTAAACGCGTGGCCCGGGG | 87672914 | 87672936 | 2.05 |
| GAAAACAAGATGCTAAACGCG | 87672924 | 87672946 | 0.01 |
| GTTCGCTTCCGCACAGTGCGC | 87672949 | 87672971 | 0.53 |
| GCAGCCGCCCAGCGCACTGTG | 87672956 | 87672978 | 2.75 |
| GGCTGCACACCGCCGAGTCC | 87672974 | 87672996 | 0.55 |
| GTCCGAGGACAGCCTGGACT | 87672986 | 87673008 | 3.69 |
| GAGGCGTCCGAGGACAGCC | 87672992 | 87673013 | 5.09 |
| GCTGGAACTCGAGGCGTCCG | 87673001 | 87673023 | 0.75 |
| GACTTGGGAGGCTGGAACTCG | 87673010 | 87673032 | 2.20 |
| GTTCCAGCCTCCCAAGTTGAT | 87673017 | 87673039 | 6.24 |
| GACTCTAATCCTATCAACTT | 87673026 | 87673048 | 0.01 |
| GATTAGAGTCTTGTGTCACCG | 87673040 | 87673062 | 0.20 |
| GTATTGATTTGGAGGTACCCA | 87673057 | 87673079 | 1.00 |
| GCGGGCTGTTATTGATTTGG | 87673066 | 87673088 | 3.19 |
| GTCAATAACAGCCCGCAGCCT | 87673074 | 87673096 | 0.00 |
| GCAGCCCGCAGCCTAGGAGCG | 87673081 | 87673103 | 5.75 |
| GCCTAGGAGCGAGGAGTGGA | 87673090 | 87673112 | 2.43 |
| GGCAAATGAGGGTGTCTTAA | 87673127 | 87673149 | 0.56 |
| GCACAGCCTATTGGCAAATGA | 87673138 | 87673160 | 4.41 |

| | | | |
|---|---|---|---|
| GCAAAACAAAACACAGCCTAT | 87673148 | 87673170 | 0.02 |
| GAACCGTCTCGCTTGAGAACC | 87673180 | 87673202 | 0.67 |
| GTCTCGCTTGAGAACCTGGGA | 87673185 | 87673207 | 2.10 |
| GCTTCAGCAATGGTCCATCCC | 87673198 | 87673220 | 0.89 |
| GCGATACTGCCCTTCAGCAA | 87673209 | 87673231 | 1.64 |
| GGCAGTATCGCCAAGTGGTC | 87673221 | 87673243 | 1.33 |
| GTCGCCAAGTGGTCAGGACCT | 87673228 | 87673250 | 0.13 |
| GTCAGGACCTAGGATATGCCC | 87673239 | 87673261 | 0.70 |
| GTCAGCTCCAGGGCATATCCT | 87673245 | 87673267 | 1.03 |
| GATCCGGGCAGTTCAGCTCCA | 87673256 | 87673278 | 3.34 |
| GAACTGCCCGGATTTCAGTTG | 87673267 | 87673289 | 0.50 |
| GGGATCCACAACTGAAATCC | 87673272 | 87673294 | 0.08 |
| GCCGCACTCAGCAAACAGTAG | 87673292 | 87673314 | 2.18 |
| GCTGAGTGCGGTTGAGCTAGT | 87673305 | 87673327 | 1.75 |
| GCTTTTTTTTTCTGCCACCT | 87673382 | 87673404 | 0.01 |
| GCTGAGTGGTGAGAGCCAAGG | 87673396 | 87673418 | 2.04 |
| GTAGGGAGAGATGATGCTGAG | 87673411 | 87673433 | 0.66 |
| GCAGCATCATCTCTCCCTAAA | 87673416 | 87673438 | 0.07 |
| GATGAGTTCTGAAGCCTTTTA | 87673429 | 87673451 | 2.03 |
| GAAGGCTTCAGAACTCATTTG | 87673435 | 87673457 | 0.31 |
| GTCAGAACTCATTTGTGGCCT | 87673441 | 87673463 | 0.03 |
| GTGATCAAATTAATCTTCCT | 87673458 | 87673480 | 0.27 |
| GGGTGGGGGAGAGGTTATTA | 87673480 | 87673502 | 0.04 |
| GAACGGGGGTGGGTGGGGGAG | 87673489 | 87673511 | 0.01 |
| GAAAAAGCAACGGGGGTGGG | 87673497 | 87673519 | 0.01 |
| GAAAGAAAAAGCAACGGGGG | 87673501 | 87673523 | 0.62 |
| GTAATTGAAAGAAAAAGCAA | 87673507 | 87673529 | 15.28 |
| GATGTCCATTATATCATACTT | 87673535 | 87673557 | 0.02 |
| GGGCTTCTCTGTTATAGCTC | 87673603 | 87673625 | 0.50 |
| GAATTAGAACTCATGCTGGG | 87673718 | 87673740 | 2.16 |
| GAAAGAATTAGAACTCATGCT | 87673721 | 87673743 | 0.41 |
| GTTTATGCTGTTATATGCAAA | 87673741 | 87673763 | 0.01 |
| GACAGACATCGTATTTCTGCC | 87673769 | 87673791 | 0.00 |
| GATTTCTGCCTGGTCTGTGCT | 87673780 | 87673802 | 0.03 |
| GACTATTACCTAGCACAGACC | 87673787 | 87673809 | 1.68 |
| GACAAATTCAACTGGTTGTGA | 87673816 | 87673838 | 0.11 |
| GCTAACAAAAACAAATTCAAC | 87673825 | 87673847 | 0.04 |
| GTTGTGAGAGAACATTATTAA | 87673909 | 87673931 | 0.01 |
| GAAAACTAGAGGTCAGGACT | 87673936 | 87673958 | 0.47 |
| GAGAAGGAAAACTAGAGGTC | 87673942 | 87673964 | 0.61 |
| GTTTTCCTTCTCTGGATATTC | 87673954 | 87673976 | 0.00 |
| GCATGTAAATGAAGAGCTTCA | 87673984 | 87674006 | 3.82 |
| GCATCAGCACTTCGTTCCTTT | 87674018 | 87674040 | 1.88 |
| GACAATTATTTAGCAACCAAA | 87674033 | 87674055 | 3.23 |
| GCAAGGAGCTAAATGAAATGT | 87674064 | 87674086 | 0.02 |
| GTTCATTTAGCTCCTTGTCCC | 87674071 | 87674093 | 0.36 |
| GCAAAACAAACTCCAGGGACA | 87674082 | 87674104 | 0.03 |
| GCCAAGCCAAAACAAACTCCA | 87674088 | 87674110 | 1.22 |
| GTTTGTTTTGGCTTGGTTTT | 87674095 | 87674117 | 0.00 |
| GGCTTGGTTTTTGGAGACA | 87674104 | 87674125 | 1.74 |
| GACGGTCTCACTCTTTAGCCT | 87674122 | 87674144 | 0.00 |
| GCTCTTTAGCCTTGGTTGACC | 87674131 | 87674153 | 0.17 |

| | | | |
|---|---|---|---|
| GACTGAGTTCCAGGTCAACCA | 87674139 | 87674161 | 0.05 |
| GGTAAACCTACTGAGTTCC | 87674149 | 87674170 | 25.79 |
| GGCAGTGTAAGTGACAACC | 87674169 | 87674190 | 1.93 |
| GCTTACACTGCCACTGCCTCC | 87674180 | 87674202 | 1.23 |
| GCCTGTAGTCCCAGGAGGCAG | 87674189 | 87674211 | 5.80 |
| GAAAGCCTGTAGTCCCAGG | 87674195 | 87674216 | 1.65 |
| GAACAGCTCAGCTGGGCAAGG | 87674218 | 87674240 | 0.06 |
| GATTCCAGTAACAGCTCAGC | 87674227 | 87674249 | 0.56 |
| GACATTTCCTCGAATCTGTAC | 87674251 | 87674273 | 0.12 |
| GAGGAACCGGTACAGATTCG | 87674257 | 87674279 | 0.30 |
| GACTGCACTGTTGGGAGGAAC | 87674270 | 87674292 | 1.04 |
| GCTCTTCACTGCACTGTTGGG | 87674276 | 87674298 | 1.85 |
| GCAGTGAAGAGTAAGCTCT | 87674288 | 87674309 | 0.34 |
| GTAAGCTCTTGGCAGCTTTC | 87674298 | 87674320 | 11.83 |
| GCTTTCTGGATCCCAGCCGA | 87674312 | 87674334 | 1.46 |
| GGATCCCAGCCGATGGGCGT | 87674319 | 87674341 | 6.30 |
| GAACCCTACCCACGCCCAT | 87674328 | 87674349 | 3.32 |
| GGGCGTGGGTAGGGTTCTGT | 87674333 | 87674355 | 0.35 |
| GTTTCTGCAGCTCGCATCTGG | 87674367 | 87674389 | 0.05 |
| GCAGGTAAAATGTCAGTAAA | 87674393 | 87674415 | 2.02 |
| GACATTTTACCTGCATGATT | 87674402 | 87674424 | 3.60 |
| GTTACATGACCCCAATCATGC | 87674411 | 87674433 | 0.02 |
| GTTATTCTAAAGCCCTGATC | 87674440 | 87674462 | 0.01 |
| GCAGGAGGCTCTCCCTGATC | 87674453 | 87674475 | 0.97 |
| GCCTCCTGCCTGAGCCTCCC | 87674467 | 87674489 | 0.03 |
| GCATCTGAACCTGGGAGGCTC | 87674475 | 87674497 | 0.02 |
| GAATTGCAGCATCTGAACCT | 87674484 | 87674506 | 3.02 |
| GTAAATATGAAGATGCATGCA | 87674506 | 87674528 | 0.02 |
| GCATCTTCATATTTATTAC | 87674514 | 87674535 | 0.02 |
| GGGACATAGGAGAGAAGGGT | 87674538 | 87674560 | 0.01 |
| GAAGTGGGACATAGGAGAGA | 87674543 | 87674565 | 0.04 |
| GGGCCAGGAAGTGGGACAT | 87674551 | 87674572 | 2.22 |
| GTCCCACTTCCTGGCCCAGT | 87674556 | 87674578 | 0.42 |
| GGCCCAGTTGGATGCACATA | 87674568 | 87674590 | 0.19 |
| GAACCCCTATGTGCATCCAAC | 87674571 | 87674593 | 2.09 |
| GAGTCTCTTGAGGATGACA | 87674594 | 87674615 | 0.01 |
| GTGCACACAAGAGTCTCTTG | 87674603 | 87674625 | 0.25 |
| GTGAATCTGTCCCTTCTGTG | 87674626 | 87674648 | 0.14 |
| GTCTGTCCCTTCTGTGGGGTG | 87674631 | 87674653 | 0.01 |
| GCTTACCCACACCCCACAGAA | 87674636 | 87674658 | 0.04 |
| GAAAGTCCTGTTTCACAGACA | 87674690 | 87674712 | 0.26 |
| GAGGGCCTTGTCTGTGAAAC | 87674695 | 87674717 | 3.74 |
| GCCCTCAGATGCTTGTCAGC | 87674712 | 87674734 | 4.63 |
| GCTTGTCAGCAGGTGTGCA | 87674722 | 87674743 | 0.01 |
| GGTGTGCACGGCCTCTCAGT | 87674733 | 87674755 | 0.79 |
| GCACATGTGTGACCCACTGAG | 87674744 | 87674766 | 1.74 |
| GTGCTGCCTTCTAGAGAAA | 87674764 | 87674785 | 0.02 |
| GAAGGGGCCATTTCTCTAGA | 87674770 | 87674792 | 0.01 |
| GAAATGGCCCCTTCCTTCAG | 87674779 | 87674801 | 1.20 |
| GTTGGTTATCCTCTGAAGGAA | 87674787 | 87674809 | 0.01 |
| GAAAGTTTGGTTATCCTCTGA | 87674792 | 87674814 | 0.58 |
| GACCCATCTCAAAAAAAGTT | 87674806 | 87674828 | 0.02 |

| | | | |
|---|---|---|---|
| GTTTTTTGAGATGGGTCTACA | 87674813 | 87674835 | 0.01 |
| GCTACATGGTGAAGCACACGA | 87674828 | 87674850 | 0.41 |
| GTGAAGCACACGATGGCCTC | 87674835 | 87674857 | 0.00 |
| GGCTAAGGTCTGAAGCCAG | 87674851 | 87674872 | 0.01 |
| GCTTCAGACCTTAGCCTCCC | 87674857 | 87674879 | 0.01 |
| GGTACCCACCAGGGAGGCTA | 87674865 | 87674887 | 0.43 |
| GTAGGTGAGGTGGTACCCACC | 87674875 | 87674897 | 0.01 |
| GGGTACCACCTCACCTATCT | 87674881 | 87674903 | 0.04 |
| GACTTCCCAGATAGGTGAGG | 87674886 | 87674908 | 0.54 |
| GAATACAGAGACTTCCCAGAT | 87674894 | 87674916 | 0.43 |
| GCTCTGTATTTCTGTAGTGCT | 87674908 | 87674930 | 0.01 |
| GTAGTGCTAGGGATAGAAGA | 87674920 | 87674942 | 2.45 |
| GCTCATTTACCTCTCCGCTCC | 87674950 | 87674972 | 0.22 |
| GTCTAAGACCCAGGAGCGGAG | 87674958 | 87674980 | 0.01 |
| GTACTGTCTAAGACCCAGGAG | 87674963 | 87674985 | 1.52 |
| GACACATTTAATAATGAGTTC | 87674986 | 87675008 | 1.37 |
| GTTCTTGGTCTTTTTACTAAC | 87675005 | 87675027 | 0.02 |
| GAACCCAGAGAACTGTTTCT | 87675021 | 87675043 | 0.02 |
| GACACAGCAGTGGTATCTATC | 87675053 | 87675075 | 2.07 |
| GATAACTACAGTACACAGCAG | 87675064 | 87675086 | 0.28 |
| GAAGAACAGAGAAGCCAGATG | 87675091 | 87675113 | 0.00 |
| GAAGGTATTCGCCACCACATC | 87675104 | 87675126 | 0.01 |
| GTACCCGTGTGCTGGGATTAA | 87675123 | 87675145 | 0.08 |
| GATCCACCTACCCGTGTGCT | 87675131 | 87675153 | 1.72 |
| GCTCTTCTAGGTGGACCAGGC | 87675167 | 87675189 | 0.01 |
| GCTGTCCTAAACTCTTCTAGG | 87675177 | 87675199 | 8.98 |
| GAAGAGTTTAGGACAGCCAA | 87675184 | 87675206 | 0.02 |
| GTGGGGACGGTGTTGAGACA | 87675220 | 87675242 | 0.02 |
| GTTGGGGGTGGAGGGTGGGGA | 87675233 | 87675255 | 0.00 |
| GCAGCGTTGGGGGTGGAGGGT | 87675238 | 87675260 | 0.16 |
| GAGTACAGCGTTGGGGGTGG | 87675243 | 87675265 | 0.01 |
| GTGTGGGAGTACAGCGTTGG | 87675249 | 87675271 | 0.00 |
| GTTGGGGGGGGGGGATGGTG | 87675266 | 87675288 | 0.03 |
| GTTTGAAGGATTTGGGGGGG | 87675277 | 87675299 | 0.03 |
| GTTTTTCAGTTTGAAGGATT | 87675285 | 87675307 | 0.00 |
| GTTAGTGTTTTTCAGTTTGA | 87675291 | 87675313 | 0.01 |
| GAAAAACACTAACTACAAAA | 87675301 | 87675323 | 0.72 |
| GCTAACTACAAAAAGGAACAG | 87675309 | 87675331 | 0.90 |
| GAGGGGCTTGTACATCTGAG | 87675328 | 87675350 | 2.75 |
| GAGAGGGCTTCGTGAAGTG | 87675345 | 87675366 | 0.81 |
| GAAGCACTCACTGCATTAA | 87675369 | 87675390 | 0.17 |
| GCAGCCCTGTCCTGTCCGGA | 87675392 | 87675414 | 0.02 |
| GAAGAACCATCCGGACAGGAC | 87675397 | 87675419 | 1.96 |
| GATGGTTCTTGCACGTGCTG | 87675410 | 87675432 | 1.98 |
| GTTCTTGCACGTGCTGTGGGT | 87675415 | 87675437 | 3.99 |
| GCTGGATGAATTCTGGGAGAG | 87675443 | 87675465 | 0.00 |
| GAAGATGGTCTGGATGAATTC | 87675451 | 87675473 | 3.28 |
| GCACGCTGAGGGAAGATGGTC | 87675462 | 87675484 | 0.42 |
| GAATATCACGCTGAGGGAAGA | 87675467 | 87675489 | 0.12 |
| GTAAAAAAATATCACGCTGA | 87675474 | 87675496 | 0.16 |
| GAACTAAAATGCAATCTTATT | 87675496 | 87675518 | 0.02 |
| GCGTCTTAGTGTGTAGCCT | 87675574 | 87675595 | 0.00 |

| | | | |
|---|---|---|---|
| GTGTGTAGCCTTGGCTAGCT | 87675582 | 87675604 | 0.34 |
| GGTAAGTTCCCAGCTAGCCA | 87675590 | 87675612 | 3.57 |
| GCAGGGGGGTCTGTGAGTCA | 87675611 | 87675633 | 0.02 |
| GGGAAGACAGAGAGGCAGGG | 87675625 | 87675647 | 0.33 |
| GCTAACACTGGGAAGACAGAG | 87675633 | 87675655 | 0.03 |
| GAGGGAGAATGCCCTAACAC | 87675646 | 87675668 | 0.48 |
| GACGTCTAAGAGGTCAGTCTG | 87675665 | 87675687 | 0.81 |
| GCCAGTACATAAACGTCTAAG | 87675676 | 87675698 | 0.02 |
| GTCTGTACATGTATGTATGCC | 87675731 | 87675753 | 0.01 |
| GGCTTTCTTCTGACCTCCCT | 87675756 | 87675778 | 0.01 |
| GAAGCCTAAAAATAGATTAAG | 87675774 | 87675796 | 0.03 |
| GAATAGATTAAGTGGCCATGT | 87675783 | 87675805 | 0.82 |
| GTTAAGTGGCCATGTAGGTGC | 87675789 | 87675811 | 1.45 |
| GCAATTGTCCCAGCACCTACA | 87675797 | 87675819 | 0.61 |
| GTGAGCCACCAGCTGGTTGC | 87675938 | 87675960 | 0.01 |
| GAAATCCCAGCAACCAGCTGG | 87675943 | 87675965 | 0.02 |
| GCTATCTCGCCAGCCCGAAAA | 87676012 | 87676034 | 0.55 |
| GGAATAAAAACTCCATTTTC | 87676024 | 87676046 | 0.91 |
| GGAGTTTTTATTCCAGTGGA | 87676033 | 87676055 | 0.02 |
| GTTTTATTCCAGTGGATGGTG | 87676038 | 87676060 | 0.01 |
| GAAAATCACCACACCATCCAC | 87676045 | 87676067 | 1.31 |
| GTGGTGATTTTATCTGTGTG | 87676057 | 87676079 | 1.30 |
| GATTTTATCTGTGTGTGGAA | 87676062 | 87676084 | 0.02 |
| GTTGACTTTATGTGTATGGA | 87676084 | 87676106 | 0.83 |
| GCTCTTCTAGATTCATGCACA | 87676128 | 87676150 | 1.76 |
| GTAGAAGAGGGCAGAGTTCCT | 87676143 | 87676165 | 1.36 |
| GCAGGAAGTATTTCAGTTCCA | 87676160 | 87676182 | 0.45 |
| GAAATACTTCCTGCTAAATC | 87676170 | 87676192 | 0.36 |
| GGATGAGACCCTGATTTAGC | 87676179 | 87676201 | 0.75 |
| GTCAAAATGCAAATAGTACCA | 87676200 | 87676222 | 0.24 |
| GTTAACATGATTAGGACAGAA | 87676249 | 87676271 | 0.01 |
| GAAGAAGCTGTTAACATGATT | 87676258 | 87676280 | 2.02 |
| GCACTTTGGAATCTAGAGGAC | 87676284 | 87676306 | 1.76 |
| GAATAGCACTTTGGAATCTAG | 87676289 | 87676311 | 2.83 |
| GATGAGGGAACAATAGCACTT | 87676299 | 87676321 | 0.75 |
| GAGACATGTGAGCTGTCATG | 87676316 | 87676338 | 2.25 |
| GGGGGGCGTATAATAATTCT | 87676338 | 87676360 | 0.01 |
| GATACGCCCCCCCCCCCAAG | 87676350 | 87676372 | 0.08 |
| GATCCTGCTCCTCTTGGGGG | 87676359 | 87676381 | 1.38 |
| GTGATCCTGCTCCTCTTGGG | 87676361 | 87676383 | 2.17 |
| GTTCTCATCTATATCTCAACT | 87676385 | 87676407 | 1.40 |
| GAGATATAGATGAGAAAGTT | 87676392 | 87676414 | 0.01 |
| GTATGCGTATCGTGTGTGTC | 87676455 | 87676477 | 0.22 |
| GTGTGTCTGGTCCCCACAA | 87676468 | 87676489 | 0.01 |
| GTCCCCACAAAGGCCAGAAG | 87676477 | 87676499 | 0.01 |
| GATCTGTAGCTCTGGTTTTAG | 87676509 | 87676531 | 0.48 |
| GCAAGTGTCCTTAACCACT | 87676588 | 87676609 | 0.04 |
| GTAACCACTAGGCCATCTCTC | 87676599 | 87676621 | 0.04 |
| GTTTCCAGGGGCCGGAGAGA | 87676610 | 87676632 | 0.14 |
| GAAGCAAGAGTTTCCAGGGGC | 87676618 | 87676640 | 0.52 |
| GAGTCTCGCTGTGTACTGCC | 87676710 | 87676732 | 1.32 |
| GTCGCTGTGTACTGCCTGGCC | 87676715 | 87676737 | 0.37 |

| | | | |
|---|---|---|---|
| GTACTGCCTGGCCTGGACC | 87676722 | 87676743 | 0.53 |
| GCTAGATCCAGGTCCAGGCC | 87676728 | 87676750 | 0.52 |
| GCTTAGGCTAGATCCAGGTCC | 87676733 | 87676755 | 0.51 |
| GGATCTAGCCTAAGCCAGCT | 87676742 | 87676764 | 0.04 |
| GTGAAAAGCCAAGCTGGCTT | 87676750 | 87676772 | 0.55 |
| GCTTGGCTTTTCACTTATAG | 87676759 | 87676781 | 0.01 |
| GCAAAGACACCCAGCTTCCTG | 87676806 | 87676828 | 0.82 |
| GAACAGAGTCCTCAGGAAGCT | 87676814 | 87676836 | 2.29 |
| GCTACAGTCAACAGAGTCCTC | 87676822 | 87676844 | 3.39 |
| GGAAAGTAGATAACCATAGA | 87676844 | 87676866 | 0.01 |
| GAACCATAGAGGGAGGCAGTC | 87676855 | 87676877 | 0.04 |
| GATCACACCCATGAGAGAATT | 87676889 | 87676911 | 3.31 |
| GCTAAAACCAAATTCTCTCAT | 87676895 | 87676917 | 0.55 |
| GTTAGAATCTTCTCAACACAC | 87676914 | 87676936 | 4.50 |
| GGGAACCCTGAGCATTTAC | 87676935 | 87676956 | 3.03 |
| GAAGAAACCTGTAAATGCTC | 87676941 | 87676963 | 5.39 |
| GTGAAGCCTAGCAATGCTG | 87676964 | 87676985 | 3.29 |
| GCCAATTTCCTCAGCATTGCT | 87676970 | 87676992 | 0.02 |
| GCAGAACTGGCTTCTGTGTAC | 87677014 | 87677036 | 0.02 |
| GACACAGAAGCCAGTTCTGTG | 87677019 | 87677041 | 1.77 |
| GTTGCTCCAACCACACAGAAC | 87677028 | 87677050 | 0.50 |
| GTGGTTGGAGCAAGCTCCC | 87677038 | 87677059 | 1.57 |
| GGAGCAAGCTCCCTGGTGAT | 87677044 | 87677066 | 3.16 |
| GCACACAGTTCCCTATCACCA | 87677054 | 87677076 | 3.30 |
| GGGAACTGTGTGTGTATCCA | 87677065 | 87677087 | 2.38 |
| GTGTGTATCCATGGTGATAA | 87677074 | 87677096 | 0.80 |
| GATCCATGGTGATAAGGGAAC | 87677080 | 87677102 | 0.19 |
| GATCCATGGTGATTGGGGAAC | 87677252 | 87677274 | 0.07 |
| GTATTGTGTGAGTGTATAAA | 87677307 | 87677329 | 5.93 |
| GAAACTCCCAGCCTGTTAC | 87677367 | 87677388 | 0.87 |
| GCTGCTTACCAGTAACAGGC | 87677374 | 87677396 | 0.41 |
| GTTACTGGTAAGCAGCTGGCT | 87677382 | 87677404 | 0.01 |
| GGCTAGGGACAGTGCTAAAA | 87677398 | 87677420 | 3.68 |
| GCCACTTGTAAACAGAACTCA | 87677439 | 87677461 | 0.46 |
| GTTTACAAGTGGAGTATGACT | 87677451 | 87677473 | 1.90 |
| GTTACTGAGAAGCCAACCC | 87677490 | 87677511 | 0.95 |
| GCATGTGTGACAGCCTGGGT | 87677502 | 87677524 | 1.71 |
| GGCTGTCACACATGCTGGGA | 87677510 | 87677532 | 0.01 |
| GCTCTGAAACTGCTCAGAGCT | 87677553 | 87677575 | 0.01 |
| GAAAACAAAGTACATTTACAA | 87677577 | 87677599 | 1.27 |
| GGCTTGCGCCACCACGCCC | 87677756 | 87677777 | 0.07 |
| GAATTTACCAGGCCGGGGCG | 87677767 | 87677789 | 1.35 |
| GAAAAAGAATTTACCAGGCCG | 87677772 | 87677794 | 4.77 |
| GATGAATAAAAAGAATTTACC | 87677778 | 87677800 | 0.40 |
| GTCTCTTGTGTGTTATGTATA | 87677800 | 87677822 | 0.03 |
| GATGGCTGAACACAATGCAGT | 87677819 | 87677841 | 2.64 |
| GACACAATGCAGTAGGGATGC | 87677827 | 87677849 | 1.87 |
| GCAGTAGGGATGCAGGAGTC | 87677834 | 87677856 | 0.16 |
| GGCCTATTCTTCTACCTGAG | 87677855 | 87677877 | 0.71 |
| GTTCAAACACTCAGACCTCTC | 87677869 | 87677891 | 15.02 |
| GCTGAGTGTTTGAACTCCATT | 87677879 | 87677901 | 0.93 |
| GTATGAATAAAAGCTTCCTAA | 87677894 | 87677916 | 3.78 |

| | | | |
|---|---|---|---|
| GACAGAGAAGATACGTTATCA | 87677924 | 87677946 | 3.37 |
| GACGTATCTTCTCTGTAGTCC | 87677932 | 87677954 | 1.44 |
| GGAGGAGTTTGAGGGCAGCC | 87677950 | 87677972 | 0.82 |
| GTCAGATCAGGAGGAGTTTGA | 87677958 | 87677980 | 1.73 |
| GGGTATAACTTCAGATCAGG | 87677968 | 87677990 | 0.43 |
| GCCACTTGGAAGTCTGAGGC | 87677990 | 87678012 | 3.44 |
| GATATCAGCAGTCTAGCCACT | 87678004 | 87678026 | 1.84 |
| GACGGGGCATGTATGTACAAC | 87678029 | 87678051 | 0.17 |
| GAACACTACGTATGTACAACG | 87678046 | 87678068 | 5.04 |
| GTTGTTCATTCAGCAGTTTGT | 87678068 | 87678090 | 2.22 |
| GTCAGCAGTTTGTTGGAAGGT | 87678076 | 87678098 | 2.49 |
| GTTGGAAGGTTGGCTTGCTG | 87678086 | 87678108 | 3.01 |
| GCTGAGGAAATGCAAGTTT | 87678102 | 87678123 | 0.16 |
| GATGTTAAAGATGAGGCTGTG | 87678154 | 87678176 | 0.87 |
| GAAATTTAAATGTTAAAGATG | 87678162 | 87678184 | 0.69 |
| GTTTATTTGTTGTTTATTTC | 87678185 | 87678207 | 0.01 |
| GCTAAGCACAACACTCTGCA | 87678216 | 87678238 | 12.44 |
| GTTTCGTGAAAGATCTTCTTC | 87678256 | 87678278 | 9.15 |
| GTATAAACTTCAACTCGATAC | 87678283 | 87678305 | 9.23 |
| GAAGTTTATAAGAATAAAGC | 87678296 | 87678318 | 2.53 |
| GTAAGAATAAAGCTGGAAATA | 87678304 | 87678326 | 8.74 |
| GCTGGAAATAAGGCGTCTA | 87678314 | 87678335 | 11.22 |
| GGCGTCTAAGGAGAATGAG | 87678325 | 87678346 | 5.20 |
| GTAAGGAGAATGAGTGGTATC | 87678331 | 87678353 | 3.79 |
| GAGTGGTATCTGGCATTTA | 87678341 | 87678362 | 10.13 |
| GTAAGGTTGTTATTTTCCTTT | 87678358 | 87678380 | 0.00 |
| GTTCTAAGACAAACATCCAAA | 87678373 | 87678395 | 0.01 |
| GCATGTTTTACTATATTAACT | 87678410 | 87678432 | 6.02 |
| GAACACTATCAAAATTTCTCA | 87678449 | 87678471 | 0.89 |
| GGAACAGTGAGACAAGGTT | 87678546 | 87678567 | 5.48 |
| GATTTCAGGAACAGTGAGACA | 87678551 | 87678573 | 0.07 |
| GATCATCTAATGTTGTATTTC | 87678566 | 87678588 | 0.64 |
| GTACAACATTAGATGATAACC | 87678573 | 87678595 | 6.19 |
| GAAATTATAACATTTATGTCC | 87678591 | 87678613 | 0.01 |
| GAAAAAATTACTTATGTGTAC | 87678646 | 87678668 | 0.02 |
| GACAGGCATGCAAGTGGTACA | 87678683 | 87678705 | 0.67 |
| GTCTAGCAACAGGCATGCAAG | 87678690 | 87678712 | 3.53 |
| GCATGCCTGTTGCTAGAGCG | 87678696 | 87678718 | 4.60 |
| GGTCTCCTCGCTCTAGCAAC | 87678701 | 87678723 | 0.42 |
| GAGCGAGGAGACCAGAGGAG | 87678711 | 87678733 | 0.41 |
| GAGACCAGAGGAGAGGGCCT | 87678718 | 87678740 | 0.83 |
| GGAGAGGGCCTTGGATCCCT | 87678727 | 87678749 | 0.11 |
| GGCCTTGGATCCCTTGGGGT | 87678733 | 87678755 | 0.94 |
| GAAGTCTAACACCAACCCCAA | 87678743 | 87678765 | 1.43 |
| GCTGGGTGCTCTTCTAGAATC | 87678771 | 87678793 | 0.93 |
| GCAGGCAGAGATTAAGAACAC | 87678790 | 87678812 | 1.96 |
| GAAAACTGGAGGCAGAAAGAC | 87678809 | 87678831 | 6.24 |
| GAACTAAAAACAAAAAACTGG | 87678821 | 87678843 | 7.16 |
| GTTTTTTTCTTTTTCTTTGG | 87678841 | 87678863 | 0.01 |
| GTCTCCCTGTGTCCTCAGGG | 87678868 | 87678890 | 6.76 |
| GTAGGCCACCCTGAGGACACA | 87678872 | 87678894 | 2.63 |
| GAAGAATCCTAGGCCACCCTG | 87678880 | 87678902 | 1.37 |

| | | | |
|---|---|---|---|
| GCAAGGATTGCCAAGAATCCT | 87678891 | 87678913 | 1.06 |
| GTCTTGGCAATCCTTGGAAAA | 87678899 | 87678921 | 4.49 |
| GATCAAGACAGCCATTTTCCA | 87678909 | 87678931 | 10.45 |
| GAAATGGCTGTCTTGATGTGC | 87678916 | 87678938 | 2.51 |
| GCAGGCTGATCTAGACCAGCC | 87678978 | 87679000 | 3.58 |
| GACCAGCCTGGCCTCTAGAT | 87678990 | 87679012 | 0.43 |
| GTTTCTCCCCATCTAGAGGCC | 87678996 | 87679018 | 0.09 |
| GCAGGGTTTCTCCCCATCTAG | 87679001 | 87679023 | 0.06 |
| GTTGTTTTTGGCTTTCAAAAC | 87679020 | 87679042 | 0.01 |
| GTCTTTTTGTTGGTTGTTTT | 87679033 | 87679055 | 0.01 |
| GATTTTTCTCTGTCTTTTTGT | 87679043 | 87679065 | 0.00 |
| GATGCCTAACTCAGCTTCTCA | 87679064 | 87679086 | 3.46 |
| GAACTCAGCTTCTCAAGGTGA | 87679070 | 87679092 | 0.48 |
| GTTCTCAAGGTGAGGGATTAA | 87679078 | 87679100 | 6.05 |
| GTTTTACTTTTCTTAGAAC | 87679115 | 87679136 | 0.01 |
| GTTCTTAGAACAGGCTCAGTC | 87679124 | 87679146 | 0.41 |
| GCTTCAGTGCTGGAGAAGTGG | 87679164 | 87679186 | 0.01 |
| GTCACTTCAGTGCTGGAGAAG | 87679167 | 87679189 | 4.90 |
| GTCTGTAATCACTTCAGTGC | 87679175 | 87679197 | 0.44 |
| GAAGGCAAAGAAAGGCAAGTG | 87679206 | 87679228 | 1.99 |
| GATATAGAAAAGGCAAAGAA | 87679215 | 87679237 | 3.23 |
| GACATACTTCAGATATAGAAA | 87679225 | 87679247 | 7.84 |
| GATATCTGAAGTATGTCAGAC | 87679233 | 87679255 | 2.53 |
| GTCCCTAATGTCTCATTTAT | 87679263 | 87679285 | 6.40 |
| GCAGTCCACACCTTTAGTGAG | 87679309 | 87679331 | 1.14 |
| GACGCTATACCGCTCACTAA | 87679318 | 87679340 | 1.63 |
| GGTATAGCGTCACACAAGCC | 87679330 | 87679352 | 1.42 |
| GCACAAGCCTGGAGGGTGCTT | 87679342 | 87679364 | 3.19 |
| GCAAAGACCAAAGCACCCTCC | 87679348 | 87679370 | 2.65 |
| GTTTGAGATAGCATCTTCCTG | 87679367 | 87679389 | 0.08 |
| GCATCTTCCTGTGGCCCACGC | 87679377 | 87679399 | 2.06 |
| GTCAAGGCCAGCGTGGGCCAC | 87679383 | 87679405 | 4.71 |
| GGTCCAATCAAGGCCAGCGT | 87679390 | 87679412 | 0.42 |
| GAATGTCAGGGTCCAATCA | 87679400 | 87679421 | 1.53 |
| GAGACTGAGACAGAATGTCA | 87679411 | 87679433 | 1.62 |
| GTCTCAGTCTCTTGTGTTCT | 87679423 | 87679445 | 0.24 |
| GGGAGGCCTGAAAATCAGTT | 87679443 | 87679465 | 2.59 |
| GATTCTGCCAAACTGATTTTC | 87679449 | 87679471 | 1.39 |
| GGCAGAATAGTTTCTCTGTA | 87679464 | 87679486 | 4.59 |
| GATAGTTTCTCTGTATGGTCC | 87679470 | 87679492 | 4.79 |
| GGCAGATCTCTGTGGTTTG | 87679526 | 87679547 | 1.61 |
| GCCTAGCACTCAAGAGGCAGA | 87679549 | 87679571 | 0.08 |
| GCCAGAGACGGGCAATGGTGG | 87679584 | 87679606 | 0.65 |
| GATTTCTCCAGAGACGGGCAA | 87679590 | 87679612 | 6.43 |
| GTTACTGATTTCTCCAGAGA | 87679597 | 87679619 | 1.92 |
| GAGAAATCAGTAACTGCAAA | 87679606 | 87679628 | 0.95 |
| GTAACTGCAAAAGGAAGAGA | 87679615 | 87679637 | 3.90 |
| GATAATTGAAAATAGACTGAC | 87679658 | 87679680 | 1.61 |
| GAAAACAAAACCAAAATTAAT | 87679708 | 87679730 | 0.04 |
| GAATAATTATCCAATTAATTT | 87679717 | 87679739 | 0.01 |
| GAAAAACAAAAACAGTTTTTA | 87679770 | 87679792 | 0.01 |
| GAACTGTTTTTGTTTTTGCTC | 87679777 | 87679799 | 0.01 |

| | | | |
|---|---|---|---|
| GTTTTTGCTCAGGCTTCTCC | 87679787 | 87679809 | 5.69 |
| GTCAAACTGAGAAAGATTGCC | 87679805 | 87679827 | 3.71 |
| GCAGTTTGAAGACATCCTGTT | 87679820 | 87679842 | 11.83 |
| GACATGTCATTGTTACCAAAC | 87679834 | 87679856 | 10.19 |
| GAATGACATGTCAGCTTCCGT | 87679847 | 87679869 | 23.77 |
| GTCAGCTTCCGTTGGCGTTA | 87679855 | 87679877 | 7.11 |
| GTTTAATACCCATAACGCCAA | 87679863 | 87679885 | 2.93 |
| GTATGGGTATTAAAATGGCCG | 87679873 | 87679895 | 5.16 |
| GATTAAAATGGCCGTGGTTGA | 87679880 | 87679902 | 4.23 |
| GATGTCTTTGACCATCAACCA | 87679890 | 87679912 | 6.06 |
| GTTGATGGTCAAAGACATGT | 87679895 | 87679917 | 7.26 |
| GGTCAAAGACATGTTGGAGT | 87679901 | 87679923 | 2.67 |
| GACATGTTGGAGTTGGGTATG | 87679909 | 87679931 | 5.59 |
| GGTATGTGGATTCCACCCAG | 87679923 | 87679945 | 6.85 |
| GATTCCACCCAGAGGAAGCT | 87679931 | 87679953 | 3.46 |
| GACACAAGCCTAGCTTCCTCT | 87679938 | 87679960 | 6.49 |
| GTTGGAGAACTGATCATTCTC | 87679967 | 87679989 | 6.32 |
| GATCAGTTCTCCAATCTCG | 87679976 | 87679997 | 3.64 |
| GAATCAGAAGAGCCTCGAGAT | 87679986 | 87680008 | 17.60 |
| GAGGCTCTTCTGATTCAGAT | 87679994 | 87680016 | 2.50 |
| GTCTGATTCAGATTGGACCAA | 87680002 | 87680024 | 12.53 |
| GCCTGGTAAAACGCATTCCTT | 87680018 | 87680040 | 3.35 |
| GTTACCAGGAGGAGAGACTAC | 87680033 | 87680055 | 5.20 |
| GGAGAGACTACTGGAGACAT | 87680042 | 87680064 | 3.74 |
| GCTGGAGACATGGGGAAACTG | 87680052 | 87680074 | 11.99 |
| GGGGAAACTGAGGCAGGTAA | 87680062 | 87680084 | 5.17 |
| GCAGGTAAGGGCATTCAGTCT | 87680075 | 87680097 | 18.90 |
| GGAAGGGGTATTTTACATGA | 87680103 | 87680125 | 0.43 |
| GTTCAAGGAAATACAGGGGA | 87680120 | 87680142 | 0.93 |
| GTTCTAGAGGTCCTAGGTTCA | 87680135 | 87680157 | 0.16 |
| GGACCTCTAGAATACAAGGC | 87680146 | 87680168 | 1.02 |
| GCACCAGCCTTGTATTCTAG | 87680149 | 87680171 | 0.83 |
| GGTGCTCTACCCCTGAGCTC | 87680167 | 87680189 | 2.52 |
| GACAGCTCAGTGCTAGAGCCC | 87680561 | 87680583 | 0.74 |
| GAAATGAAAAGCATCATCAGC | 87680588 | 87680610 | 0.99 |
| GATGATGCTTTTCATTTATC | 87680594 | 87680616 | 0.01 |
| GATTTTAATTAGAATTAAAAC | 87680626 | 87680648 | 0.07 |
| GTAAAATAATGTTTTATTTTG | 87680643 | 87680665 | 0.02 |
| GTTCTTAACTTTGAAAGAACT | 87680676 | 87680698 | 0.24 |
| GTTTTATAAGACTTGCTGAGG | 87680725 | 87680747 | 0.34 |
| GTTCCCAGCAACATCATGG | 87680797 | 87680818 | 0.01 |
| GAAGATGGCATCAGACAGAAG | 87680849 | 87680871 | 1.24 |
| GTTACTATAACTATTCTCT | 87680971 | 87680992 | 0.33 |
| GTAGTAACTATGAACTGTATT | 87680986 | 87681008 | 0.02 |
| GCTGTATTTGGCATGTTTATC | 87680999 | 87681021 | 0.43 |
| GATATTTATTTCGTATTTTGT | 87681037 | 87681059 | 0.01 |
| GTCGTATTTTGTTGGGGATCT | 87681046 | 87681068 | 0.02 |
| GTTTTGTTGGGGATCTTGGCC | 87681051 | 87681073 | 0.02 |
| GTACCTGCTTAGCACACGCC | 87681069 | 87681091 | 2.60 |
| GTAAAGAAAAAATTCTAGGTTT | 87681113 | 87681135 | 0.02 |
| GAAACTTAAAGAAAAAATTCT | 87681119 | 87681141 | 2.89 |
| GATTGTCTTACTTGACAGCCC | 87681161 | 87681183 | 0.09 |

| | | | |
|---|---|---|---|
| GCCTCAGCAGCCTGAGTTCT | 87681209 | 87681231 | 0.00 |
| GCTCGAGTAATCCTAGAACTC | 87681219 | 87681241 | 0.29 |
| GTACTCGAGTGCTACTACAGA | 87681234 | 87681256 | 0.19 |
| GCTAAATTTTAAAAGGGAAAC | 87681259 | 87681281 | 0.01 |
| GTAAAGGCTAAATTTTAAAA | 87681266 | 87681288 | 0.01 |
| GATAGACAAAAAGTTCTGTAA | 87681282 | 87681304 | 1.55 |
| GTTTAGATTTGCAGCCAAACC | 87681331 | 87681353 | 0.01 |
| GTTTAAGTTCAAGGCCAGGTT | 87681344 | 87681366 | 0.01 |
| GTCTTTTTTAAGTTCAAGGCC | 87681349 | 87681371 | 3.15 |
| GAATTTTCTTTTTTAAGTTCA | 87681354 | 87681376 | 0.10 |
| GAAAATTAGATTTATTTTCCC | 87681371 | 87681393 | 0.03 |
| GATTTATTTTCCCCGGAGAC | 87681378 | 87681400 | 0.15 |
| GACAGTGAGGCCCTGTCTCCG | 87681388 | 87681410 | 0.75 |
| GACAGCCACAGCTACACAGTG | 87681402 | 87681424 | 4.02 |
| GCAGCTTTAATTCCAAGACTT | 87681484 | 87681506 | 0.50 |
| GCACAAATTTTTAGCTGGCTA | 87681514 | 87681536 | 0.01 |
| GAACACTCACAAATTTTTAGC | 87681520 | 87681542 | 0.02 |
| GATGTGTACCGCATGAATGTC | 87681559 | 87681581 | 0.87 |
| GCATGAATGTCTGGCATCCA | 87681568 | 87681590 | 0.28 |
| GTCTGGCATCCATGGAAGTC | 87681576 | 87681598 | 0.03 |
| GCATCCATGGAAGTCAGGAGA | 87681582 | 87681604 | 0.15 |
| GGAAGTCAGGAGAAGGTATT | 87681589 | 87681611 | 0.03 |
| GGAGAAGGTATTGGGTGTCT | 87681597 | 87681619 | 1.33 |
| GAACTAAAGTTACAGATGGT | 87681619 | 87681641 | 0.02 |
| GATGGTTGGGAGCCCCCAGG | 87681633 | 87681655 | 0.82 |
| GGGAGCCCCCAGGTGGGTGC | 87681640 | 87681662 | 4.14 |
| GCTCTCCCAGCACCCACCTGG | 87681645 | 87681667 | 0.09 |
| GCAGCACTTGGAGATGGCT | 87681687 | 87681708 | 0.01 |
| GTATCTGCAGCACTTGGAGA | 87681692 | 87681714 | 0.60 |
| GTGCTGCAGATACTTTTTAA | 87681702 | 87681724 | 0.03 |
| GATCTGTCTGCTAACTCAC | 87681727 | 87681748 | 2.28 |
| GCTAACTCACTGGCCATGC | 87681736 | 87681757 | 4.37 |
| GCTGTTAGAAGCAGGCCAGCA | 87681749 | 87681771 | 0.22 |
| GAGGACTGCCTGTTAGAAGC | 87681758 | 87681780 | 1.79 |
| GCCTAGGGAGGAAGAAACATG | 87681777 | 87681799 | 3.20 |
| GTTTCTTCCTCCCTAGGGCT | 87681783 | 87681805 | 0.21 |
| GTCCCTGTAATCCTAGCCCTA | 87681793 | 87681815 | 0.10 |
| GCTACAAGGCTGTTATCAGGT | 87681827 | 87681849 | 1.62 |
| GATAACAGCCTTGTAGTACT | 87681834 | 87681856 | 0.79 |
| GTATCGGTGCCCAGTACTACA | 87681842 | 87681864 | 0.05 |
| GATAAGGTTCCCAGCATGCC | 87681861 | 87681883 | 1.50 |
| GAGAGCTTGCCTGGCATGCT | 87681870 | 87681892 | 0.07 |
| GCAGTACTAGAGAGCTTGCC | 87681879 | 87681901 | 0.01 |
| GGAAGGGGAGGCATGGCTAG | 87681907 | 87681929 | 0.19 |
| GCATGTTGAGGGAGGAAGGGG | 87681919 | 87681941 | 0.19 |
| GAGTTTGAATGCCATGTTGA | 87681931 | 87681953 | 2.21 |
| GTCTCCTCTGTTTCAGCTTCT | 87681952 | 87681974 | 4.74 |
| GCAGCTTCTTGGTTGGATTAA | 87681964 | 87681986 | 0.53 |
| GTTGAATTCAAAGTCCAGAGT | 87682050 | 87682072 | 0.42 |
| GGTTTTTTTTTTTTAATGAG | 87682072 | 87682094 | 0.02 |
| GGGTCTCACTGTGTAGATT | 87682097 | 87682118 | 0.01 |
| GCTGTGTAGATTTGGCTATCC | 87682105 | 87682127 | 0.53 |

| | | | |
|---|---|---|---|
| GGTCTTCACAAGGAGCTCC | 87682123 | 87682144 | 15.83 |
| GGTCAGCCTGGTCTTCACA | 87682132 | 87682153 | 0.96 |
| GTCTCACATCTTTGAGTTCA | 87682152 | 87682174 | 0.37 |
| GCTCAGGAGGCGGAGGTAGGT | 87682174 | 87682196 | 0.06 |
| GTCTAAGAACTCAGGAGGCGG | 87682182 | 87682204 | 1.20 |
| GTTTAAATCTAAGAACTCAGG | 87682188 | 87682210 | 0.32 |
| GATTTAAAAGTAGTTCCTGC | 87682203 | 87682225 | 0.35 |
| GTAGTTCCTGCCGGGCATGG | 87682212 | 87682234 | 0.74 |
| GCCAAGGCCACCATGCCCGGC | 87682218 | 87682240 | 1.46 |
| GATGGTGGCCTTGGGAGGCAG | 87682228 | 87682250 | 0.01 |
| GCACACACAGAGTAGTTCCTA | 87682374 | 87682396 | 0.89 |
| GCAGAGTAGTTCCTAAGGACT | 87682380 | 87682402 | 0.20 |
| GCTCACTATCCAAGTCCTT | 87682390 | 87682411 | 0.44 |
| GCTTGGATAGTGAGCAGAATT | 87682398 | 87682420 | 14.00 |
| GTGCGTGTTAGTAGTCTTCT | 87682420 | 87682442 | 0.01 |
| GTTGAGGAAATCAATGAGAAA | 87682479 | 87682501 | 0.04 |
| GAAAGATTTCAAATGACTTTG | 87682496 | 87682518 | 0.01 |
| GTTGAAATCTTTCCTATAAAC | 87682508 | 87682530 | 0.51 |
| GTCTCTGGATAACCTGTTTAT | 87682519 | 87682541 | 1.50 |
| GAAACAGGTTATCCAGAGAGG | 87682524 | 87682546 | 7.97 |
| GATCAGAATCCCTCCTCTC | 87682535 | 87682556 | 12.44 |
| GAATGTCTTTAGTGGAAAAGT | 87682573 | 87682595 | 19.56 |
| GTTCCACTAAAGACATTTATC | 87682580 | 87682602 | 2.77 |
| GACATTTATCAGGATCTCAAC | 87682591 | 87682613 | 3.27 |
| GATCTCAACCGGTTACTGAA | 87682602 | 87682624 | 8.53 |
| GCTTTTTTGCCTTTCAGTAAC | 87682610 | 87682632 | 0.11 |
| GTAGTGCTGCCCTACCAGAGA | 87682646 | 87682668 | 14.57 |
| GCTGATTCTCCATCTCTGGTA | 87682654 | 87682676 | 5.12 |
| GAGATGGAGAATCAGGTCTG | 87682662 | 87682684 | 2.21 |
| GCACTACAGTGATGTTTGTAA | 87682706 | 87682728 | 0.60 |
| GCGCACACACCCGTGCTCA | 87682769 | 87682790 | 0.01 |
| GTGGTGTGCGTCCATGAGCA | 87682779 | 87682801 | 0.09 |
| GCACACCACCGCGTGCGTG | 87682793 | 87682814 | 7.09 |
| GCCACTTCCACACGCACGCGG | 87682798 | 87682820 | 1.09 |
| GTGGAAGTGGGAACAACTTT | 87682811 | 87682833 | 1.39 |
| GGGAACAACTTTCGGGAGTT | 87682819 | 87682841 | 0.01 |
| GTTTTTTCACCGTGTGAACCC | 87682843 | 87682865 | 0.02 |
| GCTTACCCCAGGGTTCACA | 87682851 | 87682872 | 0.01 |
| GAACCCTGGGGTAAGCGCCC | 87682857 | 87682879 | 2.14 |
| GGGTAAGCGCCCAGGCTTGG | 87682865 | 87682887 | 0.20 |
| GAAGACCCTCCCACCAAGCC | 87682875 | 87682897 | 0.01 |
| GCATCTGCTGAGCCTCCTCAC | 87682897 | 87682919 | 1.54 |
| GCAGGAGTGTGGCCAGTGAGG | 87682908 | 87682930 | 0.51 |
| GCAGTGAATCTGACAGGAGTG | 87682920 | 87682942 | 0.25 |
| GATTTCACAGTGAATCTGAC | 87682927 | 87682949 | 0.07 |
| GTGAAATCTGAACTCTGCGT | 87682942 | 87682964 | 0.63 |
| GATACTATTGTACGTACAATA | 87682967 | 87682989 | 0.07 |
| GCAATAAGGTTTGTGAAAAAC | 87682982 | 87683004 | 0.22 |
| GAACAGGAAAGTGACTATGCA | 87682999 | 87683021 | 0.08 |
| GGCAGTTAATCCCAGCACTC | 87683020 | 87683042 | 0.06 |
| GTATTTAAATATAAGGCTATG | 87683195 | 87683217 | 0.18 |
| GAATAACAGTATTTAAATATA | 87683203 | 87683225 | 0.05 |

| | | | |
|---|---|---|---|
| GTTTAATTTATGTATGTGTGT | 87683225 | 87683247 | 0.02 |
| GGGTAGTATGCTGCCCTTAG | 87683247 | 87683269 | 0.79 |
| GCCCTTAGTGGATAGAGACA | 87683259 | 87683281 | 0.16 |
| GGATGAAAGCCACCTTACAT | 87683309 | 87683331 | 9.52 |
| GTTCTCAGCTCCTATGTAAGG | 87683318 | 87683340 | 1.32 |
| GTAGGAGCTGAGAAGTGAACT | 87683328 | 87683350 | 0.10 |
| GAAGTGAACTTGGGTTCTC | 87683338 | 87683359 | 0.05 |
| GCATAAGTATGATTATCGGGG | 87683388 | 87683410 | 0.23 |
| GATTGTATGTAGAGTATATG | 87683413 | 87683435 | 1.89 |
| GCTCTAACAGCTAGGATTAC | 87683471 | 87683493 | 0.15 |
| GTAGCTGTTAGAGCTTGGATC | 87683481 | 87683503 | 0.12 |
| GTTGTAAATGTTGCAAACTTG | 87683519 | 87683541 | 0.01 |
| GTTGCAAACTTGAGGCGAGCC | 87683528 | 87683550 | 9.08 |
| GAAACACAGTCTCAACCACCC | 87683546 | 87683568 | 3.26 |
| GAGACTGTGTTTCTATGAAG | 87683557 | 87683579 | 0.64 |
| GCTACAGAAGTGACAGTAGTC | 87683606 | 87683628 | 10.56 |
| GTGCACAGCAAAGGTGGGAC | 87683649 | 87683671 | 0.42 |
| GAACTGGTGGTGCACAGCAA | 87683658 | 87683680 | 0.50 |
| GCTGTGCACCACCAGTTCC | 87683663 | 87683684 | 0.90 |
| GTTATGAGAGATTCCTGGAAC | 87683674 | 87683696 | 0.02 |
| GAATAATTTATGAGAGATTCC | 87683680 | 87683702 | 0.48 |
| GCTACAGTGAACGATTGTTAT | 87683754 | 87683776 | 0.01 |
| GATAAATGAATCTTTTTTTAA | 87683811 | 87683833 | 0.03 |
| GATGGTACAAATGCCCTCTTC | 87683883 | 87683905 | 5.63 |
| GAATGATTGTGAGCTACCACG | 87683910 | 87683932 | 0.03 |
| GAATGAGAACATAAAACAGTG | 87684037 | 87684059 | 0.43 |
| GAACATAAAACAGTGTGGGC | 87684042 | 87684064 | 0.14 |
| GTAAAACAGTGTGGGCAGGGC | 87684047 | 87684069 | 0.84 |
| GTGGGCAGGGCAGGAGATGG | 87684056 | 87684078 | 0.01 |
| GTCTCAGATATATATTGAGT | 87684081 | 87684103 | 0.69 |
| GATTGAGTTGGAATACATCCT | 87684094 | 87684116 | 1.54 |
| GAGGTAGTTTCATTTAGCCA | 87684111 | 87684133 | 1.09 |
| GTAAATGAAACTACCTCAAAA | 87684118 | 87684140 | 0.39 |
| GGTTTCTATTTGCCTTTTTG | 87684130 | 87684152 | 0.01 |
| GATTTTTATTCACTCTTACT | 87684151 | 87684173 | 1.22 |
| GTAAGAGTGAATAAAAATCCT | 87684156 | 87684178 | 0.03 |
| GAAGAAAGTTTTCTAAGCCCT | 87684173 | 87684195 | 1.14 |
| GTAGAAAACTTTCTTCCTTCC | 87684182 | 87684204 | 0.13 |
| GCTTCTTTACATTTCCAGGA | 87684196 | 87684218 | 0.04 |
| GTAAAGAAGCTGGTAGAGATT | 87684210 | 87684232 | 0.58 |
| GAGTAAGAAAATCTCAAAG | 87684364 | 87684385 | 0.87 |
| GTCTTACTCTTCATCAGCAAC | 87684378 | 87684400 | 2.22 |
| GTCTTCATCAGCAACAGGGCA | 87684384 | 87684406 | 0.42 |
| GTCAGCAACAGGGCATGGTCC | 87684390 | 87684412 | 0.03 |
| GTACTGTCAGAAGTCAGCACC | 87684408 | 87684430 | 0.78 |
| GTTTTTATATAAATGAATAAT | 87684459 | 87684481 | 0.03 |
| GTCTTTCCTATTTTGCTTT | 87684493 | 87684514 | 0.01 |
| GCCTGTCTCCAAAAGCAAAAT | 87684499 | 87684521 | 0.38 |
| GAATGAGCAGTATTTTAGA | 87684590 | 87684611 | 0.01 |
| GAAGACAACCCTTGCCTGTGT | 87684618 | 87684640 | 3.48 |
| GACCCTTGCCTGTGTTGGCGG | 87684624 | 87684646 | 0.61 |
| GTGCCACCACCGCCAACAC | 87684631 | 87684652 | 0.21 |

| | | | |
|---|---|---|---|
| GTTTAATCCCAGCAGTCAGGC | 87684658 | 87684680 | 0.01 |
| GCTGCCTGCCTGCCTGACTGC | 87684665 | 87684687 | 0.00 |
| GCTGGGACATGTAGACAAGGC | 87684706 | 87684728 | 13.68 |
| GTACATGTCCCAGGATAGCCA | 87684717 | 87684739 | 0.84 |
| GAAAATCAACAGAAAACCAAC | 87684765 | 87684787 | 0.02 |
| GAAACCAACAGGCAGTCTCCA | 87684777 | 87684799 | 0.08 |
| GAGCTAGAATCGGTCCCCA | 87684794 | 87684815 | 0.28 |
| GAAGTGTTGGAGAGCTAGAAT | 87684803 | 87684825 | 2.02 |
| GCATCTCAAATAGACAAGTGT | 87684817 | 87684839 | 0.03 |
| GATGTCAACCATCTTCCCTC | 87684836 | 87684858 | 0.22 |
| GGGAGACCCAGAGGGAAGA | 87684844 | 87684865 | 1.91 |
| GTTACAAGTGGGAGACCCAGA | 87684851 | 87684873 | 1.04 |
| GTCTCCCACTTGTAAGCAAC | 87684859 | 87684881 | 1.76 |
| GTTTTTCCATGTTTTTGAGA | 87684882 | 87684904 | 0.01 |
| GCATTCCCCGTCTCAAAAACA | 87684888 | 87684910 | 0.12 |
| GGAATGACCGTATTCTTGTC | 87684905 | 87684927 | 0.46 |
| GACCGTATTCTTGTCTGGCC | 87684910 | 87684932 | 0.01 |
| GTAGACTACAGTCTGAGTTCC | 87684928 | 87684950 | 5.52 |
| GACTCAGACTGTAGTCTAGAT | 87684934 | 87684956 | 8.49 |
| GCAACTGAGAGGCAAAGGTA | 87684975 | 87684997 | 2.61 |
| GCTTTATTTCCAGCAACTGAG | 87684986 | 87685008 | 0.18 |
| GCTTGCGCCACCACTCGTGG | 87685009 | 87685031 | 1.75 |
| GTGAAGCCCACCACGAGTGG | 87685016 | 87685038 | 0.12 |
| GATTATCATGTGAGCATGTGA | 87685065 | 87685087 | 1.56 |
| GTGAGCATGTGATGGTCATG | 87685073 | 87685095 | 2.15 |
| GTCCATCTTGTTCGTGAGCCC | 87685100 | 87685122 | 0.36 |
| GTGAGCCCAGGACACTTGTG | 87685112 | 87685134 | 0.55 |
| GAGCTCCCCACAAGTGTCCT | 87685117 | 87685139 | 0.74 |
| GTCTTTTTGCCTGCTGTGTCT | 87685138 | 87685160 | 0.35 |
| GTCTGAGCCCTAGACACAGC | 87685146 | 87685168 | 2.06 |
| GACTCAGATCACAAGCCTTG | 87685166 | 87685188 | 1.21 |
| GATCACAAGCCTTGGGGGAC | 87685172 | 87685194 | 0.65 |
| GCTAAAGCACCTGTCCCCCA | 87685181 | 87685203 | 0.01 |
| GAGTTGTCACCAGCATGAG | 87685208 | 87685229 | 0.06 |
| GCAAAGGGCAGCCTCTCATGC | 87685217 | 87685239 | 6.25 |
| GACACAAGTGTACAATGCAAA | 87685233 | 87685255 | 0.53 |
| GTAGACTTGCTATGCATCCC | 87685254 | 87685276 | 0.03 |
| GTCAAGGGTGCGGCCAGCCT | 87685271 | 87685293 | 0.02 |
| GACACAGGCAAGTCAAGGGTG | 87685281 | 87685303 | 0.32 |
| GGCCAACACAGGCAAGTCAA | 87685286 | 87685308 | 2.08 |
| GCATGAGTGAAAGGCCAACAC | 87685297 | 87685319 | 2.31 |
| GAATTCTAGTCCATGAGTGAA | 87685307 | 87685329 | 4.26 |
| GCACTCATGGACTAGAATTCC | 87685312 | 87685334 | 2.03 |
| GATGGACTAGAATTCCAGGCA | 87685317 | 87685339 | 0.41 |
| GGGCATGGAGGCCCATGCC | 87685330 | 87685351 | 0.01 |
| GACAAGAAGATCAGCTGGGCA | 87685344 | 87685366 | 0.14 |
| GAAAGTACAAGAAGATCAGCT | 87685349 | 87685371 | 7.50 |
| GTACTTTTGTTTGCATTTC | 87685365 | 87685386 | 2.84 |
| GTTATTTAAATTTTTATTTTT | 87685414 | 87685436 | 0.08 |
| GTTATCAGACGATTCAAATTT | 87685481 | 87685503 | 1.39 |
| GAAATTTTGGGCAGTTTGAAC | 87685495 | 87685517 | 1.00 |
| GTACTGGCTGAAGTCAAAAG | 87685518 | 87685540 | 9.75 |

| | | | |
|---|---|---|---|
| GCTTCAGCCAGTACATGAAGT | 87685528 | 87685550 | 12.16 |
| GCCATGTCCAACTTCATGTAC | 87685534 | 87685556 | 5.86 |
| GTAGAGCCCTCAACCTTTTCC | 87685564 | 87685586 | 4.05 |
| GTTTTACCTGGAAAAGGTTGA | 87685569 | 87685591 | 1.10 |
| GTTGTCTCTTTTACCTGGAAA | 87685576 | 87685598 | 0.49 |
| GTCCAGGTAAAAGAGACAACA | 87685581 | 87685603 | 2.76 |
| GAACAAAGCATTACTCGCAAA | 87685638 | 87685660 | 0.13 |
| GCGAGTAATGCTTTGTTTAA | 87685644 | 87685666 | 4.47 |
| GTTTGTAGTTTATAACATTAT | 87685668 | 87685690 | 0.04 |
| GACGTCGACATATATTACTTG | 87685735 | 87685757 | 0.77 |
| GAATATATGTCGACGTGTCCT | 87685743 | 87685765 | 0.59 |
| GACAAAATTATTCAAAAACCT | 87685760 | 87685782 | 0.01 |
| GTTTAAAGTTCTATTTTAGTT | 87685799 | 87685821 | 0.02 |
| GGTTAAACACTGCAGTAACT | 87685820 | 87685842 | 0.02 |
| GCAGTAACTGGGAGCAGAGAC | 87685832 | 87685854 | 0.12 |
| GCAGAGACTGGGAGGACAGA | 87685844 | 87685866 | 0.59 |
| GACTGGGAGGACAGAGGGGA | 87685849 | 87685871 | 0.17 |
| GGACAGAGGGGAGGGAACTT | 87685857 | 87685879 | 0.01 |
| GAGGGGAGGGAACTTTGGTT | 87685862 | 87685884 | 0.02 |
| GTTTGGTTAGGATGAGAAAAA | 87685875 | 87685897 | 0.01 |
| GAGAAAAATGGTGTCATTGA | 87685887 | 87685909 | 2.18 |
| GCTGTTAATCTATACTTACAG | 87685919 | 87685941 | 1.44 |
| GTAGATTAACAGGTTGAAATT | 87685931 | 87685953 | 2.21 |
| GCTTAGAAACATTTGTGTGG | 87685959 | 87685981 | 0.93 |
| GAAACATTTGTGTGGGGGC | 87685964 | 87685985 | 2.73 |
| GTTGTGTGGGGGCTGGAGAGA | 87685971 | 87685993 | 0.04 |
| GCTCAGCGGGTAAGAGCACC | 87685993 | 87686015 | 0.14 |
| GTCTTCAGAAACTCCACAAG | 87686080 | 87686102 | 0.00 |
| GAACAGTTCTCAGTTTATTTT | 87686173 | 87686195 | 0.01 |
| GTAGGACATGAAATACGCTTT | 87686218 | 87686240 | 9.47 |
| GACACATCAGCAAAGACTAAT | 87686237 | 87686259 | 3.15 |
| GTATTTATTTCTGTGTTCTA | 87686274 | 87686296 | 7.30 |
| GGTTCTGTTGAAGACACCAC | 87686295 | 87686317 | 8.94 |
| GGCCAGAGACTGAGAGCCAG | 87686311 | 87686333 | 16.23 |
| GTTTGCATTTATTCAATAATG | 87686332 | 87686354 | 1.61 |
| GAATAAATGCAAAACTGCTCA | 87686343 | 87686365 | 1.36 |
| GAACTGCTCAAGGACAAAGAT | 87686354 | 87686376 | 5.16 |
| GACAAAGATTGGTTAACCAG | 87686365 | 87686387 | 8.15 |
| GTGGATCAAGCAGCCGCTCA | 87686384 | 87686406 | 7.99 |
| GTCAAGCAGCCGCTCATGGAT | 87686389 | 87686411 | 1.93 |
| GATCCTGTTCCTATCCATGAG | 87686397 | 87686419 | 9.20 |
| GCATGGATAGGAACAGGATAG | 87686402 | 87686424 | 4.84 |
| GATAGGAACAGGATAGAGGAG | 87686407 | 87686429 | 4.53 |
| GAACAGGATAGAGGAGAGGTA | 87686412 | 87686434 | 23.64 |
| GATAGAGGAGAGGTACGGCC | 87686417 | 87686439 | 1.47 |
| GCAGTGAAAGCACACACTCC | 87686435 | 87686457 | 0.01 |
| GTTAAGCACACGCTGCTCAG | 87686457 | 87686479 | 3.51 |
| GCAGCGTGTGCTTAACTGTG | 87686464 | 87686486 | 8.58 |
| GAGTATGTATCAGAGTAAG | 87686492 | 87686513 | 3.58 |
| GTTTTTCTTTTGTTTTGTTT | 87686532 | 87686554 | 0.01 |
| GTTTGTTTTGTTTTGGAGTCC | 87686540 | 87686562 | 0.01 |
| GTATTGAGTTCAGAGTCAACC | 87686558 | 87686580 | 6.26 |

| | | | |
|---|---|---|---|
| GCTGAACTCAATATTTAGTTG | 87686569 | 87686591 | 0.17 |
| GAAAAGAAATTACTATGTGCA | 87686597 | 87686619 | 0.03 |
| GCTGTCTCTACACACACACAG | 87686639 | 87686661 | 0.08 |
| GTAGAGACAGACATACAGAC | 87686652 | 87686674 | 11.72 |
| GATACAGACAGGCAGGCAGGC | 87686664 | 87686686 | 0.04 |
| GCAGACGCATGAGCACGTAC | 87686690 | 87686712 | 3.50 |
| GCATGAGCACGTACAGGTGT | 87686696 | 87686718 | 1.06 |
| GGTGTTGGCTGTACATTCG | 87686711 | 87686732 | 1.85 |
| GCCTACATGAAGCTGGAAGA | 87686737 | 87686759 | 0.00 |
| GTTCCAGCTTCATGTAGGCTC | 87686742 | 87686764 | 0.63 |
| GTATTTGCTGAGTTTGACCCC | 87686761 | 87686783 | 9.01 |
| GTCAAACTCAGCAAATAGAC | 87686767 | 87686789 | 3.00 |
| GTAGGCGAGAGAAAGGCTCAG | 87686811 | 87686833 | 4.04 |
| GTTCAAAGTTAGGCGAGAGAA | 87686819 | 87686841 | 0.95 |
| GCCCGGGAGGTAGAGGCAAA | 87686850 | 87686872 | 0.06 |
| GCCTCTACCTCCCGGGCCCT | 87686856 | 87686878 | 2.78 |
| GCGGATGGTGGCCCAGGGCC | 87686867 | 87686889 | 0.71 |
| GAAACTGCGGATGGTGGCCCA | 87686872 | 87686894 | 0.21 |
| GCCACCATCCGCAGTTTATG | 87686878 | 87686900 | 1.35 |
| GCAGTTTATGTGGTACTGGG | 87686888 | 87686910 | 1.47 |
| GCTGGGAGGTTGAAGCAGTCC | 87686903 | 87686925 | 3.17 |
| GCATCCTGAACGAGAGTTTCC | 87686921 | 87686943 | 0.01 |
| GAAAGAAAAGAAACTGGGCTA | 87686960 | 87686982 | 0.02 |
| GTTATTTTATGTATGAGTATA | 87687007 | 87687029 | 0.03 |
| GACAGCATCAGGCTAGAGAGA | 87687157 | 87687179 | 1.20 |
| GATGCTGTACTTTTAATACGC | 87687173 | 87687195 | 0.56 |
| GCTAGGTCTTGTGTAAGCTAA | 87687207 | 87687229 | 1.19 |
| GAAGGTATGCGAGTTGGGACT | 87687225 | 87687247 | 0.10 |
| GACATAGAAGGTATGCGAGTT | 87687231 | 87687253 | 3.81 |
| GACAGGGGGTGAGAACATAGA | 87687244 | 87687266 | 0.23 |
| GACCCCCTGTCTTCCTTCCCC | 87687258 | 87687280 | 0.63 |
| GCTTCCTTCCCCTGGATGATC | 87687267 | 87687289 | 0.83 |
| GTCAGTGACCGGATCATCCA | 87687275 | 87687297 | 0.41 |
| GGCATTCATTCGTCAGTGAC | 87687286 | 87687308 | 0.01 |
| GCAGACGTCATCTGGATCACC | 87687308 | 87687330 | 1.93 |
| GCATACACTCAGACGTCATC | 87687317 | 87687339 | 0.38 |
| GATGCATGCACATCACGTGTG | 87687336 | 87687358 | 4.85 |
| GCACTCTGTGCTGATGCTCTC | 87687362 | 87687384 | 5.24 |
| GTTGCTTATAACTAGTTACA | 87687392 | 87687414 | 0.44 |
| GAAGCAACTGTGACTGCTATC | 87687408 | 87687430 | 0.24 |
| GTGACTGCTATCTGGGTACT | 87687416 | 87687438 | 0.00 |
| GGACTCAAAACTGCTTTCTT | 87687437 | 87687459 | 5.06 |
| GGAGAGATGACCAAGAGCAC | 87687468 | 87687490 | 1.33 |
| GTCTCCAGCTCCCACGAACGT | 87687486 | 87687508 | 1.84 |
| GTTTTTAAAACCAACGTTCG | 87687496 | 87687518 | 0.07 |
| GTTAAAAACCCAGTGTTGGCT | 87687511 | 87687533 | 0.18 |
| GTCTTTCACCAAGCCAACACT | 87687518 | 87687540 | 3.85 |
| GGCTTGGTGAAAGATGCAG | 87687527 | 87687548 | 1.13 |
| GGTTAAAGACAGTTGCTTCC | 87687547 | 87687569 | 0.01 |
| GATGCACTAGGTCATCGGGCC | 87687565 | 87687587 | 0.00 |
| GCTCAGATGCACTAGGTCATC | 87687570 | 87687592 | 3.67 |
| GTGGGTCCCTCAGATGCACT | 87687578 | 87687600 | 0.71 |

| | | | |
|---|---|---|---|
| GCTGAGGGACCCACGCTGTAG | 87687588 | 87687610 | 2.02 |
| GATCTTCTCCCTCTACAGCGT | 87687596 | 87687618 | 0.13 |
| GATGTGTCACAGCACACACA | 87687635 | 87687657 | 0.58 |
| GATCTGTACACCCATGTACTC | 87687655 | 87687677 | 0.46 |
| GCAGTTACTTCCCGGAGTACA | 87687665 | 87687687 | 1.99 |
| GCAATGATCACAGTTACTTCC | 87687674 | 87687696 | 0.98 |
| GTTTGAAATACCAGATATCTT | 87687698 | 87687720 | 0.34 |
| GAAATCAATCCTAAGATATC | 87687707 | 87687729 | 0.01 |
| GTTGATTTCCAGAGAGCCCTT | 87687722 | 87687744 | 19.26 |
| GAGAGCCCTTTGGAATTTTA | 87687732 | 87687754 | 0.62 |
| GTTAGTCCCTAAAATTCCAA | 87687738 | 87687760 | 4.24 |
| GAATTAATTCACAATTTAAC | 87687787 | 87687809 | 0.32 |
| GTCAGCTGCACAAAATTTGAT | 87687809 | 87687831 | 0.00 |
| GTTGAATTTAACATGAGTGAT | 87687836 | 87687858 | 0.01 |
| GAAATATCAAAGCTTCGAG | 87687874 | 87687895 | 0.29 |
| GCTAAAGTTGTGAATTTAAAG | 87687895 | 87687917 | 0.01 |
| GAATTTAAAGTGGCATTGTCA | 87687906 | 87687928 | 0.83 |
| GATAACAGACAATGGAAAAA | 87687944 | 87687966 | 0.06 |
| GTTCCATTGTCTGTTATCTAG | 87687950 | 87687972 | 0.02 |
| GCACTGTAATTACTTCTTAGG | 87687983 | 87688005 | 0.83 |
| GTGAAACCAGCAATTTGTT | 87688021 | 87688042 | 1.72 |
| GAGAAGCCTAACAAATTGC | 87688027 | 87688048 | 2.14 |
| GTTAGGCTTCTCAGGATCTC | 87688037 | 87688059 | 0.95 |
| GGATCTCTGGGCCAGACTGA | 87688050 | 87688072 | 2.95 |
| GATCACAATGCTCCTTCAGTC | 87688061 | 87688083 | 2.82 |
| GTTTTTTTTTTGCTTCAAGTC | 87688108 | 87688130 | 0.02 |
| GTCCGGGTCTTGCATAGCTC | 87688125 | 87688147 | 1.38 |
| GAACACGAAATCTGAGTTTG | 87688152 | 87688174 | 0.60 |
| GTCTTCCTGTCTTCATCTCCC | 87688173 | 87688195 | 0.01 |
| GATGCTTGTATGCTAGCACCT | 87688190 | 87688212 | 0.01 |
| GAAAACTCAGGCACAACAGAC | 87688224 | 87688246 | 0.15 |
| GCAATCTTCCACAAAACTC | 87688237 | 87688258 | 0.01 |
| GTCAGGAGAGAGGGAGGGGAG | 87688343 | 87688365 | 0.01 |
| GCTGTCTCAGGAGAGAGGGAG | 87688348 | 87688370 | 0.01 |
| GAGAAACTGTCTCAGGAGAG | 87688354 | 87688376 | 5.61 |
| GAAAGGAGCAGAGTTACTTCC | 87688394 | 87688416 | 0.25 |
| GATCGGAAGAGGAGGAGGAGG | 87688445 | 87688467 | 0.03 |
| GAAATAAATCGGAAGAGGAGG | 87688451 | 87688473 | 0.75 |
| GATAAATAAATAAATCGGAAG | 87688457 | 87688479 | 0.02 |
| GCATAAAATAAATAAATAAAT | 87688463 | 87688485 | 0.02 |
| GACACAACCAGGAGAGAGCAT | 87688514 | 87688536 | 0.05 |
| GGCTTACAATGATCTATGAT | 87688539 | 87688561 | 2.26 |
| GATCATTGTAAGCCGCCATG | 87688548 | 87688570 | 0.02 |
| GAAGCGCAGGACTGGAGAGA | 87688629 | 87688651 | 0.03 |
| GTAAAAGGGGAAGCGCAGGAC | 87688637 | 87688659 | 0.87 |
| GAACTTTAAAAGGGGAAGCGC | 87688642 | 87688664 | 11.26 |
| GAAAAAAAATAACTTTAAAAG | 87688651 | 87688673 | 0.04 |
| GTTTTTTTCTTTAGCTAACCA | 87688669 | 87688691 | 0.02 |
| GTTACATGTGAGTATCACCA | 87688686 | 87688708 | 2.66 |
| GTTCCAGCCTTAAAGAGATAG | 87688713 | 87688735 | 1.72 |
| GTCACCGCCTCTATCTCTTTA | 87688719 | 87688741 | 1.42 |
| GATAGTGACTTGTGTGTGTGG | 87688744 | 87688766 | 1.10 |

| | | | |
|---|---|---|---|
| GTGGCTTGTTGCCGCACATG | 87688763 | 87688785 | 0.99 |
| GCCGCACATGTGGAAGTCAG | 87688773 | 87688795 | 0.04 |
| GTGGAAGTCAGAGGACAGCG | 87688782 | 87688804 | 0.02 |
| GTCAGTTCCCCCCAGCCGTG | 87688808 | 87688830 | 4.66 |
| GCAGGAGGTCCACACGGCTGG | 87688816 | 87688838 | 7.94 |
| GCAATCCCCAGGAGGTCCACA | 87688823 | 87688845 | 2.97 |
| GAACTGAGTTCAATCCCCAGG | 87688832 | 87688854 | 0.02 |
| GAACTCAGTTGTCAAGCTCGG | 87688846 | 87688868 | 5.87 |
| GATAAGATGGCTCAACAGGA | 87688875 | 87688897 | 0.25 |
| GTACACGAGTAAGTGATAAGA | 87688888 | 87688910 | 3.37 |
| GTACTTATTTATTTTTTTCC | 87688919 | 87688941 | 0.03 |
| GTTTTTCCAGGTTAAATTTAG | 87688932 | 87688954 | 0.02 |
| GTTTAGTGGAAGCTTTTGTCG | 87688947 | 87688969 | 1.77 |
| GTTGTCGAGGATTCAGAACTG | 87688961 | 87688983 | 7.44 |
| GACTGAGGCAGAGTTTACAGG | 87688977 | 87688999 | 13.78 |
| GTACAGGAGGATTTGCTTCGC | 87688991 | 87689013 | 3.62 |
| GCCGGTTTCCAGACCTTAAC | 87689009 | 87689031 | 5.65 |
| GTTGGCAAGCCGGTTAAGGTC | 87689017 | 87689039 | 11.32 |
| GATTTCTTGGCAAGCCGGTTA | 87689022 | 87689044 | 5.27 |
| GTCTGGAATTTCTTGGCAAGC | 87689028 | 87689050 | 5.24 |
| GCTTGTCTCTGGAATTTCT | 87689036 | 87689057 | 6.69 |
| GTAAATTCGCTGCTTGTCTC | 87689046 | 87689068 | 5.15 |
| GACTGTTACCGACTGTATCA | 87689071 | 87689093 | 15.13 |
| GGTTAATACCCTGATACAGT | 87689079 | 87689101 | 6.39 |
| GCCTGGATGACGCTGGGGAGC | 87689100 | 87689122 | 5.34 |
| GCCAGAGCCTGGATGACGCTG | 87689106 | 87689128 | 4.75 |
| GCTTCGTATTTCTCCAGAGCC | 87689118 | 87689140 | 2.61 |
| GAAATACGAAGGTATCGTG | 87689130 | 87689151 | 4.13 |
| GCTAACGTAAAACAAAGCTGC | 87689160 | 87689182 | 57.45 |
| GAAAACGGGGTGAGGGAAAAA | 87689188 | 87689210 | 3.56 |
| GCAGGGAAGTAAAACGGGGTG | 87689197 | 87689219 | 0.75 |
| GAACACAGGGAAGTAAAACG | 87689202 | 87689224 | 12.19 |
| GCAGCACATTTGTTAGAACAC | 87689216 | 87689238 | 1.23 |
| GTTCTAACAAATGTGCTGTT | 87689221 | 87689243 | 0.79 |
| GCAAATGTGCTGTTAGGTCTG | 87689228 | 87689250 | 1.86 |
| GGGATGTTACACTTTCCAAT | 87689250 | 87689272 | 3.53 |
| GCTGACTCAAAACAAACCTAT | 87689265 | 87689287 | 3.78 |
| GTTTTGAGTCAGGCCCAGAC | 87689276 | 87689298 | 1.19 |
| GCCCAGACAGGGGTCTAGTA | 87689288 | 87689310 | 0.14 |
| GACAGGGGTCTAGTAAGGAA | 87689293 | 87689315 | 1.92 |
| GTCTAGTAAGGAAAGGGACCT | 87689301 | 87689323 | 0.14 |
| GGACCTTGGTGGCTAGCTGT | 87689315 | 87689337 | 2.22 |
| GTAATGTAATGATTTAGCAA | 87689341 | 87689363 | 1.21 |
| GTAATGATTTAGCAAGGGGG | 87689346 | 87689368 | 3.07 |
| GATTTAGCAAGGGGGAGGGCA | 87689352 | 87689374 | 0.01 |
| GACGGCAAACGTGGTGCTGGG | 87689375 | 87689397 | 1.39 |
| GCACGGGAAACACGGCAAACG | 87689385 | 87689407 | 6.39 |
| GTTTGCCGTGTTTCCCGTGTT | 87689390 | 87689412 | 1.05 |
| GACCTAGTAGGCCCAAACAC | 87689402 | 87689424 | 1.14 |
| GGGCTGACCTTGACCTAGT | 87689414 | 87689435 | 3.40 |
| GACATGATGTTGTGCAGTCT | 87689433 | 87689455 | 5.96 |
| GAACATCATGTCGCAATAAAA | 87689445 | 87689467 | 2.34 |

| | | | |
|---|---|---|---|
| GTATTCTATTAGATAACTATA | 87689472 | 87689494 | 1.13 |
| GAGATCATGTCCTTGACAA | 87689504 | 87689525 | 3.21 |
| GCCACACACACACCTTTGTCA | 87689514 | 87689536 | 0.01 |
| GGTGTGTGTGTGGGCACTG | 87689524 | 87689545 | 3.59 |
| GCGTGCTTTGCATATATAGA | 87689552 | 87689574 | 0.05 |
| GCTCTGGGCACGCGTACACA | 87689586 | 87689608 | 0.01 |
| GAAACTCTCAAGTGTAGCTC | 87689602 | 87689624 | 0.49 |
| GTAGGAAAGCAAGCATCTGAA | 87689629 | 87689651 | 0.24 |
| GCAGATGCTTGCTTTCCTAGT | 87689634 | 87689656 | 1.07 |
| GTTGCTTTCCTAGTTGGGCGA | 87689641 | 87689663 | 0.18 |
| GCAACTGGCCATCGCCCAACT | 87689648 | 87689670 | 2.94 |
| GGCCAGTTGATGTGTGCTCT | 87689662 | 87689684 | 0.01 |
| GTCACACTGAGGGGGGGGGAG | 87689691 | 87689713 | 0.02 |
| GATAACCTCACACTGAGGGG | 87689698 | 87689720 | 0.75 |
| GCTCCCGATAACCTCACACTG | 87689703 | 87689725 | 4.25 |
| GCAGCCATGTCTTACTCCATC | 87689729 | 87689751 | 3.14 |
| GCCAGATCTGCAGCACCCAGA | 87689744 | 87689766 | 0.54 |
| GAAGCACTAGTTCAGGACAAA | 87689776 | 87689798 | 0.18 |
| GATGGCCAAAAGCACTAGTTC | 87689784 | 87689806 | 1.31 |
| GGGAAGATGGACGACTCAA | 87689803 | 87689824 | 1.03 |
| GTAAGGGGGAGACTGGGAAGA | 87689815 | 87689837 | 0.09 |
| GAAGGAAGCTAAGGGGGAGAC | 87689823 | 87689845 | 0.06 |
| GTCAATCCAAGGAAGCTAAGG | 87689830 | 87689852 | 1.41 |
| GAAACTTGCATTCAATCCA | 87689842 | 87689863 | 0.12 |
| GACATGTAAAGCATTTTGAGT | 87689904 | 87689926 | 0.03 |
| GCTATTAAAACATACCCCGT | 87689951 | 87689973 | 0.02 |
| GAAACATACCCCGTAGGGGCT | 87689958 | 87689980 | 0.15 |
| GTCTACTGCCAAGCCCCTACG | 87689965 | 87689987 | 1.02 |
| GTTGGCAGTAGATTGCTTATA | 87689977 | 87689999 | 0.33 |
| GCAGAGCTATTTTGAGATACT | 87690016 | 87690038 | 0.01 |
| GAAATAGCTCTGTCAGTGTTA | 87690028 | 87690050 | 2.18 |
| GAAGCTGAATTTAAATTTGTC | 87690069 | 87690091 | 0.01 |
| GAATTTATTTTGTGTAATGTG | 87690131 | 87690153 | 0.01 |
| GTGTAATGTGCGGAAGCAGG | 87690141 | 87690163 | 0.97 |
| GGAAGCAGGAGGGCAGCTTT | 87690152 | 87690174 | 2.45 |
| GGAGGGCAGCTTTGGGGAGT | 87690159 | 87690181 | 0.13 |
| GTCTGTGACCCACAGGATAGA | 87690188 | 87690210 | 0.07 |
| GTTCGGAGTTCTGTGACCCAC | 87690196 | 87690218 | 3.56 |
| GGTCACAGAACTCCGAAATC | 87690202 | 87690224 | 3.00 |
| GCAAGCATGGCTACCTGATTT | 87690214 | 87690236 | 0.01 |
| GAAGGGGCTTGCTGTCAAGCA | 87690228 | 87690250 | 11.92 |
| GAACATGGCTCAGTGGGTAAG | 87690245 | 87690267 | 0.02 |
| GGCCCACAACATGGCTCAGT | 87690252 | 87690274 | 0.81 |
| GGTGCCTCGGGCCCACAACA | 87690261 | 87690283 | 2.07 |
| GTTGTGGGCCCGAGGCACCA | 87690265 | 87690287 | 1.04 |
| GCTCAGTTACCTTGGTGCCTC | 87690273 | 87690295 | 2.00 |
| GACCAAGGTAACTGAGGGCTA | 87690281 | 87690303 | 5.58 |
| GGCTAAGGAGTTTAAGCACT | 87690296 | 87690318 | 0.53 |
| GAAGCACTTGGTACACAGGCC | 87690309 | 87690331 | 0.04 |
| GACACAGGCCTGGACTGAAGT | 87690320 | 87690342 | 0.17 |
| GAGCACCCCAACTTCAGTCC | 87690327 | 87690349 | 0.42 |
| GTTGGGGTGCTCATAGTTCC | 87690338 | 87690360 | 0.02 |

| | | | |
|---|---|---|---|
| GCTCATAGTTCCAGGTGGAGA | 87690347 | 87690369 | 8.01 |
| GTCAAAGATCCCGTCTCCACC | 87690356 | 87690378 | 0.24 |
| GGATCTTTGAAGCTCCAACC | 87690369 | 87690391 | 2.25 |
| GATGAGTCTTGCTAGCCAGGT | 87690383 | 87690405 | 0.11 |
| GTGAGCTCTAGTTTCAACT | 87690413 | 87690434 | 0.02 |
| GTTTCAACTAGGAGACCTTG | 87690423 | 87690445 | 1.05 |
| GACTAGGAGACCTTGTGGGTT | 87690429 | 87690451 | 0.00 |
| GCTCTGGATCCCGAACCCACA | 87690438 | 87690460 | 3.03 |
| GAAAACTGCGGAGCTTTTCTC | 87690455 | 87690477 | 4.62 |
| GGAAGTGCTCTAAAAACTG | 87690468 | 87690489 | 0.03 |
| GTTTTTAGAGCACTTCCAG | 87690473 | 87690494 | 0.01 |
| GTCCTGAGTTCAACTCCCTC | 87690494 | 87690516 | 0.42 |
| GCTGTCTTCAGATCCACCAG | 87690510 | 87690532 | 0.08 |
| GAATACATCTTTAAAACAGAG | 87690559 | 87690581 | 0.18 |
| GACCTTCCCTCGAGACTGCCG | 87690589 | 87690611 | 0.01 |
| GCTTCCCCACGGCAGTCTCGA | 87690594 | 87690616 | 4.13 |
| GTTCCAGACTGCTCTTCCCCA | 87690606 | 87690628 | 0.32 |
| GTCCTGACAGCAGCCTTCACT | 87690632 | 87690654 | 0.57 |
| GCGGATGCACATCCAAGTGA | 87690644 | 87690666 | 0.94 |
| GTCCGCTCGCATCTGAGCGTG | 87690662 | 87690684 | 0.20 |
| GCACACGCTCATACAAACACG | 87690686 | 87690708 | 0.01 |
| GGAAAGATGATACTTTACAA | 87690708 | 87690730 | 3.57 |
| GAAGGGTTCTTATTTTCTCAA | 87690727 | 87690749 | 0.02 |
| GAGGAGGAGTGTTTCTACAA | 87690751 | 87690773 | 15.38 |
| GAAACACTCCTCCTCTCGGT | 87690759 | 87690781 | 1.29 |
| GGCACATCTAACCAACCGAG | 87690770 | 87690792 | 0.12 |
| GGTTGGTTAGATGTGCCAAC | 87690776 | 87690798 | 0.02 |
| GATGAGCTTGTACCACCGGT | 87690791 | 87690813 | 0.56 |
| GTACAAGCTCATCACTGAGCC | 87690802 | 87690824 | 0.01 |
| GTCTAAGATCACTGGGGTCCC | 87690820 | 87690842 | 0.01 |
| GCATGGGTTCTAAGATCACT | 87690828 | 87690850 | 1.19 |
| GCTTAGAACCCATGCAGTGGA | 87690837 | 87690859 | 0.26 |
| GAGTCAGCCCTTCCACTGCA | 87690845 | 87690867 | 0.38 |
| GTGGAAGGGCTGACTCCTGC | 87690852 | 87690874 | 0.47 |
| GCTGACTCCTGCAGGGCTCC | 87690860 | 87690882 | 0.08 |
| GTGCAGACCAGGAGCCCTGC | 87690867 | 87690889 | 0.71 |
| GCCATGCTGTGTGTGCAGACC | 87690878 | 87690900 | 0.10 |
| GTCACAGAGTGGAGGAATC | 87690929 | 87690950 | 0.02 |
| GAATTCGTAGTCACAGAGTGG | 87690936 | 87690958 | 0.01 |
| GTTTATGTGATGGGGAAACTG | 87690972 | 87690994 | 0.58 |
| GATGCTACACATTTATGTGA | 87690983 | 87691005 | 0.01 |
| GCCAGACAAAATGATATAAAA | 87691015 | 87691037 | 0.04 |
| GTCATTTTGTCTGGTTTCAGC | 87691025 | 87691047 | 0.01 |
| GTCTGGTTTCAGCAGGAATT | 87691032 | 87691054 | 0.10 |
| GAATTAGGGATGTGCTCTGAC | 87691048 | 87691070 | 0.23 |
| GCTGGGCCGCTCTAAGCAGTA | 87691067 | 87691089 | 6.94 |
| GCAAACAGCTGCTGTGTGCTG | 87691090 | 87691112 | 0.97 |
| GTCTATCTGCTACTGTACAGT | 87691136 | 87691158 | 0.26 |
| GTGACTTACTAGGAAACAAA | 87691162 | 87691184 | 0.01 |
| GTGTACTAGGTGACTTACT | 87691172 | 87691193 | 0.01 |
| GAGTAATTAATCGTGTACT | 87691184 | 87691205 | 0.00 |
| GTGTCCTATGAAGCCCTCCC | 87691246 | 87691268 | 8.47 |

| | | | |
|---|---|---|---|
| GAAGCCCTCCCTGGGTGGCC | 87691255 | 87691277 | 0.00 |
| GAATTCCAGGCCACCCAGGG | 87691260 | 87691282 | 0.01 |
| GAATTCTGTATAAGCAAGGC | 87691277 | 87691299 | 4.32 |
| GCACGAAGACCTGCCCTGCTG | 87691309 | 87691331 | 0.13 |
| GCAGTGCTTTTACCTCAGCA | 87691321 | 87691343 | 26.47 |
| GATAAATAAATCACACAAGCT | 87691353 | 87691375 | 7.38 |
| GAATGTGCTAAATCACAGAAA | 87691410 | 87691432 | 0.01 |
| GCAGATCTGTGCTGAGGGAAT | 87691450 | 87691472 | 3.90 |
| GCATGACCAGATCTGTGCTGA | 87691456 | 87691478 | 0.53 |
| GACAGATCTGGTCATGAGAGG | 87691464 | 87691486 | 1.31 |
| GAGGCGGCAGAGTTTGAGGC | 87691480 | 87691502 | 1.79 |
| GAGGCCGGCCTGGTTTACAT | 87691495 | 87691517 | 0.01 |
| GTAGAACTTCCTATGTAAACC | 87691503 | 87691525 | 0.01 |
| GTCCTAGTCTGAATCGCTTT | 87691627 | 87691649 | 0.01 |
| GATGAGTGCATCTTCCTCTCT | 87691651 | 87691673 | 5.11 |
| GCATCTTCCTCTCTCGGCTCC | 87691658 | 87691680 | 3.65 |
| GTTAGAACCGGGAGCCGAGAG | 87691664 | 87691686 | 0.42 |
| GAAGTGCAGGCTTTTAGAAC | 87691677 | 87691699 | 0.40 |
| GCAGAGGATGAAGAAGTGC | 87691690 | 87691711 | 0.55 |
| GAAGCAGATGTGTAAGCAG | 87691705 | 87691726 | 0.01 |
| GCTGCTTCTGACCCTGACCGC | 87691720 | 87691742 | 1.00 |
| GTCTGACCCTGACCGCAGGGC | 87691725 | 87691747 | 27.71 |
| GTCAGCCCAGCCCTGCGGTC | 87691731 | 87691753 | 0.01 |
| GGCTGGGCTGACAGGTGCTA | 87691742 | 87691764 | 0.02 |
| GGCTACATCTATCAACAAAC | 87691767 | 87691789 | 0.01 |
| GTGTGGTGTGCGCGCTCGC | 87691788 | 87691809 | 0.94 |
| GTTCTTAAAGTTTATAAGCTC | 87691850 | 87691872 | 0.01 |
| GTTTTATGTTCCGTGTTCC | 87691881 | 87691902 | 0.38 |
| GTGTTCCCGGTTTCCTTCTG | 87691893 | 87691915 | 1.40 |
| GAGGACCCACAGAAGGAAAC | 87691899 | 87691921 | 0.02 |
| GGGATGGGAGGACCCACAGA | 87691906 | 87691928 | 2.20 |
| GTTAAAAATAATGGGGATGGG | 87691918 | 87691940 | 0.01 |
| GCTGAGCTCTTTAAAAATAA | 87691928 | 87691950 | 0.03 |
| GCAGCACAGTTCTTTAGTGTG | 87691947 | 87691969 | 0.01 |
| GCTAATATACTTTTTAAAGCA | 87691976 | 87691998 | 0.01 |
| GTCTGTAACATCTCAAAATAA | 87692002 | 87692024 | 0.17 |
| GTTGTCCTCCTGTGATGTTTT | 87692029 | 87692051 | 0.26 |
| GTCAAAACCGAAAACATCAC | 87692036 | 87692058 | 0.22 |
| GTCCAGAGAACAGCAAGAGTC | 87692081 | 87692103 | 0.01 |
| GGTGTGTTCACAGACAGCAG | 87692102 | 87692124 | 0.29 |
| GGGGCAGCAGCAGCTTGGAA | 87692122 | 87692144 | 1.44 |
| GCAGCAGCAGCTTGGAAAGGG | 87692126 | 87692148 | 0.03 |
| GAAAGGGAGGGCATTTGCAT | 87692139 | 87692161 | 2.31 |
| GTAAGATACTTGCCAGCTTAT | 87692178 | 87692200 | 1.08 |
| GCTAGAACCTCCCAATAAGC | 87692189 | 87692211 | 0.63 |
| GGGAGGTTCTAGCTGGGTAA | 87692199 | 87692221 | 0.01 |
| GTAATGGAGTTAGCTGCACAG | 87692216 | 87692238 | 0.55 |
| GTAGGAAAACTGCTTTTGTTG | 87692240 | 87692262 | 0.01 |
| GCAGTTTTCCTATCTTTGGGA | 87692252 | 87692274 | 0.40 |
| GCAGTCACCTTCCCAAAGAT | 87692259 | 87692281 | 0.16 |
| GCTTTAGTTGACCCCATGGAG | 87692286 | 87692308 | 0.30 |
| GCAGGCATCACCCCGCTCCAT | 87692297 | 87692319 | 1.22 |

| | | | |
|---|---|---|---|
| GAGGGTGATCTGGCTGGAC | 87692316 | 87692337 | 1.15 |
| GTTCTAGATGTGAGGGTGATC | 87692325 | 87692347 | 0.02 |
| GTCACCCTCACATCTAGAAAC | 87692330 | 87692352 | 0.01 |
| GATGTCTATCTCAAATGTTCC | 87692360 | 87692382 | 2.73 |
| GTTATTTTATATTTTACCC | 87692378 | 87692399 | 0.02 |
| GAAAATAACGATGTTCTACAT | 87692392 | 87692414 | 1.36 |
| GTGAGTTATCTTAATCTGC | 87692423 | 87692444 | 0.01 |
| GTTATGTCTAACTTATCTGTT | 87692463 | 87692485 | 0.06 |
| GTCTATCTGTGCCTGAAAC | 87692533 | 87692554 | 0.01 |
| GGGTCTGTAACACCAGTTTC | 87692544 | 87692566 | 0.03 |
| GTTGGTTTAGGGTCAGGGTCA | 87692583 | 87692605 | 0.42 |
| GTAGGGTTTGGTTTAGGGTCA | 87692589 | 87692611 | 0.00 |
| GCAGGGTTAGGGTTTGGTTT | 87692596 | 87692618 | 0.10 |
| GACAAGCAGGGTTAGGGTT | 87692602 | 87692623 | 0.24 |
| GGGGCTGACAAGCAGGGTTA | 87692607 | 87692629 | 0.01 |
| GCTCACAGGGGCTGACAAGCA | 87692613 | 87692635 | 0.02 |
| GTCAGCCCCTGTGAGGGTAC | 87692621 | 87692643 | 0.14 |
| GAATTCCCAGTACCCTCACAG | 87692626 | 87692648 | 0.01 |
| GGAATTGGACCCTTACCCTC | 87692643 | 87692665 | 0.03 |
| GCTGCTCTTCCAGAGGGTAA | 87692652 | 87692674 | 0.03 |
| GTCTCACCAGCCACATGCAAA | 87692697 | 87692719 | 0.15 |
| GAAATTCCATTTGCATGTGGC | 87692702 | 87692724 | 1.86 |
| GAGAGTGCATTGAGTCTTTT | 87692730 | 87692752 | 0.03 |
| GTTGGCAGAATACTGTGAAAA | 87692766 | 87692788 | 0.00 |
| GCACAGTATTCTGCCAATCTA | 87692773 | 87692795 | 0.01 |
| GATTCTGCCAATCTATGGGCG | 87692779 | 87692801 | 0.01 |
| GACACCCCACGCCCATAGAT | 87692785 | 87692807 | 2.74 |
| GTTCAGTTTGTTTATCTCTCC | 87692808 | 87692830 | 0.01 |
| GAAGCATGAAGTGTCAGGCC | 87692826 | 87692848 | 0.02 |
| GTAACTGAAGCATGAAGTGTC | 87692831 | 87692853 | 0.46 |
| GTTAAGAAGTTCACAATTCCT | 87692856 | 87692878 | 0.48 |
| GGATGTCAAAGATCACCCA | 87692873 | 87692894 | 0.10 |
| GCTTGCTCAGTAAGGGTCT | 87692893 | 87692914 | 0.00 |
| GCTCTCATGCTTGCTCAGTAA | 87692899 | 87692921 | 0.01 |
| GAAGCATGAGAGATGCTGAGA | 87692911 | 87692933 | 0.01 |
| GATGCTGAGAAGGCTCAGAGA | 87692922 | 87692944 | 0.52 |
| GCTCAACACTTAAAAGAGCAC | 87692945 | 87692967 | 0.24 |
| GCACTGGGTGCTCTTCCAC | 87692961 | 87692982 | 0.03 |
| GGGTGCTCTTCCACAGGACC | 87692966 | 87692988 | 0.01 |
| GCTATGAACCATGCTGGTGC | 87692998 | 87693020 | 0.01 |
| GCAGATGGCTATGAACCATGC | 87693004 | 87693026 | 1.01 |
| GTTCATAGCCATCTGTAAATC | 87693013 | 87693035 | 0.33 |
| GCCATCTGTAAATCAGGCCC | 87693019 | 87693041 | 1.23 |
| GACATGTTTGGTACCCAAGG | 87693063 | 87693085 | 0.04 |
| GTCTGGCATCACTGACATGTT | 87693075 | 87693097 | 1.44 |
| GTGATGCCAGACAGACAGAC | 87693087 | 87693109 | 0.27 |
| GCTTTGCCTGTCTGTCTGTC | 87693093 | 87693115 | 0.10 |
| GCAAAGCACCTGTGCACTTA | 87693109 | 87693131 | 0.50 |
| GTTTTAAAGCCTTAAGTGCAC | 87693117 | 87693139 | 0.01 |
| GATTACTGATGCTGTATATAG | 87693155 | 87693177 | 0.08 |
| GATACTAAGTTTGGGAATTAT | 87693184 | 87693206 | 0.01 |
| GTTCCCAAACTTAGTATGCTG | 87693191 | 87693213 | 1.38 |

| | | | |
|---|---|---|---|
| GACTTAGTATGCTGAGGCAGG | 87693198 | 87693220 | 0.05 |
| GTTTATGTAGCTCAGAATTTA | 87693225 | 87693247 | 0.02 |
| GATTCTGAGCTACATAAATTT | 87693231 | 87693253 | 0.02 |
| GTTGTTCTGGCTTTTGAAACA | 87693268 | 87693290 | 0.04 |
| GTCTTTTCTTCTTGTTTGTTC | 87693282 | 87693304 | 0.00 |
| GCAGAGAAACAGCCAGCTTCT | 87693319 | 87693341 | 0.01 |
| GCCAGCTTCTAGGATTTGAG | 87693329 | 87693351 | 0.01 |
| GCATCACTGGCTGATACAGTG | 87693360 | 87693382 | 0.46 |
| GATCAGCCAGTGATGCGTTTA | 87693369 | 87693391 | 0.11 |
| GCTAGCCTTAAACGCATCAC | 87693374 | 87693396 | 0.41 |
| GCATGTTGAAGTCATGAAGTG | 87693407 | 87693429 | 0.00 |
| GTCACAGTGCTAGAAAATAA | 87693441 | 87693463 | 0.03 |
| GCTAGAAAATAAGGGGAGAG | 87693449 | 87693471 | 0.64 |
| GGGGAGAGTGGTAGAGCTG | 87693461 | 87693482 | 0.16 |
| GAGAGAGAACACACACTAGC | 87693483 | 87693505 | 0.03 |
| GGTTTTGTCTTTTAATTTTA | 87693505 | 87693527 | 0.03 |
| GCTCTGCAGGCTAAGGCAACT | 87693526 | 87693548 | 0.01 |
| GTCTGGAACTCTGCAGGCTA | 87693534 | 87693556 | 0.74 |
| GAGTACTGGATGCCCTGTC | 87693551 | 87693572 | 2.34 |
| GGCATCCAGTACTCTGTAAT | 87693559 | 87693581 | 1.15 |
| GTAACTCCAATTACAGAGTAC | 87693564 | 87693586 | 0.02 |
| GATAGTTGTGAGCTGGCATG | 87693589 | 87693611 | 0.03 |
| GCTGGCATGTGGTGCTGATA | 87693600 | 87693622 | 0.80 |
| GTGGTGCTGATATGGAACCC | 87693608 | 87693630 | 0.36 |
| GATATGGAACCCAGGCCCTC | 87693616 | 87693638 | 0.01 |
| GGGCTGACTGCTCTTCCAGA | 87693631 | 87693653 | 0.07 |
| GCTTCAGACAGAGGTTAAGA | 87693651 | 87693673 | 0.01 |
| GCGGAAAGGGCTTCAGACAG | 87693660 | 87693682 | 0.08 |
| GTAGATACACAGCTGCGGAAA | 87693673 | 87693695 | 5.16 |
| GAAACACTAGATACACAGCTG | 87693679 | 87693701 | 2.21 |
| GTTTCATTTTGTGCTACAC | 87693710 | 87693731 | 0.01 |
| GTTGTGCTACACAGGCTGTTT | 87693718 | 87693740 | 0.21 |
| GAAAACTTATTTTCAGAATAA | 87693749 | 87693771 | 0.03 |
| GTAAACATATGAAATAAAACA | 87693775 | 87693797 | 0.01 |
| GAAGTGTATGGCTCTGTATCA | 87693844 | 87693866 | 0.02 |
| GAAAACAAAAACTCAAGTGTA | 87693857 | 87693879 | 0.01 |
| GAATTACAAGTGTGAGGCAGG | 87693995 | 87694017 | 0.01 |
| GATGCATTAATTACAAGTGTG | 87694002 | 87694024 | 0.30 |
| GGGACAGAGGGACAGACAGA | 87694046 | 87694068 | 0.04 |
| GCCGAAGGGAAGGAGGGACAG | 87694059 | 87694081 | 0.01 |
| GTCTCTTCCGAAGGGAAGGA | 87694066 | 87694088 | 0.99 |
| GATCTTACTGTCTCTTCCGAA | 87694074 | 87694096 | 0.96 |
| GGAAGAGACAGTAAGATATC | 87694080 | 87694102 | 0.01 |
| GCAGTAAGATATCAGGTCTCC | 87694088 | 87694110 | 0.05 |
| GACTACATTTACTCTAGCTCC | 87694106 | 87694128 | 1.08 |
| GTAGTTGTTTGCTGCCTAGTG | 87694125 | 87694147 | 0.11 |
| GTAGTTCCAAGAACCCACACT | 87694138 | 87694160 | 0.78 |
| GACTGTACGCTGCTCCCGCAG | 87694170 | 87694192 | 0.86 |
| GAAATGCATTAACTAAGGAGC | 87694202 | 87694224 | 2.16 |
| GCTTTCTAAATGCATTAACTA | 87694208 | 87694230 | 2.25 |
| GATGCATTTAGAAAGATTGTA | 87694217 | 87694239 | 0.02 |
| GTTTAGAAAGATTGTAAGGAC | 87694222 | 87694244 | 0.02 |

| | | | |
|---|---|---|---|
| GAGAGCCGGCTAAGTGTGTA | 87694335 | 87694357 | 0.17 |
| GCTCCACCTTACACACTTAGC | 87694340 | 87694362 | 0.26 |
| GCCAGTCCTTACACGCTTAGC | 87694398 | 87694420 | 0.65 |
| GAAAGCCGGCTAAGCGTGTA | 87694511 | 87694533 | 0.24 |
| GCGAGCCGGCCAAGCGTGTA | 87694569 | 87694591 | 0.56 |
| GCTCCACCTTACACGCTTGGC | 87694574 | 87694596 | 1.06 |
| GAATGCTCTTACACGCTTAGC | 87694922 | 87694944 | 0.03 |
| GGTTTGATTTCCAGCTTCTG | 87694965 | 87694987 | 1.96 |
| GTTGTGATGGCCACAGAAGC | 87694975 | 87694997 | 0.15 |
| GGAGTTTGGAATGGTTGTGA | 87694988 | 87695010 | 2.43 |
| GCCTAGAACTGGAGTTTGGAA | 87694997 | 87695019 | 0.01 |
| GATACCCTAGAACTGGAGTT | 87695002 | 87695024 | 0.03 |
| GTTATCAGATACCCTAGAAC | 87695009 | 87695031 | 0.97 |
| GATAACCTCTTCTGACTTCTG | 87695027 | 87695049 | 0.73 |
| GTTTTATATAAAATGCATCCT | 87695053 | 87695075 | 0.02 |
| GTTAAAGATAACTAAATCTAA | 87695076 | 87695098 | 0.01 |
| GATAACTAAATCTAAAGGGG | 87695081 | 87695103 | 0.01 |
| GGGGGGGATGCTGACAAGT | 87695102 | 87695123 | 0.01 |
| GGATGCTGACAAGTTGGCTG | 87695107 | 87695129 | 0.00 |
| GATTCTGTATCCCAGCTGCTC | 87695137 | 87695159 | 5.75 |
| GCCTCGGCCTCCTGAGCAGC | 87695147 | 87695169 | 0.00 |
| GAAAAATCGATGTTCCTGCCT | 87695163 | 87695185 | 0.01 |
| GTACAGCCTTGTTTCTAAGAG | 87695198 | 87695220 | 0.01 |
| GTTTCTAAGAGAGGAATGAA | 87695207 | 87695229 | 0.34 |
| GTCTGAAATTGAAGCAAGAGT | 87695230 | 87695252 | 0.82 |
| GTATTTTAAATTTGTAATTA | 87695264 | 87695286 | 0.05 |
| GAATTTGTAATTATGGATATG | 87695272 | 87695294 | 0.01 |
| GTCTGTTGACATGCGACTAT | 87695295 | 87695317 | 1.81 |
| GCGACTATAGGTGCCCATGG | 87695307 | 87695329 | 0.11 |
| GTTTCAGACTCTGGCCTCCA | 87695321 | 87695343 | 0.70 |
| GCTCCAAGGGTTTCAGACTC | 87695330 | 87695352 | 17.21 |
| GAAACTGTGAGCTGCCCAGTG | 87695366 | 87695388 | 0.01 |
| GTCAACGTTGAGACCCACACT | 87695379 | 87695401 | 0.87 |
| GTGGGTCTCAACGTTGAACC | 87695385 | 87695407 | 6.78 |
| GCATAAATTGTTCTTGCAG | 87695410 | 87695431 | 4.22 |
| GACTGAGCCATCTCGCCAACC | 87695440 | 87695462 | 0.02 |
| GCATTCCAGGTTGGCGAGA | 87695446 | 87695467 | 1.48 |
| GTTAAACTCAGCATTCCAGGT | 87695454 | 87695476 | 4.46 |
| GCTGAGTTTAAATGAAACTTT | 87695467 | 87695489 | 0.45 |
| GAAATCATTTAACAGCTTT | 87695496 | 87695517 | 0.27 |
| GTAAATGATTTCAGAGTGTGG | 87695507 | 87695529 | 5.38 |
| GTGTGGTGGATCTTTTGAC | 87695521 | 87695542 | 0.01 |
| GCTTTTGACTGGTAACAGACA | 87695532 | 87695554 | 0.17 |
| GCACCTTCATTACCTGTTTT | 87695569 | 87695591 | 0.14 |
| GCTTTTTCGCATTCCCAAAAC | 87695581 | 87695603 | 0.01 |
| GTCAAGGCAGAGCATGGACAC | 87695616 | 87695638 | 0.02 |
| GCTTGACATCAAGGCAGAGCA | 87695623 | 87695645 | 0.05 |
| GCCACAGACCCCTTGACATCA | 87695633 | 87695655 | 0.36 |
| GGCCTTGCCTTAGCTTTTGC | 87695655 | 87695677 | 0.01 |
| GGAAAGCCCTGCAAAAGCTA | 87695662 | 87695684 | 0.15 |
| GTTGCAGGGCTTTCCAGGCTG | 87695671 | 87695693 | 2.12 |
| GTTCCAGGCTGTGGTAGTTAA | 87695681 | 87695703 | 0.01 |

| | | | |
|---|---|---|---|
| GCAGTCTTTAAAAATCCAGT | 87695709 | 87695731 | 9.91 |
| GTTAAAAATCCAGTTGGAAAT | 87695716 | 87695738 | 0.02 |
| GCTTAGTTGCCAATTTCCAAC | 87695724 | 87695746 | 0.00 |
| GAATTGGCAACTAAGGTTCAG | 87695733 | 87695755 | 3.95 |
| GTTCAGTGGAGGTTAAAACTC | 87695748 | 87695770 | 1.96 |
| GGAGGTTAAAACTCAGGGA | 87695754 | 87695775 | 0.33 |
| GAAACTCAGGGAAGGAGGCTG | 87695762 | 87695784 | 19.67 |
| GTGGTCATGAAGATTCCAGG | 87695785 | 87695807 | 0.81 |
| GCCTCTCTAATGCAGACCTCC | 87695800 | 87695822 | 0.14 |
| GGTCTGCATTAGAGAGGGTA | 87695806 | 87695828 | 1.84 |
| GAGAGGGTAGGGGACACCG | 87695817 | 87695838 | 32.63 |
| GTAGGGGACACCGAGGATGAC | 87695824 | 87695846 | 3.50 |
| GTGAGGCTCCTGTCATCCT | 87695833 | 87695854 | 0.36 |
| GAAACAGCTGTTGCAGATGTG | 87695849 | 87695871 | 1.76 |
| GTCTGCAACAGCTGTTTTTTG | 87695856 | 87695878 | 0.71 |
| GTAGTGTATAGTGACTGTT | 87695880 | 87695901 | 1.49 |
| GTATAGTGACTGTTCGGCGT | 87695885 | 87695907 | 0.01 |
| GCCTGTTGCTGTGTCGTCTC | 87695913 | 87695935 | 10.38 |
| GTCAGGCACGCACCTATCCTC | 87695931 | 87695953 | 0.24 |
| GATGACAACTACCCAGAGGAT | 87695942 | 87695964 | 1.09 |
| GTCACTACCTTGTGCAGACA | 87695966 | 87695988 | 10.48 |
| GCCCTGTCCTTGTCTGCACA | 87695973 | 87695995 | 0.62 |
| GACAAGGACAGGGCTCAGGAC | 87695983 | 87696005 | 0.45 |
| GCTCAGGACAGGGCTGTGCTG | 87695995 | 87696017 | 2.86 |
| GAAGCCACAGAGCAGCATGCC | 87696024 | 87696046 | 1.11 |
| GTCAGGGCTGGCGCTAGCCC | 87696042 | 87696064 | 0.01 |
| GCAAGAAGGCAGAGTCAGGGC | 87696054 | 87696076 | 15.41 |
| GATGTGCAAGAAGGCAGAGTC | 87696059 | 87696081 | 0.31 |
| GGAACCAACAATGTGCAAGA | 87696069 | 87696091 | 3.12 |
| GAATCGTGGGAGGAGGGGAAC | 87696090 | 87696112 | 0.25 |
| GCCGAGGAATCGTGGGAGGAG | 87696096 | 87696118 | 0.71 |
| GAGCCATCCCGAGGAATCGT | 87696104 | 87696126 | 0.84 |
| GGCGGTTGAGAGCCATCCCG | 87696113 | 87696135 | 0.38 |
| GTGCTTAAATTGGAACTTGG | 87696131 | 87696153 | 2.71 |
| GCTGAATCCAGTGCTTAAAT | 87696141 | 87696163 | 3.32 |
| GGATTCAGCCTCTGCTGAT | 87696155 | 87696176 | 3.31 |
| GCCTCTGCTGATTGGAGCGC | 87696162 | 87696184 | 2.09 |
| GTTGGAGCGCTGGGTTGTTAA | 87696173 | 87696195 | 1.98 |
| GCTGGGTTGTTAATGGAAATT | 87696181 | 87696203 | 0.36 |
| GGAAATTAGGATGCAAATT | 87696194 | 87696215 | 11.03 |
| GCAAATTAGGTCTTATTCTAG | 87696207 | 87696229 | 0.41 |
| GTATTCTAGTGGCTTTGTCTT | 87696219 | 87696241 | 0.30 |
| GAATGTTTCCACACCCTCTGA | 87696259 | 87696281 | 2.42 |
| GGGTCTCCTTCAGAGGGTG | 87696266 | 87696287 | 1.76 |
| GCAGGTCTGGGTCTCCTTCAG | 87696272 | 87696294 | 5.28 |
| GAAGGAGACCCAGACCTGCA | 87696277 | 87696299 | 0.01 |
| GACTGCCCTCCCTGCAGGTCT | 87696285 | 87696307 | 1.13 |
| GTAGAGCACTGCCCTCCCTGC | 87696291 | 87696313 | 4.37 |
| GAGGGCAGTGCTCTAGTAGT | 87696299 | 87696321 | 2.44 |
| GTGCTCTAGTAGTTGGGCCT | 87696306 | 87696328 | 0.01 |
| GGCCTTGGGCACTACCACAG | 87696321 | 87696343 | 5.33 |
| GCTATAGACAGGGGACCACTG | 87696335 | 87696357 | 0.91 |

| | | | |
|---|---|---|---|
| GAAGGGTATCATCTATAGACA | 87696346 | 87696368 | 1.08 |
| GCTTTTATGCTTTAACTCAAA | 87696364 | 87696386 | 11.65 |
| GAGAATTGAGTTTAGAGACC | 87696386 | 87696408 | 9.64 |
| GCCAGAAAGCACAAATTGTCC | 87696404 | 87696426 | 0.48 |
| GATTTGTGCTTTCTGGTTCAA | 87696412 | 87696434 | 0.00 |
| GTTCTGTCTCTGCTTTGTTTT | 87696472 | 87696494 | 0.01 |
| GCTTTGTTTTAGGAAGACACC | 87696483 | 87696505 | 11.42 |
| GAAGACACCAGGCACTGTTGT | 87696495 | 87696517 | 4.37 |
| GAAACTGCCAACAACAGTGCC | 87696501 | 87696523 | 12.22 |
| GTCAGAACGAAGATCAATAAG | 87696532 | 87696554 | 4.10 |
| GATAGAAACAACTTTAGATA | 87696576 | 87696598 | 2.69 |
| GAACAACTTTAGATATGGATC | 87696582 | 87696604 | 9.10 |
| GTTCCCACAGCAAAGTATTTT | 87696623 | 87696645 | 0.01 |
| GTATGATTTTTCTGTTGTTTC | 87696660 | 87696682 | 0.02 |
| GTCTTGAGTTTGTATTTTTT | 87696701 | 87696723 | 0.01 |
| GTATTTTTTTGGCACTTTGTG | 87696713 | 87696735 | 0.01 |
| GTTTACATCAAGTGGCCACAG | 87696738 | 87696760 | 0.28 |
| GTCACTTCAGTTTACATCAAG | 87696747 | 87696769 | 6.35 |
| GTAAACTGAAGTGACAGACA | 87696756 | 87696778 | 0.19 |
| GAAGTGACAGACAAGGAAGAG | 87696764 | 87696786 | 8.02 |
| GGAAGAGTGGACTTGCCAGG | 87696777 | 87696799 | 1.74 |
| GTGGACTTGCCAGGCGGTGC | 87696783 | 87696805 | 1.15 |
| GTTTGATTCCCCAGCACCGCC | 87696792 | 87696814 | 2.96 |
| GGCACCACATTCAAGGCCCT | 87696815 | 87696837 | 8.73 |
| GGCCTTGAATGTGGTGCCCT | 87696820 | 87696842 | 0.96 |
| GCACCTTATTCTCGACCCCAA | 87696836 | 87696858 | 2.27 |
| GGGTCGAGAATAAGGTGGAG | 87696842 | 87696864 | 5.11 |
| GAGAATAAGGTGGAGTGGGT | 87696847 | 87696869 | 9.48 |
| GAGTGGGTGGGCTTTTACTA | 87696859 | 87696881 | 2.47 |
| GTACTAAGGCATACTTAGAAT | 87696874 | 87696896 | 4.65 |
| GCTTAGAATTGGTGGAGATGT | 87696886 | 87696908 | 1.68 |
| GTTGGTGGAGATGTTGGCCCT | 87696893 | 87696915 | 0.31 |
| GATGTTGGCCCTTGGCTCTG | 87696901 | 87696923 | 5.52 |
| GCAGGCATTCCACAGAGCCAA | 87696909 | 87696931 | 0.01 |
| GCTCTGTGGAATGCCTGGCTA | 87696916 | 87696938 | 9.30 |
| GTAGGCTGAGCTGCCTTAGCC | 87696928 | 87696950 | 1.68 |
| GCAGCTCAGCCTACTGTGAAG | 87696939 | 87696961 | 0.50 |
| GTGAAGAGGTCCCCGCTGGG | 87696953 | 87696975 | 0.82 |
| GAAGCAAACTGCCTCCCAGC | 87696964 | 87696986 | 0.86 |
| GGGAGGCAGTTTGCTTCAC | 87696970 | 87696991 | 4.13 |
| GTTTGCTTCACCGGTTTCAG | 87696978 | 87697000 | 0.37 |
| GTGGTCAACTCCACTGAAAC | 87696988 | 87697010 | 2.70 |
| GCCTCCAAGCAGCTTAGGCTG | 87697007 | 87697029 | 1.61 |
| GCAAACCTCCAAGCAGCTT | 87697013 | 87697034 | 1.88 |
| GCAAGCATCCATCCACAGAG | 87697043 | 87697065 | 0.03 |
| GCTCCCATCCTCTCTGTGGA | 87697051 | 87697073 | 3.91 |
| GAGAGGATGGGAGCAGCCC | 87697060 | 87697081 | 1.31 |
| GCTCAGCTCTCTTGGTTGCCT | 87697076 | 87697098 | 0.01 |
| GTTTCGCTCCTCAGCTCTCT | 87697085 | 87697107 | 1.34 |
| GGAGCGAAACACTGCCTTGT | 87697098 | 87697120 | 0.78 |
| GCCTTGTTGGGACAGAGACT | 87697111 | 87697133 | 1.72 |
| GTTGGGACAGAGACTAGGAA | 87697116 | 87697138 | 0.14 |

| | | | |
|---|---|---|---|
| GACATGATAGAGAAGTGTGCT | 87697159 | 87697181 | 0.30 |
| GTGTGCTAGGAATGAAGCT | 87697172 | 87697193 | 3.93 |
| GAAGCTTGGCCAGAAAAATG | 87697185 | 87697207 | 8.41 |
| GCAGGGCCGTCCTCATTTTTC | 87697194 | 87697216 | 0.02 |
| GTTCTCTCTCAAGGCTTCCA | 87697212 | 87697234 | 0.16 |
| GACACGGACAGTTCTCTCTCA | 87697221 | 87697243 | 0.51 |
| GGGCTTCGGGGCAGCTCACA | 87697238 | 87697260 | 3.88 |
| GACCCTGACACAGGGCTTCG | 87697250 | 87697272 | 0.21 |
| GCCTGGGTGGACCCTGACACA | 87697258 | 87697280 | 1.45 |
| GTCAGGGTCCACCCAGGTGA | 87697264 | 87697286 | 3.74 |
| GTCTCTTTGCCTTCACCTGGG | 87697272 | 87697294 | 1.08 |
| GCAAAGAGAAAGTCAATATGC | 87697287 | 87697309 | 0.18 |
| GATGCAGGGGGACAGTAAAGA | 87697303 | 87697325 | 0.39 |
| GGGGGACAGTAAAGAAGGGA | 87697308 | 87697330 | 2.63 |
| GAAAGAAGGGAGGGGTCCTTT | 87697318 | 87697340 | 0.91 |
| GGGGTCCTTTAGGTCCACAA | 87697328 | 87697350 | 4.39 |
| GCTTTACCATTGTGGACCTAA | 87697333 | 87697355 | 1.06 |
| GTCCACAATGGTAAAGTAGA | 87697340 | 87697362 | 4.86 |
| GGTAAAGTAGAAGGCAGAGG | 87697349 | 87697371 | 0.01 |
| GAAGGCAGAGGAGGAGTATT | 87697358 | 87697380 | 0.56 |
| GAGGAGGAGTATTAGGGAGG | 87697365 | 87697387 | 0.40 |
| GGGAGGAGGGTAGAAGTGT | 87697379 | 87697400 | 0.14 |
| GTAGAAGTGTTGGTAACCAC | 87697388 | 87697410 | 0.20 |
| GTTGGTAACCACAGGGTGGGT | 87697397 | 87697419 | 0.48 |
| GTGGTTCCAACCCACCCTG | 87697404 | 87697425 | 1.30 |
| GCAGTACATTAAATATGGCAG | 87697422 | 87697444 | 8.82 |
| GCCATATTTAATGTACTGTG | 87697427 | 87697449 | 0.50 |
| GATGTACTGTGTGGTAGGCAG | 87697437 | 87697459 | 2.53 |
| GCTCATGTACTGCGCGCGCT | 87697462 | 87697484 | 1.23 |
| GACTGCGCGCGCTGGGAGAGC | 87697470 | 87697492 | 0.90 |
| GGGAGAGCAGGAGCCCTGTG | 87697482 | 87697504 | 0.11 |
| GAGCCCTGTGAGGCCGTCAG | 87697492 | 87697514 | 0.06 |
| GATCTCGGACACTCCCACTGA | 87697505 | 87697527 | 2.13 |
| GTGGGAGTGTCCGAGATAGT | 87697511 | 87697533 | 3.28 |
| GCCATTACCCACCCACTATCT | 87697521 | 87697543 | 1.50 |
| GTAATGGCTTGAGACTGCAG | 87697537 | 87697559 | 0.08 |
| GCAGCGGTGGTTTTCAGAGA | 87697553 | 87697575 | 0.01 |
| GGTTTTCAGAGACGGGAAAC | 87697561 | 87697583 | 1.54 |
| GACGGGAAACTGGGGTCTGG | 87697571 | 87697593 | 1.59 |
| GAAACTGGGGTCTGGAGGGAA | 87697577 | 87697599 | 0.02 |
| GGGTCTGGAGGGAAAGGAAT | 87697583 | 87697605 | 1.12 |
| GGATAAAGACCAGCTGCGGT | 87697607 | 87697629 | 2.76 |
| GCTGCGGTGGGTAGCAGTCA | 87697619 | 87697641 | 0.61 |
| GGTGGGTAGCAGTCACGGTG | 87697624 | 87697646 | 0.01 |
| GTTCCACCCTTGAGAGTTAAC | 87697652 | 87697674 | 1.59 |
| GCAGATCCAGTTAACTCTCAA | 87697657 | 87697679 | 7.84 |
| GTTAACTGGATCTGTAGGAT | 87697666 | 87697688 | 6.34 |
| GGATCTGTAGGATAGGGACA | 87697673 | 87697695 | 0.01 |
| GATAGGGACAGGGGAGTCTG | 87697683 | 87697705 | 1.08 |
| GTCTGAGGTAGAGATCTGTG | 87697698 | 87697720 | 18.27 |
| GTAGAGATCTGTGAGGCAACT | 87697706 | 87697728 | 0.53 |
| GGCAACTAGGGAAAGAAGAT | 87697719 | 87697741 | 2.08 |

| | | | |
|---|---|---|---|
| GAAGAAGATGGGAATCAGGAA | 87697731 | 87697753 | 5.99 |
| GGGAATCAGGAAAGGTTATG | 87697739 | 87697761 | 1.75 |
| GCAGGAAAGGTTATGGGGGCC | 87697745 | 87697767 | 0.00 |
| GGGTGGCTTCAAAGTAGCTA | 87697766 | 87697788 | 0.02 |
| GCAAAGTAGCTACGGATTTCA | 87697775 | 87697797 | 2.43 |
| GGATTTCATGGCGGAGTAA | 87697787 | 87697808 | 1.22 |
| GCATGGCGGAGTAAAGGAACC | 87697793 | 87697815 | 7.05 |
| GTAAAGGAACCAGGCAGAC | 87697802 | 87697823 | 0.02 |
| GTCTGGTCATGCCTGTCTGCC | 87697811 | 87697833 | 1.06 |
| GATTCTTCTCAAGTGTTTC | 87697829 | 87697850 | 1.06 |
| GAATCACCCTACAAGAAGGC | 87697846 | 87697868 | 0.93 |
| GTTCTTCCCTGCCTTCTTGTA | 87697852 | 87697874 | 4.92 |
| GCAGGGAAGAAGAGGTGTAC | 87697864 | 87697886 | 0.05 |
| GAAGAAGAGGTGTACAGGAGG | 87697870 | 87697892 | 8.68 |
| GGAGGGGGTTCCATCTATGT | 87697885 | 87697907 | 0.50 |
| GCCTGTTCTTCCCCACATAGA | 87697895 | 87697917 | 0.02 |
| GTGGGGAAGAACAGGGTCTT | 87697903 | 87697925 | 0.02 |
| GAACAGGGTCTTAGGTGTAC | 87697911 | 87697933 | 0.46 |
| GGGTCTTAGGTGTACAGGAG | 87697916 | 87697938 | 0.09 |
| GACTTCGGGGGGGGGGCAAA | 87697944 | 87697966 | 0.02 |
| GATCACCAACTTCGGGGGGGG | 87697951 | 87697973 | 0.02 |
| GTATTATCACCAACTTCGGG | 87697956 | 87697978 | 0.74 |
| GTAATACTGTAGCATTGTCCT | 87697973 | 87697995 | 0.02 |
| GCTGTAGCATTGTCCTTGGCT | 87697978 | 87698000 | 0.09 |
| GCATTGTCCTTGGCTTGGCA | 87697983 | 87698005 | 1.35 |
| GTCGGTGACCCTGCCAAGCCA | 87697990 | 87698012 | 0.08 |
| GCTTGGCAGGGTCACCGAGTC | 87697996 | 87698018 | 4.11 |
| GTCTGGGAGCCTATGCTAGT | 87698013 | 87698035 | 0.01 |
| GGAGCCTATGCTAGTGGGGC | 87698018 | 87698040 | 1.30 |
| GGGCTGGGCCTCTCTGTGAC | 87698034 | 87698056 | 1.21 |
| GTTCTGTGCCCAGTCACAGAG | 87698042 | 87698064 | 0.21 |
| GCACAGAAGATGAGCCCACA | 87698057 | 87698079 | 0.42 |
| GAAGTCTGTATGCCCCCATG | 87698072 | 87698094 | 3.48 |
| GGGGGCATACAGACTTCTGA | 87698078 | 87698100 | 0.53 |
| GCATACAGACTTCTGATGGCC | 87698083 | 87698105 | 0.69 |
| GCTTCTGATGGCCAGGCTGCC | 87698091 | 87698113 | 0.01 |
| GGCCAGGCTGCCAGGGGGTT | 87698099 | 87698121 | 0.01 |
| GGAAGTCACGCCCAACCCCC | 87698109 | 87698131 | 0.24 |
| GTGACTTCCTGGGCTACTGA | 87698123 | 87698145 | 1.26 |
| GCGGCAACCCTCAGTAGCCC | 87698130 | 87698152 | 0.11 |
| GCTACTGAGGGTTGCCGCAGG | 87698136 | 87698158 | 0.01 |
| GCAGAAGGCTACCCCACCTG | 87698149 | 87698171 | 1.21 |
| GACAGGCTTGGCTACAGCAGA | 87698164 | 87698186 | 3.89 |
| GGCAGTTTCCTGACAGGCT | 87698177 | 87698198 | 2.82 |
| GGAGGTGGCAGTTTCCTGAC | 87698182 | 87698204 | 4.14 |
| GTCTATAAAAGCAAGAGAGG | 87698200 | 87698222 | 0.01 |
| GAAAGTCTATAAAAGCAAGAG | 87698203 | 87698225 | 0.01 |
| GTACGTGCGCCACCACGCC | 87698367 | 87698388 | 0.02 |
| GTTCCTTTGCTTTGCTTTTAA | 87698400 | 87698422 | 0.01 |
| GATTTTCATCAGCGTCTGTAC | 87698434 | 87698456 | 0.82 |
| GAAACTAAAGAAGGAAGAGGA | 87698467 | 87698489 | 3.57 |
| GTTGGAAAGAAACTAAAGA | 87698477 | 87698498 | 0.01 |

| | | | |
|---|---|---|---|
| GTTAGTTTCTTTCCAACTCTG | 87698483 | 87698505 | 0.01 |
| GTTCTTTCCAACTCTGTGGTT | 87698488 | 87698510 | 4.64 |
| GACAAGTCCTAACCACAGAGT | 87698494 | 87698516 | 1.10 |
| GGTTAGGACTTGTAGAAGTT | 87698504 | 87698526 | 0.01 |
| GGACTTGTAGAAGTTAGGTT | 87698509 | 87698531 | 0.03 |
| GCTTATTTCTGGGAGCATGGG | 87698543 | 87698565 | 1.01 |
| GATCTAGTCAGAGAAGAGCCA | 87698594 | 87698616 | 0.24 |
| GTCTTGTTCCATCTTCCCCG | 87698695 | 87698717 | 1.03 |
| GAAGATTCCCGCGGGGAAGA | 87698703 | 87698725 | 0.08 |
| GGCACGGGAAGATTCCCGCG | 87698710 | 87698732 | 0.22 |
| GTCTGGTACAGAGCCAGGCAC | 87698725 | 87698747 | 0.32 |
| GAGAATTCTGGTACAGAGCC | 87698731 | 87698753 | 0.29 |
| GCTGGGAGGCACAGAGAATTC | 87698743 | 87698765 | 0.01 |
| GAAATGGGAAGCGGGCTATC | 87698762 | 87698784 | 0.34 |
| GTTAGGCAGGAAATGGGAAGC | 87698770 | 87698792 | 0.01 |
| GCATTGCAGAGAGAATATTTT | 87698836 | 87698858 | 0.01 |
| GCTGCAATGACAGAAATAAAA | 87698851 | 87698873 | 0.02 |
| GCAGAAATAAAATGGGTAGCG | 87698860 | 87698882 | 0.03 |
| GAAAATGGGTAGCGAGGACCG | 87698867 | 87698889 | 1.55 |
| GAGGACCGTGGCCTCTGCTG | 87698879 | 87698901 | 0.08 |
| GGACTCAGCCTCCACAGCAG | 87698890 | 87698912 | 45.80 |
| GTGGAGGCTGAGTCCAGGC | 87698898 | 87698919 | 0.03 |
| GCCACGATGCAGTACCTGCC | 87698911 | 87698933 | 0.55 |
| GCAGCTTGCTGCGTAATTTCA | 87698945 | 87698967 | 5.01 |
| GAGTCAACTAAAGTTGACTT | 87698974 | 87698996 | 0.01 |
| GCTAACTCCATTCCCTGAAGG | 87699001 | 87699023 | 0.21 |
| GCTATTGCCTCCTTCAGGGAA | 87699007 | 87699029 | 2.65 |
| GATGACCTATTGCCTCCTTCA | 87699012 | 87699034 | 0.28 |
| GTCATAAGCTCCCTCCTCTGG | 87699031 | 87699053 | 0.06 |
| GTTAAGGTGGCCACCAGAGG | 87699041 | 87699063 | 0.03 |
| GGTGGCCACCTTAACCCGGC | 87699049 | 87699071 | 0.87 |
| GATTACCAGCCGGGTTAAGG | 87699054 | 87699076 | 0.93 |
| GACCCGGCTGGTAATCCCAGT | 87699062 | 87699084 | 6.08 |
| GTCAGCTAAAGAACCCAACT | 87699076 | 87699098 | 0.01 |
| GTTGGGTTCTTTAGCTGACCT | 87699081 | 87699103 | 0.01 |
| GACCTTGGCTTGTCACCCACT | 87699097 | 87699119 | 1.43 |
| GTGGACAAAACAGTCCCTAG | 87699112 | 87699134 | 0.69 |
| GTATGCAGAGTAGACCCAG | 87699131 | 87699152 | 8.08 |
| GGTAGGTTCGGGCGTCTGGC | 87699155 | 87699177 | 0.12 |
| GTCTCATTCCCGGGTAGGTTC | 87699166 | 87699188 | 0.38 |
| GGGGTTCCACTCTCATTCCC | 87699176 | 87699198 | 0.34 |
| GTCTGACCTGTACATTACACG | 87699195 | 87699217 | 0.01 |
| GATGTACAGGTCAGAGGTCAG | 87699204 | 87699226 | 0.03 |
| GCAGGTCAGAGGTCAGAGGCC | 87699209 | 87699231 | 1.94 |
| GAGGTCAGAGGCCTGGCAA | 87699216 | 87699237 | 0.00 |
| GCAGAGGCCTGGCAAAGGTAG | 87699221 | 87699243 | 0.05 |
| GAGTTCCACTACCTTTGCC | 87699227 | 87699248 | 4.90 |
| GTGGAACTCTTTACTCTAGA | 87699240 | 87699262 | 0.03 |
| GACTCTTTACTCTAGATGGAT | 87699245 | 87699267 | 0.23 |
| GCTCTAGATGGATAGGTTGGC | 87699253 | 87699275 | 4.74 |
| GATGGATAGGTTGGCAGGGA | 87699258 | 87699280 | 0.02 |
| GCCTCACCACCTTTCAGTCT | 87699282 | 87699304 | 0.16 |

| | | | |
|---|---|---|---|
| GACCACCTTTCAGTCTGGGTG | 87699287 | 87699309 | 0.25 |
| GCAGTCTGGGTGAGGTGGTGT | 87699296 | 87699318 | 0.00 |
| GTGAGGTGGTGTAGGGAGAA | 87699304 | 87699326 | 0.23 |
| GTGGTGTAGGGAGAAAGGAA | 87699309 | 87699331 | 0.03 |
| GGGAGAAAGGAACGGGAAT | 87699317 | 87699338 | 0.66 |
| GCAAGAGGTGGCAAGGGCTGA | 87699346 | 87699368 | 0.14 |
| GCCCTTGCCACCTCTTGGAG | 87699352 | 87699374 | 0.08 |
| GCCTCTGCCCACTCCAAGAGG | 87699359 | 87699381 | 0.01 |
| GTTGGAGTGGGCAGAGGAGGG | 87699366 | 87699388 | 0.01 |
| GAGTGCTGTAAGCAGCCACA | 87699398 | 87699420 | 0.45 |
| GCTGTAAGCAGCCACAGGGG | 87699402 | 87699424 | 0.04 |
| GCCACAGGGGAGGAGAATTC | 87699412 | 87699434 | 0.03 |
| GAATTCAGGCGGGGCAGACT | 87699426 | 87699448 | 0.01 |
| GGCGGGGCAGACTAGGGTGT | 87699433 | 87699455 | 0.02 |
| GCAGACTAGGGTGTGGGTTT | 87699439 | 87699461 | 0.01 |
| GTGGGTTTCGGGGTCATAGA | 87699451 | 87699473 | 0.01 |
| GTTTCGGGGTCATAGAAGGGG | 87699456 | 87699478 | 0.24 |
| GGGAGGGGGCAGAGTCGGCA | 87699473 | 87699495 | 0.33 |
| GGGCAGAGTCGGCACGGAGG | 87699479 | 87699501 | 0.75 |
| GTCGGCACGGAGGAGGGAAG | 87699486 | 87699508 | 1.53 |
| GATGGGGAGAAGGTTGAGAA | 87699508 | 87699530 | 0.26 |
| GGAGAAGGTTGAGAAAGGGG | 87699513 | 87699535 | 0.07 |
| GGGGAGGTGGCAAGTTAGAA | 87699529 | 87699551 | 0.44 |
| GCAAGTTAGAAGGGGAGCAG | 87699538 | 87699560 | 1.14 |
| GAAGGGGAGCAGTGGGAGGGG | 87699547 | 87699569 | 0.00 |
| GAGCAGTGGGAGGGGAGGAG | 87699552 | 87699574 | 0.03 |
| GTGGGAGGGGAGGAGAGGGC | 87699557 | 87699579 | 0.06 |
| GGCGGGGAAGCCAGTGACTC | 87699574 | 87699596 | 0.06 |
| GACTGAGCTGCCTGAGTCAC | 87699584 | 87699606 | 2.50 |
| GCTCAGGCAGCTCAGTCCTGG | 87699591 | 87699613 | 0.00 |
| GTCAGTCCTGGTGGATGACTC | 87699601 | 87699623 | 0.01 |
| GGTGGATGACTCAGGGTCC | 87699609 | 87699630 | 0.01 |
| GACTCAGGGTCCAGGTCCCC | 87699616 | 87699638 | 0.01 |
| GGTCCAGGTCCCCGGGTAAA | 87699623 | 87699645 | 0.40 |
| GTCTCGCTGGCCTTTTACCC | 87699633 | 87699655 | 0.18 |
| GCTTGACATCCCGTCTCGC | 87699646 | 87699667 | 0.01 |
| GAGACGGGATGTCAAGCAC | 87699651 | 87699672 | 0.76 |
| GTCCCCAAAAACTTGAACAGT | 87699688 | 87699710 | 1.11 |
| GACCTTGAAAATGTCTGCAT | 87699721 | 87699743 | 0.03 |
| GTTTACAGAGTGCCTTCTGG | 87699759 | 87699781 | 0.71 |
| GCTCTAGATAACCAATCTAAA | 87699782 | 87699804 | 0.15 |
| GATCTAGAGCATACTGAGATC | 87699797 | 87699819 | 0.10 |
| GACATCTGGCTCTTTGCTCTG | 87699820 | 87699842 | 1.26 |
| GAAGAGCCAGATGTGTTAGCC | 87699830 | 87699852 | 0.00 |
| GATGTGTTAGCCAGGTTCTG | 87699838 | 87699860 | 0.67 |
| GTGTTAGCCAGGTTCTGAGG | 87699841 | 87699863 | 1.17 |
| GATTGGGTCCACCTCAGAACC | 87699848 | 87699870 | 5.21 |
| GCTGAGAAATTTAGCTAAAT | 87699866 | 87699888 | 0.44 |
| GAAATTTCTCAGCAGACAGTT | 87699877 | 87699899 | 0.00 |
| GTTGGGGATTGATTTTAGATC | 87699896 | 87699918 | 0.01 |
| GCCCATTGTCCAGTCTTTGC | 87699923 | 87699945 | 0.01 |
| GATAAGCCAACCAGCAAAGAC | 87699932 | 87699954 | 0.10 |

| | | | |
|---|---|---|---|
| GGTTGGCTTATTTCTTGAG | 87699944 | 87699965 | 5.43 |
| GAGTGGAGAAAACCGCTTGA | 87699960 | 87699982 | 0.32 |
| GAAAACCGCTTGATGGCAAA | 87699967 | 87699989 | 1.73 |
| GATGGCAAATGGCAGGGCTC | 87699978 | 87700000 | 0.01 |
| GAATGGCAGGGCTCAGGATGT | 87699985 | 87700007 | 0.58 |
| GATGTTGGCTCAACTGCTAC | 87700000 | 87700022 | 0.09 |
| GTCTGGCTCTAGAATGGCTAC | 87700019 | 87700041 | 0.31 |
| GTAACCTTTCTGGCTCTAGAA | 87700026 | 87700048 | 0.18 |
| GCTAATCCCCTTAACCTTTC | 87700037 | 87700059 | 3.15 |
| GTAAGGGGATTAGCTCTTGGC | 87700047 | 87700069 | 0.02 |
| GCCGGTCATACTAACTTGAG | 87700065 | 87700087 | 0.11 |
| GTCATACTAACTTGAGTGGCA | 87700070 | 87700092 | 2.46 |
| GTCTCTAGGGATGGACTTAC | 87700098 | 87700120 | 4.81 |
| GCCGTGAAGGTCTCTAGGGA | 87700107 | 87700129 | 0.33 |
| GCCTGGCCGTGAAGGTCTCT | 87700112 | 87700134 | 1.29 |
| GTCTAGAAGCCTGGCCGTGA | 87700120 | 87700142 | 0.00 |
| GCCAGGCTTCTAGACGACTC | 87700128 | 87700150 | 0.01 |
| GCTCTGGGAGCTGACCCCGTG | 87700145 | 87700167 | 0.02 |
| GGAGCTGACCCCGTGAGGCC | 87700150 | 87700172 | 0.02 |
| GGGCATGGCCTGGCCTCACG | 87700158 | 87700180 | 0.34 |
| GAGGGAGGGGGGCATGGCC | 87700168 | 87700189 | 0.00 |
| GCCAGCAGAGGGAGGGGGGCA | 87700173 | 87700195 | 0.01 |
| GCAAAGCCCCAGCAGAGGGA | 87700181 | 87700203 | 0.06 |
| GAGGGCAAAGCCCCAGCAGA | 87700185 | 87700207 | 0.02 |
| GGGGCTTTGCCCTCTTGGGT | 87700194 | 87700216 | 0.11 |
| GAGGAAAAACCCCACCCAAG | 87700204 | 87700226 | 0.97 |
| GCACATAAAGAATGCAATGGG | 87700223 | 87700245 | 0.01 |
| GTGTGTTGTGCAGTGTGACT | 87700248 | 87700270 | 0.00 |
| GTGACTTGGTGGTGTTCTG | 87700262 | 87700283 | 0.20 |
| GTTCTGTGGCTCCCTATTGC | 87700275 | 87700297 | 0.01 |
| GATGCAAAGGTTCCGGCAAT | 87700287 | 87700309 | 0.01 |
| GGATTGGATGCAAAGGTTC | 87700294 | 87700315 | 0.13 |
| GTGTTACGGATTGGATGCAA | 87700300 | 87700322 | 0.01 |
| GCTGACGTGCGTGTTACGGAT | 87700309 | 87700331 | 0.09 |
| GGGTGCTGACGTGCGTGTTA | 87700314 | 87700336 | 0.09 |
| GCACGTCAGCACCCTCCAGA | 87700323 | 87700345 | 1.17 |
| GCACCCTCCAGAAGGAGGAG | 87700331 | 87700353 | 0.02 |
| GAAGGCCCCTCCTCCTTCTGG | 87700335 | 87700357 | 1.56 |
| GCCTTCAGGGTGAGCACTGC | 87700353 | 87700375 | 0.78 |
| GTGAGCACTGCAGGCTGGGG | 87700362 | 87700384 | 0.05 |
| GCAGGCTGGGGAGGGCTGCTG | 87700372 | 87700394 | 0.00 |
| GAACTGAGGAGTAACTGCTAC | 87700395 | 87700417 | 1.24 |
| GTCCCCAGTTGCAGTAACTG | 87700410 | 87700432 | 0.00 |
| GTCACAGTTTATAGTGTAGAA | 87700435 | 87700457 | 1.11 |
| GATAGTCACAGTCCTAATTAA | 87700464 | 87700486 | 0.27 |
| GCAGGAGTGCAGCCTTTAATT | 87700475 | 87700497 | 0.03 |
| GCACTCCTGGGGGCAGACCT | 87700489 | 87700511 | 0.01 |
| GCCTGCCTAGGTCTGCCCCC | 87700494 | 87700516 | 0.01 |
| GATACGGCTAGAGGCCTGCCT | 87700506 | 87700528 | 0.08 |
| GCAGGCCTCTAGCCGTATCTC | 87700512 | 87700534 | 2.84 |
| GTAGCCGTATCTCAGGTTATA | 87700520 | 87700542 | 0.03 |
| GACAAATCTCTGCGACGATGA | 87700556 | 87700578 | 2.44 |

| | | | |
|---|---|---|---|
| GATCGTCGCAGAGATTTGTAG | 87700561 | 87700583 | 0.10 |
| GTAGTTCTAAAAATGTAATC | 87700594 | 87700616 | 0.02 |
| GACTACTTTCAGAAATTCTCC | 87700612 | 87700634 | 0.38 |
| GCATCAACAACTGAGAGTCCC | 87700630 | 87700652 | 3.38 |
| GATGTCTAATCTTTGAAAGCC | 87700650 | 87700672 | 0.01 |
| GTTGTGCTCCAGCTGTGCTT | 87700672 | 87700694 | 0.01 |
| GTGAACTCCTAAGCACAGC | 87700680 | 87700701 | 3.44 |
| GTGCTTAGGAGTTCACGTGA | 87700686 | 87700708 | 0.18 |
| GGAGGGTCTCATTTACACAG | 87700707 | 87700729 | 0.02 |
| GTGGTGTGAGGGAAGGGCAC | 87700737 | 87700759 | 0.03 |
| GTTGGGGGGTGGTGTGAGGGA | 87700744 | 87700766 | 0.01 |
| GAAACACTTACTTGGGGGG | 87700756 | 87700777 | 0.14 |
| GCCACAGAAACACTTACTTG | 87700761 | 87700783 | 0.00 |
| GAAAAAAAGAGAGGACTTA | 87700790 | 87700811 | 0.02 |
| GTAGGAAAAAGAAAAAAAGAG | 87700798 | 87700820 | 9.89 |
| GTTGATCTACATTTCTAAAAT | 87700817 | 87700839 | 0.02 |
| GCCTTTTTGGAATTTTGGCTT | 87700846 | 87700868 | 0.00 |
| GTCAATGCCTTTTTGGAATTT | 87700852 | 87700874 | 0.01 |
| GTTTTTAAATCAATGCCTTTT | 87700860 | 87700882 | 0.01 |
| GTATAATTCACTGAAACACAA | 87700906 | 87700928 | 0.04 |
| GTATTAATTTTTAAAGATTTT | 87700927 | 87700949 | 0.04 |
| GATTGGATATGTTCAAAATAA | 87700969 | 87700991 | 0.03 |
| GAAGTGGAAAGAAATGCAT | 87700987 | 87701008 | 0.01 |
| GTTTCCACTTCTCCTCCGTGA | 87700999 | 87701021 | 0.48 |
| GATTAGGAGGTATGGCTGTGT | 87701055 | 87701077 | 0.01 |
| GGTGAGCTTTGAGGGCTCC | 87701098 | 87701119 | 0.24 |
| GCTGGGCAGAGTGTGAGTACC | 87701115 | 87701137 | 0.01 |
| GTACTCACACTCTGCCCAGTG | 87701120 | 87701142 | 0.08 |
| GGACTGACTTCTTCCACACT | 87701133 | 87701155 | 2.24 |
| GTCAGTCCTGGTCATCTCC | 87701148 | 87701169 | 0.44 |
| GAAGGCAGCCAGGAGATGACC | 87701154 | 87701176 | 1.28 |
| GCTTCCTGCCATGATATTAG | 87701239 | 87701261 | 0.10 |
| GCTTCAGTCCACTAATATCA | 87701247 | 87701269 | 0.01 |
| GCATGTCCAAGGCAACTCATA | 87701305 | 87701327 | 0.01 |
| GACGAATAGACGCCATGTCCA | 87701317 | 87701339 | 3.58 |
| GATGGCGTCTATTCGTCGCAA | 87701325 | 87701347 | 0.01 |
| GAAATGTAACTAAGACACCCT | 87701348 | 87701370 | 0.33 |
| GAACATGACAATGAAGACCAA | 87701364 | 87701386 | 0.01 |
| GAATTAAAAAAGGATGGAA | 87701397 | 87701418 | 0.02 |
| GAAATAGAAGTGAATTAAAAA | 87701406 | 87701428 | 0.12 |
| GCACTTCTATTTCATGTGCAT | 87701418 | 87701440 | 0.00 |
| GTCTGAAATTCCAGTTCTAG | 87701480 | 87701502 | 0.29 |
| GGCCGGATGCCTGCATGCTC | 87701524 | 87701546 | 0.01 |
| GAAGGCATCCCTGAGCATGC | 87701533 | 87701555 | 0.01 |
| GGGATGCCTTCCCCTTCCCT | 87701545 | 87701567 | 0.04 |
| GACAGCTCCCAGGGAAGGGGA | 87701551 | 87701573 | 0.04 |
| GCAGTTACAGCTCCCAGGGAA | 87701556 | 87701578 | 2.52 |
| GACTGACAGTTACAGCTCCCA | 87701561 | 87701583 | 0.21 |
| GCAGTGGTGCCGCATGTAGCC | 87701580 | 87701602 | 0.01 |
| GACGGGGATCCTGGCTACATG | 87701588 | 87701610 | 1.98 |
| GCAGGGGCTCACGGGGATCC | 87701598 | 87701620 | 0.02 |
| GCACGGATGCAGGGGCTCAC | 87701606 | 87701628 | 0.01 |

| | | | |
|---|---|---|---|
| GACATCCCTGCACGGATGCAG | 87701614 | 87701636 | 0.13 |
| GATCCGTGCAGGGATGTGTAC | 87701621 | 87701643 | 0.01 |
| GCAGGGATGTGTACTGGCT | 87701627 | 87701648 | 0.69 |
| GTGTACTGGCTCGGCATTA | 87701635 | 87701656 | 0.05 |
| GCTAACTGTACCTGCTATG | 87701661 | 87701682 | 0.02 |
| GCCAGTGCTCTCCTCATAGC | 87701671 | 87701693 | 0.01 |
| GGAGTGCAGGAAATGCTGTA | 87701704 | 87701726 | 0.36 |
| GCAGAGGATGGGAAGGAGTGC | 87701717 | 87701739 | 0.31 |
| GTTAAGCGGCAGAGGATGGGA | 87701725 | 87701747 | 0.12 |
| GTGTGTTAAGCGGCAGAGGA | 87701730 | 87701752 | 0.41 |
| GGGTGTTAAGTGTGTTAAG | 87701740 | 87701761 | 1.53 |
| GGGTGACATCGTGGGAGAAT | 87701759 | 87701781 | 1.38 |
| GGTAACCCGGGTGACATCGT | 87701767 | 87701789 | 0.01 |
| GACGGTGGAGGGTAGGTAACC | 87701780 | 87701802 | 2.32 |
| GCACACACACGGTGGAGGGT | 87701788 | 87701810 | 0.01 |
| GCCATGCACGCACACACACGG | 87701796 | 87701818 | 0.03 |
| GTTCTCTCTGTAGTGGTCAGT | 87701896 | 87701918 | 0.01 |
| GAGAAGCTTCTCTCTGTAG | 87701904 | 87701925 | 0.03 |
| GCTTCTCTGACCATCGCTG | 87701919 | 87701940 | 0.20 |
| GATCAGTGCTGTCCTCAGCGA | 87701929 | 87701951 | 0.01 |
| GCTGAGGACAGCACTGATCT | 87701934 | 87701956 | 5.67 |
| GATCTGGGTGTAAGCATAAAT | 87701950 | 87701972 | 0.02 |
| GTAAGCATAAATTGGGAAT | 87701958 | 87701979 | 0.01 |
| GCAGTTTGATGTGTCCGTTT | 87701979 | 87702001 | 0.83 |
| GATTACTGCTGTTTTCCTAAA | 87701993 | 87702015 | 1.27 |
| GTAATAGTTGTCCCCGCCTG | 87702011 | 87702033 | 0.30 |
| GAGATCACCAGCCCCAGGCG | 87702022 | 87702044 | 3.28 |
| GCTAGGGAGATCACCAGCCCC | 87702027 | 87702049 | 0.07 |
| GCTGGTGATCTCCCTAGCTG | 87702033 | 87702055 | 0.25 |
| GCTGGTCAGAAGACCTCAGCT | 87702045 | 87702067 | 0.17 |
| GCTGACCAGGTTTACAGTACC | 87702060 | 87702082 | 0.80 |
| GCACAGGAGGGAATTCCTACC | 87702078 | 87702100 | 0.37 |
| GGAATTCCCTCCTGTGGATT | 87702085 | 87702107 | 0.01 |
| GGATTTGAGGCCTAATCCAC | 87702095 | 87702117 | 0.00 |
| GAAATCCAATCCTAAAGCCGT | 87702112 | 87702134 | 0.59 |
| GATAACCAACGGCTTTAGGAT | 87702116 | 87702138 | 4.03 |
| GTTTTATGGGAATAACCAA | 87702128 | 87702149 | 0.02 |
| GAAGATCAGCATGAAGTTTTA | 87702141 | 87702163 | 0.03 |
| GAAACTTCATGCTGATCTTAG | 87702146 | 87702168 | 0.05 |
| GACACAAGCATGTGAGATGA | 87702187 | 87702209 | 1.45 |
| GTTCTACAAGGCTGAGCTTA | 87702232 | 87702254 | 0.60 |
| GTTAAGAAAATGTTCTACA | 87702244 | 87702265 | 10.81 |
| GGAAGGGGCCCATCCCACTA | 87702278 | 87702300 | 0.03 |
| GGGCCCATCCCACTATGGGT | 87702283 | 87702305 | 0.03 |
| GGGGTGGCCCTACCCATAGT | 87702291 | 87702313 | 0.03 |
| GGGTAGGGCCACCCCTGAGA | 87702299 | 87702321 | 0.06 |
| GAGCCTGGGACCCCATCTCA | 87702311 | 87702333 | 2.02 |
| GCCTGCTTTCTCACAGAGCC | 87702326 | 87702348 | 0.01 |
| GCAGGCTGAGCAAGCCATGCA | 87702344 | 87702366 | 0.01 |
| GTGCTGCTTGCTGCCTTGCA | 87702357 | 87702379 | 0.21 |
| GCTGATGCGGACGCCGTGGA | 87702380 | 87702402 | 0.01 |
| GAACTAAAACGGCAGGAACC | 87702411 | 87702433 | 0.96 |

| | | | |
|---|---|---|---|
| GAATCAGGACAAGAACTAAAA | 87702422 | 87702444 | 0.01 |
| GTCCTGATTTCTGTCAGTGG | 87702436 | 87702458 | 0.15 |
| GTCAGTGGTGGGCCACAGTG | 87702448 | 87702470 | 0.01 |
| GAATCACACTTCCACACTG | 87702460 | 87702481 | 0.27 |
| GACATAAGCCCTTGCTTCCCC | 87702482 | 87702504 | 3.95 |
| GATGGGGCACCAGGGGAAGCA | 87702490 | 87702512 | 0.53 |
| GATTGCTGAGATGGGGCACC | 87702500 | 87702522 | 0.35 |
| GGGTTACGATTGCTGAGATG | 87702507 | 87702529 | 0.63 |
| GCAGCAATCGTAACCCTAACT | 87702515 | 87702537 | 0.02 |
| GCAACAGCAGTGTCCTAGTT | 87702528 | 87702550 | 0.93 |
| GGACACTGCTGTTGCTCCAG | 87702536 | 87702558 | 0.07 |
| GAACAAACAAGATTTGCCCAC | 87702552 | 87702574 | 0.09 |
| GCAAATCTTGTTTGTTGATG | 87702559 | 87702581 | 0.29 |
| GTTGTTTGTTGATGAGGATTG | 87702566 | 87702588 | 0.28 |
| GCAGTGTCCACTTTTATGCT | 87702592 | 87702614 | 0.02 |
| GTGCTTCCAAGCATAAAAG | 87702599 | 87702620 | 0.20 |
| GCTTTTACCAACTGAGCTAGC | 87702620 | 87702642 | 0.01 |
| GCTGGCCAGCTAGCTCAGT | 87702626 | 87702647 | 0.01 |
| GAAAAAAAGAAACACTGGAGC | 87702643 | 87702665 | 0.01 |
| GCTGAAAAAAAAAAGAAACAC | 87702649 | 87702671 | 4.91 |
| GAATGGAGCAGAAATAGGAAC | 87702672 | 87702694 | 0.02 |
| GCTATTTCTGCTCCATTCTGG | 87702679 | 87702701 | 0.65 |
| GGACTGTTAGTCCTCCAGAA | 87702690 | 87702712 | 0.04 |
| GGCAGAGGGGGCAAGCTAAG | 87702711 | 87702733 | 0.42 |
| GGTCTTTCTCACGGCAGAGG | 87702723 | 87702745 | 2.18 |
| GTGAAGCATGGTCTTTCTCA | 87702732 | 87702754 | 0.45 |
| GTAATCTGTGAGGTGAAGCA | 87702744 | 87702766 | 0.13 |
| GTTCACCTCACAGATTACAGT | 87702750 | 87702772 | 0.94 |
| GACAGATTACAGTAGGTGTGG | 87702758 | 87702780 | 0.01 |
| GCTCCTGACTTGCTCCTTCAC | 87702788 | 87702810 | 0.01 |
| GTGAGAAGCTGAGCCGGTGA | 87702801 | 87702823 | 0.41 |
| GAGCCGGTGAGAAGCTGAGC | 87702807 | 87702829 | 0.04 |
| GAGCTAGAGAGAAGCTGAGC | 87702823 | 87702845 | 0.39 |
| GCTTACTCTCTTATGTGCCTC | 87702848 | 87702870 | 1.41 |
| GTGCCTCAGGGCTACTGTCC | 87702861 | 87702883 | 0.45 |
| GGCAACACCCACAGTGAGCG | 87702887 | 87702909 | 3.62 |
| GTAAGGGCCCCGCTCACTGT | 87702894 | 87702916 | 0.06 |
| GAGGATGACTTGATGTGTAA | 87702910 | 87702932 | 0.01 |
| GCAGGTAGGCAAGGCATGGG | 87702929 | 87702951 | 0.13 |
| GAAGTCGAGCAGGTAGGCA | 87702938 | 87702959 | 0.64 |
| GCCCCTCAATGAAGTCGAGC | 87702947 | 87702969 | 5.00 |
| GAGGGGCTTCCTTCTTCTC | 87702963 | 87702984 | 0.81 |
| GTAGTTTGTCACCTGAGAAGA | 87702972 | 87702994 | 1.01 |
| GGGCAGGGTTCAATACATAG | 87703008 | 87703030 | 0.11 |
| GCTATGTATTGAACCCTGCCC | 87703011 | 87703033 | 0.34 |
| GAAGAAAGGAGGAACCCGGGC | 87703024 | 87703046 | 0.02 |
| GCAGGAAAGAAAGGAGGAACC | 87703029 | 87703051 | 0.35 |
| GATTAGTCAGGAAAGAAAGG | 87703036 | 87703058 | 0.12 |
| GTTAGGAGCTAGGATTAGTC | 87703048 | 87703070 | 0.01 |
| GGGGTCAGGGGTTAGGAGCT | 87703058 | 87703080 | 0.69 |
| GTTTAGGGGTTAGGGGTCAG | 87703070 | 87703092 | 0.02 |
| GAAGTTAGGTTTAGGGGTTAG | 87703077 | 87703099 | 0.15 |

| | | | |
|---|---|---|---|
| GCCAGTTCCAAGTTAGGTTT | 87703086 | 87703108 | 0.01 |
| GTACACAGCCAGTTCCAAGTT | 87703092 | 87703114 | 1.05 |
| GGAACTGGCTGTGTATAGAT | 87703100 | 87703122 | 0.01 |
| GTGTATAGATAGGCAAGTT | 87703110 | 87703131 | 0.02 |
| GAAGACAGGCAACTGTACATG | 87703132 | 87703154 | 1.78 |
| GTGCTGGGATTAAAAGCTTG | 87703165 | 87703187 | 0.08 |
| GAAGCTTGTGGAGCCATGTTC | 87703178 | 87703200 | 0.01 |
| GTCTACTTCATGCCAGAACA | 87703190 | 87703212 | 0.04 |
| GACTTTGAAATGATCTCCC | 87703210 | 87703231 | 0.01 |
| GATCTCCCTGGTTTTATGT | 87703221 | 87703242 | 0.01 |
| GATTCTCCAACATAAAACCA | 87703226 | 87703248 | 0.04 |
| GTATGTTGGAGAATCTTTACT | 87703235 | 87703257 | 1.49 |
| GTTTACTTGGCATCAGCTAGA | 87703249 | 87703271 | 0.00 |
| GTTTCTAAACAAATGATCAGC | 87703281 | 87703303 | 0.03 |
| GAATATTTTATCTAATACTTC | 87703315 | 87703337 | 0.06 |
| GATCTAATACTTCTGGGGTAG | 87703323 | 87703345 | 0.01 |
| GATTGTCTAAGACATATCCT | 87703360 | 87703382 | 0.71 |
| GCTGAGTCAACTCTCTAGCCC | 87703377 | 87703399 | 0.03 |
| GTCATGTACGTGTTCTTGTAA | 87703404 | 87703426 | 0.01 |
| GTCTTGTAAAGGACCAAGCTT | 87703416 | 87703438 | 0.04 |
| GAAGGACCAAGCTTTGGTTTC | 87703423 | 87703445 | 0.01 |
| GGGTGCCGGAAACCAAAGCT | 87703428 | 87703450 | 0.08 |
| GTTTGGTTTCCGGCACCCACA | 87703434 | 87703456 | 0.04 |
| GTTTCCGGCACCCACAAGGCA | 87703439 | 87703461 | 0.23 |
| GTATGAGCTGCCATGCCTTGT | 87703448 | 87703470 | 1.03 |
| GCTATTTGTACCTCCAGACCC | 87703472 | 87703494 | 0.02 |
| GATGGAATCCCCTGGGTCTGG | 87703481 | 87703503 | 1.42 |
| GAGAGTCCATGGAATCCCCT | 87703489 | 87703511 | 0.94 |
| GCCTTGGGGCCAGAGAGTCCA | 87703500 | 87703522 | 0.01 |
| GTGTGTGCTTTGGTGACCTT | 87703516 | 87703538 | 0.91 |
| GTCTGTGTACTGTGTGTGCTT | 87703526 | 87703548 | 0.70 |
| GCACAGTACACAGATACTACC | 87703536 | 87703558 | 0.01 |
| GTTGGATGTGAGTGATTTCCC | 87703554 | 87703576 | 0.01 |
| GTTATATATTTTCATACTTTT | 87703573 | 87703595 | 0.02 |
| GTAAAAGATACTAAGATTTTT | 87703598 | 87703620 | 0.02 |
| GGACGTACATCAAAAGATGA | 87703637 | 87703659 | 0.52 |
| GTTGATGTACGTCCTCTATAA | 87703647 | 87703669 | 1.34 |
| GAAGAGTAGAAACCTTTATAG | 87703658 | 87703680 | 0.03 |
| GGTTTCTACTCTTTCAGAAT | 87703668 | 87703690 | 0.01 |
| GATTTGATTTCAGACCATCTC | 87703693 | 87703715 | 0.01 |
| GACTCATTAATACACCAGAGA | 87703706 | 87703728 | 0.01 |
| GTAATGAGTCCTCTAGTGACT | 87703721 | 87703743 | 1.26 |
| GTCCTCTAGTGACTTGGCTA | 87703727 | 87703749 | 0.66 |
| GAAGACTGAGAAAGGTAGGGT | 87703751 | 87703773 | 0.02 |
| GATGAAAAGACTGAGAAAGGT | 87703756 | 87703778 | 8.57 |
| GACAGACAGACAGAGGAGCA | 87703792 | 87703814 | 0.16 |
| GACAAGCCACTGGATCAGTAA | 87703832 | 87703854 | 5.03 |
| GTTGGCACTAGAACAAGCCAC | 87703843 | 87703865 | 0.36 |
| GCTTGTTCTAGTGCCAAGGT | 87703849 | 87703871 | 0.45 |
| GTCAAACAGTACCACCGACCT | 87703862 | 87703884 | 0.01 |
| GTAACATTGAGATGTCCTATG | 87703897 | 87703919 | 0.17 |
| GCCACTCGCCCCACCCACAT | 87703911 | 87703933 | 0.01 |

| | | | |
|---|---|---|---|
| GCCTCCTGAGCGATGCCCAT | 87703935 | 87703957 | 2.62 |
| GATACAGGACACACATCCCA | 87703951 | 87703973 | 0.28 |
| GACTGAAATAAACGCTGATAC | 87703966 | 87703988 | 0.04 |
| GTTTATTTCAGTCACATTAA | 87703977 | 87703999 | 1.27 |
| GTTTCAGTCACATTAAAGGCC | 87703982 | 87704004 | 0.27 |
| GCTTTAATGTGGGGAATGACC | 87704000 | 87704022 | 0.02 |
| GCATTCCCCACATTAAAGGCC | 87704006 | 87704028 | 1.42 |
| GACACAACAGGGGAAATGACC | 87704024 | 87704046 | 0.03 |
| GCAGGGGGGCTAAACACAAC | 87704037 | 87704059 | 0.01 |
| GATAGGAAAAGAGCTGCAGGG | 87704051 | 87704073 | 1.03 |
| GTAAATGTTTAAAACAAAAAT | 87704069 | 87704091 | 0.02 |
| GATGCTGCACATATACACGG | 87704130 | 87704152 | 0.40 |
| GTATGTGCAGCATCCGGGTGC | 87704140 | 87704162 | 0.01 |
| GTCCGGGTGCAGGGCCAGAGA | 87704151 | 87704173 | 0.42 |
| GATGTCTCTTCTGACCTTCTC | 87704164 | 87704186 | 0.00 |
| GTCCTCTTGAACTATAATGAT | 87704191 | 87704213 | 0.01 |
| GCCACATGGAGGCATCACATG | 87704224 | 87704246 | 0.01 |
| GCTACGCTCACGCCACCCACA | 87704239 | 87704261 | 1.10 |
| GTGAGCGTAGGAACTGAACT | 87704252 | 87704274 | 4.41 |
| GCAGCTAATTGCTCTTGCAG | 87704277 | 87704299 | 0.01 |
| GACGAGACTGGAGATGGAAC | 87704299 | 87704321 | 0.03 |
| GCTGTGACGAGACTGGAGA | 87704305 | 87704326 | 0.00 |
| GTCTGAAAGCTGTGACGAGAC | 87704311 | 87704333 | 0.55 |
| GAGGTGCATTCTCTGATCTG | 87704332 | 87704354 | 0.00 |
| GCTGATCTGAGGAAGGAGAGA | 87704344 | 87704366 | 0.21 |
| GGATACCCCTCCAGATTTG | 87704365 | 87704386 | 0.01 |
| GCGAAACCTCAAATCTGGAG | 87704370 | 87704392 | 0.03 |
| GTGAGGCGAAACCTCAAATC | 87704375 | 87704397 | 0.23 |
| GCATATAGGAAAGAAATGGTG | 87704392 | 87704414 | 2.26 |
| GTTTCCTATATGGTGGGGTTG | 87704404 | 87704426 | 0.07 |
| GTTGAGGGTAGGAACGTGGCC | 87704421 | 87704443 | 0.44 |
| GAAAAGGCAAAGTAGGGACC | 87704439 | 87704461 | 0.01 |
| GTCCCTACTTTGCCTTTTCAC | 87704444 | 87704466 | 0.01 |
| GACTTGCTTCTTCCAGTGAAA | 87704455 | 87704477 | 0.00 |
| GCCAATGCTGTCACGGAAGAT | 87704496 | 87704518 | 6.18 |
| GGGGGACCCAATGCTGTCA | 87704504 | 87704525 | 3.16 |
| GCGGGGGCGGGGCGGGGTGG | 87704521 | 87704543 | 0.01 |
| GAGGCAGGCGGGGGCGGGGC | 87704528 | 87704550 | 0.04 |
| GCAGTTACAGGAGGCAGGCGG | 87704537 | 87704559 | 0.00 |
| GTTATAACAGTTACAGGAGGC | 87704543 | 87704565 | 0.01 |
| GTCAAGACTTATAACAGTTAC | 87704550 | 87704572 | 0.17 |
| GTAAGTCTTGATCCTTATTAC | 87704563 | 87704585 | 1.77 |
| GATGAAGATTATCCAGTAATA | 87704574 | 87704596 | 0.03 |
| GTTAGTATTCTGTTCTGTTGA | 87704615 | 87704637 | 0.04 |
| GCTGTAAACTTAACTGTATTG | 87704668 | 87704690 | 1.74 |
| GAGGCTTTGTCAGAAAACTG | 87704687 | 87704709 | 0.98 |
| GGCAAAATAAGCACACAGGC | 87704719 | 87704741 | 0.02 |
| GTTATGGAAATGATAAAATAG | 87704740 | 87704762 | 0.01 |
| GCTGTTCGGCTGAAAATTTA | 87704757 | 87704779 | 0.00 |
| GTTTACTCTGACACAGCTGTT | 87704771 | 87704793 | 0.40 |
| GGCAAAGCACAATGAGACTT | 87704798 | 87704820 | 0.85 |
| GCAGACAAAAAGGTGGAGGGC | 87704820 | 87704842 | 0.02 |

| | | | |
|---|---|---|---|
| GAAACACAAAACAGACAAAA | 87704831 | 87704853 | 0.02 |
| GTCTGTTTTGTGTTTCAAGAC | 87704839 | 87704861 | 0.00 |
| GACTCTCTCTCTAGAACAGGC | 87704892 | 87704914 | 1.72 |
| GCAGGCGGATCTCTCAGCTCA | 87704915 | 87704937 | 0.03 |
| GTTCGAAGACAGACGCAGG | 87704931 | 87704952 | 1.51 |
| GCGTCTGTCTTCGAACACC | 87704937 | 87704958 | 0.43 |
| GCTGACACACATCTTTAATCC | 87704954 | 87704976 | 0.02 |
| GAAGATGTGTGTCAGTGCGCC | 87704963 | 87704985 | 2.85 |
| GTTTAAAAAGAGCAGTGGCC | 87704981 | 87705003 | 0.11 |
| GTATATGTTTAAAAAGAGCAG | 87704986 | 87705008 | 0.02 |
| GATACCAACTTCACCCTCAGC | 87705006 | 87705028 | 2.26 |
| GCACTCAGCCATGCCTGCTGA | 87705018 | 87705040 | 0.78 |
| GGCATGGCTGAGTGAGTGCG | 87705027 | 87705049 | 0.00 |
| GCTGAGTGAGTGCGTGGTTTC | 87705034 | 87705056 | 1.01 |
| GAAAAGGAGCGGCACACTCTA | 87705063 | 87705085 | 0.87 |
| GTGTGCCGCTCCTTTTTCTT | 87705070 | 87705092 | 0.03 |
| GCCTGTCCCCACCTAAGAAAA | 87705080 | 87705102 | 0.24 |
| GCTTAGGTGGGGACAGGAAAC | 87705087 | 87705109 | 0.37 |
| GTGGGGACAGGAAACTGGGA | 87705092 | 87705114 | 0.01 |
| GGAAACTGGGAAGGAGCTGG | 87705101 | 87705123 | 0.22 |
| GGTGGAATAGCTGCCTGCAC | 87705119 | 87705141 | 0.21 |
| GTACAGTAGCTTTCCCCGTGC | 87705132 | 87705154 | 6.93 |
| GCTACTGTACAACAGCTCA | 87705146 | 87705167 | 0.29 |
| GAGCAACAGAGTAATGGAG | 87705204 | 87705225 | 0.39 |
| GTAAAATGAGCAACAGAGTAA | 87705209 | 87705231 | 2.36 |
| GATTAAGGTCATCAAATGAGC | 87705242 | 87705264 | 2.18 |
| GCATATAAGGAAAGTATTA | 87705258 | 87705279 | 0.27 |
| GGGCACTTCTTTGCATATA | 87705270 | 87705291 | 1.42 |
| GACACAAAAGCTGTTTTGTCA | 87705289 | 87705311 | 0.02 |
| GCTTTTGTGTTCTTATTGTTT | 87705303 | 87705325 | 0.01 |
| GTTATTGTTTTGGTAAAGCTG | 87705314 | 87705336 | 0.01 |
| GTAGATCTTTTTAAAGATAAA | 87705341 | 87705363 | 0.01 |
| GCTTTTAATATTTTATATAGT | 87705377 | 87705399 | 0.05 |
| GATCAAATTACAGAAAGCTAG | 87705433 | 87705455 | 0.05 |
| GTCTCCTTGTGAATTCACTTT | 87705464 | 87705486 | 0.20 |
| GAAAATGTGCTCTGATTAAG | 87705492 | 87705514 | 0.68 |
| GATTAAGTGGATAATAATT | 87705505 | 87705526 | 3.33 |
| GAAAAACATATTCACTAAATA | 87705542 | 87705564 | 0.07 |
| GATTTAATAACGTTCAATGCC | 87705623 | 87705645 | 0.02 |
| GAAAAGAAGTCTATCTGTACC | 87705641 | 87705663 | 0.01 |
| GTTAAACAAATAAAATTACAA | 87705673 | 87705695 | 0.02 |
| GAAAAAAAAAGAACAATCACT | 87705722 | 87705744 | 0.03 |
| GCATTTAATTTTCTGTTTCCA | 87705761 | 87705783 | 0.01 |
| GTCAAATTAAGGAGCAAACCA | 87705778 | 87705800 | 0.02 |
| GATAGAACTGGTTTCAAATTA | 87705790 | 87705812 | 0.25 |
| GACCAGTTCTATTTAGTGTTC | 87705802 | 87705824 | 0.02 |
| GTCAGGTAAATTGCATTTTAA | 87705820 | 87705842 | 0.01 |
| GTTAAAAGCAAAGACATACAT | 87705869 | 87705891 | 0.01 |
| GTCATTTTTCATTACACTTTC | 87705901 | 87705923 | 0.00 |
| GGCTGGTTAATAATATAGC | 87705922 | 87705943 | 0.47 |
| GCTGGTAAATAATAAAAAGTC | 87705940 | 87705962 | 0.03 |
| GAAATAATAAAAAGTCAGGTG | 87705945 | 87705967 | 0.34 |

| | | | |
|---|---|---|---|
| GTACTTGGAGTAACGGTGTG | 87705970 | 87705992 | 0.02 |
| GCACCGTTACTCCAAGTACTC | 87705975 | 87705997 | 4.58 |
| GCCTCTGCCCCCGGAGTACT | 87705985 | 87706007 | 0.03 |
| GATCTCCTTGCCTCTGCCCC | 87705994 | 87706016 | 0.01 |
| GCTGCAGACTGGCACTGAACT | 87706018 | 87706040 | 0.51 |
| GACAGGTCTAGCTGCAGAC | 87706030 | 87706051 | 1.94 |
| GTAGGCTATTGCTTTGGAGAC | 87706046 | 87706068 | 0.60 |
| GCTGCTCCTAGGCTATTGCTT | 87706053 | 87706075 | 0.48 |
| GTCTTTGTAAACTGCTCCT | 87706065 | 87706086 | 0.23 |
| GACAGTGTTTAAGAGTGCCA | 87706084 | 87706106 | 0.76 |
| GTTAAGAGTGCCATGGCAAGC | 87706092 | 87706114 | 1.30 |
| GTGCCATGGCAAGCCGGGTG | 87706098 | 87706120 | 0.62 |
| GAAAAAAAAAAAAGAGTGCCA | 87706256 | 87706278 | 2.14 |
| GCTGGGATCAAATCCAGCCA | 87706273 | 87706295 | 0.75 |
| GTTTAGAGGGCAGTGAATGCT | 87706290 | 87706312 | 1.09 |
| GTTTGATGGACGCATTTTAGA | 87706304 | 87706326 | 0.18 |
| GCTCAAAATGTCAGGATTTGA | 87706319 | 87706341 | 1.58 |
| GTACACCCACCTCAAAATGTC | 87706328 | 87706350 | 0.03 |
| GCTCTCGAGGGCTGGAATTAA | 87706361 | 87706383 | 0.01 |
| GTTCCAGCCCTCGAGAGGCAG | 87706368 | 87706390 | 0.15 |
| GTCTTTGAGTCAAAACCAACA | 87706401 | 87706423 | 18.10 |
| GCAGAGTGAGCTCTGCCATGT | 87706415 | 87706437 | 4.92 |
| GCTGGCTTTGAATTCACAGAG | 87706447 | 87706469 | 1.25 |
| GCTTAAACACTTAGAAGGCAG | 87706478 | 87706500 | 0.51 |
| GCTAACACTTAAACACTTAGA | 87706484 | 87706506 | 0.40 |
| GTGTTTAAGTGTTAGGATTA | 87706492 | 87706514 | 0.30 |
| GGACAAATGCCACCACACC | 87706513 | 87706534 | 0.73 |
| GCACCACACCTGGCAGTCACA | 87706523 | 87706545 | 4.34 |
| GTCTAAACCCGTGTGACTGCC | 87706530 | 87706552 | 1.97 |
| GCACTCACACGCTCGTGCAC | 87706568 | 87706590 | 1.32 |
| GAGCGTGTGAGTGCGTGGC | 87706577 | 87706598 | 0.05 |
| GATCCCACCCTCCCTTATCTG | 87706601 | 87706623 | 1.01 |
| GCTGTCTCCACAGATAAGGGA | 87706607 | 87706629 | 3.23 |
| GTCCTCTGTCTCCACAGATA | 87706612 | 87706634 | 0.01 |
| GCAGGGTGAAAAATGACTTGC | 87706639 | 87706661 | 6.23 |
| GATTTTTCACCCTGTACCACA | 87706649 | 87706671 | 1.18 |
| GCCTGGAGCCCATGTGGTACA | 87706657 | 87706679 | 0.89 |
| GTACCACATGGGCTCCAGGGA | 87706662 | 87706684 | 0.14 |
| GGCGACCTGAGCTCCATCCC | 87706675 | 87706697 | 0.03 |
| GGAGCTCAGGTCGCCAGGCG | 87706683 | 87706705 | 0.06 |
| GAACTTAAAACTGCCACGCC | 87706696 | 87706718 | 0.53 |
| GTGCAGGCAAGGTGGCTCAT | 87706724 | 87706746 | 0.17 |
| GTCTAGCTAGTGCAGGCAAGG | 87706732 | 87706754 | 3.42 |
| GAATCTCTATCTAGCTAGTGC | 87706740 | 87706762 | 6.27 |
| GTAGATAGAGATTTTTAAGAA | 87706751 | 87706773 | 0.23 |
| GATTTTTAAGAATGGTTTTT | 87706759 | 87706781 | 0.01 |
| GTAAGAATGGTTTTTTGGAGG | 87706765 | 87706787 | 0.01 |
| GGTTTTTTGGAGGAGGCAT | 87706772 | 87706793 | 0.49 |
| GTTTGGAGGAGGCATTGGAAG | 87706777 | 87706799 | 0.46 |
| GTGGAGTGCTGTGCTGAAT | 87706796 | 87706817 | 0.93 |
| GTGCTGAATTGGAGCTATTG | 87706806 | 87706828 | 1.19 |
| GGAGCTATTGAGGTTCTTA | 87706816 | 87706837 | 3.84 |

| | | | |
|---|---|---|---|
| GCTTAAGGTAGATCTTTGGTT | 87706831 | 87706853 | 1.04 |
| GACATTCTTTAGTAATACTTA | 87706889 | 87706911 | 0.45 |
| GTACTTAAGGAAAATGCAAAT | 87706903 | 87706925 | 0.01 |
| GAAATGCAAATAGGGTAGTGT | 87706913 | 87706935 | 2.89 |
| GTTGGAGACAGCTGCATCA | 87706931 | 87706952 | 3.05 |
| GACAGCTGCATCATGGACTT | 87706937 | 87706959 | 3.54 |
| GACTTTGGGTCTGCTTCCTG | 87706952 | 87706974 | 8.14 |
| GATACACAGGAATTACCCAC | 87706968 | 87706990 | 1.04 |
| GCTTAAGAGTTCTAGATACAC | 87706981 | 87707003 | 2.54 |
| GACTTATTAACAAATTTTAAT | 87707006 | 87707028 | 0.02 |
| GTTAATAAGTAGAAAGTTCT | 87707019 | 87707041 | 0.04 |
| GTAGAAAGTTCTTGGTAGTGT | 87707028 | 87707050 | 0.58 |
| GTAGTGTTGGCTGGACATGT | 87707041 | 87707063 | 0.28 |
| GCATATCTGGTGCGCGCGGC | 87707074 | 87707096 | 3.91 |
| GTTCTTCTGCCTGTGCATATC | 87707087 | 87707109 | 3.41 |
| GAAGTCATACAAACAGATGTG | 87707134 | 87707156 | 1.82 |
| GTCCTTGCTCCTGCGATAGG | 87707162 | 87707184 | 11.75 |
| GTGGTTTTCCACCTATCGC | 87707171 | 87707192 | 4.97 |
| GAAGGTTTTACAAGGAACTCG | 87707189 | 87707211 | 3.31 |
| GATCAAATGAAGGTTTTACA | 87707198 | 87707220 | 4.65 |
| GCTCAGGTTAGGATCAAATGA | 87707208 | 87707230 | 24.33 |
| GTCTCTTAGTTCACTCAGGTT | 87707220 | 87707242 | 9.98 |
| GAGTGAACTAAGAGAAGTCA | 87707228 | 87707250 | 4.26 |
| GAAGAGAAGTCATGGATGGCC | 87707237 | 87707259 | 2.20 |
| GGACTGCATCTTCTTCTCC | 87707255 | 87707276 | 11.17 |
| GCCGGGCTGCATTTATTAAGG | 87707275 | 87707297 | 2.95 |
| GATAAATGCAGCCCGGGGGCT | 87707283 | 87707305 | 0.01 |
| GCCCGGGGGCTCGGTAAGAT | 87707292 | 87707314 | 15.38 |
| GGGGCTCGGTAAGATGGGTT | 87707297 | 87707319 | 26.36 |
| GTAAGATGGGTTTGGAGGGGC | 87707306 | 87707328 | 1.47 |
| GTTGGAGGGGCAGGGTCATTC | 87707316 | 87707338 | 0.01 |
| GGGGCAGGGTCATTCTGGCC | 87707321 | 87707343 | 0.01 |
| GAAAGGATTCGGGGACACC | 87707339 | 87707360 | 3.83 |
| GTGAAAAGAGAAAGGATTCG | 87707347 | 87707369 | 2.42 |
| GCTCTCAGGGTGAAAAGAGAA | 87707355 | 87707377 | 0.05 |
| GTGAAGCACGTGCCCTCTCA | 87707369 | 87707391 | 2.58 |
| GCACTCAGAGACTAGTCATTG | 87707389 | 87707411 | 2.92 |
| GAAAATCACAAACTGTTCACA | 87707435 | 87707457 | 5.55 |
| GCTATAATTTTTATTTATGCG | 87707467 | 87707489 | 0.03 |
| GCTGTTCTCTTTACAAATG | 87707496 | 87707517 | 0.02 |
| GATAGTTTTTATTGATAAATT | 87707525 | 87707547 | 0.12 |
| GAAATCTAGATACAAATTTGT | 87707562 | 87707584 | 0.01 |
| GTATCTAGATTTTTAAGTG | 87707573 | 87707594 | 0.01 |
| GTTTTACTCAAAGTAATTATT | 87707595 | 87707617 | 0.05 |
| GTTGAGTAAAATTGTAAGTCG | 87707608 | 87707630 | 0.02 |
| GATCTGTAAAATTTTCTATCA | 87707631 | 87707653 | 0.02 |
| GTAGAAAATTTTACAGATCTT | 87707637 | 87707659 | 3.06 |
| GTTCACAGAACAAATAATGAC | 87707678 | 87707700 | 1.77 |
| GAAGGAGACACTAGAAAAACT | 87707727 | 87707749 | 1.84 |
| GTCAGAGTCAGATTTTTGTT | 87707750 | 87707772 | 0.01 |
| GACTAGGGTATAATGAAAAGT | 87707785 | 87707807 | 0.17 |
| GCAAAGAATAGCTGAAAACTA | 87707801 | 87707823 | 1.57 |

| | | | |
|---|---|---|---|
| GTTTGGTTAAAAAGAGAAGCC | 87707820 | 87707842 | 0.91 |
| GTTAAAAAGAGAAGCCTGGCC | 87707825 | 87707847 | 0.01 |
| GCTAAGCCATCACCCCAGGCC | 87707838 | 87707860 | 0.60 |
| GATCTACTAAGCCATCACCCC | 87707843 | 87707865 | 2.16 |
| GTTAGGTAGTTAGGCTGTT | 87707876 | 87707897 | 1.27 |
| GCAGGTTCATGTTAGGTAGTT | 87707884 | 87707906 | 0.02 |
| GCTACCTAACATGAACCTGCT | 87707889 | 87707911 | 3.09 |
| GCAGGTCTCTTTCCACCAAGC | 87707903 | 87707925 | 1.73 |
| GAAGACAAATTATGGGAGTC | 87707922 | 87707944 | 0.53 |
| GAGCTCAGAAGACAAATTAT | 87707929 | 87707951 | 0.46 |
| GCTGAGCTCTACATTGAGCCA | 87707944 | 87707966 | 3.10 |
| GTGTGTGGAAATGTGTGCCA | 87707961 | 87707983 | 9.05 |
| GTCACTTTATCTATTTGTATG | 87707985 | 87708007 | 0.01 |
| GTATGCAGAACTACTTAAGCA | 87708030 | 87708052 | 0.98 |
| GAGGTCTATGAAAATGGGTA | 87708059 | 87708081 | 0.42 |
| GATGTTGGAGGTCTATGAAAA | 87708065 | 87708087 | 0.53 |
| GCACATGCTGTTTCAGATGT | 87708081 | 87708103 | 2.48 |
| GGTGGGGAGCACTGTTGGTG | 87708105 | 87708127 | 1.78 |
| GCCACTGGTGGGGAGCACTGT | 87708110 | 87708132 | 3.47 |
| GGTGGGCAGCACCACTGGTG | 87708121 | 87708143 | 1.41 |
| GCTATTGGTGGGCAGCACCAC | 87708126 | 87708148 | 4.57 |
| GATGGCAGAGCACTATTGGT | 87708138 | 87708160 | 1.56 |
| GTGCTCTGCCATCTTGGGT | 87708148 | 87708169 | 1.64 |
| GATGTGTAAACCTACCCAAGA | 87708156 | 87708178 | 0.56 |
| GTAAAAGCTGGCTTCTGACAT | 87708195 | 87708217 | 1.35 |
| GTTAAGTTCTCTTATAAAAGC | 87708208 | 87708230 | 0.02 |
| GAACTATGTGTAATATATCTA | 87708236 | 87708258 | 0.89 |
| GCACATAGTTTCCCAATGAAA | 87708250 | 87708272 | 0.03 |
| GGTTCAAATCCCATTTCATT | 87708260 | 87708282 | 0.08 |
| GTATGATTGGCTCATTGGAAA | 87708281 | 87708303 | 0.01 |
| GAAATTATGATTGGCTCAT | 87708287 | 87708308 | 0.05 |
| GTAATTACTAGAAATTATGAT | 87708295 | 87708317 | 0.02 |
| GTATGTTGCACTAATCAGATG | 87708358 | 87708380 | 0.03 |
| GCAGCCACTTTTTGGTCTAAA | 87708387 | 87708409 | 5.81 |
| GTAAATTATACAGCCACTTTT | 87708396 | 87708418 | 0.01 |
| GTTTTTTCTTTCTAATCGTAA | 87708429 | 87708451 | 0.00 |
| GTCTTTCTAATCGTAAAGGAT | 87708434 | 87708456 | 4.24 |
| GAATCGTAAAGGATTGGATCC | 87708441 | 87708463 | 12.22 |
| GTCCTGGCAAACAGATTAAAT | 87708458 | 87708480 | 14.51 |
| GTTGGACTCCAGTGCACAGTT | 87708477 | 87708499 | 13.64 |
| GATAATATCCAAACTGTGCAC | 87708484 | 87708506 | 13.68 |
| GTTATTTCCGTGTAACCTGCA | 87708503 | 87708525 | 6.28 |
| GTCTCTTCCTTGCAGGTTACA | 87708509 | 87708531 | 4.10 |
| GAAGCACTTTCTCTTCCTTGC | 87708517 | 87708539 | 5.54 |
| GCAACAAGAACTTCAGCACAG | 87708545 | 87708567 | 15.52 |
| GACAGTGGACATCCAGAAGAA | 87708561 | 87708583 | 8.93 |
| GTAAACTTCACGCCATTCTTC | 87708572 | 87708594 | 3.98 |
| GATGGCGTGAAGTTTACCAAC | 87708580 | 87708602 | 13.54 |
| GAAGCAGGCTCTCTGACCTGT | 87708595 | 87708617 | 4.73 |
| GCTTACGAGTGTGACAAGC | 87708611 | 87708632 | 5.34 |
| GTTGTCACACTCGTAAGCTTG | 87708616 | 87708638 | 3.20 |
| GTAAGCTTGTGGTTCTTCA | 87708627 | 87708648 | 1.75 |

| | | | |
|---|---|---|---|
| GTGGTTCTTCATGGCTTCTC | 87708635 | 87708657 | 3.23 |
| GTTAAAGACTTTGCTTTTAC | 87708654 | 87708676 | 0.33 |
| GAAAAATATTAACTAAGAGTC | 87708693 | 87708715 | 0.03 |
| GTAAAGTATCTACTAGCTTCT | 87708728 | 87708750 | 0.02 |
| GTACTTTAGCACTGTTAGCCC | 87708744 | 87708766 | 2.26 |
| GCTTTTAGGACGACCCCTCCT | 87708761 | 87708783 | 9.28 |
| GGGTCGTCCTAAAAGTCCCT | 87708769 | 87708791 | 2.85 |
| GTCCTAAAAGTCCCTTGGGA | 87708774 | 87708796 | 4.25 |
| GGGGGTACTGCCCCTCCCAA | 87708785 | 87708807 | 0.64 |
| GTTCTAGAACAGTACAGGAG | 87708805 | 87708827 | 2.04 |
| GCACAGGTTCTAGAACAGTAC | 87708810 | 87708832 | 0.06 |
| GTAATAGCAAGGCAGGCCAC | 87708827 | 87708849 | 0.91 |
| GACTAAGGAGTAGTAATAGCA | 87708838 | 87708860 | 1.01 |
| GTTTCTGTACTGGGAATACTA | 87708854 | 87708876 | 0.40 |
| GATCTATATGTTTTCTGTACT | 87708864 | 87708886 | 0.02 |
| GATAACGTAAGCATACAAGAG | 87708892 | 87708914 | 0.44 |
| GCTTACGTTATTTGAGTGTAC | 87708905 | 87708927 | 1.59 |
| GCATACACAAAGACAAAACAC | 87708924 | 87708946 | 2.22 |
| GATGCTCTTCTGGCTTTTAAC | 87708966 | 87708988 | 1.05 |
| GCAGGTCTGACATGCTCTTC | 87708977 | 87708999 | 4.81 |
| GCATGTCAGACCTGCTGGGGC | 87708986 | 87709008 | 1.75 |
| GCCATTAACTCCAGCCCCAGC | 87708995 | 87709017 | 0.02 |
| GCTGGAGTTAATGGACAATTG | 87709005 | 87709027 | 2.22 |
| GACAATTGTGGGACACCATA | 87709017 | 87709039 | 0.43 |
| GGACACCATATGGGTGACCC | 87709027 | 87709049 | 0.23 |
| GAAGACCCAGGGTCACCCATA | 87709032 | 87709054 | 0.41 |
| GCTGCTCTAGAAGAAGACCC | 87709045 | 87709067 | 0.08 |
| GTACTTAATAAGTTAATACA | 87709094 | 87709116 | 1.89 |
| GTTGCATGCTGACATTATTCT | 87709123 | 87709145 | 6.96 |
| GTTACACCATTTTCATAACTG | 87709157 | 87709179 | 0.03 |
| GCAGTTATGAAAATGGTGTAA | 87709160 | 87709182 | 10.18 |
| GTCTTTCAAAAAATTATTATA | 87709185 | 87709207 | 0.02 |
| GTTTTTTGAAAGAAGTACGAC | 87709196 | 87709218 | 0.02 |
| GAGATTTATCAGATCTGTGA | 87709233 | 87709255 | 1.29 |
| GTTACTAACTAGCATGTAAGC | 87709260 | 87709282 | 1.04 |
| GAAAGAGGGGAAAAAAGAGCT | 87709301 | 87709323 | 1.09 |
| GAATTAAACTGTTTAAAAGAG | 87709315 | 87709337 | 0.02 |
| GTTTGAGGGGGGGACCAGGAA | 87709388 | 87709410 | 0.38 |
| GATTTTTTTGAGGGGGGGACC | 87709393 | 87709415 | 0.02 |
| GATAGGTTTATTTTTTTGAG | 87709402 | 87709424 | 0.00 |
| GGGGGAAGAGAGGATGGGAT | 87709419 | 87709441 | 0.03 |
| GAAGCAGGGGGAAGAGAGGA | 87709425 | 87709447 | 0.02 |
| GAACTTTCTTGGTCCCATGA | 87709526 | 87709548 | 1.12 |
| GCTCTAGCTAGAACTTTCT | 87709537 | 87709558 | 0.01 |
| GACAAAAGAAAGTCAAAGGAA | 87709568 | 87709590 | 1.96 |
| GTCAAAGGAATGGATACAAAT | 87709579 | 87709601 | 4.24 |
| GCTTGGCTGAGTCACACCA | 87709835 | 87709856 | 4.04 |
| GTAATATAGTAAAAATAAAAA | 87709960 | 87709982 | 0.09 |
| GAATAAAAATGGCCATCTTGC | 87709972 | 87709994 | 0.51 |
| GGGATAGTGATTCTGCCAG | 87710133 | 87710154 | 1.19 |
| GCAATTGAGAACAAAACTGCA | 87710178 | 87710200 | 1.18 |
| GAACAAAACTGCATGGTGC | 87710185 | 87710206 | 0.91 |

| | | | |
|---|---|---|---|
| GCATGGTGCTGGTACAGAGAC | 87710196 | 87710218 | 0.05 |
| GTAAAAATGCTTTTTTCCCAC | 87710304 | 87710326 | 0.41 |
| GTTCAGGAATTCATGTGTTCG | 87710481 | 87710503 | 0.01 |
| GCAGAACTGTCAATACTGTA | 87710580 | 87710602 | 0.06 |
| GACTGTAAGGACACTGTCAAT | 87710594 | 87710616 | 0.17 |
| GTTGTATATCGGATATAGGAT | 87710654 | 87710676 | 0.03 |
| GTTCTTTGTATATCGGATAT | 87710659 | 87710681 | 0.01 |
| GCTAAACAGTTTTCAACTG | 87710738 | 87710759 | 0.02 |
| GCTCCTAACTATTATGAATA | 87711105 | 87711127 | 3.46 |
| GTATTCATAATAGTTAGGAGC | 87711108 | 87711130 | 0.04 |
| GTCTTTTGCCTAAGAATTTCA | 87711225 | 87711247 | 0.01 |
| GGCAAAAGAATAGAACTAGA | 87711239 | 87711261 | 0.56 |
| GGGGAAAAGCACCCACACTT | 87711399 | 87711421 | 0.01 |
| GGGTGCTTTTCCCCTTAGA | 87711408 | 87711429 | 2.18 |
| GATTTTGTTCTCCTTCTAAG | 87711418 | 87711440 | 2.37 |
| GTAATGTAGAGCAGAGACTGA | 87711462 | 87711484 | 0.07 |
| GTTCAGAGATTGTCCCACCTG | 87711493 | 87711515 | 0.11 |
| GTTGGTAACTATATATGGGA | 87711518 | 87711540 | 0.07 |
| GCTGAAAGGAGCTTGATAA | 87711573 | 87711594 | 0.35 |
| GCTCCCAGGGACTAAGCCAA | 87711772 | 87711794 | 1.09 |
| GTAGCACAGGATGGGCTTGTC | 87711821 | 87711843 | 21.40 |
| GTATGAGTTATATTAAGAGCT | 87711855 | 87711877 | 4.39 |
| GATGGGAGTGATATAATAAAT | 87711887 | 87711909 | 0.02 |
| GTAAGAGGTATTTTATTGAAT | 87711905 | 87711927 | 0.05 |
| GCTGTCTTTATGTAGCCTAAG | 87711921 | 87711943 | 0.28 |
| GCTACATAAAGACAGAACCTA | 87711930 | 87711952 | 2.58 |
| GACAGAACCTATGGGGTATTT | 87711940 | 87711962 | 1.12 |
| GCTTTTACCTAAATACCCCAT | 87711946 | 87711968 | 0.38 |
| GGTATTTAGGTAAAAGAAAT | 87711953 | 87711975 | 0.09 |
| GTAAAAGAAATTGGAATGAT | 87711962 | 87711984 | 1.55 |
| GAATTGGAATGATTGGCCACA | 87711970 | 87711992 | 0.30 |
| GTCTTCGCTTTTAGCACCATG | 87711985 | 87712007 | 0.03 |
| GAATCCATTACCTTCAAGTTG | 87712007 | 87712029 | 0.01 |
| GCCATCTTCTCCACAACTTGA | 87712016 | 87712038 | 2.76 |
| GAATAAGTAAATCTTTAAAAG | 87712079 | 87712101 | 0.01 |
| GCATCAGGTCACACTGTAGA | 87712152 | 87712174 | 0.36 |
| GTGGTTGCTGGGGATTGACT | 87712189 | 87712211 | 0.04 |
| GGGGATTGACTTGGAAGAAC | 87712198 | 87712220 | 3.72 |
| GACTCATTTTTCTTCTTAAA | 87712252 | 87712274 | 0.02 |
| GCTGAATAATGGATACACACT | 87712275 | 87712297 | 7.21 |
| GTAAGGTTTTATTCTGAATAA | 87712287 | 87712309 | 2.94 |
| GCAGAATAAAACCTTAGACTA | 87712295 | 87712317 | 0.02 |
| GTCATATGGGCCATAGTCTA | 87712305 | 87712327 | 4.40 |
| GGGCTGGTGCCTGGTCATAT | 87712318 | 87712340 | 1.07 |
| GAAGAGATGGGCTGGTGCC | 87712327 | 87712348 | 0.01 |
| GTCCTGCTGAAGAGATGGGC | 87712334 | 87712356 | 0.02 |
| GCAACACAAAAAGATTACTA | 87712411 | 87712433 | 0.02 |
| GTAATCTTTTTGTGTTGCTTT | 87712417 | 87712439 | 0.01 |
| GTTTGTGTTGCTTTAGGCCCA | 87712424 | 87712446 | 1.89 |
| GTTGCTTTAGGCCCATGGCC | 87712429 | 87712451 | 8.09 |
| GTAGGCCCATGGCCAGGAGCC | 87712436 | 87712458 | 0.01 |
| GATGGCCCTGGCTCCTGGCCA | 87712441 | 87712463 | 0.01 |

| | | | |
|---|---|---|---|
| GAAAGGGATGGCCCTGGCTCC | 87712447 | 87712469 | 15.96 |
| GTCAAAGAAAAGGGATGGCCC | 87712454 | 87712476 | 1.08 |
| GCTGACATCAAAGAAAAGGGA | 87712460 | 87712482 | 2.63 |
| GAAGTGCTGACATCAAAGAAA | 87712465 | 87712487 | 1.84 |
| GGTGCTTTAGAGGATTGAA | 87712500 | 87712521 | 9.74 |
| GTAAAATGAAAGGTGCTTTAG | 87712509 | 87712531 | 1.90 |
| GAAATTACAGGTTAAAATGAA | 87712520 | 87712542 | 5.20 |
| GATCTGAACAATGAAAATTAC | 87712533 | 87712555 | 0.04 |
| GCTTCCCTCGTAAGACGAATC | 87712557 | 87712579 | 1.37 |
| GACGAATCTGGATATAAATGC | 87712570 | 87712592 | 4.20 |
| GTATATTCTTCATTTAAAG | 87712636 | 87712657 | 6.54 |
| GAAGAATATACTAAGAACAA | 87712647 | 87712669 | 19.32 |
| GAACAAAGGCGAGTATGAAG | 87712661 | 87712683 | 8.36 |
| GGCGAGTATGAAGAGGCCC | 87712668 | 87712689 | 3.58 |
| GTTCTTTAACAATGGCATCCT | 87712684 | 87712706 | 5.92 |
| GATTGACAATTTCTTTAACAA | 87712693 | 87712715 | 1.51 |
| GAAATTGTCAATATTTCCTC | 87712704 | 87712726 | 6.30 |
| GCTCTATTAAGTTTACCTG | 87712720 | 87712741 | 10.39 |
| GAAACTTAATAGAGCATAAAA | 87712728 | 87712750 | 7.16 |
| GATATCCTAGCTTTTTCTAA | 87712756 | 87712778 | 0.03 |
| GACATCCCATTAGAAAAAGCT | 87712761 | 87712783 | 3.41 |
| GATCCACAGAAGTAGAAAAA | 87712918 | 87712940 | 3.34 |
| GTTTTCTACTTCTGTGGATCC | 87712922 | 87712944 | 1.14 |
| GAGTACTTTAGTTAAATCCC | 87712940 | 87712962 | 1.56 |
| GATTTAACTAAAGTACTCAGG | 87712946 | 87712968 | 8.31 |
| GGCTGGGCAGATAAATATGC | 87712969 | 87712991 | 3.26 |
| GTATCTGCCCAGCCGTCTCAC | 87712979 | 87713001 | 4.49 |
| GTAAGAGCCAGTGAGACGGC | 87712986 | 87713008 | 0.01 |
| GTCTTATCTTTTTTTCTTTTA | 87713017 | 87713039 | 0.02 |
| GTTTTTCTTTTAAGGTAGGTA | 87713026 | 87713048 | 0.01 |
| GATCAGCAACAACACAGGAAT | 87713073 | 87713095 | 0.13 |
| GAATCAACAACAACACAACAC | 87714202 | 87714224 | 1.23 |
| GTAAGGAATCAGCAACAACAC | 87714243 | 87714265 | 1.59 |
| GCAAGAGAACACTTCATCTA | 87714261 | 87714283 | 0.29 |
| GTTCTCACGTGCTGAGTTTGA | 87714290 | 87714312 | 0.28 |
| GTTAAAACAAACACTGTAGAG | 87714319 | 87714341 | 0.01 |
| GTTTGTTTTAAATCATTATC | 87714331 | 87714353 | 0.01 |
| GGGTTCACACTGAAGAGTA | 87714352 | 87714373 | 9.38 |
| GCACACAAGACAAGCTGCTGT | 87714405 | 87714427 | 0.01 |
| GATAAATTTTCAGTCTATTCT | 87714446 | 87714468 | 0.08 |
| GTCTATTCTTGGCACAGCAC | 87714457 | 87714479 | 0.36 |
| GCTATAATGGACACCCTCTTC | 87714553 | 87714575 | 0.57 |
| GCCTCCTGGGGGCTGGAGAGA | 87714656 | 87714678 | 0.11 |
| GCACAACACCTCCTGGGGGC | 87714664 | 87714686 | 0.33 |
| GATACAAAGCACAACACCTCC | 87714671 | 87714693 | 3.44 |
| GTGTTGTGCTTTGTATTTAA | 87714678 | 87714700 | 0.00 |
| GATTTAAAGGGCCGTGTGACT | 87714692 | 87714714 | 0.01 |
| GCAAGTAACATCCAAGTCACA | 87714702 | 87714724 | 0.31 |
| GATGTTACTTGCCTATAGAA | 87714714 | 87714736 | 0.01 |
| GCATATATTCACCCATTCTAT | 87714725 | 87714747 | 0.68 |
| GGTGAATATATGTTTAGTGC | 87714736 | 87714758 | 0.11 |
| GATATATGTTTAGTGCAGGGT | 87714741 | 87714763 | 0.01 |

| | | | |
|---|---|---|---|
| GTTTAGTGCAGGGTAGGTAA | 87714747 | 87714769 | 0.01 |
| GGGTAGGTAATGGTGTGTT | 87714757 | 87714778 | 0.14 |
| GGTAATGGTGTGTTAGGCTG | 87714762 | 87714784 | 0.62 |
| GGTTTAGATATGAACTTAGA | 87714784 | 87714806 | 0.01 |
| GAACTTAGATGGGACCTACAG | 87714796 | 87714818 | 0.01 |
| GGACCTACAGTGGTAACTGT | 87714806 | 87714828 | 0.35 |
| GTGGTAACTGTAGGACAGA | 87714815 | 87714836 | 0.84 |
| GGACAGATGGCATGAGCTG | 87714827 | 87714848 | 1.63 |
| GAAGAGCCACCATGCCACTG | 87714856 | 87714878 | 2.78 |
| GAACACTCCACAGTGGCATGG | 87714862 | 87714884 | 1.05 |
| GGGGCAAGAACACTCCACAG | 87714870 | 87714892 | 0.32 |
| GTCAAAGCAGGTTAAGGTCTG | 87714889 | 87714911 | 1.11 |
| GTTCTGTTTCAAAGCAGGTTA | 87714896 | 87714918 | 0.02 |
| GATGCGTTTCTGTTTCAAAGC | 87714902 | 87714924 | 1.28 |
| GCAGAAACGCATCAGATAAAA | 87714914 | 87714936 | 0.07 |
| GCATCAGATAAAATGGATCG | 87714921 | 87714943 | 0.65 |
| GGGCATTCTTTTTCATGCTT | 87714942 | 87714964 | 0.01 |
| GATTGCTGAAGAAGCATGAGA | 87714977 | 87714999 | 0.02 |
| GAAGAAGCATGAGAAGGAC | 87714983 | 87715004 | 0.04 |
| GAGAAGGACAGGCTCTGCTC | 87714993 | 87715015 | 0.02 |
| GACAGGCTCTGCTCAGGATTA | 87715000 | 87715022 | 0.01 |
| GCAGGATTAAGGGAGACTCAT | 87715012 | 87715034 | 0.49 |
| GGGCAAAACAGCCAAGCCAA | 87715033 | 87715055 | 0.39 |
| GCAGTGGCTGGGCCTTTGGCT | 87715044 | 87715066 | 0.89 |
| GTCAGTCAGTGGCTGGGCCTT | 87715049 | 87715071 | 0.11 |
| GCCTGGAGTCAGTCAGTGGCT | 87715056 | 87715078 | 0.10 |
| GCTGGCCCTGGAGTCAGTCAG | 87715061 | 87715083 | 0.01 |
| GCCGAGCCACACCCCTGGCCC | 87715074 | 87715096 | 1.02 |
| GCCAGGGGTGTGGCTCGGGA | 87715079 | 87715101 | 0.01 |
| GTGCTGTGACACCACCATCT | 87715103 | 87715125 | 0.38 |
| GACCACCATCTTGGTGTAGGA | 87715113 | 87715135 | 3.09 |
| GCTGCCCTTCCTACACCAAGA | 87715117 | 87715139 | 0.02 |
| GTGTAGGAAGGGCAGGAAGG | 87715125 | 87715147 | 0.01 |
| GGAAGGTGGCCTTCCTCTAA | 87715139 | 87715161 | 0.44 |
| GGCTCACAGCCCTTAGAGGA | 87715148 | 87715170 | 0.36 |
| GGCTGTGAGCCAAATTATTT | 87715160 | 87715182 | 0.35 |
| GTTCATCACGCCTAAATAATT | 87715169 | 87715191 | 0.01 |
| GGCGTGATGAATCTTTGAAA | 87715181 | 87715203 | 0.01 |
| GTCAGAAGAAACAGAACGTA | 87715229 | 87715251 | 0.02 |
| GATGTGCTGTCCGTACTGATG | 87715262 | 87715284 | 1.62 |
| GTCCGTACTGATGCGGGTTGA | 87715270 | 87715292 | 0.00 |
| GATGATGTGTGAAGCCTGCA | 87715293 | 87715315 | 0.17 |
| GTGAAGCCTGCATGGTTGC | 87715301 | 87715322 | 0.01 |
| GTCAGTTACCTGCAACCATGC | 87715307 | 87715329 | 0.13 |
| GCTGAAGCGCAGTAATTGAGT | 87715326 | 87715348 | 0.01 |
| GTTTCTCACTGTGCTAGTTG | 87715454 | 87715476 | 0.19 |
| GCACTGTGCTAGTTGAGGCTC | 87715460 | 87715482 | 0.01 |
| GATATAAAGCTCTTGACTCAA | 87715490 | 87715512 | 0.14 |
| GAGGGACAGAGGAGAAAAGA | 87715561 | 87715583 | 1.38 |
| GAGCCCATGGAGAGGGACAG | 87715572 | 87715594 | 0.03 |
| GAAAATCTGAGCCCATGGAGA | 87715579 | 87715601 | 0.06 |
| GATGGGCTCAGATTTTGGCCC | 87715587 | 87715609 | 0.46 |

| | | | |
|---|---|---|---|
| GTTCAACTTTGAGATTAGCCT | 87715604 | 87715626 | 1.28 |
| GATATGCTTTAAAAAAATGAT | 87715631 | 87715653 | 0.02 |
| GGGCTTATGAAATGACTTAG | 87715652 | 87715674 | 0.02 |
| GAATGACTTAGTGGGTAAAGG | 87715662 | 87715684 | 1.29 |
| GGGTAAAGGTGGTTGCTTT | 87715673 | 87715694 | 2.94 |
| GCTTTAGGCTGTGTGACCTG | 87715687 | 87715709 | 1.20 |
| GGGCCTGGAGATCAAACCCC | 87715703 | 87715725 | 0.01 |
| GATCTCCAGGCCCGACATGG | 87715713 | 87715735 | 0.02 |
| GTCCAGGCCCGACATGGTGGA | 87715717 | 87715739 | 0.12 |
| GTCTTTCCTTCCACCATGTC | 87715723 | 87715745 | 1.09 |
| GCTCAACTTGTAGGGGTAGC | 87715746 | 87715768 | 0.01 |
| GACATCAGAGCTCAACTTGT | 87715755 | 87715777 | 0.20 |
| GCATGATCTGATGCACATATG | 87715777 | 87715799 | 0.09 |
| GATTTTTATAATTAAAAAAGT | 87715838 | 87715860 | 0.06 |
| GTATGGAGGCAGAAGGGTT | 87715925 | 87715946 | 0.02 |
| GCGTGTGTATGGAGGCAGAA | 87715930 | 87715952 | 0.03 |
| GACACCGGCGTGTGTATGG | 87715938 | 87715959 | 6.88 |
| GTGTCTGCAGAAGCCAGAAA | 87715955 | 87715977 | 0.01 |
| GCAGCTACCAGTGCCCTTTTC | 87715968 | 87715990 | 0.10 |
| GTAGCTGTGAGCTACCCACTG | 87715985 | 87716007 | 0.01 |
| GAGCTACCCACTGTGGCCGC | 87715992 | 87716014 | 0.96 |
| GCAGTAACCTGCGGCCACAGT | 87715998 | 87716020 | 1.06 |
| GGCCGCAGGTTACTGAACTC | 87716006 | 87716028 | 0.33 |
| GACTGAACTCAGGGCTTCTGC | 87716017 | 87716039 | 17.98 |
| GGTTTAGTGATTAAAAAAGC | 87716045 | 87716067 | 0.01 |
| GCTAAACCACCCCTCCAGCCC | 87716061 | 87716083 | 0.02 |
| GCCACACCGGGGCTGGAGGGG | 87716066 | 87716088 | 2.09 |
| GATTTCAACCACACCGGGGC | 87716074 | 87716096 | 0.88 |
| GTTAAGATTTCAACCACACC | 87716079 | 87716101 | 4.89 |
| GTAACAAGGTTAAAAGTTATA | 87716098 | 87716120 | 0.34 |
| GGTTAAAAGTTATAAGGAGT | 87716104 | 87716126 | 0.02 |
| GTCTAACGCACGCGTCATGG | 87716152 | 87716174 | 0.69 |
| GTACCTTTGATCTCAGCACTG | 87716179 | 87716201 | 0.04 |
| GAGACAGGTGGATTTCTGAT | 87716207 | 87716229 | 0.38 |
| GAAAACAACACAAAACATAG | 87716233 | 87716255 | 0.01 |
| GCTATGTTTTGTAGCCGGGCG | 87716421 | 87716443 | 0.09 |
| GTAAAACTATGTTTTGTAGCC | 87716426 | 87716448 | 3.29 |
| GACAAAACATAGTTTTATAAT | 87716433 | 87716455 | 1.94 |
| GATGAGATGATTATTTTTATG | 87716456 | 87716478 | 0.00 |
| GAATGAACAATAAGTAGTGT | 87716532 | 87716554 | 0.02 |
| GAACAATAAGTAGTGTGGGCA | 87716537 | 87716559 | 0.13 |
| GCACCCTTAATCTCATCACTC | 87716566 | 87716588 | 0.06 |
| GCTGGCTGTGAACTCACTGA | 87716607 | 87716629 | 0.10 |
| GAGTTCACAGCCAGCCAACC | 87716615 | 87716637 | 1.40 |
| GACTATGCAGGCCAGGTTGGC | 87716625 | 87716647 | 0.01 |
| GGGTCTCACTATGCAGGCC | 87716633 | 87716654 | 0.96 |
| GTACATAGGGTCTCACTATGC | 87716638 | 87716660 | 1.82 |
| GATTTTTTGTCTTTTTACATA | 87716652 | 87716674 | 0.01 |
| GTTACTTTGTGTTTCAATGGC | 87716690 | 87716712 | 0.07 |
| GTAATTGTAAAGTTTAACTC | 87716720 | 87716742 | 0.08 |
| GGGGCATCTAGAAATTAGA | 87716741 | 87716762 | 0.02 |
| GCTAGAAATTAGAAGGCACTC | 87716748 | 87716770 | 0.04 |

| | | | |
|---|---|---|---|
| GTACTGCACCCCATTAGCA | 87716773 | 87716794 | 0.86 |
| GCAGAGTCTCCATGCTAATG | 87716781 | 87716803 | 0.75 |
| GATAACATGCGACAGCAACAA | 87716815 | 87716837 | 0.01 |
| GTTTTATCCAGCCTTAGAATC | 87716839 | 87716861 | 0.29 |
| GCACAAACCTCCCTGATTCTA | 87716849 | 87716871 | 0.81 |
| GGAGGTTTGTGTGGTAGCTT | 87716861 | 87716883 | 1.18 |
| GCTCTAATGCTGTGTCACAG | 87716883 | 87716905 | 0.01 |
| GTCTTGAATGTGTGTTAAAT | 87716907 | 87716929 | 1.89 |
| GATGTGTGTTAAATGGGCACC | 87716914 | 87716936 | 0.15 |
| GCAGGATTGAGTTGATCCC | 87716932 | 87716953 | 0.45 |
| GGATCAGCACGCTTGCTTGC | 87716949 | 87716971 | 1.48 |
| GTATTTGCAGTTTCATTTTGT | 87716970 | 87716992 | 0.00 |
| GAATGAAACTGCAAATATTCT | 87716977 | 87716999 | 0.09 |
| GCTGTTCTTGCTCATTGCTTA | 87717003 | 87717025 | 0.01 |
| GTTATGGTGATTTTTTTAAAG | 87717020 | 87717042 | 0.04 |
| GTTTTATAGTTTTCCCAGACA | 87717043 | 87717065 | 0.01 |
| GTATACAGAGAAACCGTGTC | 87717056 | 87717078 | 0.02 |
| GCTGTATACCCCTGACTCCTT | 87717071 | 87717093 | 5.76 |
| GCAGGACAGCCAAAGGAGTCA | 87717079 | 87717101 | 0.16 |
| GATTTCATCTCAGCATTCAGG | 87717156 | 87717178 | 0.10 |
| GTTCAGATCACAAGAGAGCTG | 87717193 | 87717215 | 2.71 |
| GCTCTCTTGTGATCTGAACA | 87717198 | 87717220 | 8.47 |
| GTCTCAAATTTCCCAACTTTT | 87717232 | 87717254 | 0.01 |
| GACGTACTTTCCCCTAAAAGT | 87717243 | 87717265 | 0.01 |
| GTACGTTGTGCTAGTTAATT | 87717260 | 87717282 | 1.50 |
| GTAGTTAATTGGGATTTCATA | 87717271 | 87717293 | 3.25 |
| GCTTCAAAAACAGAGAGCA | 87717299 | 87717320 | 0.01 |
| GTCTCTGTTTTTGAAGCATGA | 87717305 | 87717327 | 0.02 |
| GAAACATTTAACCCCATGTTG | 87717336 | 87717358 | 1.23 |
| GTAACCCCATGTTGAGGTTCA | 87717343 | 87717365 | 3.05 |
| GAAACTTGCTAACTTTTTAAA | 87717387 | 87717409 | 0.01 |
| GCTTATAATAGACTTAGCTCA | 87717410 | 87717432 | 0.52 |
| GGTGTATCTTATGTTTTTAC | 87717432 | 87717454 | 0.01 |
| GACATCGTTGAGCGTCTGCAT | 87717465 | 87717487 | 12.66 |
| GCAGACGCTCAACGATGTGC | 87717470 | 87717492 | 3.09 |
| GCACATGAGCGAAGCTAACAA | 87717505 | 87717527 | 7.53 |
| GACTGGTCGTACATAAGGAA | 87717541 | 87717563 | 5.74 |
| GAAGATGACTGGTCGTACATA | 87717546 | 87717568 | 4.31 |
| GTCATCTTGGAGAAAGGAAA | 87717561 | 87717583 | 5.62 |
| GAATTATATTGAAAGCCTCC | 87717586 | 87717608 | 2.34 |
| GTTCAACACAAGCATGCCTGG | 87717601 | 87717623 | 9.58 |
| GTTTTCAAAGTGCACGTCATT | 87717648 | 87717670 | 0.00 |
| GCAGATGTTCCACATCATTAC | 87717678 | 87717700 | 23.21 |
| GTTTTTTACCAGTAATGATG | 87717686 | 87717708 | 0.15 |
| GTCTTCCTAATGATGATAGAA | 87717722 | 87717744 | 0.01 |
| GATGAAGAAAGTCTCTCTTTC | 87717762 | 87717784 | 0.38 |
| GCATTAAAGAATGTATTACTC | 87717785 | 87717807 | 0.02 |
| GACATTCAGATCCTGACTAGA | 87717816 | 87717838 | 0.74 |
| GACTAGAAGGAAGACTTTTT | 87717829 | 87717851 | 0.00 |
| GAAGGAAGACTTTTTGGGG | 87717834 | 87717855 | 0.01 |
| GTTTTGGGGTGGCTAAAGTTT | 87717845 | 87717867 | 5.09 |
| GTCAGTATATAACTCACGCCC | 87717881 | 87717903 | 0.80 |

| | | | |
|---|---|---|---|
| GTCCTGGCTCTGGTGAGCCA | 87717898 | 87717920 | 2.62 |
| GCATTTTCCAAGTCCTGGCTC | 87717908 | 87717930 | 0.86 |
| GCCTACCCATTTTCCAAGTCC | 87717914 | 87717936 | 3.17 |
| GACTTGGAAAATGGGTAGGAA | 87717919 | 87717941 | 0.43 |
| GAATGGGTAGGAATGGTCTTC | 87717927 | 87717949 | 2.13 |
| GTTCTGGAAAAGCTGCAGCCT | 87717944 | 87717966 | 0.01 |
| GCTTACGAGGCGTGCTGGCCT | 87717961 | 87717983 | 0.01 |
| GAGTGTCTTACGAGGCGTGC | 87717967 | 87717989 | 2.45 |
| GAACACATGAGTGTCTTACG | 87717975 | 87717997 | 1.93 |
| GTTCATACTCATTTGAAAGG | 87717994 | 87718016 | 1.51 |
| GCTGGTTCTAGTTACTATCTC | 87718026 | 87718048 | 0.11 |
| GCTCTCTTATTTTCTAGTCTC | 87718045 | 87718067 | 0.01 |
| GAATTACATTGTAGAAATTA | 87718067 | 87718089 | 2.07 |
| GCACTTAGGGAACTAACAGGA | 87718092 | 87718114 | 1.74 |
| GATAATTTAAATACCCACTTA | 87718106 | 87718128 | 2.71 |
| GACAATTTACTTATCTGGGTT | 87718133 | 87718155 | 0.87 |
| GAATGTACAATTTACTTATCT | 87718138 | 87718160 | 0.76 |
| GATGCTACACGACATTTCTTT | 87718163 | 87718185 | 0.88 |
| GCATGGAGTTTACGTGATTTG | 87718183 | 87718205 | 0.00 |
| GTTTGAGGAGTTTGCCCAGAC | 87718199 | 87718221 | 5.19 |
| GTAGTTATATGTAACCAGTCT | 87718212 | 87718234 | 0.02 |
| GAATAGTATTCATTAATAAGT | 87718236 | 87718258 | 0.03 |
| GACTATTACTGAGTAGTCAGT | 87718253 | 87718275 | 3.83 |
| GATTAAATGAGTCTGTGAGCC | 87718290 | 87718312 | 0.01 |
| GCACTACGCACACACACTTCC | 87718308 | 87718330 | 0.43 |
| GCTAAGCTGGAGCATCACTG | 87718333 | 87718355 | 1.36 |
| GATGCTCCAGCTTAGCTGTG | 87718340 | 87718362 | 1.17 |
| GCATAAACCGCACAGCTAAGC | 87718346 | 87718368 | 12.37 |
| GGTTTATGATCCTACTCTC | 87718361 | 87718382 | 0.98 |
| GATCCTACTCTCTGGTTAGA | 87718368 | 87718390 | 0.04 |
| GTCTGGTTAGAGGGATCTGTG | 87718378 | 87718400 | 8.95 |
| GTGGGTTTGTCTGACTGAA | 87718397 | 87718418 | 2.44 |
| GAATGGTAAGAAAGTTGCATT | 87718414 | 87718436 | 1.31 |
| GAAGTTGCATTTGGGGCTGCG | 87718424 | 87718446 | 6.28 |
| GCATGTTTTTTGATGGCTTG | 87718462 | 87718484 | 0.00 |
| GTTTTTTGATGGCTTGTGGG | 87718466 | 87718488 | 0.02 |
| GATAATTGTCAATTGCAGTTT | 87718512 | 87718534 | 0.01 |
| GTTAGTAGTGAAAGAAATCAG | 87718554 | 87718576 | 0.92 |
| GTGAAAGAAATCAGTGGCC | 87718560 | 87718581 | 2.05 |
| GCTATAAGTGAATTATATGCC | 87718577 | 87718599 | 6.04 |
| GTTCACTTATAGGTCCCAATA | 87718589 | 87718611 | 6.92 |
| GTTGATTTACCTCCCATATT | 87718602 | 87718624 | 5.70 |
| GTCAACATACATTCGTCAGAC | 87718620 | 87718642 | 24.74 |
| GACCGGGGTGATTGTACTCA | 87718637 | 87718659 | 3.34 |
| GATTGTACTCATGGCCCAAAT | 87718647 | 87718669 | 1.11 |
| GGGCACAAAACACCCGATTT | 87718660 | 87718682 | 6.66 |
| GTGTTTTGTGCCCTGTGAGT | 87718670 | 87718692 | 10.65 |
| GACACTTCTGCCGACTCACA | 87718680 | 87718702 | 5.94 |
| GTCGGCAGAAGTGTCCATTG | 87718688 | 87718710 | 7.05 |
| GCAAGGATGCAATCCACAA | 87718702 | 87718723 | 3.12 |
| GATTGCATCCTTGCTCGAGT | 87718710 | 87718732 | 12.61 |
| GATCCTTGCTCGAGTCGGGGC | 87718716 | 87718738 | 7.75 |

| | | | |
|---|---|---|---|
| GCTGGTGACAGTCAACTGAA | 87718734 | 87718756 | 22.84 |
| GAAAGGCGTCTCCACATTCA | 87718751 | 87718773 | 6.41 |
| GCAGCATTTCAGCCATGAATG | 87718762 | 87718784 | 2.37 |
| GGAGACTGCTTCCATCCTC | 87718784 | 87718805 | 34.89 |
| GGGACTAGGACACATACCTG | 87718799 | 87718821 | 7.24 |
| GTGTCCTAGTCCCTTGAAAG | 87718809 | 87718831 | 3.42 |
| GCCACACGTCTCCACTTTCA | 87718820 | 87718842 | 1.01 |
| GAATGCATTTCAAATAAAACG | 87718842 | 87718864 | 0.73 |
| GATTTGTCTATAATATGCCAC | 87718870 | 87718892 | 1.34 |
| GCACTAAACTAAGAATACCTG | 87718886 | 87718908 | 2.70 |
| GATAAAGTACATACAATTCAC | 87718917 | 87718939 | 0.07 |
| GTACTTTATATTATCTTAAA | 87718931 | 87718953 | 20.01 |
| GTATTATCTTAAAAGGCTGAT | 87718939 | 87718961 | 0.02 |
| GATTGGAAGCTGTGTGTGA | 87718956 | 87718977 | 4.43 |
| GCTCGCCTTAATCTCAGCACT | 87718981 | 87719003 | 0.03 |
| GGTGGATGTTTGTGAGTTTG | 87719016 | 87719038 | 1.22 |
| GCTCATTACACAGACTTGAC | 87719039 | 87719061 | 1.59 |
| GAAGTCTGTGTAATGAGCTCA | 87719045 | 87719067 | 4.23 |
| GTAATGAGCTCAAGGATAGC | 87719053 | 87719075 | 8.21 |
| GAGATAGGGTCTGTCTTTG | 87719077 | 87719098 | 5.43 |
| GCTCTCTGGCTTTTTGAGATA | 87719090 | 87719112 | 0.37 |
| GCCAGAGAGAGAAATATGAA | 87719104 | 87719126 | 1.54 |
| GAAAACTCACATTAGTGATTT | 87719130 | 87719152 | 0.01 |
| GTACAGTATTTGTTAAACACA | 87719166 | 87719188 | 0.27 |
| GTTTAACAAATACTGTACTAC | 87719173 | 87719195 | 0.26 |
| GACGAAAGAGGCAGAGGTAG | 87719199 | 87719221 | 2.57 |
| GCAGAAAGACGAAAGAGGCAG | 87719205 | 87719227 | 2.20 |
| GGGGACAGAAAGACGAAAG | 87719211 | 87719232 | 0.96 |
| GCAGGGCTCATCATGGCAGAG | 87719229 | 87719251 | 5.62 |
| GATGCAGTCCAGGGCTCATCA | 87719237 | 87719259 | 1.20 |
| GATGATAAAAAATGCAGTCCA | 87719247 | 87719269 | 1.30 |
| GATCATGTAATTATGCGTTTC | 87719265 | 87719287 | 0.01 |
| GAATGATTATTAAGGAGTCTT | 87719295 | 87719317 | 6.96 |
| GCAGCTCATCAATGATTATTA | 87719304 | 87719326 | 3.98 |
| GATCATTGATGAGCTGGGAAG | 87719312 | 87719334 | 10.23 |
| GAGGAACCTCTACCTATGA | 87719331 | 87719352 | 8.71 |
| GACCTCTACCTATGATGGATT | 87719336 | 87719358 | 21.79 |
| GATGATGGATTTGGGTTAGCA | 87719346 | 87719368 | 8.74 |
| GATTACATTGCAACGAAGAT | 87719378 | 87719400 | 5.45 |
| GATGGGTGGCAAACATGCAAA | 87719403 | 87719425 | 14.33 |
| GAAGCAGTAAGTTCATGAAAA | 87719422 | 87719444 | 5.28 |
| GTTTTCATGAACTTACTGCTT | 87719425 | 87719447 | 9.24 |
| GATTATTAACAGTTGGTATT | 87719452 | 87719474 | 4.09 |
| GACATGTAGATTATTAACAGT | 87719459 | 87719481 | 7.19 |
| GTCACAGCGCTCACTACTG | 87719480 | 87719501 | 15.48 |
| GCACTTGGTAAAGCATAGTTA | 87719505 | 87719527 | 29.12 |
| GATGCTTTACCAAGTGAAAAA | 87719513 | 87719535 | 5.39 |
| GAAGCCCACCTTTTTTCACT | 87719521 | 87719543 | 0.02 |
| GTGGGCTTCTCCGCTCAGCG | 87719535 | 87719557 | 7.96 |
| GCCCTGAGGGCCACGCTGAG | 87719545 | 87719567 | 2.64 |
| GAATGGCACTTCAGGCCCTGA | 87719558 | 87719580 | 5.86 |
| GAATGCACTAATGGCACTTC | 87719567 | 87719589 | 0.94 |

| | | | |
|---|---|---|---|
| GAGAATAAAGAATGCACTAA | 87719576 | 87719598 | 4.75 |
| GTGCATTCTTTATTCTCCGT | 87719582 | 87719604 | 0.01 |
| GTTAAAAGCAATTAATCCCAA | 87719598 | 87719620 | 1.15 |
| GTTTAAGAACATATTTACCTC | 87719616 | 87719638 | 0.08 |
| GTCTGTAGACTAGAAAGCCAG | 87719632 | 87719654 | 4.00 |
| GTCTGTATCAAAATGATAACG | 87719671 | 87719693 | 1.90 |
| GAAAATGATAACGTGGGAGAC | 87719679 | 87719701 | 1.34 |
| GTATTATAGCTATCTGACTAT | 87719708 | 87719730 | 0.90 |
| GTTTTGTTTTTAACGTAAAGG | 87719735 | 87719757 | 0.01 |
| GGAGGATTGCAATTTCTTAA | 87719753 | 87719775 | 4.31 |
| GTGTATTATGCCTTGTGTGA | 87719796 | 87719818 | 0.75 |
| GTGCACCAACCTTCACACA | 87719806 | 87719827 | 1.74 |
| GATGACACAGAGTCTAGAAGA | 87719828 | 87719850 | 9.05 |
| GCAGAGTCTAGAAGACGGTAT | 87719834 | 87719856 | 1.62 |
| GGTATTGGCTTTTCTCAAGC | 87719849 | 87719871 | 2.70 |
| GTTCTCAAGCTGGAGCTAGAG | 87719860 | 87719882 | 3.02 |
| GTCTTAGCACTTGGGTCGGGG | 87719890 | 87719912 | 1.37 |
| GCAGGCTCAGTTCTTAGCACT | 87719900 | 87719922 | 2.45 |
| GTAAGAACTGAGCCTGTGTCC | 87719908 | 87719930 | 8.24 |
| GATAGAGCCCTTCCAGGACAC | 87719919 | 87719941 | 1.62 |
| GTAAAATCATAGAGCCCTTCC | 87719926 | 87719948 | 3.50 |
| GCCAGAAACATGGCTGGCTC | 87719950 | 87719972 | 0.15 |
| GCCATGTTTCTGGCTTTTTG | 87719959 | 87719981 | 0.01 |
| GCTTTTTGTGGTCTTTAGACC | 87719972 | 87719994 | 0.04 |
| GCCAGAGAATCTGGACTCACC | 87719990 | 87720012 | 0.82 |
| GCTAACAGTGCCAGAGAATC | 87720000 | 87720022 | 4.17 |
| GTTCTCTGGCACTGTTAGCGG | 87720005 | 87720027 | 1.48 |
| GGCACTGTTAGCGGTGGCGC | 87720011 | 87720033 | 0.86 |
| GTTAGCGGTGGCGCTGGTGT | 87720017 | 87720039 | 0.64 |
| GCGCTGGTGTTGGCGATCCC | 87720027 | 87720049 | 1.41 |
| GTGTTGGCGATCCCTGGGTG | 87720033 | 87720055 | 0.81 |
| GCTGGAAGGACGCCACACCCA | 87720044 | 87720066 | 0.69 |
| GGTGTGGCGTCCTTCCAGAA | 87720049 | 87720071 | 0.32 |
| GCGCTAACCAGTAGCCTTTC | 87720063 | 87720085 | 10.28 |
| GTCACTGACTCAGTGATGAGT | 87720085 | 87720107 | 2.60 |
| GCAGTGATGCTGACCTCCATG | 87720102 | 87720124 | 0.78 |
| GAATATAGAACCTCCTCATGG | 87720114 | 87720136 | 0.77 |
| GGTGATTATTTCAGAATAAG | 87720140 | 87720162 | 6.51 |
| GCATGTCTGTCTTCATTTCTT | 87720167 | 87720189 | 0.01 |
| GATTTCTTAGGAAAATGTGTG | 87720180 | 87720202 | 0.03 |
| GGAAAGCATCAAGACGATG | 87720262 | 87720283 | 2.57 |
| GCCGAGGCAAAAATAACAAAG | 87720282 | 87720304 | 0.04 |
| GACGCAAACTAATTCCCACCG | 87720299 | 87720321 | 0.01 |
| GTATCAATAATGCAAACAT | 87720323 | 87720344 | 2.09 |
| GTTATTGATACTTGTTAGAGA | 87720335 | 87720357 | 1.76 |
| GGCAATATTTGTAGTAGATC | 87720356 | 87720378 | 0.57 |
| GCATTCAGCACAGCATGAAAC | 87720379 | 87720401 | 10.15 |
| GAAGTATCTTAAAAGCAGGAC | 87720398 | 87720420 | 0.01 |
| GCACACAAGTATCTTAAAAGC | 87720403 | 87720425 | 1.78 |
| GTGTTGTGTGCTACAGAAG | 87720423 | 87720444 | 7.86 |
| GAAGTGGTTTCCGAATACTT | 87720438 | 87720460 | 5.76 |
| GTCATCTATCCCCTAAGTATT | 87720448 | 87720470 | 4.80 |

| | | | |
|---|---|---|---|
| GGGGATAGATGAGTATAGCG | 87720459 | 87720481 | 0.50 |
| GTAAGCCCAGCCCCCATTG | 87720493 | 87720514 | 0.65 |
| GCACGACCACAATGGGGGCT | 87720498 | 87720520 | 2.17 |
| GATGCTAGCACGACCACAATG | 87720504 | 87720526 | 5.69 |
| GTGGTCGTGCTAGCATGTAC | 87720511 | 87720533 | 2.97 |
| GCTAGCATGTACTGGACTG | 87720519 | 87720540 | 2.67 |
| GAAGCTGGACAGTGCTCAATT | 87720557 | 87720579 | 2.21 |
| GCCGCCTCAGTTACCTGAAGC | 87720573 | 87720595 | 0.64 |
| GTAAGCAGAAGTGAGGCTACG | 87720607 | 87720629 | 1.02 |
| GCCTCACTTCTGCTTACACG | 87720614 | 87720636 | 5.13 |
| GACTCGAGTAACACACGGCTA | 87720643 | 87720665 | 10.60 |
| GAAAGTACTCGAGTAACACA | 87720649 | 87720671 | 0.36 |
| GTCGAGTACTTTCTCCACTGC | 87720660 | 87720682 | 1.07 |
| GCTTTCTCCACTGCTGGTGTG | 87720667 | 87720689 | 9.66 |
| GCCTTACCGCACACCAGCAG | 87720673 | 87720695 | 0.22 |
| GCGGTAAGGCAGAATGAGCG | 87720686 | 87720708 | 1.17 |
| GAGCGAGGGTGCAGTCACA | 87720701 | 87720722 | 5.85 |
| GCAGTCACATGGTTGACGAT | 87720711 | 87720733 | 0.32 |
| GATGGTTGACGATTGGTTAAA | 87720719 | 87720741 | 0.52 |
| GTTGGTTAAAAGGTCTATCTG | 87720730 | 87720752 | 5.00 |
| GCTGAGGTGCTCTGAACCGAT | 87720747 | 87720769 | 3.06 |
| GCTCTGAACCGATTGGCTA | 87720754 | 87720775 | 5.86 |
| GTTTCAAATACCTTAGCCAAT | 87720762 | 87720784 | 0.01 |
| GTAAAATTACACTTTCTAATG | 87720787 | 87720809 | 1.27 |
| GTTTCTAATGAGGTGATAAAC | 87720798 | 87720820 | 1.97 |
| GACAGCACTTATGTGAATGGA | 87720826 | 87720848 | 2.31 |
| GAAACGGGTGGCGTGGAAG | 87720855 | 87720876 | 0.74 |
| GTCCACGCCACCCGTTTCTGT | 87720860 | 87720882 | 6.30 |
| GCAGACACCTACAGAAACGGG | 87720866 | 87720888 | 5.88 |
| GGTGTCTGTGATCAGAGTTT | 87720881 | 87720903 | 1.44 |
| GTCAGAGTTTCGGGATTCACG | 87720892 | 87720914 | 10.33 |
| GCTGAGCTCGCTAACTTCCCG | 87720915 | 87720937 | 6.21 |
| GCGCACGCTATCACGTGCCT | 87720932 | 87720954 | 3.78 |
| GATAGCGTGCGCCAAGCAGA | 87720943 | 87720965 | 3.49 |
| GAAGCTCTAGAGCCTTCTGCT | 87720954 | 87720976 | 19.27 |
| GCTTGAAGAATTTCAGAACAT | 87720974 | 87720996 | 20.12 |
| GTCAGAACATTGGAACCTCGC | 87720985 | 87721007 | 5.48 |
| GGCTTCGTCACATCCCAGCG | 87720999 | 87721021 | 9.57 |
| GGGATGTGACGAAGCCGAGC | 87721006 | 87721028 | 4.90 |
| GCGTCTCTTTGCAGCCGGCT | 87721020 | 87721042 | 10.55 |
| GGCTGCAAAGAGACGCTGCC | 87721027 | 87721049 | 10.63 |
| GAGACGCTGCCTGGAAAGAG | 87721036 | 87721058 | 15.22 |
| GCTGACCGACCTCTCTTTCC | 87721045 | 87721067 | 8.88 |
| GGTCAGCAGGGCATTCGTCA | 87721061 | 87721083 | 8.29 |
| GCAACTCATCTTGGTTGCG | 87721107 | 87721128 | 0.01 |
| GATGAGTTGCGCGTTCCCTG | 87721119 | 87721141 | 2.73 |
| GTAAATACTAGAATCCCACA | 87721134 | 87721156 | 1.49 |
| GGATTCTAGTATTTACTTTT | 87721141 | 87721163 | 0.01 |
| GTACTTTTTGGTCATTCTTAC | 87721154 | 87721176 | 0.00 |
| GCATAACCGAAAACCCATCCC | 87721191 | 87721213 | 8.61 |
| GATAATCCAGGGATGGGTTTT | 87721196 | 87721218 | 0.01 |
| GAACAACAATAATCCAGGGAT | 87721203 | 87721225 | 6.96 |

| | | | |
|---|---|---|---|
| GCCTCAAACAACAATAATCCA | 87721208 | 87721230 | 1.87 |
| GATTATTGTTGTTTGAGGTA | 87721213 | 87721235 | 2.97 |
| GTCTCACCTTATAGCTCTAGC | 87721236 | 87721258 | 0.37 |
| GTAGACCAGCTAGAGCTATA | 87721241 | 87721263 | 5.71 |
| GTCTAGCTGGTCTACAACCGC | 87721250 | 87721272 | 0.05 |
| GAGGGTGAGGATTAGACCTG | 87721266 | 87721288 | 1.06 |
| GGTTAGGAGGGTGAGGGTG | 87721279 | 87721300 | 3.86 |
| GCAGCTTAGGTTAGGAGGGTG | 87721285 | 87721307 | 7.10 |
| GCAGATCAGCTTAGGTTAGG | 87721291 | 87721313 | 0.86 |
| GTTAGAGTTGCAGATCAGCTT | 87721299 | 87721321 | 3.83 |
| GCATCCTTGGAGGCAAAGGC | 87721326 | 87721348 | 3.31 |
| GTTTAATCCCAGCATCCTTGG | 87721336 | 87721358 | 0.66 |
| GATATGAGAACCACCAAAACC | 87721358 | 87721380 | 7.30 |
| GAGAGAGAACCAGGTTTTGG | 87721367 | 87721389 | 2.28 |
| GGGAGAGACAGAGACAGAGG | 87721461 | 87721483 | 0.04 |
| GAAAGGATGGGGGGGGGGGG | 87721512 | 87721534 | 1.34 |
| GTCGTAGAAAGGATGGGGGGG | 87721517 | 87721539 | 0.01 |
| GCCTCCTTCGTAGAAAGGATG | 87721523 | 87721545 | 0.83 |
| GTCTAACCCTCCTTCGTAGAA | 87721529 | 87721551 | 4.89 |
| GCTACGAAGGAGGGTTAGAGC | 87721534 | 87721556 | 4.49 |
| GTTAGAGCTGGAGCATAGTCC | 87721547 | 87721569 | 0.95 |
| GGAGCATAGTCCTGGCACGC | 87721555 | 87721577 | 1.66 |
| GTCCTGGCACGCAGGAATCTG | 87721564 | 87721586 | 3.02 |
| GCACGCAGGAATCTGCGGATC | 87721570 | 87721592 | 3.14 |
| GCGGATCTGGCTCTGTCAC | 87721583 | 87721604 | 0.53 |
| GCACTGCCCTCCTGAGAGCTG | 87721607 | 87721629 | 2.96 |
| GCAAATCCACAGCTCTCAGGA | 87721612 | 87721634 | 0.98 |
| GAGCTGTGGATTTGGCAGA | 87721621 | 87721642 | 0.96 |
| GAATTGACATCTATAGCTTTA | 87721687 | 87721709 | 0.10 |
| GTCAATTGATAAGTGTAACAG | 87721704 | 87721726 | 0.54 |
| GTGTAACAGCGGCAGCAAAC | 87721715 | 87721737 | 0.40 |
| GACAGGACTCCTGCTCCATGC | 87721733 | 87721755 | 1.48 |
| GTCTAAGACCAGCATGGAGC | 87721741 | 87721763 | 0.24 |
| GCAGAAAGTCTAAGACCAGCA | 87721747 | 87721769 | 0.99 |
| GCTTAGACTTTCTGAGGAGAG | 87721757 | 87721779 | 0.01 |
| GTTTCTGAGGAGAGCGGACTG | 87721764 | 87721786 | 0.32 |
| GGAGAGCGGACTGTGGGCAG | 87721771 | 87721793 | 0.91 |
| GATCTTCCTCTGTAGCTATG | 87721809 | 87721831 | 2.07 |
| GTCCTCTGTAGCTATGGGGTC | 87721814 | 87721836 | 0.26 |
| GCTATGGGGTCAGGGTTAGA | 87721823 | 87721845 | 0.02 |
| GCAGGCCTGTGGCTGTGAAAA | 87721879 | 87721901 | 0.54 |
| GTTTATTACTGAGCAGGCCTG | 87721891 | 87721913 | 2.75 |
| GAAGTTCTTTTATTACTGAGC | 87721898 | 87721920 | 0.02 |
| GAAAAGAACTTCACTGCTGCC | 87721911 | 87721933 | 0.25 |
| GACTTCACTGCTGCCTGGTTC | 87721917 | 87721939 | 0.77 |
| GCCTGGTTCTGGCAGCTTCG | 87721928 | 87721950 | 1.55 |
| GTAAGGTCTGTCACAATGTA | 87721953 | 87721975 | 24.06 |
| GTCACAATGTGCAGTGCAGTA | 87721970 | 87721992 | 0.35 |
| GCATTGTGACAGGCCTTACAG | 87721985 | 87722007 | 2.38 |
| GTCACATGTATGCCACTGTA | 87721997 | 87722019 | 2.69 |
| GACATGTGACAGACCTTACAG | 87722011 | 87722033 | 0.45 |
| GTCACCACGTATGCCACTGTA | 87722023 | 87722045 | 0.78 |

| | | | |
|---|---|---|---|
| GCATACGTGGTGACAGATAA | 87722033 | 87722055 | 9.60 |
| GTATTTGTAGCTTTTACCCAG | 87722058 | 87722080 | 0.46 |
| GTACAGGAAAAAAAGCCCACT | 87722073 | 87722095 | 0.63 |
| GTTTTTTCCTGTAAGGATAGA | 87722084 | 87722106 | 0.01 |
| GTAGCAACCGTCTATCCTTAC | 87722090 | 87722112 | 0.58 |
| GTAGACGGTTGCTATACAAAA | 87722100 | 87722122 | 3.80 |
| GTCTAAAACTATGGGCAAGGT | 87722133 | 87722155 | 6.06 |
| GCTTTCCATATCTAAAACTA | 87722143 | 87722165 | 4.16 |
| GAAAGCTGCCATCCTGAGCA | 87722160 | 87722182 | 2.50 |
| GAGACACTCCGTGCTCAGGA | 87722168 | 87722190 | 0.49 |
| GGCTGGATATTAAGTGAGTC | 87722196 | 87722218 | 0.96 |
| GTCAGCTCTGTGGGTCTGGC | 87722213 | 87722235 | 0.01 |
| GACCCACAGAGCTGACGATT | 87722220 | 87722242 | 0.12 |
| GAGCTGACGATTGGGATTGG | 87722228 | 87722250 | 0.00 |
| GGGCTATGTTACATGATTCC | 87722250 | 87722272 | 0.16 |
| GTTTCAGTTTTCTTGGAAACC | 87722268 | 87722290 | 0.01 |
| GTACACAATTTCAGTTTTCT | 87722276 | 87722298 | 0.01 |
| GTATTTTGAAAAGTCAGCTAA | 87722317 | 87722339 | 0.01 |
| GTCCTTGCTGCCATTAGTCC | 87722349 | 87722371 | 2.20 |
| GGGACAAGAGCCCGGACTAA | 87722359 | 87722381 | 8.24 |
| GAAACCAAAGGGACAAGAGCC | 87722367 | 87722389 | 0.02 |
| GATGTACTTTCTAAAACCAAA | 87722379 | 87722401 | 0.03 |
| GTAGAAAGTACATTTTTATCT | 87722390 | 87722412 | 0.02 |
| GCATTTTTATCTCGGCAGTGC | 87722399 | 87722421 | 0.00 |
| GATCTCGGCAGTGCCGGAGCT | 87722406 | 87722428 | 0.97 |
| GCTCAGGCAAAGCCAAGCTC | 87722418 | 87722440 | 0.01 |
| GCTTGGCTTTGCCTGAGCGA | 87722423 | 87722445 | 24.14 |
| GCTTTGCCTGAGCGAGGGCT | 87722428 | 87722450 | 1.53 |
| GCTCCTCCCAAGCCCTCGCTC | 87722434 | 87722456 | 0.66 |
| GAGGGCTTGGGAGGAGAGAG | 87722441 | 87722463 | 6.65 |
| GAGGGGTTTTGTGTTTCAT | 87722460 | 87722481 | 9.92 |
| GCCTGCCTCACAGCACATGT | 87722497 | 87722519 | 3.09 |
| GGAGTCAGAGGTCAGTGTTC | 87722518 | 87722540 | 0.02 |
| GCTGTTGCCTGCCTACCCTGT | 87722549 | 87722571 | 0.56 |
| GCTGAGACCTACAGGGTAGGC | 87722555 | 87722577 | 0.24 |
| GTGGATCTCTGAGACCTACA | 87722563 | 87722585 | 1.41 |
| GTCTCAGAGATCCACTTCAGG | 87722572 | 87722594 | 3.51 |
| GAGATCCACTTCAGGTGGCC | 87722577 | 87722599 | 0.89 |
| GCAAGCCTGGCCACCTGAAG | 87722582 | 87722604 | 2.80 |
| GAAGGCATGTAAAGGCAAGCC | 87722595 | 87722617 | 3.84 |
| GCAAGACTGCAAGGCATGTAA | 87722604 | 87722626 | 9.06 |
| GAAGGAGCTGGCAAGACTGCA | 87722614 | 87722636 | 1.65 |
| GTCTTGCCAGCTCCTTCCGTG | 87722622 | 87722644 | 2.68 |
| GTAAAACCACACGGAAGGAGC | 87722627 | 87722649 | 0.06 |
| GTCCTTCCGTGTGGTTTTAAA | 87722632 | 87722654 | 1.40 |
| GCTCTCCATTTAAAACCACA | 87722637 | 87722659 | 0.19 |
| GATGGAGAGCTATTGTTCTTA | 87722651 | 87722673 | 1.36 |
| GATCAGCATCACAGGGGCAAC | 87722680 | 87722702 | 0.85 |
| GCCCCTGTGATGCTGATGCC | 87722686 | 87722708 | 0.14 |
| GCTGATGCCAGGTATAAAAA | 87722697 | 87722719 | 2.99 |
| GAAATGGCCCATTTTTATACC | 87722704 | 87722726 | 0.02 |
| GACAGCACATCAAAAACTAAA | 87722721 | 87722743 | 1.98 |

| | | | |
|---|---|---|---|
| GTCACAACAAATGTATGAAGT | 87722800 | 87722822 | 0.33 |
| GCTGACTTGCACAAGCAAAAT | 87722836 | 87722858 | 0.01 |
| GAGTTTCAGTGTTTATGCCT | 87722860 | 87722882 | 1.65 |
| GAAAAGAACTTAAGGTTTCCA | 87722877 | 87722899 | 0.01 |
| GTCTCAGCAAAAAAGAACTTA | 87722886 | 87722908 | 6.52 |
| GTTCCATGTCAGATGAAGCAG | 87722914 | 87722936 | 5.34 |
| GCACTGTAATTTCAGCTGACA | 87722957 | 87722979 | 0.01 |
| GTCTTATTAATAGCTTGCTC | 87722985 | 87723007 | 2.38 |
| GGATAAGTTCATAGCAACCA | 87723006 | 87723028 | 1.51 |
| GTTCATAGCAACCAAGGACTT | 87723013 | 87723035 | 0.39 |
| GTCTGAGAATCCCAAAGTCCT | 87723023 | 87723045 | 0.00 |
| GCACACATTTTTATCATAAAT | 87723054 | 87723076 | 0.01 |
| GACATCTAATAGCATATCTAG | 87723081 | 87723103 | 0.45 |
| GAAAGAGTCCATTGCTTACGC | 87723114 | 87723136 | 2.05 |
| GTCATCTCCAGCGTAAGCAA | 87723121 | 87723143 | 1.10 |
| GCCTCTGGGAAAGCAGCAAT | 87723143 | 87723165 | 1.55 |
| GCTGCTTTCCCAGAGGCCCC | 87723149 | 87723171 | 0.02 |
| GAGCCCAGCCCGGGGCCTCT | 87723157 | 87723179 | 0.01 |
| GAGGCTGGGAGCCCAGCCCG | 87723165 | 87723187 | 0.02 |
| GGGCTCCCAGCCTCCCACAC | 87723174 | 87723196 | 3.18 |
| GGTCAAGATCCACCCAGTGT | 87723187 | 87723209 | 0.08 |
| GGGTCGGATCTCCTGGAGCT | 87723219 | 87723241 | 0.07 |
| GGAGACAGGGTCGGATCTCC | 87723226 | 87723248 | 5.43 |
| GTATATGCCAGGAGACAGGGT | 87723235 | 87723257 | 0.02 |
| GCTGGCATATAAGGGTGTCCA | 87723248 | 87723270 | 1.33 |
| GTGGGTATGACATATGTCCG | 87723265 | 87723287 | 1.09 |
| GTCATTACAACGTACTCATG | 87723284 | 87723306 | 5.45 |
| GAGTACGTTGTAATGACTTT | 87723290 | 87723312 | 2.43 |
| GACACGTGTGTTTTTCAGCA | 87723312 | 87723334 | 8.95 |
| GCAAGGTGAGAAAATTATTC | 87723329 | 87723351 | 3.95 |
| GTATTCTGGAGTTCCTGTCGA | 87723344 | 87723366 | 5.81 |
| GTTCCTGTCGAAGGTCAAGC | 87723353 | 87723375 | 7.58 |
| GGTGCCCTTTACTGCCATGT | 87723374 | 87723396 | 5.09 |
| GGAGATGCTCTCCTCCGACA | 87723388 | 87723410 | 7.77 |
| GTTTCAGTTGCTTCAGCTTCG | 87723409 | 87723431 | 4.30 |
| GCTGAAGCAACTGAAAGCCG | 87723416 | 87723438 | 24.41 |
| GTTGTTCTTTGCGACCACCT | 87723433 | 87723455 | 13.88 |
| GTCGTAAACGAGATCATTTCA | 87723460 | 87723482 | 16.22 |
| GAGATCATTTCACGGATAA | 87723468 | 87723489 | 17.15 |
| GCATTTCACGGATAAAGGCTC | 87723473 | 87723495 | 67.29 |
| GATAAAGGCTCCGGCTCCGTG | 87723483 | 87723505 | 0.00 |
| GCCACTGTCCCCTCACGGAGC | 87723492 | 87723514 | 0.00 |
| GCCCACCCACTGTCCCCTCA | 87723498 | 87723520 | 0.01 |
| GGACAGTGGGTGGGCGTGCA | 87723506 | 87723528 | 1.09 |
| GACCTTTACTGTGTGCTGTTC | 87723537 | 87723559 | 1.49 |
| GTTCTGGGTAGTGCTCCCCAC | 87723554 | 87723576 | 9.77 |
| GTAGTGCTCCCCACTGGAACC | 87723561 | 87723583 | 2.74 |
| GCTGCTAGGCCAGGTTCCAG | 87723570 | 87723592 | 0.99 |
| GAACCTGGCCTAGCAGCATC | 87723576 | 87723598 | 0.65 |
| GAAACACTTCCGGATGCTGCT | 87723584 | 87723606 | 3.50 |
| GCAAGCATCACTAAACACTTC | 87723595 | 87723617 | 1.66 |
| GAAAACATTACCGTCTGTAGC | 87723618 | 87723640 | 0.01 |

| | | | |
|---|---|---|---|
| GAAACAGCACCCAGCTACAGA | 87723627 | 87723649 | 1.77 |
| GTAGCTGGGTGCTGTTTAAA | 87723633 | 87723655 | 2.72 |
| GGGTGCTGTTTAAATGGGGA | 87723639 | 87723661 | 0.23 |
| GAAATCATGTAGTTTGTACAT | 87723696 | 87723718 | 1.00 |
| GTTTCTTAGAAAAGAAATACA | 87723724 | 87723746 | 0.58 |
| GTATTTCTTTTCTAAGAAAAT | 87723729 | 87723751 | 0.06 |
| GAAGAAAATAGGTAATAGATA | 87723741 | 87723763 | 1.18 |
| GTAATAGATAAGGAACCAGTG | 87723752 | 87723774 | 12.50 |
| GATAAGGAACCAGTGAGGAA | 87723757 | 87723779 | 4.85 |
| GGCTCTGGCCCTTTCCTCAC | 87723766 | 87723788 | 4.68 |
| GCTCACTTGCTTCCTCGGCTC | 87723781 | 87723803 | 0.83 |
| GTAGCTCTCACTTGCTTCCT | 87723787 | 87723809 | 6.65 |
| GACAGGAACCACCTCACCGTG | 87723808 | 87723830 | 1.78 |
| GAACCACCTCACCGTGAGGGG | 87723813 | 87723835 | 2.67 |
| GTCTACCCACCCCTCACGGTG | 87723818 | 87723840 | 4.13 |
| GCCGACTCTACCCACCCCTCA | 87723823 | 87723845 | 14.90 |
| GGGTAGAGTCGGGTCTGCTG | 87723834 | 87723856 | 0.52 |
| GGTCTGCTGAGGGAGTACAG | 87723845 | 87723867 | 11.24 |
| GAGGGTGCTTGCACACCAGT | 87723864 | 87723886 | 3.05 |
| GTTGAATGTTTACATTCCCAC | 87723879 | 87723901 | 0.01 |
| GCACAAGTTTTCACTGTGTG | 87723903 | 87723925 | 0.03 |
| GAAACTTGTGCTTCCTATCCC | 87723916 | 87723938 | 4.62 |
| GCTTCTCTATCTTCCTGGGAT | 87723928 | 87723950 | 1.51 |
| GTCTCTCTTCTCTATCTTCCT | 87723933 | 87723955 | 0.96 |
| GAGAAACATCCCAACAGAA | 87723954 | 87723975 | 1.54 |
| GTTTGTATGTCCATTCTGTT | 87723963 | 87723985 | 0.98 |
| GTTGCATCGTAGCCAAAAGG | 87724001 | 87724023 | 3.08 |
| GCTGGGGCTGCTCCCACCTTT | 87724013 | 87724035 | 1.66 |
| GCTCATCCAGTTAGGATGCTG | 87724030 | 87724052 | 6.41 |
| GTAACACACTCTCATCCAGTT | 87724039 | 87724061 | 1.50 |
| GAGAGTGTGTTAGTGTGCTC | 87724050 | 87724072 | 8.40 |
| GTTAAAAGAAACCAAGCAACA | 87724096 | 87724118 | 0.01 |
| GCCAATCTTACCCATGTTGCT | 87724106 | 87724128 | 0.33 |
| GCAGCCACAGAAGTCCACACA | 87724162 | 87724184 | 2.94 |
| GCATCATGCAGCTACCATGTG | 87724175 | 87724197 | 1.24 |
| GGTAGCTGCATGATGAGTCG | 87724183 | 87724205 | 2.68 |
| GATGAGTCGTGGGTAAGCTG | 87724194 | 87724216 | 0.01 |
| GGGTAAGCTGGGGTTGGTGT | 87724204 | 87724226 | 1.41 |
| GCTGGGGTTGGTGTTGGGTT | 87724210 | 87724232 | 0.01 |
| GGGTTTGGTGTTAGTGAGA | 87724225 | 87724246 | 0.33 |
| GTGAGATGGAAGATCAGTCC | 87724238 | 87724260 | 1.58 |
| GACTCACAGGTGTGTGTACCC | 87724256 | 87724278 | 1.56 |
| GTTTTGGTGTGCTCACTCAC | 87724270 | 87724292 | 8.01 |
| GACACCAAAACCGCTGAGTTG | 87724283 | 87724305 | 3.10 |
| GCATGCGTGTCCACAACTCAG | 87724292 | 87724314 | 0.88 |
| GCATGATCGTTGCATGTGAAC | 87724311 | 87724333 | 0.95 |
| GCATGTGAACTGGATCAGTAA | 87724322 | 87724344 | 0.05 |
| GCAAACAGCTATAAACACAGA | 87724360 | 87724382 | 0.00 |
| GTATAAACACAGAAGGGTCTT | 87724368 | 87724390 | 0.04 |
| GACACAGAAGGGTCTTAGGGC | 87724373 | 87724395 | 3.89 |
| GGGTCTTAGGGCTGGGCAG | 87724381 | 87724402 | 0.01 |
| GCTGGGACTCACTACGTTCAG | 87724411 | 87724433 | 1.24 |

| | | | |
|---|---|---|---|
| GTTTGAATCCGTGGCTGCCCT | 87724429 | 87724451 | 0.24 |
| GCAGCCACGGATTCAAAGTC | 87724435 | 87724457 | 2.50 |
| GTCTGCTAACTAGAAAACTG | 87724461 | 87724483 | 0.64 |
| GGCAGTGAGTGGCTTGGCAT | 87724491 | 87724513 | 0.24 |
| GGAGCAGGCAGTGAGTGGCT | 87724497 | 87724519 | 0.04 |
| GGTCTGGAGGAGGATGGAGC | 87724512 | 87724534 | 0.01 |
| GGCGTGGTCAGGTCTGGAGG | 87724522 | 87724544 | 0.37 |
| GCAGGCGTGGTCAGGTCTGG | 87724525 | 87724547 | 0.86 |
| GACCTGACCACGCCTGCAAG | 87724531 | 87724553 | 0.84 |
| GGCACTGCCACTTGCAGGCG | 87724538 | 87724560 | 0.33 |
| GCAGTGGGCACTGCCACTTGC | 87724543 | 87724565 | 1.06 |
| GGCAGTGCCCACTGTTGGTC | 87724552 | 87724574 | 1.39 |
| GCTCCTATCCTGACCAACAGT | 87724559 | 87724581 | 1.07 |
| GATAGGAGGGAGTGTTCCTGG | 87724575 | 87724597 | 0.19 |
| GTTACTCAGCTTGTACCCACC | 87724590 | 87724612 | 2.20 |
| GCTAAACCATTCTGCTATCCC | 87724623 | 87724645 | 0.01 |
| GTGCCCCTGGGATAGCAGAA | 87724628 | 87724650 | 0.94 |
| GCTGCTATCCCAGGGGCACAT | 87724633 | 87724655 | 0.01 |
| GGACATTCCAATGTGCCCCT | 87724640 | 87724662 | 0.01 |
| GATTGGAATGTCCATGGCAGC | 87724651 | 87724673 | 0.06 |
| GTTTCTGTCTTCCAGCTGCCA | 87724661 | 87724683 | 2.90 |
| GGTCTGTTAGCATCGCTTGG | 87724696 | 87724718 | 0.02 |
| GATGCTAACAGACCTTCAAAC | 87724706 | 87724728 | 0.97 |
| GACCTTCAAACAGGAAGCGG | 87724715 | 87724737 | 0.46 |
| GACTCAAATTTCATGTAAGT | 87724749 | 87724771 | 7.71 |
| GAAATTTGAGTCCCAGACAA | 87724760 | 87724782 | 0.02 |
| GGGATCCTAGGCCCTTGTCT | 87724771 | 87724793 | 0.09 |
| GACAAGGGCCTAGGATCCC | 87724775 | 87724796 | 0.14 |
| GGCCTAGGATCCCTGGACTC | 87724781 | 87724803 | 0.79 |
| GTGTGTAACTCCAGAGTCCA | 87724791 | 87724813 | 0.81 |
| GCTCTGGAGTTACACACAGCT | 87724798 | 87724820 | 0.01 |
| GCACAGCTAGGAGCCACTATG | 87724811 | 87724833 | 0.46 |
| GAAATCGAACCCCAGTCCTC | 87724840 | 87724862 | 0.03 |
| GCCTAATTTCCAGAGGACTG | 87724849 | 87724871 | 0.50 |
| GTTTAGTGCCTAATTTCCAG | 87724856 | 87724878 | 0.09 |
| GCAGCTCTCTCCAGACGAGTG | 87724878 | 87724900 | 3.11 |
| GACTTAAATTCCACACTCGTC | 87724887 | 87724909 | 0.89 |
| GTGTAAAACTGTTCCCAGCA | 87724913 | 87724935 | 0.06 |
| GTTTTTTAGTTGCACCCTGCT | 87724926 | 87724948 | 0.00 |
| GACTAAAAAAATGACTAAACC | 87724940 | 87724962 | 0.00 |
| GTAAACCAGGAGAGTCCTGGT | 87724954 | 87724976 | 0.92 |
| GCTGCCCTACCAGGACTCTCC | 87724958 | 87724980 | 1.21 |
| GGGAAAAAAACCTGCCCTACC | 87724968 | 87724990 | 0.87 |
| GACTCACTGTCGGGAGACCCT | 87724988 | 87725010 | 1.17 |
| GCCACTTGGGAACTCACTGT | 87724999 | 87725021 | 1.02 |
| GAGTTCCCAAGTGGCTTTGC | 87725007 | 87725029 | 0.95 |
| GAAAGACCTGCAAAGCCACTT | 87725012 | 87725034 | 1.97 |
| GTTGAAAGCTCCTGTTAGGA | 87725036 | 87725058 | 0.68 |
| GACAAAACTGCCCATCCTAAC | 87725046 | 87725068 | 10.98 |
| GTTTCGTACATAAACGTTT | 87725067 | 87725088 | 0.01 |
| GTTTCGGTGTGAAGATAAT | 87725082 | 87725103 | 0.01 |
| GTAAACAGAACTTTAAAGGTT | 87725111 | 87725133 | 0.01 |

| | | | |
|---|---|---|---|
| GCACAGTAAACAGAACTTTAA | 87725116 | 87725138 | 0.00 |
| GAAGAGACGCTAGCAAAGAAA | 87725141 | 87725163 | 0.30 |
| GTCTTTGCTAGCGTCTCTTTT | 87725146 | 87725168 | 2.50 |
| GTGCCAAAGACTTGTGAAGA | 87725170 | 87725192 | 0.02 |
| GAAGAAGGCCAACTCAAATAA | 87725186 | 87725208 | 0.03 |
| GAAAGACCCTTTATTTGAGT | 87725193 | 87725215 | 1.44 |
| GGGTCTTTCTGTGACAAGCC | 87725207 | 87725229 | 1.12 |
| GTGACAAGCCAGGCCAGTC | 87725217 | 87725238 | 0.56 |
| GTCGACAGCTCCAGACTGGCC | 87725225 | 87725247 | 0.01 |
| GGGGATCGACAGCTCCAGAC | 87725230 | 87725252 | 0.05 |
| GCAGTGAAGGCTGCGGCAGG | 87725249 | 87725271 | 0.02 |
| GGCACCGCAGTGAAGGCTG | 87725256 | 87725277 | 0.01 |
| GGCGGTGGCACCGCAGTGA | 87725262 | 87725283 | 0.81 |
| GCGGTGCCACCGCCAGCTGC | 87725270 | 87725292 | 0.48 |
| GAATCCCCCTGCAGCTGGCGG | 87725276 | 87725298 | 0.77 |
| GCAAATAATCCCCCTGCAGC | 87725282 | 87725304 | 0.01 |
| GTTTATTATTTCTTGCTACAC | 87725316 | 87725338 | 0.01 |
| GTTCACTGTGCAACCCTGAC | 87725339 | 87725361 | 1.41 |
| GCACTGGAAGAGGCAGAGGC | 87725382 | 87725404 | 0.01 |
| GCCTCTGCCTCTTCCAGTGC | 87725385 | 87725407 | 8.79 |
| GCTTAATCCCAGCACTGGAAG | 87725392 | 87725414 | 0.04 |
| GAACGCTCTTAATCCCAGCAC | 87725398 | 87725420 | 1.98 |
| GAGCGTTCACCACCTCTCC | 87725414 | 87725435 | 1.24 |
| GCCTGGCTGAGCCAGGAGAGG | 87725423 | 87725445 | 0.04 |
| GTAAAGATCCCTGGCTGAGCC | 87725431 | 87725453 | 0.01 |
| GTTGAATTATGTAAAGATCCC | 87725441 | 87725463 | 3.92 |
| GCTGAAATATTTCCATCCCT | 87725482 | 87725504 | 0.05 |
| GCTTCACAGCCGTCCAAGGGA | 87725494 | 87725516 | 2.47 |
| GCTTGGACGGCTGTGAAGGGG | 87725500 | 87725522 | 0.08 |
| GACTGCTGCACAAAAGCACGT | 87725529 | 87725551 | 0.01 |
| GCACAAAAGCACGTTGGAGA | 87725535 | 87725557 | 0.11 |
| GAGATGGAAATCAGCTACGG | 87725551 | 87725573 | 0.09 |
| GCTACGGTGGGTTGAGTTGTT | 87725565 | 87725587 | 0.07 |
| GTTGTTAGGGCCACCGAGT | 87725579 | 87725600 | 3.21 |
| GCGTGTTGTCTCCGACTCGG | 87725589 | 87725611 | 0.46 |
| GTCGGAGACAACACGCAGTCT | 87725597 | 87725619 | 3.76 |
| GACGACTTCCATACTCTAA | 87725626 | 87725647 | 0.54 |
| GTTCCATACTCTAAAGGAAGC | 87725632 | 87725654 | 0.03 |
| GTCTGAGACATTTTTGAAAGA | 87725664 | 87725686 | 0.01 |
| GACATTTTTGAAAGATGGTT | 87725669 | 87725691 | 0.04 |
| GTTGAAAGATGGTTAGGATCC | 87725676 | 87725698 | 1.03 |
| GATGGTTAGGATCCTGGGAAC | 87725683 | 87725705 | 0.02 |
| GGCGACAACGCCAGTTCCC | 87725694 | 87725715 | 0.14 |
| GGGGTTTGGGGGGTGGGAAG | 87725714 | 87725736 | 0.03 |
| GGGCCTGGGGTTTGGGGGGT | 87725720 | 87725742 | 0.67 |
| GAAAGGAAGGGCCTGGGGTTT | 87725727 | 87725749 | 0.64 |
| GTTCCAAAAGGAAGGGCCTG | 87725733 | 87725755 | 0.11 |
| GCTTTAGGTTCCAAAAGGAA | 87725740 | 87725762 | 0.01 |
| GCCAGAAACTAGGTTGCTTT | 87725755 | 87725777 | 0.63 |
| GACTGTGACAGGCCAGAAACT | 87725765 | 87725787 | 0.01 |
| GCTGGCCTGTCACAGTTGGTC | 87725773 | 87725795 | 0.01 |
| GTCACAGTTGGTCTGGACAGA | 87725781 | 87725803 | 1.01 |

| | | | |
|---|---|---|---|
| GGTCTGGACAGATGGTTCAG | 87725789 | 87725811 | 0.04 |
| GGTGTGGGGAACTGAACTG | 87725841 | 87725862 | 0.02 |
| GTTCCCCACACCTGCATGGA | 87725851 | 87725873 | 0.00 |
| GTTGACCCTCCATGCAGGTGT | 87725855 | 87725877 | 0.01 |
| GCAGTATTGACCCTCCATGC | 87725861 | 87725883 | 0.89 |
| GTTCCAGTTCCAGGGGTCCT | 87725889 | 87725911 | 0.01 |
| GGCCAGAGGCCTAGGACCCC | 87725898 | 87725920 | 0.02 |
| GAAGTCAGAGGCCAGAGGCCT | 87725906 | 87725928 | 0.01 |
| GACTAGAAAGTCAGAGGCCAG | 87725912 | 87725934 | 9.64 |
| GCAGTACACTAGAAAGTCAG | 87725919 | 87725941 | 0.42 |
| GATTTTTTAGCTCTAATTTTA | 87726013 | 87726035 | 0.03 |
| GTAGCTCTAATTTTATGGGGT | 87726019 | 87726041 | 0.03 |
| GTCGTTTTATTCTGGTAATAC | 87726070 | 87726092 | 0.01 |
| GCTAACATACTCGTTTTATTC | 87726079 | 87726101 | 0.00 |
| GAACTCAACCCAGTGTTAACA | 87726114 | 87726136 | 0.50 |
| GACAGATTCCATGTTAACACT | 87726121 | 87726143 | 3.55 |
| GAATCTGTTAGAAATGTAAAA | 87726137 | 87726159 | 0.02 |
| GAAAAAAATCCATCTATAGCA | 87726182 | 87726204 | 2.65 |
| GATCCATCTATAGCATGGCAG | 87726188 | 87726210 | 0.01 |
| GTCTATAGCATGGCAGTGGTG | 87726193 | 87726215 | 0.01 |
| GCTCGGGAAGCAGAGTCAGGC | 87726237 | 87726259 | 0.84 |
| GCAGGTCTACAAAGTAAGTTC | 87726256 | 87726278 | 0.05 |
| GGGAGGTGGCGGGTGTTTCA | 87726296 | 87726318 | 0.00 |
| GTACATCGTGGGGGAGGTGGC | 87726306 | 87726328 | 0.01 |
| GCATCGTAGATACATCGTGG | 87726316 | 87726338 | 0.02 |
| GATTGCATCGTAGATACATCG | 87726319 | 87726341 | 0.01 |
| GTTTTTCCTTAAAAATTTTGT | 87726358 | 87726380 | 0.02 |
| GATTTTGTTGGTGGGGTTTAA | 87726371 | 87726393 | 0.01 |
| GTAAAGGTTCTAATGAAACAG | 87726388 | 87726410 | 0.00 |
| GTCTAATGAAACAGTGGGAAA | 87726395 | 87726417 | 0.52 |
| GTACTAAGTTTCCAGCTGTAC | 87726486 | 87726508 | 0.01 |
| GCCAGCTAAGCCGGTACAGC | 87726496 | 87726518 | 1.28 |
| GGCTTAGCTGGCCGGTGACT | 87726507 | 87726529 | 0.01 |
| GCTGGCCGGTGACTAGGGT | 87726513 | 87726534 | 0.01 |
| GAATATACCTACCCTAGTCAC | 87726518 | 87726540 | 0.44 |
| GTGGAACGTTGAGACTCTGA | 87726558 | 87726580 | 0.10 |
| GCACAGGATGTGCTTGGTATG | 87726577 | 87726599 | 0.54 |
| GAAGTCTCACAGGATGTGCT | 87726584 | 87726606 | 0.68 |
| GTAATACTCAAGAAGTCTCAC | 87726594 | 87726616 | 0.54 |
| GTTAAATACATCTTCTATCCT | 87726614 | 87726636 | 0.01 |
| GCTAAATGCAGAAAGACCA | 87726631 | 87726652 | 0.08 |
| GCTTTCTGCATTTAGCTTTTT | 87726638 | 87726660 | 0.01 |
| GTGTTTTTACCCTCAGCATA | 87726667 | 87726689 | 0.02 |
| GCAGCTCATACCATATGCTG | 87726677 | 87726699 | 1.23 |
| GCTGCTGATTCAGTACTGAA | 87726695 | 87726717 | 0.72 |
| GTTACATTCTGTTGATTAAAA | 87726736 | 87726758 | 0.01 |
| GAAATGGTGTGCAGAATAGTC | 87726753 | 87726775 | 0.62 |
| GCCATGTTTATTTCGGAGCAG | 87726785 | 87726807 | 0.01 |
| GCTAATTAACCATGTTTATTT | 87726793 | 87726815 | 0.01 |
| GAAACATGGTTAATTAGCTTG | 87726800 | 87726822 | 0.01 |
| GCTTGTGGAGGAGAACGAACG | 87726816 | 87726838 | 0.02 |
| GTACACAGATTTTTGTATGTT | 87726850 | 87726872 | 0.01 |

| | | | |
|---|---|---|---|
| GATTTTTGTATGTTTGGGTTA | 87726857 | 87726879 | 0.13 |
| GTATGTTTGGGTTAAGGAGAA | 87726864 | 87726886 | 2.05 |
| GTAAGGAGAAAGGGTGTGGTC | 87726875 | 87726897 | 1.45 |
| GAAAGGGTGTGGTCAGGCAAG | 87726882 | 87726904 | 0.01 |
| GAGGACAGAAATGCCCAGAA | 87726901 | 87726923 | 0.01 |
| GCACGAGCAATCTGCCATTCT | 87726914 | 87726936 | 0.03 |
| GCAGATTGCTCGTGTGAAGA | 87726923 | 87726945 | 0.02 |
| GCAGGAGCTATTCTCTCACAC | 87726955 | 87726977 | 0.01 |
| GCACAGAGGGGACATGTGGC | 87726974 | 87726996 | 0.04 |
| GAAAGCAGTGTGGGCACAGAG | 87726986 | 87727008 | 0.79 |
| GAAAATAAAATAAAGCAGTGT | 87726996 | 87727018 | 0.02 |
| GCAGCGAAAGTGTAAACTCTA | 87727059 | 87727081 | 0.03 |
| GTCAGACTCCTTCTCAGTGC | 87727098 | 87727120 | 2.21 |
| GAGAAGGAGTCTGACTTTG | 87727106 | 87727127 | 17.56 |
| GTTGCCTATGTAAATCCAGGC | 87727128 | 87727150 | 0.01 |
| GGATTTACATAGGCAATTTG | 87727135 | 87727157 | 0.01 |
| GTTTTGAGGCGGTGTAGCTCT | 87727161 | 87727183 | 0.04 |
| GTTATTTTTAGTCTTTTGAGG | 87727173 | 87727195 | 0.01 |
| GAATCTAAGAGAAGCTGTGCG | 87727199 | 87727221 | 0.01 |
| GCTCTTAGATTATGCACAGCC | 87727212 | 87727234 | 3.28 |
| GCACAGCCTGGTGCAGCCTT | 87727224 | 87727246 | 0.25 |
| GCCTGGTGCAGCCTTGGGGG | 87727229 | 87727251 | 0.05 |
| GACAAGGAGTTACCACCCCCA | 87727240 | 87727262 | 0.05 |
| GACAGTGGCACGCTGTGTACA | 87727257 | 87727279 | 0.23 |
| GGAAGGAGGAGTCGCTACAG | 87727273 | 87727295 | 4.80 |
| GCTCCTCCTTCCTCTTCTCCC | 87727285 | 87727307 | 0.02 |
| GCTTCCTCTTCTCCCAGGAGG | 87727291 | 87727313 | 0.66 |
| GACAAGACTGCCCACCTCCT | 87727302 | 87727324 | 0.01 |
| GGGCAGTCTTGTCTTGCAC | 87727312 | 87727333 | 0.01 |
| GGAGCTGGAGAAAAACACTG | 87727334 | 87727356 | 2.18 |
| GTTGCCTGAAACCAGGGAGC | 87727349 | 87727371 | 0.01 |
| GCTGAATGTTGCCTGAAACCA | 87727355 | 87727377 | 0.00 |
| GTTCAGAATATACTCCAATTC | 87727373 | 87727395 | 0.01 |
| GAAAACTGTGCTGACCGGAAT | 87727386 | 87727408 | 0.70 |
| GGTCAGCACAGTTTTACTCT | 87727394 | 87727416 | 0.03 |
| GCACAGTTTTACTCTAGGGTA | 87727400 | 87727422 | 0.01 |
| GCAGGGGGACACTTCAGAG | 87727428 | 87727449 | 0.07 |
| GTATGGCTGCTTACGTGCAGG | 87727442 | 87727464 | 1.94 |
| GTGCTCCTGGCTTTCCAATA | 87727460 | 87727482 | 0.11 |
| GATCGTACAGCTCTGTGCTCC | 87727473 | 87727495 | 0.35 |
| GCACAGAGCTGTACGATGG | 87727479 | 87727500 | 0.49 |
| GTACGATGGCGGCGCTGTT | 87727489 | 87727510 | 0.17 |
| GCTGTTAGGTAGTTCTATA | 87727502 | 87727523 | 0.02 |
| GTCCTTATTTTCTTGAGAGA | 87727527 | 87727549 | 0.02 |
| GAGAGAGGGTCTCAAGCCC | 87727541 | 87727562 | 0.01 |
| GTCAAGATACGAGGCCAGCCT | 87727557 | 87727579 | 0.02 |
| GCAAGAGGGTCTCAAGATACG | 87727567 | 87727589 | 2.54 |
| GTTAGGGAAACTGAGGCAAGA | 87727582 | 87727604 | 0.02 |
| GCCTCAGTTTCCCTAATCCT | 87727589 | 87727611 | 0.05 |
| GTTCCCTAATCCTAGGATGGT | 87727597 | 87727619 | 1.73 |
| GGGTTCCCACCTACCATCCT | 87727606 | 87727628 | 0.10 |
| GCTGGAGGAGGGGAGGCATT | 87727627 | 87727649 | 8.21 |

| | | | |
|---|---|---|---|
| GAGTAAGGCTGGAGGAGGGG | 87727634 | 87727656 | 0.04 |
| GTCTAGATTACGAGTAAGGC | 87727645 | 87727667 | 0.00 |
| GCCAGGTCTAGATTACGAGTA | 87727649 | 87727671 | 0.83 |
| GTAATCTAGACCTGGTGAC | 87727657 | 87727678 | 0.10 |
| GAACACAGTCTTCCAGTCACC | 87727667 | 87727689 | 0.04 |
| GTTGGAGTCTTTGTTCATCT | 87727687 | 87727709 | 0.99 |
| GTCTTTGTTCATCTTGGTGAG | 87727694 | 87727716 | 0.01 |
| GAAATTAGAAATTCCCTTAAA | 87727722 | 87727744 | 0.06 |
| GTTCTAAATTTAACCATTTAA | 87727734 | 87727756 | 0.03 |
| GAATGGTTAAATTTAGAAGAA | 87727740 | 87727762 | 0.02 |
| GAGAATTGCCTCAATAGTC | 87727792 | 87727813 | 0.01 |
| GCTGCAGTGACCAGACTATTG | 87727800 | 87727822 | 0.01 |
| GAAGATAACTTGTAAAACACT | 87727824 | 87727846 | 2.96 |
| GTTTTACAAGTTATCTTGGA | 87727830 | 87727852 | 0.84 |
| GGAGATAGCCAAAGCCAAGT | 87727851 | 87727873 | 0.03 |
| GAATTATTCCCCACTTGGCTT | 87727859 | 87727881 | 0.75 |
| GATTTAATTATTCCCCACT | 87727865 | 87727886 | 2.57 |
| GCAATTCAATCATTGACCTTC | 87727905 | 87727927 | 0.02 |
| GTGGGATATTTTAAGCCTGA | 87727920 | 87727942 | 0.37 |
| GAAAAAAAAAAGCATCCCGGT | 87727938 | 87727960 | 0.01 |
| GTTTTCTTTTCTTTTTGTCCC | 87727958 | 87727980 | 0.02 |
| GTCTGGGCTAAACGAAGCCC | 87727976 | 87727998 | 0.01 |
| GCACAGCAAGTTTAGTAGTCT | 87727992 | 87728014 | 0.02 |
| GCTAAACTTGCTGTGTGTAGC | 87728001 | 87728023 | 0.01 |
| GTGGGGGCAGAAAAAGTTCA | 87728030 | 87728052 | 0.05 |
| GCCCCCACCTTTCAAGTGCT | 87728045 | 87728067 | 0.02 |
| GCTATAACCCCAGCACTTGAA | 87728052 | 87728074 | 0.02 |
| GCACATAAACTGGACATGAT | 87728081 | 87728103 | 2.37 |
| GTCCAGTTTATGTGCTTCTA | 87728089 | 87728111 | 1.50 |
| GTTAAACTCTCTGTGTGTGCT | 87728113 | 87728135 | 0.18 |
| GTCGGCGTACTGCTCAGCTGT | 87728143 | 87728165 | 0.01 |
| GTTGTGTGAAAAATGGAGGCT | 87728162 | 87728184 | 0.01 |
| GAATCATTGTGTGAAAAATGG | 87728167 | 87728189 | 0.00 |
| GTTCACACAATGATTAGACTT | 87728176 | 87728198 | 0.01 |
| GGTTTTAATGTTTATTATC | 87728197 | 87728218 | 1.33 |
| GATGTTTTATTGTAAAACAC | 87728227 | 87728249 | 0.01 |
| GTTGGCATTAAGGACTCAGCA | 87728284 | 87728306 | 2.15 |
| GAGTCCTTAATGCCAAAACA | 87728291 | 87728313 | 0.08 |
| GCAAATCATGAATCCATGTTT | 87728303 | 87728325 | 0.02 |
| GATTTGCTAAACTCAGCCT | 87728320 | 87728341 | 0.01 |
| GACTTCATGAAGTAAATACCA | 87728336 | 87728358 | 0.03 |
| GATTCATCTCAGACCTCATGA | 87728360 | 87728382 | 0.00 |
| GACCTCATGAAGGTCTGAGAA | 87728371 | 87728393 | 0.01 |
| GAAGGTCTGAGAAAGGGTTG | 87728378 | 87728400 | 0.00 |
| GACAACTGCTTAGGGAGTCTT | 87728409 | 87728431 | 0.12 |
| GTAACTAAGACAACTGCTTA | 87728418 | 87728440 | 0.02 |
| GTTGGGTCATGGTGTTTGTCA | 87728445 | 87728467 | 0.01 |
| GTCTCTAAGTTCCTTGGGTCA | 87728457 | 87728479 | 0.01 |
| GTTTCCTTCTCTAAGTTCCTT | 87728463 | 87728485 | 0.01 |
| GAGAAGGAAAGATTTCATTG | 87728476 | 87728498 | 0.03 |
| GCGTGCGTGACTATCATGGC | 87728520 | 87728542 | 0.01 |
| GATCATGGCAGGAAGCACTGC | 87728532 | 87728554 | 0.01 |

| | | | |
|---|---|---|---|
| GCAGGAAGCACTGCAGGAGC | 87728538 | 87728560 | 0.01 |
| GAGCAGTAGCTGAGAGCACA | 87728575 | 87728597 | 1.00 |
| GTTGCTTTTTTCTTTTGAAAC | 87728605 | 87728627 | 0.04 |
| GCTATGTAGTTCTCATTGTTC | 87728634 | 87728656 | 0.02 |
| GTTGAGTTTGAGGTCAGTC | 87728670 | 87728691 | 0.00 |
| GTGAGTTCTCAGCTGAGTG | 87728876 | 87728897 | 0.01 |
| GCATCCTCATTTACAAACATG | 87728897 | 87728919 | 0.01 |
| GATGAGGCAGAAAGATGTAGC | 87728914 | 87728936 | 0.01 |
| GCAGAAAGATGTAGCTGGGAA | 87728920 | 87728942 | 3.57 |
| GTCACTAGGGACAGTCTTGG | 87728958 | 87728980 | 0.01 |
| GTTGGAGATGTTGTGTCACTA | 87728971 | 87728993 | 0.01 |
| GATTAGGAAGTATTTCTTTAT | 87728990 | 87729012 | 0.02 |
| GAAATACTTGGTCCCAGTTGA | 87729028 | 87729050 | 0.74 |
| GGCTTCTGTATTTAAATACT | 87729041 | 87729063 | 0.06 |
| GATTTAAATACAGAAGCCTCT | 87729047 | 87729069 | 0.03 |
| GCTAATGAGAATGGCCCCTAG | 87729062 | 87729084 | 0.03 |
| GTGGTCATACTAATGAGAA | 87729072 | 87729093 | 0.47 |
| GTATGACCACATTCTCTCCC | 87729084 | 87729106 | 10.17 |
| GTAGGGGCCAGGGAGAGAATG | 87729090 | 87729112 | 0.03 |
| GCAGCAAACCTATAGGGGCC | 87729102 | 87729124 | 0.00 |
| GATAAGGCAGCAAACCTATAG | 87729107 | 87729129 | 0.09 |
| GATGCATTTGTATTATAATA | 87729124 | 87729146 | 1.30 |
| GAGTTGAGACAGAAAAGACA | 87729166 | 87729188 | 0.27 |
| GAGACTTTGGAATTCTTAAG | 87729188 | 87729210 | 0.00 |
| GTTGGGTCTTAGGAGAGACTT | 87729201 | 87729223 | 1.89 |
| GAAGAGACGGCCTTGGGTCTT | 87729212 | 87729234 | 0.01 |
| GTACAAGTAAGAGACGGCCTT | 87729219 | 87729241 | 0.01 |
| GAGGGTTACAAGTAAGAGA | 87729226 | 87729247 | 0.01 |
| GTTGCTCTTTTGATTTTTCAG | 87729244 | 87729266 | 0.01 |
| GTATACACTTCCAATATACAA | 87729269 | 87729291 | 0.02 |
| GTTTCAAAAGAGAAGAAGTAT | 87729311 | 87729333 | 0.01 |
| GAAGAGAAGAAGTATAGGACA | 87729317 | 87729339 | 0.00 |
| GACATGGTGAAGAAATTAAC | 87729333 | 87729355 | 0.02 |
| GTTAACTGGACCAAACACAGC | 87729348 | 87729370 | 2.90 |
| GAGTTTGCCCTGCTGTGTT | 87729357 | 87729378 | 0.32 |
| GACATCAGATACGGAACTAC | 87729384 | 87729406 | 0.02 |
| GAAGCTCTCTGACATCAGATA | 87729393 | 87729415 | 0.01 |
| GCTGATGTCAGAGAGCTTAGA | 87729399 | 87729421 | 0.02 |
| GAAGGCTCTGCCTTTCCAGCT | 87729418 | 87729440 | 0.08 |
| GTAGTCAACCAAGCTGGAA | 87729427 | 87729448 | 0.01 |
| GTGCTGTAGTCAACCAAGC | 87729432 | 87729453 | 0.10 |
| GCACACTTCTCTCTTGAGA | 87729450 | 87729471 | 0.01 |
| GGAGTGTGGCATGTGGGCAG | 87729474 | 87729496 | 0.00 |
| GCAGCCAGGGAGTGTGGCATG | 87729481 | 87729503 | 0.00 |
| GGGATATCAGCCAGGGAGTG | 87729488 | 87729510 | 0.01 |
| GAGCCATGGGATATCAGCCA | 87729495 | 87729517 | 2.95 |
| GTTGGAGGTGTCAGAGCCAT | 87729508 | 87729530 | 0.03 |
| GCTCTGACACCTCCAACACCC | 87729515 | 87729537 | 0.26 |
| GTGTTGGCACCCCAGGGTGT | 87729526 | 87729548 | 0.01 |
| GAAGGATTGTGTTGGCACCCC | 87729533 | 87729555 | 0.01 |
| GATGAATGTCAAGGATTGTGT | 87729542 | 87729564 | 0.01 |
| GTCATGATTCTATGAATGTCA | 87729552 | 87729574 | 0.02 |

| | | | |
|---|---|---|---|
| GACATTCATAGAATCATGAGA | 87729557 | 87729579 | 0.05 |
| GCCTCCCAGAGCCTCCATGT | 87729579 | 87729601 | 0.00 |
| GAGTCCCTACATGGAGGCTC | 87729584 | 87729606 | 0.14 |
| GCAAGGGAGTCCCTACATGG | 87729590 | 87729612 | 0.01 |
| GGACTCCCTTGCCACATGTC | 87729601 | 87729623 | 0.54 |
| GATCATTAAGGGGAGCCACTG | 87729624 | 87729646 | 0.00 |
| GTGGCTCCCCTTAATGATAG | 87729629 | 87729651 | 0.01 |
| GAATCTTCCTCTATCATTAA | 87729636 | 87729658 | 0.79 |
| GTTCTGGCTTTAAAGTCATAA | 87729672 | 87729694 | 0.01 |
| GACTTTAAAGCCAGAACCACG | 87729680 | 87729702 | 0.02 |
| GCAGTAATGCCACGTGGTTC | 87729689 | 87729711 | 0.02 |
| GAATATGGCAGTAATGCCACG | 87729695 | 87729717 | 0.02 |
| GCCATATTTGGCTGCCTACT | 87729710 | 87729732 | 0.01 |
| GATTTGGCTGCCTACTGGGGA | 87729715 | 87729737 | 0.04 |
| GTGAGGTTCCATCCCCAGT | 87729724 | 87729745 | 2.51 |
| GATGTAACTTAGGGAGGAGTG | 87729740 | 87729762 | 0.14 |
| GCTTAAGCCAATGTAACTTA | 87729750 | 87729772 | 0.16 |
| GAAGCTTCCCATGAATTCCTT | 87729769 | 87729791 | 0.02 |
| GAAAACGCCTAAGGAATTCA | 87729776 | 87729798 | 0.02 |
| GCTTGTAAAAGAAAACGCCTA | 87729785 | 87729807 | 0.22 |
| GAAGTAGGATGCTTAGCTGTG | 87729805 | 87729827 | 0.22 |
| GCTGTGTGGCCTCTTCCCTG | 87729819 | 87729841 | 0.62 |
| GAGTGGAGCCCTCAGGGAAG | 87729828 | 87729850 | 0.01 |
| GAGAATGGAGTGGAGCCCTC | 87729835 | 87729857 | 0.01 |
| GCAGAGGAAGAGAATGGAG | 87729845 | 87729866 | 0.01 |
| GGGACTGCAGAGGAAGAGAA | 87729850 | 87729872 | 0.00 |
| GAAGTAGACAAGGGACTGCAG | 87729860 | 87729882 | 0.33 |
| GCTGAAGCACTGAAGTAGACA | 87729871 | 87729893 | 0.39 |
| GAAATATACAGTTGGAGGAGA | 87729909 | 87729931 | 0.02 |
| GCAAGTAAAATATACAGTTGG | 87729915 | 87729937 | 0.02 |
| GTCTACAGTGAAAAAGTAGG | 87729946 | 87729968 | 0.29 |
| GCTTGTGAACACTGCTATGA | 87729968 | 87729990 | 0.01 |
| GCAAGTGACAAAGTCAATATT | 87729990 | 87730012 | 0.01 |
| GTTAATCCAAAACTCCAATTT | 87730042 | 87730064 | 2.02 |
| GCTGAGCCTAAATTGGAGTTT | 87730047 | 87730069 | 0.01 |
| GAATCTTCCTGAGCCTAAAT | 87730055 | 87730077 | 0.02 |
| GTTAGGCTCAGGAAGATTCTT | 87730060 | 87730082 | 0.95 |
| GTCAGGAAGATTCTTAGGACG | 87730066 | 87730088 | 0.83 |
| GATTTTGGCAAAGTATGTGGC | 87730115 | 87730137 | 0.01 |
| GATACTTTGCCAAAATATCAT | 87730123 | 87730145 | 0.04 |
| GTTTCAGAGGGTAACTATCA | 87730158 | 87730180 | 0.00 |
| GCCTGGATCGAGGTTTCAGA | 87730170 | 87730192 | 0.01 |
| GACTATGGAGGCCTGGATCG | 87730180 | 87730202 | 0.01 |
| GCAGCGTGGACTATGGAGGCC | 87730187 | 87730209 | 0.02 |
| GAGACCAGCGTGGACTATGG | 87730192 | 87730214 | 0.17 |
| GCAGACGTGTTGAGACCAGCG | 87730202 | 87730224 | 0.24 |
| GGTCATCACAATAGGAGTT | 87730229 | 87730250 | 0.00 |
| GTTTAACAGGTCATCACAAT | 87730236 | 87730258 | 2.65 |
| GCTCAAAGTGGAGTTTAAC | 87730249 | 87730270 | 0.01 |
| GAAGCAATTGAATGCTCAAAG | 87730260 | 87730282 | 0.07 |
| GAGCATTCAATTGCTTTCC | 87730267 | 87730288 | 0.06 |
| GTCAATTGCTTTCCTGGTTCA | 87730273 | 87730295 | 0.24 |

| | | | |
|---|---|---|---|
| GACTTTGGGACCTTGAACC | 87730284 | 87730305 | 0.40 |
| GCTGGGATTAAAGGATGACTT | 87730298 | 87730320 | 0.01 |
| GTCTCTGGCTCTCAAGTGCT | 87730316 | 87730338 | 0.04 |
| GACTTGAGAGCCAGAGACAGG | 87730322 | 87730344 | 0.05 |
| GTAAAGAGATCCACCTGTCTC | 87730331 | 87730353 | 0.00 |
| GCTCTTTAAGTTTGAGGCAGC | 87730347 | 87730369 | 0.06 |
| GGTCTGAAGAGTGAGTTTCC | 87730368 | 87730390 | 0.06 |
| GCTATGTAACCATAGCTGTCC | 87730386 | 87730408 | 0.01 |
| GACATCAATAGCCCTACCTCC | 87730494 | 87730516 | 0.55 |
| GGAGTTGGTACCAGGAGGTA | 87730504 | 87730526 | 0.83 |
| GCTAAGACTGGAGTTGGTACC | 87730512 | 87730534 | 0.02 |
| GAAAGTAACTAAGACTGGAGT | 87730519 | 87730541 | 0.01 |
| GTATGGAAAAGTAACTAAGAC | 87730525 | 87730547 | 0.21 |
| GTGATGTTTTGTCACAGCTA | 87730543 | 87730565 | 0.01 |
| GTGACAAAACATCACACCA | 87730551 | 87730572 | 0.90 |
| GCGTCATGTATAAATTGCCT | 87730567 | 87730589 | 0.01 |
| GATACATGACGCTGTTTAATT | 87730579 | 87730601 | 0.02 |
| GTTTGGGGCTCTCAGTCCCAG | 87730597 | 87730619 | 0.01 |
| GTCATGGATTCTAACCCTCT | 87730612 | 87730634 | 0.05 |
| GGTTAGAATCCATGACAGTC | 87730619 | 87730641 | 0.01 |
| GATCATGCTCCCAGACTGTCA | 87730628 | 87730650 | 0.00 |
| GCATGATAGCAGACAGACA | 87730644 | 87730665 | 0.01 |
| GTAGCTAAGAGTCCACATGC | 87730676 | 87730698 | 0.02 |
| GCAGGAAGCAGAAAGCTAAG | 87730694 | 87730716 | 0.01 |
| GCAGAAAGCTAAGTGGGAA | 87730701 | 87730722 | 0.01 |
| GACTATCAATGTCAACACTTC | 87730723 | 87730745 | 0.07 |
| GAATATAATGTTAGCAGCGTT | 87730749 | 87730771 | 0.02 |
| GCGTTTGGAGCAAGACACTG | 87730764 | 87730786 | 1.30 |
| GGAGCAAGACACTGGGGGGT | 87730770 | 87730792 | 0.05 |
| GCAGGGGGTGCCTCAAATCTT | 87730800 | 87730822 | 0.02 |
| GCCAAGAGACCCTAAGATTTG | 87730809 | 87730831 | 0.15 |
| GTAGCTTGTGGAAGAACATGC | 87730836 | 87730858 | 0.02 |
| GTGAAAGCATTCTTAGCTTG | 87730849 | 87730871 | 9.59 |
| GAATGCTTTCACTAGGCTGG | 87730860 | 87730882 | 0.17 |
| GTAGGCTGGGGGGAGTAGCCC | 87730872 | 87730894 | 0.01 |
| GAGTAATAAGGAGAAGCCCA | 87730889 | 87730911 | 1.10 |
| GATATACTCAACAGAGTAATA | 87730901 | 87730923 | 0.00 |
| GCAACTGCTGACTGAGCCCCA | 87730925 | 87730947 | 1.03 |
| GCTGACTGAGCCCCATGGGCC | 87730931 | 87730953 | 0.02 |
| GCTGCTGTGTCCAGGCCCATG | 87730940 | 87730962 | 0.04 |
| GTCTTTACTCCTGCTGTGTCC | 87730949 | 87730971 | 0.83 |
| GCAGCAGTGTAGCCTACTCTG | 87730975 | 87730997 | 2.75 |
| GCCTACTCTGAGGACTGACT | 87730985 | 87731007 | 0.01 |
| GACAGAGACGAGCAGGACTG | 87731009 | 87731031 | 0.01 |
| GTTAAAGTGACAGAGACGAGC | 87731016 | 87731038 | 1.94 |
| GTCGTCTCTGTCACTTTAAAC | 87731021 | 87731043 | 0.08 |
| GGAGGAAACTGAGGAGGGGA | 87731045 | 87731067 | 0.04 |
| GCAGAAGAGGGGAGGAAACTG | 87731054 | 87731076 | 0.40 |
| GCTTAGGTTACAGAAGAGGGG | 87731063 | 87731085 | 0.01 |
| GAACATCCACTCTAATATCTT | 87731080 | 87731102 | 1.80 |
| GTTCTCAGATGCCATTCCATC | 87731107 | 87731129 | 0.15 |
| GCTCACTGGGCCCTGATGGAA | 87731117 | 87731139 | 0.01 |

| | | | |
|---|---|---|---|
| GATTTCCTCACTGGGCCCTGA | 87731122 | 87731144 | 0.13 |
| GTTCTGAACCATTTCCTCACT | 87731131 | 87731153 | 0.08 |
| GAGGAAATGGTTCAGAAAGC | 87731137 | 87731159 | 0.04 |
| GTCTGAATGCTCTTAGGGTCA | 87731173 | 87731195 | 0.01 |
| GTGCATCTCTGAATGCTCTT | 87731180 | 87731202 | 0.11 |
| GTCAGAGATGCACCGCTAGTT | 87731191 | 87731213 | 0.04 |
| GCACCGCTAGTTAGGGTTGA | 87731199 | 87731221 | 0.32 |
| GTTAGGGTTGAGGGTATAGAT | 87731209 | 87731231 | 0.01 |
| GTTGAGGGTATAGATAGGGCC | 87731215 | 87731237 | 0.01 |
| GTATAGATAGGGCCAGGGATA | 87731222 | 87731244 | 0.30 |
| GCTCATTCGAGGCCATATCCC | 87731233 | 87731255 | 0.03 |
| GAGCTGAAAAACTCATTCG | 87731245 | 87731266 | 0.01 |
| GTTGTTTTGCTATGCAGAGCT | 87731271 | 87731293 | 0.01 |
| GACATGTTTTCTTGGTTACT | 87731302 | 87731324 | 0.01 |
| GGACAAAGACATGTTTTCT | 87731310 | 87731331 | 0.02 |
| GTCCTAGTGATAAAGACAGCT | 87731329 | 87731351 | 0.01 |
| GAAAAAAATTAAGAATTCATA | 87731408 | 87731430 | 0.02 |
| GAACCACAAAAAGTCTCAAA | 87731430 | 87731452 | 0.01 |
| GAAATGGCCAAAGCAACACTG | 87731447 | 87731469 | 2.20 |
| GCTTTCTCCACAGTGTTGCTT | 87731453 | 87731475 | 0.01 |
| GAGAAAGATCATCTTTGCTG | 87731469 | 87731491 | 0.03 |
| GTTTGCTGAGGGTCTTCACGC | 87731482 | 87731504 | 11.10 |
| GCTCTAGTGATAAAGACAGCT | 87731526 | 87731548 | 0.01 |
| GAAATAAACCCATGAAGCTA | 87731600 | 87731622 | 0.02 |
| GTCAAGCGGCCATAGCTTCAT | 87731608 | 87731630 | 2.37 |
| GGCCGCTTGATGCTTGACAA | 87731621 | 87731643 | 0.01 |
| GCCAGTGTTTTTGTTTTGGTT | 87731656 | 87731678 | 0.01 |
| GCTTTCCCAGTGTTTTTGTTT | 87731661 | 87731683 | 0.01 |
| GCCTATTCTATAAATAGTGC | 87731684 | 87731706 | 0.03 |
| GTCTATAAATAGTGCTGGGGC | 87731690 | 87731712 | 0.03 |
| GTAGTGCTGGGGCTGGTGAGA | 87731698 | 87731720 | 0.05 |
| GAATAAATAAATAAATAGTGC | 87731897 | 87731919 | 0.04 |
| GCAAAACTAAATATCCACCTG | 87731920 | 87731942 | 0.01 |
| GTATCCACCTGAGGGAGAATG | 87731930 | 87731952 | 0.02 |
| GATGTAACCTCATTCTCCCTC | 87731936 | 87731958 | 3.31 |
| GAATCGATTATTCTATAGGA | 87731968 | 87731990 | 0.01 |
| GATAGAATAATCGATTCTCAG | 87731975 | 87731997 | 0.31 |
| GGGTTTTTAATTGGGGTCTT | 87731999 | 87732021 | 0.03 |
| GCTGTTTCGGGTTTTTAATTG | 87732006 | 87732028 | 0.02 |
| GTTATTTATTTTTACTGTTTC | 87732019 | 87732041 | 0.10 |
| GTGCCAGTATTAGCCAAGGC | 87732058 | 87732080 | 0.01 |
| GTCACCTGCCTTGGCTAATAC | 87732061 | 87732083 | 0.01 |
| GCCTGGGGAAATCACCTGCCT | 87732071 | 87732093 | 0.01 |
| GTTTGCACTTAACTGTCCCTG | 87732087 | 87732109 | 0.01 |
| GACAGTTAAGTGCAAAGACC | 87732094 | 87732116 | 0.02 |
| GCAAAGACCCGGTGAGAGCTC | 87732106 | 87732128 | 0.80 |
| GCCAGGCCCTGAGCTCTCACC | 87732112 | 87732134 | 0.02 |
| GGCCAGACTCGCACAGCTGC | 87732134 | 87732156 | 1.55 |
| GCACAGCTGCAGGGCTGCTCC | 87732145 | 87732167 | 2.78 |
| GCATGAAGACTAAGAGGGACC | 87732163 | 87732185 | 8.02 |
| GCTGATGCATGAAGACTAAGA | 87732169 | 87732191 | 0.02 |
| GGGGCATGCTGCACAGCAC | 87732200 | 87732221 | 0.86 |

| | | | |
|---|---|---|---|
| GCAGCATGCCCCTCCTCACTC | 87732211 | 87732233 | 0.01 |
| GTTTGTTGCCAGAGTGAGGAG | 87732218 | 87732240 | 0.01 |
| GCACATTTTGTTGCCAGAGTG | 87732223 | 87732245 | 0.00 |
| GCTGCACATTAGCTAGTAAC | 87732266 | 87732288 | 0.00 |
| GGTTCCTGTCCCCTCTTCTC | 87732287 | 87732309 | 16.95 |
| GTTCAGTCAACCTGAGAAGAG | 87732296 | 87732318 | 1.57 |
| GTCTCAGGTTGACTGAAGAGA | 87732303 | 87732325 | 0.18 |
| GGTTGACTGAAGAGATGGGG | 87732308 | 87732330 | 0.01 |
| GAGATGGGGAGGACTCCCAA | 87732319 | 87732341 | 0.01 |
| GTAGGCAGTAGACTCACCCTT | 87732334 | 87732356 | 0.07 |
| GTCTACTGCCTAGCCCTTTTC | 87732346 | 87732368 | 0.08 |
| GCAAGAAGCCAGAAAAGGGCT | 87732353 | 87732375 | 0.01 |
| GGCAGCAAGAAGCCAGAAAA | 87732358 | 87732380 | 5.20 |
| GCTGCCAAGCTGCTGTGTGC | 87732375 | 87732397 | 1.03 |
| GCTGCTGTGTGCAGGGAACAC | 87732384 | 87732406 | 0.06 |
| GTACATTAAAGATGGTCTTC | 87732409 | 87732431 | 0.24 |
| GATGATGGGTACATTAAAGA | 87732417 | 87732439 | 2.49 |
| GAATGATCTAAATAAGATGAT | 87732431 | 87732453 | 0.40 |
| GTTTCTAGATTCCTCATGAA | 87732460 | 87732482 | 0.02 |
| GTTTTAACTGGACCTTTCATG | 87732471 | 87732493 | 0.01 |
| GAAAGGTCCAGTTAAAATCT | 87732477 | 87732499 | 0.01 |
| GACTTCTTCCAAGATTTTAAC | 87732484 | 87732506 | 0.01 |
| GTCTCTGTACATCGTCCTCAG | 87732511 | 87732533 | 0.01 |
| GTCAGAGGAGTGACAAAACCC | 87732527 | 87732549 | 0.01 |
| GAGTGACAAAACCCTGGCTA | 87732533 | 87732555 | 0.52 |
| GCTTTGATTTCTCCATAGCCA | 87732544 | 87732566 | 0.01 |
| GCTATGGAGAAATCAAAGGAA | 87732550 | 87732572 | 0.03 |
| GAAAGGCAGAGCTGTTGCCC | 87732567 | 87732589 | 0.01 |
| GCAGAGCTGTTGCCCAGGTTA | 87732573 | 87732595 | 0.03 |
| GCATTCTAGTTACCTTAACCT | 87732584 | 87732606 | 0.02 |
| GTTAAGGTAACTAGAATGTG | 87732589 | 87732611 | 0.07 |
| GAGGCATCTTCTTGTATGA | 87732608 | 87732629 | 0.24 |
| GAACGTTCTAATGTTTTCATC | 87732635 | 87732657 | 0.02 |
| GTTCATCAGGGAGAGAGAAAT | 87732649 | 87732671 | 0.00 |
| GAGACTCTAGCACTTGTAT | 87732675 | 87732696 | 1.19 |
| GTTGGAGTAAAACATAAAATG | 87732693 | 87732715 | 0.09 |
| GTAAAACATAAAATGAGGCC | 87732698 | 87732720 | 0.05 |
| GGATGGGATAAATACTCCC | 87732716 | 87732737 | 0.02 |
| GTTCTAAATACTGAGAGGAT | 87732731 | 87732753 | 0.06 |
| GAGAAGTTCTAAATACTGAG | 87732736 | 87732758 | 0.01 |
| GTCATCCTTTAAGCAGCCTC | 87732782 | 87732804 | 0.01 |
| GGGATCCGGAGGCTGCTTAA | 87732787 | 87732809 | 0.04 |
| GAAGCAGCCTCCGGATCCCTT | 87732792 | 87732814 | 0.06 |
| GACATGTCCTAAGGGATCCGG | 87732798 | 87732820 | 0.12 |
| GCTGGACGTGAACATGTCCTA | 87732808 | 87732830 | 0.04 |
| GCTGGATATGAACAATGTGTC | 87732827 | 87732849 | 0.02 |
| GCACATTGTTCATATCCAGGC | 87732832 | 87732854 | 0.01 |
| GCAACAGCTTAATGTCCAGCC | 87732846 | 87732868 | 0.02 |
| GAACCTGAAAATACAATCTAG | 87732869 | 87732891 | 0.00 |
| GTATTTTCAGGTTGCAATGG | 87732879 | 87732901 | 0.14 |
| GTTGCAATGGTGGGAATAAC | 87732889 | 87732911 | 0.01 |
| GAACAGGTTCTCAGTTTTTAT | 87732906 | 87732928 | 0.03 |

| | | | |
|---|---|---|---|
| GCTCAGTTTTTATAGGTGGTC | 87732914 | 87732936 | 0.01 |
| GATAGGTGGTCAGGGCTCAGC | 87732924 | 87732946 | 0.28 |
| GATTCTCACACTTGCACAGC | 87732955 | 87732977 | 0.01 |
| GCTGGCTTTTATAGTGTTCTG | 87732989 | 87733011 | 3.33 |
| GCAGAAGCATAGCAAGAATAT | 87733009 | 87733031 | 0.01 |
| GTAGCAAGAATATCGGATCCC | 87733017 | 87733039 | 0.02 |
| GATCGGATCCCAGGGCTCCTG | 87733027 | 87733049 | 0.01 |
| GGGCTCCTGTGGGAGATGAG | 87733038 | 87733060 | 0.03 |
| GCTCCTGTGGGAGATGAGAGG | 87733041 | 87733063 | 0.11 |
| GGGAGATGAGAGGTGGAGGC | 87733048 | 87733070 | 0.03 |
| GAATTCCTGTAAATTTGTA | 87733072 | 87733093 | 0.01 |
| GTATATGCCATACAAATTTAC | 87733077 | 87733099 | 0.01 |
| GAATTTGTATGGCATATAAAA | 87733083 | 87733105 | 0.00 |
| GCAGCAAAGAGACTCTGTCTC | 87733110 | 87733132 | 0.01 |
| GCTGTCTCAGGGCAGAAAGCA | 87733123 | 87733145 | 0.66 |
| GAAAGCAAGGGCTGACTTCCA | 87733137 | 87733159 | 0.01 |
| GGTGGGAGGTCAGAGGACCT | 87733154 | 87733176 | 0.04 |
| GTACACAGGTGGGAGGTCAG | 87733161 | 87733183 | 0.01 |
| GTGCACACATGGTACACAGG | 87733172 | 87733194 | 0.35 |
| GCACGTGCACACATGGTACAC | 87733175 | 87733197 | 0.01 |
| GTATACATGCACGTGCACACA | 87733183 | 87733205 | 0.07 |
| GAGGATGTGATATAATAATG | 87733286 | 87733308 | 0.07 |
| GTAATAATGTGGATGGAGAAA | 87733298 | 87733320 | 0.01 |
| GGGTAGATGAGTGAATGGA | 87733323 | 87733344 | 0.03 |
| GAATGGATGGATCAGTGAA | 87733335 | 87733356 | 0.01 |
| GTCAGTGAATGGATGGATGAG | 87733346 | 87733368 | 0.01 |
| GATGAGTGGATGGATGGGGCA | 87733361 | 87733383 | 0.01 |
| GATGGATGGGGCATGGATGAG | 87733369 | 87733391 | 0.01 |
| GGCATGGATGAGTGGGTAGA | 87733377 | 87733399 | 0.06 |
| GTAGATGGGTTAACTAGTTC | 87733392 | 87733414 | 1.56 |
| GTTAACTAGTTCTGGATAATC | 87733401 | 87733423 | 1.87 |
| GTAGTTCTGGATAATCAGGTT | 87733406 | 87733428 | 0.01 |
| GCACTTTATAAACTTCATTTT | 87733429 | 87733451 | 0.01 |
| GGTCCTCACATAGAAACAGT | 87733453 | 87733475 | 0.16 |
| GTTCGAGATGTTAAAACAGCA | 87733494 | 87733516 | 0.01 |
| GATGTTAAAACAGCAAGGCA | 87733499 | 87733521 | 0.01 |
| GCATGCCTTTATTCCCAGAAT | 87733528 | 87733550 | 0.06 |
| GAACTCACTGCAGATCAAGC | 87733586 | 87733608 | 0.02 |
| GAATCTGGGTTTCTCTGCG | 87733623 | 87733644 | 0.00 |
| GCTTAAGTTCTCTAAGAATCT | 87733636 | 87733658 | 0.17 |
| GCTTAGAGAACTTAAGGAGTT | 87733644 | 87733666 | 0.12 |
| GAACTTAAGGAGTTTGGTAGT | 87733651 | 87733673 | 0.01 |
| GTTGGTAGTAGGAGTGTCATC | 87733663 | 87733685 | 0.01 |
| GAAACAGCCACTACTGTTCGA | 87733700 | 87733722 | 0.11 |
| GCCACTACTGTTCGAAGGTC | 87733705 | 87733727 | 0.01 |
| GTCAGGCTGATACCAAGTTGA | 87733723 | 87733745 | 0.01 |
| GGTAAAATCTACCTTCAACT | 87733734 | 87733756 | 0.01 |
| GTAGATTTTACCCAGAAGTCT | 87733746 | 87733768 | 0.59 |
| GTTACCCAGAAGTCTTGGCTC | 87733752 | 87733774 | 0.71 |
| GAAGTCTTGGCTCAGGTGT | 87733759 | 87733780 | 0.02 |
| GCTCAGGTGTAGGACTGTAG | 87733768 | 87733790 | 0.01 |
| GTGTAGGACTGTAGAGGAAC | 87733774 | 87733796 | 0.01 |

| | | | |
|---|---|---|---|
| GGAACAGGCTCAGCCACACT | 87733789 | 87733811 | 0.05 |
| GCCACACTGGGGGGCAGTGG | 87733801 | 87733823 | 0.00 |
| GGGGGGCAGTGGTGGGGATT | 87733809 | 87733831 | 0.02 |
| GTAGGGACAGTCAGCTTCAGC | 87733828 | 87733850 | 3.78 |
| GCAGTCAGCTTCAGCAGGAAT | 87733834 | 87733856 | 0.08 |
| GACTACTCTCACACTGAAA | 87733875 | 87733896 | 0.32 |
| GATTGTAGAAAGCTCTTCTGA | 87733915 | 87733937 | 0.38 |
| GAATGCTGTGTACTGAGTCCC | 87733935 | 87733957 | 0.11 |
| GCTGGGGGACAGGTGGCATCC | 87733953 | 87733975 | 0.00 |
| GGGACGTACTGGGGGACAGG | 87733961 | 87733983 | 0.11 |
| GTCCCCCAGTACGTCCCCAT | 87733967 | 87733989 | 0.03 |
| GTACGTCCCCATGGGCTGC | 87733975 | 87733996 | 0.11 |
| GGTGTAGCCTGCAGCCCATG | 87733981 | 87734003 | 0.03 |
| GACACTCTGGCATACGCGAGA | 87734002 | 87734024 | 0.46 |
| GTTCTCTCAGTACTTACACTC | 87734016 | 87734038 | 0.01 |
| GTACTGAGAGAAGGTGTCACT | 87734028 | 87734050 | 0.01 |
| GTGTCACTTGGGTGTGTAGC | 87734040 | 87734062 | 0.02 |
| GCTGGTGGCCATAGAAATTT | 87734058 | 87734080 | 0.13 |
| GATGCAGGCCCCAAATTTCTA | 87734066 | 87734088 | 2.21 |
| GTTGGGGCCTGCATGTGAGTC | 87734076 | 87734098 | 0.00 |
| GCATGTGAGTCTGGTGCTGG | 87734085 | 87734107 | 0.01 |
| GTGAGTCTGGTGCTGGAGG | 87734089 | 87734110 | 0.13 |
| GGTGCTGGAGGCGGCATCTT | 87734097 | 87734119 | 0.01 |
| GGCGGCATCTTTGGTACCT | 87734106 | 87734127 | 1.77 |
| GTAGAAAGATGGTTGGAACCA | 87734122 | 87734144 | 0.01 |
| GGCTGATGTAGAAAGATGGT | 87734130 | 87734152 | 0.02 |
| GCTGTTGAGAGAGGAGCTCA | 87734151 | 87734173 | 0.22 |
| GCTCAGTTAGGCTGTTGAGAG | 87734160 | 87734182 | 0.16 |
| GCCTAACTGAGGTGACGCTG | 87734172 | 87734194 | 0.00 |
| GACTGAGGTGACGCTGCGGGT | 87734177 | 87734199 | 0.00 |
| GTAGGGCAAGCATGCACACCC | 87734196 | 87734218 | 0.01 |
| GCATGCACACCCTGGCCCAA | 87734204 | 87734226 | 0.03 |
| GTGGCTTGTCCCTTGGGCCA | 87734213 | 87734235 | 1.08 |
| GCTAACAGTGGCTTGTCCCTT | 87734219 | 87734241 | 2.78 |
| GATAAAAACAGAGACTAACAG | 87734232 | 87734254 | 0.02 |
| GTCTCTGTTTTTATTGTGAC | 87734241 | 87734263 | 0.01 |
| GACTGAAGATCATTTTGCCTT | 87734275 | 87734297 | 0.01 |
| GGTGGGTACTGAGGACCCTA | 87734291 | 87734313 | 0.01 |
| GTAGTGAAGGGTGGGTACTG | 87734300 | 87734322 | 0.05 |
| GAAATCACAGTAGTGAAGGG | 87734309 | 87734331 | 0.01 |
| GATTTCTGTTTATGTTCGCC | 87734326 | 87734348 | 0.02 |
| GATGTTCGCCAGGTATTAGTG | 87734337 | 87734359 | 0.27 |
| GAGTAGACCTCACTAATACC | 87734344 | 87734366 | 0.01 |
| GTGTGGTCTGTGTGTGTGTT | 87734400 | 87734422 | 0.09 |
| GCTTACTCATATGACAGCAC | 87734424 | 87734446 | 0.01 |
| GAGTTATAGAGAGACACGAG | 87734454 | 87734476 | 0.01 |
| GTGTCTCTCTATAACTCCA | 87734460 | 87734481 | 0.01 |
| GTCCAAGGTCTGTGACAGCTG | 87734475 | 87734497 | 0.00 |
| GTGACAGCTGTGGAGGGAGG | 87734485 | 87734507 | 0.01 |
| GATTGCACACAATCGTGTTTC | 87734513 | 87734535 | 0.07 |
| GCTTCCACTGATGTAAAGTCA | 87734544 | 87734566 | 0.02 |
| GCACGGGGTGACCAGCCACTC | 87734562 | 87734584 | 0.03 |

| | | | |
|---|---|---|---|
| GTGACCAGCCACTCCGGTTG | 87734568 | 87734590 | 0.01 |
| GCCACTCCGGTTGTGGGGTA | 87734575 | 87734597 | 0.06 |
| GACACATCCGTACCCCACAAC | 87734581 | 87734603 | 0.23 |
| GGGGTACGGATGTGTACTTA | 87734589 | 87734611 | 0.20 |
| GACAGTGCCTAAGACTCTCCA | 87734625 | 87734647 | 1.43 |
| GCTTGTCCCATGGAGAGTCTT | 87734631 | 87734653 | 0.01 |
| GCTTCTTGTCCCTTGTCCCA | 87734642 | 87734664 | 1.11 |
| GACAAGGGACAAGAAGCCAGT | 87734649 | 87734671 | 0.07 |
| GAAGCCAGTTGGAGGTAGAG | 87734660 | 87734682 | 0.02 |
| GAGGTAGAGGGGACCACACT | 87734671 | 87734693 | 0.01 |
| GGGGACCACACTAGGACGTG | 87734679 | 87734701 | 0.16 |
| GCTAGGACGTGAGGTTGGAGC | 87734689 | 87734711 | 1.44 |
| GATATCTACTTCAGCCCCTC | 87734711 | 87734733 | 0.18 |
| GACTTCAGCCCCTCTGGCCCA | 87734718 | 87734740 | 0.26 |
| GGGGCGACCTTGGGCCAGAG | 87734725 | 87734747 | 0.00 |
| GCTGAACTCAGGGGCGACCTT | 87734734 | 87734756 | 0.01 |
| GACGGAAAGCCTGAACTCAG | 87734744 | 87734766 | 1.40 |
| GTTCAGGCTTTCCGTCCCTA | 87734751 | 87734773 | 0.32 |
| GTACAGAGAGACCCCTAGGGA | 87734762 | 87734784 | 3.63 |
| GTTAGTACAGAGAGACCCCT | 87734767 | 87734789 | 0.01 |
| GACTAACGCTGTGACAGCCTC | 87734784 | 87734806 | 1.08 |
| GCAGTGTGTCTGTCCTTCCAG | 87734800 | 87734822 | 0.02 |
| GTACAGAATGAGACACATTAA | 87734826 | 87734848 | 0.78 |
| GTGCCTGTGAAGCTATTCGT | 87734854 | 87734876 | 0.71 |
| GAAGCTATTCGTAGGAAGAA | 87734862 | 87734884 | 0.02 |
| GTAGGAAGAAGGGTTTATTT | 87734872 | 87734894 | 0.11 |
| GTTATTTTGGTTCATTGTTTG | 87734886 | 87734908 | 0.01 |
| GTTGTTTGAGGGTGCTGTCCT | 87734899 | 87734921 | 0.35 |
| GAGGGTGCTGTCCTCGGAAT | 87734905 | 87734927 | 0.16 |
| GTACACAACTTCCCTATTCCG | 87734916 | 87734938 | 0.09 |
| GGGAAGTTGTGTAACGAGAG | 87734926 | 87734948 | 0.01 |
| GCTATGCCAGTTGTTCTTCCT | 87734955 | 87734977 | 0.01 |
| GTTCCCATGGACTGGGTCCT | 87734972 | 87734994 | 0.02 |
| GTGGCATGTTCCCATGGACT | 87734979 | 87735001 | 0.08 |
| GCATGTGGTGGCATGTTCCCA | 87734985 | 87735007 | 0.55 |
| GGCATGTGGGTGGCATGTGG | 87734998 | 87735020 | 0.26 |
| GGCATATGATGGCATGTGGG | 87735008 | 87735030 | 0.68 |
| GGTAGCATGTGGTAGCATGT | 87735043 | 87735065 | 0.00 |
| GTAGTAGCATGTGGTAGCATG | 87735087 | 87735109 | 0.00 |
| GGCATGTGGGTAGTAGCATG | 87735097 | 87735119 | 0.01 |
| GTGGCATGTGGGTGGCATGT | 87735121 | 87735143 | 0.02 |
| GTGATGGCATGTGATGTATG | 87735143 | 87735165 | 0.35 |
| GGCATGTGGGTGGCATGTGA | 87735159 | 87735181 | 0.01 |
| GACATATGATGGCATGTGGG | 87735169 | 87735191 | 0.25 |
| GGCATGTGGTTGACATATGA | 87735180 | 87735202 | 0.11 |
| GGTTGGCATATGATGGCATG | 87735194 | 87735216 | 0.31 |
| GGTATGTGGTTGGCATATGA | 87735201 | 87735223 | 0.01 |
| GATATGTGGTGGTATGTGGT | 87735211 | 87735233 | 0.01 |
| GGATGGCATATGATGATATG | 87735225 | 87735247 | 0.01 |
| GGGTAGCATGTGGTGGCATG | 87735246 | 87735268 | 0.01 |
| GGCATGTGGGTAGCATGTGG | 87735253 | 87735275 | 0.01 |
| GATGATGGCATGTGATGTATG | 87735288 | 87735310 | 0.00 |

| | | | |
|---|---|---|---|
| GGCATGTAGTGGCATGTGGG | 87735314 | 87735336 | 0.23 |
| GCAAATGTGGGTGGCATGTAG | 87735325 | 87735347 | 0.00 |
| GACGAGAGAGGACACAAATGT | 87735338 | 87735360 | 0.01 |
| GCCAGAGACAGTCAACGAGAG | 87735351 | 87735373 | 11.67 |
| GTCTCTGGGACAGCTTCTAC | 87735366 | 87735388 | 0.59 |
| GGGACAGCTTCTACAGGCAG | 87735372 | 87735394 | 0.01 |
| GCAGGCAGAGGTGTGTTTTCC | 87735385 | 87735407 | 2.27 |
| GTGATTCCGCAGTGAGCTAA | 87735407 | 87735429 | 0.01 |
| GATTCCGCAGTGAGCTAATGG | 87735410 | 87735432 | 0.27 |
| GATGGCTTGAGTTTGATTCT | 87735443 | 87735465 | 0.03 |
| GTCAAACTCAAGCCATCAGGT | 87735450 | 87735472 | 0.16 |
| GGGACTTGTCACCAACCTGA | 87735461 | 87735483 | 0.47 |
| GTTGAGATGTTCAGAAGGAAA | 87735482 | 87735504 | 0.02 |
| GGGCTGTTGAGATGTTCAGA | 87735488 | 87735510 | 0.01 |
| GACATCTCAACAGCCCAGGAC | 87735496 | 87735518 | 0.42 |
| GAAAATTACATTTCCAGTCCT | 87735508 | 87735530 | 0.01 |
| GCACAGATACTTAGGCTTACA | 87735540 | 87735562 | 0.00 |
| GCTAGCTACACACAGATACTT | 87735549 | 87735571 | 0.87 |
| GCTAGTGGGACATTAGAGGA | 87735567 | 87735589 | 0.03 |
| GTATCTAGCGAACACTTTACA | 87735594 | 87735616 | 0.01 |
| GCGAACACTTTACATGGTCC | 87735600 | 87735622 | 0.02 |
| GTTACATGGTCCGGGGAGTAT | 87735609 | 87735631 | 0.01 |
| GTCTCTAGTGCCTATACTCCC | 87735618 | 87735640 | 0.00 |
| GTTCCTGCAGAGGGTGGGAGC | 87735650 | 87735672 | 0.02 |
| GAGGTGCTTCCTGCAGAGGG | 87735657 | 87735679 | 0.01 |
| GGAAGCACCTCCATCTCAGT | 87735669 | 87735691 | 3.23 |
| GAAACACACCTACTGAGATGG | 87735676 | 87735698 | 0.01 |
| GGACCAGCAACTGGATGTGA | 87735703 | 87735725 | 0.58 |
| GGTGCGGCAGGACCAGCAAC | 87735712 | 87735734 | 0.08 |
| GTCCTGCCGCACCCTGTCCA | 87735722 | 87735744 | 5.16 |
| GCTGTCTCCATGGACAGGGTG | 87735728 | 87735750 | 0.01 |
| GCAGTTCTGTCTCCATGGACA | 87735733 | 87735755 | 0.01 |
| GTTATCCCAGTTCTGTCTCCA | 87735739 | 87735761 | 0.01 |
| GGGATAAGTTGTTCAAGCC | 87735755 | 87735776 | 0.32 |
| GTTCAAGCCAGGCAGACTCT | 87735765 | 87735787 | 0.02 |
| GCATCTACCAAGAGTCTGCC | 87735772 | 87735794 | 0.52 |
| GCTCTTGGTAGATGCTACCAG | 87735781 | 87735803 | 0.01 |
| GCCCATAGATAAGGAACCAC | 87735797 | 87735819 | 0.01 |
| GATGTAAGGGGCCCATAGATA | 87735806 | 87735828 | 0.02 |
| GAAGAGGAGAGAGATGTAAG | 87735819 | 87735841 | 0.06 |
| GATCTCTCTCCTCTTCCCTTC | 87735827 | 87735849 | 0.04 |
| GTCTGCCACCCTGAAGGGAAG | 87735835 | 87735857 | 1.01 |
| GATGGTCTCTGCCACCCTGAA | 87735841 | 87735863 | 0.45 |
| GTCAAATGAAGATCTGGCCTA | 87735860 | 87735882 | 0.03 |
| GATGTCATTCAAATGAAGATC | 87735867 | 87735889 | 0.02 |
| GAAGAGGGTGTGGCAAGTC | 87735899 | 87735920 | 0.01 |
| GTTTTTTACTGAAGAGGGTG | 87735908 | 87735930 | 0.01 |
| GATGCAGTTTTTTACTGAAGA | 87735913 | 87735935 | 0.01 |
| GCATGGCATGCCCAAAAGCT | 87735932 | 87735954 | 0.05 |
| GACTGACAGAGGGACAAGTT | 87736028 | 87736050 | 0.02 |
| GTCCCTCTGTCAGTCGGTGAA | 87736037 | 87736059 | 0.01 |
| GTTCCTAGGATTTTGGAACAC | 87736147 | 87736169 | 0.01 |

| | | | |
|---|---|---|---|
| GTCAACGATTTCCTAGGATTT | 87736155 | 87736177 | 0.01 |
| GTTTACAAAAATCAAAGCCCA | 87736183 | 87736205 | 0.01 |
| GAATTGAGCTAGAAATCTCA | 87736283 | 87736305 | 0.01 |
| GTTGTTCTAAGTATACTTTGT | 87736310 | 87736332 | 0.03 |
| GTATACTTAGAACAATAGCT | 87736318 | 87736340 | 0.01 |
| GCTGGGTCACTCCCATACAC | 87736335 | 87736357 | 0.03 |
| GCAGGCATTTACAATGGCCAA | 87736354 | 87736376 | 0.01 |
| GTATAATTCACAATAGGCCTT | 87736370 | 87736392 | 0.02 |
| GTCTATTGTATAATTCACAAT | 87736377 | 87736399 | 0.02 |
| GACTGCCTGGTTCCTGTTAGC | 87736498 | 87736520 | 0.00 |
| GCAGCATACATACCAGCTAAC | 87736509 | 87736531 | 0.01 |
| GTCAAGGTACACTGAGGTTAC | 87736567 | 87736589 | 0.59 |
| GGTCATTCAAGGTACACTG | 87736574 | 87736595 | 0.11 |
| GATACAATAAGGTCATTCA | 87736584 | 87736605 | 0.11 |
| GAATTAGGGTCCCAGTATTC | 87736724 | 87736746 | 0.04 |
| GACTGGGACCCTAATTCCCAG | 87736731 | 87736753 | 0.01 |
| GCCTCAACCCTCTGGGAATTA | 87736738 | 87736760 | 0.01 |
| GTGCTGTGGACTGGACTCAG | 87736764 | 87736786 | 0.04 |
| GTATCAGTCAGTGCTGTGGAC | 87736773 | 87736795 | 4.03 |
| GCCTCTTATCAGTCAGTGCTG | 87736778 | 87736800 | 0.00 |
| GACTGACTGATAAGAGGGACC | 87736785 | 87736807 | 0.01 |
| GGGACCTGGTTGCAGTCACA | 87736799 | 87736821 | 0.01 |
| GTTGCAGTCACATGGAGCCAG | 87736808 | 87736830 | 0.00 |
| GCAGCACCTCTGGCCTGCCGC | 87736824 | 87736846 | 0.03 |
| GCAACAGTCAGTCAGCACCTC | 87736835 | 87736857 | 0.04 |
| GCTGACTGACTGTTGCCATC | 87736843 | 87736865 | 0.02 |
| GTTAGAAGACGCTTATCCAGA | 87736858 | 87736880 | 0.04 |
| GTTGAGGGACACATTCACAGA | 87736896 | 87736918 | 0.05 |
| GAAGCATACTCTTTCCTTTTG | 87736913 | 87736935 | 0.01 |
| GAAAGAGTATGCTTTCAAGAG | 87736923 | 87736945 | 0.01 |
| GTTTCAAGAGTGGATTAGAGT | 87736934 | 87736956 | 0.01 |
| GTTATTCTGTACTTCCCGCTG | 87736962 | 87736984 | 0.00 |
| GTACTTCCCGCTGAGGCGG | 87736969 | 87736990 | 0.02 |
| GCAAGGTACCTCCGCCTCAGC | 87736975 | 87736997 | 0.01 |
| GTGCAAACACTTGGACTCA | 87736993 | 87737014 | 0.01 |
| GTCCAAGTGTTTGCACTGCC | 87736999 | 87737021 | 0.01 |
| GAAGGTCACACTGTGTTACCC | 87737017 | 87737039 | 0.10 |
| GCAGTTTGTTCTGGAGTCA | 87737036 | 87737057 | 0.00 |
| GATTTGGTCAGCAGTTTGTTC | 87737044 | 87737066 | 0.01 |
| GAAGACATCACATTCTTCATT | 87737061 | 87737083 | 0.20 |
| GTGATGTCTTTGCTTCATGA | 87737074 | 87737096 | 0.03 |
| GCTGAGACCTCCTACTGGAG | 87737096 | 87737118 | 0.02 |
| GTATATACCACTCCAGTAGG | 87737103 | 87737125 | 0.01 |
| GTCTTTAAAAATAAGGTAAAA | 87737125 | 87737147 | 0.03 |
| GAAAATCAATCTTTAAAAATA | 87737133 | 87737155 | 0.02 |
| GTTTAAAGATTGATTTTATTT | 87737140 | 87737162 | 0.01 |
| GCACCGTTTGTACGTGTGTGC | 87737187 | 87737209 | 0.01 |
| GTTGGGAAGTTCACAAACTC | 87737259 | 87737281 | 0.02 |
| GAACTTCCCAACATGGTGCT | 87737270 | 87737292 | 0.11 |
| GGCACACCAAGCACCATGTT | 87737276 | 87737298 | 0.01 |
| GCCTCTAAACTGTTTTTGCT | 87737296 | 87737318 | 0.87 |
| GAAACTGTTTTTGCTTGGCTG | 87737302 | 87737324 | 0.01 |

| | | | |
|---|---|---|---|
| GTTTTTGCTTGGCTGAGGTT | 87737307 | 87737329 | 0.01 |
| GGAAACTGGCAAGGGATTCT | 87737349 | 87737371 | 0.01 |
| GATCTGGAAGGAAACTGGCAA | 87737357 | 87737379 | 0.01 |
| GAAGCCCATCTGGAAGGAAAC | 87737363 | 87737385 | 0.01 |
| GACCCATCCACAAGCCCATC | 87737374 | 87737396 | 0.09 |
| GGATGGGTCACAGTTGCTGA | 87737388 | 87737410 | 0.39 |
| GTCACAGTTGCTGAGGGATG | 87737394 | 87737416 | 1.90 |
| GGTGATGACAAGACAGACAT | 87737415 | 87737437 | 0.01 |
| GTTGTAGCTCGTTGTTATTGC | 87737450 | 87737472 | 0.01 |
| GTAGGGGGTCATGGTAAAGCT | 87737474 | 87737496 | 0.03 |
| GTGGGGTCTCTAGGGGGTCA | 87737484 | 87737506 | 0.01 |
| GCTGCAGTGGGGTCTCTAGG | 87737490 | 87737512 | 0.01 |
| GACACTCTGTAGCTGCAGTG | 87737501 | 87737523 | 0.07 |
| GAGTGTCCCCTCAGCTAAAC | 87737517 | 87737539 | 0.00 |
| GTGGCTCCAGTTTAGCTGAG | 87737523 | 87737545 | 0.01 |
| GAAACTGGAGCCACTCTCACC | 87737533 | 87737555 | 4.12 |
| GCCACTCTCACCAGGACCA | 87737541 | 87737562 | 0.31 |
| GCTTTAAGTTAGCCATGGTCC | 87737551 | 87737573 | 0.01 |
| GATTGTTCTTTAAGTTAGCCA | 87737557 | 87737579 | 0.01 |
| GCTTAAAGAACAATAGTTTTC | 87737567 | 87737589 | 0.11 |
| GGTCGTCTTGTGACCATGTT | 87737588 | 87737610 | 0.33 |
| GCTTTGTCAAATGACCTAACA | 87737601 | 87737623 | 0.00 |
| GTTAGGTCATTTGACAAAGTT | 87737606 | 87737628 | 0.44 |
| GCCTTCAAAGGCACGGCGCTA | 87737653 | 87737675 | 0.01 |
| GAACGCCTCCCTTCAAAGGCA | 87737661 | 87737683 | 0.67 |
| GTTCATCTAGACCTTCGCC | 87737681 | 87737702 | 0.11 |
| GTTACAATGCGGGCCAGGCGA | 87737692 | 87737714 | 0.57 |
| GATTGACTTACAATGCGGGCC | 87737698 | 87737720 | 0.68 |
| GAAGACGATTGACTTACAATG | 87737704 | 87737726 | 0.01 |
| GTCGTCTTAGGCCATTCTACA | 87737720 | 87737742 | 0.01 |
| GCTGCACTATCCCGTGTAGAA | 87737730 | 87737752 | 0.02 |
| GGGATAGTGCAGAAATAGGC | 87737741 | 87737763 | 0.02 |
| GGCAGGAACAAACGTGCTGG | 87737758 | 87737780 | 2.90 |
| GACGTGCTGGGGGCTCCATGT | 87737769 | 87737791 | 1.59 |
| GTCCATGTTGGCTCCCCTTCC | 87737782 | 87737804 | 0.00 |
| GTTTATTTCTCTCCCAGGAA | 87737795 | 87737817 | 0.01 |
| GAAGAAGTTTATTTCTCTCCC | 87737800 | 87737822 | 0.01 |
| GAAATCATACTGGCTAACAAT | 87737824 | 87737846 | 0.01 |
| GACAGAAATCCAAAATCATAC | 87737835 | 87737857 | 0.00 |
| GCTTCCATGTCTAAGTTTCA | 87737895 | 87737917 | 0.38 |
| GACATGGAAGCCTCATTCCC | 87737907 | 87737929 | 0.01 |
| GCCTCATTCCCAGGGACTCA | 87737916 | 87737938 | 0.36 |
| GATGGGTTCCCGTGAGTCCC | 87737925 | 87737947 | 0.02 |
| GGGAACCCATCACTAGCTG | 87737937 | 87737958 | 1.43 |
| GCCGTCACCTCAGCTAGTGA | 87737943 | 87737965 | 0.00 |
| GCCGTCGACTCCCCAGTTGT | 87737964 | 87737986 | 0.02 |
| GTATCAGATCCCAACAACTG | 87737974 | 87737996 | 0.17 |
| GCTTTGTCCTTTCCACACTGT | 87738001 | 87738023 | 0.13 |
| GAGAGCCCAACAGTGTGGAA | 87738007 | 87738029 | 0.10 |
| GCTGCTGAGAGCCCAACAGTG | 87738012 | 87738034 | 0.01 |
| GCTCAACTGCTTTGACCTTTT | 87738057 | 87738079 | 0.41 |
| GACCTTTTAGGAACACGCC | 87738069 | 87738090 | 0.25 |

| | | | |
|---|---|---|---|
| GACACTGTGAAAGGGTTGTCC | 87738086 | 87738108 | 0.01 |
| GCCTTCTGAACACTGTGAAA | 87738095 | 87738117 | 0.01 |
| GTCACAGTGTTCAGAAGGCTG | 87738100 | 87738122 | 0.01 |
| GTTCAGAAGGCTGAGGACCA | 87738107 | 87738129 | 0.01 |
| GATGAAATCATCTCTCCCG | 87738124 | 87738145 | 0.01 |
| GATGTGAGGGTTCTCAGCTTT | 87738151 | 87738173 | 0.02 |
| GAAGCAAGGCTAGTAATGTGA | 87738165 | 87738187 | 0.21 |
| GCTAGCCTTGCTTAGACCACA | 87738176 | 87738198 | 0.86 |
| GCTTAGCAAAAGCTAGCCTTG | 87738191 | 87738213 | 0.01 |
| GCTAAGGGGAAACCTCTTTC | 87738208 | 87738230 | 0.01 |
| GTAAGGAAGTGTCCCAGAAAG | 87738220 | 87738242 | 0.01 |
| GAAGCTAGGTTTAAGGAGTA | 87738238 | 87738260 | 0.03 |
| GTTAAACCTAGCTTCTTTGCC | 87738247 | 87738269 | 0.09 |
| GCCACACCTGGCAAAGAAGCT | 87738252 | 87738274 | 0.18 |
| GATGGTGAATGCCACCACACC | 87738265 | 87738287 | 1.47 |
| GCATTCACCATTACTTTCAG | 87738277 | 87738299 | 1.40 |
| GCCTCCAACCACTGAAAGTAA | 87738284 | 87738306 | 0.00 |
| GCTTTCAGTGGTTGGAGGCAG | 87738290 | 87738312 | 0.74 |
| GACTCACTATCCAGTCCAGGC | 87738341 | 87738363 | 0.02 |
| GATTTGTAATCCTGGCTGTCT | 87738365 | 87738387 | 0.11 |
| GAAGGTCTTTATTTGTAATCC | 87738374 | 87738396 | 0.02 |
| GAGAGAAAGAAAGAAATAAA | 87738528 | 87738550 | 0.03 |
| GCTCCATTAGGGAGATGCTG | 87738555 | 87738577 | 0.03 |
| GAAGGACAATGAGCTCCATT | 87738567 | 87738589 | 0.01 |
| GAGGACAAATAGGCTAAGCC | 87738589 | 87738611 | 0.00 |
| GCTTAGCCTATTTGTCCTCC | 87738593 | 87738615 | 0.08 |
| GAGAACCCTGGAGGACAAAT | 87738599 | 87738621 | 0.01 |
| GATGGAAACTGAGAACCCTGG | 87738608 | 87738630 | 0.17 |
| GGCTGGAAGTGAGACCACA | 87738627 | 87738648 | 0.01 |
| GTTTCAGGCTAAGTAGGTGGC | 87738643 | 87738665 | 0.16 |
| GCCCCAGGTTTCAGGCTAAGT | 87738650 | 87738672 | 0.02 |
| GCCTGAAACCTGGGGCCTCT | 87738658 | 87738680 | 0.01 |
| GTTGTAAGTCCAAGAGGCCCC | 87738666 | 87738688 | 0.01 |
| GACAAAAATTGTAAGTCCAAG | 87738673 | 87738695 | 0.01 |
| GTTGTTAGGATTGTGTTTATT | 87738692 | 87738714 | 0.00 |
| GTTGCCGTGACACATTCGTGG | 87738734 | 87738756 | 2.13 |
| GACACATTCGTGGAGGTCAG | 87738741 | 87738763 | 0.04 |
| GATTCTTTCTGTCTGCCATG | 87738780 | 87738802 | 0.06 |
| GATTCAAGCTCAACCCCACA | 87738795 | 87738817 | 0.00 |
| GGGGTTGAGCTTGAATCTTC | 87738801 | 87738823 | 0.40 |
| GCTCAGGAAGTCGACACATC | 87738831 | 87738853 | 0.01 |
| GAAAGATCAGTGAGGCAGCTC | 87738847 | 87738869 | 0.01 |
| GTCTGAGAGAAAGATCAGTG | 87738856 | 87738878 | 0.01 |
| GCTCAGACTTCTGCCCACAAG | 87738872 | 87738894 | 0.11 |
| GCCATGACTGTGCCCGCTTG | 87738885 | 87738907 | 0.93 |
| GGCACAGTCATGGCTTCTGT | 87738894 | 87738916 | 0.01 |
| GCTGTGGAAGGGAGGGGGGCT | 87738917 | 87738939 | 0.01 |
| GAAAAGTCTGTGGAAGGGAG | 87738924 | 87738946 | 0.02 |
| GGGAAGAAAAGTCTGTGGAA | 87738929 | 87738951 | 0.06 |
| GTCAAGGGAAGAAAAGTCTG | 87738934 | 87738956 | 0.01 |
| GTTCTTCCCTTGACATTCTAC | 87738944 | 87738966 | 0.01 |
| GCTCATCCTGTAGAATGTCA | 87738950 | 87738972 | 0.01 |

| | | | |
|---|---|---|---|
| GGATGAGCTGAAACTGCTT | 87738965 | 87738986 | 1.04 |
| GGCTTACGAAGCCTTTCATG | 87738985 | 87739007 | 0.06 |
| GCTGAGGGTCAGCCTCATGAA | 87738996 | 87739018 | 0.06 |
| GCAGAACGAAGATGTGACTGA | 87739012 | 87739034 | 0.01 |
| GTTCTGTCACCCCAGACCTG | 87739029 | 87739051 | 0.02 |
| GTCGTTCGCGCCTCAGGTCTG | 87739038 | 87739060 | 0.02 |
| GAGCATATCGTTCGCGCCTC | 87739045 | 87739067 | 0.03 |
| GCGAACGATATGCTCCTTTC | 87739053 | 87739075 | 0.82 |
| GAGGCAGGCTCTTTCCAGAA | 87739067 | 87739089 | 0.02 |
| GCGGCACTGAAGACAAACAG | 87739086 | 87739108 | 0.04 |
| GCTACTCACGGGCAGAGGGAG | 87739105 | 87739127 | 0.01 |
| GGAGCCTACTCACGGGCAGA | 87739110 | 87739132 | 0.00 |
| GCTCCAGGGAGCCTACTCAC | 87739117 | 87739139 | 0.20 |
| GCATAGGAAGAGGAGGCTCCA | 87739131 | 87739153 | 0.01 |
| GACTGAGTCCATAGGAAGAGG | 87739139 | 87739161 | 0.01 |
| GCTTTCACACTGAGTCCAT | 87739148 | 87739169 | 0.67 |
| GAAAGCTTTCTCTGCAGCAT | 87739164 | 87739186 | 0.11 |
| GAGTGACCACAAGTTTTCAG | 87739186 | 87739208 | 0.00 |
| GCTGGGACCACTGAAAACTTG | 87739192 | 87739214 | 4.56 |
| GCACAGTCACTTGTGGATGCT | 87739210 | 87739232 | 0.01 |
| GTCTGTAGCCACAGTCACTTG | 87739218 | 87739240 | 0.06 |
| GCGTGTCAGCACAGGCGGGG | 87739242 | 87739264 | 0.41 |
| GGCTCTGGCGTGTCAGCAC | 87739250 | 87739271 | 0.02 |
| GCACGCCAGAGCCTGGAGTCC | 87739260 | 87739282 | 0.01 |
| GCGGCAGACCCTGGACTCC | 87739270 | 87739291 | 0.01 |
| GAGTTAGCGCGGCAGACCC | 87739278 | 87739299 | 0.01 |
| GAATGAGAAAACGAGTTAGCG | 87739288 | 87739310 | 0.01 |
| GTTATTGAATCAGAGGCTGA | 87739316 | 87739338 | 0.01 |
| GTTTGGGGTTATTGAATCAG | 87739323 | 87739345 | 0.05 |
| GTTAAGGGCTTCTCTAGTTT | 87739339 | 87739361 | 0.01 |
| GAGAAGCCCTTAACTTCTG | 87739348 | 87739369 | 0.42 |
| GCCCTTAACTTCTGCGGCCC | 87739353 | 87739375 | 0.01 |
| GCGGCCCAGGACAAACTGGA | 87739366 | 87739388 | 0.13 |
| GTTCCCCCTCCAGTTTGTCCT | 87739370 | 87739392 | 0.01 |
| GCATGCAGCAGGTCAGGCTGC | 87739438 | 87739460 | 5.93 |
| GAACTTAGCATGCAGCAGGTC | 87739445 | 87739467 | 0.02 |
| GAAGGGAACTTAGCATGCAGC | 87739450 | 87739472 | 0.14 |
| GATGCTAAGTTCCCTTTTCTT | 87739458 | 87739480 | 0.18 |
| GCTAATGAGTACCAAAGAAAA | 87739468 | 87739490 | 0.02 |
| GACACAGATATCTTCATAGAA | 87739493 | 87739515 | 0.00 |
| GAAGGTGACCAACCACAGAGG | 87739512 | 87739534 | 0.06 |
| GACCAACCACAGAGGAGGCA | 87739517 | 87739539 | 1.97 |
| GCTCACCCTGCCTCCTCTG | 87739523 | 87739544 | 0.02 |
| GCAGGGTGAGCGATTCCACCT | 87739535 | 87739557 | 0.00 |
| GAGTCCAACGGCGAATGCCT | 87739552 | 87739574 | 0.29 |
| GATGACATTTTAGGAGTCCAA | 87739564 | 87739586 | 0.95 |
| GGACTCCTAAAATGTCATTC | 87739569 | 87739591 | 0.01 |
| GAATGTCATTCAGGAATCACG | 87739579 | 87739601 | 0.42 |
| GTTCAGGAATCACGAGGTCTT | 87739586 | 87739608 | 0.01 |
| GTGACCTTTCGCTGCTTTTC | 87739610 | 87739632 | 0.00 |
| GATCTCTTCATTGAATGATTC | 87739632 | 87739654 | 0.75 |
| GATGTATGTTCCACACAGCT | 87739652 | 87739674 | 0.01 |

| | | | |
|---|---|---|---|
| GCCAGCTGTGGCCCAGCTGTG | 87739662 | 87739684 | 0.01 |
| GTTGATGAGGGTCTCCAGCTG | 87739675 | 87739697 | 0.01 |
| GACCCTCATCAAACACAGAGC | 87739687 | 87739709 | 0.01 |
| GCTGGTGGGTGACTGAAGCCC | 87739706 | 87739728 | 0.13 |
| GCTAAAATTCTGGACACACC | 87739724 | 87739746 | 0.55 |
| GCATTAGCTAGGCTAAAATTC | 87739734 | 87739756 | 0.02 |
| GCCTAGCTAATGGTCTTAAT | 87739745 | 87739767 | 0.02 |
| GAATGGTCTTAATTGGACACA | 87739753 | 87739775 | 0.02 |
| GCAGTAGCATTTCTGGAAAGT | 87739786 | 87739808 | 0.00 |
| GTTCCAGAAATGCTACTGGTC | 87739792 | 87739814 | 0.01 |
| GTCAGGGTGCTTTAAGTGGG | 87739809 | 87739831 | 0.02 |
| GGGAGTGTGACTCCACAGA | 87739830 | 87739851 | 0.03 |
| GCTCAATTGGTTCACCATCTG | 87739842 | 87739864 | 0.00 |
| GCTAGAGAAAGCTGCTCAAT | 87739856 | 87739878 | 1.58 |
| GTTCTCTAGCATTAAAACAAA | 87739870 | 87739892 | 0.01 |
| GTTAAAACAAAAGGCAAAAAC | 87739880 | 87739902 | 0.01 |
| GACAAAAGGCAAAAACTGGTA | 87739885 | 87739907 | 0.60 |
| GAAAAACTGGTATGGAGTCAG | 87739894 | 87739916 | 0.38 |
| GCATGATAAGAATAGGCATGC | 87739968 | 87739990 | 0.01 |
| GCATACACATGATAAGAAT | 87739976 | 87739997 | 0.01 |
| GTCATGTGTATGCAGTGCTCA | 87739986 | 87740008 | 0.01 |
| GCAGGGACCAACGCCCTCTTC | 87740012 | 87740034 | 0.01 |
| GGGCGTTGGTCCCTGGAAC | 87740020 | 87740041 | 0.01 |
| GTACAGCCAATTGTAAGCAAC | 87740045 | 87740067 | 3.72 |
| GAATCCCAGTTGCTTACAAT | 87740050 | 87740072 | 0.01 |
| GCAACTGGGATTCCAAGAAGC | 87740061 | 87740083 | 0.01 |
| GAAGATTGGATCCCAGCTTCT | 87740072 | 87740094 | 0.02 |
| GCAGTTGCAGAGGGCCAAGAT | 87740087 | 87740109 | 0.70 |
| GAGGTTAAGAGCAGTTGCAG | 87740098 | 87740120 | 1.05 |
| GGGATGGAGACATGATTAG | 87740117 | 87740138 | 0.00 |
| GACAAAAAGTTTAATCAGGGA | 87740132 | 87740154 | 0.01 |
| GATGGAACAAAAAGTTTAATC | 87740137 | 87740159 | 4.31 |
| GCATGACATTACATGATAAAA | 87740156 | 87740178 | 0.22 |
| GAAAGTCCCTGCTGTAGACA | 87740178 | 87740200 | 0.01 |
| GCCTTCTCCTTGTCTACAGCA | 87740184 | 87740206 | 0.01 |
| GACAAGGAGAAGGGAGAGGC | 87740194 | 87740216 | 0.03 |
| GAGAGGCTGGTACATTTGGT | 87740207 | 87740229 | 0.00 |
| GCTGGTACATTTGGTTGGTTG | 87740213 | 87740235 | 0.01 |
| GTTTGGTTGGTTGCGGTCAGC | 87740221 | 87740243 | 3.91 |
| GGTCAGCAGGGGTAGAGTTT | 87740234 | 87740256 | 0.05 |
| GGGGTAGAGTTTAGGATGC | 87740242 | 87740263 | 0.01 |
| GCTGGAGAGTTTGTAGACTT | 87740259 | 87740281 | 0.03 |
| GTTTGTAGACTTTGGCGGGT | 87740267 | 87740289 | 0.02 |
| GTTGGCGGGTGGGGCCAGGAG | 87740278 | 87740300 | 0.00 |
| GCGGGTGGGGCCAGGAGAGG | 87740281 | 87740303 | 0.01 |
| GCTTGCTCCACCCACCTCTCC | 87740291 | 87740313 | 0.00 |
| GAGGTGGGTGGAGCAAGGGT | 87740297 | 87740319 | 0.15 |
| GGGTGGAGCAAGGGTGGGTG | 87740302 | 87740324 | 0.02 |
| GCAAGGGTGGGTGTGGCCA | 87740309 | 87740330 | 0.01 |
| GGTGGGTGTGGCCATGGATT | 87740314 | 87740336 | 0.01 |
| GCTTCCATGCATCCTAATCCA | 87740325 | 87740347 | 0.14 |
| GGATGCATGGAAGATGATAA | 87740335 | 87740357 | 0.10 |

| | | | |
|---|---|---|---|
| GATGATAAAGGAGGTAGAAAC | 87740348 | 87740370 | 0.01 |
| GAAAGGAGGTAGAAACTGGTT | 87740353 | 87740375 | 0.98 |
| GAAACTGGTTAGGAAGTTTA | 87740363 | 87740385 | 2.81 |
| GTTTCTTTCTTGGCTAAAAGC | 87740415 | 87740437 | 0.01 |
| GGCTAAAAGCAGGAAATGTC | 87740425 | 87740447 | 0.01 |
| GGAAATGTCAGGCCTACCC | 87740436 | 87740457 | 0.01 |
| GCATCTCTGAGCTCTAGCCA | 87740452 | 87740474 | 0.27 |
| GCATTTGAGCTTAAACATTTC | 87740474 | 87740496 | 0.01 |
| GTTTAAGCTCAAATGTCTC | 87740482 | 87740503 | 0.39 |
| GAAATGTCTCTGGTTCTGGTA | 87740492 | 87740514 | 0.01 |
| GTTCTGGTATGGTCAGAGGC | 87740503 | 87740525 | 0.01 |
| GTCAGAGGCTGGGCAACGGG | 87740514 | 87740536 | 0.01 |
| GTTTTCAGGGAAATCGAGATA | 87740588 | 87740610 | 0.02 |
| GCAGGAGGTGGGTATTTTTTC | 87740603 | 87740625 | 0.01 |
| GCATTATTATTTCCAGGAGGT | 87740615 | 87740637 | 0.04 |
| GAAAGTTCTAGACACGTGCCA | 87740650 | 87740672 | 0.34 |
| GTAGACACGTGCCATGGGAAC | 87740657 | 87740679 | 0.01 |
| GCTGGTCAGCCCAGTTCCCA | 87740667 | 87740689 | 1.45 |
| GGGAGAGGGGGAAGGCAAGC | 87740685 | 87740707 | 0.02 |
| GATGTTGGGGAAAGGGAGAGG | 87740697 | 87740719 | 0.41 |
| GAGTTAATAATGTTGGGGAA | 87740706 | 87740728 | 0.00 |
| GCCTTGGAGTTAATAATGTT | 87740712 | 87740734 | 0.01 |
| GTTATTAACTCCAAGGCAGCC | 87740719 | 87740741 | 0.03 |
| GTCCAAGGCAGCCAGGCTTAG | 87740727 | 87740749 | 0.22 |
| GAAATAGGGTACCACTAAGCC | 87740737 | 87740759 | 0.23 |
| GCTTAGTGGTACCCTATTTCC | 87740742 | 87740764 | 0.02 |
| GCAGGGAGGATACCAGGAAAT | 87740753 | 87740775 | 0.21 |
| GTCTGTAGCAGGGAGGATACC | 87740760 | 87740782 | 0.02 |
| GAACAGAGGTCCTCTGTAGC | 87740772 | 87740794 | 0.26 |
| GAGGAAAAGAAAAAGAACAG | 87740786 | 87740808 | 0.02 |
| GAATAGGCAAACACTTACAAG | 87740805 | 87740827 | 0.02 |
| GATGTTTGTTATAGACCAAT | 87740822 | 87740844 | 0.02 |
| GCATCTCTCTAACACCTTCTG | 87740841 | 87740863 | 0.01 |
| GGGATTCACAGACCACAGA | 87740854 | 87740875 | 0.01 |
| GTTTGAAAAATTTGAAGAGT | 87740873 | 87740895 | 0.01 |
| GCAAACAAGTACCTGATATCT | 87740891 | 87740913 | 0.01 |
| GAAGTACCTGATATCTTGGGG | 87740896 | 87740918 | 0.01 |
| GCAAAGCCACCCCAAGATATC | 87740901 | 87740923 | 0.01 |
| GGGGTGGCTTTGGTGGCTAT | 87740912 | 87740934 | 0.01 |
| GTTTGGTGGCTATTGGATTTC | 87740920 | 87740942 | 0.00 |
| GGGGCCCTAGATCCCGAGAG | 87740941 | 87740963 | 0.69 |
| GCAGAGCCTCTCTCGGGATCT | 87740946 | 87740968 | 0.06 |
| GGGACAACAGAGCCTCTCTC | 87740953 | 87740975 | 0.00 |
| GATTATGGAGGAGTCTTGGG | 87740977 | 87740999 | 0.59 |
| GCAGGATTATGGAGGAGTCT | 87740981 | 87741003 | 0.46 |
| GCTAGAGGTGCAGGATTATGG | 87740989 | 87741011 | 1.30 |
| GTAATCCTGCACCTCTAGGAG | 87740995 | 87741017 | 0.01 |
| GCCTCTGCGCGCCCCTCCTAG | 87741005 | 87741027 | 0.01 |
| GAGGGGCGCGCAGAGGAGGA | 87741012 | 87741034 | 0.01 |
| GTCTCAGTAGCTCTGTGATGC | 87741038 | 87741060 | 0.19 |
| GCTGTGATGCTGGAGCTGTAA | 87741049 | 87741071 | 0.01 |
| GTTCTAGTGCTTTCTTTAGCA | 87741076 | 87741098 | 0.07 |

| | | | |
|---|---|---|---|
| GCTAGAAGGAACCCATCTCCC | 87741093 | 87741115 | 0.82 |
| GCCTCATGTCCCCAGGGAGAT | 87741103 | 87741125 | 0.01 |
| GGGGGCCCCTCATGTCCCCA | 87741110 | 87741132 | 0.01 |
| GAGGCATCACGAGGACATGG | 87741128 | 87741150 | 0.00 |
| GATGTCCTCGTGATGCCTCTC | 87741133 | 87741155 | 0.06 |
| GCAAGGTGTCCCCACCCTGAG | 87741147 | 87741169 | 1.95 |
| GACAAAGTGTGCAGAGATGCA | 87741165 | 87741187 | 3.02 |
| GTACTTCTGCTTACTCCTAAC | 87741194 | 87741216 | 1.83 |
| GCTTACTCCTAACAGGGGTG | 87741201 | 87741223 | 0.03 |
| GCATGACTCCACACCCCTGTT | 87741208 | 87741230 | 0.07 |
| GTGGAGTCATGAACCCATT | 87741220 | 87741241 | 0.10 |
| GATGAACCCATTTGGAAGATG | 87741228 | 87741250 | 0.01 |
| GCTCTCCTCATCTTCCAAAT | 87741233 | 87741255 | 0.02 |
| GAAGATGAGGAGAGCAAGGAG | 87741242 | 87741264 | 0.01 |
| GGATCTAGATCTGCAGTCA | 87741273 | 87741294 | 0.08 |
| GCTGATCAGAGCTCTGGATGC | 87741293 | 87741315 | 0.02 |
| GATCCAGAGCTCTGATCAGAG | 87741298 | 87741320 | 0.02 |
| GTACAGACAGCAAGCGCATTG | 87741328 | 87741350 | 0.02 |
| GCGCTTGCTGTCTGTAGTGC | 87741335 | 87741357 | 0.01 |
| GTACTGTTGTAAGGAGTGC | 87741369 | 87741390 | 0.02 |
| GCAGGCATTAGTACTGTTGTA | 87741377 | 87741399 | 0.01 |
| GCCTGCCCAGTGTCCCTCCT | 87741395 | 87741417 | 0.35 |
| GGGCCAGGCCCTACCCAGGA | 87741408 | 87741430 | 0.01 |
| GCTTGAAAAGATGCTGGGGCC | 87741423 | 87741445 | 0.01 |
| GTGATCTTGAAAAGATGCTG | 87741428 | 87741450 | 0.23 |
| GCATCTTTTCAAGATCACTT | 87741433 | 87741455 | 0.01 |
| GATCACTTTGGAGGGTTGTG | 87741445 | 87741467 | 0.25 |
| GGAGGGTTGTGTGGACAACC | 87741454 | 87741476 | 0.01 |
| GACAACCAGGTTTGAGAACA | 87741467 | 87741489 | 0.06 |
| GCATGACCATGTTCTCAAACC | 87741472 | 87741494 | 0.00 |
| GGTAGAGTCAGAGCAAGCA | 87741498 | 87741519 | 0.41 |
| GTCTACCAACCTAGCCCAGCC | 87741514 | 87741536 | 0.01 |
| GAACCTAGCCCAGCCTGGCTC | 87741520 | 87741542 | 0.02 |
| GAGCTACCCCTGAGCCAGGC | 87741528 | 87741550 | 0.01 |
| GTAGAACAATCAATCCCCT | 87741560 | 87741581 | 0.08 |
| GTCAGACTTGTGGCTGCCAAG | 87741574 | 87741596 | 0.03 |
| GCCACAAGTCTGAAAGGGCC | 87741584 | 87741606 | 0.01 |
| GAAGTCTGAAAGGGCCGGGAG | 87741589 | 87741611 | 0.10 |
| GCCTGCTCACACTGCCTCTCC | 87741602 | 87741624 | 0.01 |
| GGCAGTGTGAGCAGGGCAAC | 87741610 | 87741632 | 0.43 |
| GTGAGCAGGGCAACAGGAGG | 87741616 | 87741638 | 0.00 |
| GAGCTGTTTTGCTTTCTGTC | 87741647 | 87741669 | 0.01 |
| GCAGAAAGCAAAACAGCTCAA | 87741652 | 87741674 | 0.01 |
| GAAAACAGCTCAAAGGGTGGC | 87741660 | 87741682 | 0.44 |
| GTTTGCTCTGAGAGCACTAAA | 87741712 | 87741734 | 0.01 |
| GTCTCAGAGCAAAGTGCTGCT | 87741723 | 87741745 | 0.32 |
| GTGTTGTTTTCAAGTGCTGT | 87741756 | 87741778 | 0.06 |
| GGGGAGTGGTTCGAGCATGC | 87741783 | 87741805 | 2.30 |
| GATGCTGTGTTCTCTGGGGAG | 87741797 | 87741819 | 0.65 |
| GTTCTGATGCTGTGTTCTCTG | 87741802 | 87741824 | 0.00 |
| GCAGAAATGAGTAGTAATGCC | 87741820 | 87741842 | 0.02 |
| GCCAGGGCCAGCTTCCTTGG | 87741837 | 87741859 | 0.69 |

| | | | |
|---|---|---|---|
| GCCGGCCCCCCCCAAGGAAGC | 87741844 | 87741866 | 0.01 |
| GCTTCCTTGGGGGGGGCCGG | 87741847 | 87741869 | 0.01 |
| GGGGGGGCCGGGGGGGGGGT | 87741856 | 87741878 | 0.02 |
| GGCCGGGGGGGGGGTCGGGG | 87741861 | 87741883 | 0.07 |
| GGGGGGGTCGGGGAGGAAGC | 87741868 | 87741890 | 0.03 |
| GGAGGAAGCAGGGTCTTGGG | 87741879 | 87741901 | 0.01 |
| GGGAGGGGTTGCTGCAGAA | 87741896 | 87741917 | 0.01 |
| GCTCCTCCTCAGAACATAGTG | 87741924 | 87741946 | 0.01 |
| GCCAGCCTCACTATGTTCTG | 87741929 | 87741951 | 0.01 |
| GGCTGGCTTTGCTCTGTCAC | 87741945 | 87741967 | 0.60 |
| GCTTTGCTCTGTCACAGGTC | 87741950 | 87741972 | 0.16 |
| GTCACAGGTCAGGATGCCTAT | 87741961 | 87741983 | 0.00 |
| GTCAGGATGCCTATAGGTTC | 87741967 | 87741989 | 0.01 |
| GTATAGGTTCTGGGGCTTCTC | 87741978 | 87742000 | 0.01 |
| GCTCTGGGGTCACGAGAGTCC | 87741995 | 87742017 | 0.01 |
| GAGTCCAGGCCCCACATCTG | 87742009 | 87742031 | 0.70 |
| GTCTCAACACCACAGATGTG | 87742018 | 87742040 | 0.00 |
| GTGGTGTTGAGACCATGTG | 87742028 | 87742049 | 0.01 |
| GTTGAGACCATGTGTGGCC | 87742033 | 87742054 | 1.07 |
| GAAGCCATTCCTGGCCACACA | 87742040 | 87742062 | 0.80 |
| GGACCTACTGAAGCCATTCC | 87742050 | 87742072 | 0.02 |
| GTTTTCCCCTTCCCACTCTGC | 87742073 | 87742095 | 0.12 |
| GCTCCGCCAGCAGAGTGGGA | 87742079 | 87742101 | 0.02 |
| GCGGAGCCTTCAGAGTCACG | 87742095 | 87742117 | 0.03 |
| GATAGCACCACGTGACTCTGA | 87742101 | 87742123 | 0.01 |
| GGCTTTGTCTGTCTGTCTCT | 87742132 | 87742154 | 0.01 |
| GCAAAGCCCCAGCGTCTCTTC | 87742148 | 87742170 | 0.01 |
| GCAGTGTCCAGAAGAGACGCT | 87742154 | 87742176 | 0.02 |
| GACTGAAGTGCCCTGAGCTGC | 87742173 | 87742195 | 0.33 |
| GCCCTGAGCTGCTGGCTGGC | 87742181 | 87742203 | 0.01 |
| GATGCTCTATCAGTGTCTGGG | 87742205 | 87742227 | 0.01 |
| GTAACCTTTTACACTCATAG | 87742242 | 87742264 | 0.01 |
| GGTTACATTAAGATAGACCC | 87742259 | 87742281 | 0.02 |
| GATAGACCCAGGTGGTCCAT | 87742270 | 87742292 | 0.01 |
| GACCCAGGTGGTCCATAGGCC | 87742275 | 87742297 | 0.01 |
| GTGGTCCATAGGCCCGGTGT | 87742281 | 87742303 | 0.01 |
| GTCAGCCAACACCGGGCCTA | 87742286 | 87742308 | 0.01 |
| GGACCGAGTCAGCCAACACC | 87742293 | 87742315 | 0.24 |
| GTAGAAGAGACACTTCTGGAG | 87742314 | 87742336 | 0.02 |
| GATCAATAGAAGAGACACTTC | 87742319 | 87742341 | 0.01 |
| GTCTTACAGGTTTTGGAGTGA | 87742355 | 87742377 | 0.03 |
| GCACCTCTTCTTACAGGTTT | 87742363 | 87742385 | 0.01 |
| GCAGAAGGCACCTCTTCTTAC | 87742369 | 87742391 | 0.04 |
| GAGGTGCCTTCTGGGTGTTT | 87742379 | 87742401 | 0.32 |
| GGTCACCAAAACACCCAGA | 87742385 | 87742406 | 0.02 |
| GGTGACCATCTACTTCTTAG | 87742400 | 87742422 | 0.51 |
| GCTTAGGGGTGGGCTTGAGAC | 87742415 | 87742437 | 0.02 |
| GTGGGCTTGAGACTGGATGC | 87742422 | 87742444 | 3.77 |
| GCACAAAACTCCCCAACACTG | 87742479 | 87742501 | 0.12 |
| GCTTTGTTCCCCTCAGTGTT | 87742489 | 87742511 | 0.04 |
| GACTGAGGGGAACAAAGCCCT | 87742495 | 87742517 | 0.02 |
| GAATTCTCTCGAGATCTCCTA | 87742511 | 87742533 | 0.03 |

| | | | |
|---|---|---|---|
| GATCTCGAGAGAATTAAAC | 87742519 | 87742540 | 0.06 |
| GAAACCGTTCTTCTCAAAG | 87742546 | 87742567 | 0.06 |
| GCTTCTCAAAGTGGCCTCACC | 87742555 | 87742577 | 0.05 |
| GCTGAGGGACCCTCCCAGGTG | 87742568 | 87742590 | 0.35 |
| GCCTTTCTGAGGGACCCTCCC | 87742573 | 87742595 | 0.01 |
| GAAGTCACTAGCCCTTTCTGA | 87742584 | 87742606 | 0.09 |
| GAAAGGGCTAGTGACTTGTTG | 87742591 | 87742613 | 0.00 |
| GCGTGTACGCACGACACGCA | 87742623 | 87742645 | 0.01 |
| GACGCACGACACGCAAGGGCT | 87742629 | 87742651 | 0.00 |
| GGGCTCCCTGTCACTGTTCA | 87742650 | 87742672 | 0.01 |
| GCTGTCACTGTTCAAGGGTGG | 87742657 | 87742679 | 0.01 |
| GGTGGAGGCCTGAGATTGTC | 87742672 | 87742694 | 0.49 |
| GCTAAGTGGCCAGACAATCTC | 87742680 | 87742702 | 0.44 |
| GCCACTTAGACCATCCCACC | 87742694 | 87742716 | 0.01 |
| GTATAGCTCAAGGTCCTGGT | 87742708 | 87742730 | 0.01 |
| GACCAGGACCTTGAGCTATAC | 87742711 | 87742733 | 0.25 |
| GAGCTATACTGGGAAGACAC | 87742722 | 87742744 | 1.13 |
| GACACAGGGACTGACTGCA | 87742737 | 87742758 | 0.21 |
| GACTGACTGCATGGCTTTAG | 87742745 | 87742767 | 0.56 |
| GCATGGCTTTAGTGGGTGA | 87742753 | 87742774 | 0.01 |
| GTGGGTGATGGCATCAACAG | 87742764 | 87742786 | 1.53 |
| GAAGCCTTCTTAGAGTTAATT | 87742799 | 87742821 | 0.82 |
| GAAGGCTTGATGTCCAGCCCC | 87742815 | 87742837 | 0.73 |
| GACACAGAACACAGAAGCCAG | 87742831 | 87742853 | 0.01 |
| GAATCCTTTGTCTTCTGGTTG | 87742856 | 87742878 | 0.27 |
| GACAGTGTTCTAGTGCATTGT | 87742906 | 87742928 | 1.23 |
| GTGTGTGTGTACCTGAGGTC | 87742959 | 87742981 | 1.93 |
| GGCTCACCTGCCTGACCTC | 87742970 | 87742991 | 0.02 |
| GCAGGTGAGCCGCAGCCCAC | 87742981 | 87743003 | 0.01 |
| GCTCTCAGCTCCTGTGGGCTG | 87742990 | 87743012 | 0.39 |
| GCCAATGCTCTCAGCTCCTGT | 87742996 | 87743018 | 0.02 |
| GCATTGGTGAGCTGCCAGCCA | 87743013 | 87743035 | 0.24 |
| GGTGTCTCTTGAATGACCCA | 87743030 | 87743052 | 0.26 |
| GTCAAGAGACACCCATGATCA | 87743041 | 87743063 | 0.01 |
| GACACCCATGATCATGGAAG | 87743047 | 87743069 | 0.01 |
| GGAAGAGGTGTTACCATCTA | 87743062 | 87743084 | 0.18 |
| GGTGTTACCATCTAAGGATG | 87743068 | 87743090 | 0.01 |
| GAAGGTCTCCACATCCTTAGA | 87743075 | 87743097 | 0.37 |
| GTGGAGACCTTCTTAGTAAA | 87743087 | 87743109 | 0.05 |
| GTCAGGACCCCTTTACTAAGA | 87743094 | 87743116 | 0.02 |
| GGGAACAGCGACGTCGATC | 87743112 | 87743133 | 0.01 |
| GTCGCTGTTCCCAAAGTGAT | 87743122 | 87743144 | 0.09 |
| GAAAAGCTCTCCTATCACTTT | 87743131 | 87743153 | 0.01 |
| GCTTCTTAGCTTTCCCTTAAA | 87743155 | 87743177 | 0.01 |
| GTGGTATGGCTTCCCGTTTA | 87743168 | 87743190 | 0.01 |
| GATCAATGGAGTTATGTGGTA | 87743182 | 87743204 | 0.91 |
| GATGGATCAATGGAGTTATG | 87743187 | 87743209 | 0.18 |
| GCAGGGGACTTGATGGATCAA | 87743197 | 87743219 | 0.02 |
| GATCCATCAAGTCCCCTGACC | 87743203 | 87743225 | 0.08 |
| GAAGCTGGGGCTTCCGGGTCA | 87743215 | 87743237 | 0.01 |
| GCTTGAAAAGCTGGGGCTTCC | 87743221 | 87743243 | 0.00 |
| GCTGAGCTCTTGAAAAGCTG | 87743229 | 87743251 | 0.05 |

| | | | |
|---|---|---|---|
| GTTCAAGAGCTCAGCAGCCTC | 87743238 | 87743260 | 0.15 |
| GCTCAGCAGCCTCTGGGCATC | 87743246 | 87743268 | 0.01 |
| GTCAGTTCCCCAGATGCCCAG | 87743254 | 87743276 | 0.01 |
| GATCTGGGGAACTGAATCCTT | 87743263 | 87743285 | 1.00 |
| GAATCCTTTGGTTTGTACAG | 87743275 | 87743297 | 0.01 |
| GTTTGTACAGGGGAGAATGA | 87743285 | 87743307 | 0.64 |
| GGTATGTCGTACCACATAGT | 87743306 | 87743328 | 0.01 |
| GTACCACATAGTAGGACAGAC | 87743315 | 87743337 | 0.01 |
| GACAGACTGGTGTTATGTGG | 87743328 | 87743350 | 0.02 |
| GTTATGTGGTGGGGACCAGA | 87743339 | 87743361 | 0.01 |
| GTGGTGGGGACCAGAGGGAC | 87743344 | 87743366 | 0.01 |
| GCATCTTGGTCCCTGTCCCTC | 87743354 | 87743376 | 0.02 |
| GGACAGGGACCAAGATGTAT | 87743360 | 87743382 | 0.45 |
| GCCAGTGACACCTATACATCT | 87743369 | 87743391 | 0.61 |
| GAGATACTATGTATTGTACT | 87743397 | 87743419 | 0.12 |
| GATACTATGTATTGTACTTGG | 87743400 | 87743422 | 0.01 |
| GTGGAGGACAGAGATTTCTC | 87743419 | 87743441 | 0.02 |
| GTTCTCTGGGAAGTCACGAGA | 87743434 | 87743456 | 0.02 |
| GTCACGAGAAGGAAGGAGAA | 87743445 | 87743467 | 0.00 |
| GTAGTGTGAGGGGGCAGGGAA | 87743473 | 87743495 | 0.00 |
| GACGCATAGTGTGAGGGGGC | 87743479 | 87743501 | 1.43 |
| GAAATAGACGCATAGTGTGA | 87743485 | 87743507 | 0.01 |
| GTATGCGTCTATTTCCTTTAG | 87743494 | 87743516 | 0.01 |
| GCAGTTAGAGCAAGCCTCTAA | 87743507 | 87743529 | 0.01 |
| GGCTTTGGAAGAAGGCAAG | 87743533 | 87743554 | 0.01 |
| GTATGCTTTGGCTTTGGAAGA | 87743540 | 87743562 | 0.01 |
| GTCCAAAGCCAAAGCATAGGG | 87743546 | 87743568 | 0.01 |
| GCCAAAGCATAGGGAGGGAA | 87743552 | 87743574 | 0.13 |
| GAGGGAATGGGAAACAGCTA | 87743565 | 87743587 | 1.07 |
| GTCTGCGTTGCTTACGTTGCA | 87743588 | 87743610 | 0.11 |
| GCAGAGAGCCTAAGAAGCA | 87743606 | 87743627 | 0.57 |
| GCCTCTGTCACCATGCTTCTT | 87743614 | 87743636 | 0.23 |
| GGTGACAGAGGATGCTGCCA | 87743626 | 87743648 | 0.00 |
| GCAGAGGATGCTGCCAAGGGA | 87743631 | 87743653 | 0.01 |
| GCTGCCAAGGGACGGACGCT | 87743639 | 87743661 | 0.97 |
| GCATTCTTCTTCCCCAGAGG | 87743661 | 87743683 | 0.01 |
| GTCTTCCCCAGAGGAGGCTCC | 87743668 | 87743690 | 0.02 |
| GCCACCTCCAGGAGCCTCCTC | 87743674 | 87743696 | 0.00 |
| GAGGATGGGGAGCCACCTCC | 87743686 | 87743708 | 0.55 |
| GGTGGCTCCCCATCCTCTCA | 87743692 | 87743714 | 0.05 |
| GAATCCTCCGTGAGAGGATG | 87743699 | 87743721 | 0.78 |
| GTCTGTGGAATCCTCCGTGAG | 87743705 | 87743727 | 0.41 |
| GAACAAACACGTAGCTCTG | 87743721 | 87743742 | 0.86 |
| GTGTTTGTTCATCAGTACGG | 87743733 | 87743755 | 0.25 |
| GTTCATCAGTACGGTGGCC | 87743739 | 87743760 | 0.01 |
| GGTGGCCTGGATGTCTTAGT | 87743751 | 87743773 | 0.96 |
| GACGGACCTACTAAGACATCC | 87743756 | 87743778 | 0.42 |
| GTTAGTAGGTCCGTCTAGCTG | 87743766 | 87743788 | 2.02 |
| GTCCGTCTAGCTGTGGTAACT | 87743774 | 87743796 | 0.01 |
| GGAGGGAGCTCTGGCACAGT | 87743798 | 87743820 | 0.39 |
| GTGCCAGAGCTCCCTCCCTA | 87743804 | 87743826 | 0.01 |
| GTAGCAGTTTGCCCATAGGGA | 87743815 | 87743837 | 0.69 |

| | | | |
|---|---|---|---|
| GCTGCTATTTTGCCTCTGCCA | 87743832 | 87743854 | 0.01 |
| GAAGCAGAGATCCCATGGCAG | 87743843 | 87743865 | 0.04 |
| GGGGGGAAGCAGAGATCCCA | 87743849 | 87743871 | 1.04 |
| GCTCTGCTTCCCCCCTCTGAA | 87743858 | 87743880 | 0.03 |
| GAGCTGAGCCATTCAGAGGG | 87743866 | 87743888 | 1.52 |
| GTAGGCAACAGAAATAACTTG | 87743888 | 87743910 | 0.17 |
| GCCTAGGTGACATCTCTAAT | 87743906 | 87743928 | 0.01 |
| GTAATCTGTTCCTCTTTTAAA | 87743938 | 87743960 | 0.00 |
| GCAGGCTTATCCATTTAAAAG | 87743947 | 87743969 | 0.01 |
| GAACCTGGAAGTAAAGCATC | 87743966 | 87743988 | 0.02 |
| GTACTTCCAGGTTCTGTCAGA | 87743976 | 87743998 | 0.01 |
| GCTAGGCCTTCTGACAGAACC | 87743981 | 87744003 | 0.16 |
| GTTCTGTCAGAAGGCCTAGAC | 87743986 | 87744008 | 0.01 |
| GAATATACTTTAGCCAGTCT | 87743999 | 87744021 | 0.06 |
| GTTGCATCTGAATGATGGCTT | 87744027 | 87744049 | 0.01 |
| GATGAACTTGCATCTGAATGA | 87744033 | 87744055 | 0.00 |
| GTTCATGAGCTAGCCCTGC | 87744050 | 87744071 | 0.01 |
| GCCCTGCTGGACAATCACAG | 87744062 | 87744084 | 2.77 |
| GCACAGTGGATCCTGAAAGCA | 87744077 | 87744099 | 0.12 |
| GATCCTGAAAGCATGGCTTC | 87744084 | 87744106 | 0.01 |
| GTAGTCTTGCTAAATCTTCAA | 87744118 | 87744140 | 0.01 |
| GTCCTGCTAGCCCTACTTCAC | 87744149 | 87744171 | 1.52 |
| GATGGGACACCAGTGAAGTA | 87744158 | 87744180 | 0.01 |
| GAACACAGGCTCTAAACTGAT | 87744175 | 87744197 | 0.03 |
| GTTTAGAGCCTGTGTTTAA | 87744182 | 87744203 | 0.38 |
| GTACAGTTGGCCTTTAAACAC | 87744190 | 87744212 | 0.02 |
| GTTAAAGGCCAACTGTAAAGC | 87744197 | 87744219 | 0.03 |
| GGCCAACTGTAAAGCAGGTT | 87744202 | 87744224 | 1.49 |
| GCAAACCAAGTCCCTGCAACC | 87744232 | 87744254 | 0.01 |
| GAAGAACTAACCCAGGTTGCA | 87744242 | 87744264 | 0.01 |
| GGAAGGAAAAGAACTAACCC | 87744250 | 87744272 | 0.02 |
| GCTTTTCCTTCCTATGTGAAC | 87744262 | 87744284 | 0.70 |
| GTTTCCCCTTCCAGTTCACAT | 87744271 | 87744293 | 0.00 |
| GGAGTTTGACATTCAAGCA | 87744296 | 87744317 | 0.00 |
| GCATTCAAGCATGGTTCTTAA | 87744305 | 87744327 | 0.01 |
| GACACTTCAGGCACATTGCCA | 87744383 | 87744405 | 3.52 |
| GCAGGCACATTGCCAAGGAGA | 87744389 | 87744411 | 0.01 |
| GCTCAGTATCATCCTTCTCCT | 87744400 | 87744422 | 0.01 |
| GTACTGAGGGGACACTTTTGC | 87744416 | 87744438 | 0.31 |
| GCTGGAATTCCTACCAGAAG | 87744434 | 87744456 | 0.08 |
| GCACTGTGCACCACTTCTGGT | 87744443 | 87744465 | 3.08 |
| GTGTTTATAAGCACACAGTG | 87744467 | 87744489 | 0.76 |
| GACATGTAAGAAAAGTAGTGT | 87744489 | 87744511 | 0.01 |
| GCATTTCTTTAGATAAGACAA | 87744515 | 87744537 | 0.01 |
| GATAAGACAAAGGGCAGAAG | 87744525 | 87744547 | 0.01 |
| GGCAGAGTTTGGAGCTGTGA | 87744552 | 87744574 | 0.02 |
| GTTCATTGAAATGGCAGAGTT | 87744563 | 87744585 | 0.01 |
| GCTGCCATTTCAATGAAGCCT | 87744570 | 87744592 | 0.01 |
| GTCAATGAAGCCTTGGGCATC | 87744578 | 87744600 | 0.01 |
| GAGGGACGTCCAGATGCCCA | 87744587 | 87744609 | 0.01 |
| GATCTGGACGTCCCTCTGCAG | 87744595 | 87744617 | 0.01 |
| GCCAGACTCGTCCTCTGCAGA | 87744605 | 87744627 | 0.72 |

| | | | |
|---|---|---|---|
| GTCTGGGAGGTTCTGTCCCT | 87744622 | 87744644 | 0.02 |
| GGTTCTGTCCCTTGGCCTG | 87744630 | 87744651 | 0.00 |
| GCTGAATGGTCCTCAGGCCAA | 87744638 | 87744660 | 0.33 |
| GACACTCTCTGAATGGTCCTC | 87744645 | 87744667 | 0.00 |
| GAAAGGAACACTCTCTGAA | 87744653 | 87744674 | 0.09 |
| GCAGAGGAGTCATAGAATGAA | 87744669 | 87744691 | 2.27 |
| GTCTATGACTCCTCTGGGTTC | 87744677 | 87744699 | 0.01 |
| GGGAGCTGCCAGAACCCAG | 87744686 | 87744707 | 0.01 |
| GGTAGAGGAGGCAGGAGGGA | 87744705 | 87744727 | 0.02 |
| GCCGGAGGTAGAGGAGGCAGG | 87744710 | 87744732 | 0.02 |
| GAATTCTACACCGGAGGTAG | 87744720 | 87744742 | 0.16 |
| GTACTGCATTGAATTCTACAC | 87744729 | 87744751 | 2.36 |
| GAATGCAGTAAACAGCCCACC | 87744743 | 87744765 | 0.04 |
| GACAGCCCACCTGGTTATTTG | 87744753 | 87744775 | 0.00 |
| GTGGTCCTCAAATAACCAGG | 87744758 | 87744780 | 0.01 |
| GATCATGTTCTAAGTCATGTG | 87744777 | 87744799 | 1.45 |
| GATCTCAAACACTATGTGGT | 87744797 | 87744819 | 0.02 |
| GCAAACACTATGTGGTGGGCA | 87744802 | 87744824 | 0.01 |
| GGTGGGCATGGGAGCTTAAG | 87744814 | 87744836 | 0.02 |
| GTGGCCTGACATTTGTAATA | 87744833 | 87744855 | 1.62 |
| GCATTTGTAATATGGCTGCTT | 87744842 | 87744864 | 0.38 |
| GATATGGCTGCTTTGGGTTTC | 87744850 | 87744872 | 0.01 |
| GTTCTGGTGCTGCAGACCCTT | 87744867 | 87744889 | 0.01 |
| GCAGACCCTTTGGGAGTAGA | 87744877 | 87744899 | 0.02 |
| GCTCTGCCGTCTACTCCCAAA | 87744882 | 87744904 | 0.30 |
| GTCCCTAATCAAAGAAGAGG | 87744904 | 87744926 | 0.02 |
| GTAATCAAAGAAGAGGTGGAG | 87744909 | 87744931 | 0.02 |
| GAAGAGGTGGAGGGGGGGGA | 87744917 | 87744939 | 0.03 |
| GGGCTCTGCTCTGCACCTCT | 87744937 | 87744959 | 3.78 |
| GCACCTCTAGGCTCTTTAGAA | 87744950 | 87744972 | 0.77 |
| GAAAGGGGAAGAATTGAGC | 87744967 | 87744988 | 0.04 |
| GGAAGAATTGAGCTGGTCAA | 87744973 | 87744995 | 0.01 |
| GCTGGTCAATGGTAGTTAAG | 87744984 | 87745006 | 0.00 |
| GTAACAAAGTCTTCTCCAAG | 87745013 | 87745035 | 0.05 |
| GCTTCTCCAAGAGGAGACAGC | 87745023 | 87745045 | 0.02 |
| GGAGACCAGCTGTCTCCTCT | 87745028 | 87745050 | 0.79 |
| GGGAGGCTTTCTATAACTAA | 87745049 | 87745071 | 0.30 |
| GACAAGCTTAATGCACACGCA | 87745069 | 87745091 | 0.01 |
| GTAAGCTTGTGAATGAAGAGA | 87745083 | 87745105 | 0.00 |
| GTGAATGAAGAGAAGGTTGA | 87745090 | 87745112 | 0.01 |
| GTCAGAGAGCCCCTCCCTGC | 87745153 | 87745175 | 0.00 |
| GCCCAGGGGGACCTGCAGGG | 87745164 | 87745186 | 0.04 |
| GTTCTACTTGCAGAAGCCCA | 87745179 | 87745201 | 0.60 |
| GCTTCTGCAAGTAGAACTTA | 87745185 | 87745207 | 0.09 |
| GCAAGTAGAACTTATGGAG | 87745191 | 87745212 | 0.01 |
| GCTTATGGAGTGGACTAGCCG | 87745201 | 87745223 | 0.00 |
| GACTAGCCGCGGGCTGAGAA | 87745212 | 87745234 | 0.32 |
| GCTGGCCTTTCTCAGCCCG | 87745218 | 87745239 | 0.01 |
| GCAAATGGTGAGGGGCTTAGC | 87745235 | 87745257 | 0.27 |
| GTTGAAAATTGCAAATGGTGA | 87745245 | 87745267 | 0.00 |
| GACATACTTGAAAATTGCAAA | 87745251 | 87745273 | 0.01 |
| GCAATTTTCAAGTATGTGTG | 87745257 | 87745279 | 2.99 |

| | | | |
|---|---|---|---|
| GTTCAAGTATGTGTGGGGGTG | 87745263 | 87745285 | 0.01 |
| GTATGTGTGGGGGTGAGGGT | 87745268 | 87745290 | 0.04 |
| GGTGGGGTATGCTGCCGCCG | 87745291 | 87745313 | 0.06 |
| GAAACACAGCACCCACCGCGG | 87745305 | 87745327 | 0.01 |
| GTGTAGTATTATGCAATTAG | 87745328 | 87745350 | 0.01 |
| GACACCATTCCCTGTGTGTAG | 87745347 | 87745369 | 0.00 |
| GTAGAACAGCCACTACACACA | 87745355 | 87745377 | 0.01 |
| GCTGTTCTATATATATACAC | 87745369 | 87745391 | 0.01 |
| GATACACAGGGAGGCAACATA | 87745383 | 87745405 | 0.12 |
| GTCACAGTTTTGACAGGTGG | 87745415 | 87745437 | 3.88 |
| GATATAGTCACAGTTTTGAC | 87745421 | 87745443 | 0.38 |
| GACGACCAGAAGCAAGCTGCT | 87745451 | 87745473 | 0.07 |
| GCAGCCTTTAGAATACTGGCT | 87745474 | 87745496 | 0.01 |
| GCTCCTCAGCCTTTAGAATAC | 87745479 | 87745501 | 0.00 |
| GTAATTCAGAGCCATGAAAGG | 87745503 | 87745525 | 0.01 |
| GCATATGGTGCCGCCTTTCA | 87745513 | 87745535 | 0.07 |
| GAAAGGCGGCACCATATGCTT | 87745518 | 87745540 | 0.78 |
| GCTGGCAACCCCAAAGCATA | 87745528 | 87745550 | 0.01 |
| GCCAGCTGCTTATGTGCATG | 87745545 | 87745567 | 0.00 |
| GCATCCTGGCTTTCGCAGAC | 87745580 | 87745602 | 1.27 |
| GAAGAGAGTGAGTGCATCC | 87745594 | 87745615 | 1.56 |
| GCTCTTCGCTGCAGTATGCAC | 87745610 | 87745632 | 0.00 |
| GCTGCAGTATGCACTGGCG | 87745616 | 87745637 | 0.01 |
| GTATGCACTGGCGCGGAGCG | 87745622 | 87745644 | 0.00 |
| GGCGCGGAGCGGGGAATTTG | 87745631 | 87745653 | 0.29 |
| GGTGCCACTTTTTCTCCTTC | 87745652 | 87745674 | 0.03 |
| GCAACAAATTTCCCCTCCAGA | 87745667 | 87745689 | 0.04 |
| GGGGGCCACGATTCTCCAAG | 87745689 | 87745711 | 0.00 |
| GTTTTTTTTTGAGTCCCCTCT | 87745704 | 87745726 | 0.02 |
| GAACTTGCCCTTGATAACGTC | 87745743 | 87745765 | 0.01 |
| GCCCTTGATAACGTCAGGCC | 87745748 | 87745770 | 0.01 |
| GTAAAGAAATACTATTGGGCC | 87745766 | 87745788 | 0.01 |
| GTTTTCTAAAGAAATACTAT | 87745772 | 87745794 | 0.01 |
| GAAAACATGTTTGTTTTGG | 87745789 | 87745810 | 0.02 |
| GTTTTGGTGGTTGTTTTTT | 87745801 | 87745822 | 0.00 |
| GTTTTTTGGTTTCGTTTTTAT | 87745815 | 87745837 | 0.01 |
| GATTAAGAAAGCGCCATCT | 87745843 | 87745864 | 0.82 |
| GCTTCACTTCTCTCCGAGA | 87745856 | 87745877 | 0.44 |
| GAAGTGAAGCAAACCCGCC | 87745868 | 87745889 | 0.01 |
| GGGGCTGCGCGCCGCCGGGC | 87745881 | 87745903 | 0.04 |
| GGCGGCGCGCAGCCCCGGCC | 87745888 | 87745910 | 0.01 |
| GGCCCGGTCCGCGACGCTGG | 87745904 | 87745926 | 0.01 |
| GCCGCCCCCAGCGTCGCGGAC | 87745907 | 87745929 | 0.01 |
| GCCGCCCGCCCCCAGCGTCG | 87745912 | 87745934 | 0.01 |
| GGGCGGGCGGCCGCTCTACC | 87745924 | 87745946 | 1.46 |
| GCCGCTCTACCTGGAGGCGC | 87745933 | 87745955 | 0.01 |
| GGCAGAGACCAGCGCCTCC | 87745942 | 87745963 | 0.00 |
| GGCGCTGGTCTCTGCCAGC | 87745948 | 87745969 | 0.03 |
| GGGCATCATGAACAACCGGC | 87745962 | 87745984 | 0.01 |
| GTCGGGCGGCGTGGGCGCGCT | 87745982 | 87746004 | 0.11 |
| GAACGGCTTCTCGGGCGGCG | 87745992 | 87746014 | 0.01 |
| GACGCGACAGAACGGCTTCTC | 87746000 | 87746022 | 0.05 |

| | | | |
|---|---|---|---|
| GCGTCAACACGCGACAGAA | 87746009 | 87746030 | 1.24 |
| GTGTTGACGCACACGCTGCG | 87746021 | 87746043 | 0.00 |
| GCACGCTGCGCGGGTAGCCGT | 87746032 | 87746054 | 0.01 |
| GGGTAGCCGTTGGCCGTCT | 87746042 | 87746063 | 0.01 |
| GCAGCTGTCCGAGACGGCCAA | 87746048 | 87746070 | 0.00 |
| GCAGAAGCAGCTGTCCGAGA | 87746055 | 87746077 | 0.32 |
| GCAGCGCCAGCGACACCTTGT | 87746080 | 87746102 | 1.69 |
| GGCGTCCAACAAGGTGTCGC | 87746085 | 87746107 | 0.32 |
| GACACCTTGTTGGACGCCGTG | 87746091 | 87746113 | 0.40 |
| GCATCTCTATGCGCGACCTCA | 87746106 | 87746128 | 0.01 |
| GTAGAGATGAGCTCGCCCGAC | 87746121 | 87746143 | 0.04 |
| GAGCTCGCCCGACAGGCGG | 87746128 | 87746149 | 0.16 |
| GCCCGACAGGCGGCGGCCCT | 87746134 | 87746156 | 0.00 |
| GACTCGCGACAGCGATGCCG | 87746151 | 87746173 | 0.01 |
| GCTGTCGCGAGTCGCGGGGT | 87746161 | 87746183 | 0.02 |
| GGTCGGCACCCAGCGCGGCC | 87746178 | 87746200 | 0.01 |
| GCGCCGTCGCCTGGCCGCGC | 87746187 | 87746209 | 0.01 |
| GGCCAGGCGACGGCGCAGCC | 87746194 | 87746216 | 0.11 |
| GACGGCGCAGCCGGGAGCCC | 87746202 | 87746224 | 0.49 |
| GCAGCCGGGAGCCCGGGGTGA | 87746209 | 87746231 | 0.02 |
| GCCGGCGCAACGCCATCACCC | 87746220 | 87746242 | 0.01 |
| GATGGCGTTGCGCCGGGCTA | 87746227 | 87746249 | 0.01 |
| GCGCCGGGCTAGGGGCGCGC | 87746236 | 87746258 | 0.04 |
| GCTAGGGGCGCGCCGGGAGC | 87746243 | 87746265 | 0.35 |
| GACGCGCTGCTGCCCGGCTCC | 87746255 | 87746277 | 0.35 |
| GGCAGCAGCGCGTGCAGCAG | 87746265 | 87746287 | 0.61 |
| GTCATCAAGCAGGTGCTCAAC | 87746308 | 87746330 | 2.52 |
| GAGCACCTGCTTGATGAGGA | 87746314 | 87746336 | 0.02 |
| GCATCTCCATCCTCATCAAGC | 87746319 | 87746341 | 0.07 |
| GATGCAGCACACGCCGAGC | 87746359 | 87746380 | 0.01 |
| GACACGCCGAGCAGGATGAAG | 87746367 | 87746389 | 0.02 |
| GAACTTCCTCTTCATCCTGCT | 87746372 | 87746394 | 0.21 |
| GGATGAAGAGGAAGTTGCCC | 87746379 | 87746401 | 0.97 |
| GAACCAGGGGCTCTACCGCC | 87746397 | 87746419 | 0.12 |
| GTAGAGCCCCTGGTTCCGGT | 87746404 | 87746426 | 0.02 |
| GACGCCGCCTACCGGAACCAG | 87746410 | 87746432 | 0.04 |
| GGTTCCGGTAGGCGGCGTGT | 87746415 | 87746437 | 0.31 |
| GGCGTGTTGGCTGCTCACC | 87746428 | 87746449 | 0.31 |
| GCACCAGGTCCCCGAAGCCGA | 87746443 | 87746465 | 0.07 |
| GCTTCAGCACCATCGGCTTCG | 87746451 | 87746473 | 1.60 |
| GTTCGTCACCTTCAGCACCAT | 87746459 | 87746481 | 0.15 |
| GTAGTCCCAGCCTTCCACGC | 87746503 | 87746525 | 0.07 |
| GTACACCAGCGTGGAAGGCT | 87746508 | 87746530 | 0.01 |
| GCTCGGCCATGTACACCAGCG | 87746517 | 87746539 | 0.01 |
| GTACATGGCCGAGGCGCAAC | 87746527 | 87746549 | 0.03 |
| GCTGCTGGCCTGTTGCGCCT | 87746535 | 87746557 | 0.00 |
| GGGCCTGTTCGCCGTGCTGC | 87746550 | 87746572 | 0.07 |
| GGCCCAGGATGAGCAGCACG | 87746568 | 87746590 | 0.02 |
| GAGCAGCACGTGGTACACCG | 87746578 | 87746600 | 0.03 |
| GCCTGGCGGGCTGGAAGCCCT | 87746595 | 87746617 | 0.02 |
| GCTTCCAGCCCGCCAGGCTGT | 87746602 | 87746624 | 0.01 |
| GTCGGAGGCCGACAGCCTGG | 87746610 | 87746632 | 0.16 |

| | | | |
|---|---|---|---|
| GCCGCGGCTCGGCGCTGTCGG | 87746625 | 87746647 | 1.50 |
| GCAGCGCCGAGCCGCGGCGGA | 87746632 | 87746654 | 0.02 |
| GAGCCGCGGCGGAAGGTGGC | 87746639 | 87746661 | 0.00 |
| GGCGGAAGGTGGCGGGCAGC | 87746646 | 87746668 | 0.01 |
| GGCGGGCAGCAGGCCACTG | 87746656 | 87746677 | 0.01 |
| GCAGGCCACTGCGGCGCAGC | 87746664 | 87746686 | 0.02 |
| GAGCGCCAGCTGCGCCGCAG | 87746669 | 87746691 | 1.84 |
| GCGCAGCTGGCGCTCCCGGC | 87746677 | 87746699 | 0.02 |
| GCTTCATCATGCGCGCCTGCC | 87746691 | 87746713 | 0.11 |
| GAGCGCATCATCTCGCTGC | 87746715 | 87746736 | 2.05 |
| GATGATGCGCTCTAGAAAG | 87746725 | 87746746 | 0.83 |
| GAAAGAGGTTGAAGAACAAGA | 87746740 | 87746762 | 0.01 |
| GACGGACTGTTCGGCTGCGCG | 87746764 | 87746786 | 0.07 |
| GCTCATCGCCTACGGACTGTT | 87746774 | 87746796 | 0.01 |
| GCAGTCCGTAGGCGATGAGGA | 87746779 | 87746801 | 0.04 |
| GAAGGCCTTCCCGCCCACTG | 87746797 | 87746819 | 0.39 |
| GCAACACCAGCCACAGTGGGC | 87746806 | 87746828 | 0.15 |
| GTTGTCATGCCGAAACCTG | 87746825 | 87746846 | 0.34 |
| GCTGTTATCTCCGCAGGTTT | 87746834 | 87746856 | 0.01 |
| GATAACAGCCAGGGTTAGAG | 87746848 | 87746870 | 0.00 |
| GCAGCCAGGGTTAGAGAGGGC | 87746853 | 87746875 | 0.90 |
| GCTGGTGGTCGCATGTGGTG | 87746871 | 87746893 | 0.74 |
| GTGGTCGCATGTGGTGTGGC | 87746875 | 87746897 | 0.01 |
| GTGGCAGGCGAATGCAGATG | 87746890 | 87746912 | 0.00 |
| GCTGACCGACTTAAGTCAGG | 87746926 | 87746948 | 0.08 |
| GACCGACTTAAGTCAGGTGG | 87746929 | 87746951 | 0.01 |
| GGGGCGGGACAGTACTTAGA | 87746948 | 87746970 | 3.29 |
| GACTTAGACGGTTCCCTCTTT | 87746961 | 87746983 | 0.68 |
| GATCAAGAGCCACCAAAAGA | 87746973 | 87746995 | 0.01 |
| GCATGATGAATTCCAACAAGA | 87746995 | 87747017 | 0.03 |
| GGGCAGAACTCCATCTTGT | 87747006 | 87747027 | 0.01 |
| GGAGTTCTGCCCTATGCCTT | 87747016 | 87747038 | 0.37 |
| GTTAAATACCCCTAAGGCATA | 87747025 | 87747047 | 0.02 |
| GAGTAGTTTAAATACCCCTA | 87747032 | 87747054 | 2.79 |
| GTTTAAACTACTCAGGTAGAG | 87747043 | 87747065 | 0.00 |
| GCAGGTAGAGCGGATATGCAC | 87747054 | 87747076 | 0.01 |
| GATGCACTGGACGAGACCAAT | 87747068 | 87747090 | 0.06 |
| GCTGCTCAGTGCATCCTAT | 87747083 | 87747104 | 1.63 |
| GGATGCACTGAGCAGCACC | 87747089 | 87747110 | 0.24 |
| GAGGTTTGCATCTGCGCACC | 87747106 | 87747128 | 0.00 |
| GCTGGTTTGTGGGCAGCTCAG | 87747125 | 87747147 | 2.19 |
| GGTGTCATTCACTGGTTTGT | 87747136 | 87747158 | 0.00 |
| GGGGAACAGGTGTCATTCAC | 87747144 | 87747166 | 0.01 |
| GGAAATTCAGTTAGGGGAAC | 87747157 | 87747179 | 0.02 |
| GTCCAGAGGAAATTCAGTTA | 87747164 | 87747186 | 16.64 |
| GAATTTCCTCTGGACTCTGT | 87747172 | 87747194 | 0.01 |
| GTCTGGACTCTGTCGGCAGAC | 87747180 | 87747202 | 0.45 |
| GCTGTCGGCAGACAGGCTTGT | 87747188 | 87747210 | 0.02 |
| GGCAGACAGGCTTGTTGGGC | 87747193 | 87747215 | 0.06 |
| GCTTCCAACATCTTGTAATT | 87747216 | 87747238 | 1.31 |
| GACAGGCAATTCTCTCTCACT | 87747254 | 87747276 | 0.17 |
| GAGAATTGCCTGTTAGCTC | 87747264 | 87747285 | 0.01 |

| | | | |
|---|---|---|---|
| GTAAGTGAACCAGAGCTAAC | 87747272 | 87747294 | 0.02 |
| GTCACTTACGAAGCCCACACA | 87747287 | 87747309 | 0.03 |
| GCCCACACAAGGATCATTTG | 87747298 | 87747320 | 0.56 |
| GTCTAAAGGCAGGAGGGGAAC | 87747322 | 87747344 | 0.01 |
| GTTTGCACTCTAAAGGCAGG | 87747330 | 87747352 | 0.00 |
| GTCCAGGTTTGCACTCTAA | 87747337 | 87747358 | 0.77 |
| GCAAACCTGGACATCTGTCAC | 87747348 | 87747370 | 0.01 |
| GACATCTGTCACTGGACTTC | 87747356 | 87747378 | 0.01 |
| GGACTTCAGGTCCCCCTCC | 87747369 | 87747390 | 0.01 |
| GAAACATGGTGGTCCTGGAGG | 87747380 | 87747402 | 0.26 |
| GTAGACCAAACATGGTGGTCC | 87747386 | 87747408 | 0.00 |
| GGGCCCAGCTAGACCAAACA | 87747395 | 87747417 | 0.55 |
| GTTTGGTCTAGCTGGGCCCTG | 87747400 | 87747422 | 0.11 |
| GGCCCTGTGGTGCCAGTATG | 87747413 | 87747435 | 0.00 |
| GACAAAACTCTAACCACATAC | 87747425 | 87747447 | 0.01 |
| GGTTAGAGTTTTGTCCTGGA | 87747434 | 87747456 | 0.01 |
| GAGCATGGGAGAGACCTTCC | 87747448 | 87747470 | 0.18 |
| GCTAAGCAGCTTTCCGAGCAT | 87747462 | 87747484 | 0.01 |
| GTGCTTTGAATCCTTTAATA | 87747484 | 87747506 | 0.01 |
| GACAGGTTACCTCCTTATTAA | 87747495 | 87747517 | 0.01 |
| GCTGGCTTTCAACAAGTGGAC | 87747513 | 87747535 | 4.31 |
| GTTGAAAGCCAGGCCCTGAA | 87747524 | 87747546 | 0.01 |
| GGCCCTGAAGGGTTTGTTAA | 87747535 | 87747557 | 0.01 |
| GAATGGACTCCCACAGAAAGC | 87747553 | 87747575 | 0.01 |
| GCCGTGGCAGCCTGCTTTCTG | 87747562 | 87747584 | 0.03 |
| GAAGCAGGCTGCCACGGGGTG | 87747569 | 87747591 | 0.01 |
| GGCTGCCACGGGGTGCGGGT | 87747574 | 87747596 | 7.08 |
| GTTCTCCCCACCCGCACCCCG | 87747579 | 87747601 | 0.33 |
| GATTAGACATCTCCGCATCCA | 87747650 | 87747672 | 0.00 |
| GAAGGTCTGCTCCCGTGGATG | 87747661 | 87747683 | 0.00 |
| GCCTCTGAAGGTCTGCTCCCG | 87747667 | 87747689 | 0.01 |
| GGGAGTGGTGTGCCTCTGA | 87747680 | 87747701 | 0.01 |
| GTCTGTTGGGTGTGAAGGGAG | 87747694 | 87747716 | 0.01 |
| GCCTTTTCTGTTGGGTGTGAA | 87747699 | 87747721 | 0.01 |
| GACGGGTTGTCCTTTTCTGTT | 87747708 | 87747730 | 0.01 |
| GCTGCTGCACAGCAGCGTGCA | 87747727 | 87747749 | 0.02 |
| GTGCAGCAGGCCCAGCTCT | 87747741 | 87747762 | 0.01 |
| GCTTGACTTCTTCCTAGAGCT | 87747751 | 87747773 | 0.03 |
| GCAAGGATACTTTGTCCCATT | 87747770 | 87747792 | 0.02 |
| GAAGGAGATAATAAGCCAAAT | 87747784 | 87747806 | 0.02 |
| GTATGCTCTCTGGAATAGTTA | 87747803 | 87747825 | 0.53 |
| GTGGTAGGTGTATGCTCTC | 87747814 | 87747835 | 0.01 |
| GAATGTGCTCTTCAAGTGGT | 87747828 | 87747850 | 0.01 |
| GTGATTAGCATCTAAGCCTA | 87747850 | 87747872 | 0.01 |
| GTAATCACGTTCTTGATTCCC | 87747866 | 87747888 | 0.12 |
| GCAACTTGGGCACTTCCTCC | 87747884 | 87747906 | 1.37 |
| GATAAGATGAACAGGCAACTT | 87747897 | 87747919 | 2.79 |
| GTCAGTAAGCATAAGATGAAC | 87747906 | 87747928 | 0.01 |
| GATGCTTACTGACTGGAGTTC | 87747918 | 87747940 | 0.01 |
| GATATTGCTCATTGAGAAAGA | 87747953 | 87747975 | 0.02 |
| GTATTCTTTGCGTTCCACATT | 87747978 | 87748000 | 0.01 |
| GAATTCTTTTGTGGCCAAATG | 87747991 | 87748013 | 0.01 |

| | | | |
|---|---|---|---|
| GCTTCTTTTAAAATTCTTTTG | 87748001 | 87748023 | 0.00 |
| GCATAACAAAGAAAATGTTAG | 87748037 | 87748059 | 0.00 |
| GTTGCCCAGAAATTTGTAAGA | 87748061 | 87748083 | 0.05 |
| GTAAAGCCAAACTCAAGTCCC | 87748098 | 87748120 | 0.01 |
| GAAACTCAAGTCCCTGGGCTC | 87748105 | 87748127 | 0.01 |
| GTCGATTTCTTCCTGAGCCC | 87748116 | 87748138 | 0.01 |
| GAAGAAATCGACACTGTTTG | 87748127 | 87748149 | 0.01 |
| GAATCGACACTGTTTGTGGAT | 87748132 | 87748154 | 0.03 |
| GTTTGTGGATTGGAGTATGT | 87748142 | 87748164 | 0.01 |
| GATTGGAGTATGTGGGGGGG | 87748149 | 87748171 | 0.02 |
| GTAGAAGTTCAAAATCATCTT | 87748242 | 87748264 | 0.01 |
| GTCTTTGGCTACATAGTAAGA | 87748258 | 87748280 | 0.01 |
| GGGTCTCCTATATCCTAGGC | 87748284 | 87748306 | 0.02 |
| GAAATTTAGAAAGTCCTAGAC | 87748324 | 87748346 | 0.03 |
| GAAAGTCCTAGACTGGAGGC | 87748331 | 87748353 | 0.01 |
| GCTTCTCCAGCCTCCAGTCT | 87748337 | 87748359 | 0.00 |
| GGGACTACAGGTACTCAGAT | 87748382 | 87748404 | 0.21 |
| GAGTACCTGTAGTCCCTAAA | 87748389 | 87748411 | 0.01 |
| GAGGGGCTGCTCCCCCATTT | 87748403 | 87748425 | 1.90 |
| GCCATAGGCTGCAGCTTAGA | 87748425 | 87748447 | 0.02 |
| GGGCATCTTACAGCAGCCAT | 87748440 | 87748462 | 0.01 |
| GCTGCTGTAAGATGCCCCCAT | 87748447 | 87748469 | 0.01 |
| GCATGGTAGAAGCTCCGATGG | 87748460 | 87748482 | 0.00 |
| GGTGGCACCTGTTAAAATGG | 87748482 | 87748504 | 0.02 |
| GACAGGAGCCTCCATTTTAAC | 87748489 | 87748511 | 0.01 |
| GAAATGGAGGCTCCTGTAAAA | 87748496 | 87748518 | 1.37 |
| GTCATGGTGCTGCCATTTTAC | 87748507 | 87748529 | 0.00 |
| GCAGACTAAGGAGGTGTCA | 87748524 | 87748545 | 0.01 |
| GTGATGTCTGCAGACTAAGG | 87748532 | 87748554 | 0.01 |
| GCTGAGTCATGAAAGGAGCA | 87748564 | 87748586 | 0.02 |
| GTCCTAGGCTGAGTCATGAA | 87748571 | 87748593 | 0.14 |
| GGTATTTCACGAAGGTCCT | 87748586 | 87748607 | 0.02 |
| GCCTGCCGTGGTATTTCACGA | 87748593 | 87748615 | 0.13 |
| GCTGTGGGGCAGCTCCTGCCG | 87748606 | 87748628 | 1.47 |
| GCCACTGTGGACAGCTTCTG | 87748623 | 87748645 | 0.02 |
| GCTAGGGAGGCTGGAGAGG | 87748650 | 87748671 | 0.07 |
| GTAGCTGAGGCACCATCTCTG | 87748676 | 87748698 | 0.02 |
| GATGGTGCCTCAGCTAAAGC | 87748683 | 87748705 | 0.01 |
| GATGCAGCCTGCTTTAGCTG | 87748690 | 87748712 | 0.01 |
| GAAAGCAGGCTGCATCCGGGA | 87748698 | 87748720 | 0.03 |
| GAGAGTGGGAAGTCCTTCC | 87748712 | 87748733 | 0.06 |
| GGACTTCCCACTCTCATCCA | 87748719 | 87748741 | 0.73 |
| GTTGGGCCCATGGATGAGAGT | 87748725 | 87748747 | 0.46 |
| GCTCATCCATGGGCCCAATGC | 87748731 | 87748753 | 0.01 |
| GCCCAATGCAGGGAAAAAGA | 87748742 | 87748764 | 1.94 |
| GCAGGGAAAAAGAAGGTGTG | 87748749 | 87748771 | 0.01 |
| GAAAAAGAAGGTGTGGGGGGG | 87748755 | 87748777 | 1.12 |
| GTTCTCTGCTCAGGGATTGA | 87748782 | 87748804 | 0.01 |
| GATTGGTGGGTTCTCTGCTC | 87748791 | 87748813 | 0.01 |
| GAGCTCTCTTGCTCAGGGAT | 87748808 | 87748830 | 0.01 |
| GTTGGTGAGCTCTCTTGCTCA | 87748813 | 87748835 | 0.01 |
| GAGCTCACCAATCCCTGAGC | 87748825 | 87748847 | 0.02 |

| | | | |
|---|---|---|---|
| GTTGATGGGTTCTCCTGCTCA | 87748837 | 87748859 | 0.02 |
| GTCTCTTGCTCAGGGATTGAT | 87748852 | 87748874 | 0.01 |
| GTTGATAGGTTCTCTTGCTCA | 87748861 | 87748883 | 0.00 |
| GAACCTATCAATCGCTGAGC | 87748873 | 87748895 | 0.01 |
| GTTTTCCTGCTGGGGATTGG | 87748901 | 87748923 | 0.01 |
| GATTGGTGGGTTTTCCTGCT | 87748910 | 87748932 | 0.15 |
| GATTGATGGGTTCTCCTGCT | 87748958 | 87748980 | 0.02 |
| GAACCCATCAATCCCTGAGC | 87748968 | 87748990 | 0.22 |
| GTTCTCCTGCTGGGGATTGG | 87748996 | 87749018 | 0.01 |
| GAGCAGGAGAACCCATCTGT | 87749031 | 87749053 | 0.01 |
| GGGGGCGGCTCCCAACAGAT | 87749042 | 87749064 | 0.06 |
| GAAGGGCATCGACTTCAAGG | eGFP_control1 | eGFP_control1 | 18.00 |
| GAGCTGGACGGCGACGTAAA | eGFP_control10 | eGFP_control10 | 8.74 |
| GAGGAGGACGGCAACATCCTG | eGFP_control11 | eGFP_control11 | 18.03 |
| GATCCGCCACAACATCGAGGA | eGFP_control12 | eGFP_control12 | 14.60 |
| GATGCCGTTCTTCTGCTTGT | eGFP_control13 | eGFP_control13 | 40.41 |
| GATGTGATCGCGCTTCTCGTT | eGFP_control14 | eGFP_control14 | 17.37 |
| GCAACATCCTGGGGCACAAGC | eGFP_control15 | eGFP_control15 | 36.82 |
| GCAACGAGAAGCGCGATCACA | eGFP_control16 | eGFP_control16 | 23.77 |
| GCAACTACAAGACCCGCGCCG | eGFP_control17 | eGFP_control17 | 32.11 |
| GCAAGATCCGCCACAACATCG | eGFP_control18 | eGFP_control18 | 27.30 |
| GCAAGGAGGACGGCAACATCC | eGFP_control19 | eGFP_control19 | 32.67 |
| GAAGGGCGAGGAGCTGTTCAC | eGFP_control2 | eGFP_control2 | 48.93 |
| GCAAGTTCAGCGTGTCCGGCG | eGFP_control20 | eGFP_control20 | 21.35 |
| GCACGGGGCCGTCGCCGATG | eGFP_control21 | eGFP_control21 | 54.08 |
| GCACTGCACGCCGTAGGTC | eGFP_control22 | eGFP_control22 | 22.17 |
| GCAGCACGGGGCCGTCGCCGA | eGFP_control23 | eGFP_control23 | 13.72 |
| GCAGCCACAACGTCTATATCA | eGFP_control24 | eGFP_control24 | 30.57 |
| GCAGCGTGTCCGGCGAGGGCG | eGFP_control25 | eGFP_control25 | 18.54 |
| GCATGCCGAGAGTGATCCCGG | eGFP_control26 | eGFP_control26 | 11.16 |
| GCCAGGGCACGGGCAGCTTGC | eGFP_control27 | eGFP_control27 | 29.04 |
| GCCCATCCTGGTCGAGCTGGA | eGFP_control28 | eGFP_control28 | 87.00 |
| GCCGAGAGTGATCCCGGCGG | eGFP_control29 | eGFP_control29 | 9.45 |
| GAAGTTCAGCGTGTCCGGCGA | eGFP_control3 | eGFP_control3 | 19.04 |
| GCCGATGGGGGTGTTCTGC | eGFP_control30 | eGFP_control30 | 22.82 |
| GCCGCGCCGAGGTGAAGTTCG | eGFP_control31 | eGFP_control31 | 22.97 |
| GCCGGCAAGCTGCCCGTGCCC | eGFP_control32 | eGFP_control32 | 29.69 |
| GCCGTCCAGCTCGACCAGGA | eGFP_control33 | eGFP_control33 | 14.19 |
| GCCGTCCAGCTCGACCAGGAT | eGFP_control34 | eGFP_control34 | 26.48 |
| GCCGTCCTCGATGTTGTGG | eGFP_control35 | eGFP_control35 | 55.44 |
| GCCGTCGTCCTTGAAGAAGA | eGFP_control36 | eGFP_control36 | 39.51 |
| GCCTCGAACTTCACCTCGGCG | eGFP_control37 | eGFP_control37 | 23.11 |
| GCGAGGAGCTGTTCACCGGGG | eGFP_control38 | eGFP_control38 | 11.27 |
| GCGCCGGGATCACTCTCGGCA | eGFP_control39 | eGFP_control39 | 11.24 |
| GAAGTTCGAGGGCGACACCC | eGFP_control4 | eGFP_control4 | 32.90 |
| GCGCGATCACATGGTCCTGC | eGFP_control40 | eGFP_control40 | 39.22 |
| GCGCGCCGAGGTGAAGTTCGA | eGFP_control41 | eGFP_control41 | 31.07 |
| GCTCCTGGACGTAGCCTTC | eGFP_control42 | eGFP_control42 | 43.29 |
| GCTCGAACTTCACCTCGGCGC | eGFP_control43 | eGFP_control43 | 12.06 |
| GCTCGTTGGGGTCTTTGCTCA | eGFP_control44 | eGFP_control44 | 19.24 |
| GCTGAAGCACTGCACGCCGT | eGFP_control45 | eGFP_control45 | 19.96 |
| GCTGAAGGGCATCGACTTCA | eGFP_control46 | eGFP_control46 | 27.60 |

| | | | |
|---|---|---|---|
| GCTGAAGTTCATCTGCACCAC | eGFP_control47 | eGFP_control47 | 10.84 |
| GCTGCACGCCGTAGGTCAGGG | eGFP_control48 | eGFP_control48 | 19.11 |
| GCTGCCGTCCTCGATGTTG | eGFP_control49 | eGFP_control49 | 35.13 |
| GACCAGGATGGGCACCACCC | eGFP_control5 | eGFP_control5 | 5.39 |
| GCTGGACGTAGCCTTCGGGCA | eGFP_control50 | eGFP_control50 | 17.71 |
| GCTTCAGGGTCAGCTTGCCGT | eGFP_control51 | eGFP_control51 | 7.92 |
| GCTTCATGTGGTCGGGGTAG | eGFP_control52 | eGFP_control52 | 74.27 |
| GGAGCGCACCATCTTCTTCA | eGFP_control53 | eGFP_control53 | 54.72 |
| GGCATCGACTTCAAGGAGGA | eGFP_control54 | eGFP_control54 | 26.72 |
| GGCATCGCCCTCGCCCTCGC | eGFP_control55 | eGFP_control55 | 24.97 |
| GGCCATGATATAGACGTTG | eGFP_control56 | eGFP_control56 | 37.88 |
| GGCGGTCACGAACTCCAGC | eGFP_control57 | eGFP_control57 | 24.14 |
| GGGCACGGGCAGCTTGCCGG | eGFP_control58 | eGFP_control58 | 46.07 |
| GGGCGAGGAGCTGTTCACCG | eGFP_control59 | eGFP_control59 | 37.01 |
| GACCATCTTCTTCAAGGACGA | eGFP_control6 | eGFP_control6 | 12.80 |
| GGTCAGGGTGGTCACGAGGG | eGFP_control60 | eGFP_control60 | 11.20 |
| GGTCTTTGCTCAGGGCGGAC | eGFP_control61 | eGFP_control61 | 26.18 |
| GGTGAACCGCATCGAGCTGA | eGFP_control62 | eGFP_control62 | 9.35 |
| GGTGCTCAGGTAGTGGTTGT | eGFP_control63 | eGFP_control63 | 21.36 |
| GGTGGTCACGAGGGTGGGCC | eGFP_control64 | eGFP_control64 | 1.82 |
| GGTTGTCGGGCAGCAGCACG | eGFP_control65 | eGFP_control65 | 29.91 |
| GTACCAGCAGAACACCCCCAT | eGFP_control66 | eGFP_control66 | 26.29 |
| GTAGGTCAGGGTGGTCACG | eGFP_control67 | eGFP_control67 | 31.87 |
| GTAGGTCAGGGTGGTCACGA | eGFP_control68 | eGFP_control68 | 25.76 |
| GTCAGCTCGATGCGGTTCACC | eGFP_control69 | eGFP_control69 | 16.02 |
| GACGAGGGTGGGCCAGGGCAC | eGFP_control7 | eGFP_control7 | 14.87 |
| GTCAGGGCGGACTGGGTGCTC | eGFP_control70 | eGFP_control70 | 10.63 |
| GTCGCCCTCGAACTTCACCT | eGFP_control71 | eGFP_control71 | 26.80 |
| GTCGCCGTCCAGCTCGACC | eGFP_control72 | eGFP_control72 | 56.93 |
| GTCGTGCTGCTTCATGTGGT | eGFP_control73 | eGFP_control73 | 15.65 |
| GTCGTGCTGCTTCATGTGGTC | eGFP_control74 | eGFP_control74 | 1.23 |
| GTGAACCGCATCGAGCTGAA | eGFP_control75 | eGFP_control75 | 11.28 |
| GTGGAGTTCGTGACCGCCGCC | eGFP_control76 | eGFP_control76 | 37.57 |
| GTTCAAGTCCGCCATGCCCGA | eGFP_control77 | eGFP_control77 | 42.60 |
| GACGTAGCCTTCGGGCATGG | eGFP_control8 | eGFP_control8 | 33.24 |
| GAGCGTGTCCGGCGAGGGCGA | eGFP_control9 | eGFP_control9 | 31.93 |
| GAAAACGTACGCGCGTCATAT | Neg_control | Neg_control | 0.03 |
| GAACCGCGACTATCGCGCGAT | Neg_control | Neg_control | 0.01 |
| GAACGGCGACGTACTTCGACG | Neg_control | Neg_control | 0.01 |
| GAACGGGGTCGATTCGGTACG | Neg_control | Neg_control | 0.00 |
| GAAGGTCGACGCATATCGACG | Neg_control | Neg_control | 0.00 |
| GAATACGTTTCGTACGGTCGA | Neg_control | Neg_control | 0.00 |
| GAATCGACTAACGCGCCGCG | Neg_control | Neg_control | 0.35 |
| GAATTCGTGGCGTACGACGTA | Neg_control | Neg_control | 0.01 |
| GACACGTACGCGATCCGTAT | Neg_control | Neg_control | 0.00 |
| GACATCACGCGTTACGTAACG | Neg_control | Neg_control | 0.00 |
| GACCGAAACCCGCGTTATACG | Neg_control | Neg_control | 0.01 |
| GACCGCTTTCGCGTATATCGC | Neg_control | Neg_control | 0.01 |
| GACCGGACGATACGCGACGT | Neg_control | Neg_control | 0.01 |
| GACCGTGCGCGTAACGACGCG | Neg_control | Neg_control | 0.00 |
| GACGATACGCGACGAACGCGT | Neg_control | Neg_control | 0.01 |
| GACGCCGATTATCGTACGACG | Neg_control | Neg_control | 0.01 |

| | | | |
|---|---|---|---|
| GACGGCCATATTATCGCGTCG | Neg_control | Neg_control | 0.01 |
| GACGTAAAGCGCGGTTCGGTA | Neg_control | Neg_control | 0.00 |
| GACGTACGCGTATCGCGACGG | Neg_control | Neg_control | 0.01 |
| GACGTCACTATACGCGGTTCG | Neg_control | Neg_control | 0.00 |
| GACGTCGTGCGACGCGTATCG | Neg_control | Neg_control | 0.06 |
| GACTCCGTCGCGCGATATTA | Neg_control | Neg_control | 0.00 |
| GATACTGTCGTCGTAACGC | Neg_control | Neg_control | 0.01 |
| GATAGATCCGCGACGAACGCG | Neg_control | Neg_control | 0.01 |
| GATAGATTGACGCGACGTATA | Neg_control | Neg_control | 0.02 |
| GATAGGTCCGTTCGCGATACG | Neg_control | Neg_control | 0.07 |
| GATCAACGTCGCGATACGA | Neg_control | Neg_control | 0.00 |
| GATCAATTTTACGCGCCGTAT | Neg_control | Neg_control | 0.01 |
| GATCATTCGCGTAATCGGCGG | Neg_control | Neg_control | 0.01 |
| GATCGGATATCGCGCGAATAG | Neg_control | Neg_control | 0.00 |
| GATCGGTTATTCGGCGATACG | Neg_control | Neg_control | 0.01 |
| GATCGTACGTAGTCGCGCGAA | Neg_control | Neg_control | 0.00 |
| GATCGTCAACGGTCGAACCGT | Neg_control | Neg_control | 0.01 |
| GATGGCGTAATCGGTACGCGT | Neg_control | Neg_control | 0.04 |
| GATTACCGGCGCGATACGTAT | Neg_control | Neg_control | 6.53 |
| GATTATCCGTCGACCCGCGAT | Neg_control | Neg_control | 1.26 |
| GATTATCGTCGACCGGTCGTA | Neg_control | Neg_control | 0.01 |
| GATTTGACTGCGCGAATACGA | Neg_control | Neg_control | 0.01 |
| GATTTGATAGCGCGTCGAT | Neg_control | Neg_control | 0.00 |
| GCAAACGCGACGATCGAAC | Neg_control | Neg_control | 0.01 |
| GCAACCGACCGCGATATCGTA | Neg_control | Neg_control | 0.00 |
| GCAACGATCTATCGTCGCGTA | Neg_control | Neg_control | 0.01 |
| GCACATCCGTTGCGCGTAT | Neg_control | Neg_control | 0.02 |
| GCACGGGTACGATCGCGGT | Neg_control | Neg_control | 0.01 |
| GCACTATAACGCGACGGATAT | Neg_control | Neg_control | 0.00 |
| GCATAATACTCGATATCGCGT | Neg_control | Neg_control | 0.02 |
| GCATTCGTCGTCGACCGACGA | Neg_control | Neg_control | 0.01 |
| GCCCCGATTCGTCGCGCGT | Neg_control | Neg_control | 0.00 |
| GCCGACTTGCGTAACGCGACT | Neg_control | Neg_control | 0.01 |
| GCCGCTGTCGATCGCGAATA | Neg_control | Neg_control | 0.17 |
| GCCGTTCGACGTCGTGCGATA | Neg_control | Neg_control | 0.01 |
| GCCTACCCGGCGCGATTAC | Neg_control | Neg_control | 0.01 |
| GCGTATGCCGAACGCGTCG | Neg_control | Neg_control | 0.00 |
| GCGTGCGTCGCGCTAATCG | Neg_control | Neg_control | 0.01 |
| GCTAAACTCGTCGAATCGTC | Neg_control | Neg_control | 0.01 |
| GCTAACATGTCGTAACGCGCG | Neg_control | Neg_control | 0.01 |
| GCTACCGTATATCGCACGACG | Neg_control | Neg_control | 0.01 |
| GCTAGATCGGCGGCGCGTATA | Neg_control | Neg_control | 0.00 |
| GCTATAGTACGTAGTTCGCGT | Neg_control | Neg_control | 0.01 |
| GCTCCGAATCGACCGTCGTCG | Neg_control | Neg_control | 0.00 |
| GCTTAATAACGCGACGACG | Neg_control | Neg_control | 0.01 |
| GCTTCAGTACGGCCGTACG | Neg_control | Neg_control | 0.01 |
| GCTTCGATTAACGCGCGTTA | Neg_control | Neg_control | 0.01 |
| GCTTCGTTCGGTCGTCGCGTA | Neg_control | Neg_control | 0.01 |
| GGACGCTTAATCGCGGCGTA | Neg_control | Neg_control | 0.00 |
| GGATGACCCGATTTCGCGCG | Neg_control | Neg_control | 0.01 |
| GGCGTACTCGATTCGCGAAC | Neg_control | Neg_control | 0.01 |
| GGTACAAGTTCGTCGCGTA | Neg_control | Neg_control | 0.01 |

| | | | |
|---|---|---|---|
| GGTCTTGATTCGCGCGAATA | Neg_control | Neg_control | 0.01 |
| GGTGATACTACGACGCGTTA | Neg_control | Neg_control | 0.01 |
| GTAAACATATCGCGCTACGA | Neg_control | Neg_control | 0.01 |
| GTAAACTATCGAGTACGTCGC | Neg_control | Neg_control | 0.00 |
| GTAAATTTCGCGACGTTACGT | Neg_control | Neg_control | 0.00 |
| GTACCGACGCCGCGAATATCG | Neg_control | Neg_control | 0.14 |
| GTACGACGAACCGGTTACGCG | Neg_control | Neg_control | 0.00 |
| GTACGGTCGACGCGTTCGG | Neg_control | Neg_control | 0.51 |
| GTACTGTGCGCGATACGCGAT | Neg_control | Neg_control | 0.02 |
| GTAGCTAAGCGTCGCGCGATT | Neg_control | Neg_control | 0.22 |
| GTAGTGCGCACGCGCGTCGAA | Neg_control | Neg_control | 3.50 |
| GTAGTTATAACGCGACGTCG | Neg_control | Neg_control | 0.00 |
| GTATAACGCGCATCGCGTAA | Neg_control | Neg_control | 0.01 |
| GTATAACGTACGCGCGTTACG | Neg_control | Neg_control | 0.01 |
| GTATCATTATCGCGGTCGA | Neg_control | Neg_control | 0.01 |
| GTATCGGCGGCGTAACGAC | Neg_control | Neg_control | 0.01 |
| GTATCTCCGGCGAACGATACG | Neg_control | Neg_control | 0.01 |
| GTATGAATTACCGCGCCGATA | Neg_control | Neg_control | 0.01 |
| GTATGACCTTTCGACGCGCGA | Neg_control | Neg_control | 0.02 |
| GTCAACTGGTAGCGACGCGCG | Neg_control | Neg_control | 0.01 |
| GTCCCAAGTCGCGCCGATCGA | Neg_control | Neg_control | 0.00 |
| GTCCCGAATCGATCGTCGTAC | Neg_control | Neg_control | 0.02 |
| GTCCGGCTACGTTACGACCGT | Neg_control | Neg_control | 0.00 |
| GTCGACCCTCGTTCGGTACGC | Neg_control | Neg_control | 0.01 |
| GTCGATCGATACGGCGTCGAC | Neg_control | Neg_control | 0.01 |
| GTCGATTCGCGACTCGCGCGT | Neg_control | Neg_control | 0.01 |
| GTCGCGAAGCCGCGTATCGTT | Neg_control | Neg_control | 0.02 |
| GTCGCGTCGTACGCGACGTCG | Neg_control | Neg_control | 0.01 |
| GTCGTACGGATCGAACGCGTA | Neg_control | Neg_control | 0.51 |
| GTCGTCGAACGTCGACGTACC | Neg_control | Neg_control | 0.01 |
| GTCTATACGCTATACGACG | Neg_control | Neg_control | 0.01 |
| GTCTCGCTAGTCGTCGATACG | Neg_control | Neg_control | 0.00 |
| GTGCCGACCGCCGTATCGAT | Neg_control | Neg_control | 0.01 |
| GTGCGTCAACGCGAATAGCG | Neg_control | Neg_control | 0.01 |
| GTGTCACCGATCGTCGCAA | Neg_control | Neg_control | 0.01 |
| GTTAAACAATTACGCGTCG | Neg_control | Neg_control | 0.11 |
| GTTACGAATCGTATCGCGCG | Neg_control | Neg_control | 0.18 |
| GTTACGCGTCGCGTATCGTCG | Neg_control | Neg_control | 0.00 |
| GTTCGCCGTCGAATTCGCGTA | Neg_control | Neg_control | 0.01 |
| GTTCGTATGCGTCGCCGAATA | Neg_control | Neg_control | 0.01 |
| GTTCTCGCGCGTAACGTCGTC | Neg_control | Neg_control | 0.01 |
| GTTGAGCGACCCGCGCGATA | Neg_control | Neg_control | 0.00 |
| GTTGCACAATCGACGCGGTAA | Neg_control | Neg_control | 0.01 |
| GTTTAAACGCGTCGCGTTT | Neg_control | Neg_control | 0.01 |
| GTTTGTCGAGCGCGTAACGGT | Neg_control | Neg_control | 0.01 |
