# Supplementary material for: Detection of gene cis-regulatory element perturbations in single-cell transcriptomes
Source: PLoS Comput Biol. 2021 Mar 12;17(3):e1008789. doi: 10.1371/journal.pcbi.1008789 (PMC8011753; doi:10.1371/journal.pcbi.1008789)
Supplement: S2 Table — (PDF) [file pcbi.1008789.s012.pdf]

			gRNA UMIs/cell (all)		gRNA UMIs/cell (assigned)	
	Number of cells	Number of reads	Median	Max	Median	Max
CROP-seq_Jurkat_TCR_stimulated	3259	768,559,887	1	38	1	38
CROP-seq_Jurkat_TCR_unstimulated	2646	592,105,684	1	65	1	65
PAC-seq (Purified)	9807	18,320,348	84	3926	35	2670
PAC-seq (Bulk)	8886	17,770,245	149	8259	62	4453
