# Supplementary material for: Detection of gene cis-regulatory element perturbations in single-cell transcriptomes
Source: PLoS Comput Biol. 2021 Mar 12;17(3):e1008789. doi: 10.1371/journal.pcbi.1008789 (PMC8011753; doi:10.1371/journal.pcbi.1008789)
Supplement: S3 Table — (PDF) [file pcbi.1008789.s013.pdf]

Gibson Assembly primers for cloning gRNAs	
gRNA Gibson fw	TAACTTGAAAGTATTTCGATTTCTTGGCTTTATATATCTTGTGGAAAGGACGAAACACCG
gRNA Gibson rv	GTTGATAACGGACTAGCCTTATTTAAACTTGCTATGCTGTTTCCAGCATAGCTCTTAAAC
Specific gRNA protospacers in Gibson Assembly amplification format	
sgGFP1	GGAAAGGACGAAACACC GCCCATCCTGGTCGAGCTGGA GTTTAAGAGCTATGCTGGAAAC
sgGFP2	GGAAAGGACGAAACACC GTCGCCCTCGAACTTCACCT GTTTAAGAGCTATGCTGGAAAC
sgGFP3	GGAAAGGACGAAACACC GTGAACCGCATCGAGCTGAA GTTTAAGAGCTATGCTGGAAAC
sgMsh2cis1	GGAAAGGACGAAACACC GGTGGGTGGGTAGGTGTGCT GTTTAAGAGCTATGCTGGAAAC
sgMsh2cis2	GGAAAGGACGAAACACC GCAAAATGTACACCAAGTCTG GTTTAAGAGCTATGCTGGAAAC
sgMsh2cis3	GGAAAGGACGAAACACC GAAACTCAGGGAAGGAGGCTG GTTTAAGAGCTATGCTGGAAAC
sgTdgf1cis1	GGAAAGGACGAAACACC GATTCAATACTTAGAAACATT GTTTAAGAGCTATGCTGGAAAC
sgTdgf1cis2	GGAAAGGACGAAACACC GTTACTGGCTTCGGTTTCTTG GTTTAAGAGCTATGCTGGAAAC
sgTdgf1cis3	GGAAAGGACGAAACACC GCCTTTCCATGATCACTTG GTTTAAGAGCTATGCTGGAAAC
sgZfp42cis1	GGAAAGGACGAAACACC GTGTTGAACTGCAACGCAAA GTTTAAGAGCTATGCTGGAAAC
sgZfp42cis2	GGAAAGGACGAAACACC GTGTCTCTCATTCCTCTGAA GTTTAAGAGCTATGCTGGAAAC
sgZfp42cis3	GGAAAGGACGAAACACC GGACGGATATGGCTTTGCGC GTTTAAGAGCTATGCTGGAAAC
sgMsh2ORF1	GGAAAGGACGAAACACC GAGGCGTCTAAGGAGAATGAG GTTTAAGAGCTATGCTGGAAAC
sgMsh2ORF2	GGAAAGGACGAAACACC GGTTAATACCCTGATACAGT GTTTAAGAGCTATGCTGGAAAC
Msh2 GFP/mCherry knock-in	GGAAAGGACGAAACACCGGACAGTGGGTGGGCGTGCAGTTTAAGAGCTATGCTGGAAAC
Tdgf1 GFP/mCherry knock-in	GGAAAGGACGAAACACC GAGATGGGGTACTTCTCATCC GTTTAAGAGCTATGCTGGAAAC
sgMsh2cis1 deletion 5'	GGAAAGGACGAAACACC GGTCTACCAATCGAGTTCCA GTTTAAGAGCTATGCTGGAAAC
sgMsh2cis1 deletion 3'	GGAAAGGACGAAACACC GCACCATGCAGCGCTGCGCCC GTTTAAGAGCTATGCTGGAAAC
sgMsh2cis2 deletion 5'	GGAAAGGACGAAACACC GCCAGCCTATTTGGCTATTTG GTTTAAGAGCTATGCTGGAAAC
sgMsh2cis2 deletion 3'	GGAAAGGACGAAACACC GCACTTCTGCTAGTGATACCC GTTTAAGAGCTATGCTGGAAAC
sgMsh2cis3 deletion 5'	GGAAAGGACGAAACACC GCTCCAAGGGTTTCAGACTC GTTTAAGAGCTATGCTGGAAAC
sgMsh2cis3 deletion 3'	GGAAAGGACGAAACACC GTAGAGCACTGCCCTCCCTGC GTTTAAGAGCTATGCTGGAAAC
sgGFP deletion 5'	GGAAAGGACGAAACACC GGCATCGCCCTCGCCCTCGC GTTTAAGAGCTATGCTGGAAAC
sgGFP deletion 3'	GGAAAGGACGAAACACC GCTGAAGCACTGCACGCCGT GTTTAAGAGCTATGCTGGAAAC
Primers to PCR amplify genomic regions surrounding cis-gRNA mutated sites	
Msh2cis1 upstream fw	TGTATAACGCACACTCAAGGCTGGG
Msh2cis1 downstream rv	CCAATCATAAGCGAGCAAGGTCCCA
Msh2cis2 upstream fw	TCT GAA GGG CAA AAC TGG AAT CTC A
Msh2cis2 downstream rv	GAA GGA AAC GAG AGA GGG GTT GGG
Msh2cis3 upstream fw	CCC ACG CCG AAC AGT CAC TAT ACA C
Msh2cis3 downstream rv	TTG ATG TCA AGG GGT CTG TGG GC
Tdgf1cis1 upstream fw	GGC CCC ACC CTT ACA CAC ACA ATT T
Tdgf1cis1 downstream rv	CTC GTC CCA TCA GTC ATG TCG GTG
Tdgf1cis2 upstream fw	TCA CTC AGG GAT GTG TTT CTC AGC G
Tdgf1cis2 downstream rv	GGA GGG ATT TGA GTC TGG GTT TGG G
Tdgf1cis3 upstream fw	CAG GGG TGG GAT TCT GGG AAA ACA T
Tdgf1cis3 downstream rv	GCT GGG CCT CCT TCA TTA ACA AGC T
Zfp42cis1 upstream fw	AAA AGT TGG TCG GTA GTA TGG CGG C
Zfp42cis1 downstream rv	TCA CTC CAT TTT CCA ATC AAG CAT CCA
Zfp42cis2 upstream fw	TGG AAA CCT GGA GTA AAG CAG TTC C
Zfp42cis2 downstream rv	GTT CAC CAC TGA CTA CAA ACG GTC

Zfp42cis3 upstream fw	CTG CTC AAC ACC ACA CCC CTG A
Zfp42cis3 downstream rv	TGG TAG CTC ACA AGA CCA GGT AG
Msh2cis1 deep sequencing tailed primer fw	CTTTCCCTACACGACGCTCTTCCGATCTCGTTTCCGGTCACTTGATAAAACAAA
Msh2cis1 deep sequencing tailed primer rv	GGAGTTCAGACGTGTGCTCTTCCGATCTTCCCGCGAACTCTAGGACTCT
Msh2cis2 deep sequencing tailed primer fw	CTTTCCCTACACGACGCTCTTCCGATCTGGGGCCAGGCAAAGGAGGC
Msh2cis2 deep sequencing tailed primer rv	GGAGTTCAGACGTGTGCTCTTCCGATCTGAAGGAAACGAGAGAGGGGTTGG
Msh2cis3 deep sequencing tailed primer fw	CTTTCCCTACACGACGCTCTTCCGATCTCGGTGTCCCCTACCCTCTCTAA
Msh2cis3 deep sequencing tailed primer rv	GGAGTTCAGACGTGTGCTCTTCCGATCTAGGCTGTGGTAGTTAATGGAGAATGC
Sequencing adapter-tailed reverse transcription primer	
Adapter-oligo(dT)	CGATGACG TAATACGACTCACTATAGGG ATACCACCATGG CTCTTTCCCTACACGACGCTCTTCCGATCT TTTTTTTTTTTTTTTTTTTTV
RT-qPCR primers	
Gapdh fw	TTGATGGCAACAATCTCCAC
Gapdh rv	CGTCCCGTAGACAAAATGGT
Actb fw	ATGGAGGGGAATACAGCCC
Actb rv	TTCTTTGCAGCTCCTTCGTT
Msh2-1 fw	AAGCTAGGCTTGTGTGAGTTC
Msh2-1 rv	CTCCTGGTAAAACGCATTCCTT
Msh2-2 fw	TGGCAGTTTTTGTGACTCCT
Msh2-2 rv	AAGGAACTCGTGGTTTTCCA
Tdgf1-1 fw	CCTCCAACGTTTTTACGAGC
Tdgf1-1 rv	GGTCCAAATTCAAACGCACT
Tdgf1-2 fw	TTTTACGAGCCGTCGAAGAT
Tdgf1-2 rv	TCTGATGGCAAGGTCTCTCC
gRNA hairpin fw	ACAGCATAGCAAGTTTAAATAAGGCT
gRNA hairpin rv	CGACTCGGTGCCACTTTT
GFP/mCherry double knock-in HDR primers	
Msh2 GFP HDR primer fw	CGTAAACGAGATCATTTCACGGATAAAGGCTCCGGCTCCGGTGAGCAAGGGCGAGGAGCT
Msh2 mCherry HDR primer fw	CGTAAACGAGATCATTTCACGGATAAAGGCTCCGGCTCCG GTGAGCAAGGGCGAGGAGGA
Msh2 GFP/mCherry HDR primer rv	GAGCACTACCCAGAACAGCACACAGTAAAGGTGAGCTCTA CTTGTACAGCTCGTCCATGC
Msh2 HDR Extension primer fw	AAGCCGAGGTGGTCGCAAAGAACAACAGCTTCGTAAACGAGATCATTTCACGGATAAAGG
Msh2 HDR Extension primer rv	CTAAACACTTCCGGATGCTGCTAGGCCAGGTTCCAGTGGGGAGCACTACCCAGAACAGCA
Msh2 knock-in validation fw	GCGAAGCTGAAGCAACTGA
Msh2 knock-in validation rv	CGTCCCCATTTAAACAGCAC
Tdgf1 GFP HDR primer fw	TTGTCTTTTCCTCCAACGTTTTTACGAGCCGTCGAAGATG GTGAGCAAGGGCGAGGAGCT
Tdgf1 mCherry HDR primer fw	TTGTCTTTTCCTCCAACGTTTTTACGAGCCGTCGAAGATG GTGAGCAAGGGCGAGGAGGA
Tdgf1 GFP/mCherry HDR primer rv	TCCGAAGTGGCTATCTCCAGCAACCAAAAAGTCAAGGTTA CTTGTACAGCTCGTCCATGC
Tdgf1 HDR Extension primer fw	TGGCTTTATGAACTAAAGCCATCTGCTAATATTGTGTTTCTTGTCTTTTCCTCCAACGTT
Tdgf1 HDR Extension primer rv	GCAAGACAAAAATCAGAGCGTCATAGAACGTGATTTTCCGAAGTGGCTATCTCCAGCAAC
Tdgf1 knock-in validation fw	TTGGGTGTTTCGAGAATGGC
Tdgf1 knock-in validation rv	GTTACTGACAAGGTTCAAGCAA
10X Chromium custom library prep primers	
For Msh2/Tdgf1/Zfp42 gene-specific library prep	
Msh2 gene-specific primer with read 2 adapter half-tail	GTGACTGGAGTTCAGACGTGTGCTCTTCCGATCT NNN tgtgctgttctgggtagtgctc
Tdgf1 gene-specific primer with read 2 adapter half-tail	GTGACTGGAGTTCAGACGTGTGCTCTTCCGATCT NNN aCACCCGAGTCTCTTTTCTGCAA
Zfp42 gene-specific primer with read 2 adapter half-tail	GTGACTGGAGTTCAGACGTGTGCTCTTCCGATCT agttcctaattgcctagcttttacct
Read 1 adapter half-tail primer to pair with above mix	GAGATCTACACTCTTTCC CTACACGACGCTCTTCCGATCT
Read 2 adapter full-tail with index N701	CAAGCAGAAGACGGCATACGAGAT TCGCCTTA GTGACTGGAGTTCAGACGTGTGCT
Read 2 adapter full-tail with index N702	CAAGCAGAAGACGGCATACGAGAT CTAGTACG GTGACTGGAGTTCAGACGTGTGCT
Read 2 adapter full-tail with index N703	CAAGCAGAAGACGGCATACGAGAT TTCTGCCT GTGACTGGAGTTCAGACGTGTGCT
Read 2 adapter full-tail with index N704	CAAGCAGAAGACGGCATACGAGAT GCTCAGGA GTGACTGGAGTTCAGACGTGTGCT

Read 1 adapter full-tail	AATGATACGGCGACCACC GAGATCTACACTCTTTCCCTACACGACGCTCTTCC
For gRNA-specific library prep	
gRNA sequence capture probe	/5Biosg/GTTTAAGAGCTATGCTGGAAACAGCATAGCAAGTTTAAATAAGGCTAGTCC GTTATCAACTTGAAAAAGTGGCACCGAGTCGGTGCAAAA
Sequence capture blocking oligo 1	CTACACGACGCTCTTCCGATCT/3SpC3/
Sequence capture blocking oligo 2	AAGCAGTGGTATCAACGCAGAGTACATGGG/3SpC3/
cDNA-specific primer with read 2 adapter half-tail, version 1 for pool	GTGACTGGAGTTCAGACGTGTGCTCTTCCGATC T GGTATCAACGCAGAGTACATGGG
cDNA-specific primer with read 2 adapter half-tail, version 2 for pool	GTGACTGGAGTTCAGACGTGTGCTCTTCCGATCT N GGTATCAACGCAGAGTACATGGG
cDNA-specific primer with read 2 adapter half-tail, version 3 for pool	GTGACTGGAGTTCAGACGTGTGCTCTTCCGATCT NN GGTATCAACGCAGAGTACATGGG
cDNA-specific primer with read 2 adapter half-tail, version 4 for pool	GTGACTGGAGTTCAGACGTGTGCTCTTCCGATCT NNN GGTATCAACGCAGAGTACATGGG
cDNA-specific primer with read 2 adapter half-tail, version 5 for pool	GTGACTGGAGTTCAGACGTGTGCTCTTCCGATCT NNNN GGTATCAACGCAGAGTACATGG
Read 1 adapter half-tail primer to pair with above mix	GAGATCTACACTCTTTCC CTACACGACGCTCTTCCGATCT
Read 2 adapter full-tail with index N701	CAAGCAGAAGACGGCATACGAGAT TCGCCTTA GTGACTGGAGTTCAGACGTGTGCT
Read 2 adapter full-tail with index N702	CAAGCAGAAGACGGCATACGAGAT CTAGTACG GTGACTGGAGTTCAGACGTGTGCT
Read 2 adapter full-tail with index N703	CAAGCAGAAGACGGCATACGAGAT TTCTGCCT GTGACTGGAGTTCAGACGTGTGCT
Read 2 adapter full-tail with index N704	CAAGCAGAAGACGGCATACGAGAT GCTCAGGA GTGACTGGAGTTCAGACGTGTGCT
Read 1 adapter full-tail	AATGATACGGCGACCACCGAGATCTACA CTCTTTCCCTACACGACGCTCTTCC
