# Supplementary material for: Detection of gene cis-regulatory element perturbations in single-cell transcriptomes
Source: PLoS Comput Biol. 2021 Mar 12;17(3):e1008789. doi: 10.1371/journal.pcbi.1008789 (PMC8011753; doi:10.1371/journal.pcbi.1008789)
Supplement: S1 File — (DOCX) [file pcbi.1008789.s014.docx]

Follow the 10XChromium protocol up until the Ampure purification of cDNA. During this step, perform the 0.6X Ampure purification to purify transcriptome cDNA, but keep the supernatant from this purification, add 1.0X Ampure beads to this supernatant (which yields a 1.6X Ampure reaction), and purify the bead-bound fraction to purify short cDNA which contains polyA-gRNAs. Re-amplify the short cDNA using 020417_10XTSO_r2halftail_v1-5 and 120216_10Xr1seq_halftail_rv primers until you have >1000 ng such that you can use half for sequence capture.

Follow this protocol with 500 ng of the short cDNA sample.

**Sequence Capture**

1. 500ng cDNA lib. + 5ug Cot-1 DNA + 1nmol each blocking oligo (120216_10xr1_seqcapblock + 120216_10xr2_seqcapblock); Speedvac on medium for 15’-1hr. Store overnight at RT if needed.
2. Thaw reagents at RT. Add –

8.5ul xGen 2x Hybridization buffer

2.7ul xGen Hybridization buffer enhancer

1.8ul Nuclease free water (per rxn)

Incubate for 5’ and pipette to mix and transfer 13ul to PCR tube -keep a PCR block ready

1. Incubate in a thermal cycler @ 95C for 10min. Set another block to the 65C (**75C lid**).
2. Remove from thermal cycler and immediately add 4ul of 0.75pmol/ul suspension (3pmol each) capture probe (092016_gRNAFEreg_seqcaptprobe). Vortex and briefly spin down.
3. Incubate @65C for 24hrs in a thermal cycler (**75C lid**).
4. Start preparing beads and wash buffers at least 30-45min before incubation is supposed to end.
   1. Prepare 1X Bead wash buffers first
   2. Transfer 75ul MYOne Streptavidin C1 beads to a 1.7ml tube
   3. Pellet beads using magnet and discard supernatant
   4. Add 200ul 1X bead wash buffer and vortex for 10sec – magnet – discard sup.
   5. Repeat step d.
   6. Add 100ul 1X bead wash buffer and re-suspend beads. Once hybridization is over - Transfer beads to a PCR tube – magnet – discard sup.
5. Transfer hybridization mix to the PCR tube with beads – mix thoroughly with pipette (10 times)
6. Bind DNA to beads by placing PCR tube in a thermal cycler at 65C for 45min. Vortex for 3sec every 12min during this incubation. Prepare all other wash buffers and set heat-block to 65C. Preheat necessary wash buffers.
7. 65C wash –
   1. Add 100ul preheated 1X Wash buffer I to tube. Vortex for 10sec. Transfer mixture to a 1.7ml tube. Vortex briefly – magnet – discard sup
   2. Add 200ul preheated 1X Stringent wash buffer. Slowly pipette 10 times. Avoid bubbles.
   3. Incubate at 65C in heat-block (with water preferably) for 5min – magnet – discard sup
   4. Repeat b – c.
8. RT washes –
   1. Add 200ul RT 1X wash buffer I and vortex for 2min – magnet – discard sup
   2. Add 200ul 1X wash buffer II and vortex for 1min – magnet – discard sup
   3. Add 200ul 1X wash buffer III and vortex for 30sec – magnet – discard sup
9. Remove tube from magnetic rack and add 20ul nuclease free water and resuspend beads (mix 10 times).
10. Post-capture PCR 1 – 100ul PCR rxn using 2X NEBNext Mastermix, 8 cycles
    1. 2X NEBNext Mastermix – 50ul

Beads – 23 to 25ul

500 nM mix of 020417_10XTSO_r2halftail_v1-5

500 nM 120216_10Xr1seq_halftail_rv

Make up to 100ul with nuclease free water

(98C – 30s; 8 cycles of 98C – 10s, 62C – 30s, 72C – 30s; 72C – 5’)

1. Ampure purification –
   1. Vortex Ampure XP beads and take 1.5X PCR volume (150ul to 100ul PCR) in a 1.7ml tube
   2. Mix PCR+Dynabeads and Ampure XP beads – Incubate at RT for 5min
   3. Quick spin – magnet – discard sup
   4. Wash with 80% Ethanol – magnet – discard sup
   5. Repeat d. Remove ethanol and air dry or incubate at 37C heat block for 5min (until color of beads turn lighter – appear cracked).
   6. Add 40ul Qiagen Buffer EB and transfer to PCR tube. Use small magnet to pellet beads. Collect sup and transfer it to a 1.7ml tube. Save 20ul as backup.
2. qPCR with 0.1 uL input template to determine cycle count using the EvaGreen approach with 030617_10xr2seq_24bp_N701_fw and 030617_10Xr1seq_25bp_full_rv, NEBNext Ta=72.
3. Post-capture PCR 2 – 100ul PCR rxn using NEBNext mix, n cycles
   1. 2X NEBNext mix – 50ul

Eluted sample – 20 ul

20 uM 030617_10xr2seq_24bp_NXXX_fw - 2.5 uL

20 μM 030617_10Xr1seq_25bp_full_rv – 2.5ul

(98C – 30s; X cycles of 98C – 10s, 72C – 30s, 72C – 30s; 72C – 5’)

16. Expect ~307-311 bp band. Run 2 uL aliquot on gel. If clean band, proceed to Ampure. If not, gel purify using 2% SYBR gel.

1. Ampure purification –
   1. Vortex Ampure XP beads and take 1.2X PCR volume (120ul to 100ul PCR) in a 1.7ml tube
   2. Mix PCR+Dynabeads and Ampure XP beads – Incubate at RT for 5min
   3. Quick spin – magnet – discard sup
   4. Wash with 80% Ethanol – magnet – discard sup
   5. Repeat d. Remove ethanol and air dry or incubate at 37C heat block for 5min (until color of beads turn lighter – appear cracked).
   6. Add 40ul Qiagen Buffer EB and transfer to PCR tube. Use small magnet to pellet beads. Collect sup and transfer it to a 1.7ml tube.
2. TapeStation. Store at 4°C overnight or at –20°C for up to 1 week
   1. Reads should be split as follows:
      1. Read 1: 26 cycles
      2. i7 index: 8 cycles
      3. i5 index: 0 cycles
      4. Read 2: 57 cycles

**Blocking oligos**

120216_10xr1_seqcapblock CTACACGACGCTCTTCCGATCT/3SpC3/

120216_10Xr2_seqcapblock AAGCAGTGGTATCAACGCAGAGTACATGGG/3SpC3/

**Biotin probe oligos**

092016_gRNAFEreg_seqcaptprobe /5Biosg/**GTTTAAGAGCTATGCTGGAAACAGCATAGCAAGTTTAAATAAGGCTAGTCCGTTATCAACTTGAAAAAGTGGCACCGAGTCGGTGCAAAA**

020417_10XTSO_r2halftail_v1 GTGACTGGAGTTCAGACGTGTGCTCTTCCGATC T GGTATCAACGCAGAGTACATGGG

020417_10XTSO_r2halftail_v2 GTGACTGGAGTTCAGACGTGTGCTCTTCCGATCT N GGTATCAACGCAGAGTACATGGG

020417_10XTSO_r2halftail_v3 GTGACTGGAGTTCAGACGTGTGCTCTTCCGATCT NN GGTATCAACGCAGAGTACATGGG

020417_10XTSO_r2halftail_v4 GTGACTGGAGTTCAGACGTGTGCTCTTCCGATCT NNN GGTATCAACGCAGAGTACATGGG

020417_10XTSO_r2halftail_v5 GTGACTGGAGTTCAGACGTGTGCTCTTCCGATCT NNNN GGTATCAACGCAGAGTACATGG

Pair with:

120216_10Xr1seq_halftail_rv GAGATCTACACTCTTTCC CTACACGACGCTCTTCCGATCT

Use NEBNext Ta=62

2^nd^ PCR:

030617_10xr2seq_24bp_N701_fw CAAGCAGAAGACGGCATACGAGAT TCGCCTTA GTGACTGGAGTTCAGACGTGTGCT

031317_10xr2seq_24bp_N702_fw CAAGCAGAAGACGGCATACGAGAT CTAGTACG GTGACTGGAGTTCAGACGTGTGCT

031317_10xr2seq_24bp_N703_fw CAAGCAGAAGACGGCATACGAGAT TTCTGCCT GTGACTGGAGTTCAGACGTGTGCT

031317_10xr2seq_24bp_N704_fw CAAGCAGAAGACGGCATACGAGAT GCTCAGGA GTGACTGGAGTTCAGACGTGTGCT

With:

030617_10Xr1seq_25bp_full_rv AATGATACGGCGACCACCGAGATCTACA CTCTTTCCCTACACGACGCTCTTCC

Full library product

Knockout gRNA hairpin:

CAAGCAGAAGACGGCATACGAGATNNNNNNNNGTGACTGGAGTTCAGACGTGTGCTCTTCCGATCTNNNNGGTATCAACGCAGAGTACATGGGGNNNNNNNNNNNNNNNNNNNGTTTAAGAGCTATGCTGGAAACAGCATAGCAAGTTTAAATAAGGCTAGTCCGTTATCAACTTGAAAAAGTGGCACCGAGTCGGTGCAAAAAAAAAAAAAAAAAAAAAAAAAAAAAANNNNNNNNNNNNNNNNNNNNNNNNAGATCGGAAGAGCGTCGTGTAGGGAAAGAGTGTAGATCTCGGTGGTCGCCGTATCATT
