# Supplementary material for: Detection of gene cis-regulatory element perturbations in single-cell transcriptomes
Source: PLoS Comput Biol. 2021 Mar 12;17(3):e1008789. doi: 10.1371/journal.pcbi.1008789 (PMC8011753; doi:10.1371/journal.pcbi.1008789)
Supplement: S2 File — (ZIP) [file pcbi.1008789.s015.zip › S2_File/S2_File.docx]

**S2 File. Comparison of differential expression results across different differential expression methods**

To assess the sensitivity of our results with respect to the choice of differential expression method, we also conducted differential expression analysis with MAST and DESeq2. Overall, we found that while there were slight differences in the sets of differentially expressed genes identified by each of the three methods, our main results that (1) Target genes are differentially expressed in sorted populations, (2) Trh was consistently differentially expressed in sorted populations of cells receiving Msh2-targeting gRNAs, (3) The analysis was underpowered to discover differentially expressed genes in unsorted populations when rate of mono/biallelic loss was low, remained unchanged.

We had chosen to use the Wilcoxon Rank Sum test in the main manuscript as it is the default test for the popular scRNA-seq analysis package Seurat [1], and because it had been shown by Soneson & Robinson (2018) [2] to have a relatively low false discovery rate when benchmarked against other methods, despite being a relatively simple test. We reasoned that using the Wilcoxon test hence provides a more conservative lower bound with regards to the expected discovery rate in the power analysis.

However, other methods may have higher sensitivity. Hence, we also conducted differential expression analysis with two other methods: (1) MAST [3], which is a single-cell differential expression method that uses a two-part generalized linear model consisting of one component that models the discrete expression rate of each gene and a second component that models the continuous expression level conditioned on the gene being expressed, as well as (2) DESeq2 [4], which was originally designed for the bulk setting and is based on the negative binomial distribution.

When used to identify differentially expressed genes in the GFPChe (sorted), we found that all methods found Msh2 to be significantly differentially expressed vs. the ZfpSC1 control (adjusted p-value < 0.05) when using transcript-targeted sequencing. This was also the case for Tdgf1 vs. the ZfpSC2 control (adjusted p-value < 0.05).

For cells receiving Msh2-targeting gRNAs, 75.0%, 75.9% and 61.5% of all differentially expressed genes identified by at least 1 method were identified by at least 2 methods (adjusted p-value < 0.05, for n = 120, 82, 26 genes in cells receiving MshSC1, MshSC2 and MshSC3 respectively, S9A Fig). Overlap of the number of differentially expressed genes detected across Msh2-targeting gRNAs for each test was more challenging to assess because of the different number of cells assigned to have received each gRNA (MshSC1 = 1657, MshSC2 = 631, MshSC3 = 187). However, for all methods, the larger the number of cells, the greater the number of differentially expressed genes detected (S9B Fig). Trh was found to be significantly differentially expressed by both the Wilcoxon test and MAST across all Msh-targeting gRNAs (S9C Fig).

For cells receiving Tdgf1-targeting gRNAs, all tests identified Tdgf1 as the only significantly differentially expressed gene, with the exception of the Wilcoxon test in cells receiving TdgfSC1, where Lrrc2 was significant at adjusted p = 0.0479. Lrrc2 is the neighboring gene to Tdgf1, and Rajagopal et al. (2018) [5] had previously found that neighboring genes often share regulatory input. Hence, Lrrc2 could be a true positive. Nevertheless, the effect is weak and inconsistent across gRNAs.

We also repeated the analysis in the unsorted population. Focusing on the expected positive controls, we found that the analysis was often underpowered to detect genes that should be differentially expressed, even with transcript-targeted sequencing (Figure 4). Differential expression results were the same for MAST and DESeq2, with the exception of Trh where DESeq2 found Trh to also be differentially expressed in cells receiving MshSC1 (S9D Fig). In particular, only Tdgf1 was differentially expressed across all gRNAs in all three methods.

When we conducted power simulations using MAST (without empirical bayes regularization of the gene variance, so that genes are treated independently), we observe that MAST is more sensitive than the Wilcoxon test, as we might expect (S9 Fig)

1. Butler A, Hoffman P, Smibert P, Papalexi E, Satija R. Integrating single-cell transcriptomic data across different conditions, technologies, and species. Nat Biotechnol. 2018;36: 411–420.

2. Soneson C, Robinson MD. Bias, robustness and scalability in single-cell differential expression analysis. Nat Methods. 2018;15: 255–261.

3. Finak G, McDavid A, Yajima M, Deng J, Gersuk V, Shalek AK, et al. MAST: a flexible statistical framework for assessing transcriptional changes and characterizing heterogeneity in single-cell RNA sequencing data. Genome Biol. 2015;16: 278.

4. Love MI, Huber W, Anders S. Moderated estimation of fold change and dispersion for RNA-seq data with DESeq2. Genome Biol. 2014;15: 550.

5. Rajagopal N, Srinivasan S, Kooshesh K, Guo Y, Edwards MD, Banerjee B, et al. High-throughput mapping of regulatory DNA. Nat Biotechnol. 2016;34: 167–174.
