# Supplementary material for: Detection of gene cis-regulatory element perturbations in single-cell transcriptomes
Source: PLoS Comput Biol. 2021 Mar 12;17(3):e1008789. doi: 10.1371/journal.pcbi.1008789 (PMC8011753; doi:10.1371/journal.pcbi.1008789)
Supplement: S3 File — (ZIP) [file pcbi.1008789.s016.zip › S3_File/powerpAC.html]

powerpAC


In [1]:

```
# core
library(Seurat)
library(tidyr)
library(dplyr)

# plotting
library(ggplot2)

# for installation
library(devtools)
```

```
Attaching package: ‘dplyr’


The following objects are masked from ‘package:stats’:

    filter, lag


The following objects are masked from ‘package:base’:

    intersect, setdiff, setequal, union


Loading required package: usethis

Registered S3 method overwritten by 'cli':
  method     from    
  print.boxx spatstat
```

# Installation¶

Install the package using devtools by running `devtools::install("/file/to/package")`. Then, load the package.

In [2]:

```
library(powerpAC)
```

Documentation for the various functions can be accessed via `?`

In [3]:

```
?runPowerAnalysis
```

|  |  |
| --- | --- |
| runPowerAnalysis {powerpAC} | R Documentation |

## Simulation-based power analysis

### Description

Runs a simulation-based power analysis that takes into account several factors
including rate of mono/biallelic loss, base gene expression, and the number of
cells per target gRNA

### Usage

```
runPowerAnalysis(
  sce.control,
  ko.frac,
  biallelic.ko.frac,
  save.path,
  num.buckets = 10,
  num.replicates = 100,
  ns.treatment.cells = c(1:9) * 100,
  runDE = runWilcox
)
```

### Arguments

|  |  |
| --- | --- |
| `sce.control` | Seurat object of the transcriptome of a control population |
| `ko.frac` | Fraction of mono + bi/allelic loss |
| `biallelic.ko.frac` | Fraction of biallelic loss |
| `save.path` | Save path for simulation results (optional). |
| `num.buckets` | Number of gene buckets. Default is 10. |
| `num.replicates` | Number of replicates Default is 100. |
| `ns.treatment.cells` | Range of treatment cells to test over. Default is `c(1:9)*100`. |
| `runDE` | Differential expression method that takes as input a Seurat object and returns a dataframe containing raw p-values and average log-fold change. Default is `runWilcox`. |

### Value

Dataframe containing power analysis results with raw p-values and average log fold-change per gene bucket and number of treatment cells

---

[Package *powerpAC* version 0.1.0.0 ]

# Inputs¶

The simulation framework requires as input (a) a control cell population, (b) the fraction of monoallelic + biallelic loss, (c) the fraction of biallelic loss

Optionally, users may also pass simulation parameters like the number of buckets, the number of replicates, the range of treatment cells over which to simulate, and the differential expression method. Also, if `save.path` is provided, an output file is saved to that path.

## Control¶

Load or create a Seurat object containing the transcriptomes of a control population. As an example, we will create one from our wildtype (unsorted) data, extracting only the control cells

In [4]:

```
load("../processed/wt_txm.Robj")
```

In [5]:

```
sce <- CreateSeuratObject(transcriptome@raw.data[,row.names(transcriptome@meta.data)])
sce
```

```
An object of class Seurat 
18207 features across 7923 samples within 1 assay 
Active assay: RNA (18207 features, 0 variable features)
```

Normalize data

In [6]:

```
sce <- NormalizeData(sce)
```

In [7]:

```
assignments <- read.csv("../data/wt.labels.csv", row.names = 1, header = F)
ident <- assignments$V2
names(ident) <- gsub("-1", "", row.names(assignments))
table(ident)
```

```
ident
Control  MshSC1  MshSC2  MshSC3 TdgfSC1 TdgfSC2 TdgfSC3  ZfpSC1  ZfpSC2  ZfpSC3 
   1150     580     727     724     841    1129    1018     880     819    1019
```

In [8]:

```
ident.hq <- ident[row.names(transcriptome@meta.data)]
names(ident.hq) <- row.names(transcriptome@meta.data)
table(ident.hq)
```

```
ident.hq
Control  MshSC1  MshSC2  MshSC3 TdgfSC1 TdgfSC2 TdgfSC3  ZfpSC1  ZfpSC2  ZfpSC3 
    952     485     654     635     701     937     865     741     689     854
```

In [9]:

```
Idents(sce) <- factor(ident.hq, levels = c('Control', 'MshSC1', 'MshSC2', 'MshSC3', 'TdgfSC1', 'TdgfSC2', 'TdgfSC3', 'ZfpSC1', 'ZfpSC2', 'ZfpSC3'))
levels(Idents(sce))
```

1. 'Control'
2. 'MshSC1'
3. 'MshSC2'
4. 'MshSC3'
5. 'TdgfSC1'
6. 'TdgfSC2'
7. 'TdgfSC3'
8. 'ZfpSC1'
9. 'ZfpSC2'
10. 'ZfpSC3'

In [10]:

```
sce.control <- sce[,(Idents(sce) == 'Control') & (complete.cases(Idents(sce)))]
sce.control
```

```
An object of class Seurat 
18207 features across 952 samples within 1 assay 
Active assay: RNA (18207 features, 0 variable features)
```

In [11]:

```
dim(sce.control)
```

1. 18207
2. 952

## Fraction of loss, and fraction of biallelic loss¶

In [19]:

```
ko.frac <- 0.288
biallelic.ko.frac <- 0.036
```

# Simulate¶

Run a small simulation with only 5 replicates for each gene bucket/number of treatment cell combination

In [20]:

```
res <- runPowerAnalysis(
    sce.control = sce.control,
    ko.frac = ko.frac, 
    biallelic.ko.frac = biallelic.ko.frac, 
    save.path = "sims/test.csv", 
    num.gene.buckets = 10, 
    num.replicates = 5,
    ns.treatment.cells = c(1:9)*100, 
    runDE = runWilcox 
)
```

In [21]:

```
head(res)
```

A data.frame: 6 × 4

|  | p\_val | avg\_logFC | gene\_bucket\_avg | num\_treatment\_cells |
| --- | --- | --- | --- | --- |
|  | <dbl> | <dbl> | <dbl> | <dbl> |
| Rep1 | 0.1805067 | -0.056065969 | 0.2245916 | 100 |
| Rep3 | 0.4018188 | -0.030855662 | 0.2245916 | 100 |
| Rep2 | 0.4269342 | -0.005099857 | 0.2245916 | 100 |
| Rep5 | 0.6451888 | -0.028403110 | 0.2245916 | 100 |
| Rep4 | 0.6481927 | -0.001324286 | 0.2245916 | 100 |
| Rep51 | 0.2883810 | -0.031711604 | 0.2245916 | 200 |

# Visualize¶

Load the results of the power analysis from file, and summarize

In [12]:

```
path <- paste0("sims/MshSC2_wilcox.csv")
res <- read.csv(path, row.names = 1)
head(res)
```

A data.frame: 6 × 4

|  | p\_val | avg\_logFC | gene\_bucket\_avg | num\_treatment\_cells |
| --- | --- | --- | --- | --- |
|  | <dbl> | <dbl> | <dbl> | <int> |
| Rep55 | 0.01108061 | 0.05682877 | 0.2245916 | 100 |
| Rep24 | 0.02613791 | -0.10818114 | 0.2245916 | 100 |
| Rep27 | 0.04716698 | -0.06695882 | 0.2245916 | 100 |
| Rep39 | 0.08738013 | 0.05033013 | 0.2245916 | 100 |
| Rep63 | 0.11270395 | -0.03430788 | 0.2245916 | 100 |
| Rep17 | 0.12017205 | 0.01657176 | 0.2245916 | 100 |

In [13]:

```
options(repr.plot.width = 8, repr.plot.height = 6)
plotPowerAnalysis(res)
```

The log normalized expression of Msh2\* (with transcript-targeted sequencing) is 1.82

In [16]:

```
options(repr.plot.width = 8, repr.plot.height = 6)
plotCorrectedPowerAnalysis(res, sce.control, target.expr = 1.82)
```

In [17]:

```
options(repr.plot.width = 8, repr.plot.height = 6)
plotIFCorrectedPowerAnalysis(res, sce.control, target.expr = 1.82)
```

# Session information¶

In [18]:

```
sessionInfo()
```

```
R version 4.0.2 (2020-06-22)
Platform: x86_64-pc-linux-gnu (64-bit)
Running under: Ubuntu 20.04.1 LTS

Matrix products: default
BLAS:   /usr/lib/x86_64-linux-gnu/blas/libblas.so.3.9.0
LAPACK: /usr/lib/x86_64-linux-gnu/lapack/liblapack.so.3.9.0

locale:
 [1] LC_CTYPE=C.UTF-8       LC_NUMERIC=C           LC_TIME=C.UTF-8       
 [4] LC_COLLATE=C.UTF-8     LC_MONETARY=C.UTF-8    LC_MESSAGES=C.UTF-8   
 [7] LC_PAPER=C.UTF-8       LC_NAME=C              LC_ADDRESS=C          
[10] LC_TELEPHONE=C         LC_MEASUREMENT=C.UTF-8 LC_IDENTIFICATION=C   

attached base packages:
[1] stats     graphics  grDevices utils     datasets  methods   base     

other attached packages:
[1] powerpAC_0.1.0.0 devtools_2.3.2   usethis_1.6.3    ggplot2_3.3.2   
[5] dplyr_1.0.2      tidyr_1.1.2      Seurat_3.2.2    

loaded via a namespace (and not attached):
  [1] Rtsne_0.15            colorspace_1.4-1      deldir_0.1-29        
  [4] ellipsis_0.3.1        ggridges_0.5.2        rprojroot_1.3-2      
  [7] IRdisplay_0.7.0       base64enc_0.1-3       fs_1.5.0             
 [10] spatstat.data_1.4-3   leiden_0.3.4          listenv_0.8.0        
 [13] remotes_2.2.0         ggrepel_0.8.2         fansi_0.4.1          
 [16] codetools_0.2-16      splines_4.0.2         pkgload_1.1.0        
 [19] polyclip_1.10-0       IRkernel_1.1.1        jsonlite_1.7.1       
 [22] ica_1.0-2             cluster_2.1.0         png_0.1-7            
 [25] uwot_0.1.8            shiny_1.5.0           sctransform_0.3.1    
 [28] compiler_4.0.2        httr_1.4.2            backports_1.1.10     
 [31] assertthat_0.2.1      Matrix_1.2-18         fastmap_1.0.1        
 [34] lazyeval_0.2.2        cli_2.1.0             later_1.1.0.1        
 [37] htmltools_0.5.0       prettyunits_1.1.1     tools_4.0.2          
 [40] rsvd_1.0.3            igraph_1.2.6          gtable_0.3.0         
 [43] glue_1.4.2            RANN_2.6.1            reshape2_1.4.4       
 [46] Rcpp_1.0.5            spatstat_1.64-1       vctrs_0.3.4          
 [49] nlme_3.1-149          lmtest_0.9-38         stringr_1.4.0        
 [52] globals_0.13.1        ps_1.4.0              testthat_2.3.2       
 [55] mime_0.9              miniUI_0.1.1.1        lifecycle_0.2.0      
 [58] irlba_2.3.3           goftest_1.2-2         future_1.19.1        
 [61] MASS_7.3-52           zoo_1.8-8             scales_1.1.1         
 [64] promises_1.1.1        spatstat.utils_1.17-0 parallel_4.0.2       
 [67] RColorBrewer_1.1-2    memoise_1.1.0         reticulate_1.18      
 [70] pbapply_1.4-3         gridExtra_2.3         rpart_4.1-15         
 [73] stringi_1.5.3         desc_1.2.0            pkgbuild_1.1.0       
 [76] repr_1.1.0            rlang_0.4.8           pkgconfig_2.0.3      
 [79] matrixStats_0.57.0    evaluate_0.14         lattice_0.20-41      
 [82] ROCR_1.0-11           purrr_0.3.4           tensor_1.5           
 [85] patchwork_1.0.1       htmlwidgets_1.5.2     cowplot_1.1.0        
 [88] tidyselect_1.1.0      processx_3.4.4        RcppAnnoy_0.0.16     
 [91] plyr_1.8.6            magrittr_1.5          R6_2.4.1             
 [94] generics_0.1.0        pbdZMQ_0.3-3.1        pillar_1.4.6         
 [97] withr_2.3.0           mgcv_1.8-33           fitdistrplus_1.1-1   
[100] survival_3.1-12       abind_1.4-5           tibble_3.0.4         
[103] future.apply_1.6.0    crayon_1.3.4          uuid_0.1-4           
[106] KernSmooth_2.23-17    plotly_4.9.2.1        grid_4.0.2           
[109] data.table_1.13.2     callr_3.5.1           digest_0.6.27        
[112] xtable_1.8-4          httpuv_1.5.4          munsell_0.5.0        
[115] viridisLite_0.3.0     sessioninfo_1.1.1
```
