# Supplementary material for: Detection of gene cis-regulatory element perturbations in single-cell transcriptomes
Source: PLoS Comput Biol. 2021 Mar 12;17(3):e1008789. doi: 10.1371/journal.pcbi.1008789 (PMC8011753; doi:10.1371/journal.pcbi.1008789)
Supplement: Attachment — Submitted filename: 201123_response-to-reviewers.docx [file pcbi.1008789.s017.docx]

All reviewers agree that the experimental method presented is important and advances the field. As you prepare your revision, I would encourage you to focus in particular on reviewer comments related to computational analysis and modeling, given the audience of PLOS Computational Biology.

We thank the reviewers for their comments and suggestions. Please find attached our responses in blue inline.

Reviewer #1: In this manuscript, Yeo et al present a novel design of sgRNAs that enables direct detection of sgRNAs during single-cell RNA-seq. The authors accomplish this by appending a polyA tail downstream of the sgRNA sequence. They then identify non-coding regions to test using their CRISPR screening approach, and test pAC-Seq on these regions.

Overall, the general concept of this paper is clear and has potential to improve recently developed single-cell perturbation assays. I reviewed a previous version of this manuscript at a different journal over 2 years ago and my previous concerns have been addressed in this new version.

We would like to thank reviewer #1 for their kind comments. We are glad that we were able to address their concerns in our new manuscript.

Reviewer #2: The manuscript by Yeo and collaborators presents a new protocol to directly measure gRNAs in scRNA-seq. Poly-adenine CRISPR gRNA-based scRNA-seq (pAC-Seq) is proposed as a method to edit regulatory elements and measuring the effect effect on gene expression through scRNA-seq. The method is very relevant for the field as it combines the ability to directly measuring gRNA in each individual cell without the limitations of barcode swapping due to lentiviral recombination. Despite the excitement for the potential technological advancement associated with this new method, several limitations were identified that overall diminish this reviewer’s enthusiasm, as detailed below.

We would like to thank reviewer #2 for their constructive comments.

Major comments:

1. It appears that the results presented are from individual experiments of pAC-Seq, without experimental replicates. While in the very first studies of scRNA-seq it was acceptable to consider individual cells within one experiment as technical replicates, this is not the case anymore. Technical replicates are especially important in this case to demonstrate that the results of the editing experiment are consistent across technical iterations of the protocol.

We have included Sanger sequencing indicating consistent, robust activity of each of the gRNAs targeting Msh2, Tdgf1 and Zfp42 in Fig S1. We note that we used three different gRNAs for each target. In addition, for the one gene that we found to be consistently and significantly differentially expressed in Msh2-targeted cells in our pAC-Seq data (Trh), we subsequently validated this causal dependency by knocking out the Msh2-ORF in Trh-GFP cells (Fig 3F). Thus, while in general we agree with the reviewer that technical replicates are important for verifying reproducibility, in this case we have validated the results with independent downstream experiments.

2. Minimal consideration is given to off target effects, by specifically analyzing only the genes of interest. Even in the only analysis where widespread effects on gene expression are considered, the use of the MWU test is not appropriate. A negative binomial model that directly models count data (DESEQ2) or another method specifically suited for scRNA-seq would be more appropriate for the analysis of differential gene expression in these experiments.

We have now extended our analysis to two other scRNA-seq differential expression methods, DESeq2 and MAST. We found that while there were slight differences in the sets of differentially expressed genes identified by each of the three methods, our main results that (1) Target genes are differentially expressed in sorted populations, (2) Trh was consistently differentially expressed in sorted populations of cells receiving Msh2-targeting gRNAs, (3) The analysis was underpowered to discover differentially expressed genes in unsorted populations when rate of mono/biallelic loss was low, remain unchanged.

To clarify how we carried out the differential expression analysis and the presentation of the results: We conducted the analysis with multiple testing correction on the full set of genes but focused our results on the three targeted genes because they served as positive controls. We conducted differential expression on first the purified population, to determine any other downstream effects, and then on the unsorted population.

We chose to use the MWU (Wilcoxon Rank Sum) test because (a) it is the default test for the popular scRNA-seq analysis package Seurat, and (b) it was shown by Soneson & Robinson (2018) to have a relatively low false discovery rate when benchmarked against other methods, despite being a relatively simple test. We reasoned that using the Wilcoxon test hence provides a more conservative lower bound with regards to the expected discovery rate in the power analysis.

However, it is true that other methods may provide higher sensitivity. Hence, we have now also conducted differential expression analysis with two other methods: (1) MAST (Finak 2015), which is a single-cell differential expression method that uses a two-part generalized linear model consisting of one component that models the discrete expression rate of each gene and a second component that models the continuous expression level conditioned on the gene being expressed, as well as (2) DESeq2 (Love 2014), which was originally designed for the bulk setting and is based on the negative binomial distribution.

When used to identify differentially expressed genes in the GFPChe (sorted), we found that all methods found Msh2 to be significantly differentially expressed vs. the ZfpSC1 control (adjusted p-value < 0.05) when using transcript-targeted sequencing. This was also the case for Tdgf1 vs. the ZfpSC2 control (adjusted p-value < 0.05). (Figure 3D, reproduced below)

In addition, for cells receiving Msh2-targeting gRNAs, 75.0%, 75.9% and 61.5% of all differentially expressed genes identified by at least 1 method were identified by at least 2 methods (adjusted p-value < 0.05, for n = 120, 82, 26 genes in cells receiving MshSC1, MshSC2 and MshSC3 respectively, Figure S10A, reproduced below). Overlap of the number of differentially expressed genes detected across Msh2-targeting gRNAs for each test was more challenging to assess because of the different number of cells assigned to have received each gRNA (MshSC1 = 1657, MshSC2 = 631, MshSC3 = 187). However, for all methods, the larger the number of cells, the greater the number of differentially expressed genes detected (Figure S10B). Trh was found to be significantly differentially expressed by both the Wilcoxon test and MAST (Figure 3E, reproduced above). As previously noted, this result was independently validated using Msh2-ORF knockout in Trh-GFP cells.

For cells receiving Tdgf1-targeting gRNAs, all tests identified Tdgf1 as the only significantly differentially expressed gene, with the exception of the Wilcoxon test in cells receiving TdgfSC1, where Lrrc2 was significant at adjusted p = 0.0479. Lrrc2 is the neighboring gene to Tdgf1, and many studies including our previous tiled CRISPR screening work in Rajagopal et al. (2018) have found that neighboring genes often share regulatory input. Hence, Lrrc2 could be a true positive. However, since the effect is weak and inconsistent across gRNAs, we do not pursue this analysis.

We also repeated the analysis in the unsorted population. Focusing on the expected positive controls, we found that the analysis was often underpowered to detect genes that should be differentially expressed, even with transcript-targeted sequencing. We have reproduced here the results from Figure 4, where * indicates significant differential expression at adjusted p-value < 0.05 by the Wilcoxon test. These results were the same for MAST and DESeq2, with the exception of Trh where DESeq2 found Trh to also be differentially expressed in cells receiving MshSC1. In particular, only Tdgf1 was differentially expressed across all gRNAs in all three methods. We hypothesized that lack of power was due to a combination of factors, including the number of cells assigned to each condition, the mean expression of the gene in the control, and the fraction of mono/bi-allelic loss. We hence designed a simulation-based power analysis taking into consideration these factors.

We have now included these additional analyses as a supplementary note (File S2).

3. There are very limited details on the modeling analysis to aid in future study design. Most importantly, no experimental validation is provided on whether the outcome of the modeling is actually predictive of the best parameters for designing future studies.

We apologize for the lack of clarity. We now provide more extensive documentation as well as tutorials to supplement the code and analysis notebooks that had previously been provided. We also provide more detailed examples of how our model matches the results we had observed in our experiment, and how it could be used for future design. The simulation framework takes as input the biallelic and monoallelic loss fractions, and computes the power of the test for detecting differential gene expression for varying numbers of treatment cells as well as varying mean log normalized expression of the gene in the control. We have now included results for the power analysis at the observed mono- and bi-allellic loss fractions for each of the Msh2-targeting and Tdgf1-targeting gRNAs.

We show here how our power analysis agreed with our experimental observations for Msh2-targeting gRNAs (Figure 5, middle column, reproduced below). We had been unable to observe differential expression of Msh2 without transcript-targeted sequencing in cells containing Msh2-targeting gRNAs. This is explained by our power analysis, which shows that given Msh2’s log normalized expression in the control (black vertical line), no number of treatment cells enables detection. Furthermore, we had also observed in our data that differential expression of Msh2 with transcript-targeted sequencing (Msh2*, as indicated by the red line) could be detected for MshSC1 and MshSC3. Our power analysis indicates this to be the expected result, as the minimum number of cells required to detect differential expression of Msh2* (green dashed line) is lower than the actual number of cells observed (black horizontal line) only for MshSC1 and MshSC3, but not MshSC2. Similarly, for Tdgf1-targeting gRNAs, our power analysis suggests the study is well-powered to detect differential expression of Tdgf1 despite the lower rate of mono- and bi-allelic loss, due to the relatively high baseline expression of Tdgf1 and relatively high number of cells collected.

Hence, for future experimental design, experimentalists should plan accordingly for the minimum required number of cells given the mean log normalized expression of their genes of interest. Furthermore, for low-expressing genes like Msh2, transcript-targeted sequencing is required to enable detection. Finally, given that power to detect low-expressing genes is extremely low, the number of treatment cells could be used to determine the threshold of independent filtering (as in Bourgon, Gentleman, and Huber, 2010) to reduce the multiple testing burden (Figure 5, right-most column).

We have also extended the power analysis to run with other differential expression methods that can be supplied by the user. User-supplied methods should take a Seurat single cell object and return p-values for differential expression. However, one limitation is that the simulation assumes that genes are treated independently by the differential expression method. To demonstrate how other differential expression methods can be used for the power analysis, we have included an example using MAST (without empirical bayes regularization of the gene variance so that the model treats each gene independently). In our simulations, we observe that MAST is more sensitive than the Wilcoxon test, as we would expect (S9 Fig).

4. The manuscript claims that this method has high multiplexing potential and could be used to study a large number of regulatory elements, however this potential is not demonstrated in the manuscript, rather the method seems to have limited sensitivity and requires a relatively large number of cells to detect allelic events, which may not be amenable to high multiplexing studies.

We apologize again for the lack of clarity. While in theory multiplexing CRISPR mutational screens could be used to study a large number of regulatory elements, we found that the low rate of biallelic loss by CRISPR significantly reduced sensitivity for detecting downstream effects. We believe that our modeling is important in gauging the multiplexing capacity of this sort of approach. Because we demonstrate pAC-Seq in the presence of targeted gene detection, as new highly scalable scRNA-seq platforms continue to emerge, it may become increasingly feasible to profile a large number of regulatory elements in a large number of cells in a single experiment. We have tried to further clarify this point via the power analysis.

Minor comments.

Introduction: The first paragraph of the introduction should reference MPRA/STARR-seq studies that have provided answers to some of the specific questions highlighted: identify functional regulatory elements and their regulatory effects on gene expression, analyze the function of non-coding mutations.

We now discuss MPRA/STARR-seq in the introduction as suggested.

Figure 1B: In the caption please explain what the horizontal segment indicates.

The horizontal segments indicate gates for GFP+ and GFP- cells. We have added this information to the legend.

Line 149: How is the significant enrichment defined?

We used a one-sided t-test. We have added this information to the manuscript. All non-control gRNAs were significantly enriched in %Msh2-GFP- cells with respect to sgControl (p-value < 0.05).

Line 290: The manuscript states that “the data was insufficient to impute with confidence” allelic expression. Please specify why the data were insufficient. Would a different study design (e.g. number of cells analyzed) be more appropriate to ask this question. As this was one of the major goals of this new method, new experiments to address this limitation should be considered.

We now include plots (Figure S7B, reproduced below) showing the distribution of the log normalized expression expression of Msh2 and Tdgf1 in cells receiving Msh2-targeting gRNAs (above) and Tdgf1-targeting gRNAs (below) with transcript-targeted sequencing in unsorted wild-type experiment. We were unable to impute if cells had mono-allelic or bi-allelic loss as we did not observe clear tri-modal distributions indicating subpopulations corresponding to mono-allelic, bi-allelic and no-loss. Detecting if cells contained mono or bi-allelic loss was not a major goal of this study. Instead, we used the observed mono or bi-allelic loss to guide parameter selection for the power analysis.

Reviewer #3: Overall:

Yeo et al present highly creative, thorough, and well-written work. They successfully demonstrate a novel gRNA-capture single-cell RNA-seq method with high editing efficiency and high single-cell capture rates. Furthermore, they pursue a sequence-interruption based perturbation of the noncoding genome, a risky yet required step in the field’s application of single-cell perturbation screens to study noncoding elements. Overall, the work is thorough, detailed, and advances the field. However, the manuscript lacks some discussion of 1) why such they detect such a low rate of gene expression reduction, and 2) the pros/cons of targeted capture single-cell RNA seq in these screens. I recommend acceptance into PloS Computational Biology with these two additional discussions and other minor changes.

We would like to thank reviewer #3 for their constructive comments.

Major

1) The authors cite lack of signal according to single-cell RNA’s lack of power to detect [Lines 171-174; Fig S1; Line 388-389.]. However, though it is implied, I think this should be clearly stated that this is a) likely due to the very small effect sizes that noncoding sequence changes of < 50 basepairs will have upon enhancers/promoters (this could be phrased in citations from the MPRA field, the length of TFBSs, etc) and b) the diverse indel editing outcomes from one gRNA adds an extreme amount of noise to their assay. Even if a small sequence change has an effect (as in A) it is very unlikely it would be detectable given the inability to genotype the sequence change per cell (as in B). Please add discussion clarifying this, and why it could underlie the detected <5% loss of target gene expression.

We have now included a discussion of the reasons underlying the low rate of biallelic loss, and included references to MPRA.

2) As mentioned in lines 240-243, the authors are performing a targeted single-cell RNA seq experiment. Please clarify this earlier in the work (ideally the abstract): especially now that 10x Genomics offers custom targeted single-cell RNA-seq panels (https://www.10xgenomics.com/products/targeted-gene-expression). I applaud the authors for including it in their Fig 5 simulation, as this will be very useful for future users.

We have now included a reference to 10X’s targeted scRNA-seq product.

Minor

Lines 57: Xie et al (Mosaic-seq) should be cited in addition to Gasperini et al. Additionally, another flaw of CRISPRi is it is unknown whether the KRAB-repression accurately perturbs enhancer/promoter function.

We have included a citation to Xie et al. (2017) and extended the discussion on CRISPRi.

Figure 1A: For clarity, please include a clearer diagram of the construct, ideally with actual letters ie a portion of the U6 promoter, ~20Ns, the actual hairpin sequence, and the 25xAs, and the Pol3 termination sequence.

We have included a clearer diagram of the construct (Figure 1a, reproduced below). Highlighted regions constitute the gRNA transcript, while unhighlighted regions flanking the gRNA transcript are part of the plasmid but not the transcript. The FE-modified gRNA hairpin is as in Chen et al. 2014.

Use of “protospacer” vs “spacer”: If I am not mistaken, the ‘protospacer’ sequence refers to the in-genome CRISPR-target DNA sequence (hence its use of the term ‘protospacer adjacent motif’). The “spacer” is the sequence included on the actual guide-RNA itself. Please verify and correct this use in the manuscript ie “We then cloned three GFP-targeting protospacers into either the wildtype gRNA “ changed to “three GFP-targeting spacers”. (Less an issue with the manuscript and more with confusion in the field itself).

Line 80: Polyadenine spelling typo.

You are correct, and we apologize for the mistake in nomenclature. We have replaced it with spacer and corrected the typo.

Fig 1: This is a higher editing rate than I would expect. Were the GFP gRNAs used in Fig1 newly designed or previously published? Please clarify either gRNA design or cite. Additionally please clarify in the Materials & Methods a) the protocol used to Sanger edits (was PCR used? Due to length bias, this can enrich for deletion-bearing fragments depending on the protocol) and b) the length of time between gRNA delivery and phenotyping.

We consistently achieve >99% GFP loss upon stable selection (>4 days) for cells receiving Cas9 and GFP-targeting gRNA. We note that often labs do not perform selection for integrated Cas9 and gRNA, which can lower the observed mutation efficiency. The gRNAs used were designed for this study and are listed in Table S3. Regarding Sanger sequencing, we performed genomic DNA isolation 1-2 weeks after transfection and performed PCR amplicon sequencing using primers listed in Table S3. Because indels are predominantly short (<20-nt), we do not think there is likely to be strong selection for deletion-bearing fragments. We have added this information to the methods.

Figure 3B: Please provide a similar figure showing plotting not just an individual gRNA but of any gRNAs in the population (ie plot each cell by its most-abundant gRNA, and do not limit which gRNA it might be on the graph). Additionally, please include a brief discussion for why the gRNA count-fraction is < 0.5, rather than closer to 1 given there should only be 1 gRNA per cell.

We have now included additional plots showing gRNA count fraction vs. the log total gRNA counts in each cell in the sorted population for MshSC1, the control gRNA, as well as the most abundant gRNA in each cell (S4A Fig, reproduced below). When log total gRNA count is high, it is straightforward to distinguish between a signal population (i.e. cells that contain the gRNA), and a noise population. We observed that the mean count fraction of these components (corresponding to the binomial probability of each component) differed between gRNAs-- for example, the mean count fraction of both noise and signal components are higher for the control gRNA (center) than for MshSC1 (left). As such, it is difficult to identify if a cell contains a particular gRNA by looking only at the count fraction of the most abundant gRNA (right). This motivates fitting the binomial count model for each gRNA independently.

Although models are fit per gRNA, of the 15000 cells with the highest total gRNA counts in the sorted population, 10738 (71.6%) cells were assigned 1 gRNA, while only 274 (1.83%) cells were assigned more than 1 gRNA. Results were similar for the unsorted population, where 9995 (66.7%) cells were assigned 1 gRNA, and only 237 (1.6%) of cells were assigned more than 1 gRNA. Cell barcodes to which 1 gRNA was assigned are then cross-referenced with the cell barcodes of the high-quality transcriptomes to determine the detection rate (see next comment). We believe the noise component could be as a result of technical artefacts in the gRNA mapping pipeline, which could be reduced via a more stringent mapping pipeline that also implements better cell-barcode and UMI error-correction.

Figure 3C: Perhaps I am missing it, but please include the number of cells that do not have any gRNA assigned to them. This will clarify the % assignment rates in Line 229 (75.3% and 91.2%) for each experiment. Why do these assignment rates differ between the experiments?

In the sorted population, 9808/13016 (75.3%, 3208 unassigned) of high quality transcriptomes were assigned a gRNA. In the unsorted population, 8887/9746 (91.2%, 859 unassigned) of high quality transcriptomes were assigned a gRNA. Here, high quality transcriptomes refer to the cells retained by 10X. We believe that assignment rates could differ due to differences in transfection efficiency.

Line 481: The sequence capture method listed here is complicated. In comparison, did you attempt a simpler PCR-enrichment in parallel (as performed in citation 22)? Which would you recommend for pAC-seq?

We did not compare our sequence capture method with a PCR-enrichment method, so we cannot comment on the relative efficiency. We believe there is value in having different methods to identify gRNAs in single-cell RNA-seq, and as such methods are rapidly proliferating, we believe that future effort could be spent comparing their properties.

Figure 5: This simulation is a highly useful tool for the field, and I commend the authors for including it.
